# A monograph of the Xyleborini (Coleoptera, Curculionidae, Scolytinae) of the Indochinese Peninsula (except Malaysia) and China

**DOI:** 10.3897/zookeys.983.52630

**Published:** 2020-11-03

**Authors:** Sarah M. Smith, Roger A. Beaver, Anthony I. Cognato

**Affiliations:** 1 Department of Entomology, Michigan State University, 288 Farm Lane, East Lansing, Michigan 48824, USA Michigan State University East Lansing United States of America; 2 161/2 Mu 5, Soi Wat Pranon, T. Donkaew, A. Maerim, Chiangmai 50180, Thailand Unaffiliated Chiangmai Thailand

**Keywords:** ambrosia beetles, biodiversity, new combinations, new species, new synonymy, Oriental region, Scolytidae, taxonomy

## Abstract

The Southeast Asian xyleborine ambrosia beetle fauna is reviewed for the first time. Thirty-four genera and 315 species are reviewed, illustrated, and keyed to genera and species. Sixty-three new species are described: *Amasa cycloxyster***sp. nov.**, *Amasa galeoderma***sp. nov.**, *Amasa gibbosa***sp. nov.**, *Amasa lini***sp. nov.**, *Amasa tropidacron***sp. nov.**, *Amasa youlii***sp. nov.**, *Ambrosiophilus caliginestris***sp. nov.**, *Ambrosiophilus indicus***sp. nov.**, *Ambrosiophilus lannaensis***sp. nov.**, *Ambrosiophilus papilliferus***sp. nov.**, *Ambrosiophilus wantaneeae***sp. nov.**, *Anisandrus achaete***sp. nov.**, *Anisandrus auco***sp. nov.**, *Anisandrus auratipilus***sp. nov.**, *Anisandrus congruens***sp. nov.**, *Anisandrus cryphaloides***sp. nov.**, *Anisandrus feronia***sp. nov.**, *Anisandrus hera***sp. nov.**, *Anisandrus paragogus***sp. nov.**, *Anisandrus sinivali***sp. nov.**, *Anisandrus venustus***sp. nov.**, *Anisandrus xuannu***sp. nov.**, *Arixyleborus crassior***sp. nov.**, *Arixyleborus phiaoacensis***sp. nov.**, *Arixyleborus setosus***sp. nov.**, *Arixyleborus silvanus***sp. nov.**, *Arixyleborus sittichayai***sp. nov.**, *Arixyleborus titanus***sp. nov.**, *Coptodryas amydra***sp. nov.**, *Coptodryas carinata***sp. nov.**, *Coptodryas inornata***sp. nov.**, *Cyclorhipidion amasoides***sp. nov.**, *Cyclorhipidion amputatum***sp. nov.**, *Cyclorhipidion denticauda***sp. nov.**, *Cyclorhipidion muticum***sp. nov.**, *Cyclorhipidion obesulum***sp. nov.**, *Cyclorhipidion petrosum***sp. nov.**, *Cyclorhipidion truncaudinum***sp. nov.**, *Cyclorhipidion xeniolum***sp. nov.**, *Euwallacea geminus***sp. nov.**, *Euwallacea neptis***sp. nov.**, *Euwallacea subalpinus***sp. nov.**, *Euwallacea testudinatus***sp. nov.**, *Heteroborips fastigatus***sp. nov.**, *Heteroborips indicus***sp. nov.**, *Microperus latesalebrinus***sp. nov.**, *Microperus minax***sp. nov.**, *Microperus sagmatus***sp. nov.**, *Streptocranus petilus***sp. nov.**, *Truncaudum bullatum***sp. nov.**, *Xyleborinus cuneatus***sp. nov.**, *Xyleborinus disgregus***sp. nov.**, *Xyleborinus echinopterus***sp. nov.**, *Xyleborinus ephialtodes***sp. nov.**, *Xyleborinus huifenyinae***sp. nov.**, *Xyleborinus jianghuansuni***sp. nov.**, *Xyleborinus thaiphami***sp. nov.**, *Xyleborinus tritus***sp. nov.**, *Xyleborus opacus***sp. nov.**, *Xyleborus sunisae***sp. nov.**, *Xyleborus yunnanensis***sp. nov.**, *Xylosandrus bellinsulanus***sp. nov.**, *Xylosandrus spinifer***sp. nov.**. Thirteen new combinations are given: *Ambrosiophilus consimilis* (Eggers) **comb. nov.**, *Anisandrus carinensis* (Eggers) **comb. nov.**, *Anisandrus cristatus* (Hagedorn) **comb. nov.**, *Anisandrus klapperichi* (Schedl) **comb. nov.**, *Anisandrus percristatus* (Eggers) **comb. nov.**, *Arixyleborus resecans* (Eggers) **comb. nov.**, *Cyclorhipidion armiger* (Schedl) **comb. nov.**, *Debus quadrispinus* (Motschulsky) **comb. nov.**, *Heteroborips tristis* (Eggers) **comb. nov.**, *Leptoxyleborus machili* (Niisima) **comb. nov.**, *Microperus cruralis* (Schedl) **comb. nov.**, *Planiculus shiva* (Maiti & Saha) **comb. nov.**, *Xylosandrus formosae* (Wood) **comb. nov.** Twenty-four new synonyms are proposed: *Ambrosiophilus osumiensis* (Murayama, 1934) (= *Xyleborus nodulosus* Eggers, 1941 **syn. nov.**); *Ambrosiophilus subnepotulus* (Eggers, 1930) (= *Xyleborus cristatuloides* Schedl, 1971 **syn. nov.**); *Ambrosiophilus sulcatus* (Eggers, 1930) (= *Xyleborus sinensis* Eggers, 1941 **syn. nov.**; = *Xyleborus sulcatulus* Eggers, 1939 **syn. nov.**); *Anisandrus hirtus* (Hagedorn, 1904) (= *Xyleborus hirtipes* Schedl, 1969 **syn. nov.**); *Cnestus protensus* (Eggers, 1930) (= *Cnestus rostratus* Schedl, 1977 **syn. nov.**); *Cyclorhipidion bodoanum* (Reitter, 1913) (= *Xyleborus misatoensis* Nobuchi, 1981 **syn. nov.**); *Cyclorhipidion distinguendum* (Eggers, 1930) (= *Xyleborus fukiensis* Eggers, 1941 **syn. nov.**; = *Xyleborus ganshoensis* Murayama, 1952 **syn. nov.**); *Cyclorhipidion inarmatum* (Eggers, 1923) (= *Xyleborus vagans* Schedl, 1977 **syn. nov.**); *Debus quadrispinus* (Motschulsky, 1863) (= *Xyleborus fallax* Eichhoff, 1878 **syn. nov.**); *Euwallacea gravelyi* (Wichmann, 1914) (= *Xyleborus barbatomorphus* Schedl, 1951 **syn. nov.**); *Euwallacea perbrevis* (Schedl, 1951) (= *Xyleborus molestulus* Wood, 1975 **syn. nov.**; *Euwallacea semirudis* (Blandford, 1896) (= *Xyleborus neohybridus* Schedl, 1942 **syn. nov.**); *Euwallacea sibsagaricus* (Eggers, 1930) (= *Xyleborus tonkinensis* Schedl, 1934 **syn. nov.**); *Euwallacea velatus* (Sampson, 1913) (= *Xyleborus rudis* Eggers, 1930 **syn. nov.**); *Microperus kadoyamaensis* (Murayama, 1934) (= *Xyleborus pubipennis* Schedl, 1974 **syn. nov.**; =*Xyleborus denseseriatus* Eggers, 1941 **syn. nov.**); *Stictodex dimidiatus* (Eggers, 1927) (=*Xyleborus dorsosulcatus* Beeson, 1930 **syn. nov.**); *Webbia trigintispinata* Sampson, 1922 (= *Webbia mucronatus* Eggers, 1927 **syn. nov.**); *Xyleborinus artestriatus* (Eichhoff, 1878) (= *Xyelborus angustior* [*sic*] Eggers, 1925 **syn. nov.**; = *Xyleborus undatus* Schedl, 1974 **syn. nov.**); *Xyleborinus exiguus* (Walker, 1859) (= *Xyleborus diversus* Schedl, 1954 **syn. nov.**); *Xyleborus muticus* Blandford, 1894 (= *Xyleborus conditus* Schedl, 1971 **syn. nov.**; = *Xyleborus lignographus* Schedl, 1953 **syn. nov.**). Seven species are removed from synonymy and reinstated as valid species: *Anisandrus cristatus* (Hagedorn, 1908), *Cyclorhipidion tenuigraphum* (Schedl, 1953), *Diuncus ciliatoformis* (Schedl, 1953), *Euwallacea gravelyi* (Wichmann, 1914), *Euwallacea semirudis* (Blandford, 1896), *Microperus fulvulus* (Schedl, 1942), *Xyleborinus subspinosus* (Eggers, 1930).

## Introduction

Xyleborine ambrosia beetles (Curculionidae: Scolytinae) occur throughout the forested regions of the world with the highest diversity occurring in the tropical and subtropical regions (Hulcr et al. 2015). It is hypothesized that xyleborines originated in the Orient given the region’s high species and generic diversity (Hulcr et al. 2015; Cognato et al. 2018). Since their origin 20 million years ago, xyleborines have successfully dispersed across the world, sparking radiations of species wherever colonists landed (especially the Neotropics) (Jordal and Cognato 2012; Cognato et al. 2018). There are approximately 1200 species currently recognized and they comprise the largest scolytine tribe, representing approximately 20% of total diversity. However, this total diversity has yet to be fully realized with an estimated 25–75% awaiting discovery and description (Hulcr et al. 2015; Smith et al. 2017a) and approximately 30% in tropical Asia. The biology of these beetles makes them extremely well-suited for colonization (Jordal et al. 2001; Gohli et al. 2016). They have a strongly female-skewed haplodiploid mating system with extreme inbreeding (Kirkendall 1993; Kirkendall et al. 2015). Usually, females mate with a brother before leaving the natal gallery. If unmated, a female lays haploid eggs, which develop into males. The adult male, which is dwarfed and flightless, may mate with his mother who then produces diploid eggs which develop into females. These beetles also cultivate symbiotic fungal gardens within tunnels they bore into trees. The beetles have specialized body parts (mycangia) which fill with fungi and provide secure transport of the fungi to new habitats. Mycangia are invaginated pouches which occur in the head near the mandibles, pronotum/mesonotum, and in the elytral bases (Beaver 1989). The type of mycangium tends to be taxon specific and several fungal genera form specific symbiotic relationships with xyleborine genera (Beaver 1989; Hulcr and Cognato 2010; Hulcr and Stelinski 2017). Thus, upon arrival at a new location, even an unmated female provisioned with symbiotic fungi can produce a fungal garden and a family which can eventually grow into a population of beetles. This great colonizing potential has led to the accidental introduction through global trade of 31 and 12 species to North America and Europe, respectively (Kirkendall and Faccoli 2010; Garonna et al. 2012; Terekhova and Skrylnik 2012; Dodelin 2018; Rabaglia et al. 2019, 2020a). Most of these introduced species were native to SE Asia (Haack and Rabaglia 2013). In North America, three SE Asian species *Euwallacea fornicatus* (Eichhoff, 1868), *E. kuroshio* (Gomez & Hulcr, 2018), and *Xyleborus glabratus* Eichhoff, 1877, have caused major economic and ecological damage to trees in urban/suburban and natural areas (Eskalen et al. 2013; Boland 2016; Carillo et al. 2016; Hughes et al. 2017; Coleman et al. 2019).

Taxonomic knowledge of xyleborines is mostly limited to alpha-level taxonomy that began in earnest with the description of *Xyleborus* by Eichhoff (1864), and progressed with major contributions from Eichhoff, Blandford, Eggers, Schedl, Browne, Murayama, Nobuchi and Wood (Wood and Bright 1992). Given the unique aspects of xyleborine biology (as described above), morphological aberrations that occur within a single foundress can rapidly propagate among progeny which may ultimately grow to population levels. This intraspecific variation has historically been problematic and confounded the delineation of species limits. This has led to numerous subjective synonyms for many species, especially widespread taxa (e.g., *Xyleborus affinis* Eichhoff, 1868, *X. perforans* (Wollaston, 1857), *Xyleborinus exiguus* (Walker, 1859). Many species were described from short series or singletons which insufficiently assessed intraspecific variation (e.g., *Euwallacea fornicatus* complex). Single individuals of multiple species from a variety of locations often seemingly formed a continuous spectrum of variation which has led to their synonymization (Hulcr and Cognato 2013). Generic taxonomy began with the description of *Eccoptopterus* (Motschulsky, 1863), *Xyleborus* (Eichhoff, 1864), and *Amasa* (Lea, 1894) and by 1990, 24 genera had been described through the efforts of Blandford, Hagedorn, Hopkins, Reitter, and Sampson (Wood and Bright 1992). The 2000’s brought the use of molecular phylogenies to identify monophyletic groups and elucidate taxon limits (Gomez et al. 2018b; Cognato et al. 2019, 2020a; Smith et al. 2020). Currently, there are 42 recognized xyleborine genera with the likely recognition of additional genera given the extensive morphological variation observed in the polyphyletic *Xyleborus* (Cognato et al. 2020a). Comprehensive species reviews and identification keys are limited to generic level studies (e.g., Beaver and Hulcr 2008; Beaver 2010; Dole and Cognato 2010; Smith 2017; Beaver et al. 2019) and faunal reviews of geographic regions: North and Central America (Wood 1982), China (Yin et al. 1984), Europe (Pfeffer 1994), South America (Wood 2007), India (Maiti and Saha 2004), Papua New Guinea (Hulcr and Cognato 2010), Taiwan (Beaver and Liu 2010), Thailand (Beaver et al. 2014) and the West Indies (Bright 2019). These geographic reviews and monographs provide a necessary foundation for understanding the xyleborine fauna, but quickly become outdated as new species are found and taxonomic changes made. Nevertheless, the keys provide a gateway into identifying this economically important group of beetles. A comprehensive publication for SE Asia is conspicuously absent and lack of this resource has caused delays in identifying non-native species or mistaken identities (Smith and Cognato 2015; Smith et al. 2017b; Hoebeke et al. 2018).

Given that SE Asia species are intercepted at US and other ports every year and have proven pestiferous (Haack and Rabaglia 2013), a review and key for the xyleborine fauna of SE Asia is critically needed (Smith and Cognato 2015; Smith et al. 2017b; Rabaglia et al. 2019). In 2016, AIC was funded to create identification tools including DNA barcodes and a Lucid key of this fauna (Smith et al. 2019a; Cognato et al. 2020b). As indicated by the title, the geographic region of study is awkward; it focuses on the Indochinese Peninsula (Cambodia, Myanmar, Laos, Thailand, Vietnam) excluding Malaysia and insular SE Asian countries, and includes subalpine Himalayan areas (Northern India, Nepal, Bhutan), Bangladesh, China, and Taiwan. This was intentional in order to focus the study on the region of greatest potential for harboring future pests in non-native regions outside the equatorial tropical rain forest belt (McCullough et al. 2006; Haack and Rabaglia 2013). As a result of creating these identification tools, a review of the fauna was accomplished, which is detailed in this publication.

## Materials and methods

Examined specimens came from our own collections, fieldwork and through loans from several institutions. All descriptions, keys and diagnoses are based on females as males are largely unknown, rarely encountered, and not often present without a female of the same species. Type material was examined by all authors. Specimens were assembled and examined from the following entomological collections by one or more authors:

**BPBM**Bernice P. Bishop Museum, Honolulu, USA;

**CASC**California Academy of Sciences, San Francisco, USA;

**CSLC** Ching-Shan Lin collection, Chang Hua, Taiwan;

**FRI**Forest Research Institute, Dehra Dun, India;

**HNHM**Hungarian Natural History Museum, Budapest, Hungary;

**IRSNB**Institut Royale des Sciences Naturelles, Brussels, Belgium;

**IZAS**Institute of Zoology, Chinese Academy of Sciences, Beijing, China;

**FSCA**Florida State Collection of Arthropods, Gainesville, USA;

**LYLC** Lan-Yu Liu collection, Yilan, Taiwan;

**MCG**Museo Civico di Storia Naturale “Giacomo Doria”, Genova, Italy;

**MCZ**Museum of Comparative Zoology, Cambridge, USA;

**MFNB** Museum für Naturkunde, Berlin, Germany;

**MIZ** Museum and Institute of Zoology, Polish Academy of Sciences, Warsaw, Poland;

**MNHN**Muséum National d'Histoire Naturelle, Paris, France;

**MNHP** Museum of Natural History, Prague, Cechia;

**MSUC**Michigan State University Arthropod Research Collection, East Lansing, USA;

**NHMB**Natural History Museum, Basel, Switzerland;

**NHMUK**Natural History Museum, London, UK;

**NHMW**Naturhistorisches Museum Wien, Austria;

**NIAES**National Institute for Agro-Environmental Sciences, Tsukuba, Japan;

**NKME**Naturkunde Museum, Erfurt, Germany;

**NMNH**National Museum of Natural History, Smithsonian Institution, Washington, D.C., USA;

**OMNH**Sam Noble Oklahoma Museum of Natural History, University of Oklahoma, Norman, USA;

**PPST** Plant Protection Station, Tokyo, Japan;

**QDAFB** Queensland Department of Agriculture and Fisheries, Brisbane, Australia;

**QSBG** Queen Sirikit Botanical Garden, Chiang Mai, Thailand;

**RABC** Roger A. Beaver collection, Chiang Mai, Thailand;

**RIFID** Research Institute of Forest Insect Diversity, Namyangju, South Korea;

**RJRC** Robert J. Rabaglia collection, Annapolis, USA;

**RMNH**Naturalis Biodiversity Centre, Leiden, Netherlands;

**SDEI**Senckenberg Deutsches Entomologisches Institut, Müncheberg, Germany;

**SEMC**University of Kansas Biodiversity Institute, Manhattan, USA;

**SMNH**Swedish Museum of Natural History, Stockholm, Sweden;

**SSC** Sunisa Sanguansub collection, Khampaengsaen, Thailand;

**TARI**Taiwan Agricultural Research Institute, Taichung, Taiwan;

**UFFE** University of Florida, Forest Entomology Laboratory, Gainesville, USA;

**UHZM** Universität Hamburg – Zoological Museum, Hamburg, Germany;

**VNMN**Vietnam National Museum of Nature, Hanoi, Vietnam;

**ZFMK**Zoological Research Museum Alexander Koenig, Bonn, Germany;

**ZIN**Zoological Institute of the Russian Academy of Sciences, St. Petersburg, Russia;

**ZMMU**Zoological Museum at Moscow State University, Moscow, Russia;

**ZSI**Zoological Survey of India, Calcutta, India.

All the primary literature as well as types of nearly all 280 species and many of their synonyms known prior to this study were obtained so to assure correct identity of examined specimens. We employed a species concept *sensu*Hey (2006) and Yeates (2011), that is, species are hypotheses of evolutionary lineages, which are tested with available data. For most species, combinations of morphologically diagnostic characters were taken as evidence for species. In other cases, monophyly based on phylogenies derived from mitochondrial cytochrome oxidase I (COI) and nuclear CAD DNA sequences provided direct evidence of a species equating with an evolutionary lineage (Cognato et al. 2019, 2020b). Decisions to recognize monophyletic groups as species was based on the presence of morphological diagnostic characters and the demonstration of > 10% COI and > 2% CAD average pairwise uncorrected “p” distance between sister clades (Cognato et al. 2020b).

Specimens were primarily photographed by SMS with some by Rachel Osborn (MSU) with a Visionary Digital Passport II system (Dun Inc., Palmyra, VA) using a Canon EOS 5D Mark II, 65.0 mm Canon Macro photo lens, two Dynalite (Union, NJ) MH2015 road flash heads, Dynalite RoadMax MP8 power pack and a Stack Shot (Cognisys, Inc, Traverse City, MI). Montage images were assembled using Helicon Focus Mac Pro 6.7.1 (Helicon Soft, Kharkov, Ukraine). Additional photos were contributed by Wisut Sittichaya (Prince of Songkhla University) and AIC (methods detailed in Smith et al. 2019a).

Specimens were examined using Leica (Wetzlar, Germany) MZ6 and MZ16 stereomicroscopes and illuminated with an Ikea Jansjö LED work lamp (Delft, Netherlands). Length was measured from pronotum apex to the apex of the declivity and a maximum of five specimens per species were measured. Pedicel is not included in the number of funicle segments, following Hulcr and Smith (2010). Taxa are listed alphabetically by genus and then by species within each genus. Unless stated as examined, the location of type species is not given but can be found in Wood and Bright (1992), Bright and Skidmore (1997, 2002), or Bright (2014). This catalog and its supplements contain additional references on the biology of many of the included species. Distribution data were collected from: Wood and Bright 1992; Beaver and Liu 2010; Knížek 2011; Beaver et al. 2014; Zheng et al. 2017; Mandelshtam et al. 2018; Smith et al. 2018b, c; Lin et al. 2019; Sittichaya et al. 2019; Park et al. 2020; Li et al. 2020; Rabaglia et al. 2020b) and other sources are given for each species. New distribution records are denoted with an asterisk.

## Terminology

**Figure 1.**
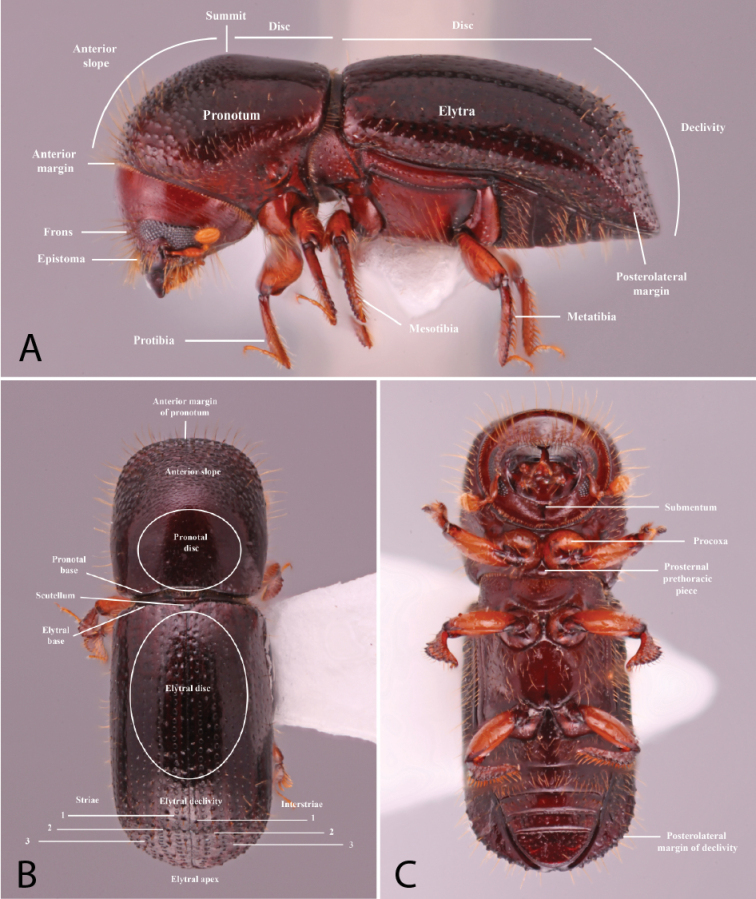
Anatomical terminology illustrated on *Euwallacea sibsagaricus***A** lateral habitus **B** dorsal habitus **C** ventral habitus.

**Figure 2.**
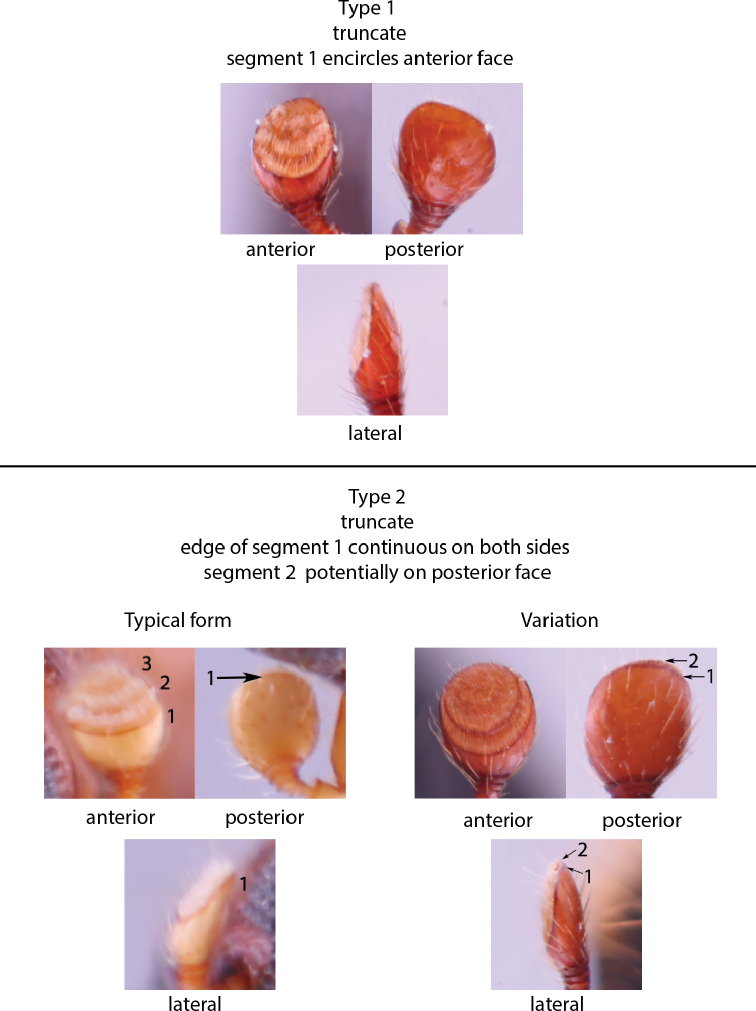
Obliquely truncate antennal clubs, types 1 and 2 (Hulcr et al. 2007). Type 1, *Anisandrus percristatus*; typical type 2, *Xyleborus affinis*; variation of type 2, *Hadrodemius comans*.

**Figure 3.**
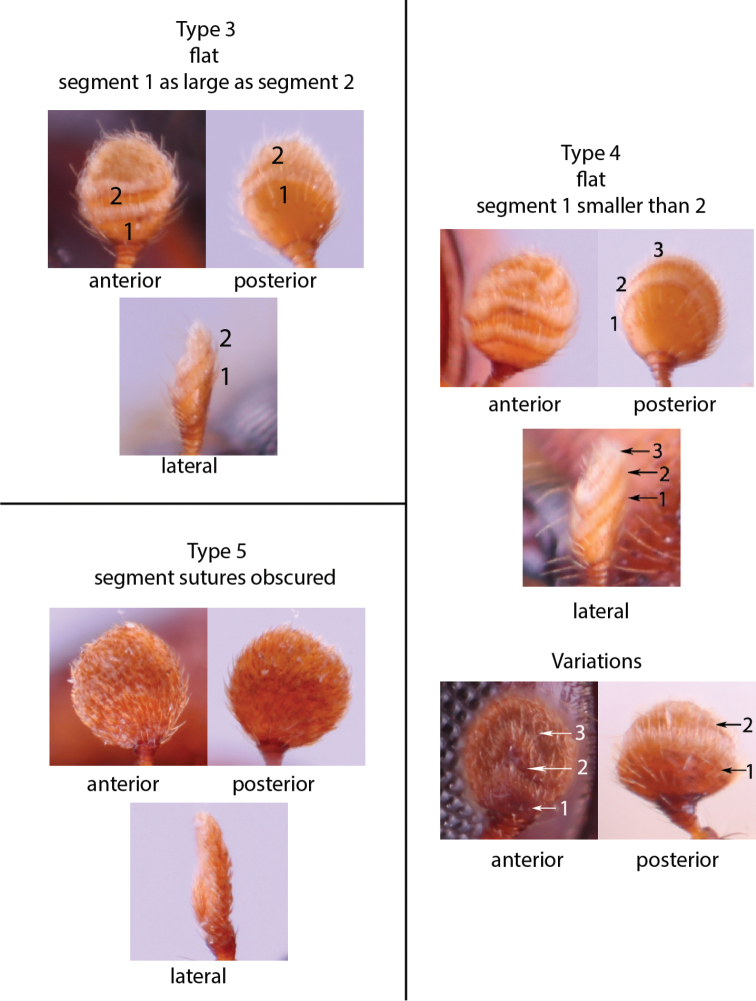
Flattened antennal clubs, types 3, 4, and 5 (Hulcr et al. 2007). Type 3, *Euwallacea interjectus*; typical type 4, *Amasa schlichii*; variation type 4 (anterior), *Fortiborus major*; variation type 4 (posterior), *Schedlia sumatrana*; type 5, *Amasa beesoni*.

**Figure 4.**
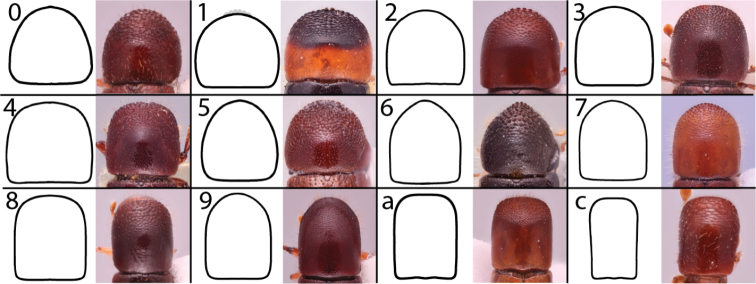
Dorsal pronotal types. Type 0, *Heteroborips seriatus*; type 1 rounded, *Cnestus gravidus*; type 2 basic and parallel-sided, *Amasa gibbosa*; type 3 subquadrate with anterolateral corners slightly prominent, *Cyclorhipidion amasoides*; type 4 quadrate with anterolateral corners conspicuous and sides almost parallel, *Euwallacea destruens*; type 5 conical and elongate, *Leptoxyleborus sordicauda*; type 6 strongly conical, *Anisandrus cryphaloides*; type 7 rounded frontally and long, *Tricosa cattienensis*; type 8 elongate and subquadrate or quadrate, *Euwallacea piceus*; type 9 long and rounded frontally, *Debus amphicranoides*; type a long and quadrate frontally, *Webbia duodecimspinata*; type c conspicuously elongate and quadrate frontally, *Streptocranus bicuspis*. Drawings modified from Hulcr et al. 2007.

**Figure 5.**
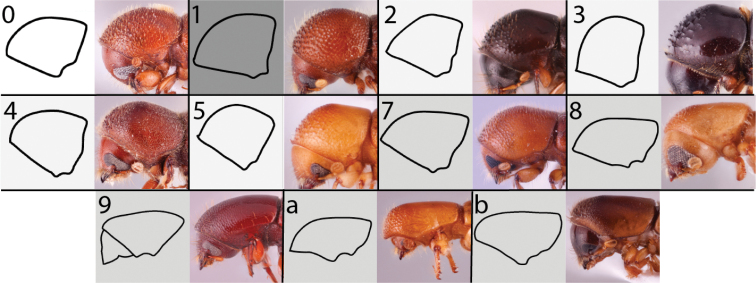
Lateral pronotal types. Type 0 basic, *Xylosandrus mancus*; type 1 uniformly rounded without distinct summit, *Ambrosiodmus rubricollis*; type 2 taller than basic, *Euwallacea perbrevis*; type 3 short and tall, *Anisandrus percristatus*; type 4 robust with summit moved anteriad, *Schedlia sumatrana*; type 5 robust, subquadrate or rounded, *Diuncus haberkorni*; type 7 disc as long or slightly longer than anterior slope, *Tricosa cattienensis*; type 8 disc much longer than anterior slope, *Cryptoxyleborus stenographus*; type 9 anterior slope much longer than disc, *Debus amphicranoides*; type a very long ‘hooded frontally’, *Streptocranus mirabilis*; type b long flattened and bulging frontally, *Webbia duodecimspinata*. Drawings modified from Hulcr et al. 2007.

**Figure 6.**
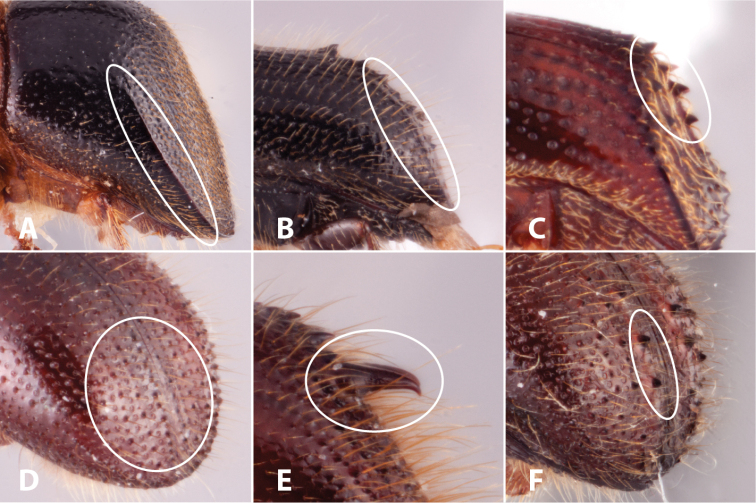
Illustrated glossary of terms **A** carina **B** costa **C** denticles **D** granules **E** spine **F** tubercles.

Anatomical terminology is illustrated in Figure 1. Antennal club types (Figs 2, 3) and pronotal types from dorsal (Fig. 4) and lateral (Fig. 5) views follow those in Hulcr et al. (2007). The following commonly used terms are here defined:

alutaceous with fine, leather-like reticulation;

asperity(-ies) small flat denticle-like structures frequently arranged in rows or confined to specific areas;

carina a sharply elevated ridge or keel, not necessarily high or acute (Fig. 6A);

costa a more gradually elevated ridge that is rounded at its crest, without a sharp appearance (Fig. 6B);

declivity/declivital the downward slope of the elytra/pertaining to the declivity;

denticle a small tooth, the sides of which are equal, and the tip is above the middle of the base (Fig. 6C);

glabrous devoid of vestiture;

granule a small rounded protuberance, like a grain of sand (Fig. 6D);

opalescent showing varying colors, like an opal;

serrations row of asperities (flat denticles), a saw-like structure;

shagreened with a rough surface of closely set granules;

spine an elongate projection of the exoskeleton that is longer than its basal width (Fig. 6E);

summit highest point, used for pronotum and elytra, denotes the peak between pronotal frontal slope and disc, and between elytral disc and declivity;

tubercle a small knob-like or rounded protuberance of the exoskeleton (Fig. 6F);

unarmed without cuticular protuberances, e.g., granules, denticles, tubercles or spines;

vermiculate tortuous; marked by repeated twists, like worm tracks.

## Results and discussion

We identified 34 genera and 315 species as occurring in the study region. Sixty-three new species, 24 new synonyms and 13 new combinations were identified. Previously published records of two additional species were not confirmed as occurring in the region and are therefore considered dubious:

1. *Cnestus bicornis* (Eggers, 1923) is listed as occurring in India (Assam) (Wood and Bright 1992: 802) but a published Indian record was not found and no Indian specimens could be located. Occurrence in India is therefore doubtful and probably represents a misidentification of the morphologically similar *C. bicornioides* which does occur in India.

2. *Xyleborus aquilus* Blandford, 1894 was described from Japan and was previously reported from China (Fujian, Hunan, Sichuan), South Korea and Taiwan. Images of syntypes from NHMUK were compared to the description and diagnosis of Yin et al. (1984) in which the species is reported from China. The syntypes are of a *Xyleborus* species closely related to *X. festivus* Eichhoff, 1866 while Yin et al.’s description, illustration, and diagnosis represent an *Euwallacea* which we were unable to determine to species. Examination of Yin’s specimens in IZAS did not reveal any specimens bearing this name (You Li, pers. comm.). *Xyleborus aquilus* was reported from Korea by Murayama (1930) but no vouchered specimens have been found or since collected (Park et al. 2020). Beaver and Liu (2010) considered the Taiwan record dubious. It is very likely that *X. aquilus* is distributed only in Japan.

In part, this study relied on DNA based phylogenies to help resolve generic and species identities and designate species limits (Cognato et al. 2020b). The rubric of monophyly, a sequence difference threshold, and morphological diagnostic characters provided guidance for recognizing species given sufficient specimens, for example, *Xyleborus glabratus* (Cognato et al. 2019). These DNA based phylogenies and our examination of specimens revealed additional taxonomic problems. 1. *Coptodryas* Hopkins, 1915, *Cryptoxyleborus* Wood & Bright, 1992, *Microperus* Wood, 1980, *Xyleborus* and *Xylosandrus* Reitter, 1913 are likely not monophyletic (Cognato et al. 2020b). A more robust dataset including more genes and taxa will likely help resolve the monophyly for some genera. In other cases, reexamination of aberrant species may lead to the stabilization of genera (i.e. reassignment of generic placement for certain species) and recognition of new genera as with *Fraudatrix* Cognato, Smith & Beaver, 2020 and *Tricosa* Cognato, Smith & Beaver, 2020 (Cognato et al. 2020a). 2. Several *Euwallacea* species were para- or polyphyletic (Cognato et al. 2020b). These species (e.g., *E. andamanensis* Blandford, 1896) exhibit little morphological difference yet demonstrate > 12% COI nucleotide difference (Cognato et al. 2020b). Morphometric analyses as with the *E. fornicatus* complex may be necessary to help tease out cryptic species (Gomez et al. 2018b). 3. Some species are morphologically variable. For example, *Ambrosiophilus osumiensis* (Murayama, 1934), demonstrates < 7.5% COI nucleotide difference yet the morphological variation is associated with three species which we synonymized. These morphological characters, mainly the size, position, and number of declivital granules have been traditionally used to recognize species. Given these observations, other suspect variable species should be re-evaluated and caution given to future recognition of new species based on subtle morphological differences.

We discovered a total of 75 new species reported in this and associated publications (Smith et al. 2018c; Cognato et al. 2019, 2020a; Park et al. 2020) and additional new records for southern Thailand (Sittichaya et al. 2019). Given the wide range of some species distributions, examination of the broader region was necessary and as a result we identified new species and nomenclatural changes for Japan and Korea (Smith et al. 2018b; Park et al. 2020), and clarified species limits for some Indo-Malayan species that were incorrectly placed in synonymy (Smith et al. 2020). We also recognized a new species from insular SE Asia (Wang et al. 2020). Thus, this publication is the foundation for a future monograph of all the SE Asian fauna. Based on this study we estimate that 30% of the species are undescribed, but given that scolytine taxonomists have not collected in many areas especially Myanmar, Laos, Cambodia, and Philippines, the number of undiscovered species is likely greater. Also, generic taxonomy will likely continue to improve with delimitation and descriptions of new genera identified among *Xyleborus* species (Cognato et al. 2020b).

This study provides the first taxonomic review of xyleborine species occurring in mainland SE Asia and adjacent areas. The associated taxonomic tools, Lucid key, DNA sequences, and images complement this monograph and provide additional resources for species and generic identifications (Smith et al. 2019a; Cognato et al. 2020b). We consider the Lucid key and DNA database living “documents”, and as we continue to treat the Asian fauna, we will amend these tools with the goal of taxon completion.

### Checklist of the Xyleborini of Southeast Asia

***Amasa* Lea, 1894**

*Pseudoxyleborus* Eggers, 1930

*Anaxyleborus* Wood, 1980

*Amasa aspersa* (Sampson, 1921)

*Amasa beesoni* (Eggers, 1930)

*Amasa concitata* (Schedl, 1969a)

*Amasa cycloxyster* sp. nov.

*Amasa cylindrotomica* (Schedl, 1939b)

*Xyleborus semitruncatus* Schedl, 1942c

*Xyleborus truncatellus* Schedl, 1951a

*Xyleborus jucundus* Schedl, 1954

*Amasa eugeniae* (Eggers, 1930)

*Amasa galeoderma* sp. nov.

*Amasa gibbosa* sp. nov.

*Amasa lini* sp. nov.

*Amasa opalescens* (Schedl, 1937a)

*Amasa resecta* (Eggers, 1923)

*Xyleborus abruptus* Eggers, 1923

*Xyleborus opacicauda* Eggers, 1940

*Amasa schlichii* (Stebbing, 1907)

*Acanthotomicus truncatus* Stebbing, 1907

*Xyleborus glaber* Eggers, 1930

*Xyleborus uniseriatus* Eggers, 1936b

*Xyleborus verax* Schedl, 1939b

*Amasa tropidacron* sp. nov.

*Amasa versicolor* (Sampson, 1921)

*Amasa youlii* sp. nov.

***Ambrosiodmus* Hopkins, 1915a**

*Phloeotrogus* Motschulsky, 1863

*Brownia* Nunberg, 1963

*Ambrosiodmus asperatus* (Blandford, 1895)

*Xyleborus nepotulus* Eggers, 1923

*Xyleborus citri* Beeson, 1930

*Xyleborus nepotulomorphus* Eggers, 1936b

*Ambrosiodmus brunneipes* (Eggers, 1940)

*Ambrosiodmus conspectus* (Schedl, 1964b)

*Ambrosiodmus lewisi* (Blandford, 1894b)

*Ozopemon tuberculatus* Strohmeyer, 1912

*Xyleborus lewekianus* Eggers, 1923

*Xyleborus tegalensis* Eggers, 1923

*Ambrosiodmus minor* (Stebbing, 1907)

*Xyleborus crassus* Hagedorn, 1910a

*Ambrosiodmus rubricollis* (Eichhoff, 1876a)

*Xyleborus taboensis* Schedl, 1952b

*Xyleborus strohmeyeri* Schedl, 1975b

***Ambrosiophilus* Hulcr & Cognato, 2009**

*Ambrosiophilus atratus* (Eichhoff, 1876a)

*Xyleborus collis* Niisima, 1910

*Ambrosiophilus caliginestris* sp. nov.

*Ambrosiophilus consimilis* (Eggers, 1923), comb. nov.

*Ambrosiophilus cristatulus* (Schedl, 1953b)

*Ambrosiophilus indicus* sp. nov.

*Ambrosiophilus lannaensis* sp. nov.

*Ambrosiophilus latisulcatus* (Eggers, 1940)

*Ambrosiophilus osumiensis* (Murayama, 1934)

*Xyleborus metanepotulus* Eggers, 1939b

*Xyleborus nodulosus* Eggers, 1941b, syn. nov.

*Xyleborus pernodulus* Schedl, 1957

*Xyleborus hunanensis* Browne, 1983b

*Ambrosiophilus peregrinus* Smith & Cognato, 2015

*Ambrosiophilus papilliferus* sp. nov.

*Ambrosiophilus satoi* (Schedl, 1966b)

*Ambrosiophilus sexdentatus* (Eggers, 1940)

*Ambrosiophilus subnepotulus* (Eggers, 1930)

*Xyleborus cristatuloides* Schedl, 1971a, syn. nov.

*Ambrosiophilus sulcatus* (Eggers, 1930)

*Xyleborus sulcatulus* Eggers, 1939a, syn. nov.

*Xyleborus sinensis* Eggers, 1941b, syn. nov.

*Ambrosiophilus wantaneeae* sp. nov.

***Ancipitis* Hulcr & Cognato, 2013**

*Ancipitis puer* (Eggers, 1923)

*Xyleborus ceramensis* Schedl, 1937a

*Ancipitis punctatissimus* (Eichhoff), 1880

*Xyleborus spatulatus* Blandford, 1896b

***Anisandrus* Ferrari, 1867**

*Anisandrus achaete* sp. nov.

*Anisandrus apicalis* (Blandford, 1894b)

*Anisandrus auco* sp. nov.

*Anisandrus auratipilus* sp. nov.

*Anisandrus carinensis* (Eggers, 1923), comb. nov.

*Anisandrus congruens* sp. nov.

*Anisandrus cristatus* (Hagedorn, 1908), comb. nov., stat. res.

*Xyleborus fabricii* Schedl, 1964c

*Anisandrus cryphaloides* sp. nov.

*Anisandrus dispar* (Fabricius, 1792)

*Bostrichus brevis* Panzer, 1793

*Bostrichus thoracicus* Panzer, 1793

*Scolytus pyri* Peck, 1817

*Bostrichus tachygraphus* Sahlberg, 1836

*Bostrichus ratzeburgi* Kolenati, 1846

*Xyleborus ishidai* Niisima, 1909

*Anisandrus aequalis* Reitter, 1913

*Anisandrus swainei* Drake, 1921

*Xyleborus dispar rugulosus* Eggers, 1922

*Xyleborus cerasi* Eggers, 1937

*Xyleborus khinganensis* Murayama, 1943

*Anisandrus eggersi* (Beeson, 1930)

*Anisandrus feronia* sp. nov.

*Anisandrus geminatus* (Hagedorn, 1904)

*Anisandrus hera* sp. nov.

*Anisandrus hirtus* (Hagedorn, 1904)

*Xyleborus hagedorni* Stebbing, 1914

*Xyleborus hirtuosus* Beeson, 1930

*Xyleborus hagedornianus* Schedl, 1952d

*Xyleborus tectonae* Nunberg, 1956

*Xyleborus hirtipes* Schedl, 1969b, syn. nov.

*Xyleborus taiwanensis* Browne, 1980b

*Anisandrus improbus* (Sampson, 1913)

*Anisandrus klapperichi* (Schedl, 1955b), comb. nov.

*Anisandrus lineatus* (Eggers, 1930)

*Xyleborus melancranis* Beeson, 1930

*Anisandrus longidens* (Eggers, 1930)

*Anisandrus maiche* (Kurentzov, 1941)

*Xyleborus maiche* Eggers, 1942

*Anisandrus mussooriensis* (Eggers, 1930)

*Anisandrus niger* (Sampson, 1912)

*Anisandrus paragogus* sp. nov.

*Anisandrus percristatus* (Eggers, 1939a), comb. nov.

*Anisandrus sinivali* sp. nov.

*Anisandrus ursulus* (Eggers, 1923)

*Anisandrus venustus* sp. nov.

*Anisandrus xuannu* sp. nov.

***Arixyleborus* Hopkins, 1915a**

*Xyleboricus* Eggers, 1923

*Arixyleborus crassior* sp. nov.

*Arixyleborus grandis* (Schedl, 1942c)

*Arixyleborus granifer* (Eichhoff, 1878a)

*Xyleborus granifer borneensis* Schedl, 1965

*Arixyleborus granulifer* (Eggers, 1923)

*Arixyleborus hirsutulus* Schedl, 1969a

*Arixyleborus leprosulus* Schedl, 1953b

*Arixyleborus aralidii* Nunberg, 1961

*Arixyleborus malayensis* (Schedl, 1954)

*Arixyleborus mediosectus* (Eggers, 1923)

*Arixyleborus angulatus* Schedl, 1942a

*Arixyleborus minor* (Eggers, 1940)

*Arixyleborus trux* Schedl, 1975c

*Arixyleborus moestus* (Eggers, 1930)

*Arixyleborus nudulus* Smith, Rabaglia & Cognato, 2018 (in Smith et al. 2018c)

*Arixyleborus phiaoacensis* sp. nov.

*Arixyleborus puberulus* (Blandford, 1896b)

*Xyleborus hirtipennis* Eggers, 1940

*Arixyleborus resecans* (Eggers, 1930), comb. nov.

*Arixyleborus rugosipes* Hopkins, 1915a

*Webbia medius* Eggers, 1927b

*Webbia camphorae* Eggers, 1936a

*Arixyleborus scabripennis* (Blandford, 1896b)

*Arixyleborus setosus* sp. nov.

*Arixyleborus silvanus* sp. nov.

*Arixyleborus sittichayai* sp. nov.

*Arixyleborus suturalis* (Eggers, 1936b)

*Arixyleborus titanus* sp. nov.

*Arixyleborus tuberculatus* (Eggers, 1940)

*Arixyleborus yakushimanus* (Murayama, 1958)

***Beaverium* Hulcr & Cognato, 2009**

*Beaverium lantanae* (Eggers, 1930)

*Beaverium latus* (Eggers, 1923)

*Beaverium magnus* (Niisima, 1910)

Xyleborus rufobrunneus var. dihingensis Eggers, 1930

*Xyleborus chujoi* Schedl, 1951a

***Cnestus* Sampson, 1911**

*Tosaxyleborus* Murayama, 1950

*Cnestus ater* (Eggers, 1923)

*Xyleborus retusiformis* Schedl, 1936d

*Cnestus aterrimus* (Eggers, 1927a)

*Xyleborus glabripennis* Schedl, 1942a

*Tosaxyleborus pallidipennis* Murayama, 1950

*Cnestus nitens* Browne, 1955

*Cnestus murayamai* Schedl, 1962a

*Cnestus murayamai* Browne, 1963

*Cnestus pseudosuturalis* Schedl, 1964c

*Cnestus maculatus* Browne, 1983b

*Cnestus bicornioides* (Schedl, 1952a)

*Cnestus gravidus* (Blandford, 1898)

*Cnestus improcerus* (Sampson, 1921)

*Cnestus mutilatus* (Blandford, 1894b)

*Xyleborus sampsoni* Eggers, 1930

*Xyleborus banjoewangi* Schedl, 1939b

*Xyleborus taitonus* Eggers, 1939b

*Cnestus nitidipennis* (Schedl, 1951a)

*Cnestus protensus* (Eggers, 1930)

*Cnestus rostratus* Schedl, 1977, syn. nov.

*Cnestus quadrispinosus* Sittichaya & Beaver, 2018

*Cnestus suturalis* (Eggers, 1930)

*Cnestus testudo* (Eggers, 1939b)

***Coptodryas* Hopkins, 1915a**

*Coptodryas amydra* sp. nov.

*Coptodryas bella* (Sampson, 1921)

*Coptodryas carinata* sp. nov.

*Coptodryas concinna* (Beeson, 1930)

*Xyleborus flexicostatus* Schedl, 1942c

*Coptodryas confusa* Hopkins, 1915a

*Xyleborus cryphaloides* Schedl, 1942a

*Coptodryas elegans* (Sampson, 1923)

*Coptodryas inornata* sp. nov.

*Coptodryas mus* (Eggers, 1930)

*Coptodryas nudipennis* (Schedl, 1951a)

*Coptodryas quadricostata* (Schedl, 1942c)

***Cryptoxyleborus* Wood & Bright, 1992**

*Cryptoxyleborus* Schedl, 1937a

*Cryptoxyleborus barbieri* Schedl, 1953a

*Cryptoxyleborus confusus* Browne, 1950

*Cryptoxyleborus eggersi* Schedl, 1936c

*Cryptoxyleborus dryobalanopsis* Schedl, 1942a

*Xyleborus eggersianus* Schedl, 1960b

*Cryptoxyleborus percuneolus* (Schedl, 1951a)

*Cryptoxyleborus quadriporus* Beaver, 1990

*Cryptoxyleborus stenographus* (Schedl, 1971b)

*Cryptoxyleborus subnaevus* Schedl, 1937a

*Cryptoxyleborus turbineus* (Sampson, 1923)

***Cyclorhipidion* Hagedorn, 1912b**

*Terminalinus* Hopkins, 1915a

*Notoxyleborus* Schedl, 1934b

*Kelantanius* Nunberg, 1961

*Cyclorhipidion amasoides* sp. nov.

*Cyclorhipidion amputatum* sp. nov.

*Cyclorhipidion armiger* (Schedl, 1953c), comb. nov.

*Cyclorhipidion bodoanum* (Reitter, 1913)

*Xyleborus punctulatus* Kurentzov, 1948

*Xyleborus californicus* Wood, 1975b

*Xyleborus misatoensis* Nobuchi, 1981a, syn. nov.

*Cyclorhipidion circumcisum* (Sampson, 1921)

*Xyleborus obtusus* Eggers, 1923

*Xyleborus subobtusus* Schedl, 1942a

*Cyclorhipidion denticauda* sp. nov.

*Cyclorhipidion distinguendum* (Eggers, 1930)

*Xyleborus fukiensis* Eggers, 1941b, syn. nov.

*Xyleborus ganshoensis* Murayama, 1952, syn. nov.

*Cyclorhipidion fouqueti* (Schedl, 1937b)

*Cyclorhipidion inarmatum* (Eggers, 1923)

*Xyleborus vagans* Schedl, 1977, syn. nov.

*Cyclorhipidion japonicum* (Nobuchi, 1981a)

*Cyclorhipidion miyazakiense* (Murayama, 1936)

*Xyleborus armipennis* Schedl, 1953c

*Xyleborus wakayamensis* Nobuchi, 1981a

*Cyclorhipidion muticum* sp. nov.

*Cyclorhipidion neocavipenne* (Schedl, 1977)

*Cyclorhipidion obesulum* sp. nov.

*Cyclorhipidion ohnoi* (Browne, 1980a)

*Cyclorhipidion pelliculosum* (Eichhoff, 1878a)

*Xyleborus seiryorensis* Murayama, 1930

*Xyleborus quercus* Kurentzov, 1948

*Xyleborus starki* Nunberg, 1956

*Cyclorhipidion perpilosellum* (Schedl, 1935a)

*Xyleborus punctatopilosus* Schedl, 1936b

*Cyclorhipidion petrosum* sp. nov.

*Cyclorhipidion pilipenne* (Eggers, 1940)

*Cyclorhipidion pruinosulum* Browne, 1979

*Cyclorhipidion pruinosum* (Blandford, 1896b)

*Xyleborus arcticollis* Blandford, 1896b

*Xyleborus decipiens* Eggers, 1923

*Cyclorhipidion sisyrnophorum* (Hagedorn, 1910a)

*Cyclorhipidion tenuigraphum* (Schedl, 1953) stat. res.

*Cyclorhipidion trucaudinum* sp. nov.

*Cyclorhipidion umbratum* (Eggers, 1941b)

*Cyclorhipidion vigilans* (Schedl, 1939b)

*Cyclorhipidion xeniolum* sp. nov.

*Cyclorhipidion xyloteroides* (Eggers, 1939b)

***Debus* Hulcr & Cognato, 2010a**

*Debus adusticollis* (Motschulsky, 1863)

*Xyleborus vestitus* Schedl, 1931

*Debus amphicranoides* (Hagedorn, 1908)

*Xyleborus amphicranoides latecavatus* Eggers, 1927b

*Xyleborus amphicranoides parvior* Browne, 1981b

*Debus birmanus* (Eggers, 1930)

*Debus detritus* (Eggers, 1927a)

*Xyleborus maniensis* Browne, 1981a

*Debus emarginatus* (Eichhoff, 1878a)

*Xyleborus exesus* Blandford, 1894b

*Ips cinchonae* Veen, 1897

*Xyleborus cordatus* Hagedorn, 1910a

*Xyleborus palmeri* Hopkins, 1915a

*Xyleborus terminaliae* Hopkins, 1915a

*Xyleborus emarginatus semicircularis* Schedl, 1973

*Debus pumilus* (Eggers, 1923)

*Xyleborus cylindricus* Eggers, 1927b

*Xyleborus neocylindricus* Schedl, 1942a

*Ips kelantanensis* Browne, 1955

*Xyleborus ipidia* Schedl, 1972a

*Xyleborus planodeclivis* Browne, 1974

*Debus quadrispinus* (Motschulsky, 1863), comb. nov.

*Xyleborus fallax* Eichhoff, 1878a, syn. nov.

*Xyleborus amphicranulus* Eggers, 1923

*Xyleborus fastigatus* Schedl, 1935a

*Debus shoreae* (Stebbing, 1907)

*Tomicus assamensis* Stebbing, 1909

***Diuncus* Hulcr & Cognato, 2009**

*Diuncus ciliatoformis* (Schedl, 1953d) stat. res.

*Diuncus corpulentus* (Eggers, 1930)

*Diuncus dossuarius* (Eggers, 1923)

*Diuncus haberkorni* (Eggers, 1920)

*Xyleborus approximatus* Schedl, 1951a

*Xyleborus taichuensis* Schedl, 1952b

*Xyleborus potens* Schedl, 1964a

*Diuncus javanus* (Eggers, 1923)

*Xyleborus perdix* Schedl, 1939a

*Diuncus justus* (Schedl, 1931)

*Xyleborus marginicollis* Schedl, 1936c

*Xyleborus ciliatus* Eggers, 1940

*Xyleborus apiculatus* Schedl, 1942a

*Diuncus mucronatulus* (Eggers, 1930)

*Diuncus mucronatus* (Eggers, 1923)

*Diuncus quadrispinulosus* (Eggers, 1923)

*Xyleborus parvispinosus palembangensis* Schedl, 1939b

*Xyleborus parvispinosus* Schedl, 1951a

***Dryoxylon* Bright & Rabaglia, 1999**

*Dryoxylon onoharaense* (Murayama, 1934)

***Eccoptopterus* Motschulsky, 1863**

*Platydactylus* Eichhoff, 1886

*Eurydactylus* Hagedorn, 1909

*Eccoptopterus limbus* Sampson, 1911

*Xyleborus auratus* Eggers, 1923

*Xyleborus squamulosus duplicatus* Eggers, 1923

*Xyleborus squamulosus* Eggers, 1923

*Eccoptopterus spinosus* (Olivier, 1800)

*Eccoptopterus sexspinosus* Motschulsky, 1863

*Xyleborus abnormis* Eichhoff, 1869

*Platydactylus gracilipes* Eichhoff, 1886

*Xyleborus sexspinosus multispinosus* Hagedorn, 1908

*Xyleborus collaris* Eggers, 1923

*Eccoptopterus sagittarius* Schedl, 1939b

*Eccoptopterus sexspinosus pluridentatus* Schedl, 1942c

*Xyleborus eccoptopterus* Schedl, 1951b

***Euwallacea* Hopkins, 1915a**

*Wallacellus* Hulcr & Cognato, 2010a

*Euwallacea andamanensis* (Blandford, 1896b)

*Xyleborus noxius* Sampson, 1913

*Xyleborus siobanus* Eggers, 1923

*Xyleborus burmanicus* Beeson, 1930

*Xyleborus granulipennis* Eggers, 1930

*Xyleborus intextus* Beeson, 1930

*Xyleborus senchalensis* Beeson, 1930

*Xyleborus talumalai* Browne, 1966

*Euwallacea aplanatus* (Wichmann, 1914)

*Euwallacea destruens* (Blandford, 1896b)

*Xyleborus barbatus* Hagedorn, 1910a

*Xyleborus barbatulus* Schedl, 1934b

*Xyleborus pseudobarbatus* Schedl, 1942a

*Xyleborus nandarivatus* Schedl, 1950a

*Xyleborus procerrimus* Schedl, 1969a

*Euwallacea fornicatior* (Eggers, 1923)

*Xyleborus schultzei* Schedl, 1951a

*Euwallacea fornicatus* (Eichhoff, 1868b)

*Xyleborus whitfordiodendrus* Schedl, 1942a

*Xyleborus tapatapaoensis* Schedl, 1951b

*Euwallacea funereus* (Lea, 1910)

*Xyleborus nepos* Eggers, 1923

*Xyleborus nepos robustus* Schedl, 1933

*Xyleborus signatus* Schedl, 1949

*Euwallacea geminus* sp. nov.

*Euwallacea gravelyi* (Wichmann, 1914) stat. res.

*Xyleborus ovalicollis* Eggers, 1930

*Xyleborus barbatomorphus* Schedl, 1951a, syn. nov.

*Euwallacea interjectus* (Blandford, 1894c)

*Xyleborus pseudovalidus* Eggers, 1925

*Euwallacea kuroshio* Gomez & Hulcr, 2018 (in Gomez et al. 2018b)

*Euwallacea luctuosus* (Eggers, 1939a)

*Euwallacea malloti* (Eggers, 1930)

*Euwallacea minutus* (Blandford, 1894b), comb. nov.

*Xyleborus breviusculus* Schedl, 1942a

*Xyleborus pernitidus* Schedl, 1954

*Euwallacea neptis* sp. nov.

*Euwallacea perbrevis* (Schedl, 1951a)

*Xyleborus molestulus* Wood, 1975, syn. nov.

*Euwallacea piceus* (Motschulsky, 1863)

*Xyleborus indicus* Eichhoff, 1878a

*Xyleborus imitans* Eggers, 1927a

*Xyleborus indicus subcoriaceus* Eggers, 1927b

*Xyleborus samoensis* Beeson, 1929

*Euwallacea semiermis* (Schedl, 1934c)

*Euwallacea semirudis* (Blandford, 1896b) stat. res.

*Xyleborus dubius* Eggers, 1923

*Xyleborus sereinuus* Eggers, 1923

*Xyleborus hybridus* Eggers, 1927b

*Xyleborus interruptus* Eggers, 1940

*Xyleborus neohybridus* Schedl, 1942a, syn. nov.

*Xyleborus longehirtus* Nunberg, 1956

*Euwallacea sibsagaricus* (Eggers, 1930)

*Xyleborus dalbergiae* Eggers, 1930

*Xyleborus tonkinensis* Schedl, 1934a, syn. nov.

*Euwallacea similis* (Ferrari, 1867)

*Bostrichus ferrugineus* Bohemann, 1858

*Xyleborus parvulus* Eichhoff, 1868b

*Xyleborus dilatatus* Eichhoff, 1878b

*Xyleborus submarginatus* Blandford, 1896b

*Xyleborus bucco* Schaufuss, 1897

*Xyleborus capito* Schaufuss, 1897

*Xyleborus novaguineanus* Schedl, 1936b

*Xyleborus dilatatulus* Schedl, 1953a

*Euwallacea subalpinus* sp. nov.

*Euwallacea testudinatus* sp. nov.

*Euwallacea validus* (Eichhoff, 1876a)

*Euwallacea velatus* (Sampson, 1913)

*Xyleborus assamensis* Eggers, 1930

*Xyleborus rudis* Eggers, 1930, syn. nov.

*Xyleborus asperipennis* Eggers, 1934b

***Fortiborus* Hulcr & Cognato, 2010a**

*Fortiborus macropterus* (Schedl, 1935b)

*Fortiborus major* (Stebbing, 1909)

*Xyleborus siclus* Schedl, 1936d

*Fortiborus pseudopilifer* (Schedl, 1936a)

***Fraudatrix* Cognato, Smith & Beaver, 2020**

*Fraudatrix cuneiformis* (Schedl, 1958b)

*Fraudatrix melas* (Eggers, 1927b)

*Fraudatrix simplex* (Browne, 1949)

***Hadrodemius* Wood, 1980**

*Hadrodemius comans* (Sampson, 1919)

*Xyleborus amorphus* Eggers, 1926

*Xyleborus metacomans* Eggers, 1930

*Hadrodemius globus* (Blandford, 1896b)

*Xyleborus ursus* Eggers, 1923

*Xyleborus ursus fuscus* Eggers, 1923

*Xyleborus tomentosus* Eggers, 1939a

*Hadrodemius pseudocomans* (Eggers, 1930)

*Xyleborus artecomans* Schedl, 1953c

***Heteroborips* Reitter, 1913**

*Heteroborips fastigatus* sp. nov.

*Heteroborips indicus* sp. nov.

*Heteroborips seriatus* (Blandford, 1894b)

*Xyleborus orientalis* Eggers, 1933b

*Xyleborus todo* Kôno, 1938

*Xyleborus orientalis aceris* Kurentzov, 1941

*Xyleborus orientalis kalopanacis* Kurentzov, 1941

*Xyleborus perorientalis* Schedl, 1957

*Heteroborips tristis* (Eggers, 1930), comb. nov.

***Immanus* Hulcr & Cognato, 2013**

*Immanus desectus* (Eggers, 1923)

*Xyleborus desectus arduus* Schedl, 1942a

*Immanus sarawakensis* (Eggers, 1923)

***Leptoxyleborus* Wood, 1980**

*Leptoxyleborus machili* (Niisima, 1910), comb. nov.

*Xyleborus depressus* Eggers, 1923

*Xyleborus kojimai* Murayama, 1936

*Xyleborus sejugatus* Schedl, 1942a

*Leptoxyleborus sordicauda* (Motschulsky, 1863)

*Phloeotrogus attenuatus* Motschulsky, 1863

*Xyleborus concisus* Blandford, 1894b

*Xyleborus marginatus* Eggers, 1927b

*Xyleborus sordicaudulus* Eggers, 1927b

*Xyleborus incurvus* Eggers, 1930

*Xyleborus sordicaudulus peguensis* Eggers, 1930

***Microperus* Wood, 1980**

*Microperus alpha* (Beeson, 1929)

*Microperus chrysophylli* (Eggers, 1930)

*Microperus corporaali* (Eggers, 1923)

*Microperus cruralis* (Schedl, 1975b), comb. nov.

*Microperus diversicolor* (Eggers, 1923)

*Xyleborus myristicae* Schedl, 1939b

*Xyleborus brevipilosus* Eggers, 1940

*Xyleborus theae* Eggers, 1940

*Xyleborus cylindripennis* Schedl, 1954

*Xyleborus atavus* Schedl, 1979b

*Microperus fulvulus* (Schedl, 1942c) stat. res.

*Xyleborus fulvus* Schedl, 1939b

*Microperus kadoyamaensis* (Murayama, 1934)

*Xyleborus denseseriatus* Eggers, 1941b, syn. nov.

*Xyleborus nameranus* Murayama, 1954

*Xyleborus pubipennis* Schedl, 1974, syn. nov.

*Xyleborus huangi* Browne, 1983b

*Microperus kirishimanus* (Murayama, 1955)

*Microperus latesalebrinus* sp. nov.

*Microperus minax* sp. nov.

*Microperus nudibrevis* (Schedl, 1942a)

*Microperus nugax* (Schedl, 1939a)

*Xyleborus pertuberculatus* Eggers, 1940

*Microperus perparvus* (Sampson, 1922b)

*Xyleborus tsukubanus* Murayama, 1954

*Microperus pometianus* (Schedl, 1939a)

*Microperus quercicola* (Eggers, 1926)

*Xyleborus izuensis* Murayama, 1952

*Microperus recidens* (Sampson, 1923)

*Xyleborus minusculus* Eggers, 1923

*Xyleborus minutissimus* Eggers, 1930

*Xyleborus crassitarsus* Schedl, 1936d

*Xyleborus artegraphus* Schedl, 1942c

*Xyleborus extensus* Schedl, 1955a

*Xyleborus tuberculosus* Browne, 1981b

*Microperus sagmatus* sp. nov.

*Microperus undulatus* (Sampson, 1919)

*Xyleborus leprosulus* Schedl, 1936d

***Planiculus* Hulcr & Cognato, 2010a**

*Planiculus bicolor* (Blandford, 1894b)

*Xyleborus laevis* Eggers, 1923

*Xyleborus bicolor unimodus* Beeson, 1929

*Xyleborus rodgeri* Beeson, 1930

*Xyleborus rodgeri privatus* Beeson, 1930

*Xyleborus rameus* Schedl, 1940a

*Xyleborus artelaevis* Schedl, 1942a

*Xyleborus ashuensis* Murayama, 1954

*Xyleborus tumidus* Schedl, 1975c

*Xyleborus filiformis* Schedl, 1975c

*Xyleborus glabratulus* Browne, 1983a

*Planiculus limatus* (Schedl, 1942b)

*Xyleborus subemarginatus* Eggers, 1940

*Xyleborus subparallelus* Eggers, 1940

*Planiculus shiva* (Maiti & Saha, 1986), comb. nov.

***Pseudowebbia* Browne, 1961a**

*Pseudowebbia trepanicauda* (Eggers, 1923)

***Schedlia* Browne, 1950b**

*Schedlia allecta* (Schedl, 1942c)

*Schedlia sumatrana* (Hagedorn, 1908)

***Stictodex* Hulcr & Cognato, 2013**

*Stictodex dimidiatus* (Eggers, 1927a)

*Xyleborus dorsosulcatus* Beeson, 1930, syn. nov.

*Xyleborus tunggali* Schedl, 1936d

*Xyleborus decumans* Schedl, 1953b

*Xyleborus cruciatus* Schedl, 1973

***Streptocranus* Schedl, 1939b**

*Streptocranus bicolor* (Browne, 1949)

*Streptocranus bicuspis* (Eggers, 1940)

*Streptocranus recurvus* Browne, 1949

*Streptocranus fragilis* Browne, 1949

*Streptocranus mirabilis* Schedl, 1939b

*Streptocranus petilus* sp. nov.

***Tricosa* Cognato, Smith & Beaver, 2020**

*Tricosa cattienensis* Cognato, Smith & Beaver, 2020 (in Cognato et al. 2020a)

*Tricosa indochinensis* Cognato, Smith & Beaver, 2020 (in Cognato et al. 2020a)

*Tricosa jacula* Cognato, Smith & Beaver, 2020 (in Cognato et al. 2020a)

*Tricosa metacuneolus* (Eggers, 1940)

*Xyleborus kaimochii* Nobuchi, 1981a

***Truncaudum* Hulcr & Cognato, 2010a**

*Truncaudum agnatum* (Eggers, 1923)

*Xyleborus polyodon* Eggers, 1923

*Xyleborus gratiosus* Schedl 1942a

*Xyleborus nutans* Schedl, 1942a

*Xyleborus delicatus* Schedl, 1955a

*Xyleborus subagnatus* Wood, 1992

*Truncaudum bullatum* sp. nov.

***Webbia* Hopkins, 1915b**

*Xelyborus* Schedl, 1939a

*Prowebbia* Browne, 1962

*Webbia biformis* Browne, 1958

*Webbia cornuta* Schedl, 1942a

*Webbia dasyura* Browne, 1981a

*Webbia dipterocarpi* Hopkins, 1915b

*Webbia diversicauda* Browne, 1972

*Webbia duodecimspinata* Schedl, 1942a

*Webbia pabo* Sampson, 1922

*Webbia quatuordecimspinata* Sampson, 1921

*Webbia trigintispinata* Sampson, 1922

*Webbia vigintisexspinata* Sampson, 1922

*Webbia mucronatus* Eggers, 1927, syn. nov.

*Webbia turbinata* Maiti & Saha, 1986

***Xyleborinus* Reitter, 1913**

*Xyleborinus andrewesi* (Blandford, 1896b)

*Xyleborus persphenos* Schedl, 1970a

*Xyleborus insolitus* Bright, 1972

*Cryptoxyleborus gracilior* Browne, 1984a

*Xyleborinus artestriatus* (Eichhoff, 1878b)

*Xyleborus laticollis* Blandford, 1896b

*Xyelborus angustior* Eggers, 1925, syn. nov.

*Xyleborus rugipennis* Schedl, 1953b

*Xyleborus undatus* Schedl, 1974, syn. nov.

*Xyleborus beaveri* Browne, 1978

*Xyleborinus attenuatus* (Blandford, 1894b)

*Xyleborus alni* Niisima, 1909

*Xyleborus canus* Niisima, 1909

*Xyleborinus cuneatus* sp. nov.

*Xyleborinus disgregus* sp. nov.

*Xyleborinus echinopterus* sp. nov.

*Xyleborinus ephialtodes* sp. nov.

*Xyleborinus exiguus* (Walker, 1859)

*Xyleborus muriceus* Eichhoff, 1878a

*Xyleborus diversus* Schedl, 1954b, syn. nov.

*Xyleborus perexiguus* Schedl, 1971b

*Xyleborus ankius* Schedl, 1975c

*Xyleborinus huifenyinae* sp. nov.

*Xyleborinus jianghuasuni* sp. nov.

*Xyleborinus octiesdentatus* (Murayama, 1931)

*Xyleborinus perpusillus* (Eggers, 1927a)

*Xyleborus perminutissimus* Schedl, 1934b

*Xyleborus angustatulus* Schedl, 1942c

*Xyleborinus saxesenii* (Ratzeburg, 1837)

*Xyleborus dohrni* Wollaston, 1854

*Xyleborus decolor* Boieldieu, 1859

*Xyleborus aesculi* Ferrari, 1867

*Xyleborus subdepressus* Rey, 1883

*Xyleborus frigidus* Blackburn, 1885

*Xyleborus arbuti* Hopkins, 1915a

*Xyleborus floridensis* Hopkins, 1915a

*Xyleborus pecanis* Hopkins, 1915a

*Xyleborus quercus* Hopkins, 1915a

*Xyleborus sobrinus* Eichhoff, 1876a

*Xyleborinus librocedri* Swaine, 1934

*Xyleborinus tsugae* Swaine, 1934

*Xyleborus pseudogracilis* Schedl, 1937c

*Xyleborus retrusus* Schedl, 1940b

*Xyleborus peregrinus* Eggers, 1944

*Xyleborus pseudoangustatus* Schedl, 1948

*Xyleborus paraguayensis* Schedl, 1949

*Xyleborus opimulus* Schedl, 1976

*Xyleborinus schaufussi* (Blandford, 1894b)

*Xyleborus kraunhiae* Niisima, 1910

*Xyleborinus sculptilis* (Schedl, 1964b)

*Xyleborinus speciosus* (Schedl, 1975b)

*Xyleborinus spinipennis* (Eggers, 1930)

*Xyleborinus subgranulatus* (Eggers, 1930)

*Xyleborinus subspinosus* (Eggers, 1930) stat. res.

*Xyleborinus thaiphami* sp. nov.

*Xyleborinus tritus* sp. nov.

***Xyleborus* Eichhoff, 1864**

*Anaeretus* Dugès, 1888

*Progenius* Blandford, 1896a

*Mesoscolytus* Broun, 1904

*Boroxylon* Hopkins, 1915a

*Xyleborus affinis* Eichhoff, 1868b

*Xyleborus affinis fuscobrunneus* Eichhoff, 1878b

*Xyleborus affinis mascarensis* Eichhoff, 1878b

*Xyleborus affinis parvus* Eichhoff, 1878b

*Xyleborus sacchari* Hopkins, 1915a

*Xyleborus societatis* Beeson, 1935a

*Xyleborus subaffinis* Eggers, 1933a

*Xyleborus proximus* Eggers, 1943

*Xyleborus bidentatus* (Motschulsky, 1863)

*Xyleborus subcostatus* Eichhoff, 1869a

*Xyleborus riehlii* Eichhoff, 1878b

*Progenius fleutiauxi* Blandford, 1896a

*Xyleborus laeviusculus* Blandford, 1896a

*Boroxylon stephegynis* Hopkins, 1915a

*Boroxylon webbi* Hopkins, 1915a

*Xyleborus subcostatus dearmatus* Eggers, 1923

*Xyleborus brevidentatus* Eggers, 1930

*Xyleborus quadridens* Eggers, 1930

*Xyleborus cognatus* Blandford, 1896a

*Xyleborus ferrugineus* (Fabricius, 1801)

*Tomicus trypanaeoides* Wollaston, 1867

*Xyleborus confusus* Eichhoff, 1868a

*Xyleborus fuscatus* Eichhoff, 1868a

*Xyleborus retusicollis* Zimmermann, 1868

*Xyleborus amplicollis* Eichhoff, 1869

*Xyleborus insularis* Sharp, 1885

*Xyleborus tanganus* Hagedorn, 1910a

*Xyleborus nyssae* Hopkins, 1915a

*Xyleborus soltaui* Hopkins, 1915a

*Xyleborus hopkinsi* Beeson, 1929

*Xyleborus argentinensis* Schedl, 1931

*Xyleborus rufopiceus* Eggers, 1932

*Xyleborus schedli* Eggers, 1934a

*Xyleborus nesianus* Beeson, 1940

*Xyleborus notatus* Eggers, 1941a

*Xyleborus subitus* Schedl, 1949

*Xyleborus festivus* Eichhoff, 1876a

*Xyleborus pinicola* Eggers, 1930

*Xyleborus detectus* Schedl, 1975a

*Xyleborus pinivorus* Browne, 1980a

*Xyleborus glabratus* Eichhoff, 1877

*Xyleborus kumamotoensis* Murayama, 1934

*Xyleborus insidiosus* Cognato & Smith, 2019

*Xyleborus muticus* Blandford, 1894b

*Xyleborus lignographus* Schedl, 1953c, syn. nov.

*Xyleborus conditus* Schedl, 1971b, syn. nov.

*Xyleborus mysticulus* Cognato & Smith, 2019

*Xyleborus opacus* sp. nov.

*Xyleborus perforans* (Wollaston, 1857)

*Bostrichus testaceus* Walker, 1859

*Xyleborus duponti* Montrouzier, 1861

*Anodius denticulus* Motschulsky, 1863

*Anodius tuberculatus* Motschulsky, 1863

*Xyleborus kraatzii* Eichhoff, 1868b

*Xyleborus kraatzii philippinensis* Eichhoff, 1878b

*Xyleborus immaturus* Blackburn, 1885

*Xylopertha hirsuta* Lea, 1894

*Xyleborus whitteni* Beeson, 1935b

*Xyleborus apertus* Schedl, 1939a

*Xyleborus criticus* Schedl, 1950b

*Xyleborus cylindrus* Schedl, 1951a

*Xyleborus shionomisakiensis* Murayama, 1951

*Xyleborus minimus* Schedl, 1955a

*Xyleborus pfeilii* (Ratzeburg, 1837)

*Bostrichus alni* Mulsant & Rey, 1856

*Xyleborus vicarius* Eichhoff, 1876a

*Xyleborus adumbratus* Blandford, 1894b

*Xyleborus septentrionalis* Niisima, 1909

*Xyleborus singhi* Park & Smith, 2020

*Xyleborus sunisae* sp. nov.

*Xyleborus volvulus* (Fabricius, 1775)

*Xyleborus torquatus* Eichhoff, 1868b

*Xyleborus alternans* Eichhoff, 1869

*Xyleborus badius* Eichhoff, 1869

*Xyleborus interstitalis* Eichhoff, 1878b

*Xyleborus guanajuatensis* Dugès, 1887

*Xyleborus grenadensis* Hopkins, 1915a

*Xyleborus hubbardi* Hopkins, 1915a

*Xyleborus rileyi* Hopkins, 1915a

*Xyleborus schwarzi* Hopkins, 1915a

*Xyleborus continentalis* Eggers, 1920

*Xyleborus silvestris* Beeson, 1929

*Xyleborus vagabundus* Schedl, 1949

*Xyleborus granularis* Schedl, 1950b

*Xyleborus yunnanensis* sp. nov.

***Xylosandrus* Reitter, 1913**

*Apoxyleborus* Wood, 1980

*Xylosandrus adherescens* Schedl, 1971b

*Xylosandrus amputatus* (Blandford, 1894c)

*Xyleborus melli* Schedl, 1938

*Xylosandrus beesoni* Saha, Maiti & Chakraborti, 1992

*Xylosandrus bellinsulanus* sp. nov.

*Xylosandrus borealis* Nobuchi, 1981b

*Xylosandrus brevis* (Eichhoff, 1877)

*Xyleborus cucullatus* Blandford, 1894b

*Xyleborus montanus* Niisima, 1910

*Xylosandrus compactus* (Eichhoff, 1876a)

*Xyleborus morstatti* Hagedorn, 1912a

*Xylosandrus crassiusculus* (Motschulsky, 1866)

*Xyleborus semiopacus* Eichhoff, 1878b

*Xyleborus semigranosus* Blandford, 1896b

*Dryocoetes bengalensis* Stebbing, 1908

*Xyleborus mascarenus* Hagedorn, 1908

*Xyleborus ebriosus* Niisima, 1909

*Xyleborus okoumeensis* Schedl, 1935b

*Xyleborus declivigranulatus* Schedl, 1936d

*Xylosandrus dentipennis* Park & Smith, 2020

*Xylosandrus derupteterminatus* (Schedl, 1951a)

*Xylosandrus discolor* (Blandford, 1898)

*Xyleborus posticestriatus* Eggers, 1939b

*Xylosandrus diversepilosus* (Eggers, 1941b)

*Xylosandrus eupatorii* (Eggers, 1940)

*Xylosandrus formosae* (Wood), comb. nov.

*Xyleborus formosanus* Browne, 1981a

*Xylosandrus germanus* (Blandford, 1894b)

*Xyleborus orbatus* Blandford, 1894b

*Xylosandrus jaintianus* (Schedl, 1967)

*Xylosandrus mancus* (Blandford, 1898)

*Xyleborus abruptus* Sampson, 1914

*Xyleborus mancus formosanus* Eggers, 1930

*Xylosandrus mesuae* (Eggers, 1930)

*Xylosandrus metagermanus* (Schedl, 1951a)

*Xylosandrus morigerus* (Blandford, 1894a)

*Xyleborus coffeae* Wurth, 1908

*Xyleborus difficilis* Eggers, 1923

*Xyleborus luzonicus* Eggers, 1923

*Xyleborus abruptoides* Schedl, 1955a

*Xylosandrus spinifer* sp. nov.

*Xylosandrus subsimiliformis* (Eggers, 1939a)

*Xylosandrus subsimilis* (Eggers, 1930)

## Taxonomic treatment

### Key to Xyleborini genera of Southeast Asia (females only)

**Table:** 

1	Scutellum not easily visible in dorsal view, apparently absent (Fig. 73A), or conical (Fig. 86A), or narrow, minute and convex (Fig. 67E), or visible only on anterior slope of elytral bases (Fig. 62E)	**2**
–	Scutellum distinctly visible, linguiform, flush with the elytra, or medially depressed below elytra	**13**
2	Scutellum conical and surrounded by setae (Fig. 86A)	*** Xyleborinus ***
–	Scutellum apparently absent (Fig. 73A), or narrow, minute and convex (Fig. 67E), or visible only on anterior slope of elytral bases (Fig. 62E)	**3**
3	Pronotum with a dense basal mycangial tuft (Fig. 62A); antennal scape long and slender, gradually thickening to apex	*** Hadrodemius ***
–	Pronotum without a mycangial tuft (Fig. 79G); antennal scape short and thick, or of even thickness	**4**
4	Mesonotal mycangial tuft in two or four pit mycangia located on the elytra either near the scutellum or along the base (Fig. 39A, C, E), or mycangial tufts absent (Fig. 38G); body elongate with elytral apex attenuate or acuminate	*** Cryptoxyleborus ***
–	Mesonotal mycangial tuft on elytral bases (Fig. 79G); body stouter with rounded or truncate elytral apex	**5**
5	Anterior margin of pronotum quadrate or subquadrate, and emarginated; posterior face of protibiae inflated, with or without granules	**6**
–	Anterior margin of pronotum rounded, never emarginated; posterior face of protibiae flat and unarmed by granules	**7**
6	Pronotum 1.1–2.0× longer than wide; pronotal disc smooth, finely punctate; antennal funicle 2- or 3-segmented; posterior face of protibiae inflated and unarmed by granules	*** Webbia ***
–	Pronotum wider than long; pronotal disc coarse, finely asperate; antennal funicle 4-segmented; posterior face of protibiae inflated and granulate	*** Schedlia ***
7	Declivity truncate, circular, completely surrounded by a circle of pointed teeth	*** Pseudowebbia ***
–	Declivity not as above if truncate, then not surrounded by a circle of pointed teeth	**8**
8	Antennal club obliquely truncate, type 2 with one or two sutures visible on posterior face (Fig. 2); pronotal disc punctate	***Microperus* , in part**
–	Antennal club flattened, types 3 or 4 with two or three sutures visible on posterior face (Fig. 3); pronotal disc finely asperate or punctate	**9**
9	Pronotal disc finely asperate (Fig. 35G)	***Coptodryas* , in part**
–	Pronotal disc punctate (Fig. 36E)	**10**
10	Antennal club circular	***Coptodryas amydra* sp. nov.**
–	Antennal club longer than wide	***Microperus* , in part**
11	Elytral bases straight (Fig. 67C)	***Microperus fulvulus***
–	Elytral bases bisinuate (Fig. 36E)	**12**
12	Protibiae distinctly triangular, denticles on apical 1/3 of outer margin	***Coptodryas inornata* sp. nov.**
–	Protibiae semi-circular with evenly rounded outer edge, denticles along most of length or obliquely triangular with denticles on apical half	***Microperus* , in part**
13	Elytral with oblong pit mycangia in distinctly impressed area immediately adjacent to the scutellum on each elytron (Fig. 63C)	*** Heteroborips ***
–	Elytra without pit mycangia (Fig. 45G)	**14**
14	Mycangial tuft present on basal margin of pronotum (Fig. 94H) (tuft faint in several species, e.g., Fig. 94B)	**15**
–	Pronotum without mycangial tufts (Fig. 7F)	**18**
15	Procoxae widely separated	***Xylosandrus* , in part**
–	Procoxae contiguous or narrowly separated	**16**
16	Metatibiae conspicuously enlarged and flattened; pronotal disc asperate	*** Eccoptopterus ***
–	Metatibiae similar to pro- and mesotibiae, never enlarged; pronotal disc punctate	**17**
17	Lateral margins of pronotum carinate (Fig. 33D)	***Cnestus* , in part**
–	Lateral margins of pronotum obliquely costate (Fig. 22D)	***Anisandrus* , in part**
18	Elytral apex divaricate and ornamented with a pair of distal projections; very elongate, 3.85–4.75× as long as wide	*** Streptocranus ***
–	Elytral apex entire without a pair of distal projections; stout to elongate, 2.1–3.4× as long as wide	**19**
19	Posterior face of protibiae inflated, granulate	**20**
–	Posterior face of protibiae flat, without granules	**23**
20	Declivital face with three striae (Fig. 8L); antennal club flattened, types 4 or 5 with zero or three sutures on posterior face (Fig. 3)	***Amasa* , in part**
–	Declivital face with five or six striae (Fig. 28C); antennal club obliquely truncate, type 1 or 2 with zero or one suture on posterior face (Fig. 2)	**21**
21	Elytra with distinctive deep strial furrows and interstrial ridges, ridges either granulate or carinate (Fig. 26E)	***Arixyleborus* , in part**
–	Elytra without strial furrows and interstrial ridges (Fig. 28C)	**22**
22	Declivital posterolateral margin rounded; lateral profile of declivity appearing obliquely truncate; declivity armed with numerous tubercles; declivital striae 1 variably undulating, never parallel to suture (Fig. 74)	*** Stictodex ***
–	Declivital posterolateral margin carinate forming a circumdeclivital ring; lateral profile of declivity appearing truncate; declivity unarmed; declivital striae 1 parallel to suture (Fig. 28C, D, J)	***Arixyleborus resecans***
23	Scutellum flush with elytra and medially impressed (Fig. 28G), or depressed below elytra (Fig. 30A)	***Arixyleborus* , in part**
–	Scutellum flush with elytra and flat (Fig. 25C)	**24**
24	Elytra with distinctive deep strial furrows and interstrial ridges, ridges either granulate or carinate	***Arixyleborus* , in part**
–	Elytra without strial furrows and interstrial ridges	**25**
25	Anterior margin of pronotum feebly emarginate (Fig. 52A); submentum not depressed below ventral surface of head	*** Dryoxylon ***
–	Anterior margin of pronotum entire (Fig. 12C); submentum depressed below ventral surface of head (except *Ancipitis*, some *Diuncus*)	**26**
26	Pronotal disc asperate (Fig. 12C), coarsely sculptured	**27**
–	Pronotal disc punctate (Fig. 47C), finely sculptured	**29**
27	Anterior margin of pronotum with separate asperities of almost equal size, not larger than those on anterior slope (Fig. 12C)	*** Ambrosiodmus ***
–	Anterior margin of pronotum with two or more distinctly larger asperities, which may be fused to form a recurved carina (Fig. 31E)	**28**
28	Protibiae with normal socketed denticles, their bases elevated; declivity distinctly flattened and posterolaterally widened, posterolateral margin costate to interstriae 5; declivital interstriae 2 without spines or tubercles (Fig. 31E)	*** Beaverium ***
–	Protibiae with denticles reduced or absent, only the raised bases present; declivity either convex with posterolateral margin costate to interstriae 7, or truncate, its margin forming a circular rim around the declivity; spines or tubercles present on declivital interstriae 2 (Fig. 64A)	*** Immanus ***
29	Elytral apex emarginate and/or explanate (Fig. 48A)	**30**
–	Elytral apex entire (Fig. 33C)	**31**
30	Elytra never explanate or excavated	***Planiculus* , in part**
–	Elytra explanate and weakly to strongly excavated (not explanate, strongly excavated and apex appearing subquadrate in *D. adusticollis*)	*** Debus ***
31	Lateral margin of pronotum carinate (Fig. 33D)	***Cnestus* , in part**
–	Lateral margin of pronotum obliquely costate (Fig. 7D)	**32**
32	Procoxae narrowly separated	**33**
–	Procoxae contiguous	**35**
33	Elytra truncate; antennal club flattened, types 4 or 5 (Fig. 3)	***Amasa* , in part**
–	Elytra rounded; antennal club obliquely truncate, types 1 or 2 (Fig. 2)	**34**
34	Declivity unarmed, lacking granules or tubercles (some granules on disc)	***Xylosandrus formosae***
–	Declivity bearing granules or tubercles	***Anisandrus* , in part**
35	Antennal club flattened, types 3, 4 or 5 (Fig. 3)	**36**
–	Antennal club obliquely truncate, types 1 or 2 (Fig. 2)	**46**
36	Elytral disc with at least interstrial punctures confused (Fig. 42G)	*** Cyclorhipidion ***
–	Elytral disc with interstrial punctures uniseriate or interstriae impunctate (Fig. 57E)	**37**
37	Submentum large, distinctly triangular and flat, flush with genae	*** Ancipitis ***
–	Submentum variable, slightly or deeply depressed below genae	**38**
38	Protibiae semi-circular with evenly rounded outer edge	***Euwallacea* , in part**
–	Protibiae obliquely or distinctly triangular	**39**
39	Anterior margin of pronotum conspicuously extended anteriad with prominent serrations (Fig. 60A)	**40**
–	Anterior margin of pronotum not conspicuously extended anteriad, without serrations (Fig. 14G)	**41**
40	Elytral apex rounded; eyes very large, deeply emarginate; elytral apex angulate; larger, 4.8–6.6 mm	*** Fortiborus ***
–	Elytral apex acuminate; eyes small, feebly emarginate, almost entire; smaller, 3.4–3.5 mm	***Xyleborus bidentatus***
41	Anterior margin of pronotum subquadrate or quadrate in dorsal view (Fig. 4)	**42**
–	Anterior margin of pronotum conical or rounded in dorsal view (Fig. 4)	**43**
42	Pronotum wider than long; stouter species, 2.3–2.7× as long as wide	***Ambrosiophilus osumiensis* , in part**
–	Pronotum at least 1.15× longer than wide; elongate species, 2.78–2.89× as long as wide	***Euwallacea semiermis***
43	Elytral apex attenuate, sides parallel in basal 30–60%; declivital slope very gradually rounded; scutellum small	*** Tricosa ***
–	Elytral apex narrowly or broadly rounded, sides parallel in basal 66–80%; declivital slope evenly or steeply rounded; scutellum large	**44**
44	Protibiae with six or more socketed denticles	***Ambrosiophilus* , in part**
–	Protibiae with five socketed denticles	**45**
45	Declivital interstriae unarmed by tubercles or granules	***Ambrosiophilus lannaensis* sp. nov.**
–	Declivital interstriae 2 and 3 each bearing three large tubercles	***Xyleborus singhi***
46	Antennal club 2-segmented, elytra attenuate	*** Fraudatrix ***
–	Antennal club 3- or 4-segmented, elytra variable but never attenuate	**47**
47	Antennal club type 1, segment 1 encircling anterior face, no sutures on posterior face (Fig. 2); antennal funicle long and slender; anterior margin of pronotum serrate (absent in *D. ciliatoformis*)	*** Diuncus ***
–	Antennal club type 2, with at least one suture on posterior face (Fig. 2); antennal funicle regularly thick or short and thick; anterior margin of pronotum without serrations	**48**
48	Protibiae semi-circular with evenly rounded outer edge	**49**
–	Protibiae obliquely or distinctly triangular without evenly rounded edge	**52**
49	Elytral disc with interstrial punctures confused	**50**
–	Elytral disc with interstrial punctures uniseriate	**51**
50	Declivity steeply rounded, posterolateral margin costate and tuberculate	***Xyleborus* , in part**
–	Declivity truncate and encircled by a tuberculate circumdeclivital carina	***Truncaudum bullatum* sp. nov.**
51	Pronotal summit prominent	***Euwallacea* , in part**
–	Pronotal summit low, indistinct	***Truncaudum agnatum***
52	Declivity extremely flat, laterally broadened and densely setose, setae star-shaped scales or bristle-like; declivital slope very gradual	*** Leptoxyleborus ***
–	Declivity variably convex or slightly broadened and slightly to moderately setose, setae hair-like; declivital slope steep or evenly rounded	**53**
53	Posterolateral margin of declivity acutely carinate; elytral apex laterally broadened	**54**
–	Posterolateral margin of declivity rounded or costate; elytral apex variably rounded	**55**
54	Declivital interstriae 2 armed by tubercles and granules; body unicolored	***Xyleborus* , in part**
–	Declivital interstriae 2 unarmed by tubercles, typically unarmed by granules; body typically bicolored	***Planiculus bicolor***
55	Declivital interstriae 1 laterally broadened from base to declivital midpoint and then narrowing towards apex	***Xyleborus* , in part**
–	Declivital interstriae 1 parallel to suture along its length	**56**
56	Declivity with tubercles on interstriae 1 and 3 equally sized or those of interstriae 3 the largest	***Xyleborus* , in part (*Xyleboruss. s.*)**
–	Declivity with tubercles on interstriae 1, 2 and 3 equally sized or those of interstriae 1 the largest	***Euwallacea* , in part**

### *Amasa* Lea, 1894

#### Amasa

Taxon classificationAnimaliaColeopteraCurculionidae

Lea, 1894

Amasa Lea, 1894: 322.
Pseudoxyleborus Eggers, 1930: 206. Synonymy: Wood 1984: 223.
Anaxyleborus Wood, 1980: 90. Synonymy: Wood 1983: 647.

##### Type species.

*Amasa thoracica* Lea, 1894 = *Tomicus truncatus* Erichson, 1842; monotypy.

##### Diagnosis.

2.5–5.0 mm, 2.11–3.4× as long as wide. *Amasa* is distinguished by the declivity truncate, margined with a circumdeclivital ring; antennal club flattened, types 4 or 5 (typically type 4), club sutures sinuate, two sutures visible on posterior face; protibiae typically slender, inflated and granulate on posterior face (rarely distinctly triangular or unarmed on posterior face); anterior margin of pronotum with a row of serrations; scutellum flat, flush with elytral surface; declivital face with three striae; procoxae contiguous or narrowly separated; and mycangial tufts absent.

##### Similar genera.

*Cyclorhipidion*, *Pseudowebbia*, *Truncaudum*, *Webbia*, *Xylosandrus*.

##### Distribution.

Distributed throughout Asia and Australasia, also occurring in Madagascar. One species has been introduced to Brazil, Chile and Uruguay (Flechtmann and Cognato 2011; Gómez et al. 2017; Kirkendall 2018).

##### Gallery system.

This usually comprises a short radial tunnel leading to a single, large, flat brood chamber, extending in the longitudinal plane.

##### Remarks.

*Amasa* is easily confused with other species possessing truncate declivities in the genera listed above. Most species can be readily distinguished by the type 4 antennal club with sinuate sutures and the presence of only three striae on the declivital face.

Previous morphological studies of *Amasa* have suggested that species are very morphologically variable (Hulcr and Cognato 2013). As a result, many species were considered conspecific and part of a morphological continuum. Molecular data generated as part of this study has demonstrated that *Amasa* species are actually morphologically conserved even across broad ranges (Smith et al. 2020). *Amasa* species outside our coverage area are thus in need of revision. Potentially much of the diversity is awaiting discovery.

#### Key to *Amasa* species (females only)

**Table:** 

1	Antennal club type 5, with sutures almost or completely reduced, club covered with pubescence (Fig. 3)	**2**
–	Antennal club type 4, with sutures visible and partly corneous (Fig. 3)	**5**
2	Eye completely divided (Fig. 7D); declivity with striae impressed, and all interstriae densely punctate; 4.5 mm	*** beesoni ***
–	Eye moderately to strongly emarginate (Fig. 7F), other characters variable; 2.0–3.9 mm	**3**
3	Declivital face with strial and interstrial punctures deeply confused, indistinguishable; larger, 3.7–3.9 mm	*** aspersa ***
–	Declivital face with three striae clearly indicated on each elytron; smaller, 2.0–3.2 mm	**4**
4	Stout, 2.0× as long as wide; pronotum from dorsal view round and stout, type 1. 2.0–2.4 mm	*** cylindrotomica ***
–	Slender, 2.8–3.2× as long as wide; pronotum from dorsal view elongate, type 7. 2.0–3.2 mm	*** eugeniae ***
5	Declivital striae 2 not equidistant between 1 and 3 (Fig. 10I)	**6**
–	Declivital striae 2 equidistant between 1 and 3 (Fig. 7K)	**7**
6	Declivital striae 1 clearly laterally displaced, striae 2 nearly touching striae 1, striae 3 displaced near circumdeclivital margin (Fig. 9I)	***lini* sp. nov.**
–	Declivital striae 2 medially displaced toward striae 1; distance between striae 1 and 3 twice the distance between 1 and 2 (Fig. 10I)	***youlii* sp. nov.**
7	Declivity not granulate, or only interstriae 1 granulate, or only interstriae 1 and 2	**8**
–	Declivity with all interstriae granulate	**10**
8	All declivital interstriae smooth, never granulate; larger, 4.5–4.8 mm	*** opalescens ***
–	Interstriae 1, or 1 and 2 granulate; smaller, 2.9–3.6 mm	**9**
9	Declivital face flat, strongly shagreened to opalescent; interstriae 1 granulate (typically near apex)	*** schlichii ***
–	Declivital face convex, strongly shiny; interstriae 1 and 2 moderately inflated from apex to near midpoint of declivity	***gibbosa* sp. nov.**
10	Declivital face setose, sparsely to moderately covered with recumbent or semi-recumbent hair-like setae, sometimes difficult to see	**11**
–	Declivital face without setae	**13**
11	Declivity strongly shiny; interstriae very finely setose, setae semi-erect; larger, 4.3–4.5 mm	*** concitata ***
–	Declivity shagreened, dull; interstriae sparsely to moderately covered with semi-recumbent hair-like setae; smaller, 2.5–3.0 mm	**12**
12	Setae on declivital interstriae short, less than 1/2 width of an interstria; margin of circumdeclivital ring with short, erect, hair-like setae	***galeoderma* sp. nov.**
–	Setae on declivital interstriae approximately as long as the width of an interstria; margin of circumdeclivital ring with long, erect, bristle-like setae	*** versicolor ***
13	Declivity strongly shiny; declivital interstriae 1 carinate along at least apical 1/2	***tropidacron* sp. nov.**
–	Declivity shagreened, dull; declivital interstriae 1 granulate	**14**
14	Declivital interstriae convex; larger, 3.4 mm, and more elongate, 3.4× as long as wide	***cycloxyster* sp. nov.**
–	Declivital interstriae 2–4 flat; smaller, 2.8–3.2 mm, and stouter, 2.3–2.4× as long as wide	*** resecta ***

#### Amasa aspersa

Taxon classificationAnimaliaColeopteraCurculionidae

(Sampson, 1921)

Fig. 7A, B, I

Xyleborus aspersus Sampson, 1921: 31.
Amasa aspersus [*sic*] (Sampson): Wood and Bright 1992: 682.

##### Type material.

***Holotype*** (NHMUK).

##### Diagnosis.

3.7–3.9 mm long (mean = 3.82 mm; n = 2); 2.11–2.17× as long as wide. This species is distinguished by the dense and strongly confused declivital strial and interstrial punctures with striae and interstriae indistinguishable.

##### Similar species.

None.

##### Distribution.

Brunei, East & West Malaysia, Thailand.

##### Host plants.

All host records are from the genus *Eugenia* (Myrtaceae), and the species appears to have a fixed host association with this family (Browne 1961b).

**Figure 7.**
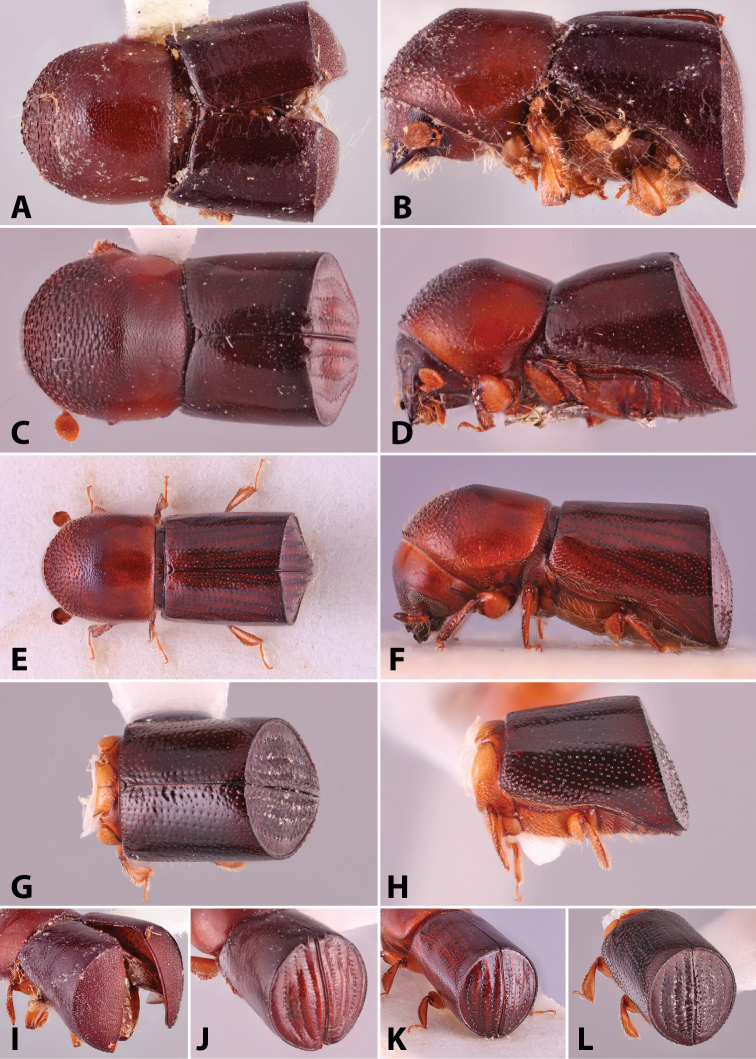
Dorsal, lateral and declivital view of *Amasa aspersa*, 3.7–3.9 mm (**A, B, I**), *A. beesoni* paratype, 5.0 mm (**C, D, J**), *A. concitata*, 4.3–4.5 mm (**E, F, K**), and *A. cycloxyster* holotype, 3.4 mm (**G, H, L**).

#### Amasa beesoni

Taxon classificationAnimaliaColeopteraCurculionidae

(Eggers, 1930)

Fig. 7C, D, J

Pseudoxyleborus beesoni Eggers, 1930: 207.
Amasa beesoni (Eggers): Wood 1984: 223.

##### Type material.

***Holotype*** (FRI), ***paratype*** (NHMW, 1).

##### Diagnosis.

5.0 mm long (n = 1); 2.17× as long as wide. This species is distinguished from all other species in Southeast Asia, except the Malaysian species, *A. glauca* (Sampson, 1921), by the completely divided eye. It is easily distinguished from *A. glauca* by the presence of a small tooth on the first interstriae at the top of the declivity, the impressed declivital striae, and densely punctured declivital interstriae.

##### Similar species.

*Amasa glauca* (from Indomalayan region), *A. opalescens*.

##### Distribution.

‘Borneo’, West Malaysia, Myanmar, Thailand.

##### Host plants.

The only host records are from the family Sapindaceae (*Nephelium*, *Xerospermum*), and the species may have a fixed host association with this family (Browne 1961b).

#### Amasa concitata

Taxon classificationAnimaliaColeopteraCurculionidae

(Schedl, 1969)

Fig. 7E, F, K

Xyleborus concitatus Schedl, 1969a: 214.
Amasa concitatus [*sic*] (Schedl): Wood and Bright 1992: 682.

##### Type material.

***Holotype*** (PPST). Not examined.

##### New records.

China: Jiangxi, Longnan County, Jiulianshan, 24.58; 114.44, 382 m, 1.vii.2018, Lv-Jia, S.C. Lai, ex unknown [host tree] (LYLC, 1).

##### Diagnosis.

4.3–4.5 mm long (n = 2); 2.32–2.5× as long as wide. This species is distinguished by the pronotum appearing basic (type 2) when viewed dorsally, anterior margin serrate; declivital surface smooth, strongly shiny; large size; declivital interstriae very finely setose, setae semi-erect; declivital face convex towards suture; declivital interstriae 1 inflated from apex to near midpoint of declivity; declivital striae 1–3 approximately equidistant.

##### Similar species.

*Amasa gibbosa*, *A. lini*, *A. tropidacron*, *A. youlii*.

##### Distribution.

China* (Jiangxi), Taiwan.

##### Host plants.

Recorded only from ‘Formosan hardwood’ and ‘angiosperm wood’ (Beaver and Liu 2010).

#### Amasa cycloxyster sp. nov.

Taxon classificationAnimaliaColeopteraCurculionidae

http://zoobank.org/AAE768AB-65F5-4427-91F5-1FA7CEFFC93A

Fig. 7G, H, L

##### Type material.

***Holotype***, female, Thailand: Surat Thani, Khao Sok National Park, 22.iii.2006, Hulcr et al., ex “Mai Naun Pang” tree (MSUC).

##### Diagnosis.

3.4 mm long (n = 1); 3.4× as long as wide. The species is distinguished by the pronotum appearing basic (type 2) when viewed dorsally, anterior margin serrate; declivital surface shagreened, dull, opaque; declivity glabrous; declivital interstriae 1–3 multiseriate granulate, granules strongly confused; and declivital interstriae convex.

##### Similar species.

*Amasa galeoderma*, *A. resecta*, *A. schlichii*, *A. versicolor*.

##### Description

**(female).** 3.4 mm long (n = 1); 3.4× as long as wide. Body bicolored: pronotum, head, legs, antennae and abdomen orange, elytra dark brown. ***Head***: epistoma entire, transverse, with a row of hair-like setae. Frons weakly convex to upper level of eyes; median impression between eyes; surface shagreened, impunctate, alutaceous, asperate; asperities longitudinal, smaller, rounder, denser above epistoma, increasing in size and length and decreasing in density dorsally and laterally. Eyes very deeply emarginate just above antennal insertion, upper part smaller than lower part. Submentum triangular, deeply impressed. Antennal scape regularly thick, as long as club. Pedicel as wide as scape, shorter than funicle. Funicle 4-segmented, segment 1 shorter than pedicel. Club approximately circular and flat, type 4; segment 1 corneous, transverse on anterior face, occupying basal 1/5; segment 2 narrow, larger than segment 1, corneous; segments 1–3 present on posterior face. ***Pronotum***: 1.08× as long as wide. In dorsal view basic and parallel-sided, type 2, sides parallel in basal 1/2, rounded anteriorly; anterior margin with a row of five serrations. In lateral view basic, type 0, disc flat, summit at midpoint. Anterior slope shagreened, with densely spaced, fine asperities, becoming lower and more strongly transverse towards summit, bearing long, fine, semi-recumbent, hair-like setae. Disc shiny, alutaceous, impunctate, glabrous. Lateral margins obliquely costate. Base transverse, posterior angles narrowly rounded. ***Elytra***: 1.4× as long as wide, 1.3× as long as pronotum. Scutellum moderately sized, broad, linguiform, flush with elytra, flat, shiny. Elytral base transverse, edge oblique, humeral angles rounded, parallel-sided in basal 3/4, then sharply angulate to apex. Disc ascending posteriorly, shiny, glabrous; striae and interstriae laterally diverging from base to declivital summit; striae not impressed, punctures separated by 1–4 diameters of a puncture; interstriae flat, finely punctate, punctures 1/2 the size of strial punctures, strongly confused. Declivity truncate, face convex, strongly shagreened, dull, glabrous; three striae present, striae moderately impressed, equidistant, strial punctures shiny, very large, shallow, much larger than on disc, punctures subcontiguous to spaced by two diameters of a puncture; interstriae impunctate, convex, interstriae 1 more strongly convex, interstriae 1–3 multiseriate granulate, granules strongly confused. Posterolateral margin forming a circumdeclivital carina, carina glabrous. ***Legs***: procoxae contiguous; prosternal coxal piece bulging. Protibiae slender, broadest at apical 1/3; posterior face inflated, coarsely granulate; apical 1/2 of outer margin with six small socketed denticles, their length as long as basal width. Meso- and metatibiae broad, flattened, outer margins evenly rounded with 11 small and nine small to minute socketed denticles, respectively; posterior faces unarmed; anterior faces finely granulate.

##### Etymology.

G. *kyklos* = circle; *xyster* = rasp. In reference to acute granules on the round declivital face. A noun in apposition.

##### Distribution.

Thailand.

##### Host plants.

Unknown.

##### Remarks.

The holotype specimen is a DNA voucher, SAX40. The head and pronotum were separated from the specimen prior to DNA extraction and point mounted with the elytra.

#### Amasa cylindrotomica

Taxon classificationAnimaliaColeopteraCurculionidae

(Schedl, 1939)

Fig. 8A, B, I

Pseudoxyleborus cylindrotomicus Schedl, 1939b: 40.
Xyleborus cylindrotomicus (Schedl): Schedl 1942c: 6.
Xylosandrus cylindrotomicus (Schedl): Wood 1989: 177.
Amasa cylindrotomica (Schedl): Dole and Cognato 2010: 525.
Xyleborus semitruncatus Schedl, 1942c: 35. Synonymy: Schedl 1951a: 79; Wood 1989: 177.
Xyleborus truncatellus Schedl, 1951a: 79. Synonymy: Kalshoven 1959a: 95.
Xyleborus jucundus Schedl, 1954a: 138 (new name for Xyleborus truncatellus Schedl, 1951 nec Schedl 1949). Synonymy: Kalshoven 1959a: 95.

##### Type material.

***Lectotype*** (NHMW). Not examined.

##### Diagnosis.

2.1–2.4 mm long (mean = 2.25 mm; n = 2); 2.0× as long as wide (Sittichaya et al. 2019). This species is distinguished by its minute size, stout form with the pronotum approximately as long as the elytra; declivital surface shagreened, dull, glabrous; and antennal club type 5.

##### Similar species.

*Amasa opalescens*.

##### Distribution.

Indonesia (Java, Sumatra), Thailand.

##### Host plants.

Recorded from *Syzygium aromaticum* (Myrtaceae) (Sittichaya et al. 2019).

**Figure 8.**
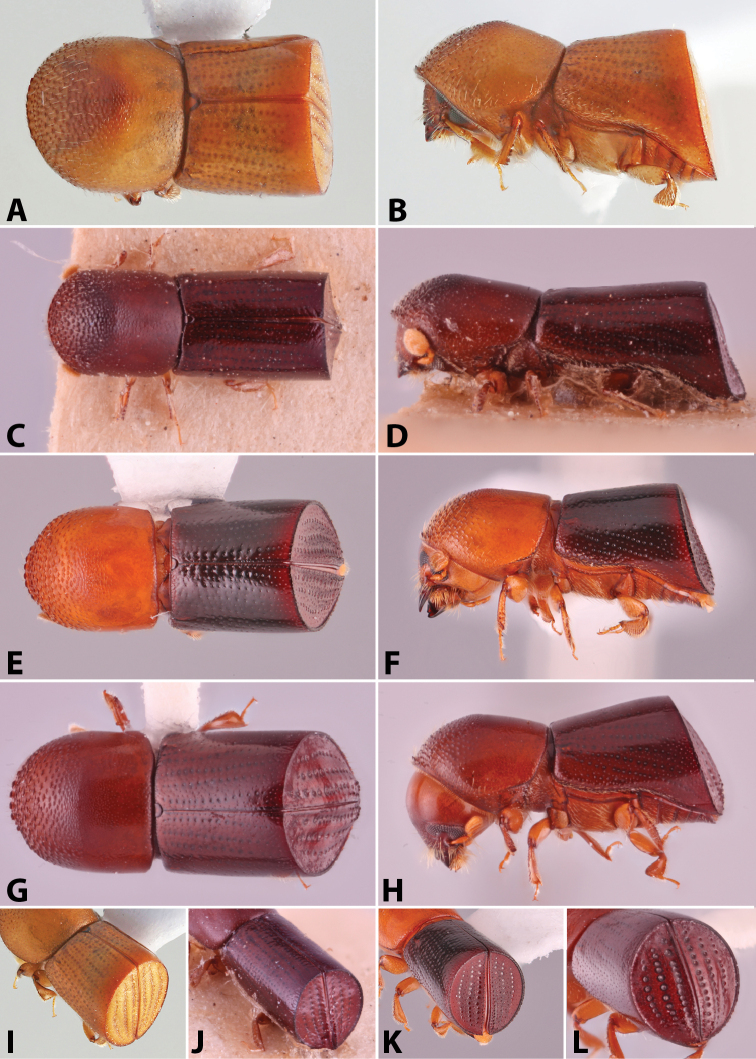
Dorsal, lateral and declivital view of *Amasa cylindrotomica*, 2.1–2.4 mm (**A, B, I**), *A. eugeniae* paratype, 2.8–3.2 mm (**C, D, J**), *A. galeoderma* holotype, 3.0 mm (**E, F, K**), and *A. gibbosa* holotype, 3.5–3.6 mm (**G, H, L**).

#### Amasa eugeniae

Taxon classificationAnimaliaColeopteraCurculionidae

(Eggers, 1930)

Fig. 8C, D, J

Xyleborus eugeniae Eggers, 1930: 183.
Amasa eugeniae (Eggers): Wood and Bright 1992: 683.

##### Type material.

***Holotype*** (FRI), ***paratypes*** (FRI, 1; NHMW, 1; NMNH, 1).

##### Diagnosis.

2.8–3.2 mm long (mean = 2.65 mm; n = 5); 2.8–3.2× as long as wide. This species is distinguished by its very elongate body and pronotum (type 7) when viewed dorsally; antennal club type 5; and declivital surface shagreened, dull.

##### Similar species.

*Cyclorhipidion amasoides*.

##### Distribution.

India (Uttarakhand, West Bengal), Sri Lanka.

##### Host plants.

Recorded from two species of *Eugenia* (Myrtaceae), and *Elaeocarpus* (Elaeocarpaceae) (Maiti and Saha 2004).

#### Amasa galeoderma sp. nov.

Taxon classificationAnimaliaColeopteraCurculionidae

http://zoobank.org/77A6AA8D-E16C-4FBD-ADA5-A77AE0CACD50

Fig. 8E, F, K

##### Type material.

***Holotype***, female, Vietnam: Dong Nai, Cat Tien N.P., 11.44221, 107.43114, 379 m, 20.ii.2017, VN79, A.I. Cognato, T.A. Hoang, ex 4 cm diameter branch (MSUC). ***Paratypes***, female, as holotype (NHMW, 1; NHMUK, 1; NMNH, 1; VMNH, 1).

##### Diagnosis.

3.0 mm long (mean = 3.0 mm; n = 5); 2.5× as long as wide. This species is distinguished by the pronotum appearing basic (type 2) when viewed dorsally, anterior margin serrate; declivital surface shagreened, dull, opaque; declivital interstriae granulate, granules multiseriate, confused; declivital interstriae 1 moderately covered with semi-recumbent fine hair-like setae, less than 1/2 width of an interstria; and circumdeclivital carina margin setose, setae short, erect, hair-like.

##### Similar species.

*Amasa cycloxyster*, *A. resecta*, *A. schlichii*, *A. versicolor*.

##### Description

**(female).** 3.0 mm long (mean = 3.0 mm; n = 5); 2.5× as long as wide. Body bicolored: pronotum, head, legs and antennae orange, elytra and abdomen dark brown. ***Head***: epistoma entire, transverse, with a row of hair-like setae. Frons weakly convex to upper level of eyes; median impression between eyes; surface shagreened, impunctate, alutaceous, asperate; asperities longitudinal, smaller, rounder, denser above epistoma, increasing in size and length and decreasing in density dorsally and laterally. Eyes very deeply emarginate just above antennal insertion, upper part smaller than lower part. Submentum triangular, deeply impressed. Antennal scape regularly thick, as long as club. Pedicel as wide as scape, shorter than funicle. Funicle 4-segmented, segment 1 shorter than pedicel. Club approximately circular and flat, type 4; segment corneous, 1 convex on anterior face, occupying approximately basal 1/4; segment 2 narrow, larger than segment 1, corneous; segments 1–3 present on posterior face. ***Pronotum***: 1.0× as long as wide. In dorsal view basic and parallel-sided, type 2, sides parallel in basal 1/2, rounded anteriorly; anterior margin with a row of 5–7 serrations. In lateral view basic, type 0, disc flat, summit at midpoint. Anterior slope strongly shiny, with widely spaced, moderate asperities, becoming lower and more strongly transverse towards summit, bearing long, fine, semi-recumbent, hair-like setae. Disc shiny, alutaceous, sparsely finely punctate, glabrous. Lateral margins obliquely costate. Base transverse, posterior angles narrowly rounded. ***Elytra***: 1.35× as long as wide, 1.25× as long as pronotum. Scutellum moderately sized, broad, linguiform, flush with elytra, flat, shiny. Elytral base transverse, edge oblique, humeral angles rounded, parallel-sided in basal 3/4, then sharply angulate to apex. Disc ascending posteriorly, shiny, glabrous; striae and interstriae laterally diverging from base to declivital summit; striae not impressed, punctures separated by 2–3 diameters of a puncture; interstriae flat, finely uniseriate punctate, punctures 1/3 size of strial punctures. Declivity truncate, face flattened, strongly shagreened, dull, glabrous; three striae present, striae moderately impressed, striae 2 equidistant between striae 1 and 3, strial punctures shiny, very large, shallow, much larger than on disc, punctures subcontiguous; interstriae impunctate, convex, interstriae 1 more strongly convex, interstriae 1–3 multiseriate granulate, granules multiseriate, confused, interstriae 1 moderately covered with fine, semi-recumbent, hair-like setae, less than 1/2 width of an interstria. Posterolateral margin forming a circumdeclivital carina; carina setose, setae short, erect, hair-like. ***Legs***: procoxae contiguous, prosternal coxal piece flat, inconspicuous. Protibiae slender, broadest at apical 1/3; posterior face inflated, finely granulate; apical 1/2 of outer margin with six small socketed denticles, their length as long as basal width. Meso- and metatibiae broad, flattened, outer margins evenly rounded with nine and 11 small socketed denticles, respectively, posterior faces unarmed; anterior faces finely granulate.

##### Etymology.

G. *galeos* = shark; *derma* = skin. In reference to the shagreened face of the declivity. Noun in apposition.

##### Distribution.

Vietnam.

##### Host plants.

Unknown.

#### Amasa gibbosa sp. nov.

Taxon classificationAnimaliaColeopteraCurculionidae

http://zoobank.org/359F611F-95A0-4631-94B6-67F1A8DC5BCD

Fig. 8G, H, L

##### Type material.

***Holotype***, female, Thailand: Kanchanaburi, Thong Pha Phoom Dist., Phu Yae subdist[rict], 400 m, 14.944N, 98.674E, 16.vii.2002, Cognato, Gillogly, Harlin (MSUC). ***Paratypes***, female, as holotype (MSUC, 1; NHMUK, 1; RABC, 1); Suratthani, Khao Sok N.P., 1.ii.2015, 19°21'41.8"N, 98°55'03.4"E, W. Sittichaya, ex ethanol baited trap, tropical rain forest (MSUC, 1).

##### Diagnosis.

3.5–3.6 mm long (mean = 3.53 mm; n = 3); 2.33–2.41× as long as wide. This species is distinguished by the pronotum appearing basic (type 2) when viewed dorsally, anterior margin serrate; declivital surface glabrous, smooth, strongly shiny; moderate size; declivital face convex, interstriae 1 and 2 moderately inflated from apex to near midpoint of declivity; declivital striae 1–3 approximately equidistant.

##### Similar species.

*Amasa concitata*, *A. lini*, *A. tropidacron*, *A. youlii*.

##### Description

**(female).** 3.5–3.6 mm long (mean = 3.53 mm; n = 3); 2.33–2.41× as long as wide. Body dark red-brown. Legs and antennae light brown. ***Head***: epistoma entire, transverse, with a row of hair-like setae. Frons weakly convex to upper level of eyes; median impression between eyes; surface shagreened, impunctate, alutaceous, asperate; asperities longitudinal, smaller, rounder, denser above epistoma, increasing in length and decreasing in width and density dorsally. Eyes very deeply emarginate just above antennal insertion, upper part smaller than lower part. Submentum triangular, deeply impressed. Antennal scape regularly thick, as long as club. Pedicel as wide as scape, shorter than funicle. Funicle 4-segmented, segment 1 shorter than pedicel. Club approximately circular and flat, type 4; segment 1 corneous, convex on anterior face, occupying approximately basal 1/4; segment 2 broad, larger than segment 1, corneous; segments 1–3 present on posterior face. ***Pronotum***: 1.02× as long as wide. In dorsal view basic and parallel-sided, type 2, sides parallel in basal 1/2, rounded anteriorly; anterior margin with a row of six serrations. In lateral view basic, type 0, disc flat, summit at midpoint. Anterior slope strongly shiny with densely spaced, fine asperities, becoming lower and more strongly transverse towards summit, bearing long, fine, semi-recumbent, hair-like setae. Disc shiny, alutaceous, densely finely punctate behind summit, punctures decreasing in density toward base, glabrous. Lateral margins obliquely costate. Base transverse, posterior angles narrowly rounded. ***Elytra***: 1.48× as long as wide, 1.45× as long as pronotum. Scutellum moderately sized, broad, linguiform, flush with elytra, flat, shiny. Elytral base transverse, edge oblique, humeral angles rounded, parallel-sided in basal 3/4, then sharply angulate to apex. Disc ascending posteriorly, shiny, glabrous; striae and interstriae laterally diverging from base to declivital summit; striae not impressed, punctures separated by five diameters of a puncture; interstriae flat, finely punctate, punctures 1/2 size of strial punctures, strongly confused. Declivity truncate, face convex, strongly shiny, smooth, glabrous; three striae present, striae weakly impressed, striae 2 equidistant between striae 1 and 3, strial punctures subshiny, very large and deep, much larger and deeper than on disc, punctures subcontiguous to spaced by one diameter of a puncture; interstriae impunctate, convex, interstriae 1 and 2 moderately inflated from apex to near midpoint of declivity; apical 1/4 of interstriae 1 and 2 with a row of uniseriate rugae. Posterolateral margin forming a circumdeclivital carina; carina setose, setae short, erect hair-like. ***Legs***: procoxae contiguous; prosternal coxal piece flat, inconspicuous. Protibiae distinctly triangular, broadest at apical 1/3; posterior face inflated, coarsely granulate; apical 1/2 of outer margin with six or seven small socketed denticles, their length as long as basal width. Meso- and metatibiae broad, flattened; outer margins evenly rounded with 11 and nine small to minute socketed denticles, respectively; posterior faces unarmed; anterior faces finely granulate.

##### Etymology.

L. *gibbosa* = humped. In reference to the rather bulging declivity. A variable adjective.

##### Distribution.

Thailand.

##### Host plants.

Unknown.

#### Amasa lini sp. nov.

Taxon classificationAnimaliaColeopteraCurculionidae

http://zoobank.org/AE746EB3-4A92-4977-BD5B-DF3BC45FE625

Fig. 9A, B, I

##### Type material.

***Holotype***, female, Taiwan: Nantou Dist., Sun Moon Lake, 23.vi.2016, C.-S. Lin (TARI).

##### Diagnosis.

3.5 mm long (n = 1); 2.33× as long as wide. This species is distinguished by the pronotum appearing basic (type 2) when viewed dorsally, anterior margin serrate; declivital surface smooth, shiny; large size; declivity glabrous; declivital interstriae 1 strongly tumescent and granulate; declivital striae 1 strongly laterally displaced, nearly touching striae 2, striae 3 displaced to near circumdeclivital carina margin; and declivital striae 2 not appearing equidistant between striae 1 and 3.

##### Similar species.

*Amasa concitata*, *A. gibbosa*, *A. tropidacron*, *A. youlii*.

##### Description

**(female).** 3.5 mm long (n = 1); 2.33× as long as wide. Body bicolored: pronotum reddish, elytra and abdomen dark brown, head, legs, and antennae light brown. ***Head***: epistoma entire, transverse, with a row of hair-like setae. Frons weakly convex to upper level of eyes; median impression between eyes; surface shagreened, impunctate, alutaceous, asperate; asperities longitudinal, larger, rounder, denser above epistoma, increasing in length and decreasing in width and density dorsally. Eyes very deeply emarginate just above antennal insertion, upper part smaller than lower part. Submentum triangular, deeply impressed. Antennal scape regularly thick, as long as club. Pedicel as wide as scape, shorter than funicle. Funicle 4-segmented, segment 1 shorter than pedicel. Club approximately circular and flat, type 4; segment 1 corneous, sinuate on anterior face, occupying approximately 1/5 of club; segment 2 narrow, larger than segment 1, corneous; segments 1–3 present on posterior face. ***Pronotum***: 1.4 × as long as wide. In dorsal view basic and parallel-sided, type 2, sides parallel in basal 1/2, rounded anteriorly; anterior margin with a row of eight serrations. In lateral view basic, type 0, disc flat, summit at midpoint. Anterior slope shagreened, with densely spaced, fine asperities, becoming lower and more strongly transverse towards summit, bearing long, fine, semi-recumbent hair-like setae. Disc subshiny, alutaceous, densely, finely punctate, glabrous. Lateral margins obliquely costate. Base transverse, posterior angles broadly rounded. ***Elytra***: 1.4× as long as wide, 1.43× as long as pronotum. Scutellum moderately sized, broad, linguiform, flush with elytra, flat, shiny. Elytral base transverse, edge oblique, humeral angles rounded, parallel-sided in basal 3/4, then sharply angulate to apex. Disc flat, shiny, glabrous; striae and interstriae laterally diverging from base to declivital summit; striae not impressed, punctures separated by 3–5 diameters of a puncture; interstriae flat, finely punctate, punctures 1/3 size of strial punctures, strongly confused. Declivity truncate, face convex, smooth, shiny, glabrous; three striae present, striae weakly impressed, striae 1 strongly laterally displaced, striae 2 nearly touching striae 1, striae 3 displaced to near circumdeclivital carina, strial punctures dull, small, shallow, larger than on disc, punctures spaced by a diameter of a puncture; interstriae impunctate, convex, interstriae 1 strongly tumescent and granulate, granules strongly confused, apical 1/6 of interstriae 1 carinate. Posterolateral margin forming a circumdeclivital carina; carina glabrous. ***Legs***: procoxae contiguous, prosternal coxal piece flat, inconspicuous. Protibiae slender, broadest at apical 1/3; posterior face inflated, finely granulate; apical 1/2 of outer margin with five small socketed denticles, their length as long as basal width. Meso- and metatibiae broad, flattened, outer margins evenly rounded with 11 and nine small socketed denticles, respectively, posterior faces unarmed; anterior faces finely granulate.

##### Etymology.

The species is named for Mr. Ching-Shan Lin, the collector, for his contributions to our knowledge of bark and ambrosia beetles. Noun in genitive.

##### Distribution.

Taiwan.

##### Host plants.

Unknown.

**Figure 9.**
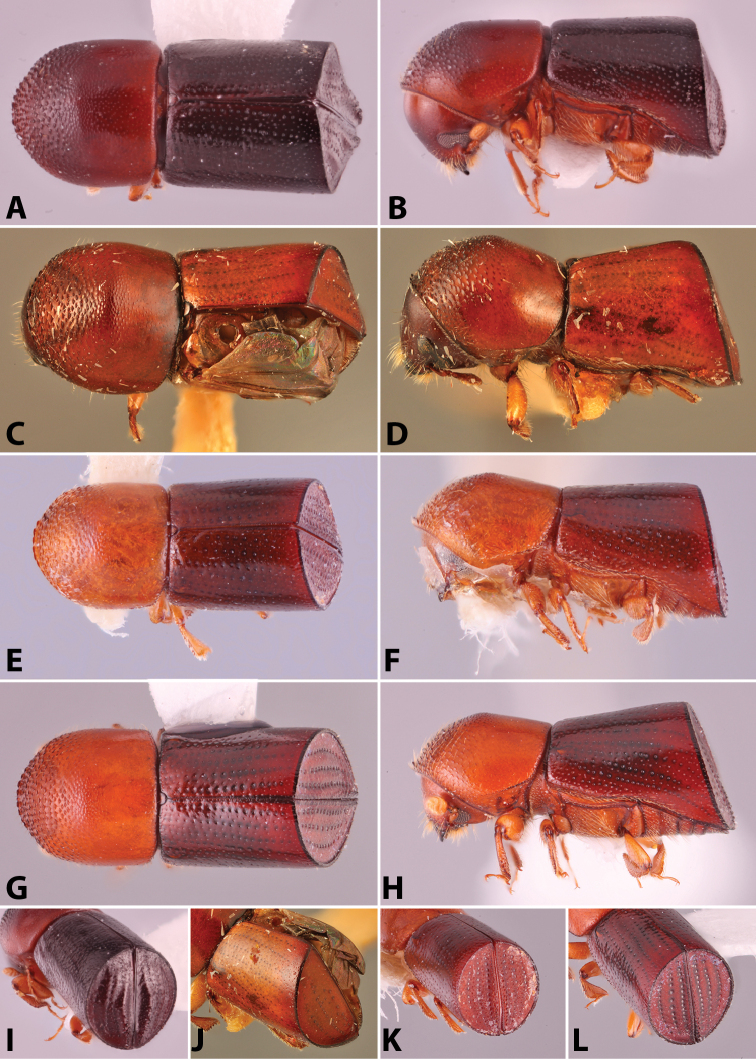
Dorsal, lateral and declivital view of *Amasa lini* holotype, 3.5 mm (**A, B, I**), *A. opalescens* lectotype, 4.5–4.8 mm (**C, D, J**), *A. resecta*, 2.85–3.2 mm (**E, F, K**), and *A. schlichii*, 2.9–3.5 mm (**G, H, L**).

#### Amasa opalescens

Taxon classificationAnimaliaColeopteraCurculionidae

(Schedl, 1937)

Fig. 9C, D, J

Xyleborus opalescens Schedl, 1937a: 550.
Amasa opalescens (Schedl): Wood and Bright 1992: 684.

##### Type material.

***Lectotype*** (NHMW).

##### Diagnosis.

4.5–4.8 mm long (4.7 mm long; n = 3); 2.4–2.5× as long as wide. This species is distinguished by its large size; pronotum rounded, robust from lateral view (type 5); declivital interstriae 1 unarmed (lacking granules) and flat; declivital strial punctures very large, irregularly spaced; and declivital surface appearing smooth and opalescent.

##### Similar species.

*Amasa beesoni*, *A. cylindrotomica*, *A. schlichii*.

##### Distribution.

East & West Malaysia, Thailand, Vietnam.

##### Host plants.

Recorded only from species of *Eugenia* and *Tristania* (Myrtaceae), and possibly with a fixed association with this family (Browne 1961b).

#### Amasa resecta

Taxon classificationAnimaliaColeopteraCurculionidae

(Eggers, 1923)

Fig. 9E, F, K

Xyleborus abruptus Eggers, 1923: 169.
Xyleborus resectus Eggers, 1927a: 391 (new name for X. abruptus Eggers, 1923 nec Sampson 1914).
Amasa resectus [*sic*] (Eggers): Wood and Bright 1992: 684.
Xyleborus opacicauda Eggers, 1940: 136. Synonymy: Kalshoven 1959b: 159.

##### Type material.

***Syntype****Xyleborus resectus* (MIZ, 1).

##### New records.

China: Hainan, Wu-zhi-shan Town, 18.902N, 109.663E, 703 m, 2.xii.2016, Tian-Shang, Lv-Jia (RABC, 2).

##### Diagnosis.

2.85–3.2 mm long (mean = 2.94 mm; n = 4); 2.29–2.38× as long as wide. This species is distinguished by the pronotum appearing basic (type 2) when viewed dorsally, anterior margin serrate; declivital surface shagreened, dull, opaque; declivity glabrous; declivital interstriae 1–3 multiseriate granulate, granules strongly confused; and declivital interstriae 2–4 flat.

##### Similar species.

*Amasa cycloxyster*, *A. galeoderma*, *A. schlichii*, *A. versicolor*, *A. youlii*.

##### Distribution.

China (Hainan), Indonesia (Java, Sumatra), East Malaysia, New Guinea, Sri Lanka, Thailand.

##### Host plants.

Recorded by Kalshoven (1959b) from five genera in five different families. Evidently polyphagous.

##### Remarks.

Hulcr and Cognato (2013) synonymized *Xyleborus fulgens* Schedl, 1975c with this species, but we believe it to be distinct. Hence it is not included in the list of synonyms.

#### Amasa schlichii

Taxon classificationAnimaliaColeopteraCurculionidae

(Stebbing, 1907)

Fig. 9G, H, L

Acanthotomicus truncatus Stebbing, 1907: 40.
Xyleborus schlichii Stebbing, 1914: 592 (new name for Xyleborus (Acanthotomicus) truncatus (Stebbing, 1907) nec Erichson 1842).
Amasa schlichi [*sic*] (Stebbing): Wood 1989: 169.
Xyleborus glaber Eggers, 1930: 185. Synonymy: Wood 1989: 169.
Xyleborus uniseriatus Eggers, 1936b: 89. Synonymy: Schedl 1963b: 268.
Xyleborus verax Schedl, 1939b: 43. Synonymy: Kalshoven 1959a: 95.

##### Type material.

***Holotype***, *Xyleborus glaber* (FRI), ***paratype*** (NHMW, 1). ***Syntype****Xyleborus schlichii* (FRI, 1).

##### New records.

China: Hong Kong, Tai Po Kau, vi.2017, J. Skelton (MSUC, 1). S-Yunnan, Xishuangbanna, Sanchahe Nat. Res., 22°09.784'N, 100°52.256'E, 2186 m, 29–30.v.2008, A.I. Cognato (MSUC, 2); as previous except: 23 km NW Jinghong, vic. Na Ban village (NNNR), 22°10'N, 100°39'E, 700–1000 m, v–vii. 2009, L. Meng (RABC, 2). Japan: Okinawa Pref., Iriomote-jima Island, 26.vi.2016, H. Kajimura, ex *Machilus thunbergii* (MSUC, 1). Vietnam: Cao Bang, 22°33.9981'N, 105°52.591'E, 1051 m, 12–17.iv.2014, VN11, Cognato, Smith, Pham, ex FIT (MSUC, 3). N. Ninh Binh, 90 km SW Hanoi, Cuc Phuong N.P., primate rescue centre, 20°14'24"N, 105°42'53"E, 190 m, 25.iv.2012, A. Weigel, ex light trap (NKME, 1). Thua Thien-Hue, Bach Ma N.P., 16.22897, 107.85349, 415 m, 15.ii.2017, VN61, A.I. Cognato, T.A. Hoang, ex 5 cm diameter branch (MSUC, 1).

##### Diagnosis.

2.9–3.5 mm long (mean = 3.21 mm; n = 10); 2.23–2.54× as long as wide. This species is distinguished by the pronotum appearing basic (type 2) when viewed dorsally, anterior margin serrate; declivital surface shagreened to opalescent, dull, opaque; declivity glabrous; and declivital interstriae 1 granulate (typically near apex), interstriae 2 and 3 unarmed.

##### Similar species.

*Amasa cycloxyster*, *A. galeoderma*, *A. resecta*, *A. versicolor*, *A. youlii*.

##### Distribution.

China* (Hong Kong, Yunnan), India (Assam, West Bengal), Indonesia (Java), Japan*, East & West Malaysia, Thailand, Vietnam*.

##### Host plants.

Apparently polyphagous (Beeson 1961; Beaver and Browne 1979; Maiti and Saha 2004).

##### Remarks.

This species had previously been considered to be extremely morphologically variable (Hulcr and Cognato 2013) but Cognato et al. (2020b) and Smith et al. (2020) demonstrated that very little intraspecific morphological variation is present and removed the Papua New Guinean species *A. striatotruncata* (Schedl, 1936) and *A. umbratula* (Schedl, 1975) from synonymy.

Wood (1989: 169) considered *Xyleborus glaber* to be a synonym of *X. schlichii*. Beaver et al. (2014: 20) later considered it to be a distinct species. Upon our examination of the photos of the holotype and a paratype specimen we found this species to be conspecific with *Amasa schlichii* and it is here returned to synonymy.

#### Amasa tropidacron sp. nov.

Taxon classificationAnimaliaColeopteraCurculionidae

http://zoobank.org/D6F42632-2E07-47D3-BBDA-8C97934C7E12

Fig. 10A, B, G

##### Type material.

***Holotype***, female, Japan: Okinawa, Iriomote-jima, Isd. Code. 1, 9.xi.2012, Kajimura (MSUC). ***Paratypes***, female, as holotype (MSUC, 1); as previous except: Yona, 1.xi.2010, J. Hulcr, ex *Castanopsis*, uffeID 7348 (UFFE, 2), uffeID 7389 (UFFE, 4); Vietnam: Ninh Binh, Cuc Phuong N.P., Mac Lake, 20°15'29.0"N, 105°42'27.5"E, 155 m, 4–7.v.2009, J.B. Heppner, ex blacklight trap (FSCA, 1).

##### Diagnosis.

2.5–2.8 mm long (mean = 2.65 mm; n = 2); 2.5–2.54× as long as wide. This species is distinguished by the pronotum appearing basic (type 2) when viewed dorsally, anterior margin serrate; declivital surface glabrous, smooth, strongly shiny; small size; declivital face flattened; and interstriae 1 carinate, weakly inflated from apex to near midpoint of declivity; declivital striae 1–3 approximately equidistant.

##### Similar species.

*Amasa concitata*, *A. gibbosa*, *A. lini*, *A. youlii*.

##### Description

**(female).** 2.5–2.8 mm long (mean = 2.65 mm; n = 2); 2.5–2.54× as long as wide. Body light red-brown. Head, legs, and antennae light brown. ***Head***: epistoma entire, transverse, with a row of hair-like setae. Frons weakly convex to upper level of eyes; median impression between eyes; surface shagreened, impunctate, alutaceous, asperate; asperities longitudinal, smaller, rounder, denser above epistoma, increasing in length and decreasing in width and density dorsally and laterally. Eyes deeply emarginate just above antennal insertion, upper part smaller than lower part. Submentum triangular, deeply impressed. Antennal scape regularly thick, as long as club. Pedicel as wide as scape, shorter than funicle. Funicle 4-segmented, segment 1 shorter than pedicel. Club approximately circular and flat, type 4; segment 1 corneous, convex on anterior face, occupying approximately basal 1/5; segment 2 broad, larger than segment 1, corneous; segments 1–3 present on posterior face. ***Pronotum***: 1.13× as long as wide. In dorsal view basic, type 2, sides parallel in basal 1/2, rounded anteriorly; anterior margin with a row of 6–8 serrations. In lateral view basic, type 0, disc flat, summit at midpoint. Anterior slope shagreened with densely spaced, fine asperities, becoming lower and more strongly transverse towards summit; bearing long, fine, semi-recumbent hair-like setae. Disc shiny, alutaceous, impunctate, glabrous. Lateral margins obliquely costate. Base transverse, posterior angles narrowly rounded. ***Elytra***: 1.4× as long as wide, 1.23× as long as pronotum. Scutellum moderately sized, broad, linguiform, flush with elytra, flat, shiny. Elytral base transverse, edge oblique, humeral angles rounded, parallel-sided in basal 3/4, then sharply angulate to apex. Disc ascending posteriorly, shiny, glabrous; striae and interstriae laterally diverging from base to declivital summit; striae not impressed, punctures separated by 1–4 diameters of a puncture; interstriae flat, finely punctate, punctures 1/5 size of strial punctures, strongly confused. Declivity truncate, face flattened, strongly shiny, smooth, glabrous; three striae present, striae weakly impressed, equidistant, strial punctures strongly shiny, very large, deep, much larger and deeper than on disc, punctures subcontiguous to spaced by one diameter of a puncture; interstriae impunctate, convex, interstriae 1 weakly inflated from apex to below declivital midpoint, interstriae 1 uniseriate granulate, 2–4 multiseriate granulate, granules strongly confused; apical 1/4 of interstriae 1 and 2 costate with a row of rugae. Posterolateral margin forming a circumdeclivital carina; carina setose, setae short, erect, hair-like. ***Legs***: procoxae contiguous; prosternal coxal piece flat, inconspicuous. Protibiae slender, broadest at apical 1/3; posterior face inflated, finely granulate; apical 1/2 of outer margin with five small socketed denticles, their length as long as basal width. Meso- and metatibiae broad, flattened, outer margins evenly rounded with 11 and ten small socketed denticles, respectively; posterior faces unarmed; anterior faces finely granulate.

##### Etymology.

G. *tropis* = keel, ridge; *akron* = end. In reference to the inflated costate apex of the declivity. Noun in apposition.

##### Distribution.

Japan, Vietnam.

##### Host plants.

This species has been recorded from *Castanopsis* (Fagaceae).

**Figure 10.**
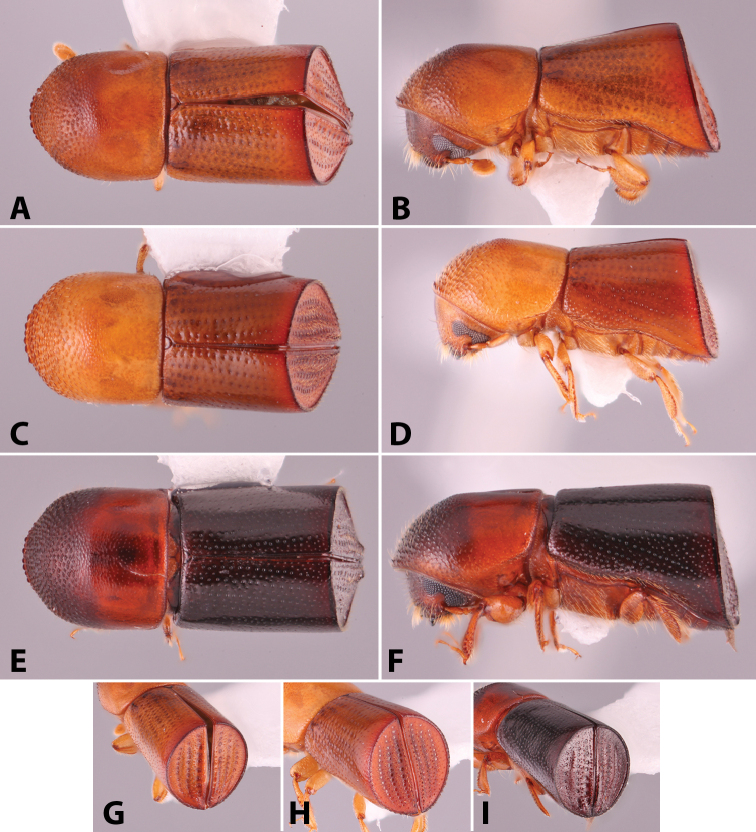
Dorsal, lateral and declivital view of *Amasa tropidacron* holotype, 2.5–2.8 mm (**A, B, G**), *A. versicolor*, 2.5–2.6 mm (**C, D, H**), and *A. youlii* holotype, 2.9–3.0 mm (**E, F, I**).

#### Amasa versicolor

Taxon classificationAnimaliaColeopteraCurculionidae

(Sampson, 1921)

Fig. 10C, D, H

Xyleborus versicolor Sampson, 1921: 29.
Amasa versicolor (Sampson): Wood and Bright 1992: 685.

##### Type material.

***Holotype*** (NHMUK), ***allotype*** (NHMUK).

##### New records.

Ceylon [Sri Lanka]: Kalutara Dist., Kanneliya, 250 m, 23.v.1973, S.L. Wood, ex limbs (NMNH, 1); Morapitiya, 250 m, 27.v.1973, S.L. Wood (NMNH, 2).

##### Diagnosis.

2.5–2.6 mm long (mean = 2.57 mm; n = 5); 2.27–2.43× as long as wide. The species is distinguished by the pronotum appearing basic (type 2) when viewed dorsally, anterior margin serrate; declivital surface shagreened, dull, opaque; declivital interstriae granulate, granules multiseriate, confused; declivity setose, interstriae moderately covered with semi-recumbent hair-like setae, approximately as long as the width of an interstria; and circumdeclivital carina margin setose, setae long, erect, bristle-like.

##### Similar species.

*Amasa cycloxyster*, *A. galeoderma*, *A. resecta*, *A. schlichii*, *A. youlii*.

##### Distribution.

Federated States of Micronesia, India (‘Bengal’), Indonesia (Java), East & West Malaysia, Myanmar, Sri Lanka*, Thailand.

##### Host plants.

Polyphagous (Browne 1961b; Beaver and Browne 1979).

#### Amasa youlii sp. nov.

Taxon classificationAnimaliaColeopteraCurculionidae

http://zoobank.org/5AAB68A6-B0EC-46B6-A0E8-D3A649A4B11C

Fig. 10E, F, I

##### Type material.

***Holotype***, female, China: Fujian, Fuzhou, Qishan, 31.iii.2018, Y. Li, ex 5 cm diameter twig, possibly Fagaceae (IZAS). ***Paratypes***, female, as holotype (MSUC, 1; NMNH, 1)

##### Diagnosis.

2.9–3.0 mm long (mean = 2.93 mm; n = 3); 2.42–2.5× as long as wide. This species is distinguished by the pronotum appearing basic (type 2) when viewed dorsally, anterior margin serrate; declivital surface smooth, moderately shiny; small size; declivital interstriae setose, setae recumbent; declivital face flattened; and interstriae 1 weakly inflated from apex to near midpoint of declivity; and declivital striae 2 medially displaced, not appearing equidistant between striae 1 and 3.

##### Similar species.

*Amasa concitata*, *A. gibbosa*, *A. lini*, *A. tropidacron*.

##### Description

**(female).** 2.9–3.0 mm long (mean = 2.93 mm; n = 3); 2.42–2.5 × as long as wide. Body bicolored: pronotal disc, head, legs, and antennae reddish, anterior slope of pronotum, elytra, and abdomen dark brown. ***Head***: epistoma entire, transverse, with a row of hair-like setae. Frons weakly convex to upper level of eyes; median impression between eyes; surface shagreened, impunctate, alutaceous, asperate; asperities longitudinal, smaller, rounder, denser above epistoma, increasing in size and length and decreasing in density dorsally and laterally. Eyes deeply emarginate just above antennal insertion, upper part smaller than lower part. Submentum triangular, deeply impressed. Antennal scape regularly thick, longer than club. Pedicel as wide as scape, shorter than funicle. Funicle 4-segmented, segment 1 shorter than pedicel. Club approximately circular and flat, type 4; segment 1 corneous, sinuate on anterior face, occupying approximately basal 1/4; segment 2 narrow, larger than segment 1, corneous; segments 1–3 present on posterior face. ***Pronotum***: 0.88× as long as wide. In dorsal view basic and parallel-sided, type 2, sides parallel in basal 1/2, rounded anteriorly; anterior margin with a row of 4–6 serrations. In lateral view basic, type 0, disc flat, summit at midpoint. Anterior slope strongly shagreened with densely spaced, short fine asperities, becoming lower and more strongly transverse towards summit, bearing long, fine, semi-recumbent hair-like setae. Disc shiny, alutaceous, densely minutely punctate, glabrous. Lateral margins obliquely costate. Base transverse, posterior angles narrowly rounded. ***Elytra***: 1.45× as long as wide, 1.65× as long as pronotum. Scutellum moderately sized, broad, linguiform, flush with elytra, flat, shiny. Elytral base transverse, edge oblique, humeral angles rounded, parallel-sided in basal 3/4, then sharply angulate to apex. Disc flat, shiny, glabrous; striae and interstriae laterally diverging from base to declivital summit; striae not impressed, punctures separated by 1–4 diameters of a puncture; interstriae flat, finely punctate, punctures 1/2 size of strial punctures, strongly confused. Declivity truncate, face flattened, moderately shiny, smooth, setose; three striae present, striae weakly impressed, striae 2 medially displaced near striae 1, strial punctures shiny, moderately large, moderately deep, much larger than on disc, punctures subcontiguous to spaced by three diameters of a puncture; interstriae impunctate, convex, interstriae 1 moderately inflated from apex to above declivital midpoint, interstriae 1 uniseriate granulate, 2–4 multiseriate granulate, granules strongly confused; apical 1/2 of interstriae 1 carinate to just before apex, becoming flattened, apical 1/4 of interstriae 2 costate, nearly carinate, with a row of rugae. Posterolateral margin forming a circumdeclivital carina; carina setose, setae short, erect hair-like. ***Legs***: procoxae contiguous, prosternal coxal piece flat, inconspicuous. Protibiae slender, broadest at apical 1/3; posterior face inflated, coarsely granulate; apical 1/2 of outer margin with six small socketed denticles, their length as long as basal width. Meso- and metatibiae broad, flattened, outer margins evenly rounded with 11 and nine small socketed denticles, respectively, posterior faces unarmed; anterior faces finely granulate.

##### Etymology.

Named after the collector Dr. You Li for his generous contributions to this project. Noun in genitive, invariable.

##### Distribution.

China (Fujian).

##### Host plants.

Unknown but potentially collected from Fagaceae.

### *Ambrosiodmus* Hopkins, 1915

#### Ambrosiodmus

Taxon classificationAnimaliaColeopteraCurculionidae

Hopkins, 1915

Ambrosiodmus Hopkins, 1915a: 55.
Phloeotrogus Motschulsky, 1863: 512. Wood 1969: 113.
Brownia Nunberg, 1963: 37. Synonymy: Wood 1980: 96.

##### Type species.

*Xyleborus tachygraphus* Zimmerman, 1868; original designation.

##### Diagnosis.

2.5–4.8 mm, 1.7–2.8× as long as wide, body usually stout and darkly colored. *Ambrosiodmus* is distinguished by the pronotum short and rounded, types 1 or 2 in dorsal view; pronotal disc entirely asperate; pronotum anterior margin without a carina or serrations; elytral disc convex; declivity rounded and steep at apex; antennal club flattened, type 4; scutellum flat, flush with elytra; mycangial tufts absent; and procoxae contiguous.

##### Similar genera.

*Ambrosiophilus*, *Beaverium*, *Immanus*.

##### Distribution.

Temperate and tropical regions of the world.

##### Gallery system.

This consists of a radial entrance tunnel leading to branched tunnels. These usually lie predominantly in one horizontal plane but may extend into three dimensions. They lack enlarged brood chambers. Many gallery systems are often started in a small area of the tree. Unlike many xyleborines, the galleries of different individuals often interconnect so that beetles can move between galleries (Beeson 1961; Kasson et al. 2016).

##### Remarks.

Recent studies suggest that all *Ambrosiodmus* and *Ambrosiophilus* species (see below) are associated with a single species of polypore basidiomycete ambrosia fungus (*Flavodon ambrosius*) (Kasson et al. 2016; Li et al. 2017). This fungus has greater ability to break down lignocellulose than most ambrosia fungi. This enables the beetles to colonize wood at a more advanced state of decay than most ambrosia beetles, and to persist in the same tree over several generations (Kasson et al. 2016; Li et al. 2017).

#### Key to *Ambrosiodmus* species (females only)

**Table:** 

1	Declivity granulate (Fig. 12F)	**2**
–	Declivity tuberculate or denticulate, never granulate (Fig. 12E)	**4**
2	Declivital interstriae with uniformly sized and spaced granules from base to apex; declivital interstriae bearing erect hair-like setae	*** rubricollis ***
–	Declivity with uniformly sized and spaced granules on declivital interstriae from base to declivity midpoint, apical 1/2 of interstriae with granules irregularly spaced; declivital interstriae slightly elevated and bearing erect thick setae	**3**
3	Larger, 3.2–3.4 mm; apical 1/2 of declivital interstriae 1 with five or six granules	*** brunneipes ***
–	Smaller, 2.9–3.1 mm; apical 1/2 of declivital interstriae 1 with three or four granules	*** conspectus ***
4	Declivital interstriae tuberculate, except interstriae 1 unarmed (rarely a few granules in some individuals); smaller, 2.5–2.8 mm	*** asperatus ***
–	All declivital interstriae tuberculate; larger, 3.4–4.8 mm	**5**
5	Tubercles of declivital interstriae 2 distinctly larger than those of other interstriae (Fig. 11L); usually larger, 3.4–4.8 mm	*** lewisi ***
–	Tubercles of declivital interstriae 2 similarly sized to those of other interstriae (Fig. 12E); usually smaller, 3.5–4.0 mm	*** minor ***

#### Ambrosiodmus asperatus

Taxon classificationAnimaliaColeopteraCurculionidae

(Blandford, 1895)

Fig. 11A, B, I

Xyleborus asperatus Blandford, 1895: 321.
Ambrosiodmus asperatus (Blandford): Wood 1989: 169.
Xyleborus nepotulus Eggers, 1923: 179. Synonymy: Schedl 1958c: 151.
Xyleborus citri Beeson, 1930: 215. Synonymy: Wood 1989: 169.
Xyleborus nepotulomorphus Eggers, 1936b: 88. Synonymy: Schedl 1958c: 151.

##### Type material.

***Holotype****Xyleborus asperatus* (NHMUK). ***Paratype****Xyleborus nepotulomorphus* (MFNB).

##### New records.

China: Guangxi, Shiwandashan, 25.iii.2018, Y. Li, ex *Quercus griffithii* (UFFE, 1). Hong Kong, Tai Po Kau, vi.2017, J. Skelton (MSUC, 1). Japan: South-western Japan, Okinawa, Iriomote-jima Island, H. Kajimura, ex *Machilus thunbergii* tree (MSUC, 1). Vietnam: Thua Thien-Hue, Bach Ma N.P., 16.22897, 107.85349, 415 m, 15.ii.2017, VN57, A.I. Cognato, T.A. Hoang, ex 5 cm diameter branch; twig (MSUC, 1).

##### Diagnosis.

2.5–2.8 mm long (mean = 2.64 mm; n = 5); 2.4–2.8× as long as wide. This species is distinguished by declivital interstriae 2 bearing a row of 3–5 denticles that are larger than those on other interstriae, and declivital interstriae 1 distinctly impressed.

##### Similar species.

*Ambrosiophilus cristatulus*, *A. osumiensis*, *A. subnepotulus*.

##### Distribution.

Australia, Brunei, China (Guizhou, Guangxi*, Hainan, Hong Kong*, Xizang), India (Tamil Nadu, West Bengal), Indonesia (Java, Sulawesi, Sumatra), Japan (Ryukyu Is), West Malaysia, Nepal, New Guinea, Sri Lanka, Taiwan, Thailand, Vietnam*.

##### Host plants.

Polyphagous (Beaver and Liu 2010).

##### Remarks.

This species has a very similar appearance and size to several *Ambrosiophilus* species which also have three or four denticles on declivital interstriae 2. The two genera are easily separated by the pronotal disc sculpturing: punctate in *Ambrosiophilus* and asperate in *Ambrosiodmus*.

**Figure 11.**
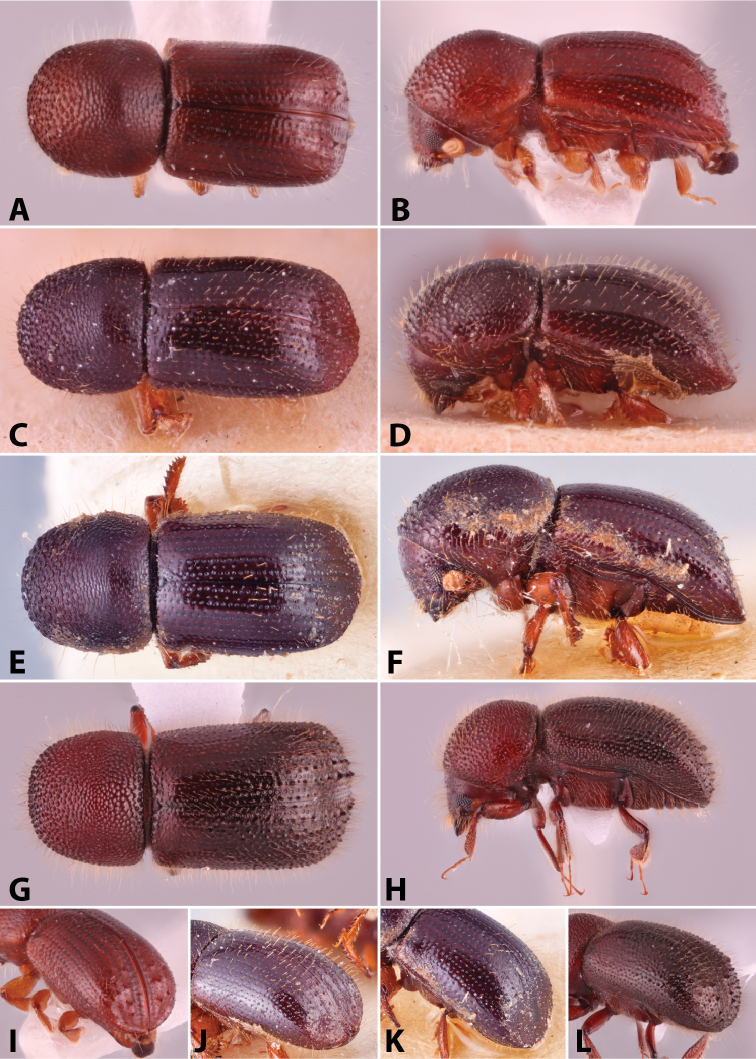
Dorsal, lateral and declivital view of *Ambrosiodmus asperatus*, 2.5–2.8 mm (**A, B, I**), *A. brunneipes*, 3.2–3.4 mm (**C, D, J**), *A. conspectus* paratype, 2.9–3.1 mm (**E, F, K**), and *A. lewisi*, 3.4–4.8 mm (**G, H, L**).

#### Ambrosiodmus brunneipes

Taxon classificationAnimaliaColeopteraCurculionidae

(Eggers, 1940)

Fig. 11C, D, J

Xyleborus brunneipes Eggers, 1940: 138.
Ambrosiodmus brunneipes (Eggers): Wood and Bright 1992: 671.

##### Type material.

***Allotype*** (NHMW).

##### Diagnosis.

3.2–3.4 mm long (mean = 3.38 mm; n = 5); 2.43–2.5× as long as wide. This species is distinguished by the declivital interstriae with uniformly sized and spaced granules from base to declivital midpoint, apical 1/2 of interstriae with granules irregularly spaced; declivital interstriae slightly elevated and bearing thick, erect setae, setae located ventrad of granules; declivital surface strongly shagreened; and dark brown color.

This species is very closely related to *A. conspectus* and is distinguished by the larger size and five or six granules on the apical 1/2 of declivital interstriae 1.

##### Similar species.

*Ambrosiodmus conspectus*, *A. rubricollis*.

##### Distribution.

Indonesia (Java), East & West Malaysia, Thailand.

##### Host plants.

Recorded from *Parartocarpus* (Moraceae), *Octomeles* (Tetramelaceae), and rattans (Arecacae). Probably polyphagous (Beaver et al. 2014).

#### Ambrosiodmus conspectus

Taxon classificationAnimaliaColeopteraCurculionidae

(Schedl, 1964)

Fig. 11E, F, K

Xyleborus conspectus Schedl, 1964b: 247.
Ambrosiodmus conspectus (Schedl): Wood and Bright 1992: 672.

##### Type material.

***Paratypes*** (NHMW, 2).

##### Diagnosis.

2.9–3.1 mm long (mean = 3.01 mm; n = 5); 2.48–2.73× as long as wide. This species is distinguished by declivity with uniformly sized and spaced granules on declivital interstriae from base to declivity midpoint, apical 1/2 of interstriae with granules irregularly spaced; declivital interstriae slightly elevated and bearing thick, erect setae, setae located ventrad of granules; declivital surface strongly shagreened; and dark brown color.

This species is very closely related to *A. brunneipes* and is distinguished by the smaller size and the and three or four granules on the apical 1/2 of declivital interstriae 1.

##### Similar species.

*Ambrosiodmus brunneipes*, *A. rubricollis*.

##### Distribution.

East Malaysia, Thailand.

##### Host plants.

Recorded only from rattan (Arecacae) (Schedl 1964b).

#### Ambrosiodmus lewisi

Taxon classificationAnimaliaColeopteraCurculionidae

(Blandford, 1894)

Fig. 11G, H, L

Xyleborus lewisi Blandford, 1894b: 104.
Ambrosiodmus lewisi (Blandford): Wood 1989: 170.
Ozopemon tuberculatus Strohmeyer, 1912: 38. Synonymy: Beaver and Liu 2010: 20.
Xyleborus lewekianus Eggers, 1923: 181. Synonymy: Wood 1989: 170.
Xyleborus tegalensis Eggers, 1923: 181. Synonymy: Schedl 1962a: 208.

##### Type material.

***Syntypes****Xyleborus lewisi* (NHMUK). ***Syntypes****Ozopemon tuberculatus* (SDEI).

##### New records.

China: Hong Kong, Sheung Shui, 22.vi.1964, ex soaked in oil (BPBM, 1); Tai Po Kau, 23.ix.1965, Lee Kit Ming, Hui Wai Ming, ex light trap (BPBM, 1), as previous except: 30.vi.1964, (BPBM, 1), as previous except: 2–6.vii.1964 (BPBM, 1), as previous except: 3–4.vii.1965 (BPBM, 1). India: Arunachal Pradesh, Hunli vicinity, 28°19'32"N, 95°57'31"E, 1300±100 m, 26.v.2012, L. Dembický (ZFMK, 1). Vietnam: Cao Bang, Phia Oac Hotel, 22°37.702'N, 105°54.5467'E, 847 m, 10–17.iv.2014, VN1, Cognato, Smith, Pham, ex in flight (MSUC, 1). Lao Cai, pass 8 km NW Sapa, 22°21'13"N, 103°46'01"E, 2030 m, 10.viii.2013, forested margin, V. Assing (MFNB, 1); Hoang Lien N.P., 22.35, 103.77, 1500–2000 m, 19.v.2019, VN168, S.M. Smith, A.I. Cognato, ex 10 cm branch (MSUC, 7). Ninh Binh, Cuc Phuong N.P., Mac Lake, 20°15'29.0"N, 105°42'27.5"E, 155 m, 4–7.v.2009, J.B. Heppner, ex blacklight trap (FSCA, 1). Thua Thien-Hue, Bach Ma N.P., 16.18902, 107.8498, 1193 m, 15.ii.2017, VN54, A.I. Cognato, T.A. Hoang, ex 1–4 cm diameter branch (MSUC, 1).

##### Diagnosis.

3.4–4.8 mm long (mean = 4.26 mm; n = 5); 1.7–2.53× as long as wide. This species is distinguished by each declivital interstriae variously tuberculate, never granulate; and red-brown color.

This species strongly resembles *A. minor* from which it can usually be distinguished by the larger size and the tubercles on declivital interstriae 2 distinctly larger than those of other interstriae.

##### Similar species.

*Ambrosiodmus minor*.

##### Distribution.

China (Guangdong, Guizhou, Guangxi, Hainan, Hong Kong*, Sichuan, Xizang, Yunnan), India (Arunachal Pradesh*, Assam, Tamil Nadu, West Bengal), Indonesia (Java, Kalimantan, Sumatra), Japan, East & West Malaysia, Myanmar, Philippines, South Korea, Sri Lanka, Taiwan, Thailand, Vietnam. Established in USA (Hoebeke 1991; Gomez et al. 2018a).

##### Host plants.

The species is polyphagous but may show some preference for Dipterocarpaceae in the southern part of its range, and for Fagaceae in the northern part (Browne 1961b).

#### Ambrosiodmus minor

Taxon classificationAnimaliaColeopteraCurculionidae

(Stebbing, 1907)

Fig. 12A, B, E

Phloeosinus minor Stebbing, 1907: 37.
Dryocoetes minor (Stebbing): Stebbing 1914: 549.
Xyleborus minor (Stebbing): Beeson 1930: 70.
Ambrosiodmus minor (Stebbing): Wood and Bright 1992: 676.
Xyleborus crassus Hagedorn, 1910a: 8. Synonymy: Schedl 1962a: 208.

##### Type material.

***Holotype****Phloeosinus minor* (FRI).

##### New records.

China: Chongqing, NanShan, 15.viii.2015, J-G Wang, Lv-Jia, Tian-Shang (RABC, 3). Jiangsu, Nanjing, Zijinshan, 10.viii.2017, Y. Li, ex unknown log (MSUC, 1). Jiangxi, Jinggang Shan Mts, Jingzhushan Zhufeng, forested slopes of river valley, 26°32.0'N, 114°08.6'E, 805 m, 29.iv.2011, M. Ficáček, J. Hájek (MNHP, 1). Zhejiang, Tianmu Shan, pass 25 km NW Linan, 620–820 m, 30°25'40"N, 119°35'30"E, creek valley with bamboo and mixed forest, litter, sifted, 16.vi.2007, M. Schülke (MFNB, 1). Laos: Louangnantha, Nantha to Muang Sing, 21°09'N, 101°19'E, 900–1200 m, 5–31.v.1997, V. Kubáň (NHMB, 3). NE, Hua Phan, Ban Saluei, Phou Pan (Mt.), 20°12'N, 104°01'E, 1300–1900 m, 7.iv–25.v.2010, C. Holzschuh (NHMUK, 2); NW, 5 km SW Muang Sing, Chiang Tung (Stupa) GH, 750 m, 26.iii–5.iv.2010, S. Murzin (IRSNB, 4); N, 10 km N Luang Prabang, Mekon [*sic*] riv., 240 km N. Vientiane, hill county [*sic*], sparse, settled primary vegetation, ix.1992, I. Somay (RABC, 1). Taiwan: [Pingtung Co.], Henchun, Kuraru [Kenting Forestry Park], 250 m, 3.iv.1965, C.M. Yoshimoto (BPBM, 1). Vietnam: Hoa Binh, 1929, A. DeCooman (MNHN, 1); as previous except: 1934 (MNHN, 1). Lao Cai, Nam Tha, 22.01218, 104.37685, 9.v.2015, Pham Thu, ex funnel trap (RJRC, 1).

##### Diagnosis.

3.5–4.0 mm long (mean = 3.74 mm; n = 5); 2.19–2.53× as long as wide. This species is distinguished by each declivital interstriae variously tuberculate, never granulate; and red-brown color.

This species strongly resembles *A. lewisi* from which it can usually be distinguished by the smaller size and tubercles on interstriae 2 not distinctly larger than those of other interstriae.

##### Similar species.

*Ambrosiodmus lewisi*.

##### Distribution.

Bangladesh, Bhutan, China (Chongqing, Guangxi, Jiangsu, Jiangxi*, Sichuan, Yunnan, Zhejiang), India (Assam, Meghalaya, Madhya Pradesh, Maharashtra, Uttarakhand, Uttar Pradesh, West Bengal), Laos*, East Malaysia, Myanmar, Nepal, Taiwan, Thailand, Vietnam. Established in the USA (Rabaglia and Okins 2011; Gomez et al. 2018a).

##### Host plants.

Polyphagous (Beeson 1930, 1961; Ohno 1990; Maiti and Saha 2004; Lin et al. 2019).

##### Remarks.

Wood and Bright (1992) considered the species as being described by Stebbing in 1909 (Stebbing 1909: 20) rather than in 1907 despite listing this publication under the taxonomy section for the species. This error undoubtedly occurred because Stebbing classified *Phloeosinus minor* as a new species in both publications. However, in 1909 he states “this amplifies the description given of this insect in [1907]” at the end of the species description. This error has been unknowingly perpetuated throughout the literature published since 1992.

**Figure 12.**
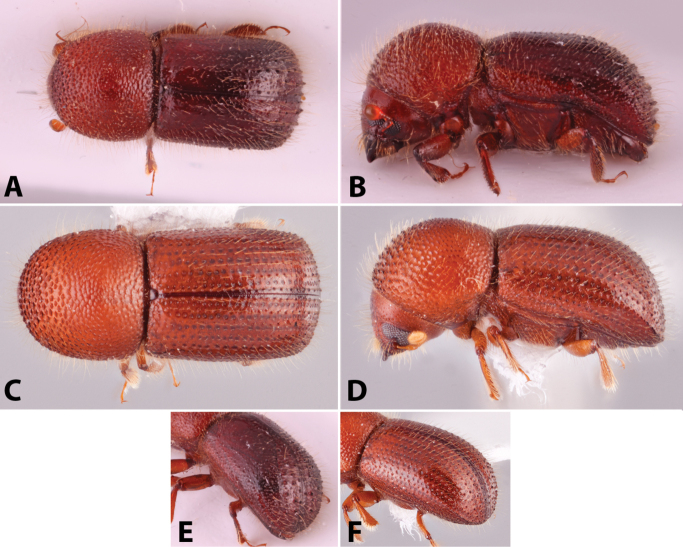
Dorsal, lateral and declivital view of *Ambrosiodmus minor*, 3.5–4.0 mm (**A, B, E**), and *A. rubricollis*, 2.5–2.8 mm (**C, D, F**).

#### Ambrosiodmus rubricollis

Taxon classificationAnimaliaColeopteraCurculionidae

(Eichhoff, 1876)

Fig. 12C, D, F

Xyleborus rubricollis Eichhoff, 1876a: 202.
Ambrosiodmus rubricollis (Eichhoff): Wood 1989: 170.
Xyleborus taboensis Schedl, 1952b: 65. Synonymy: Wood 1989: 170.
Xyleborus strohmeyeri Schedl, 1975b: 457. Synonymy: Wood 1989: 170.

##### Type material.

***Holotype*** (IRSNB) . Not examined.

##### New records.

China: Chongqing, Simian mtn, 7.v.2015, Tian-Shang, Lv-Jia (RABC, 1); as previous except: Jinfo mtn, 10.v.2015 (RABC, 2). Guangdong, Lantau Is., Shi Bi pool, hardwood plantation, 4.vi.2004, Li, Z-R. (RABC, 1). Guangxi, Jiangidi, 25°55.6'N, 110°14.8'E, 365 m, terraced fields surrounded with shrubs and bamboo forest, 12.iv.2013, M. Ficáček, J. Hájek, J. Růžička (MNHP, 1). Hong Kong Is., Shek O, secondary broadleaf trees & bamboo forest, 24.viii.2004 (RABC, 1); as previous except: Tai Po Kau, vi.2017, J. Skelton (MSUC, 1). Jiangxi, Gan Zhou, 7.vii.2016, Lv-Jia & Lai-S-C, ex *Senna surattensis* (RABC, 1); as previous except: Jinggang Shan, Jingzhushan Zhufeng, 26°31.0'N, 114°05.9'E, 640 m, stream valley, 25.iv.2011, M. Ficáček, J. Hájek (MNHP, 1); as previous except: 26°32.0'N, 114°08.6'E, 805 m, forested slopes of river valley, 29.iv.2011 (RABC, 1). Laos: Vientiane, Ban Van Eue, 15.xii.1965, native collector (BPBM, 1). Vietnam: Cao Bang, 22°33.118'N, 105°52.537'E, 1048 m, 12–17.vi.2014, VN9, Cognato, Smith, Pham, FIT (MSUC, 5). Thua Thien-Hue, Bach Ma N.P., 16.18902, 107.8498, 1193 m, 15.ii.2017, VN54, A.I. Cognato, T.A. Hoang, ex 1–4 cm diameter branch (MSUC, 1). Tuyen Quang, Doi Can Tuyen Quang, 21.72740, 105.22742, 15.iv.2015, R.J. Rabaglia, funnel trap (RJRC, 1). Yen Bai, Tan Huong, 21.82410, 104.89651, 15.iv.2015, funnel trap (RJRC, 1).

##### Diagnosis.

2.5–2.8 mm long (mean = 2.7 mm; n = 7); 2.45–2.55× as long as wide. This species is distinguished by the declivital interstriae with uniformly sized and spaced granules from base to apex; declivital interstriae slightly elevated and bearing erect hair-like setae, setae located ventrad of each granule; declivital surface shiny, and light red-brown color.

##### Similar species.

*Ambrosiodmus brunneipes*, *A. conspectus*.

##### Distribution.

China (Anhui, Beijing, Chongqing*, Fujian, Guangdong*, Guangxi*, Guizhou, Hebei, Heilongjiang, Hong Kong*, Hunan, Jiangxi*, Shaanxi, Shandong, Sichuan, Xizang, Yunnan, Zhejiang), Japan, South & North Korea, Laos*, West Malaysia, Taiwan, Thailand, Vietnam. Introduced to Australia (Wood and Bright 1992), Italy (Faccoli et al. 2009), North America (Bright 1968), and South America (Wood 2007).

##### Host plants.

A polyphagous species (Faccoli et al. 2009), which usually attacks smaller stems (Browne 1961b).

### *Ambrosiophilus* Hulcr & Cognato, 2009

#### Ambrosiophilus

Taxon classificationAnimaliaColeopteraCurculionidae

Hulcr & Cognato, 2009

Ambrosiophilus Hulcr & Cognato, 2009: 21.

##### Type species.

*Xyleborus restrictus* Schedl, 1939b; original designation.

##### Diagnosis.

1.95–4.5 mm, stout to elongate (2.27–2.92× as long as wide) with elytral apex rounded and entire. *Ambrosiophilus* is distinguished by the pronotum anterior margin typically without a carina or serrations; pronotal disc punctate; declivity rounded and steep; antennal club flattened, types 3 or 4; scutellum flat, flush; mycangial tufts absent; protibiae obliquely triangular; and procoxae contiguous.

*Ambrosiophilus* most closely resembles *Ambrosiodmus* and is distinguished by the pronotal disc and lateral areas punctate, never asperate, and lateral profile of pronotal and elytral discs flat.

##### Similar genera.

*Ambrosiodmus*.

##### Distribution.

Found in temperate and tropical Asia, two species are established in the United States (Gomez et al. 2018a).

##### Gallery system.

Similar to *Ambrosiodmus* (see above).

##### Remarks.

*Ambrosiophilus atratus* and *A. subnepotulus* are believed to use the same basidiomycete as *Ambrosiodmus* (see above) (Kasson et al. 2016; Li et al. 2017). However, some species are mycocleptic (Hulcr and Cognato 2010b). The female starts its gallery close to galleries of other ambrosia beetles. The fungus established by the ‘host’ species grows in the galleries of *Ambrosiophilus* which consequently has no need to transport its own ambrosia fungus, and lacks mycangia (Hulcr and Cognato 2010b; Kasson et al. 2016).

#### Key to *Ambrosiophilus* species (females only)

**Table:** 

1	Interstriae 1 armed with at least minute granules, other interstriae variously granulate or tuberculate (Fig. 13J)	**2**
–	Interstriae 1 unarmed, lacking even minute granules, other interstriae variously granulate or tuberculate (Fig. 14G)	**7**
2	Declivital interstriae 1–3 each armed by one major tubercle surrounding declivital sulcus; anterior margin of pronotum apically produced with a row of six serrations	*** latisulcatus ***
–	Declivital interstriae granulate, never armed by major tubercles; pronotum rounded and lacking serrations	**3**
3	Declivital interstriae granulate only on upper 1/2 of declivity; declivital face flattened, opalescent (Fig. 14J)	**4**
–	Declivital interstriae granulate along the entire length; declivital face rounded, shiny (Fig. 13J)	**5**
4	Smaller, 1.95–2.05 mm, more elongate, 2.6–2.7× as long as wide	***lannaensis* sp. nov.**
–	Larger, 2.5–2.75 mm, less elongate, 2.5–2.6× as long as wide	*** satoi ***
5	Pronotum from lateral view long (type 8) with summit displaced towards anterior margin (Fig. 5); dark brown or black, sometimes with reddish declivity	*** atratus ***
–	Pronotum from lateral view basic (type 2) with median summit (Fig. 5); red brown anteriorly with darker brown declivity	**6**
6	Declivital striae 1 weakly impressed; declivital interstriae moderately and uniformly granulate, granules spaced by a distance of four diameters of a granule	***caliginestris* sp. nov.**
–	Declivity weakly to strongly sulcate between striae 1 and interstriae 3; interstriae densely and uniformly granulate, granules on interstriae 3 spaced by a distance of less than the diameter of a granule	*** sulcatus ***
7	Declivity strongly sulcate, lateral margins of sulcus rounded, armed with three large spines, one at the base of interstriae 2, one at the declivital midpoint of interstriae 3 and one on the apical 1/3 of interstriae 3	*** sexdentatus ***
–	Declivity never strongly sulcate or armed with spines as described above	**8**
8	Tubercles on declivital interstriae 3 distinctly larger than those on interstriae 2 (Fig. 13E)	**9**
–	Tubercles of declivital interstriae 3 as large as or smaller than those of interstriae 2 (Fig. 14G)	**11**
9	Tubercles on declivital interstriae 3 very large, distinctly larger than those of other interstriae; tubercles present on interstriae 2 at declivital summit and often on declivital face; declivital surface coarsely sculptured	*** consimilis ***
–	Tubercles on declivital interstriae 3 small, but somewhat larger than those of other interstriae; tubercles on interstriae 2 only present at declivital summit; declivital surface finely sculptured, smooth	**10**
10	Declivital interstriae 3 bearing three small denticles; pronotal discal punctures small, fine, moderately spaced by 1–3 diameters of a puncture; pronotal disc shagreened	*** cristatulus ***
–	Declivital interstriae 3 bearing two large tubercles; pronotal discal punctures minute, very fine, widely spaced by 2–6 diameters of a puncture, pronotal disc shiny	*** subnepotulus ***
11	Declivital interstriae 2 armed by a single tubercle at declivital summit, remainder of interstriae 2 unarmed (Fig. 14A)	***indicus* sp. nov.**
–	Declivital interstriae 2 variously armed along its length (Fig. 14G)	**12**
12	Pronotum from dorsal view conical and elongate (type 5) (Fig. 16C); smaller, 2.0–2.1 mm	***wantaneeae* sp. nov.**
–	Pronotum from dorsal view basic or subquadrate (types 2 or 3) (Fig. 14G); larger, 2.3–3.2 mm	**13**
13	Tubercles of interstriae 2 larger than those of interstriae 3	*** osumiensis ***
–	Tubercles of interstriae 2 and 3 equally sized	***papilliferus* sp. nov.**

#### Ambrosiophilus atratus

Taxon classificationAnimaliaColeopteraCurculionidae

(Eichhoff, 1876)

Fig. 13A, B, I

Xyleborus atratus Eichhoff, 1876a: 201.
Ambrosiophilus atratus (Eichhoff): Hulcr and Cognato 2009: 22.
Xyleborus collis Niisima, 1910: 12. Synonymy: Smith et al. 2018b: 392.

##### Type material.

The ***holotype*** of *Xyleborus atratus* was destroyed in the bombing of UHZM in World War II (Wood and Bright 1992). ***Syntypes*** of *Xyleborus collis* should be housed in NIAES but have not been located (Smith et al. 2018b).

##### New records.

China: Chongqing, Nanshan, 20.viii.2015, Wang, J-G., Lv-Jia, Tian-Shang (RABC, 4); as previous except: Simian mtn, 7.v.2016, Tian-Shang, Lv-Jia (RABC, 1). Fukien [Fujian], Shaowu, Tachuland, 10–14.iv.1943, T.C. Ma (BPBM, 1).

##### Diagnosis.

3.3–3.5 mm long (mean = 3.46 mm; n = 5); 2.75–2.92× as long as wide. This species is distinguished by all declivital interstriae granulate along the entire length; pronotum from lateral view long (type 8); declivital striae 1 and 2 moderately to strongly impressed; declivital interstriae moderately and uniformly granulate, granules on interstriae 3 spaced by a distance of 2–3 diameters of a granule; interstrial setae long, hair-like; and large size.

##### Similar species.

*Ambrosiophilus caliginestris*, *A. satoi*, *A. sulcatus*.

##### Distribution.

China (Chongqing*, Fujian, Shanxi), Japan, South & North Korea, Taiwan. Introduced to Europe and North America (Atkinson et al. 1990; Faccoli 2008; Gomez et al. 2018a).

##### Host plants.

Polyphagous (Faccoli 2008; Beaver and Liu 2010).

##### Remarks.

Kasson et al. (2016) have shown that the symbiotic association of the species with the fungus, *Flavodon ambrosius*, has allowed niche expansion with large, long-lived, interconnecting colonies, overlapping generations, and pre-dispersal oviposition by young females.

**Figure 13.**
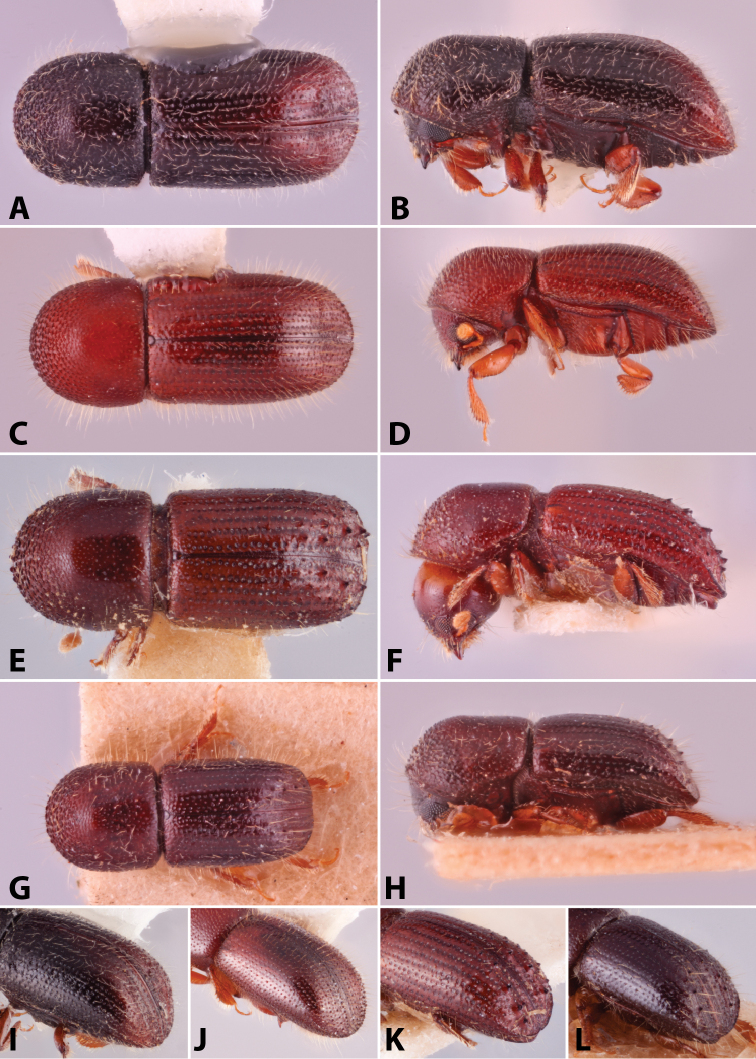
Dorsal, lateral and declivital view of *Ambrosiophilus atratus*, 3.3–3.5 mm (**A, B, I**), *A. caliginestris* holotype, 2.9 mm (**C, D, J**), *A. consimilis* holotype, 2.6–3.5 mm (**E, F, K**), and *A. cristatulus*, 2.1–2.3 mm (**G, H, L**).

#### Ambrosiophilus caliginestris sp. nov.

Taxon classificationAnimaliaColeopteraCurculionidae

http://zoobank.org/03B24F5A-E7B4-4AE8-AA98-FC688F1B0828

Fig. 13C, D, J

##### Type material.

***Holotype***, female, Vietnam: Cao Bang, 22°36.454'N, 105°52.083'E, 1661 m, 15.iv.2014, VN35, Cognato, Smith, Pham, ex phloem (MSUC).

##### Diagnosis.

2.9–3.1 mm long (mean = 3.0 mm; n = 2); 2.42–2.58× as long as wide. This species is distinguished by all declivital interstriae granulate along the entire length; pronotum from lateral view tall (type 2); declivital striae 1 weakly impressed; declivital interstriae moderately and uniformly granulate, granules spaced by a distance of four diameters of a granule; interstrial setae long, hair-like; and moderate size.

##### Similar species.

*Ambrosiophilus atratus*, *A. satoi*, *A. sulcatus*.

##### Description

**(female).** 2.9 mm long (n = 1); 2.42× as long as wide. Body ferruginous. Legs and antennae light brown. ***Head***: epistoma entire, transverse, with a row of hair-like setae. Frons weakly convex to upper level of eyes; surface shagreened, punctate; punctures dense, becoming shallower and sparser on reticulate upper part of frons. Eyes feebly emarginate just above antennal insertion, upper part smaller than lower part. Submentum narrow, triangular, slightly impressed. Antennal scape regularly thick, longer than club. Pedicel as wide as scape, much shorter than funicle. Funicle 4-segmented, segment 1 shorter than pedicel. Club approximately circular and flat, type 3; segment 1 corneous, transverse on anterior face, occupying approximately basal 1/2; segment 2 narrow, corneous; segments 1 and 2 present on posterior face. ***Pronotum***: 0.9× as long as wide. In dorsal view basic and parallel-sided, type 2, sides parallel in basal 1/2, rounded anteriorly; anterior margin without serrations. In lateral view tall, type 2, disc flat, summit pronounced. Anterior slope with densely spaced small asperities, becoming lower and more strongly transverse towards summit. Disc shagreened with sparse, fine punctures bearing long, fine, erect hair-like setae, some longer hair-like setae at margins. Lateral margins obliquely costate. Base transverse, posterior angles broadly rounded. ***Elytra***: 1.54× as long as wide, 1.6× as long as pronotum. Scutellum moderately sized, linguiform, flush with elytra, flat, shiny. Elytral base transverse, edge oblique, humeral angles rounded, parallel-sided in basal 3/4, then broadly rounded to apex. Disc shiny, striae not impressed, punctures moderately coarse, shallow, separated by less than one diameter of a puncture, glabrous; interstriae flat, finely punctate, punctures more widely separated than those of striae, with long, fine, erect hair-like setae. Declivity steep, strongly convex, shiny; strial punctures larger than on disc, striae 1 weakly impressed; interstriae moderately and uniformly granulate, granules spaced by a distance of four diameters of a granule, each granule with a moderately long, erect hair-like seta. Posterolateral margin carinate, granulate to interstriae 7. ***Legs***: procoxae contiguous; prosternal coxal piece tall, pointed. Protibiae slender, obliquely triangular, broadest at apical 1/3; posterior face smooth; apical 1/2 of outer margin with eight large socketed denticles, their length longer than basal width. Meso- and metatibiae flattened; outer margins evenly rounded with eight large socketed denticles.

##### Etymology.

L. *caligo* = fog; -*estris* = belonging to. In reference to the climate of the type localities. An adjective.

##### Distribution.

Vietnam.

##### Host plants.

Unknown.

#### Ambrosiophilus consimilis

Taxon classificationAnimaliaColeopteraCurculionidae

(Eggers, 1923) comb. nov.

Fig. 13E, F, K

Xyleborus consimilis Eggers, 1923: 180.
Ambrosiodmus consimilis (Eggers): Wood and Bright 1992: 672.

##### Type material.

***Holotype*** (MCG).

##### New records.

India: Bengal [West Bengal], Samsing, xi.1933, B. Singh, ex *Litsea* sp. (NMNH, 3).

##### Diagnosis.

2.6–3.5 mm long (mean = 3.06 mm; n = 5); 2.36–2.91× as long as wide. This species is distinguished by declivital interstriae 1 unarmed, interstriae 2 armed by one tubercle at declivital summit, remainder of interstriae 2 unarmed or with a few granules, interstriae 3 with two or three large denticles; declivity weakly bisulcate from sutural margin to striae 2, interstriae 3 moderately and distinctly convex; pronotal disc surface shiny, punctures small, fine, widely spaced by 2–4 diameters of a puncture; and declivital surface coarsely sculptured, shiny; and large size.

##### Similar species.

*Ambrosiophilus cristatulus*, *A. indicus*, *A. osumiensis*, *A. subnepotulus*.

##### Distribution.

India (Tamil Nadu, West Bengal*), East Malaysia.

##### Host plants.

This species has only been recorded from *Litsea* (Lauraceae).

##### Remarks.

The species has the generic characters of *Ambrosiophilus* and is here transferred to that genus.

#### Ambrosiophilus cristatulus

Taxon classificationAnimaliaColeopteraCurculionidae

(Schedl, 1953)

Fig. 13G, H, L

Xyleborus cristatulus Schedl, 1953b: 300.
Ambrosiodmus cristatulus (Schedl): Wood and Bright 1992: 672.
Ambrosiophilus cristatulus (Schedl): Beaver et al. 2014: 24.

##### Type material.

***Lectotype*** (NHMW). Not examined.

##### Diagnosis.

2.1–2.3 mm long (mean = 2.21 mm; n = 4); 2.3–2.39× as long as wide. This species is distinguished by declivital interstriae 1 unarmed, interstriae 2 armed by one tubercle at declivital summit, remainder of interstriae 2 unarmed, interstriae 3 with three small denticles; declivity weakly bisulcate from sutural margin to striae 2, interstriae 3 weakly convex; pronotal surface shagreened, discal punctures small, fine, moderately spaced by 1–3 diameters of a puncture; declivital surface smooth, shiny; and small size.

##### Similar species.

*Ambrosiophilus consimilis*, *A. indicus*, *A. subnepotulus*.

##### Distribution.

China (Fujian), East & West Malaysia, Thailand.

##### Host plants.

Unknown.

#### Ambrosiophilus indicus sp. nov.

Taxon classificationAnimaliaColeopteraCurculionidae

http://zoobank.org/1D40CC15-8862-41A6-8630-F5E9E2567C60

Fig. 14A, B, I

##### Type material.

***Holotype***, female, India: Bengal [West Bengal], Kalimpong, Samsingh, 25.x.1933, N.C. Chatterjee, ex “kanda lahara” (NMNH). ***Paratypes***, female, as holotype (NMNH, 2). All specimens are individually point mounted to a single pin. The top specimen is the holotype and the bottom two are paratypes.

##### Diagnosis.

2.4 mm long (mean = 2.4 mm; n = 3); 2.67× as long as wide. This species is distinguished by declivital interstriae 1 unarmed, interstriae 2 armed by one tubercle at declivital summit, remainder of interstriae 2 unarmed, interstriae 3 with three minute tubercles equally spaced from base to apex; declivity weakly bisulcate from sutural margin to striae 2, interstriae 3 feebly convex; pronotal surface shagreened, discal punctures minute, very fine, widely spaced by four diameters of a puncture; declivital surface shagreened; and small size.

##### Similar species.

*Ambrosiophilus consimilis*, *A. cristatulus*, *A. subnepotulus*.

##### Description

**(female).** 2.4 mm long (mean = 2.4 mm; n = 3); 2.67× as long as wide. Body ferruginous, antennae and legs light brown. ***Head***: epistoma entire, transverse, with a row of hair-like setae. Frons weakly convex to upper level of eyes; surface subshiny, punctate; punctures moderately dense, becoming shallower and sparser on reticulate upper part. Eyes shallowly emarginate just above antennal insertion, upper part smaller than lower part. Submentum narrow, triangular, slightly impressed. Antennal scape regularly thick, longer than club. Pedicel as wide as scape, as long as funicle. Funicle 4-segmented, segment 1 shorter than pedicel. Club approximately circular and flat, type 4; segment 1 transverse on anterior face, occupying approximately basal 1/6; segment 2 narrow, larger than segment 1, corneous; segments 1–3 present on posterior face. ***Pronotum***: 1.1× as long as wide. In dorsal view basic, type 2, sides parallel in basal 1/2, rounded anteriorly; anterior margin without serrations. In lateral view basic, type 0, disc flat, summit pronounced. Anterior slope with closely spaced, coarse asperities, becoming lower and more strongly transverse towards summit. Disc shagreened with sparse, small, fine punctures bearing short, fine, erect hair-like setae, some longer hair-like setae at margins. Lateral margins obliquely costate. Base transverse, posterior angles broadly rounded. ***Elytra***: 1.3× as long as wide, 1.58× as long as pronotum. Scutellum moderately sized, linguiform, flush with elytra, flat, shiny. Elytral base transverse, edge oblique, humeral angles rounded, parallel-sided in basal 3/4, then broadly rounded to apex. Disc subshiny, striae not impressed, with moderately coarse, shallow punctures separated by 1–2 diameters of a puncture, glabrous; interstriae flat, finely punctate, punctures more widely separated than those of striae, with long, fine, erect hair-like setae. Declivity steep, strongly convex, shagreened; strial punctures larger than on disc, weakly bisulcate from sutural margin to striae 2; interstriae 1 unarmed, interstriae 2 armed by one tubercle at declivital summit, remainder of interstriae 2 unarmed, interstriae 3 with three minute equally spaced tubercles from base to apex; interstriae 3 feebly convex. Posterolateral margin carinate to interstriae 7. ***Legs***: procoxae contiguous. Protibiae obliquely triangular, broadest at apical 1/3; posterior face smooth; outer margin of apical 1/2 with six moderate socketed denticles, approximately as long as basal width. Meso- and metatibiae flattened; outer margins evenly rounded with seven large socketed denticles.

##### Etymology.

L. *indicus* = of India. An adjective.

##### Distribution.

India (West Bengal).

##### Host plants.

Unknown.

**Figure 14.**
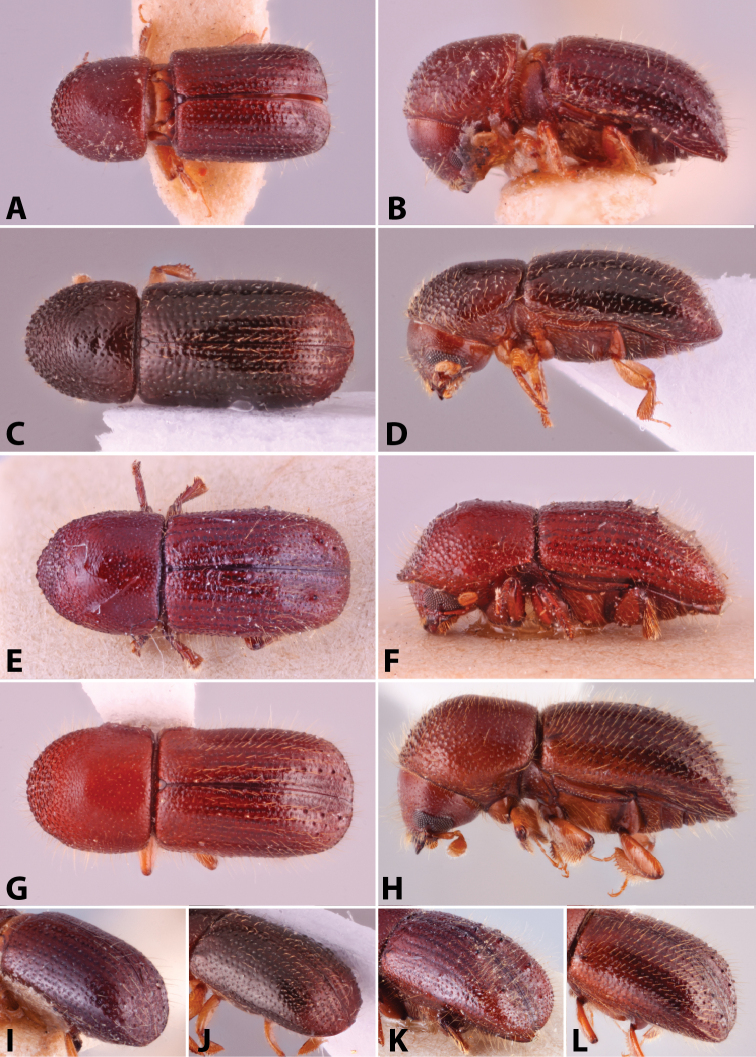
Dorsal, lateral and declivital view of *Ambrosiophilus indicus* holotype, 2.4 mm (**A, B, I**), *A. lannaensis* holotype holotype, 1.95–2.05 mm (**C, D, J**), *A. latisulcatus*, 3.9–4.2 mm (**E, F, K**), and *A. osumiensis*, 2.3–3.2 mm (**G, H, L**).

#### Ambrosiophilus lannaensis sp. nov.

Taxon classificationAnimaliaColeopteraCurculionidae

http://zoobank.org/A9A545CB-1BEC-4B49-93AA-9A7208B0A24A

Fig. 14C, D, J

##### Type material.

***Holotype***, female, Thailand: Chiang Mai, Doi Pui, 18°50'23"N, 98°53'53"E, 1200–1300 m, 2-BM-Jun-B-23 (2016), [vi.2016], S. Sanguansub et al., ex *Butea monosperma* (MSUC). ***Paratypes***, female, as holotype except: 3-BM-Jun-B-26 (NHMUK, 1); as previous except: 2-CAJun-B-14, ex *Castanopsis armata* (QSBG, 1); as previous except: 4-CA-Jun-B-114 (RABC, 1); as previous except: 4-CA-Jun-B-8 (SSC, 1); as previous except: 4-CA-Jun-B-87 (SSC, 1); as previous except: 2-LT-Jun-B-91, ex *Lithocarpus tenuinervis* (SSC, 1); as previous except: Doi Pui, Chiang Khian Highl. Res. Stn, 27.vii.2013, S. Buranapanichpan, ex persimmon, *Diospyros kaki* (RABC, 1); as previous except: ex *Mangifera indica* (MSUC, 1); as previous except: Doi Pui, 1400 m, 25.v–2.vi.2006, W. Puranasakul, ex EtOH trap (RABC, 1).

##### Diagnosis.

1.95–2.05 mm long (mean = 2.01 mm; n = 5); 2.67–2.73× as long as wide. This species is distinguished by all declivital interstriae granulate on upper 1/2 of declivity; pronotum from dorsal view conical and elongate (type 5), from lateral view type 7; pronotal disc shiny, punctures moderately fine and separated by several times their diameter; posterolateral margins of elytra rounded; lower part of declivity flattened; and declivital striae not impressed, interstriae finely, sparsely granulate on upper part of declivity only.

##### Similar species.

*Ambrosiophilus atratus*, *A. caliginestris*, *A. satoi*, *A. wantaneeae*.

##### Description

**(female).** 1.95–2.05 mm long (mean = 2.01 mm; n = 5); 2.67–2.73 × as long as wide. Body dark brown, antennae and legs light brown. ***Head***: epistoma entire, transverse, with a row of hair-like setae. Frons weakly convex to upper level of eyes; surface subshiny, punctate; punctures moderately dense, becoming shallower and sparser on reticulate upper part. Eyes deeply emarginate just above antennal insertion, upper part smaller than lower part. Submentum distinctly triangular, slightly impressed. Antennal scape regularly thick, approximately as long as club. Pedicel as wide as scape, shorter than funicle. Funicle 4-segmented, segment 1 shorter than pedicel. Club approximately circular and flat, type 3; segment 1 corneous, transverse on anterior face, occupying approximately basal 1/3; segment 2 narrow, corneous; segments 1 and 2 present on posterior face. ***Pronotum***: 1.13× as long as wide. In dorsal view conical and elongate, type 5, sides almost parallel in basal 1/2, conical anteriorly; anterior margin without serrations. In lateral view elongate, disc longer than anterior slope, type 7, summit not pronounced, on anterior 1/3. Anterior slope with widely spaced, small coarse asperities, becoming lower and more strongly transverse towards summit. Disc subshiny with moderately dense small, deep punctures bearing short, fine, erect hair-like setae, some longer hair-like setae at margins. Lateral margins obliquely costate. Base transverse, posterior angles broadly rounded. ***Elytra***: 1.63× as long as wide, 1.67× as long as pronotum. Scutellum small, triangular, flush with elytra, flat, shiny. Elytral base transverse, edge oblique, humeral angles rounded, parallel-sided in basal 3/4, then broadly rounded to apex. Disc shiny, striae not impressed, with moderately coarse, shallow punctures separated by one width of their diameter, each bearing a short, semi-erect hair-like seta; interstriae flat, finely punctate, punctures more widely separated than those of striae, with fine, semi-erect setae. Declivity steep, strongly convex, shagreened; strial punctures larger than on disc, striae 1 and 2 very weakly impressed; interstriae unarmed by granules, each puncture bearing a moderately long, erect hair-like seta. Posterolateral margin rounded, unarmed. ***Legs***: procoxae contiguous; prosternal coxal piece short, pointed. Protibiae slender, obliquely triangular, broadest at apical 1/3; posterior face smooth; apical 1/2 of outer margin with five large socketed denticles, their length longer than basal width. Meso- and metatibiae flattened; outer margins evenly rounded with seven large socketed denticles.

##### Etymology.

The specific name refers to the old Northern Thai kingdom ‘Lan Na’. Latinized adjective.

##### Distribution.

Thailand.

##### Host plants.

This species is evidently polyphagous and is here reported from *Mangifera indica* (Anacardiaceae), *Diospyros kaki* (Ebenaceae), *Butea monosperma* (Fabaceae), *Castanopsis armata*, and *Lithocarpus tenuinervis* (Fagaceae).

#### Ambrosiophilus latisulcatus

Taxon classificationAnimaliaColeopteraCurculionidae

(Eggers, 1940)

Fig. 14E, F, K

Xyleborus latisulcatus Eggers, 1940: 142.
Ambrosiodmus latisulcatus (Eggers): Wood and Bright 1992: 675.
Ambrosiophilus latisulcatus (Eggers): Beaver et al. 2014: 25.

##### Type material.

***Holotype****Xyleborus latisulcatus* (NMNH).

##### Diagnosis.

3.9–4.2 mm long (mean = 4.05 mm; n = 2); 2.52–2.8× as long as wide. This species is distinguished by declivital interstriae 1–3 each armed by one major tubercle surrounding declivital sulcus; pronotum from dorsal view conical frontally (type 6); pronotal anterior slope steep, flat; anterior margin with a row of six serrations; pronotum from lateral view tall (type 2); pronotal surface reticulate, discal punctures coarse, dense, spaced less than the diameter of a puncture; declivity moderately sulcate to interstriae 3, margins of sulcus armed with three equally sized tubercles: one at the base of interstriae 1, one on interstriae 2 just ventrad to the first, and one at the midpoint of interstriae 3.

##### Similar species.

*Ambrosiophilus sexdentatus*, *A. sulcatus*.

##### Distribution.

Indonesia (Java), Thailand.

##### Host plants.

Unknown.

#### Ambrosiophilus osumiensis

Taxon classificationAnimaliaColeopteraCurculionidae

(Murayama, 1934)

Fig. 14G, H, L

Xyleborus osumiensis Murayama, 1934: 292.
Ambrosiophilus osumiensis (Murayama): Smith et al. 2018b: 393.
Xyleborus metanepotulus Eggers, 1939b: 119. Synonymy: Smith et al. 2018b: 393.
Xyleborus nodulosus Eggers, 1941b: 233. syn. nov.
Xyleborus pernodulus Schedl, 1957: 85. Unnecessary replacement name. Synonymy: Browne 1961c: 50.
Xyleborus hunanensis Browne, 1983b: 33. Synonymy: Beaver 2011: 283.
Ambrosiophilus peregrinus Smith & Cognato, 2015: 216. Synonymy: Smith et al. 2017: 552.

##### Type material.

***Holotype****Xyleborus hunanensis* (IZAS). ***Holotype****Xyleborus metanepotulus* (TARI). ***Holotype****Xyleborus nodulosus* (ZMFK). ***Holotype****Xyleborus osumiensis* (NMNH). ***Holotype****Ambrosiophilus peregrinus* (NMNH), ***paratypes*** (MSUC, 5).

##### New records.

China: Anhui, Chuxian, 32.25N, 118.28E, 1.v.1965, *Pistacia chinensis* (NMNH, 4). Chongqing, Nan Shan, 20.viii.2015, Wang, J-G., Lv-Jia, Tian-Shang (RABC, 4); as previous except: Simian mtn, 7.v.2016, Tian-Shang, Lv-Jia (RABC, 2); as previous except: Youyang, 5.vi.2016, Tian-Shang (RABC, 1). Guangxi, Malu, 27.iii.2018, Y. Li, ex *Cinnamomum cassia* (UFFE, 1); as previous except: Shangsi, 25.iii.2018, ex *Broussonetia papyrifera* (UFFE, 1); as previous except: unknown host (UFFE, 1); as previous except: Shiwandashan, 25.iii.2018, Y. Li, ex *Quercus griffithii* (UFFE, 1); as previous except: Shangsi, 26.iii.2018, Y. Li, ex *Quercus griffithii* (UFFE, 1). Jiangxi, Ganzhou, Lv-Jia, ex *Ligustrum lucidum* (RABC, 1). Sichuan, Emei mtn, 18.viii.2016, Tian-Shang (RABC, 1). Yunnan, Xishuangbanna, Sanchahe Nat. Res., 22°09.784'N, 100°52.256'E, 2186 m, 29–30.v.2008, A.I. Cognato (MSUC, 1). Vietnam: Cao Bang, 22°34.118'N, 105°52.537'E, 1048 m, 12–17.iv.2014, VN9, Cognato, Smith, Pham, FIT (MSUC, 1). Ninh Binh, Cuc Phuong N.P., 7.iii.2018, 20.34932, 105.59669, 431 m, A.I. Cognato, S.M. Smith, VN 130, ex standing dead laurel (MSUC, 2). Thua Thien-Hue, Bach Ma N.P., 16.18902, 107.8498, 1193 m, 15.ii.2017, VN54, A.I. Cognato, T.A. Hoang, ex 1–4 cm diameter branch (MSUC, 1).

##### Diagnosis.

2.3–3.2 mm long (mean = 2.6 mm; n = 7); 2.3–2.67× as long as wide. This species is distinguished by declivital interstriae 1 unarmed, 2 armed by 3–5 pointed tubercles along its length, major declivital tubercles on interstriae 2; weakly to moderately sulcate to striae 1, interstriae 2 convex, bearing 3–5 pointed tubercles and several small granules (near apical and basal margins) along its length; pronotum from dorsal view basic or subquadrate (type 2 or 3); and pronotum from lateral view basic (type 0).

##### Similar species.

*Ambrosiophilus papilliferus*, *A. subnepotulus*, *A. wantaneeae*.

##### Distribution.

China (Anhui, Chongqing*, Fujian, Guangxi*, Guizhou, Hunan, Jiangxi*, Sichuan*, Yunnan), Japan, Taiwan, Vietnam. Imported and established in USA (Smith and Cognato 2015; Schiefer 2018).

##### Host plants.

This species is likely polyphagous and has been recorded from numerous host families including *Pistacia* (Anacardiaceae), *Ilex* (Aquifoliaceae), *Quercus* (Fagaceae), *Cinnamomum* (Lauraceae), *Broussonetia* (Moraceace), and *Ligustrum* (Oleaceae).

##### Remarks.

The morphology of *A. osumiensis* is highly variable in regard to numerous characteristics that are routinely used to diagnose other xyleborine species. Such variation includes: the antennal club type either 3 or 4; pronotum basic (type 2) or subquadrate (type 3) from dorsal view; declivity shiny or shagreened; pronotal disc shiny or shagreened; number and size of tubercles on declivital interstriae 2; and a large size range with individuals differing by up to 0.9 mm in length. This variation led to *A. osumiensis* being described several times. Types of each species are distinct and diagnosable. Examination of the specimens listed above in ‘new records’ as well as the holotypes showed that these species formed a continuous spectrum of variation. During our fieldwork we were able to collect and sequence specimens that fell within the concept of *X. metanepotulus* (Vietnam), *X. hunanensis* (China), *X. nodulosus* (China) and *A. peregrinus* (Georgia, USA) and an additional larger morphospecies from multiple localities in Vietnam. COI sequences showed that all populations differed by no more than 7.4% supporting the hypothesis of one morphologically variable species. Typical intraspecific variation in xyleborines is below 10% (Cognato et al. 2020b). *Xyleborus hunanensis*, *X. metanepotulus*, *X. nodulosus* and *A. peregrinus* are thus all conspecific and considered synonyms of the oldest name, *A. osumiensis*.

The identification of *A. nodulosus* from East Malaysia by Browne (1980b) and Ohno (1990) appears to be incorrect. We have therefore omitted East Malaysia from the distribution, and also omitted the associated host records. The host records reported in Smith et al. (2017) are therefore incorrect.

#### Ambrosiophilus papilliferus sp. nov.

Taxon classificationAnimaliaColeopteraCurculionidae

http://zoobank.org/B552010D-0595-470E-8A24-01C0C81F7964

Fig. 15A, B, I

##### Type material.

***Holotype***, female, 贵州 平塘 核桃 1981.VI.6 采集者：罗禄怡 [China: Guizhou, Pingtang, 6.vi.1981, Luyi Luo, ex *Carya* sp.] (NMNH). ***Paratype***, female, Vietnam: Thua Thien-Hue, Bach Ma N.P., 16.18902, 107.8498, 1193 m, 15.ii.2017, VN54, A.I. Cognato, T.A. Hoang, ex 1–4 cm diameter branch (MSUC).

##### Diagnosis.

2.5 mm long (n = 1); 2.5× as long as wide. This species is distinguished by declivital interstriae 1 unarmed, interstriae 2 armed by four or five moderately sized and variably spaced denticles along its length, interstriae 3 armed by five larger denticles; declivital striae 1 and 2 moderately impressed; and pronotum from dorsal view basic (type 2), lateral view basic (type 0).

##### Similar species.

*Ambrosiophilus osumiensis*, *A. wantaneeae*.

##### Description

**(female).** 2.5 mm long (n = 1); 2.5× as long as wide. Body color red-brown, antennae and legs light brown. ***Head***: epistoma entire, transverse, with a row of hair-like setae. Frons weakly convex to upper level of eyes; surface subshiny, punctate; punctures moderately dense, becoming shallower and sparser on reticulate upper part. Eyes deeply emarginate just above antennal insertion, upper part smaller than lower part. Submentum narrow, triangular, slightly impressed. Antennal scape regularly thick, shorter than club. Pedicel as wide as scape, shorter than funicle. Funicle 4-segmented, segment 1 shorter than pedicel. Club tall and oval, flat, type 3; segment 1 convex and small on anterior face, occupying approximately basal 1/6; segment 2 corneous, narrow; segments 1–3 present on posterior face. ***Pronotum***: 0.79× as long as wide. In dorsal view basic, type 2, sides parallel in basal 1/2, rounded anteriorly; anterior margin without serrations. In lateral view basic, type 0, disc flat, summit pronounced. Anterior slope with widely spaced, small coarse asperities, becoming lower and more strongly transverse towards summit. Disc subshiny with moderately dense small, shallow punctures bearing short, fine, erect hair-like setae, some longer hair-like setae at margins. Lateral margins obliquely costate. Base transverse, posterior angles broadly rounded. ***Elytra***: 1.64× as long as wide, 2.1× as long as pronotum. Scutellum moderately sized, linguiform, flush with elytra, flat, shiny. Elytral base transverse, edge oblique, humeral angles rounded, parallel-sided in basal 3/4, then broadly rounded to apex. parallel-sided in basal 3/4, then broadly rounded to apex. Disc opalescent, striae weakly impressed, with moderately coarse, shallow, and irregular punctures separated by 0.5–1 diameter of a puncture, glabrous; interstriae flat, finely punctate, punctures more widely separated than those of striae, with fine, erect hair-like setae. Declivity steep, strongly convex, shagreened; strial punctures larger than on disc, striae 1 and 2 moderately impressed, strial punctures bearing short, recumbent setae 1× width of a puncture; interstriae 1 unarmed by granules, interstriae 2 with four or five coarse granules, interstriae 3 and 4 with four or five slightly smaller granules, each granule with a moderately long, erect hair. Posterolateral margin carinate to interstriae 7. ***Legs***: procoxae contiguous, prosternal coxal piece short, pointed. Protibiae slender, obliquely triangular, broadest at apical 1/3; posterior face smooth; apical 1/2 of outer margin with six large socketed denticles, their length longer than basal width. Meso- and metatibiae flattened, outer margins evenly rounded with eight large socketed denticles.

##### Etymology.

L. *papilla* = nipple; adjectival suffix *ferus* = bearer. In reference to the denticles on the declivity. An adjective.

##### Distribution.

China (Guizhou), Vietnam.

##### Host plants.

Recorded only from *Carya* (Juglandaceae).

##### Remarks.

Locality labels on the holotype are in Chinese and were translated by You Li. An English locality label has been placed on the specimen below the original locality labels.

**Figure 15.**
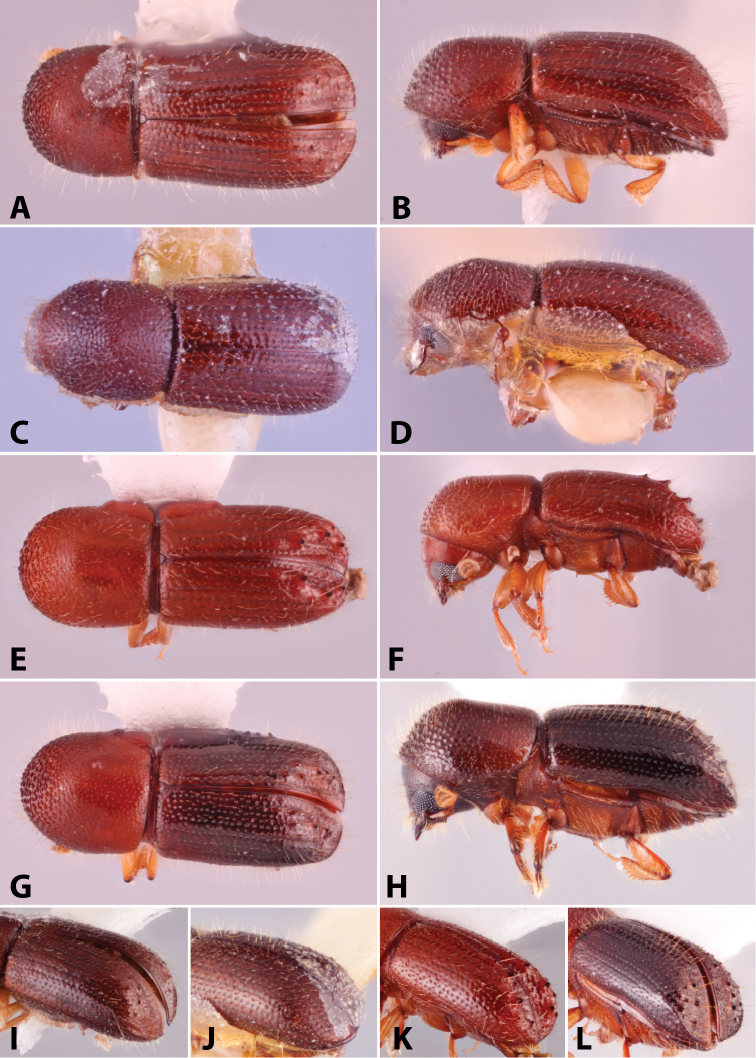
Dorsal, lateral and declivital view of *Ambrosiophilus papilliferus* holotype, 2.5 mm (**A, B, I**), *A. satoi* paratype, 2.5–2.75 mm (**C, D, J**), *A. sexdentatus*, 2.7–3.0 mm (**E, F, K**), and *A. subnepotulus*, 2.5–2.8 mm (**G, H, L**).

#### Ambrosiophilus satoi

Taxon classificationAnimaliaColeopteraCurculionidae

(Schedl, 1966)

Fig. 15C, D, J

Xyleborus satoi Schedl, 1966b: 39.
Ambrosiophilus satoi (Schedl): Beaver and Liu 2010: 22.

##### Type material.

***Paratype*** (NHMW).

##### New records.

Thailand: Chiang Mai, Doi Suthep, 1400 m, EtOH trap, 16–20.v.2005, W. Puranasakul (RABC, 1); as previous except: 20.vii.2016, S. Sanguansub et al. (RABC, 1); as previous except: 18°50'23"N, 98°53'53"E, 1200–1300 m, vi.2016, S. Sanguansub et al., ex *Castanopsis armata* (RABC, 1); as previous except: ex *Lithocarpus tenuinervis* (RABC, 1).

##### Diagnosis.

2.5–2.75 mm long (mean = 2.67 mm; n = 5); 2.5–2.57× as long as wide. This species is distinguished by all declivital interstriae granulate on upper 1/2 of declivity; pronotum from lateral view basic (type 0); declivity rounded, face flattened; declivital interstriae sparsely and uniformly granulate, granules spaced by a distance of at least four diameters of a granule; interstrial setae short, bristle-like; and small size.

##### Similar species.

*Ambrosiophilus atratus*, *A. caliginestris*, *A. latisulcatus*, *A. sulcatus*.

##### Distribution.

Bhutan, Taiwan, Thailand*.

##### Host plants.

Recorded from a ‘camphor log’ (probably *Cinnamomum camphora* (Lauraceae)) (Schedl 1966b), and from *Castanopsis armata* and *Lithocarpus tenuinervis* (Fagaceae).

#### Ambrosiophilus sexdentatus

Taxon classificationAnimaliaColeopteraCurculionidae

(Eggers, 1940)

Fig. 15E, F, K

Xyleborus sexdentatus Eggers, 1940: 148.
Ambrosiodmus sexdentatus (Eggers): Wood and Bright 1992: 680.
Ambrosiophilus sexdentatus (Eggers): Hulcr and Cognato 2009: 24.

##### Type material.

***Holotype*** (NMNH).

##### Diagnosis.

2.7–3.0 mm long (mean = 2.84 mm; n = 5); 2.7–2.9× as long as wide. This species is distinguished by declivital interstriae 2 (1 spine), interstriae 3 (2 spines) surrounding declivital sulcus; pronotum from dorsal view basic (type 2); pronotal anterior slope rounded, convex; pronotum anterior margin lacking serrations; pronotum from lateral view tall (type 2); pronotal discal punctures small, fine spaced by at least two diameters of a puncture, surface shiny; declivity strongly sulcate to interstriae 3, lateral margins of sulcus rounded, margin armed with three large spines, one at the base of interstriae 2, one at the declivital midpoint of interstriae 3 and one on the apical 1/3 of interstriae 3.

##### Similar species.

*Ambrosiophilus latisulcatus*, *A. sulcatus*.

##### Distribution.

Indonesia (Java), New Guinea, Thailand.

##### Host plants.

Recorded from *Quercus* (Fagaceae) and *Tectona* (Lamiaceae) in Java (Kalshoven 1959b).

##### Remarks.

A mycocleptic associate of *Beaverium* species (Hulcr and Cognato 2010b, 2013).

#### Ambrosiophilus subnepotulus

Taxon classificationAnimaliaColeopteraCurculionidae

(Eggers, 1930)

Fig. 15G, H, L

Xyleborus subnepotulus Eggers, 1930: 178.
Ambrosiodmus subnepotulus (Eggers): Wood and Bright 1992: 680.
Ambrosiophilus subnepotulus (Eggers): Beaver and Liu 2010: 22.
Xyleborus cristatuloides Schedl, 1971a: 284. syn. nov.

##### Type material.

***Holotype****Xyleborus subnepotulus* (FRI). ***Lectotype****Xyleborus cristatuloides* (NHMW).

##### New records.

China: Guizhou, Guiyang, Huaxi, 31.iv.2015, Y. Li, ex in flight (UFFE, 9). Hong Kong, vi.2017, J. Skelton (MSUC, 15). Laos: Vientiane, Ban Van Eue, 31.xii.1965, native collector (BPBM, 2).

##### Diagnosis.

2.5–2.8 mm long (mean = 2.64 mm; n = 7); 2.27–2.6× as long as wide. This species is distinguished by declivital interstriae 1 unarmed, interstriae 2 armed by one tubercle at declivital summit, remainder of interstriae 2 unarmed, interstriae 3 with two large tubercles; declivity weakly bisulcate from sutural margin to striae 2; interstriae 3 weakly convex; pronotal surface shiny, discal punctures minute, very fine, widely spaced by 2–6 diameters of a puncture; and declivital surface smooth, shiny; and moderate size.

##### Similar species.

*Ambrosiophilus consimilis*, *A. cristatulus*, *A. indicus*, *A. osumiensis*.

##### Distribution.

China* (Guizhou, Hong Kong*), Indonesia (Java), Laos*, Myanmar, Sri Lanka, Taiwan.

##### Host plants.

The only recorded host is *Albizia lebbeck* (Fabaceae) (Beeson 1930).

##### Remarks.

Wood (1989) considered *X. cristatuloides* Schedl as a synonym of *Ambrosiodmus asperatus*. However, the lectotype has a punctate pronotal disc and declivital sculpturing that is almost identical with *Ambrosiophilus subnepotulus* and it is here placed in synonymy.

#### Ambrosiophilus sulcatus

Taxon classificationAnimaliaColeopteraCurculionidae

(Eggers, 1930)

Fig. 16A, B, E

Xyleborus sulcatus Eggers, 1930: 180.
Ambrosiodmus sulcatus (Eggers): Wood and Bright 1992: 680.
Cyclorhipidion sulcatum (Eggers): Maiti and Saha 2004: 118.
Ambrosiophilus sulcatus (Eggers): Beaver and Liu 2018: 537.
Xyleborus sulcatulus Eggers, 1939a: 13. syn. nov.
Xyleborus sinensis Eggers, 1941b: 224. syn. nov.

##### Type material.

***Holotype****Xyleborus sinensis* (ZMFK). ***Holotype****Xyleborus sulcatus* (FRI). ***Holotype****Xyleborus sulcatulus* (NHRS).

##### New records.

China: Jiangxi, Wu-Yi Mt., 19.vii.2017, Lai, S-C, Tian Shang et al. (RABC, 1). India: Bengal [West Bengal], Darjeeling, Debrepani, 6000 ft, 15.ix.1929, J.C.M. Gardner, unknown wood (NMNH, 1). Taiwan: [Formosa], Taiheizan, 9.v.[19]32, L. Gressitt (NMNH, 1). Chiayi Co., Fenkihu, 1370 m, 10–12.iv.1965, C.M. Yoshimoto, B.D. Perkins (BPBM, 1). Vietnam: Hoa Binh, 1940, A. DeCooman (MNHN, 1), Lao Cai, 16 km W of Sa Pa, 1800 m, at light, 17.iii.1998, L. Peregovits, T. Vásárhelyi (RABC, 1).

##### Diagnosis.

3.4–4.5 mm long (mean = 3.94 mm; n = 5); 2.5–2.87× as long as wide. This species is distinguished by all declivital interstriae granulate along the entire length; pronotum from dorsal view basic (type 2); pronotal anterior slope rounded; pronotal anterior margin without a row of serrations; pronotum from lateral view tall (type 2); declivity weakly to strongly bisulcate between striae 1 and interstriae 3; interstriae densely and uniformly granulate, granules on interstriae 3 spaced by a distance of less than the diameter of a granule; interstrial setae long, hair-like, and of large size.

*Ambrosiophilus sulcatus* is variable in body length, the degree of bisulcation of the declivity and in the size of the declivital granules, but all specimens form a continuous spectrum of variation. Specimens from India and China (Fujian) are larger, more strongly bisulcate and have slightly larger granules than specimens occurring further south (Myanmar and Vietnam).

##### Similar species.

*Ambrosiophilus atratus*, *A. caliginestris*, *A. latisulcatus*, *A. satoi*.

##### Distribution.

China (Fujian, Jiangxi*), India (Assam, West Bengal*), Myanmar, Nepal, Taiwan*, Vietnam*.

##### Host plants.

Recorded only from *Artocarpus* (Moraceae) (Beeson 1930).

##### Remarks.

The type specimens of *Xyleborus sinensis*, *X. sulcatulus* and type images of *X. sulcatus*, were directly examined. The specimens differ in size (2.8 mm *X. sulcatulus*, 3.0 mm, *X. sulcatus*, 4.2 mm *X. sinensis*), the depth of the declivital sulci and in the degree development of interstrial granules. Additional non-type specimens were also examined. We found that size, depth of the declivital sulci and development of interstrial granules formed a continuum of variation and should be considered a single morphologically variable species.

**Figure 16.**
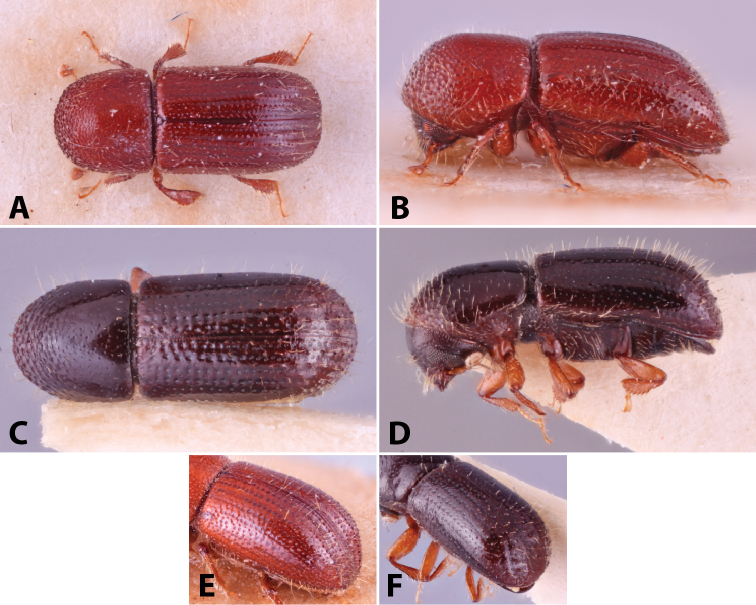
Dorsal, lateral and declivital view of *Ambrosiophilus sulcatus* holotype, 3.4–4.5 mm (**A, B, E**), and *A. wantaneeae* holotype, 2.0–2.1 mm (**C, D, F**).

#### Ambrosiophilus wantaneeae sp. nov.

Taxon classificationAnimaliaColeopteraCurculionidae

http://zoobank.org/1BF7E0E1-F0EC-4121-8CA5-02D5122BD6C1

Fig. 16C, D, F

##### Type material.

***Holotype***, female, Thailand: Chiang Mai, Doi Pui, 1400 m, 17.iv.–8.v.2006, W. Puranasakul, ex EtOH trap (NHMUK). ***Paratypes***, female, as holotype except: 25.iv.–16.v.2005 (QSBG, 1; RABC, 1); as previous except: flight intercept trap (MSUC, 1); as previous except: 18°50'23"N, 98°53'53"E, 1200–1300 m, 30.iv.2014, S. Sanguansub et al., ex EtOH trap (RABC, 1).

##### Diagnosis.

2.0–2.1 mm long (mean = 2.03 mm; n = 4); 2.63–2.77× as long as wide. This species is distinguished by declivital interstriae 1 unarmed, 2 armed by four or five coarse granules along its length, interstriae 3 with four or five slightly smaller granules; declivital striae 1 and 2 very weakly impressed; and pronotum from dorsal view conical (type 0) to subelongate (type 7), lateral view long (type 7).

##### Similar species.

*Ambrosiophilus osumiensis*, *A. papilliferus*.

##### Description

**(female).** 2.0–2.1 mm (mean = 2.02 mm; n = 4); 2.63–2.67× as long as wide. Body dark brown to pitchy black, antennae and legs light brown. ***Head***: epistoma entire, transverse, with a row of hair-like setae. Frons weakly convex to upper level of eyes; surface subshiny, punctate; punctures moderately dense, becoming shallower and sparser on reticulate upper part. Eyes deeply emarginate just above antennal insertion, upper part smaller than lower part. Submentum narrow, triangular, slightly impressed. Antennal scape regularly thick, approximately as long as club. Pedicel as wide as scape, shorter than funicle. Funicle 4-segmented, segment 1 shorter than pedicel. Club approximately circular, type 3; segment 1 corneous, transverse on anterior face, occupying approximately basal 1/3; segment 2 narrow, corneous; segments 1 and 2 present on posterior face. ***Pronotum***: 1.0–1.1× as long as wide. In dorsal view conical and elongate, type 5, sides almost parallel in basal 1/2, conical anteriorly; anterior margin without serrations. In lateral view elongate, disc as long as anterior slope, type 7, summit not pronounced, at midpoint. Anterior slope with widely spaced, small asperities, becoming lower and more strongly transverse towards summit. Disc strongly shiny with sparse, small, deep punctures bearing short, fine, erect hair-like setae. Some longer hair-like setae at margins. Lateral margins obliquely costate. Base transverse, posterior angles broadly rounded. ***Elytra***: 1.6–1.7× as long as wide, 1.6–1.7× as long as pronotum. Scutellum small, triangular, flush with elytra, flat, shiny. Elytral base transverse, edge oblique, humeral angles rounded, parallel-sided in basal 3/4, then broadly rounded to apex. Disc shiny, striae not impressed, parallel, with moderately coarse, shallow punctures separated by 1–2× their diameter, without hair-like setae; interstriae flat, finely punctate, punctures more widely separated than those of striae, with fine, erect hair-like setae. Declivity shiny, steep, strongly convex; strial punctures larger than on disc, striae 1 and 2 very weakly impressed; interstriae 1 without granules, interstriae 2 with four or five coarse granules, interstriae 3 and 4 with four or five slightly smaller granules, each granule with a moderately long, erect hair-like seta. Posterolateral margin rounded, unarmed. ***Legs***: procoxae contiguous; prosternal coxal piece short, pointed. Protibiae slender, broadest at apical 1/3; posterior face smooth; apical 1/2 of outer margin with six moderate socketed denticles, their length slightly longer than basal width. Meso- and metatibiae flattened; outer margins evenly rounded with eight small socketed denticles.

##### Etymology.

The species is named for Ms. Wantanee Puranasakul (then at Chiang Mai University, Thailand) who collected several new species of Scolytinae during her MSc studies. Noun in genitive.

##### Distribution.

Thailand.

##### Host plants.

Unknown.

### *Ancipitis* Hulcr & Cognato, 2013

#### Ancipitis

Taxon classificationAnimaliaColeopteraCurculionidae

Hulcr & Cognato, 2013

Ancipitis Hulcr & Cognato, 2013: 41.

##### Type species.

*Xyleborus puer* Eggers, 1923; original designation.

##### Diagnosis.

1.9–5.4 mm long, 2.08–2.73× as long as wide. *Ancipitis* is distinguished by the flat submentum that is flush with genae and shaped as a distinct large triangle; elytra extremely long, flattened, very gradually descending, broadened laterally and elongated apically; declivital face appearing somewhat depressed below posterolateral costa and covered with hair-like setae; pronotum extended anteriad, appearing conical, type 0 in dorsal view, without serrations on anterior margin; antennal club flattened, type 3 with three sutures visible on the posterior face; scape long and slender; protibiae slender, all tibia bearing large denticles; procoxae appearing tall, longer than basal width; scutellum flat, flush with elytra; procoxae narrowly separated; mycangial tufts absent; elytra unarmed.

##### Similar genera.

*Diuncus*, *Leptoxyleborus*.

##### Distribution.

Distributed in temperate and tropical Asia and Melanesia.

##### Gallery system.

This consists of branched tunnels without brood chambers (Browne 1961b). There may also be surface galleries between the bark and the sapwood (Kalshoven 1959b; Browne 1961b).

#### Key to *Ancipitis* species (females only)

**Table:** 

1	Declivity weakly sulcate between interstriae 3 in middle of declivity; sutural interstriae weakly raised and striae 1 impressed in apical third; larger, 4.9–5.4 mm	*** punctatissimus ***
–	Declivity not sulcate between interstriae 3 in middle of declivity; sutural interstriae not raised and striae 1 not impressed in apical third; smaller, 3.0–3.6 mm	*** puer ***

#### Ancipitis puer

Taxon classificationAnimaliaColeopteraCurculionidae

(Eggers, 1923)

Fig. 17A, B, E

Xyleborus puer Eggers, 1923: 191.
Ancipitis puer (Eggers): Hulcr and Cognato 2013: 42.
Xyleborus ceramensis Schedl, 1937a: 549. Synonymy: Hulcr and Cognato 2013: 42.

##### Type material.

***Holotype*** (MCG), ***syntypes*** (MCG, 2; NMNH, 2).

##### Diagnosis.

Moderately sized, 3.0–3.6 mm long (mean = 3.2 mm; n = 5); 2.14–2.73× as long as wide. This species is distinguished by its moderate size, declivity not sulcate between interstriae 3 in middle of declivity; sutural interstriae not raised and striae 1 not impressed in apical third; and declivital striae and interstriae both bearing long hair-like setae that are erect on the interstriae and semi-recumbent on the striae.

##### Similar species.

*Leptoxyleborus machili*, *L. sordicauda*.

##### Distribution.

Indonesia (Ceram, Sumatra), East & West Malaysia, New Guinea, Thailand.

##### Host plants.

Recorded only from *Shorea* (Dipterocarpaceae) and *Intsia* (Fabaceae) (Browne 1961b), but probably polyphagous.

**Figure 17.**
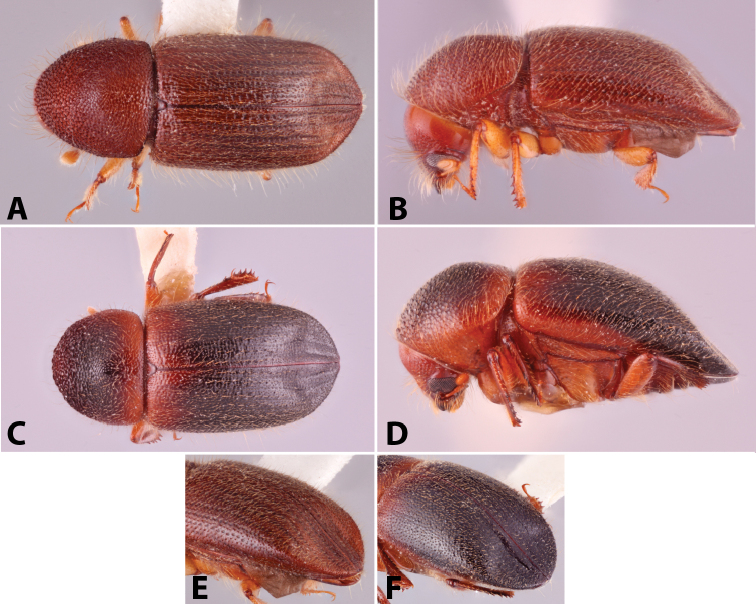
Dorsal, lateral and declivital view of *Ancipitis puer*, 3.0–3.6 mm (**A, B, E**), and *A. punctatissimus*, 4.9–5.4 mm (**C, D, F**).

#### Ancipitis punctatissimus

Taxon classificationAnimaliaColeopteraCurculionidae

(Eichhoff, 1880)

Fig. 17C, D, F

Xyleborus punctatissimus Eichhoff, 1880: 189.
Leptoxyleborus punctatissimus (Eichhoff): Wood and Bright 1992: 660.
Ancipitis punctatissimus (Eichhoff): Beaver et al. 2014: 26.
Xyleborus spatulatus Blandford, 1896b: 218. Synonymy: Kalshoven 1959a: 95.

##### Type material.

***Syntypes****Xyleborus spatulatus* (NHMUK).

##### Diagnosis.

The largest *Ancipitis* species, 4.9–5.4 mm long (mean = 5.16 mm; n = 5); 2.08–2.41× as long as wide. This species is distinguished by its large size; declivity weakly sulcate between interstriae 3 in middle of declivity; sutural interstriae weakly raised and striae 1 impressed in apical third; and declivital interstriae with three rows of mixed short erect and recumbent hair-like setae.

##### Similar species.

*Ancipitis puer*, *Leptoxyleborus sordicauda*.

##### Distribution.

Indonesia (Java, Sumatra), East & West Malaysia, Thailand.

##### Host plants.

Recorded from four different families of angiosperm trees, and from *Pinus merkusii* (Pinaceae) (Browne 1961b; Ohno 1990; Schedl 1969a).

### *Anisandrus* Ferrari, 1867

#### Anisandrus

Taxon classificationAnimaliaColeopteraCurculionidae

Ferrari, 1867

Anisandrus Ferrari, 1867: 24.

##### Type species.

*Apate dispar* Fabricius, 1793; monotypy.

##### Diagnosis.

2.1–5.9 mm, 1.88–2.78× as long as wide, body usually stout and dark. *Anisandrus* is distinguished most easily by the antennal club obliquely truncate type 1 (*A. achaete* type 2), club taller than wide (*A. achaete* wider than tall), procoxae narrowly separated, protibiae slender, obliquely or distinctly triangular, outer margin with 5–8 large socketed denticles on distal 1/2, posterior face unarmed, mesonotal mycangial tufts typically present along the pronotal base (missing in three species), either as a small tuft the length of the scutellum and directly opposite it or extending laterally from the scutellum to striae 3 and with elytral base broadly, shallowly emarginated from the scutellum to striae 3. Additional diagnostic characters include: pronotum from dorsal view typically types 0 and 1 (*A. cryphaloides*, type 6), pronotum from lateral view tall (type 3), or rounded and robust (type 5), pronotum anterior margin with a row of serrations, pronotum lateral margins obliquely costate, scutellum flat, flush with elytra, and the elytral disc either convex or variously transversely impressed with a saddle-like depression. Species range from nearly glabrous to densely setose and are typically black or dark brown.

##### Similar genera.

*Cnestus*, *Cyclorhipidion*, *Hadrodemius*, *Xylosandrus*. *Anisandrus* is closely related to *Cnestus*, *Hadrodemius* and *Xylosandrus*, all of which possess a mesonotal mycangium and the associated dense tuft of hair-like setae at the scutellar area and pronotal base (Gohli et al. 2017; Johnson et al. 2018).

##### Distribution.

Uncommon genus with species occurring in forests of the Holarctic and Paleotropical regions.

##### Gallery system.

The species usually attack stems of small diameter, and the gallery system consists of a radial or circumferential gallery with several longitudinal branches without brood chambers. SMS collected several species (*A. cristatus*, *A. lineatus*, *A. longidens*) in northern Vietnam that had a preference for attacking small saplings just above the soil line.

##### Remarks.

This genus is remarkably diverse in montane habitats across Asia but most species are poorly known. It is very likely that many additional new species await description.

#### Key to *Anisandrus* species (females only)

**Table:** 

1	Pronotal mycangial tuft moderate to densely setose, very broad, extending laterally from the scutellum to striae 3 (Fig. 23E)	**2**
–	Pronotal mycangial tuft absent (Fig. 23C) or just anteriad and roughly equal in width to scutellum, lightly to moderately setose (Fig. 22C)	**7**
2	Posterolateral margin of elytra rounded (Fig. 18L); declivital face convex or flattened; smaller, 2.8–3.1 mm	**3**
–	Posterolateral margin of elytra costate or carinate to interstriae 5 (Fig. 23K); declivital face variably sulcate; larger, 3.9–5.6 mm	**4**
3	Elytral disc flat; declivital face moderately steep and convex; declivital summit with interstriae 1 unarmed, a small denticle on interstriae 2 and a minute denticle on interstriae 3; declivity shiny	***auratipilus* sp. nov.**
–	Elytral disc with a broad and weak transverse saddle-like depression; declivital face steep, flattened; declivital summit with a minute denticle on interstriae 1, a small denticle on interstriae 2, and interstriae 3 unarmed; declivity opalescent	***venustus* sp. nov.**
4	At least punctures of declivital striae 2 strongly confused, minute; pronotal asperities large, widely spaced; elytral disc with a profound transverse saddle-like depression; declivity broadly sulcate to interstriae 5	*** percristatus ***
–	Declivital strial punctures all uniseriate, large; pronotal asperities small, densely spaced; elytral disc with a weak to deep transverse saddle-like depression; declivity sulcate to interstriae 3	**5**
5	Elytral disc with a weak transverse saddle-like depression (Fig. 21B); declivital interstriae uniseriately punctate, and setose, setae erect, very long, very fine and hair-like	***hera* sp. nov.**
–	Elytral disc with a deep transverse saddle-like depression (Fig. 21H); declivital interstriae impunctate or with biseriate punctures, and setae semi-erect, short, thick, or scale-like	**6**
6	Declivital interstriae impunctate, setose, setae semi-erect, short and thick; declivital summit with large incurved spine on interstriae 2; declivital interstriae 3 with six additional unequally sized incurved spines on basal 1/2 of declivity; larger, 5.4–5.6 mm	*** klapperichi ***
–	Declivital interstriae minutely biseriately punctate, setose, setae bristle-like, erect; declivital summit with a large incurved spine on interstriae 2, interstriae 3 unarmed; smaller, 4.0–4.15 mm	***xuannu* sp. nov.**
7	Mesonotal mycangial tuft absent (Fig. 23C)	**8**
–	Mesonotal mycangial tuft just anteriad and roughly equal in width to scutellum, lightly to moderately setose (Fig. 22C)	**10**
8	Antennal club wider than longer, type 2, one suture visible on posterior face (Fig. 2); protibiae distinctly triangular; anterior margin of the pronotum without serrations	*** achaete ***
–	Antennal club longer than wide, type 1, no sutures visible on posterior face (Fig. 2); elytral disc convex; protibiae obliquely triangular; anterior margin of the pronotum with a row of serrations	**9**
9	Declivital interstriae 1 and 3 armed by 4–5 unequally sized tubercles; declivital striae strongly impressed; elytral disc with a weak transverse saddle-like depression; pronotal disc coarsely punctate; larger, 4.5 mm	*** carinensis ***
–	Declivital interstriae uniseriate granulate on basal 1/2, granules equally sized; striae clearly impressed; elytral disc convex; pronotal disc finely punctate; smaller, 2.8 mm	***paragogus* sp. nov.**
10	Interstriae 2 and 3 of equal width at midpoint of declivity (Fig. 18K)	**17**
–	Interstriae 2 and 3 not equal in width at midpoint of declivity (Fig. 22J)	**11**
11	Interstriae 2 narrower than interstriae 3 at midpoint of declivity (Fig. 22J)	**12**
–	Interstriae 3 narrower than interstriae 2 at midpoint of declivity (Fig. 20J)	**15**
12	Declivity rounded, posterolateral margin rounded	**13**
–	Declivity obliquely truncate, posterolateral margin costate	**14**
13	Elytral disc with a weak transverse saddle-like depression; declivital interstriae 2 armed with a blunt tubercle at summit, interstriae 3 armed by one or two denticles near declivital summit ventrad to tubercle on interstriae 2	***sinivali* sp. nov.**
–	Elytral disc convex; declivity unarmed	*** hirtus ***
14	Declivity weakly bisulcate, margins ornamented by large sharp spines on interstriae 2–7, spine on interstriae 3 the largest; declivital interstriae impunctate; posterolateral margin costate to interstriae 5.	*** longidens ***
–	Declivity steeply rounded and flat, declivital summit armed by a minute denticle on interstriae 2 and 3; granules present on basal 1/2 of interstriae 2–4; declivital interstriae clearly punctate, posterolateral margin costate to interstriae 7	*** improbus ***
15	Declivity steeply rounded and flat; elytral apex sharply angulate, nearly subquadrate; posterolateral margin costate to interstriae 5; pronotum rounded, type 1, in dorsal view	*** eggersi ***
–	Declivity gradual and convex, elytral apex broadly rounded; posterolateral margin rounded; pronotum conical, type 0, in dorsal view	**16**
16	Declivity strongly shagreened or opalescent; striae weakly impressed; smaller, 2.1–2.4 mm	***cryphaloides* sp. nov.**
–	Declivity strongly shiny, striae deeply impressed; larger, 2.6–3.3 mm	*** lineatus ***
17	Declivital interstriae 2 punctate, punctures either uniseriate or confused	**18**
–	Declivital interstriae 2 impunctate, punctures may be replaced by granules	**22**
18	Declivital interstriae 2 punctures multiseriate and confused; body densely covered by erect dark brown pubescence	*** ursulus ***
–	Declivital interstriae 2 punctures uniseriate; body nearly glabrous or at most moderately setose	**19**
19	Declivity rounded and convex; posterolateral margin rounded	***auco* sp. nov.**
–	Declivity steep and face variably impressed; posterolateral margin costate or carinate	**20**
20	Declivital summit unarmed; declivital face flat and weakly depressed below lateral margins	*** mussooriensis ***
–	Declivital summit ornamented by two small sharp incurved spines at the base of interstriae 2 and 3; declivital face flat and moderately bisulcate or concave	**21**
21	Declivity moderately bisulcate; declivital interstriae bearing erect fine hair-like setae	***feronia* sp. nov.**
–	Declivital face concave; declivital interstriae bearing erect pointed bristle-like setae	*** geminatus ***
22	Posterolateral margins of elytra rounded; larger, 5.8–5.9 mm	*** niger ***
–	Posterolateral margins of elytra costate or carinate; smaller, 2.2–3.7 mm	**23**
23	Declivital summit without a sharp hooked spine on interstriae 2; declivital interstriae 2 face densely granulate or denticulate; elytral disc typically without a weak transverse saddle-like depression	**24**
–	Declivital summit with a sharp hooked spine on interstriae 2; declivital interstriae 2 face sparsely granulate; elytral disc flat, with a weak transverse saddle-like depression (rarely flat in some *apicalis* and *cristatus*)	**25**
24	Declivital interstriae denticulate; elytral discal interstriae punctures uniseriate; declivity appearing bisulcate with declivity impressed from striae 1 to interstriae 2, interstriae 3 distinctly raised; smaller, 2.2–2.5 mm	*** maiche ***
–	Declivital interstriae granulate; elytral discal interstriae with 2–3 confused rows of punctures; declivital interstriae 1 slightly raised, interstriae 2 and 3 flush; larger, 3.1–3.5 mm	*** dispar ***
25	Spine at declivital summit of interstriae 2 backwardly pointed; smaller, 2.6–2.8 mm	***congruens* sp. nov.**
–	Spine at declivital summit of interstriae 2 incurved; larger, 3.05–3.7 mm	**26**
26	Spines interstriae 3 not backwardly hooked, much smaller than spine at the summit of interstriae 2; smaller, 3.05–3.4 mm; declivity weakly sulcate	*** apicalis ***
–	Spines interstriae 3 backwardly hooked, subequal to the spine at the summit of interstriae 2; larger, 3.35–3.7 mm; declivity moderately sulcate	*** cristatus ***

#### Anisandrus achaete sp. nov.

Taxon classificationAnimaliaColeopteraCurculionidae

http://zoobank.org/53ED4F36-7BC7-4354-945D-2EE41160D8D9

Fig. 18A, B, I

##### Type material.

***Holotype***, female, 云南 勐养 700m 寄主:栎 1984.VII.19 [China: Yunnan, Mengyang, 700 m, 19.vii.1984, ex Fagaceae] (NMNH). ***Paratype***, female, as holotype (IZAS).

##### Diagnosis.

3.5 mm long (mean = 3.5 mm; n = 2); 2.33× as long as wide. This species is distinguished by the mesonotal mycangial tuft absent; antennal club type 2, one suture on posterior face; elytral disc with a weak transverse saddle-like depression near declivital summit; declivity unarmed by spines; declivital striae strongly impressed, interstriae granulate; and anterior margin of pronotum without serrations.

##### Similar species.

*Anisandrus apicalis*.

##### Description

**(female).** 3.5 mm long (mean = 3.5 mm; n = 2); 2.33× as long as wide. Body bicolored with pronotal and elytral bases light brown, remainder of elytra red-brown. Head, legs, and antennae light brown. ***Head***: epistoma entire, transverse, with a row of hair-like setae. Frons weakly convex to upper level of eyes, strongly shiny, finely punctate; lateral areas weakly rugose, setose; each shallow ruga or puncture bearing a very long, erect hair-like seta. Eyes shallowly emarginate just above antennal insertion, upper part smaller than lower part. Submentum large, distinctly triangular, slightly impressed. Antennal scape regularly thick, as long as club. Pedicel as wide as scape, shorter than funicle. Funicle 4-segmented, segment 1 shorter than pedicel. Club wider than long, obliquely truncate, type 2; segment 1 corneous, transverse on anterior face, occupying basal 2/5, nearly covering posterior face; segment 2 narrow, corneous; segment 1 present on posterior face. ***Pronotum***: 0.89× as long as wide. In dorsal view basic, type 2, sides parallel in basal 1/2, rounded anteriorly; anterior margin without serrations. In lateral view basic, type 0, disc as long as anterior slope, summit at apical 2/5. Anterior slope with densely spaced, large fine asperities, becoming lower and more strongly transverse towards summit. Disc impressed behind summit, shiny, impunctate, glabrous, some long hair-like setae at margins. Lateral margins obliquely costate. Base transverse, posterior angles acutely rounded. Mycangial tuft absent. ***Elytra***: 1.55 × as long as wide, 1.75× as long as pronotum. Scutellum narrow, moderately sized, linguiform, flush with elytra, flat, shiny. Elytral base transverse, edge oblique, humeral angles rounded, parallel-sided in basal 2/3, then broadly rounded to apex; surface shiny. Disc with a weak medial transverse saddle-like depression, striae 1–3 distinctly impressed, other striae not impressed, punctures small, deep, separated by 2–4 diameters of a puncture, glabrous; interstriae glabrous, unarmed, interstriae 1–4 feebly convex, punctate, punctures minute, confused. Declivity occupying approximately 1/3 of elytra, steeply rounded, declivital face flattened; striae deeply impressed, strial punctures much larger and deeper than those of disc; interstriae impunctate, uniseriate granulate, granules bearing setae 1.5× width of interstriae 2, erect, hair-like, interstriae 3 narrower than interstriae 2 at midpoint of declivity. Posterolateral margin rounded, unarmed by granules. ***Legs***: procoxae contiguous, prosternal coxal piece tall and pointed. Protibiae distinctly triangular, broadest at apical 4/5, posterior face smooth; apical 1/2 of outer margin with eight moderate socketed denticles, their length slightly longer than basal width. Mesotibiae flattened, distinctly triangular, apical 1/2 with nine moderate socketed denticles on outer margin; metatibiae flattened, obliquely triangular, apical 1/2 with nine moderate socketed denticles on outer margin.

##### Etymology.

G. *a* = without; *chaite* = long hair. In reference to the uncharacteristically reduced number of elytral setae. Noun in apposition.

##### Distribution.

China (Yunnan).

##### Host plants.

Recorded from Fagaceae.

##### Remarks.

Locality labels on the holotype and paratype are in Chinese and were translated by You Li. An English locality label has been placed on each specimen below the original locality labels.

#### Anisandrus apicalis

Taxon classificationAnimaliaColeopteraCurculionidae

(Blandford, 1894)

Fig. 18C, D, J

Xyleborus apicalis Blandford, 1894b: 105.
Ambrosiodmus apicalis (Blandford): Wood 1989: 169.
Anisandrus apicalis (Blandford): Hulcr et al. 2007: 578.

##### Type material.

***Holotype*** (NHMUK).

##### New records.

China: Jiangxi, Wu-Yi Mt., 17.vii.2017, Lai, S-C, Tian, S et al. (RABC, 1). Sichuan, Jiuzhago Nature Reserve, 33°08.865'N, 103°55.134'E, 2483 m, 5.vii.2005, A.I. Cognato, ex *Pinus armandii* (MSUC).

##### Diagnosis.

3.05–3.4 mm long (mean = 3.17 mm; n = 5); 2.33–2.43× as long as wide. This species is distinguished by the mesonotal mycangial tuft the length of the scutellum; elytral disc with or without a weak transverse saddle-like depression; declivital posterolateral margin costate to interstriae 5; declivity appearing bisulcate, weakly impressed from striae 1 and 2, interstriae 3 feebly inflated and tuberculate from base to apical 1/2 then becoming flattened and unarmed to apex; and moderately sized sharp incurved spine at base of declivity on interstriae 2.

This species strongly resembles *A. cristatus* and *A. congruens* and is most easily distinguished by the moderate size, the less strongly impressed declivital sulci and smaller spines on interstriae 3 that are not backwardly hooked and much smaller than the spine at the summit of interstriae 2.

##### Similar species.

*Anisandrus congruens*, *A. cristatus*, *A. geminatus*, *A. niger*, *A. sinivali*, *A. venustus*.

##### Distribution.

China (Anhui, Guangxi, Guizhou, Hainan, Jiangxi*, Shanxi, Sichuan, Xizang, Yunnan), India (Meghalaya, Sikkim, West Bengal), Japan, South & North Korea, Kuril Islands, Nepal, Thailand.

##### Host plants.

A polyphagous species usually attacking angiosperms, but also recorded from *Pinus* (Pinaceae) (Murayama 1936; Nobuchi 1966).

##### Remarks.

Published records from India, Nepal, Thailand, and some Chinese provinces may refer to *Anisandrus cristatus* or *A. congruens*, with which *A. apicalis* has been confused previously.

#### Anisandrus auco sp. nov.

Taxon classificationAnimaliaColeopteraCurculionidae

http://zoobank.org/97A9EB18-B9CC-4BDF-91FC-E636111196F5

Fig. 18E, F, K

##### Type material.

***Holotype***, female, Vietnam: Cao Bang, 22°36.3'N, 105°52.6'E, 1435–1601 m, 13–17.iv.2014, VN16, Cognato, Smith, Pham, ex FIT (MSUC).

##### Diagnosis.

2.9 mm long (n = 1); 2.23× as long as wide. This species is distinguished by the mesonotal mycangial tuft the length of the scutellum; elytral disc flat; declivital interstriae clearly punctate; declivity gradual and convex, posterolateral margins rounded; pronotum rounded when viewed dorsally (type 1); and pronotum armed by four uniformly sized coarse serrations on anterior margin.

##### Similar species.

*Anisandrus cryphaloides*.

##### Description

**(female).** 2.9 mm long (n = 1); 2.23× as long as wide. Body bicolored with pronotal and elytral bases lighter than rest of body. Pronotal and elytral bases, head, legs, and antennae light brown, remainder of elytra red-brown. ***Head***: epistoma entire, transverse, with a row of hair-like setae. Frons weakly convex to upper level of eyes, subshiny, punctate; punctures large, shallow, dense; punctures bearing a long, erect hair-like seta. Eyes shallowly emarginate just above antennal insertion, upper part smaller than lower part. Submentum large, distinctly triangular, slightly impressed. Antennal scape regularly thick, as long as club. Pedicel as wide as scape, shorter than funicle. Funicle 4-segmented, segment 1 as long as pedicel. Club longer than wide, obliquely truncate, type 1; segment 1 corneous, encircling anterior face; segment 2 narrow, concave, corneous on anterior face only; sutures absent on posterior face. ***Pronotum***: 0.85× as long as wide. In dorsal view rounded, type 1, sides convex, rounded anteriorly; anterior margin with a row of four very large, coarse serrations. In lateral view short and tall, type 3, disc as long as anterior slope, summit at midpoint. Anterior slope with densely spaced, very large coarse asperities, becoming lower and more strongly transverse towards summit. Disc subshiny with moderately dense, large, shallow punctures bearing moderate, semi-recumbent, hair-like setae, some longer hair-like setae at margins. Lateral margins obliquely costate. Base transverse, posterior angles acutely rounded. Mycangial tuft present along basal margin, tuft moderately setose, approximately the width of scutellum. ***Elytra***: 1.49× as long as wide, 1.75× as long as pronotum. Scutellum broad, large, linguiform, flush with elytra, flat, shiny. Elytral base transverse, edge oblique, humeral angles rounded, parallel-sided in basal 1/2, then broadly rounded to apex; surface shiny. Disc flat, striae not impressed, with moderately-sized, deep punctures separated by less than one diameter of a puncture, setose, setae as long as two punctures, recumbent, hair-like; interstriae flat, punctate, punctures strongly confused, setose, setae 1× width of interstriae 2, erect, hair-like, unarmed by granules. Declivity occupying approximately 2/5 of elytra, gradually rounded, declivital face convex; striae weakly impressed, strial punctures larger and deeper than those of disc, punctures setose, setae slightly longer than the diameter of a puncture, semi-erect, hair-like; interstriae uniseriate punctate, setae 2× width of interstriae 2, erect, hair-like, interstriae 2 as wide as interstriae 3 at midpoint of declivity. Posterolateral margin rounded, unarmed by granules. ***Legs***: procoxae contiguous. Protibiae obliquely triangular, broadest at apical 1/3; posterior face smooth; apical 1/2 of outer margin with six large socketed denticles, their length longer than basal width. Meso- and metatibiae flattened; outer margins evenly rounded with nine and ten small socketed denticles, respectively.

##### Etymology.

Vietnamese mythology, Âu Cơ – mountain fairy that gave birth to the ancestors of the Vietnamese people. Pronunciation – *ò-ghá.* Noun in apposition.

##### Distribution.

Vietnam.

##### Host plants.

Unknown.

**Figure 18.**
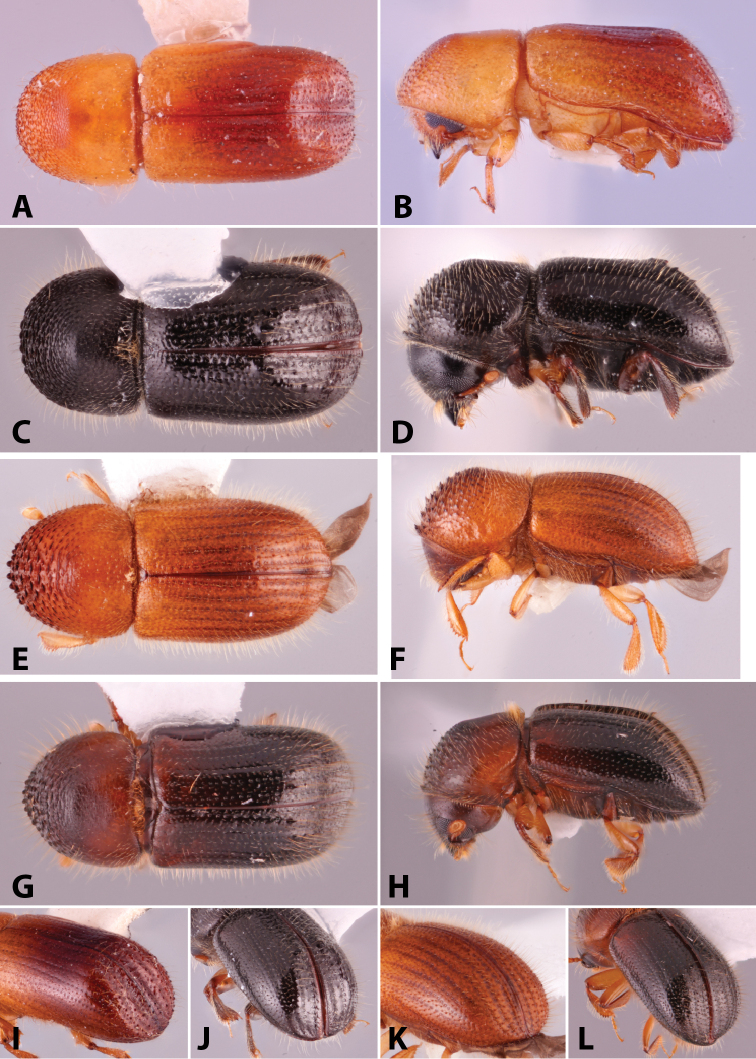
Dorsal, lateral and declivital view of *Anisandrus achaete* holotype, 3.5 mm (**A, B, I**), *A. apicalis*, 3.05–3.4 mm (**C, D, J**), *A. auco* holotype, 2.9 mm (**E, F, K**), and *A. auratipilus* holotype, 2.8 mm (**G, H, L**).

#### Anisandrus auratipilus sp. nov.

Taxon classificationAnimaliaColeopteraCurculionidae

http://zoobank.org/07D53FDF-F903-4459-8B69-EE3C1D1DFEFE

Fig. 18G, H, L

##### Type material.

***Holotype***, female, China: Fujian, Fuzhou, 18.iii.2018, Y. Li, ex unknown twig (IZAS). ***Paratypes***, female, as holotype (MSUC, 2).

##### Diagnosis.

2.8 mm long (n = 1); 2.15× as long as wide. This species is distinguished by the moderately dense mesonotal mycangial tuft that extends laterally from the scutellum to striae 3; declivital posterolateral margin rounded; elytral disc flat; declivital face moderately steep, convex; declivital interstriae 1 unarmed; declivital summit with a small denticle on interstriae 2 and a minute denticle on interstriae 3; interstriae 3 with three denticles on basal 1/2; declivital striae weakly impressed, punctures small, shallow and seriate; interstriae convex, minutely punctate, punctures strongly confused, setose, setae erect hair-like; body shiny, abundantly covered with long erect hair-like setae; elytral disc finely punctate; and pronotal asperities large, coarse, moderately spaced.

##### Similar species.

*Anisandrus apicalis*, *A. hera*, *A. klapperichi*, *A. percristatus*, *A. venustus*, *A. xuannu*.

##### Description

**(female).** 2.8 mm long (n = 1); 2.15× as long as wide. Body bicolored with pronotal and elytral bases lighter than rest of body. Pronotal and elytral bases brown, remainder of elytra and head dark brown. Legs and antennae light brown. ***Head***: epistoma entire, transverse, with a row of hair-like setae. Frons weakly convex to upper level of eyes, impunctate, median area of with a small ovate smooth, glabrous, strongly shiny area; lateral areas shagreened, weakly rugose, setose, each shallow ruga bearing a long, erect hair-like seta. Eyes shallowly emarginate just above antennal insertion, upper part smaller than lower part. Submentum large, distinctly triangular, slightly impressed. Antennal scape regularly thick, as long as club. Pedicel as wide as scape, shorter than funicle. Funicle 4-segmented, segment 1 shorter than pedicel. Club longer than wide, obliquely truncate, type 1; segment 1 corneous, encircling anterior face; segment 2 narrow, concave, corneous on anterior face only; sutures absent on posterior face. ***Pronotum***: 0.7× as long as wide. In dorsal view conical, type 0, sides convex, conical anteriorly; anterior margin with a row of four moderate serrations. In lateral view type 3, short and tall, disc as long as anterior slope, summit at midpoint. Anterior slope with moderately spaced, large, coarse, asperities, becoming lower and more strongly transverse towards summit. Disc subshiny with dense, large, shallow punctures bearing short to moderate, erect hair-like setae, some longer hair-like setae at margins. Lateral margins obliquely costate. Base transverse, posterior angles broadly rounded. Mycangial tuft present along basal margin tuft broad, moderately setose, laterally extending to elytral striae 3. ***Elytra***: 1.6× as long as wide, 2.26× as long as pronotum. Scutellum broad, large, linguiform, flush with elytra, flat, shiny. Elytral base transverse, edge oblique, humeral angles rounded, parallel-sided in basal 2/3, then narrowly rounded to apex; surface shiny. Disc flat, striae not impressed, with small, shallow punctures separated by one diameter of a puncture, setose, setae as long as a puncture, semi-recumbent, hair-like; interstriae flat, minutely punctate, punctures strongly confused, setose, setae 1× width of interstriae 2, erect hair-like, unarmed by granules. Declivity occupying approximately 2/5 of elytra, steeply rounded, declivital face convex; striae weakly impressed, strial punctures somewhat larger and deeper than those of disc, and bearing setae as described for disc; interstriae sparsely minutely uniseriate punctate, setae 1–1.5× width of interstriae 2, erect, hair-like, interstriae 2 as wide as interstriae 3 at midpoint of declivity, declivital summit with a small denticle on interstriae 2 and a minute denticle on interstriae 3; interstriae 3 with three denticles on basal 1/2. Posterolateral margin rounded, unarmed by granules. ***Legs***: procoxae contiguous; prosternal coxal piece short, inconspicuous. Protibiae obliquely triangular, broadest at apical 1/3; posterior face smooth; apical 1/2 of outer margin with five large socketed denticles, their length longer than basal width. Meso- and metatibiae flattened; outer margins evenly rounded with seven and eight large socketed denticles, respectively.

##### Etymology.

L. *auratus* = golden; *pilus* = hair. In reference to the golden setae covering the elytra. Noun in apposition.

##### Distribution.

China (Fujian).

##### Host plants.

Unknown.

#### Anisandrus carinensis

Taxon classificationAnimaliaColeopteraCurculionidae

(Eggers, 1923) comb. nov.

Fig. 19A, B, I

Xyleborus carinensis Eggers, 1923: 180.

##### Type material.

***Holotype*** (MCG).

##### Diagnosis.

4.5 mm long (n = 1); 2.25× as long as wide. This species is distinguished by the mesonotal mycangial tuft absent; antennal club type 1 with segment 1 encircling anterior face; elytral disc with a weak transverse saddle-like depression; declivital interstriae 1 and 3 armed by four or five unequally sized tubercles; and a row of serrations on anterior margin of pronotum.

##### Similar species.

*Anisandrus achaete*.

##### Distribution.

Myanmar.

##### Host plants.

Unknown.

##### Remarks.

The species has the generic characters of *Anisandrus* and is here transferred to that genus.

**Figure 19.**
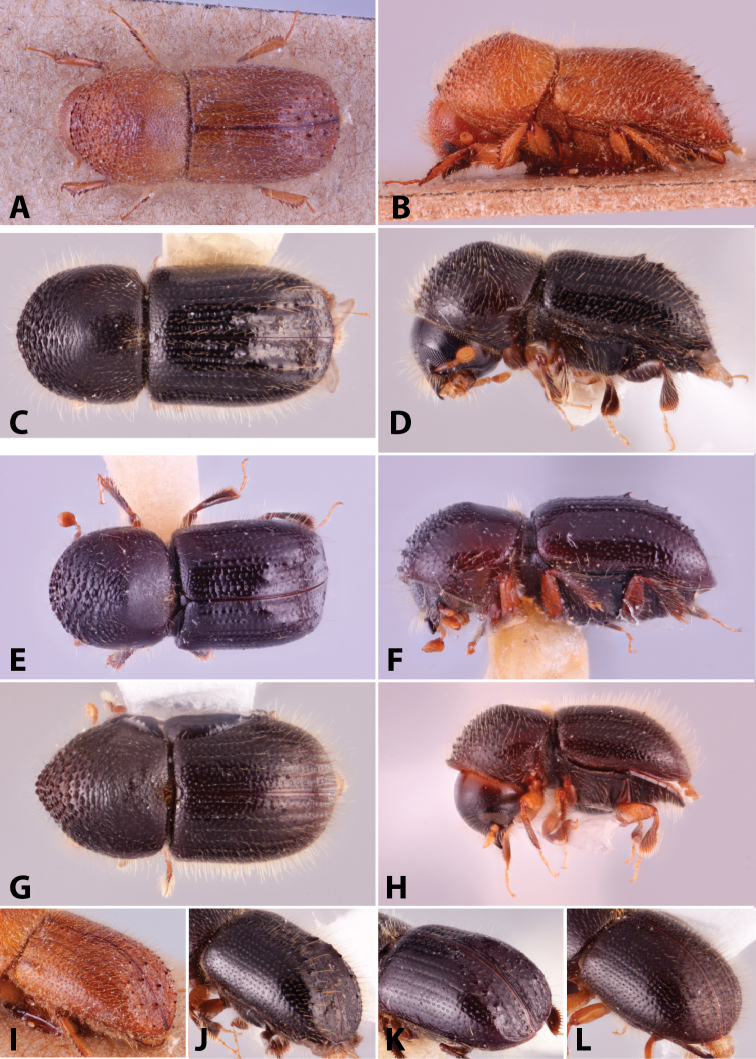
Dorsal, lateral and declivital view of *Anisandrus carinensis* holotype, 4.5 mm (**A, B, I**), *A. congruens* holotype, 2.6–2.8 mm (**C, D, J**), *A. cristatus*, 3.35–3.7 mm (**E, F, K**), and *A. cryphaloides* holotype, 2.1–2.4 mm (**G, H, L**).

#### Anisandrus congruens sp. nov.

Taxon classificationAnimaliaColeopteraCurculionidae

http://zoobank.org/BD7E0ACE-07E2-47D2-A3CE-A160BFA5ED5C

Fig. 19C, D, J

##### Type material.

***Holotype***, female, Vietnam: Cao Bang, 22°36.3'N, 105°52.6'E, 1435–1601 m, 13–17.iv.2014, VN16, Cognato, Smith, Pham, ex FIT (MSUC). ***Paratypes***, female, Thailand: Chiang Mai, Doi Pui, 1400 m, 20–24.xii.2004, W. Puranasakul, ex EtOH trap (RABC, 1); Vietnam: Lao Cai, Hoang Lien N.P., 22.35, 103.77, 1500–2000 m, 19.v.2019, VN169, S.M. Smith, A.I. Cognato (MSUC, 1); as previous except: 18–19.v.2019, ex FIT (MSUC, 1).

##### Diagnosis.

2.6–2.8 mm long (mean = 2.7 mm; n = 2); 2.16–2.36× as long as wide. This species is distinguished by the mesonotal mycangial tuft the length of the scutellum; elytral disc with a weak to moderate transverse saddle-like depression; posterolateral margin costate to interstriae 5; declivity appearing bisulcate, moderately impressed from striae 1 and 2, interstriae 3 strongly inflated, tuberculate from summit to apical 1/4 then becoming flattened and unarmed to apex; and moderate sharp backwardly pointed spine at base of declivital interstriae 2.

This species strongly resembles *A. apicalis* and *A. cristatus* and is most easily distinguished by the smaller size, more strongly impressed declivital sulci than *A. apicalis* and larger spines on interstriae 3 that are sharply pointed but not strongly backwardly hooked.

##### Similar species.

*Anisandrus apicalis*, *A. cristatus*, *A. geminatus*, *A. niger*, *A. sinivali*.

##### Description

**(female).** 2.6–2.8 mm long (mean = 2.7 mm; n = 2); 2.16–2.36× as long as wide. Body uniformly dark brown, except dark red-brown declivity. Legs and antennae light brown. ***Head***: epistoma entire, transverse, with a row of hair-like setae. Frons weakly convex to upper level of eyes, alutaceous, subshiny, punctate; punctures large, shallow, setose; punctures bearing a long, erect hair-like seta. Eyes shallowly emarginate just above antennal insertion, upper part smaller than lower part. Submentum large, distinctly triangular, slightly impressed. Antennal scape regularly thick, as long as club. Pedicel as wide as scape, shorter than funicle. Funicle 4-segmented, segment 1 shorter than pedicel. Club longer than wide, obliquely truncate, type 1; segment 1 corneous, encircling anterior face; segment 2 narrow, concave, corneous on anterior face only; sutures absent on posterior face. ***Pronotum***: 1.0× as long as wide. In dorsal view rounded, type 1, sides convex, rounded anteriorly; anterior margin with a row of four serrations. In lateral view robust and rounded, type 5, disc as long as anterior slope, summit at midpoint. Anterior slope with densely spaced, large coarse asperities, becoming lower and more strongly transverse towards summit. Disc subshiny, alutaceous with sparse fine punctures bearing short, recumbent, hair-like setae, some longer hair-like setae at margins. Lateral margins obliquely costate. Base transverse, posterior angles acutely rounded. Mycangial tuft present along basal margin, tuft moderately setose, approximately the width of scutellum. ***Elytra***: 1.5× as long as wide, 1.5× as long as pronotum. Scutellum broad, large, linguiform, flush with elytra, flat, shiny. Elytral base transverse, edge oblique, humeral angles rounded, parallel-sided in basal 1/2, then broadly rounded to apex; surface shiny. Disc with a weak to moderate medial transverse saddle-like depression, striae not impressed, with small, deep punctures separated by two diameters of a puncture, setose, setae as long as a puncture, recumbent, hair-like; interstriae flat, punctate, punctures uniseriate subequal to those of striae, setose, setae 1× width of interstriae 2, erect, hair-like, unarmed by granules. Declivity occupying approximately 1/2 elytra, evenly rounded, declivital face weakly bisulcate, moderately impressed from striae 1 and 2, interstriae 3 strongly inflated, tuberculate from summit to apical 1/4 then becoming flattened and unarmed to apex; striae not impressed, strial punctures much larger and deeper than those of disc, and bearing setae as described for disc; interstriae impunctate, sparsely minutely granulate, setae 1–2× width of interstriae 2, erect, hair-like, interstriae 2 as wide as interstriae 3 at midpoint of declivity, declivital summit with a moderate sharp backwardly pointed spine at base of declivital interstriae 2. Posterolateral margin costate to interstriae 5. ***Legs***: procoxae contiguous; prosternal coxal piece short, inconspicuous. Protibiae obliquely triangular, broadest at apical 1/3; posterior face smooth; apical 1/2 of outer margin with seven large socketed denticles, their length longer than basal width. Meso- and metatibiae flattened; outer margins evenly rounded with eight and ten large socketed denticles, respectively.

##### Etymology.

L. *congruens* = agreeing with. In reference to its similarity to *apicalis* and *cristatus*. A participle.

##### Distribution.

Thailand, Vietnam.

##### Host plants.

Unknown.

#### Anisandrus cristatus

Taxon classificationAnimaliaColeopteraCurculionidae

(Hagedorn, 1908) comb. nov., stat. res.

Fig. 19E, F, K

Xyleborus cristatus Hagedorn, 1908: 377.
Xyleborus fabricii Schedl, 1964c: 217. Unnecessary replacement name.

##### Type material.

***Syntypes*** (IRSNB). Not examined.

##### New records.

China: Yunnan, Gaoligong Mts, 24.57; 98.45, 2200–2500 m, 8–16.v.1995, V. Kuban (NHMB, 3; RABC, 1). India: [West Bengal], Darjeeling D[istrict], Rally, 850 m, 3.iv.1979, Bhakta B. (NHMB, 1); as previous except: Lepchajagat 7000 ft, 11.ix.1929, J.C.M. Gardner, ex *Symplocos theaefolia* (NMNH, 1); as previous except: Rangirum, 6000 ft (NMNH, 1). Laos: NE, Hua Phan, Ban Saluei, Phou Pan (Mt.), 20°12'N, 104°01'E, 1300–1900 m, 7.iv–25.v.2010, C. Holzschuh (NHMUK, 4; RABC, 1). Myanmar: Kambaiti, 7000 ft, 22.iv.1934, R. Malaise (NMNH, 1). Nepal: Arun Valley, Deurali, 27°30'N, 87°16'E, ~ 2100 m NN, 10.v.2014, J. Schmidt (NKME, 3); Koshi, Gorza, 2100 m, 5–6.vi.1985, M. Brancucci (NHMB, 9; RABC, 2); Kathmandu V[alley], Gufa–Gorza, 2800–2100 m, M. Brancucci (NHMB, 4); Koli Gandaki Khola, Chitra, Ghar Khola, 2400 m, Bhakta B. (NHMB, 1); Manaslu Mts, E slope of Ngadi Khola valley, 28°22'N, 84°29'E, 2000–2300 m, 14–16.v.2005, J. Schmidt (RABC, 2). Thailand: Chiang Mai, Doi Inthanon, 5.viii.[20]02, R. A. Beaver (RABC, 1). Vietnam: Cao Bang, 22°36.402'N, 105°52.397'E, 1601 m, 13.iv.2014, VN17, Cognato, Smith, Pham, ex standing stump (MSUC, 1). Lao Cai, Hoang Lien N.P., 22.35, 103.77, 1500–2000 m, 19.v.2019, VN171, S.M. Smith, A.I. Cognato, ex 1 cm DBH dead sapling (MSUC, 1).

##### Diagnosis.

3.35–3.7 mm long (mean = 3.55 mm; n = 5); 2.2–2.47× as long as wide. This species is distinguished by the mesonotal mycangial tuft the length of the scutellum; elytral disc with or without a weak transverse saddle-like depression; declivital posterolateral margin costate to interstriae 5; declivity appearing bisulcate, moderately impressed from striae 1 and 2, interstriae 3 moderately inflated, tuberculate from base to apical 1/4 then becoming flattened and unarmed to apex; and large sized sharp incurved spine on interstriae 2 at base of declivity.

This species strongly resembles *A. apicalis* and *A. congruens* and is most easily distinguished by the larger size, more strongly impressed declivital sulci than *A. apicalis* and larger spines on interstriae 3 that are sharply pointed and backwardly hooked and subequal in size to the spine at the summit of interstriae 2.

##### Similar species.

*Anisandrus apicalis*, *A. congruens*, *A. geminatus*, *A. niger*, *A. sinivali*.

##### Distribution.

Bhutan*, China* (Yunnan), India (Meghalaya, ‘Naga Hills’, Sikkim, West Bengal), Laos*, Myanmar*, Nepal*, Thailand*, Vietnam*.

##### Host plants.

This species has been recorded from *Alnus* (Betulaceae), *Quercus* (Fagaceae), *Symplocos* (Symplocaceae) (Beeson 1930).

##### Remarks.

*Xyleborus cristatus* has the generic characters of *Anisandrus* and is here transferred to that genus. This species was synonymized with *Ambrosiodmus apicalis* (Blandford) [*sic*] by Wood (1989). It is here removed from synonymy and reinstated as a distinct species, based on the characters given above, and differences in DNA (Cognato et al. 2020b).

#### Anisandrus cryphaloides sp. nov.

Taxon classificationAnimaliaColeopteraCurculionidae

http://zoobank.org/7C75FBB1-6168-4E35-BC38-6C62FE3882DD

Fig. 19G, H, L

##### Type material.

***Holotype***, female, Vietnam: Cao Bang, 22°36.804'N, 105°51.982'E, 1831 m, 17.iv.2014, VN42, Cognato, Smith, Pham, ex 0.3–3 cm twigs/branches (NMNH). ***Paratypes***, female, as holotype (MSUC, 4; NHMUK, 2; NMNH, 2; VMNH 1); Lao Cai, Hoang Lien N.P., 22.35, 103.77, 1500 m, 17.v.2019, VN152, S.M. Smith, A.I. Cognato, ex 1–3 cm branch (MSUC, 3); as previous except: VN153, ex branch; 1–2 cm (MSUC, 1); as previous except: 1500–2000 m, 20.v.2019, VN185, ex branch; 1–2 cm (MSUC, 1); 1500–2000 m, 20.v.2019, VN186, ex branch; 1–2 cm (MSUC, 1).

##### Diagnosis.

2.1–2.4 mm long (mean = 2.26 mm; n = 5); 2.2–2.4× as long as wide. This species is distinguished by the mesonotal mycangial tuft the length of the scutellum; elytral disc convex; declivity gradual and convex, with rounded posterolateral margins; pronotum conical frontally when viewed dorsally (type 0); pronotum armed by four coarse serrations on anterior margin (median pair larger than lateral pair); elytra strongly shagreened or opalescent; and declivital striae weakly impressed.

##### Similar species.

*Anisandrus auco*.

##### Description

**(female).** 2.1–2.4 mm long (mean = 2.26 mm; n = 5); 2.2–2.4× as long as wide. Body dark brown. Antennae and legs light brown. ***Head***: epistoma entire, transverse, with a row of hair-like setae. Frons weakly convex to upper level of eyes, alutaceous, subshiny, punctate, punctures large, shallow, setose; punctures bearing a long, erect hair-like seta. Eyes feebly emarginate, almost entire, just above antennal insertion, upper part smaller than lower part. Submentum large, distinctly triangular, slightly impressed. Antennal scape regularly thick, as long as club. Pedicel as wide as scape, shorter than funicle. Funicle 4-segmented, segment 1 as long as pedicel. Club longer than wide, obliquely truncate, type 1; segment 1 corneous, encircling anterior face; segment 2 narrow, concave, corneous on anterior face only; sutures absent on posterior face. ***Pronotum***: 0.89× as long as wide. In dorsal view conical, type 0, sides convex, conical anteriorly; anterior margin with a row of four coarse serrations, median pair larger than lateral pair. In lateral view type 3, short and tall, disc as long as anterior slope, summit at midpoint. Anterior slope with moderately spaced, large coarse asperities, becoming lower and more strongly transverse towards summit. Disc strongly shiny with moderately dense, large, shallow punctures bearing moderate, erect, hair-like setae or short, recumbent, hair-like setae, some longer hair-like setae at margins. Lateral margins obliquely costate. Base transverse, posterior angles acutely rounded. Mycangial tuft present along basal margin, tuft moderately setose, approximately the width of scutellum. ***Elytra***: 1.26× as long as wide, 1.4× as long as pronotum. Scutellum broad, large, linguiform, flush with elytra, flat, shiny. Elytral base transverse, edge oblique, humeral angles rounded, parallel-sided in basal 1/2, then broadly rounded to apex; surface opalescent to shagreened. Disc convex, striae not impressed, with small, shallow punctures separated by less than one diameter of a puncture, setose, setae as long as two punctures, recumbent, hair-like; interstriae flat, punctate, punctures strongly confused, setose, setae longer than the width of interstriae 2, erect hair-like, unarmed by granules. Declivity occupying approximately 1/2 elytra, gradually rounded, declivital face convex; striae weakly impressed, strial punctures somewhat larger and deeper than those of disc; interstriae sparsely uniseriate punctate, setae 2–3× width of an interstria, erect, hair-like, interstriae 3 narrower than interstriae 2 at midpoint of declivity, interstriae 2 with a small incurved spine at declivital summit. Posterolateral margin rounded, unarmed by granules. ***Legs***: procoxae contiguous; prosternal coxal piece short, inconspicuous. Protibiae obliquely triangular, broadest at apical 1/3; posterior face smooth; apical 1/2 of outer margin with six very large socketed denticles, their length much longer than basal width. Meso- and metatibiae flattened; outer margins evenly rounded with eight very large socketed denticles.

##### Etymology.

Resembling *Cryphalus* Erichson, 1836, in reference to the coarse asperities in concentric rows on the anterior half of the pronotum. Noun in apposition.

##### Distribution.

Vietnam.

##### Host plants.

Unknown.

#### Anisandrus dispar

Taxon classificationAnimaliaColeopteraCurculionidae

(Fabricius, 1792)

Fig. 20A, B, I

Apate dispar Fabricius, 1792: 363.
Anisandrus dispar (Fabricius): Ferrari 1867: 24.
Xyleborus dispar (Fabricius): Hagedorn 1910b: 98.
Anisandrus dispar (Fabricius): Hulcr et al. 2007: 578.
Bostrichus thoracicus Panzer, 1793: 34. Synonymy: Hagedorn 1910b: 102.
Scolytus pyri Peck, 1817: 207. Synonymy: Hubbard 1897: 22; Swaine 1918: 124.
Bostrichus tachygraphus Sahlberg, 1836: 152. Synonymy: Eichhoff 1876b: 378.
Bostrichus ratzeburgi Kolenati, 1846: 39. Synonymy: Ferrari 1867: 27.
Xyleborus ishidai Niisima, 1909: 156. Synonymy: Smith et al. 2018b: 393.
Anisandrus aequalis Reitter, 1913: 81. Synonymy: Knížek 2011: 242.
Anisandrus swainei Drake, 1921: 203. Synonymy: Wood 1957: 403.
Xyleborus dispar rugulosus Eggers, 1922: 17. Synonymy: Schedl 1964d: 314.
Xyleborus cerasi Eggers, 1937: 335. Synonymy: Schedl 1964c: 220.
Xyleborus khinganensis Murayama, 1943: 100. Synonymy: Knížek 2011: 242.

##### Type material.

***Holotype****Anisandrus swainei* (NMNH). ***Lectotype****Xyleborus dispar rugulosus* (NMNH). ***Lectotype****Xyleborus ishidai* (NIAES). ***Holotype****Xyleborus khinganensis* (NMNH).

##### Diagnosis.

3.1–3.5 mm long (mean = 3.4 mm; n = 5); 2.27–2.5× as long as wide. This species is distinguished by the mesonotal mycangial tuft sparse, the length of the scutellum; declivital interstriae uniseriate granulate; discal interstriae with two or three confused rows of punctures; declivital interstriae 1 slightly raised, interstriae 2 and 3 even; declivital face smooth, shiny; and declivital interstrial setae erect, 1.5× the width of an interstria.

##### Similar species.

*Anisandrus maiche*, *A. paragogus*, *Xylosandrus germanus*.

##### Distribution.

Europe and North Africa, through Russia and Central Asia to China (Heilongjiang, Shaanxi), North Korea, and Japan. Introduced to Canada and USA (Wood 1977; Gomez et al. 2018a).

##### Host plants.

Polyphagous attacking both angiosperms and conifers (Wood and Bright 1992; Beaver et al. 2014).

##### Remarks.

The biology of the species is described by Palm (1959), Chararas (1962), Egger (1973), and French and Roeper (1975). Speranza et al. (2009) examine the effects of temperature and rainfall on flight activity. Like many xyleborines, the species is attracted to ethanol (Saruhan and Akyol 2012; Galko et al. 2014). It is an important pest of hazel (*Corylus avellana*) (Betulaceae) in the Mediterranean area (e.g., Bucini et al. 2005; Saruhan and Akyol 2012), and an occasional pest of fruit trees in the USA (Wood 1982).

**Figure 20.**
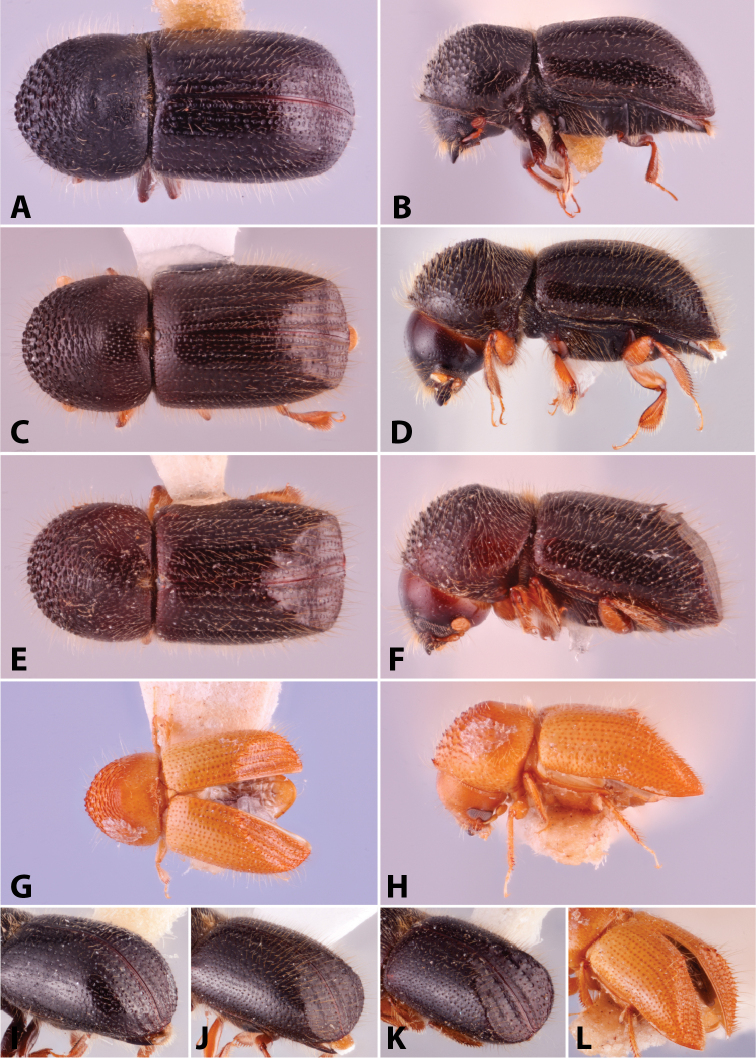
Dorsal, lateral and declivital view of *Anisandrus dispar*, 3.1–3.5 mm (**A, B, I**), *A. eggersi*, 3.1–3.2 mm (**C, D, J**), *A. feronia* holotype, 2.9 mm (**E, F, K**), and *A. geminatus*, 2.9–3.2 mm (**G, H, L**).

#### Anisandrus eggersi

Taxon classificationAnimaliaColeopteraCurculionidae

(Beeson, 1930)

Fig. 20C, D, J

Xyleborus eggersi Beeson, 1930: 215.
Cyclorhipidion eggersi (Beeson): Maiti and Saha 2004: 105.
Anisandrus eggersi (Beeson): Hulcr et al. 2007: 578.

##### Type material.

***Paratypes*** (FRI, 1; NMNH, 1).

##### New records.

Bhutan: km 87 von Phuntsholing, 22.v.1972, Nat.-Hist. Museum Basel – Bhutan Expedition (NHMB, 1) [Misdetermined by K. E. Schedl as *Xyleborus fabricii* Schedl]. Thailand: Chiang Mai, Doi Inthanon, 5.viii.[20]02, R.A. Beaver, K. Koivisto (RABC, 1); as previous except: 13.xi.[20]11, W. Sittichaya (RABC, 2). Loei, Phu Hin Rongkla N. Park, Huai Man Daeng Naoi @ trail, 16°57'N, 101°03'E, 17.iii-10.iv.2003, G.W. Courtney, ex malaise trap (MSUC, 2). Vietnam: Cao Bang, 22°36.804'N, 105°51.982'E, 1831 m, 17.iv.2014, Cognato, Smith, Pham, 0.3–3.0 cm twigs/branches (MSUC, 10). Lao Cai, Hoang Lien N.P., 22.35, 103.77, 1500–2000 m, 20.v.2019, VN185, S.M. Smith, A.I. Cognato, ex branch; 1–2 cm (MSUC, 1); as previous except: 20.v.2019, VN194, ex dead sapling; 1 cm at base (MSUC, 1).

##### Diagnosis.

3.1–3.2 mm long (mean = 3.12 mm; n = 5); 2.21–2.29× as long as wide. This species is distinguished by the mesonotal mycangial tuft the length of the scutellum; elytral disc convex; declivity appearing flat when viewed laterally; two or three small tubercles present on basal 1/2 of interstriae 2; declivital posterolateral margin costate to interstriae 5; declivital face strongly shagreened; and declivital interstriae clearly punctate.

##### Similar species.

*Anisandrus feronia*, *A. improbus*, *A. mussooriensis*.

##### Distribution.

Bhutan*, India (West Bengal), Myanmar, Nepal, Thailand*, Vietnam*.

##### Host plants.

Polyphagous, recorded from five genera in five different families (Euphorbiaceae, Lauraceae, Rosaceae, Staphyleaceae, Symplocaceae) (Maiti and Saha 2004).

##### Remarks.

Maiti and Saha (2004) suggest that it is a high-altitude species.

#### Anisandrus feronia sp. nov.

Taxon classificationAnimaliaColeopteraCurculionidae

http://zoobank.org/E32E26AC-AA3F-40BC-B0C7-A72C1F3CEB2D

Fig. 20E, F, K

##### Type material.

***Holotype***, female, 福建 崇安 1500m 芥桔子 1978.V.7 采集者:黄復生 [China: Fujian, Chong’an, 1500 m, 7.v.1978, Shuyong Wang, ex *Fortunella margarita*] (NMNH). ***Paratypes***, female, as holotype (IZAS, 1; NMNH, 1).

##### Diagnosis.

2.9 mm long (mean = 2.9 mm; n = 3); 2.23× as long as wide. This species is distinguished by the mesonotal mycangial tuft the length of the scutellum; elytral disc flat; declivital interstriae punctate; declivital posterolateral margin carinate to interstriae 5; declivity moderately bisulcate; declivital margins ornamented by only two small sharp incurved spines at the base of interstriae 2 and 3; and declivital interstriae bearing fine erect hair-like setae.

##### Similar species.

*Anisandrus eggersi*, *A. longidens*, *A. mussooriensis*.

##### Description

**(female).** 2.9 mm long (mean = 2.9 mm; n = 3); 2.23× as long as wide. Body dark red-brown. Legs and antennae light brown. ***Head***: epistoma entire, transverse, with a row of hair-like setae. Frons weakly convex to upper level of eyes, alutaceous, subshiny, punctate; punctures large, shallow, setose; punctures bearing a long, erect hair-like seta. Eyes shallowly emarginate just above antennal insertion, upper part smaller than lower part. Submentum large, distinctly triangular, slightly impressed. Antennal scape regularly thick, as long as club. Pedicel as wide as scape, shorter than funicle. Funicle 4-segmented, segment 1 as long as pedicel. Club longer than wide, obliquely truncate, type 1; segment 1 corneous, encircling anterior face; segment 2 narrow, concave, corneous on anterior face only; sutures absent on posterior face. ***Pronotum***: 0.77× as long as wide. In dorsal view rounded, type 1, sides convex, rounded anteriorly; anterior margin with a row of 6–8 serrations. In lateral view short and tall, type 3, disc shorter than anterior slope, summit at basal 2/5. Anterior slope with densely spaced, large coarse asperities, becoming lower and more strongly transverse towards summit. Disc subshiny with dense, fine punctures bearing moderate, semi-erect hair-like setae, some longer hair-like setae at margins. Lateral margins obliquely costate. Base transverse, posterior angles acutely rounded. Mycangial tuft present along basal margin, tuft moderately setose, approximately the width of scutellum. ***Elytra***: 1.52× as long as wide, 1.97× as long as pronotum. Scutellum broad, large, linguiform, flush with elytra, flat, shiny. Elytral base transverse, edge oblique, humeral angles rounded, parallel-sided in basal 3/5, then narrowly rounded to apex; surface opalescent. Disc weakly convex, striae not impressed, with small, deep punctures separated by approximately one diameter of a puncture, setose, setae as long as two punctures, recumbent, hair-like; interstriae flat, punctate, punctures strongly confused, setose, setae 1–1.5× width of interstriae 2, erect, hair-like, unarmed by granules. Declivity occupying approximately 1/2 elytra, steeply rounded, declivital face moderately bisulcate to interstriae 4; striae not impressed, strial punctures much larger and deeper than those of disc, and bearing setae as described for disc; interstriae minutely uniseriate punctate, setae 1–1.5× width of interstriae 2, erect, hair-like, interstriae 2 as wide as interstriae 3 at midpoint of declivity, declivital margins ornamented by only two small sharp incurved spines at base of interstriae 2 and 3. Posterolateral margin carinate to interstriae 5. ***Legs***: procoxae contiguous; prosternal coxal piece short, inconspicuous. Protibiae obliquely triangular, broadest at apical 1/3; posterior face smooth; apical 1/2 of outer margin with seven very large socketed denticles, their length much longer than basal width. Meso- and metatibiae flattened; outer margins evenly rounded with nine and ten large socketed denticles, respectively.

##### Etymology.

Roman mythology, Feronia – goddess of wildlife, fertility, abundance. Noun in apposition.

##### Distribution.

China (Fujian).

##### Host plants.

Recorded from *Fortunella margarita* (Rutaceae).

##### Remarks.

Locality labels on the holotype and paratypes are in Chinese and were translated by You Li. An English locality label has been placed on the specimen below the original locality labels.

#### Anisandrus geminatus

Taxon classificationAnimaliaColeopteraCurculionidae

(Hagedorn, 1904)

Fig. 20G, H, L

Xyleborus geminatus Hagedorn, 1904: 126.
Amasa geminata (Hagedorn): Wood and Bright 1992: 683.
Anisandrus geminatus (Hagedorn): Beaver and Liu 2018: 537.

##### Type material.

The holotype was destroyed in the bombing of UHZM in World War II (Wood and Bright 1992).

##### New records.

India: Darjeeling, Rangirum, 6000 ft, J.C.M. Gardner, 3.ix.1929, ex misc. timber (NMNH, 1).

##### Diagnosis.

2.9–3.2 mm long (mean = 3.03 mm; n = 3); 2.31–2.37× as long as wide. This species is distinguished by the mesonotal mycangial tuft the length of the scutellum; elytral disc flat; declivital interstriae punctate; and posterolateral margin costate to interstriae 7; declivital face concave; declivital interstriae 2 and 3 each armed with a small sharp incurved spine at the summit; and declivital interstriae bearing erect pointed bristle-like setae.

##### Similar species.

*Anisandrus apicalis*, *A. congruens*, *A. cristatus*, *A. niger*, *A. sinivali*.

##### Distribution.

India (West Bengal), Nepal.

##### Host plants.

Unknown.

#### Anisandrus hera sp. nov.

Taxon classificationAnimaliaColeopteraCurculionidae

http://zoobank.org/1155CC1E-4DAF-40B9-8C45-DE700FBA0AF4

Fig. 21A, B, I

##### Type material.

***Holotype***, female, 四川 峨边 1900公尺 木合 川 1960-VI-29 采集者：殷惠芬 [China: Sichuan, E’bian; 1900 m, 29.vi.1960, Huifen Yin, ex *Schima superba*] (NMNH).

##### Diagnosis.

3.9 mm long (n = 1); 2.05× as long as wide. This species is distinguished by the dense mesonotal mycangial tuft that extends laterally from the scutellum to striae 3; declivital posterolateral margin obliquely costate to interstriae 5; elytral disc with a weak transverse saddle-like depression; declivital summit with large incurved spine on interstriae 2, interstriae 3 with two additional unequally sized denticles ventrad to large spine; declivity weakly sulcate to interstriae 3; declivital strial punctures large each bearing a recumbent seta, interstriae minutely punctate, punctures uniseriate, setose, setae erect, hair-like; body moderately sized and abundantly covered with long erect hair-like setae; declivity shiny; and pronotal asperities small, coarse, densely spaced.

##### Similar species.

*Anisandrus auratipilus*, *A. klapperichi*, *A. percristatus*, *A. venustus*, *A. xuannu*.

##### Description

**(female).** 3.9 mm long (n = 1); 2.05× as long as wide. Body dark brown. Legs and antennae light brown. ***Head***: epistoma entire, transverse, with a row of hair-like setae. Frons moderately impressed above epistoma then weakly convex to upper level of eyes, impunctate, median area of with a oval-shaped smooth, glabrous, strongly shiny area; lateral areas shagreened, coarsely rugose, setose; each ruga bearing a long, erect hair-like seta. Eyes shallowly emarginate just above antennal insertion, upper part smaller than lower part. Submentum large, distinctly triangular, slightly impressed. Antennal scape regularly thick, as long as club. Pedicel as wide as scape, shorter than funicle. Funicle 4-segmented, segment 1 as long as pedicel. Club longer than wide, obliquely truncate, type 1; segment 1 corneous, encircling anterior face; segment 2 narrow, concave, corneous on anterior face only; sutures absent on posterior face. ***Pronotum***: 0.89× as long as wide. In dorsal view rounded, type 1, sides convex, rounded anteriorly; anterior margin with a row of six large serrations. In lateral view type 3, short and tall, disc as long as anterior slope, summit at midpoint. Anterior slope with widely spaced, large coarse asperities, becoming lower and more strongly transverse towards summit. Disc subshiny, median area weakly rugose, lateral areas with dense, large, shallow punctures bearing moderate, erect hair-like setae, some longer hair-like setae at margins. Lateral margins obliquely costate. Base transverse, posterior angles broadly rounded. Mycangial tuft present along basal margin tuft broad, densely setose, laterally extending to elytral striae 3. ***Elytra***: 1.0× as long as wide, 1.13× as long as pronotum. Scutellum narrow, large, linguiform, flush with elytra, flat, shiny. Elytral base transverse, edge oblique, humeral angles rounded, parallel-sided in basal 2/3, then narrowly rounded to apex; surface shiny. Disc with a weak medial transverse saddle-like depression, striae not impressed, with small, shallow punctures separated by 2–4 diameters of a puncture, setose, setae as long as a puncture, recumbent, hair-like; interstriae flat, punctate, punctures strongly confused, setose, setae 1.5× width of interstriae 2, erect, hair-like, unarmed by granules. Declivity occupying approximately 1/2 elytra, evenly rounded, declivital face weakly sulcate to interstriae 3; striae not impressed, strial punctures somewhat larger and deeper than those of disc, and bearing setae as described for disc; interstriae sparsely minutely uniseriate punctate, setae 1–2× width of interstriae 2, erect, hair-like, interstriae 2 narrower than interstriae 3 at midpoint of declivity, declivital summit with a large incurved spine on interstriae 2, interstriae 3 costate with two additional unequally sized denticles ventrad to large spine. Posterolateral margin costate to interstriae 5. ***Legs***: procoxae contiguous; prosternal coxal piece short, inconspicuous. Protibiae distinctly triangular, broadest at apical 9/10; posterior face smooth; apical 1/2 of outer margin with seven large socketed denticles, their length longer than basal width. Meso- and metatibiae flattened; outer margins obliquely triangular with 11 and 14 small socketed denticles, respectively.

##### Etymology.

Greek mythology, Hera – goddess of women, marriage, family, and childbirth. Noun in apposition.

##### Distribution.

China (Sichuan).

##### Host plants.

Recorded from *Schima* (Theaceae).

##### Remarks.

Locality labels on the holotype are in Chinese and were translated by You Li. An English locality label has been placed on the specimen below the original locality labels.

**Figure 21.**
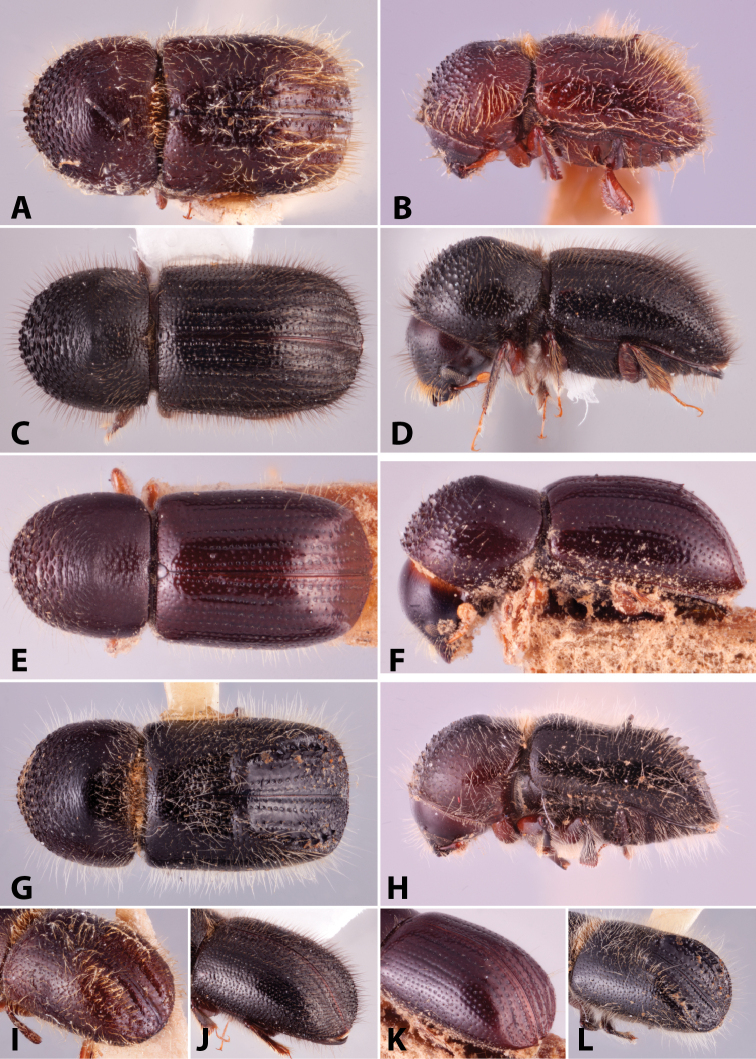
Dorsal, lateral and declivital view of *Anisandrus hera* holotype, 3.9 mm (**A, B, I**), *A. hirtus*, 3.4–4.5 mm (**C, D, J**), *A. improbus* holotype, 3.3–3.4 mm (**E, F, K**), and *A. klapperichi* 5.4–5.6 mm (**G, H, L**).

#### Anisandrus hirtus

Taxon classificationAnimaliaColeopteraCurculionidae

(Hagedorn, 1904)

Fig. 21C, D, J

Xyleborus hirtus Hagedorn, 1904: 126.
Cyclorhipidion hirtum (Hagedorn): Wood and Bright 1992: 700.
Anisandrus hirtus (Hagedorn): Hulcr et al. 2007: 578.
Xyleborus hagedorni Stebbing, 1914: 596 nec Iglesias 1914.
Xyleborus hirtuosus Beeson, 1930: 217. Synonymy: Wood 1989: 175.
Xyleborus hagedornianus Schedl, 1952d: 164. Unnecessary replacement name for hagedorni.
Xyleborus tectonae Nunberg, 1956: 209. Unnecessary replacement name for hagedorni.
Xyleborus hirtipes Schedl, 1969b: 53. syn. nov.
Xyleborus taiwanensis Browne, 1980b: 386. Synonymy: Beaver and Liu 2010: 22.

##### Type material.

***Holotype****Xyleborus hirtipes* (NHMW). ***Holotype****Xyleborus taiwanensis* (NHMUK)

##### New records.

China: Guangxi A. R., Longsheng hot spring, 25°53.6'N, 110°12.4'E, 360 m, forested river valley, wet rocks, M. Ficáček, J. Hájek, J. Růžička (MNHP, 2; RABC, 1). Jiangxi, Jinggang Shan Mts, Songmuping, 26°34.7'N, 114°04.3'E, 1280 m, stream valley, M. Ficáček, J. Hájek (MNHP, 1; RABC, 1). Sichuan, E’bian, 29.vi.1960, Fusheng Huang, ex Fagaceae (NMNH, 2). Tibet [Xizang], Dongqiong, Chayu, 16.vii.1973, Fusheng Huang; ex *Phoebe* or *Machilus* (NMNH, 1). Yunnan, Xishuangbanna, 20 km NW Jinghong, vic. Man Dian (NNNR), 22°07.80'N, 100°40.0'E, 730 m, forest, EK, 6.iv.2009, L. Meng (NKME, 1; RABC, 1). Vietnam: Cao Bang, 22°36.454'N, 105°52.083'E, 1661 m, 15.iv.2014, VN33, Cognato, Smith, Pham, ex branches from large tree fall (MSUC, 9; NHMUK, 2; NMNH, 2; VMNH, 2). Lao Cai, Hoang Lien N.P., 22.35, 103.77, 1500–2000 m, 19.v.2019, VN171, S.M. Smith, A.I. Cognato, ex dead sapling 1 cm DBH (MSUC, 4).

##### Diagnosis.

3.4–4.5 mm long (mean = 3.92 mm; n = 5); 1.95–2.53× as long as wide. This species is distinguished by the mesonotal mycangial tuft the length of the scutellum; elytral disc convex; declivity rounded, posterolateral margins rounded; declivity unarmed, surface opalescent to shagreened; declivital striae clearly impressed; and body densely covered by erect dark brown pubescence.

##### Similar species.

*Anisandrus ursulus*.

##### Distribution.

Bhutan, Cambodia, China (Fujian, Guangxi*, Jiangxi*, Sichuan*, Xizang*, Yunnan*), India (Meghalaya, West Bengal), Laos, Myanmar, Nepal, Taiwan, Thailand, Vietnam.

##### Host plants.

Polyphagous, recorded from five genera in five different families (Lamiaceae, Lauraceae, Magnoliaceae, Rutaceae, Symplocaceae) (Wood and Bright 1992; Beaver and Liu 2010).

##### Remarks.

The *Xyleborus hirtipes* holotype was examined and found to be conspecific to other specimens of *Anisandrus hirtus* and is here placed in synonymy.

#### Anisandrus improbus

Taxon classificationAnimaliaColeopteraCurculionidae

(Sampson, 1913)

Fig. 21E, F, K

Xyleborus improbus Sampson, 1913: 444.
Anisandrus improbus (Sampson): Hulcr et al. 2007: 578.

##### Type material.

***Holotype*** (NHMUK).

##### Diagnosis.

3.3–3.4 mm long (mean = 3.4 mm; n = 2); 2.43–2.54× as long as wide. This species is distinguished by the mesonotal mycangial tuft the length of the scutellum; elytral disc convex; declivity appearing flat when viewed laterally; declivital striae clearly impressed; declivital summit armed by a minute denticle on each interstriae 2 and 3; granules present on basal 1/2 of interstriae 2–4; declivital posterolateral margin costate to interstriae 7; declivital face strongly shiny; and declivital interstriae clearly punctate.

##### Similar species.

*Anisandrus eggersi*, *A. feronia*, *A. mussooriensis*.

##### Distribution.

China (Xizang), India (Assam, West Bengal).

##### Host plants.

Recorded from *Quercus* (Fagaceae), *Machilus* (Lauraceae), and *Eucalyptus* (Myrtaceae) (Maiti and Saha 2004).

#### Anisandrus klapperichi

Taxon classificationAnimaliaColeopteraCurculionidae

(Schedl, 1955) comb. nov.

Fig. 21G, H, L

Xyleborus klapperichi Schedl, 1955b: 46.
Cnestus klapperichi (Schedl): Wood and Bright 1992: 802.

##### Type material.

***Holotype*** (ZMFK). Not examined.

##### New records.

China: Fujian, Shaowu, Tachulan, 2.vi.1943, T. Maa (NMNH, 1); as previous except: 1000 m, 13.vi.1943 (NMNH, 1); as previous except: Chong’an, 1000 m, 8.v.1978, ex *Cinnamomum* sp. (NMNH, 2).

##### Diagnosis.

5.4–5.6 mm long (mean = 5.53 mm; n = 4); 2.12–2.24× as long as wide. This species is distinguished by the dense mesonotal mycangial tuft that extends laterally from the scutellum to striae 3; declivital posterolateral margin costate to interstriae 5; elytral disc with a deep transverse saddle-like depression; declivital summit with large incurved spine on interstriae 2; declivital interstriae 3 with six additional unequally sized incurved spines on basal 1/2; declivity strongly sulcate to interstriae 3; strial punctures large, seriate; interstriae impunctate, setose, setae semi-erect, short and thick; declivity shagreened, abundantly covered with long erect hair-like setae; and pronotal asperities small, coarse, densely spaced.

##### Similar species.

*Anisandrus auratipilus*, *A. hera*, *A. percristatus*, *A. venustus*, *A. xuannu*.

##### Distribution.

China (Fujian).

##### Host plants.

This species has only been reported from *Cinnamomum* (Lauraceae).

##### Remarks.

This species is transferred to *Anisandrus* because of the visible scutellum, pronotal base with a large, dense setal tuft (indicating a mesonotal mycangium), procoxae contiguous, antennal club type 1, taller than wide, and protibiae triangular.

#### Anisandrus lineatus

Taxon classificationAnimaliaColeopteraCurculionidae

(Eggers, 1930)

Fig. 22A, B, I

Xyleborus lineatus Eggers, 1930: 177.
Cyclorhipidion lineatum (Eggers): Maiti and Saha 2004: 114.
Anisandrus lineatus (Eggers): Beaver and Liu 2018: 538.
Xyleborus melancranis Beeson, 1930: 179. Synonymy: Saha and Maiti 1996: 822.

##### Type material.

***Holotype****Xyleborus lineatus* (FRI), paratype (NMNH, 1).

##### New records.

China: Sichuan, Leibo, 19.iv.1964, ex either *Acer* or *Carpinus* (NMNH, 1); as previous except: Chudian, E’mei Mountain, 8.v.1964, Fusheng Huang, ex Lauraceae (NMNH, 2). India: Uttarakhand, Darjeeling, Senchal range, 21.iv.1923, J.C.M. Gardner, ex *Alnus nepalensis* (NMNH, 1). Vietnam: Lao Cai, Hoang Lien N.P., 22.35, 103.77, 1500–2000 m, 20.v.2019, VN194, S.M. Smith, A.I. Cognato, ex dead sampling; 1 cm at base (MSUC, 2).

##### Diagnosis.

2.6–3.3 mm long (mean = 2.96 mm; n = 5); 2.2–2.6× as long as wide. This species is distinguished by the mesonotal mycangial tuft the length of the scutellum; elytral disc convex; declivity gradual and convex, with rounded posterolateral margins; pronotum conical frontally when viewed dorsally (type 0); pronotum armed by four coarse serrations on anterior margin (median pair larger than lateral pair); elytra smooth, strongly shiny; and declivital striae deeply impressed.

##### Similar species.

*Xylosandrus formosae*.

##### Distribution.

China* (Sichuan), India (Uttarakhand, West Bengal), Nepal, Vietnam*.

##### Host plants.

Recorded from *Machilus* (Lauraceae), *Symplocos* (Symplocaceae) (Beeson 1930) and *Alnus* (Betulaceae).

**Figure 22.**
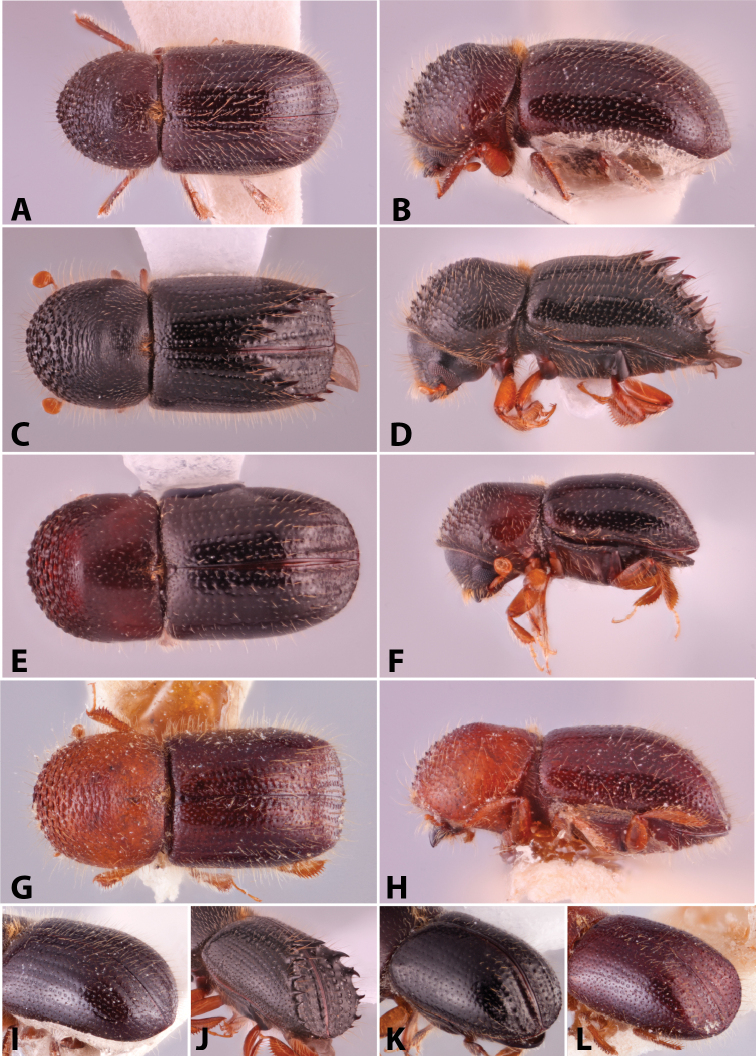
Dorsal, lateral and declivital view of *Anisandrus lineatus*, 2.6–3.3 mm (**A, B, I**), *A. longidens*, 3.0 mm (**C, D, J**), *A. maiche*, 2.2–2.5 mm (**E, F, K**), and *A. mussooriensis* paratype, 3.0–3.25 mm (**G, H, L**).

#### Anisandrus longidens

Taxon classificationAnimaliaColeopteraCurculionidae

(Eggers, 1930)

Fig. 22C, D, J

Xyleborus longidens Eggers, 1930: 181.
Anisandrus longidens (Eggers): Hulcr et al. 2007: 578.

##### Type material.

***Holotype*** (FRI), ***paratype*** (NHMW, 1).

##### New records.

Vietnam: Lao Cai, Hoang Lien N.P., 22.35, 103.77, 1500–2000 m, 20.v.2019, VN185, S.M. Smith, A.I. Cognato, ex 1–2 cm branch (MSUC, 1); as previous except: 19–20.v.2019, ex FIT (MSUC, 2; NMNH, 1).

##### Diagnosis.

3.0–3.2 mm long (mean = 3.1 mm; n = 2); 2.5–2.83× as long as wide. This species is distinguished by the mesonotal mycangial tuft the length of the scutellum; convex elytral disc; declivity weakly bisulcate, margins ornamented by large sharp spines on interstriae 2–7, spine on interstriae 3 the largest; posterolateral margin costate to interstriae 5; and declivital interstriae impunctate.

##### Similar species.

*Anisandrus feronia*.

##### Distribution.

India (Meghalaya), Vietnam*.

##### Host plants.

Unknown.

#### Anisandrus maiche

Taxon classificationAnimaliaColeopteraCurculionidae

(Kurentzov, 1941)

Fig. 22E, F, K

Xyleborus maiche Kurentzov, 1941: 192.
Anisandrus maiche (Kurentzov): Nikulina et al. 2015: 43.
Anisandrus maiche Stark, 1936: 142 [*sic*]. Hulcr et al. 2007: 578.
Xyleborus maiche Eggers, 1942: 36. Homonym. Synonymy: Pfeffer 1944: 131.

##### Type material.

***Syntypes*** (ZIN). Not examined.

##### New records.

China: Shanghai, Dongchuan, vii–viii.2017, Lei Gao, ex trap w/ querciverol (MSUC, 4). Japan: Honshu, Saitama, Chichibu, Takikawa Catchm., 35°55'N, 138°49'E, 850–1060 m, 6.viii.2013 (RABC, 1).

##### Diagnosis.

2.2–2.5 mm long (mean = 2.3 mm; n = 5); 2.3–2.78× as long as wide. This species is distinguished by the mesonotal mycangial tuft the length of the scutellum; declivital interstriae 1–4 uniseriate denticulate; discal interstriae punctures uniseriate; declivity appearing bisulcate with impressed from striae 1 to interstriae 2, interstriae 3 distinctly raised; declivital punctures small, uniseriate; shiny appearance; and small body size.

##### Similar species.

*Anisandrus dispar*, *A. paragogus*, *Xylosandrus germanus*.

##### Distribution.

China (Heilongjiang, Shanghai*), Japan*, South & North Korea, Russia (European (introduced), Far East), Ukraine. Introduced to USA (Rabaglia et al. 2009; Gomez et al. 2018a).

##### Host plants.

Polyphagous, recorded from eight families of trees (Rabaglia et al. 2009).

##### Remarks.

Kurentzov (1941) and Terekhova and Skrylnik (2012) provide information on the biology and gallery system, which are similar to *A. dispar* (see above).

Preliminary phylogenies suggest that *Anisandrus maiche* is sister to *Xylosandrus* (Cognato et al. 2020b). Kurenzov (1941) provided the first valid description of *Xyleborus maiche* rather than Eggers (1942), which has been widely and incorrectly cited in the literature (Nikulina et al. 2015).

#### Anisandrus mussooriensis

Taxon classificationAnimaliaColeopteraCurculionidae

(Eggers, 1930)

Fig. 22G, H, L

Xyleborus mussooriensis Eggers, 1930: 179.
Cyclorhipidion mussooriense (Eggers): Maiti and Saha 2004: 116.
Anisandrus mussooriensis (Eggers): Beaver and Liu 2018: 538.

##### Type material.

***Holotype*** (FRI), ***cotype*** (NMNH, 1).

##### Diagnosis.

3.0–3.25 mm long (mean = 3.1 mm; n = 5); 2.3–2.33× as long as wide. This species is distinguished by the mesonotal mycangial tuft the length of the scutellum; elytral disc flat; declivital interstriae clearly punctate; declivital posterolateral margin carinate to interstriae 5; declivity appearing flat when viewed laterally, weakly depressed below lateral margins; and basal 1/2 of declivital interstriae 2 with two or three small tubercles.

##### Similar species.

*Anisandrus eggersi*, *A. feronia*, *A. improbus*.

##### Distribution.

India (Uttarakhand), Nepal.

##### Host plants.

Recorded only from *Berberis* (Berberidaceae) (Beeson 1930).

#### Anisandrus niger

Taxon classificationAnimaliaColeopteraCurculionidae

(Sampson)

Fig. 23A, B, I

Xyleborus niger Sampson, 1912: 247.
Anisandrus niger (Sampson): Beaver and Liu 2018: 538.

##### Type material.

***Holotype*** (NHMUK).

##### New records.

Laos: NE, Houa Phan, Ban Saluei, Phou Pan Mt, 20°12–13.5'N, 103°59.5–104°01'E, 1340–1780 m, 15.iv.–15.v.2008, Lao collectors (RABC, 1); as previous except: 20°12'N, 104°01'E, 1300–1900 m, 7.iv.–25.v.2010, C. Holzschuh (RABC, 1). Vietnam: Cao Bang, 22°36.3'N, 105°52.6'E, 1435–1601 m, 13–17.iv.2014, VN16, Cognato, Smith, Pham, FIT (MSUC, 1).

##### Diagnosis.

5.8–5.9 mm long (mean = 5.87 mm; n = 3); 2.0–2.19× as long as wide. This species is distinguished by its large size, mesonotal mycangial tuft the length of the scutellum; elytral disc convex; declivital interstriae impunctate; elytral surface smooth, shiny to weakly shagreened; declivital face flattened when viewed laterally; declivity appearing weakly bisulcate; declivital interstriae 2 weakly impressed, declivital interstriae 1 and 3 tuberculate to apex, interstriae 2 with a tubercle at summit and three or four irregularly spaced granules along its length; and declivital posterolateral margin rounded.

##### Similar species.

*Anisandrus apicalis*, *A. congruens*, *A. cristatus*, *A. geminatus*, *A. sinivali*.

##### Distribution.

Laos*, Myanmar, Nepal, Vietnam*.

##### Host plants.

Unknown.

**Figure 23.**
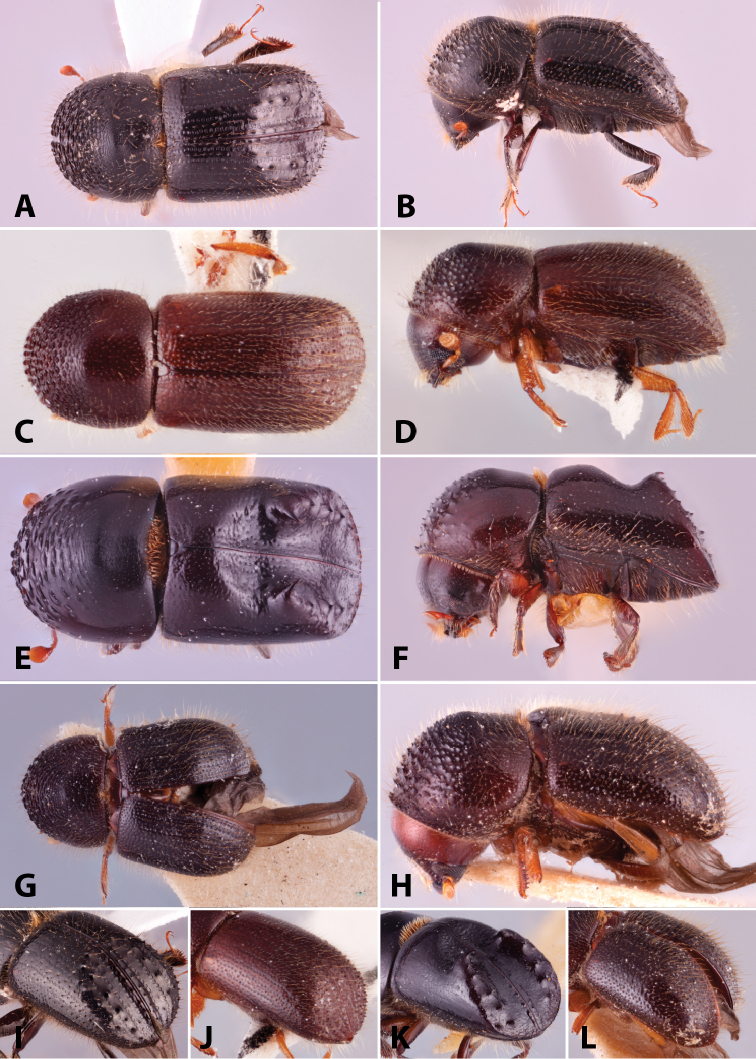
Dorsal, lateral and declivital view of *Anisandrus niger*, 5.8–5.9 mm (**A, B, I**), *A. paragogus* holotype, 2.8 mm (**C, D, J**), *A. percristatus*, 5.5 mm (**E, F, K**), and *A. sinivali* holotype, 3.9 mm (**G, H, L**).

#### Anisandrus paragogus sp. nov.

Taxon classificationAnimaliaColeopteraCurculionidae

http://zoobank.org/CB28B458-4610-45AB-9C89-1EFA27E88EBF

Fig. 23C, D, J

##### Type material.

***Holotype***, female, 西藏 73084 察隅洞穷1973.7.15 桢楠 采集者 : 黄复生 [China: Tibet [Xizang], Dongqiong, Chayu, 15.vii.1973, Fusheng Huang, ex *Machilus* sp.] (NMNH).

##### Diagnosis.

2.8 mm long (n = 1); 2.55× as long as wide. This species is distinguished by the mesonotal mycangial tuft absent; declivital interstriae uniseriate granulate on basal 1/2; declivital face opalescent; declivital interstrial setae erect, 3× width of an interstria; and a row of serrations on anterior margin of pronotum.

##### Similar species.

*Anisandrus dispar*, *A. maiche*, *Xylosandrus germanus*.

##### Description

**(female).** 2.8 mm long (n = 1); 2.55× as long as wide. Body brown. Legs and antennae light brown. ***Head***: epistoma entire, transverse, with a row of hair-like setae. Frons weakly convex to upper level of eyes, subshiny, punctate; punctures large, shallow, moderately dense; punctures bearing a long, erect hair-like seta. Eyes shallowly emarginate just above antennal insertion, upper part smaller than lower part. Submentum large, distinctly triangular, slightly impressed. Antennal scape regularly thick, shorter than club. Pedicel as wide as scape, shorter than funicle. Funicle 4-segmented, segment 1 shorter than pedicel. Club much longer than wide, obliquely truncate, type 1; segment 1 corneous, encircling anterior face; segment 2 narrow, concave, corneous on anterior face only; sutures absent on posterior face. ***Pronotum***: 0.73× as long as wide. In dorsal view rounded, type 1, sides convex, rounded anteriorly; anterior margin with a row of seven very large serrations. In lateral view robust and rounded, type 5, disc longer than anterior slope, summit at apical 2/5. Anterior slope with densely spaced, large coarse asperities, becoming lower and more strongly transverse towards summit. Disc subshiny with dense, small, fine punctures bearing short erect hair-like setae, some longer hair-like setae at margins. Lateral margins obliquely costate. Base transverse, posterior angles broadly rounded. Mycangial tuft absent. ***Elytra***: 1.6× as long as wide, 2.2× as long as pronotum. Scutellum broad, large, linguiform, flush with elytra, flat, shiny. Elytral base transverse, edge oblique, humeral angles rounded, parallel-sided in basal 3/4, then broadly rounded to apex. Disc flat, opalescent, striae not impressed, with small, shallow punctures separated by 1–2 diameters of a puncture, setose, setae short, in-curved, hair-like; interstriae flat, punctate, punctures strongly confused, setose, setae long, erect hair-like, unarmed by granules. Declivity occupying approximately 1/3 of elytra, steeply rounded, declivital face convex, opalescent; striae distinctly impressed, strial punctures much larger and deeper than those of disc; interstriae impunctate, granulate, granules widely and regularly spaced from base to apex, granules setose, setae 3× width of interstriae 2, erect, hair-like, interstriae weakly laterally broadened from declivital summit to midpoint then narrowed to apex. Posterolateral margin costate, granulate to interstriae 7. ***Legs***: procoxae narrowly separated. Protibiae obliquely triangular, broadest at apical 1/3; posterior face smooth; apical 1/2 of outer margin with five large socketed denticles, their length longer than basal width. Meso- and metatibiae flattened; outer margins evenly rounded with nine and ten moderate socketed denticles, respectively.

##### Etymology.

G. *paragogos* = misleading. In reference to its resemblance to *Ambrosiophilus*.

##### Distribution.

China (Xizang).

##### Host plants.

Recorded only from *Machilus* (Lauraceae).

##### Remarks.

Locality labels on the holotype are in Chinese and were translated by You Li. An English locality label has been placed on the specimen below the original locality labels.

#### Anisandrus percristatus

Taxon classificationAnimaliaColeopteraCurculionidae

(Eggers, 1939) comb. nov.

Fig. 23E, F, K

Xyleborus percristatus Eggers, 1939a: 12.

##### Type material.

***Paratype*** (NMNH, 1).

##### New records.

China: Sichuan, E’bian, 1900 m, 2.vi.1960, Huifen Yin, ex *Schima superba* (NMNH, 1).

##### Diagnosis.

5.5 mm long (mean = 5.5 mm; n = 3); 2.12–2.2× as long as wide. This species is distinguished by the dense mesonotal mycangial tuft that extends laterally from the scutellum to striae 3; declivital posterolateral margin carinate to interstriae 5; elytral disc with a profound transverse saddle-like depression; declivital base with very large incurved spine on interstriae 3, interstriae 3 with four additional equally sized and spaced denticles; declivity broadly sulcate to interstriae 5; elytral disc sulcate anteriad to spine on interstriae 3; large body size; body shiny, appearing polished, largely glabrous, minutely punctate; declivital punctures confused; and pronotal asperities very broad, fine, widely spaced.

##### Similar species.

*Anisandrus auratipilus*, *A. hera*, *A. klapperichi*, *A. venustus*, *A. xuannu*.

##### Distribution.

China (Sichuan, Yunnan), Myanmar.

##### Host plants.

Recorded from *Schima superba* (Theaceae).

##### Remarks.

This species is transferred to *Anisandrus* because of the visible scutellum, pronotal base with a large, dense setal tuft (indicating a mesonotal mycangium), contiguous procoxae; antennal club type 1, taller than wide, and protibiae triangular.

#### Anisandrus sinivali sp. nov.

Taxon classificationAnimaliaColeopteraCurculionidae

http://zoobank.org/FEDF5FDF-F95F-4201-A6FC-4BF2707A5FEC

Fig. 23G, H, L

##### Type material.

***Holotype***, female, India: Bengal [West Bengal], Kalimpong, Samsingh, 7.x.1933, C.F.C. Beeson (NMNH).

##### Diagnosis.

3.9 mm long (n = 1); 2.29× as long as wide. This species is distinguished by the mesonotal mycangial tuft the length of the scutellum; elytral disc with a weak transverse saddle-like depression; declivity posterolateral margins rounded; elytral surface opalescent; declivital interstriae 2 armed with a blunt tubercle at summit, interstriae 3 armed by one or two denticles near declivital summit ventrad to tubercle on interstriae 2; declivital face convex, evenly rounded toward apex; and pronotal disc feebly asperate.

##### Similar species.

*Anisandrus apicalis*, *A. congruens*, *A. cristatus*, *A. geminatus*, *A. niger*.

##### Description

**(female).** 3.9 mm long (n = 1); 2.29× as long as wide. Body dark brown. Legs and antennae light brown. ***Head***: epistoma entire, transverse, with a row of hair-like setae. Frons weakly convex to upper level of eyes, finely reticulate, sparsely finely punctate; punctures bearing a long, erect hair-like seta. Eyes shallowly emarginate just above antennal insertion, upper part smaller than lower part. Submentum large, distinctly triangular, slightly impressed Antennal scape regularly thick, longer than club. Pedicel as wide as scape, shorter than funicle. Funicle 4-segmented, segment 1 longer than pedicel. Club longer than wide, obliquely truncate, type 1; segment 1 corneous, encircling anterior face; segment 2 narrow, concave, corneous on anterior face only; sutures absent on posterior face. ***Pronotum***: 0.86× as long as wide. In dorsal view rounded, type 1, sides convex, rounded anteriorly; anterior margin with a row of five large serrations. In lateral view type 3, short and tall, disc as long as anterior slope, summit at midpoint. Anterior slope with densely spaced, large coarse asperities, becoming lower and more strongly transverse towards summit. Disc subshiny, impunctate, feebly asperate, basal and lateral areas densely finely punctate, each puncture bearing moderate, erect, hair-like setae, some longer hair-like setae at margins. Lateral margins obliquely costate. Base transverse, posterior angles broadly rounded. Mycangial tuft present along basal margin, tuft densely setose, approximately the width of scutellum. ***Elytra***: 1.06× as long as wide, 1.24× as long as pronotum. Scutellum broad, large, linguiform, flush with elytra, flat, shiny. Elytral base transverse, edge oblique, humeral angles rounded, parallel-sided in basal 1/2, then broadly rounded to apex; surface opalescent. Disc with a weak medial transverse saddle-like depression, striae not impressed, with small, shallow punctures separated by less than one diameter of a puncture, setose, setae as long as two punctures, recumbent, hair-like; interstriae flat, punctate, punctures strongly confused, setose, setae 2–3× width of interstriae 2, erect hair-like, unarmed by granules. Declivity occupying approximately 1/2 elytra, evenly rounded, declivital face convex; striae weakly impressed, strial punctures somewhat larger and deeper than those of disc; interstriae sparsely uniseriate punctate, setae 2–4× width of interstriae 2, erect, hair-like, interstriae 2 narrower than interstriae 3 at midpoint of declivity, declivital interstriae 2 armed with a blunt tubercle at summit, interstriae 3 armed by one or two denticles near declivital summit ventrad to tubercle on interstriae 2. Posterolateral margin rounded, unarmed by granules. ***Legs***: procoxae contiguous. Protibiae obliquely triangular, broadest at apical 1/3; posterior face smooth; apical 1/2 of outer margin with seven very large socketed denticles, their length much longer than basal width. Meso- and metatibiae flattened; outer margins evenly rounded with at least five and seven large socketed denticles, respectively.

##### Etymology.

Hindu mythology, Sinivali – goddess of fecundity. Pronunciation – *Sinivālī*. Noun in apposition.

##### Distribution.

India (West Bengal).

##### Host plants.

Unknown.

##### Remarks.

The holotype is card mounted obscuring ventral characters, including mesotibial denticles.

#### Anisandrus ursulus

Taxon classificationAnimaliaColeopteraCurculionidae

(Eggers, 1923)

Fig. 24A, B, G

Xyleborus ursulus Eggers, 1923: 173.
Xylosandrus ursulus (Eggers): Wood and Bright 1992: 801.
Anisandrus ursulus (Eggers): Dole and Cognato 2010: 527.

##### Type material.

***Holotype*** (SDEI). Not examined.

##### New records.

China: Guangdong, W of Qixing, Heishiding nature reserve, 27°27.9'N, 111°54.3'E, 190 m, forested stream valley, at light, 1–3.v.2011, M. Ficáček, J. Hájek (MNHP, 1). Guangxi A. R., Longsheng hot spring, 25°53.6'N, 110°12.4'E, 360 m, forested river valley, wet rocks, M. Ficáček, J. Hájek, J. Růžička (MNHP, 1). Jiangxi, Long Nan, 12.vii.2016, Lv-Jia, Lai, S-C., ex *Cyclobalanopsis glauca* (RABC, 1).

##### Diagnosis.

4.3–4.9 mm long (mean = 4.5 mm; n = 5); 1.88–1.96× as long as wide. This species is distinguished by the mesonotal mycangial tuft the length of the scutellum; elytral disc convex; declivity obliquely truncate with lateral margins obliquely costate; declivity opalescent and unarmed; declivital striae not impressed; body stout and densely covered by erect dark brown pubescence.

Similar to *Cnestus mutilatus* and *Hadrodemius* species but declivity less steeply truncate, with posterolateral margins rounded, never carinate, procoxae contiguous and the mesonotal mycangial tuft the length of the scutellum.

##### Similar species.

*Anisandrus hirtus*, *Cnestus ater*, *C. mutilatus*, *Hadrodemius* spp.

##### Distribution.

China (Fujian, Guangdong*, Guangxi*, Jiangxi*), India (Nicobar Is, West Bengal), Indonesia (Bali, Batoe Is, Java, Maluku, Sulawesi, Sumatra), Laos, East & West Malaysia, New Guinea, Philippines, Solomon Islands, Thailand, Vietnam.

##### Host plants.

The species is polyphagous (Browne 1961b).

**Figure 24.**
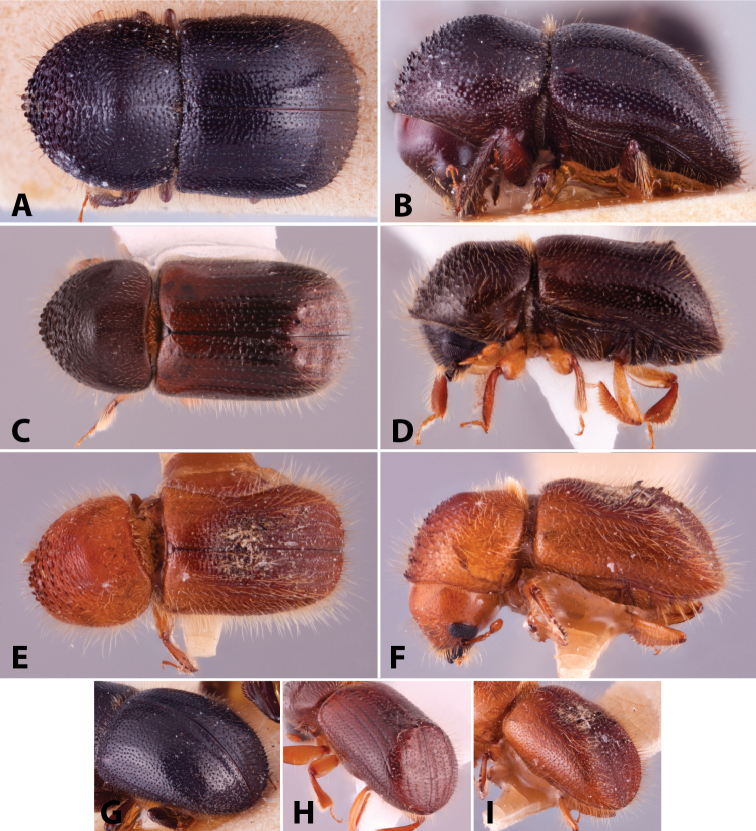
Dorsal, lateral and declivital view of *Anisandrus ursulus*, 4.3–4.9 mm (**A, B, G**), *A. venustus* holotype, 3.1 mm (**C, D, H**), and *A. xuannu* holotype, 4.0–4.15 mm (**E, F, I**).

#### Anisandrus venustus sp. nov.

Taxon classificationAnimaliaColeopteraCurculionidae

http://zoobank.org/719C65BA-BB8D-4343-B729-2F4ED776C0AD

Fig. 24C, D, H

##### Type material.

***Holotype***, female, Taiwan: Taichung, Heping Dist., 2.iv.2014, C.-S. Lin (TARI). ***Paratypes***, female, as holotype (MSUC, 2; NHMUK, 1; NMNH, 1); Yilan Co., Chilan cypress forest trail, 12.5K, EtOH+pinene, 22.xii.2018, Liu, Lan-Yu (LLYC, 2; RABC, 1).

##### Diagnosis.

3.1 mm long (mean = 3.1 mm; n = 4); 2.38× as long as wide. This species is distinguished by the dense mesonotal mycangial tuft that extends laterally from the scutellum to striae 3; declivital posterolateral margin rounded; elytral disc with a broad, weak transverse saddle-like depression; declivital summit with a small denticle on interstriae 2 and a minute denticle on interstriae 1, interstriae 3 unarmed; declivital strial punctures large, seriate, each bearing a recumbent seta, interstriae flat, minutely punctate, punctures strongly confused, setose, setae hair-like, erect; declivity opalescent; elytral disc shiny and finely punctate; body abundantly covered with long erect hair-like setae; and pronotal asperities large, coarse, moderately spaced.

##### Similar species.

*Anisandrus apicalis*, *A. auratipilus*, *A. hera*, *A. klapperichi*, *A. percristatus*, *A. xuannu*.

##### Description

**(female).** 3.1 mm long (mean = 3.1 mm; n = 4); 2.38× as long as wide. Body bicolored with pronotal and elytral bases lighter than rest of body. Pronotal and elytral bases brown, remainder of elytra and head dark brown. Legs and antennae light brown. ***Head***: epistoma entire, transverse, with a row of hair-like setae. Frons weakly convex to upper level of eyes, impunctate, median area with a broad diamond-shaped smooth, glabrous, strongly shiny area; lateral areas shagreened, weakly rugose, setose; each shallow ruga bearing a long, erect hair-like seta. Eyes shallowly emarginate just above antennal insertion, upper part smaller than lower part. Submentum large, distinctly triangular, slightly impressed. Antennal scape regularly thick, shorter than length of club. Pedicel as wide as scape, shorter than funicle. Funicle 4-segmented, segment 1 shorter than pedicel. Club longer than wide, obliquely truncate, type 1; segment 1 corneous, encircling anterior face; segment 2 narrow, concave, corneous on anterior face only; sutures absent on posterior face. ***Pronotum***: 0.64× as long as wide. In dorsal view conical, type 0, sides convex, conical anteriorly; anterior margin with a row of 6–8 moderate serrations. In lateral view short and tall, type 3, disc as long as anterior slope, summit at midpoint. Anterior slope with moderately spaced, large, coarse asperities, becoming lower and more strongly transverse towards summit. Disc subshiny with dense, large, fine punctures bearing short to moderate, erect hair-like setae, some longer hair-like setae at margins. Lateral margins obliquely costate. Base transverse, posterior angles broadly rounded. Mycangial tuft present along basal margin tuft broad, densely setose, laterally extending to elytral striae 3. ***Elytra***: 1.44× as long as wide, 2.23× as long as pronotum. Scutellum broad, large, linguiform, flush with elytra, flat, shiny. Elytral base transverse, edge oblique, humeral angles rounded, parallel-sided in basal 3/4, then narrowly rounded to apex; surface shiny. Disc shiny, with a broad, weak transverse saddle-like depression behind declivital summit, striae not impressed, with moderate, shallow punctures separated by 1–2 diameters of a puncture, setose, setae as long as a puncture, recumbent, hair-like; interstriae flat, minutely punctate, punctures strongly confused, setose, setae 1–1.5× width of interstriae 2, erect hair-like, unarmed by granules. Declivity occupying approximately 1/3 of elytra, steeply rounded, declivital face flattened, opalescent; striae not impressed, strial punctures much larger and deeper than those of disc, and bearing setae 2× as long as those of disc; interstriae densely minutely punctate, punctures strongly confuses, setose, setae 1–1.5× width of interstriae 2, erect, hair-like, interstriae 2 as wide as interstriae 3 at midpoint of declivity, declivital summit with a small denticle on interstriae 2 and a minute denticle on interstriae 1, interstriae 3 unarmed. Posterolateral margin rounded, unarmed. ***Legs***: procoxae contiguous; prosternal coxal piece short, inconspicuous. Protibiae obliquely triangular, broadest at apical 1/3; posterior face smooth; apical 1/2 of outer margin with six large socketed denticles, their length longer than basal width. Meso- and metatibiae flattened; outer margins evenly rounded with seven large, narrow socketed denticles.

##### Etymology.

L. *venustus* = like Venus, lovely, beautiful, elegant, graceful. An adjective.

##### Distribution.

Taiwan.

##### Host plants.

Unknown.

#### Anisandrus xuannu sp. nov.

Taxon classificationAnimaliaColeopteraCurculionidae

http://zoobank.org/4FFF4E2C-330F-4A01-9EC2-5496FC0A5B73

Fig. 24E, F, I

##### Type material.

***Holotype***, female, 四川 : 峨眉山 洪椿坪295 1964-V-12 采集者 : 黄复生 [China: Sichuan, Hongchunping, Emeishan Mt., 12.v.1964, Fusheng Huang, ex Fagaceae] (NMNH). ***Paratypes***, female, China: Chongqing, Simian Shan, 7.v.2016, Tian-Shang, Lv-Jia (RABC, 1); Sichuan, Mt. Emei, 600–1050 m, 5–19.v.1989, L. Bocák (RABC, 1).

##### Diagnosis.

4.0–4.15 mm long (mean = 4.08 mm; n = 3); 2.0–2.31× as long as wide. This species is distinguished by the dense mesonotal mycangial tuft that extends laterally from the scutellum to striae 3; declivital posterolateral margin costate to interstriae 5; elytral disc with a deep transverse saddle-like depression, depressed area sulcate; declivital summit with large incurved spine on interstriae 2, interstriae 3 unarmed; declivity moderately sulcate to interstriae 4; declivital strial punctures large, seriate, interstriae minutely biseriately punctate, setose, setae short erect bristle-like; moderate body size; declivity shagreened; elytral disc rugose; body abundantly covered with long erect hair-like setae; and pronotal asperities small, coarse, densely spaced.

##### Similar species.

*Anisandrus auratipilus*, *A. hera*, *A. klapperichi*, *A. percristatus*, *A. venustus*.

##### Description

**(female).** 4.0–4.15 mm long (mean = 4.08 mm; n = 3); 2.0–2.31× as long as wide. Body bicolored with pronotal and elytral bases lighter than rest of body. Pronotal and elytral bases, head, legs, and antennae light brown, remainder of elytra red-brown. ***Head***: epistoma entire, transverse, with a row of hair-like setae. Epistoma entire, transverse, with a row of hair-like setae. Frons weakly convex to upper level of eyes, impunctate, shagreened, weakly rugose, setose; each shallow ruga bearing a long, erect hair-like seta. Eyes shallowly emarginate just above antennal insertion, upper part smaller than lower part. Submentum large, distinctly triangular, slightly impressed. Antennal scape regularly thick, as long as club. Pedicel as wide as scape, shorter than funicle. Funicle 4-segmented, segment 1 as long as pedicel. Club longer than wide, obliquely truncate, type 1; segment 1 corneous, encircling anterior face; segment 2 narrow, concave, corneous on anterior face only; sutures absent on posterior face. ***Pronotum***: 0.78× as long as wide. In dorsal view rounded, type 1, sides convex, rounded anteriorly; anterior margin with a row of six very large serrations. In lateral view type 3, short and tall, disc as long as anterior slope, summit at midpoint. Anterior slope with densely spaced, large coarse asperities, becoming lower and more strongly transverse towards summit. Disc subshiny, median area impunctate, reticulate, lateral areas with dense, small, shallow punctures bearing moderate, erect hair-like setae, some longer hair-like setae at margins. Lateral margins obliquely costate. Base transverse, posterior angles broadly rounded. Mycangial tuft present along basal margin tuft broad, densely setose, laterally extending to elytral striae 3. ***Elytra***: 1.45× as long as wide, 1.86× as long as pronotum. Scutellum broad, large, linguiform, flush with elytra, flat, shiny. Elytral base transverse, edge oblique, humeral angles rounded, parallel-sided in basal 1/2, then broadly rounded to apex. Disc rugose, shiny, with a deep transverse saddle-like depression just behind declivital summit, depressed area sulcate; striae not impressed, with small, shallow punctures separated by two diameters of a puncture, setose, setae as long as a puncture, recumbent, hair-like; interstriae flat, punctate, punctures strongly confused, setose, setae 1× width of interstriae 2, erect hair-like, unarmed by granules. Declivity occupying approximately 1/2 elytra, evenly rounded, declivital face nearly flat, moderately sulcate to interstriae 4, shagreened; striae not impressed, strial punctures much larger and deeper than those of disc, and bearing setae as described for disc; interstriae minutely biseriately punctate, setose, setae short, erect, bristle-like, interstriae 2 as broad as interstriae 3 at midpoint of declivity, declivital summit with large incurved spine on interstriae 2, interstriae 3 unarmed; lateral margins of declivity densely setose with very long, erect hair-like setae 2–4× width of interstriae 2. Posterolateral margin costate to interstriae 5. ***Legs***: procoxae contiguous. Protibiae obliquely triangular, broadest at apical 1/3; posterior face smooth; apical 1/2 of outer margin with six large socketed denticles, their length longer than basal width. Meso- and metatibiae flattened; outer margins evenly rounded with at least eight large socketed denticles.

##### Etymology.

Chinese mythology, Xuannü “mysterious lady”- the goddess of fertility. Noun in apposition.

##### Distribution.

China (Chongqing, Sichuan).

##### Host plants.

Recorded from Fagaceae.

##### Remarks.

The holotype is point mounted with an excessive amount of opaque glue which obscures the examination of ventral characters. Locality labels on the holotype are in Chinese and were translated by You Li. An English locality label has been placed on the specimen below the original locality labels.

### *Arixyleborus* Hopkins, 1915

#### Arixyleborus

Taxon classificationAnimaliaColeopteraCurculionidae

Hopkins, 1915

Arixyleborus Hopkins, 1915a: 59.
Xyleboricus Eggers, 1923: 212. Synonymy: Schedl 1952d: 162.

##### Type species.

*Arixyleborus rugosipes* Hopkins, 1915a; original designation.

##### Diagnosis.

1.35–5.2 mm, 2.0–3.5× as long as wide. *Arixyleborus* is distinguished by the elytra with distinctive deep strial furrows and interstrial ridges, ridges either granulate or carinate (three species without). *Arixyleborus* can be further diagnosed by the obliquely truncate antennal club with segment 1 almost covering the posterior face (type 2), club wider than long or as long as wide; protibiae slender or evenly rounded, posterior face flat and unarmed or inflated and granulate; scutellum variable either flush with elytra and flat, flush with elytra and medially impressed or flat and depressed below elytra; elytra from dorsal view typically angulate apically, rarely rounded; mycangial tufts absent; and procoxae contiguous.

*Arixyleborus* is similar to *Stictodex* with which it shares a broad antennal club but which lacks the distinctive elytral ridges and furrows. In addition, *Arixyleborus* has declivital striae 1 parallel to the suture while in *Stictodex* they are not parallel but undulating.

##### Similar genera.

*Cnestus*, *Pseudowebbia*, *Stictodex*, *Truncaudum*, *Webbia*.

##### Distribution.

Distributed throughout tropical Asia and Oceania.

##### Gallery system.

An unbranched radial or curved entrance tunnel, sometimes with a few branches. As the larvae develop, their feeding activity extends part of the main gallery into a single longitudinal brood chamber usually approximately rectangular in shape, and the width of the main gallery (Browne 1961b).

#### Key to *Arixyleborus* species (females only)

**Table:** 

1	Posterior face of protibiae inflated and granulate; scutellum flush with elytra and flat; lateral margin of pronotum costate or carinate	**2**
–	Posterior face of protibiae flat and unarmed; scutellum flush with elytra and medially impressed or depressed below level of elytra; lateral margin of pronotum oblique	**15**
2	Declivital posterolateral carina forming a circumdeclivital ring; lateral profile of declivity appearing truncate; pronotum from dorsal view type 8, with disc very long compared to anterior slope	*** resecans ***
–	Declivital posterolateral costa extending to interstriae 7; lateral profile of declivity appearing rounded or obliquely truncate; pronotum from dorsal view type 7, with disc as long or slightly longer than anterior slope	**3**
3	Anterior margin of pronotum viewed from above slightly angularly projecting, the asperities on the margin distinctly larger than those on the anterior slope, and separated from them by the height of a serration or more (Fig. 25C)	**4**
–	Anterior margin of pronotum viewed from above evenly rounded, the asperities on the anterior margin not distinctly larger than those on the anterior slope, and separated from them by the less than the height of a serration (Fig. 27E)	**8**
4	Smaller, 1.35–1.5 mm; dorsal profile of elytral apex rounded; elytral posterolateral costa denticulate	*** tuberculatus ***
–	Larger, 1.9–3.5 mm; dorsal profile of elytral apex angulate; elytral posterolateral costa carinate and unarmed	**5**
5	Larger, 3.2–3.5 mm; pronotal disc rugose; lateral margin of pronotum carinate	*** grandis ***
–	Smaller, 1.9–2.2 mm; pronotal disc punctate; lateral margin of pronotum costate	**6**
6	Declivital face without strial furrows and interstrial ridges below	*** leprosulus ***
–	Declivital face with strial furrows and interstrial ridges at least to midpoint	**7**
7	Declivital strial furrows at least 1.5× the width of interstrial ridges on disc; interstrial ridges denticulate, setose, setae recumbent, hair-like, as long as striae 2 with at declivital base; striae strongly impressed; declivity weakly shagreened, interstrial ridges almost appear shiny (Fig. 26E)	*** malayensis ***
–	Declivital strial furrows equal in width to interstrial ridges on disc; interstrial ridges finely tuberculate, glabrous or with minute setae no longer than 1/2 width of a strial furrow; striae moderately impressed; declivity strongly shagreened (Fig. 30E)	*** yakushimanus ***
8	Posterolateral declivital costa carinate and unarmed	**9**
–	Posterolateral declivital costa acute or not, armed with granules or denticles	**10**
9	Declivity with odd interstriae more strongly elevated than even interstriae; declivital interstriae minutely and equally denticulate	*** minor ***
–	Declivital interstriae 1 strongly elevated on apical 1/2, other interstriae similarly elevated; declivital interstriae 1 denticulate, denticles very large, denticles on remaining interstriae greatly reduced and less abundant	*** suturalis ***
10	Elytral strial furrows and interstrial ridges of striae and interstriae 1–3 anteriorly extending no further than apical 1/3 of disc (Fig. 26L)	**11**
–	Elytral strial furrows and interstrial ridges of striae and interstriae 1–3 anteriorly extending at least to midpoint of disc (Fig. 27K)	**12**
11	More elongate form, 2.9–3.3× as long as wide; more elongate pronotum (1.3 × longer than wide; declivity with short coarse setae	*** mediosectus ***
–	Less elongate form, 2.6–2.7× as long as wide; less elongate pronotum (1.1–1.2× longer than wide; declivity with fine hair-like setae	***silvanus* sp. nov.**
12	Elytral strial furrows and interstrial ridges anteriorly extending to apical 1/4 of disc; interstriae densely setose with long hair-like setae and bristles	*** rugosipes ***
–	Elytral strial furrows and interstrial ridges anteriorly extending just beyond the midpoint of disc; interstriae lightly setose, nearly glabrous	**13**
13	Declivity interstriae 1–3 strongly and uniformly convex from base to apex	*** nudulus ***
–	Declivity interstriae 1–3 feebly convex, convexity variably decreasing from base to apex	**14**
14	Antennal club as wide as long; larger 2.2 mm; elytra 1.35× longer than pronotum	***phiaoacensis* sp. nov.**
–	Antennal club wider than long; smaller, 2.0 mm; elytra 1.24× longer than pronotum	***crassior* sp. nov.**
15	Elytral disc with a transverse saddle-like depression (Fig. 30B)	**16**
–	Elytral disc flat, without a transverse saddle-like depression (Fig. 29F)	**17**
16	Larger, 5.2 mm; scutellum depressed below level of elytra and flat	***titanus* sp. nov.**
–	Smaller, 2.8–3.0 mm; scutellum flush with elytra and medially impressed	*** granifer ***
17	Striae and interstriae on disc never forming strial furrows (Fig. 29E)	**18**
–	Striae and interstriae on disc forming deep strial furrows and interstrial ridges (Fig. 28A)	**20**
18	Declivital interstrial granules large, widely spaced and uniseriate	*** hirsutulus ***
–	Declivital interstriae granules small, densely spaced and confused	**19**
19	Elytral interstriae bearing two rows of long thick semi-erect hair-like setae; shallow strial furrows on declivity	***sittichayai* sp. nov.**
–	Elytral interstriae bearing one row of short erect black bristles and longer semi-erect hair-like setae; strial furrows never present on declivity	*** granulifer ***
20	Discal interstriae with tubercles larger than those on the declivity	*** scabripennis ***
–	Discal and declivital interstriae with multiple rows of confused tubercles of equal size	**21**
21	Discal striae deeply impressed; elytral interstriae with at least two rows of tubercles and long erect, fine hair-like setae, setae 2× the width of an interstria	*** puberulus ***
–	Discal striae weakly impressed; elytral interstriae with two rows of granules and long semi-recumbent fine hair-like setae, setae 1–1.5× the width of an interstria	**22**
22	Elytral vestiture comprised of only hair-like setae on both disc and declivity, setae long, fine, and semi-recumbent	*** moestus ***
–	Elytral vestiture comprised of hair-like setae and golden scales, long semi-recumbent fine hair-like setae on disc; declivital interstriae densely covered by two or three rows of dense, confused golden scales	***setosus* sp. nov.**

#### Arixyleborus crassior sp. nov.

Taxon classificationAnimaliaColeopteraCurculionidae

http://zoobank.org/608E08A7-2078-4063-8254-9349A1CDF9C6

Fig. 25A, B, I

##### Type material.

***Holotype***, female, India: Arunachal Pradesh, Etalin vicinity, 28°36'56"N, 95°53'21"E, 700 m, 12–25.v.2012, L. Dembický (ZFMK).

##### Diagnosis.

2.0 mm long (n = 1); 2.5× as long as wide. This species is distinguished by the protibiae posterior faces inflated, granulate; antennal club wider than long; pronotum lateral margin oblique; pronotum anterior margin without serrations; posterolateral carina acute, granulate.

It can be further distinguished from the closely related *A. silvanus* by the more stout form (2.6–2.7× as long as wide in *A. silvanus*), more elongate pronotum (1.3 × longer than wide vs. 1.1–1.2× in *A. silvanus*), the more finely granulate interstriae, moderately impressed striae at the apex of the elytral disc, and the presence of short coarse setae on the declivity rather than fine hair-like setae. It can be further distinguished from the closely related *A. mediosectus* by the more stout form (2.86–3.33× as long as wide in *mediosectus*) and short coarse setae on the declivity.

##### Similar species.

*Arixyleborus mediosectus*, *A. phiaoacensis*, *A. silvanus*.

##### Description

**(female).** 2.0 mm (n = 1); 2.5× as long as wide. Body uniformly dark red-brown. Legs and antennae yellow-brown. ***Head***: epistoma entire, transverse, lined with a row of hair-like setae. Frons slightly convex from epistoma to upper level of eyes; surface alutaceous, shiny, sparsely punctate; punctures above epistoma large, coarse, shallow; punctures decreasing in size, coarseness, and depth from epistoma to upper level of eyes. Eyes deeply emarginated above level of antennal insertion, upper portion of eyes smaller than lower part. Scape regularly thick, shorter than club. Pedicle as long as funicle. Antennal funicle 4-segmented, segment 1 shorter than pedicel. Club wider than long and asymmetrical, club type 1; obliquely truncate, segment 2 not visible on posterior face; segment 1 covering posterior face, its margin completely costate; segment 2 narrow, pubescent with corneous part, visible on anterior face only. ***Pronotum***: 1.15× as long as wide. In dorsal view long and rounded frontally, type 7, sides parallel in basal 3/4, rounded anteriorly; anterior margin without serrations. In lateral view elongate with disc much longer than anterior slope, type 8, summit low. Surface shagreened, anterior 1/2 finely asperate; asperities close, arranged in concentric rings from midpoint of pronotum to anterior margin; anterolateral areas unarmed; disc minutely and sparsely punctate; glabrous. Lateral margins obliquely costate. Base weakly bisinuate; setal tuft absent. ***Elytra***: 1.5× as long as wide, 1.24× longer than pronotum. Scutellum moderately sized, linguiform, flush with elytra, flat. Elytral base weakly bisinuate, edge oblique, humeral angles rounded; sides straight from base to apical 1/2 of declivity then rounded to apex. Disc longer than declivity, distinctly separated; interstriae shiny, minutely, finely uniseriate punctate from base to midpoint, sparsely setose, nearly glabrous, basal 1/2 shagreened, dull, becoming sharply carinate and tuberculate; striae impressed on basal 1/2, strial punctures larger, shallower than on apical 1/2, interstriae laterally diverging from base to declivity and narrowed on declivity. Declivity obliquely truncate, densely shagreened, dull, sculpturing consisting of much weaker interstrial carinae and impressed striae; striae punctate, punctures large, shallow; interstriae tuberculate, tubercles small, each bearing a short, recumbent seta, less than the distance between tubercles in length, interstriae 1 strongly inflated on apical 1/2, interstriae 1–3 carinae extending to apex of declivity. Posterolateral margin carinate to interstriae 7. ***Legs***: protibiae slender, slightly broadened distally; posterior face inflated, tuberculate; outer margin of apical 1/2 with six small socketed denticles. Meso- and metatibiae flattened, outer margin evenly rounded, eight and ten socketed denticles on outer margin, respectively; posterior face unarmed.

##### Etymology.

L. *crassior* = stouter, comparative form of *crassus* (stout). An adjective.

##### Distribution.

India (Arunachal Pradesh).

##### Host plants.

The species has only been recorded from *Castanopsis* (Fagaceae).

##### Remarks.

The holotype is card mounted. Characters on the ventral surface including the submentum, prosternal posterocoxal piece, and denticles on the outer margins of the tibia were unable to be viewed. Socketed denticles are present on all tibiae.

**Figure 25.**
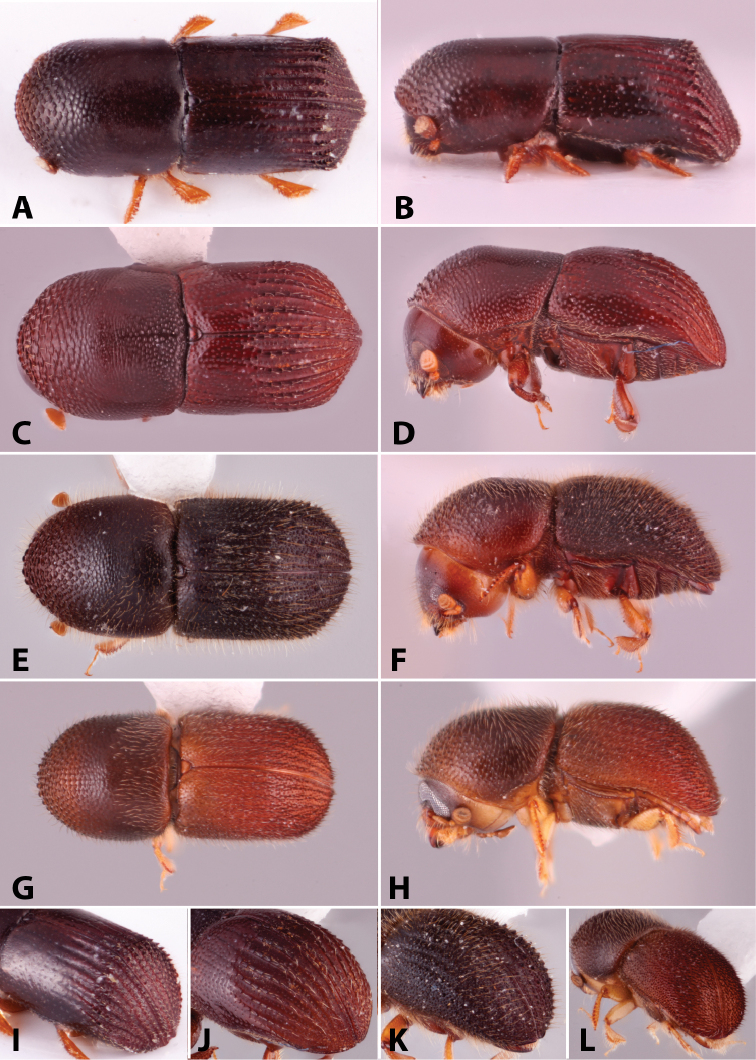
Dorsal, lateral and declivital view of *Arixyleborus crassior* holotype, 2.0 mm (**A, B, I**), *A. grandis*, 3.2–3.5 mm (**C, D, J**), *A. granifer*, 2.8–3.0 mm (**E, F, K**), and *A. granulifer*, 1.9–2.0 mm (**G, H, L**).

#### Arixyleborus grandis

Taxon classificationAnimaliaColeopteraCurculionidae

(Schedl, 1942)

Fig. 25C, D, J

Xyleboricus grandis Schedl, 1942c: 27.
Arixyleborus grandis (Schedl): Schedl 1952d: 161.

##### Type material.

***Lectotype*** (NHMW), ***paralectotype*** (NHMW, 1).

##### Diagnosis.

3.2–3.5 mm long (mean = 3.43 mm; n = 4); 2.13–2.33× as long as wide. This species is distinguished by the protibiae posterior faces inflated, granulate; antennal club wider than long; posterolateral costa carinate; pronotum lateral margin distinctly costate, nearly carinate; pronotum anterior margin elevated with row of serrations; large size; strial furrows 3× the width of interstrial ridges on disc; interstrial ridges setose, setae recumbent, hair-like, as long as striae 2 width at declivital base; interstrial ridges denticulate; striae moderately impressed; and declivity weakly shagreened, interstrial ridges almost appear shiny.

##### Similar species.

*Arixyleborus malayensis*, *A. tuberculatus*, *A. yakushimanus*.

##### Distribution.

Indonesia (Java), East Malaysia, New Guinea, Philippines, Thailand.

##### Host plants.

Recorded from *Canarium* (Burseraceae), *Dipterocarpus* (Dipterocarpaceae), *Mangifera* (Anacardiaceae), and *Palaquium* (Sapotaceae) (Beaver et al. 2014).

##### Remarks.

Kalshoven (1959b) gives some details of gallery systems and brood found in *Canarium* in Java.

#### Arixyleborus granifer

Taxon classificationAnimaliaColeopteraCurculionidae

(Eichhoff, 1878)

Fig. 25E, F, K

Xyleborus granifer Eichhoff, 1878a: 391.
Arixyleborus granifer (Eichhoff): Browne 1955: 350.
Xyleborus granifer borneensis Schedl, 1965: 27. Synonymy: Wood and Bright 1992: 666.

##### Type material.

Syntype(s) in UHZM destroyed in World War II (Wood and Bright 1992).

##### New records.

China: Yunnan, Banna, 24.i.2018, Shengchang Lai, ex *Hevea brasiliensis* (UFFE, 1). Laos: Kham Mouan, Ban Khun Ngeun, 18°07'N, 104°29'E, ~ 200 m, 24–29.iv.2001, Pacholátko (NHMB, 1). Louangphrabang, Ban Song Cha (5 km W), 20°33–4'N, 102°14'E, 1200 m, 1–16.iv.1999, V. Kubáň (RABC, 1); as previous except: Thong Khan, 19°55'N, 101°58'E, ~ 750 m, 11–21.v.2002 (NHMB, 3; RABC, 2).

##### Diagnosis.

2.8–3.0 mm long (mean = 2.94 mm; n = 5); 2.23–2.31× as long as wide. This species is distinguished by the protibiae posterior faces flat, unarmed; antennal club as broad as tall; posterolateral carina oblique, granulate; elytral disc with weak transverse saddle-like depression; and moderate size.

##### Similar species.

*Arixyleborus titanus*.

##### Distribution.

Borneo, China* (Yunnan), Laos*, East & West Malaysia, Myanmar, Philippines, Thailand.

##### Host plants.

Polyphagous. The frequent records from Dipterocarpaceae may simply reflect the abundance of this family in the forests of the region rather than indicating a preference for the family (Beaver et al. 2014).

##### Remarks.

The supposed syntype in MIZ (Wegrzynowicz and Mokrzycki 1996) is actually a specimen of *Xyleborus ferrugineus* (F.) (RAB pers. obs.).

#### Arixyleborus granulifer

Taxon classificationAnimaliaColeopteraCurculionidae

(Eggers, 1923)

Fig. 25G, H, L

Xyleborus granulifer Eggers, 1923: 206.
Arixyleborus granulifer (Eggers): Browne 1955: 350.

##### Type material.

***Lectotype*** (NMNH).

##### Diagnosis.

1.9–2.0 mm long (mean = 1.98 mm; n = 5); 2.11–2.44× as long as wide. This species is distinguished by the protibiae posterior faces flat, unarmed; antennal club as broad as tall; posterolateral carina oblique, granulate; elytral disc flat, without a transverse depression; striae not impressed; declivital interstriae bearing a row of short erect bristles and longer semi-erect hair-like setae, setae as long as an interstrial width.

##### Similar species.

*Arixyleborus hirsutulus*, *A. sittichayai*.

##### Distribution.

‘Borneo’, Indonesia (Mentawai Is, Sulawesi, Sumatra), East & West Malaysia, Philippines, Sri Lanka, Thailand.

##### Host plants.

Polyphagous. Ohno (1990), for example, records twenty different genera in fifteen different families.

##### Remarks.

Browne (1961b) describes the gallery system, and notes that the life cycle takes approximately 8 weeks.

#### Arixyleborus hirsutulus

Taxon classificationAnimaliaColeopteraCurculionidae

Schedl, 1969

Fig. 26A, B, I

Arixyleborus hirsutulus Schedl, 1969a: 212.

##### Type material.

***Holotype*** (PPST). Not examined.

##### Diagnosis.

2.0 mm long (n = 1); 2.27× as long as wide. This species is distinguished by the protibiae posterior faces flat, unarmed; antennal club as broad as tall; posterolateral carina oblique, granulate; elytral weakly convex, without a transverse depression; striae not impressed; declivital striae and interstriae covered with small equally sized granules; and elytra densely covered with setae, setae increasing in density towards apex.

##### Similar species.

*Arixyleborus granulifer*, *A. sittichayai*.

##### Distribution.

Philippines, Thailand. Imported to Japan from ‘Borneo’ and Indonesia (Maluku) (Sittichaya et al. 2019).

##### Host plants.

Recorded from *Anisoptera*, *Dipterocarpus*, *Dryobalanops*, *Shorea*. (Dipterocarpaceae), *Artocarpus* (Moraceae), and an unidentified species of Sapotaceae (Ohno 1990).

**Figure 26.**
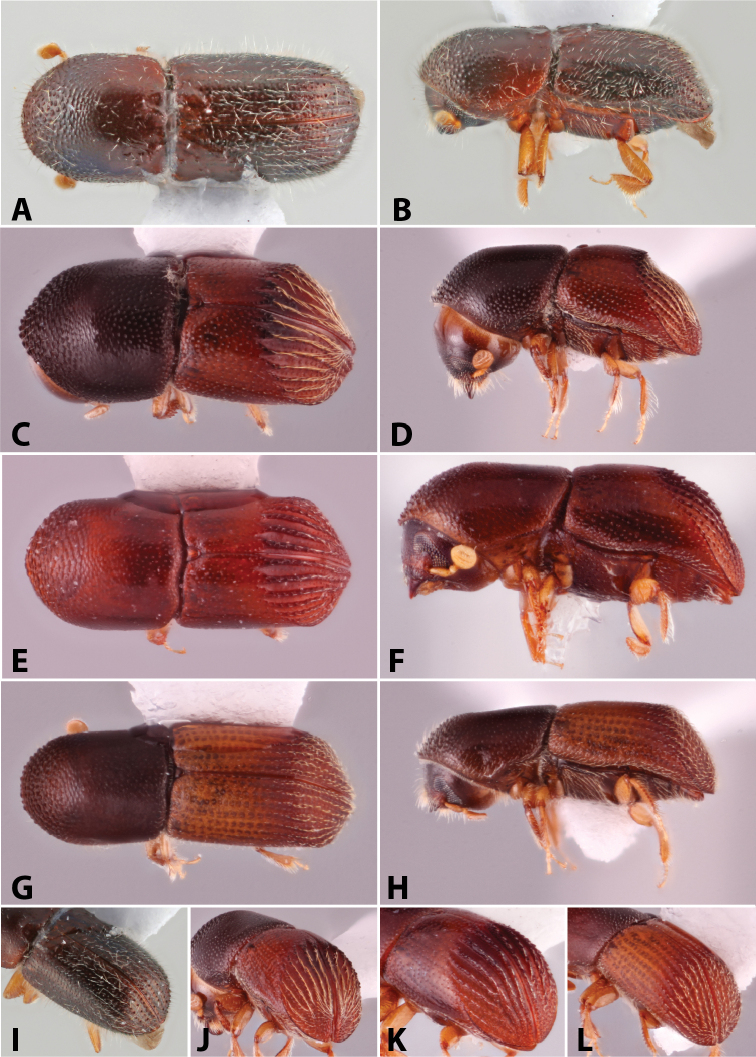
Dorsal, lateral and declivital view of *Arixyleborus hirsutulus*, 2.0 mm (**A, B, I**), *A. leprosulus*, 1.9–2.0 mm (**C, D, J**), *A. malayensis*, 2.1 mm (**E, F, K**), and *A. mediosectus*, 1.9–2.0 mm (**G, H, L**).

#### Arixyleborus leprosulus

Taxon classificationAnimaliaColeopteraCurculionidae

Schedl, 1953

Fig. 26C, D, J

Arixyleborus leprosulus Schedl, 1953b: 300.
Arixyleborus aralidii Nunberg, 1961: 618. Synonymy: Schedl 1962b: 699.

##### Type material.

***Lectotype****Arixyleborus leprosulus* (NHMW).

##### Diagnosis.

1.9–2.0 mm long (mean = 1.94 mm; n = 5); 2.38–2.5× as long as wide. This species is distinguished by the protibiae posterior faces inflated, granulate; antennal club wider than long; posterolateral carina costate to interstriae 7; elytra obliquely truncate, boundary between elytral disc and declivity distinct, declivital face without strial furrows and interstrial ridges; declivital interstriae setose, setae long, hair-like, recumbent, as long as 1.5 strial widths.

##### Similar species.

*Arixyleborus resecans*.

##### Distribution.

Brunei, West Malaysia, Thailand.

##### Host plants.

Recorded from *Dryobalanops* and *Shorea* (Dipterocarpaceae), *Castanopsis* (Fagaceae), *Palaquium* (Sapotaceae) (Browne 1961b), and *Aralidium* (Torricelliaceae) (Nunberg 1961).

#### Arixyleborus malayensis

Taxon classificationAnimaliaColeopteraCurculionidae

(Schedl, 1954)

Fig. 26E, F, K

Xyleboricus malayensis Schedl, 1954a: 150.
Arixyleborus malayensis (Schedl): Schedl 1958c: 145.

##### Type material.

***Lectotype*** (NHMW), ***paralectotypes*** (NHMW, 3).

##### New records.

China: S -Yunnan, Xishuangbanna, 37 km NW Jinghong, vic. Guo Men Shan, 22°14.48'N, 100°36.22'E, 780 m, 06.iv.2009, L. Meng (RABC, 1).

##### Diagnosis.

2.1 mm long (mean = 2.1 mm; n = 5); 2.63× as long as wide. This species is distinguished by the protibiae posterior faces inflated, granulate; antennal club wider than long; posterolateral carina costate; pronotum lateral margin distinctly costate, nearly carinate; pronotum anterior margin elevated with a row of serrations; strial furrows 3× width of interstrial ridges on disc; interstrial ridges setose, setae recumbent, hair-like, as long as striae 2 width at declivital base; interstrial ridges denticulate; striae strongly impressed; declivity weakly shagreened, interstrial ridges almost appear shiny; and moderate size.

##### Similar species.

*Arixyleborus grandis*, *A. tuberculatus*, *A. yakushimanus*.

##### Distribution.

China* (Yunnan), Indonesia (Java, Sumatra), West Malaysia, Sri Lanka, Thailand, Vietnam.

##### Host plants.

Polyphagous (Beaver et al. 2008).

##### Remarks.

The gallery system is typical of the genus. One gallery excavated by Kalshoven (1959b) contained 47 offspring.

#### Arixyleborus mediosectus

Taxon classificationAnimaliaColeopteraCurculionidae

(Eggers, 1923)

Fig. 26G, H, L

Xyleboricus mediosectus Eggers, 1923: 215.
Arixyleborus mediosectus (Eggers): Schedl 1958c: 145.
Arixyleborus angulatus Schedl, 1942a: 183. Synonymy: Wood 1989: 170.

##### Type material.

***Holotype*** (NMNH).

##### New records.

Laos: 10 km N Luang-Prabang, Mekhong river, 240 km N Vientiane, hills c. 250 m, poor settlem[ent], prim[ary] veget[ation], lux, iii.1993, Insomsay Somsy (MFNB, 1); Vientiane, Nan Van Eue, 15.xii.1966, native collector, ex light trap (BPBM, 1); as previous except: Gi Sion vill. De Tha Ngone, 28.ii.1965, J.L. Gressitt, ex light trap (BPBM, 1). Vietnam: Dong Nai, Cat Tien N.P., 11.42232, 107.42834, 128 m, 19.ii.2017, VN74, A.I. Cognato, T.A. Hoang, ex bottle trap (MSUC, 1).

##### Diagnosis.

1.9–2.1 mm long (mean = 1.98 mm; n = 5); 2.86–3.33× as long as wide. This species is distinguished by the protibiae posterior faces inflated, granulate; antennal club wider than long; pronotum lateral margin oblique; pronotum anterior margin without serrations; posterolateral carina acute, granulate.

It can be further distinguished from the closely related *A. silvanus* by the more elongate form (2.6–2.7× as long as wide in *A. silvanus*), more elongate pronotum (1.3 × longer than wide vs. 1.1–1.2× in *A. silvanus*), the more finely granulate interstriae, and shallowly impressed striae at the apex of the elytral disc, and the presence of short coarse setae on the declivity rather than fine hair-like setae. It can be further distinguished from the closely related *A. crassior* by the more elongate form (2.5× as long as wide in *A. crassior*) and short coarse setae on the declivity.

##### Similar species.

*Arixyleborus crassior*, *A. phiaoacensis*, *A. silvanus*.

##### Distribution.

‘Borneo’, Cambodia, India (Andaman Is, Assam), Indonesia (Sumatra), Laos*, East & West Malaysia, Philippines, Sri Lanka, Thailand, Vietnam.

##### Host plants.

Polyphagous (Maiti and Saha 2004). It has also been collected from the crop of an edible-nest swiftlet (*Collocalia fuciphaga*) (Beaver and Browne 1979).

#### Arixyleborus minor

Taxon classificationAnimaliaColeopteraCurculionidae

(Eggers, 1940)

Fig. 27A, B, I

Xyleboricus minor Eggers, 1940: 134.
Arixyleborus minor (Eggers): Schedl 1958c: 145.
Arixyleborus trux Schedl, 1975c: 359. Synonymy: Hulcr and Cognato 2013: 47.

##### Type material.

Not examined. Potentially housed in Museum Zoologicum Bogoriense, Cibinong, Java, Indonesia (Hulcr and Cognato 2013).

##### Diagnosis.

1.2–1.4 mm long (mean = 1.28 mm; n = 5); 3.0–3.5× as long as wide. This species is distinguished by its minute size; protibiae posterior faces inflated, granulate; antennal club as broad as tall; pronotum lateral margin oblique; pronotum anterior margin without serrations; posterolateral carina acute, carinate; and odd interstriae more strongly elevated than even interstriae.

##### Similar species.

*Arixyleborus suturalis*.

##### Distribution.

Indonesia (Java), East & West Malaysia, New Guinea, Thailand.

##### Host plants.

Recorded from *Dalbergia* (Fabaceae), *Castanea* (Fagaceae), *Dryobalanops* and *Shorea* (Dipterocarpaceae), *Palaquium* (Sapotaceae) (Browne 1961b; Kalshoven 1959b).

**Figure 27.**
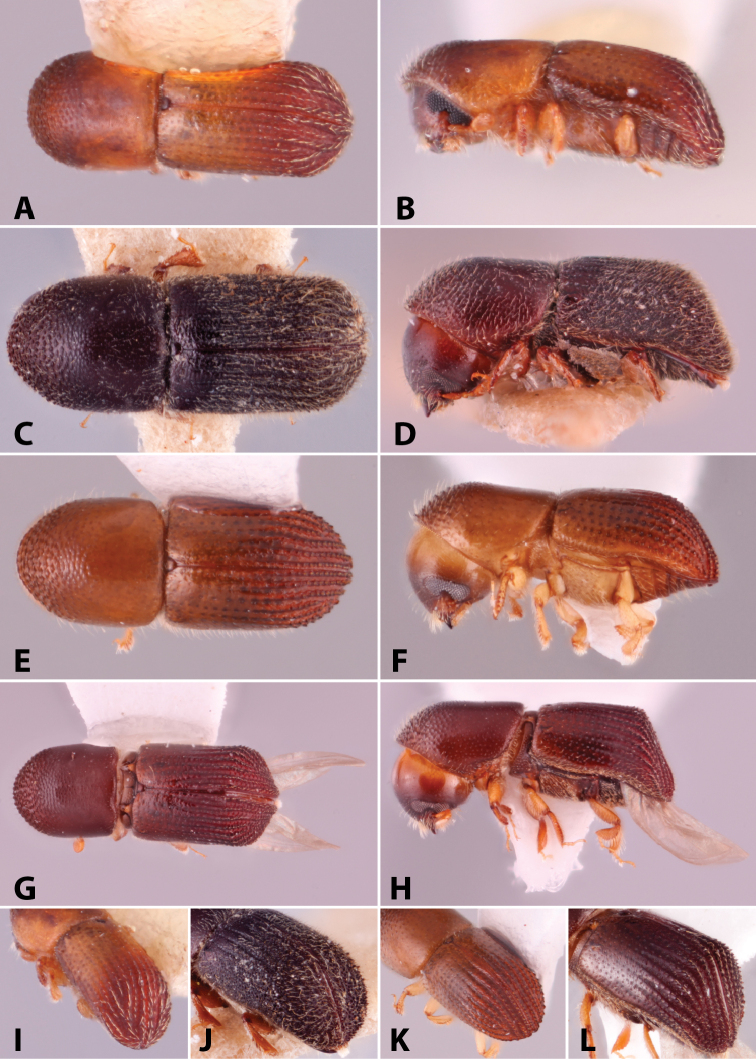
Dorsal, lateral and declivital view of *Arixyleborus minor*, 1.2–1.4 mm (**A, B, I**), *A. moestus* paratype, 2.5–2.7 mm (**C, D, J**), *A. nudulus* holotype, 1.5–1.8 mm (**E, F, K**), and *A. phiaoacensis* holotype, 2.2 mm (**G, H, L**).

#### Arixyleborus moestus

Taxon classificationAnimaliaColeopteraCurculionidae

(Eggers, 1930)

Fig. 27C, D, J

Xyleborus moestus Eggers, 1930: 189.
Arixyleborus moestus (Eggers): Browne 1955: 350.

##### Type material.

***Holotype*** (FRI).

##### New records.

Bhutan: W. distr. Thimpu, E Dochu La Menshunang, 2400 m, 7.vii.1988, C. Holzschuh (RABC, 1). Laos: Houa Phan, Ban Saluei – Phou Pan Mt., 20°12–13.5'N, 103°59.5–104°01'E, 1340–1780 m, 15.iv–15.v.2008, Lao collectors (MNHP, 1); as previous except: 20°12'N, 104°01'E, 1300–1900 m, 7.iv–25.v.2010, C. Holzschuh (NHMUK, 1). Louangnamtha, Namtha to Muang Sing, 21°09'N, 101°19'E, 900–1200 m, 5–31.v.1997, V. Kubáň (NHMB, 1). Oudomxai, Oudomxai, 17 km NE, 20°45'N, 102°09'E, ~ 1100 m, 1–9.v.2002, V. Kubáň (NHMB, 1).

##### Diagnosis.

2.5–2.7 mm long (mean = 2.62 mm; n = 5); 2.36–2.7× as long as wide. This species is distinguished by the protibiae posterior faces flat, unarmed; antennal club as broad as tall; posterolateral carina oblique, granulate; elytral disc flat, without a transverse depression; elytral striae weakly impressed; elytral interstriae with two rows of granules and long semi-recumbent fine hair-like setae, setae 1–1.5× width of an interstria.

##### Similar species.

*Arixyleborus puberulus*, *A. scabripennis*, *A. setosus*.

##### Distribution.

Bhutan*, India (Meghalaya, West Bengal), Laos*, Nepal.

##### Host plants.

Recorded only from *Quercus lamellosa* (Fagaceae) (Beeson 1930).

#### Arixyleborus nudulus

Taxon classificationAnimaliaColeopteraCurculionidae

Smith, Rabaglia & Cognato, 2018

Fig. 27E, F, K

Arixyleborus nudulus Smith, Rabaglia & Cognato, 2018 (in Smith et al. 2018c): 841.

##### Type material.

***Holotype*** (NMNH), ***paratypes*** (MSUC, 3; NMNH, 1).

##### Diagnosis.

1.5–1.8 mm long (mean = 1.56 mm; n = 5); 2.5–3.0× as long as wide. This species is distinguished by the protibiae posterior faces inflated, granulate; antennal club wider than long; pronotum lateral margin oblique; pronotum anterior margin without serrations; posterolateral carina acute, denticulate; strial furrows and interstrial ridges anteriorly extending no further than midpoint of disc; and interstriae sparsely setose with minute bristles, almost appearing glabrous.

##### Similar species.

*Arixyleborus mediosectus*.

##### Distribution.

Vietnam.

##### Host plants.

Unknown.

#### Arixyleborus phiaoacensis sp. nov.

Taxon classificationAnimaliaColeopteraCurculionidae

http://zoobank.org/C8B46393-B184-4DDD-823B-D4A5C6006E6D

Fig. 27G, H, L

##### Type material.

***Holotype***, female, Vietnam: Cao Bang, 22°33.118'N, 105°52.537'E, 1048 m, 12–17.vi.2014, VN9, Cognato, Smith, Pham, FIT (MSUC).

##### Diagnosis.

2.2 mm long (n = 1); 2.75× as long as wide. This species is distinguished by its moderate size; protibiae slender, slightly broadened distally, posterior faces inflated, granulate; antennal club as broad as tall; pronotum lateral margin oblique; pronotum anterior margin without serrations; posterolateral carina acute, granulate.

##### Similar species.

*Arixyleborus crassior*, *A. mediosectus*, *A. silvanus*.

##### Description

**(female).** Length 2.2 mm (n = 1); 2.75× as long as wide. Body uniformly red-brown. Legs and antennae yellow-brown. ***Head***: epistoma entire, transverse, lined with a row of hair-like setae. Frons slightly convex from epistoma to upper level of eyes; surface shagreened, dull, punctate; punctures above epistoma large, coarse, shallow; punctures decreasing in size, coarseness, and depth from epistoma to upper level of eyes; area between upper level of eyes reticulate. Eyes deeply emarginate above level of antennal insertion, upper portion of eyes smaller than lower part. Submentum deeply impressed, very narrow, triangular. Scape short and thick, shorter than club. Antennal funicle 4-segmented, segments equal in size. Pedicle as long as funicle. Club wider than long, asymmetrical, club type 1; obliquely truncate, segment 2 not visible on posterior face; segment 1 covering posterior face, its margin completely costate; segment 2 narrow, pubescent with corneous part, visible on anterior face only. ***Pronotum***: 1.25× as long as wide. In dorsal view long and rounded frontally, type 7, sides parallel in basal 2/3, rounded anteriorly; anterior margin with a row of serrations. In lateral view elongate with disc much longer than anterior slope, type 8, summit low. Surface shagreened, anterior 1/2 finely asperate, asperities close, arranged in concentric rings from midpoint of pronotum to anterior margin; anterolateral areas unarmed; disc minutely and sparsely punctate; punctures bearing minute setae slightly longer than puncture width. Lateral margins obliquely costate. Base weakly bisinuate, median region with a row of setae. ***Elytra***: 1.52× as long as wide, 1.34× longer than pronotum. Scutellum moderately sized, linguiform, flush with elytra, flat. Elytral base weakly bisinuate, humeral angles rounded, sides straight from base to apical 1/2 of declivity, then slightly acuminate to apex. Disc longer than declivity, distinctly and abruptly separated; interstriae shiny, minutely, finely uniseriate punctate from base to midpoint, sparsely setose, nearly glabrous, basal 1/2 shagreened, dull, becoming sharply carinate, tuberculate; striae impressed, strial punctures larger, shallower than on apical 1/2, interstriae laterally diverging from base to declivity and narrowed on declivity. Declivity obliquely truncate, densely shagreened, dull, sculpturing consisting of much weaker interstrial carinae and impressed striae; striae punctate, punctures large, shallow; interstriae tuberculate, tubercles small, bearing a short, recumbent seta, less than the distance between tubercles in length; interstriae 1 inflated on apical 1/2; interstriae 1–3 carinae extending to apex of declivity. Posterolateral margin carinate, granulate to interstriae 7. ***Legs***: procoxae contiguous; prosternal posterocoxal piece short, conical. Protibiae slender, slightly broadened distally; posterior faces inflated, granulate; outer margin of apical 1/2 with six small socketed denticles. Meso- and metatibiae flattened; outer margin evenly rounded, seven and eight socketed denticles on outer margin, respectively; posterior face unarmed.

##### Etymology.

In reference to the type locality, Phia Oac Nature Reserve. Latinized adjective.

##### Distribution.

Vietnam.

##### Host plants.

Unknown.

#### Arixyleborus puberulus

Taxon classificationAnimaliaColeopteraCurculionidae

(Blandford, 1896)

Fig. 28A, B, I

Xyleborus puberulus Blandford, 1896b: 215.
Arixyleborus puberulus (Blandford): Browne 1955: 351.
Xyleborus hirtipennis Eggers, 1940: 146. Synonymy: Hulcr 2010: 106.

##### Type material.

***Holotype****Xyleborus puberulus* (NHMUK).

##### Diagnosis.

2.6–2.9 mm long (mean = 2.66 mm; n = 5); 2.48–2.64× as long as wide. This species is distinguished by the protibiae posterior faces flat, unarmed; antennal club as broad as tall; posterolateral carina oblique, granulate; elytral disc flat, without a transverse depression; elytral striae deeply impressed on disc; elytral interstriae with at least two rows of tubercles and long erect fine hair-like setae, setae 2× width of an interstria.

##### Similar species.

*Arixyleborus moestus*, *A. scabripennis*, *A. setosus*.

##### Distribution.

Indonesia (Java), East Malaysia, New Guinea, Thailand.

##### Host plants.

Recorded from three genera of Dipterocarpaceae, *Canarium* (Burseraceae) (Ohno 1990) and *Hevea* (Euphorbiaceae).

##### Remarks.

Hulcr and Cognato (2013) synonymised *Xyleborus morio* Eggers, 1923 with this species. However, we consider it to be a distinct species. Hence it is not included in the list of synonyms.

**Figure 28.**
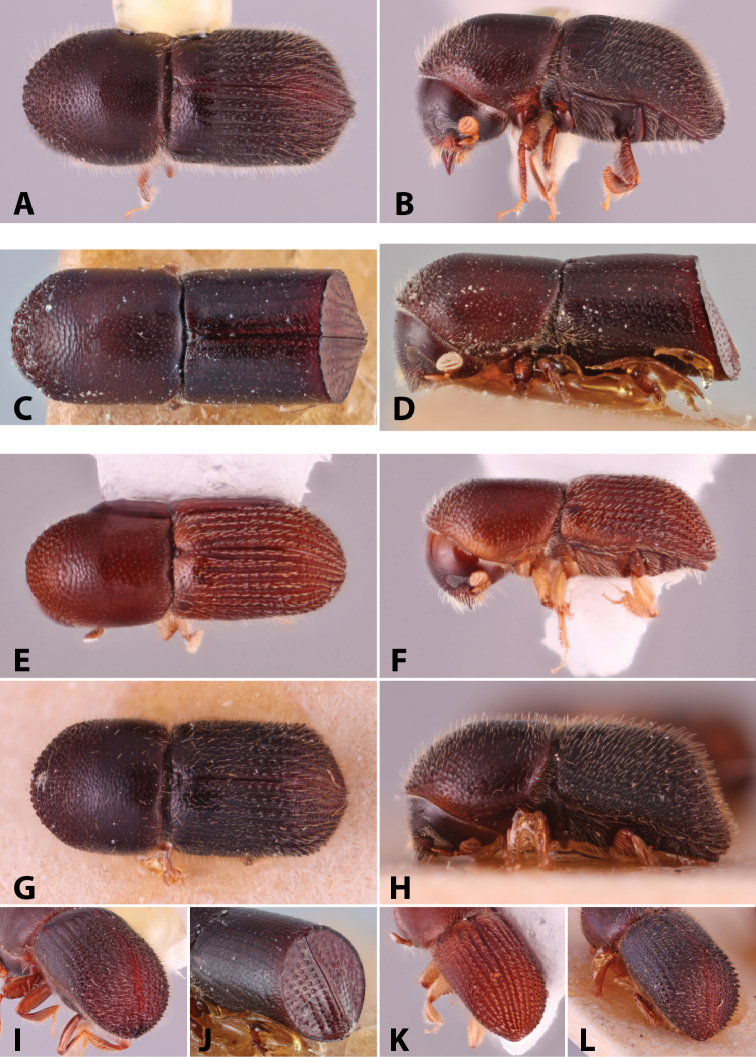
Dorsal, lateral and declivital view of *Arixyleborus puberulus*, 2.6–2.9 mm (**A, B, I**), *A. resecans* paratype, 3.0 mm (**C, D, J**), *A. rugosipes*, 1.7–2.0 mm (**E, F, K**), and *A. scabripennis*, 2.5–2.55 mm (**G, H, L**).

#### Arixyleborus resecans

Taxon classificationAnimaliaColeopteraCurculionidae

(Eggers, 1930) comb. nov.

Fig. 28C, D, J

Xyleborus resecans Eggers, 1930: 184.
Amasa resecans (Eggers): Wood and Bright 1992: 684.

##### Type material.

***Holotype*** (FRI), ***paratype*** (NMNH, 1).

##### Diagnosis.

3.0 mm long (n = 1); 2.72× as long as wide. This species is distinguished by the protibiae posterior faces inflated, granulate; antennal club wider than long; posterolateral carina costate; elytra truncate, surrounded by a circumdeclivital carina; boundary between elytral disc and declivity distinct, elytral disc without strial furrows and interstrial ridges; circumdeclivital carina emarginated at each striae (striae 5 and 6 may be weakly indicated); and declivital striae setose, setae minute, recumbent, as long as a strial puncture.

##### Similar species.

*Amasa* spp., *Arixyleborus leprosulus*.

##### Distribution.

India (Andaman Is, Assam).

##### Host plants.

Recorded from two species of *Dipterocarpus* (Dipterocarpaceae) (Maiti and Saha 2004).

##### Remarks.

This species is transferred to *Arixyleborus* from *Amasa*. This species displays a superficial morphological resemblance to *Amasa*, however it possesses the unique characteristics exhibited by *Arixyleborus* including protibiae slender, inflated and granulate on posterior faces, antennal club type 2, six striae present on the declivity, and pronotum from dorsal view elongated basic shape with rounded frontal margin (type 7).

#### Arixyleborus rugosipes

Taxon classificationAnimaliaColeopteraCurculionidae

Hopkins, 1915

Fig. 28E, F, K

Arixyleborus rugosipes Hopkins, 1915a: 59.
Webbia medius Eggers, 1927b: 104. Synonymy: Schedl 1952d: 162; Beaver and Liu 2010: 22.
Webbia camphorae Eggers, 1936a: 634. Synonymy: Browne 1955: 351; Beaver and Liu 2010: 22.

##### Type material.

***Holotype****Arixyleborus rugosipes* (NMNH). ***Holotype***, ***paratypes****Webbia camphorae* (NHMUK, 2). ***Lectotype****Webbia medius* (NMNH), ***paralectotype*** (NHMUK, 1).

##### New records.

Laos: Vientiane, Nan Van Eue, 15.xii.1966, native collector, ex light trap (BPBM, 2).

##### Diagnosis.

1.7–2.0 mm long (mean = 1.84 mm; n = 5); 2.83–3.33× as long as wide. This species is distinguished by the protibiae posterior faces inflated, granulate; antennal club wider than long; pronotum lateral margin oblique; pronotum anterior margin without serrations; posterolateral carina acute, denticulate; strial furrows and interstrial ridges anteriorly extending to basal 1/4 of elytral disc; and interstriae densely setose with long hair-like setae and bristles.

##### Similar species.

*Arixyleborus nudulus*.

##### Distribution.

India (Andaman Is), Indonesia (Java, Maluku, Sumatra), Laos*, East & West Malaysia, Philippines, Taiwan, Thailand, Vietnam.

##### Host plants.

Polyphagous. Browne (1961b) suggests a possible preference for Dipterocarpaceae, but this may simply reflect the abundance of this family in the forests of the region.

##### Remarks.

Browne (1961b) describes the condition of attacked host material, the gallery system and development of the species.

#### Arixyleborus scabripennis

Taxon classificationAnimaliaColeopteraCurculionidae

(Blandford, 1896)

Fig. 28G, H, L

Xyleborus scabripennis Blandford, 1896b: 216.
Arixyleborus scabripennis (Blandford): Browne 1955: 351.

##### Type material.

***Holotype*** (NHMUK).

##### New records.

Vietnam: Thua Thien-Hue, Bach Ma N.P., 16.20089, 107.84824, 919 m, 16.ii.2017, VN67, A.I. Cognato, T.A. Hoang, ex 2 cm dia; 8 cm diameter branch (MSUC, 1); as previous except: 16.22897, 107.85349, 415 m, 15.ii.2017, VN55, ex FIT (MSUC, 1).

##### Diagnosis.

2.5–2.55 mm long (mean = 2.51 mm; n = 4); 2.27–2.55× as long as wide. This species is distinguished by the protibiae posterior faces flat, unarmed; antennal club as broad as tall; posterolateral carina oblique, granulate; elytral disc flat, without a transverse depression; elytral striae weakly impressed; elytral interstriae with one row of dominant tubercles.

##### Similar species.

*Arixyleborus moestus*, *A. puberulus*, *A. setosus*.

##### Distribution.

Indonesia (Java, Maluku, Sumatra), East & West Malaysia, New Guinea, Sri Lanka, Thailand, Vietnam*.

##### Host plants.

Polyphagous (Browne 1961b).

##### Remarks.

Browne (1961b) gives some details of brood sizes and development.

#### Arixyleborus setosus sp. nov.

Taxon classificationAnimaliaColeopteraCurculionidae

http://zoobank.org/59AA24A6-6EBC-4548-8DD9-E36401E5ECA1

Fig. 29A, B, I

##### Type material.

***Holotype***, female, Vietnam: Cao Bang, 22°36.3'N, 105°52.6'E, 1435–1601 m, 13–17.iv.2014, VN16, Cognato, Smith, Pham, ex FIT (MSUC.). ***Paratypes***, female, Vietnam: Cao Bang, 22°36.454'N, 105°52.083'E, 1661 m, 15.iv.2014, VN39, Cognato, Smith, Pham, ex 3–6 cm branches (MSUC, 1); Lao Cai, Hoang Lien N.P., 22.35, 103.77, 1500–2000 m, 22.v.2019, VN186, S.M. Smith, A.I. Cognato, ex FIT (MSUC, 1).

##### Diagnosis.

2.5 mm long (n = 2); 2.5× as long as wide. This species is distinguished by the protibiae posterior faces flat, unarmed; antennal club as broad as tall; posterolateral carina oblique, granulate; elytral disc flat, without a transverse depression; elytral striae moderately impressed on disc; elytral interstriae with two rows of granules and long semi-recumbent fine hair-like setae on disc, setae 1–1.5× width of an interstria; and declivital interstriae densely covered by two or three rows of dense confused golden scales.

##### Similar species.

*Arixyleborus rugosipes*.

##### Description

**(female).** 2.5 mm long (n = 2); 2.5× as long as wide. Body color uniformly dark brown. Legs and antennae yellow-brown. Densely setose appearance on elytra, especially the declivity. ***Head***: epistoma entire, transverse, lined with a row of hair-like setae. Frons slightly convex from epistoma to upper level of eyes; surface shagreened, dull, punctate; punctures above epistoma large, coarse, shallow; punctures decreasing in size, coarseness, and depth from epistoma to upper level of eyes. Eyes deeply emarginate above level of antennal insertion, upper portion of eyes smaller than lower part. Submentum deeply impressed, triangular. Scape short and thick, approximately 3/4 length of club. Pedicle as long as funicle. Antennal funicle 4-segmented, segments equal in size. Club wider than long, asymmetrical, club type 1; obliquely truncate, segment 2 not visible on posterior face; segment 1 covering most of posterior face, its margin completely costate; segment 2 narrow, pubescent with corneous part, visible on anterior face only. ***Pronotum***: 1.0× as long as wide. In dorsal view long and rounded frontally, type 7, sides parallel in basal 3/4, rounded anteriorly; anterior margin without serrations. In lateral view elongate with disc slightly longer than anterior slope, type 7, summit low. Surface shagreened, anterior 1/2 finely asperate, asperities close, arranged in concentric rings from midpoint of pronotum to anterior margin; anterolateral areas unarmed; disc minutely and sparsely punctate; punctures bearing long, erect hair-like setae, as long as width of discal interstriae 2. Lateral margins obliquely costate. Base weakly bisinuate with a row of erect setae. ***Elytra***: 1.5× as long as wide, 1.53× as long as pronotum. Scutellum moderately sized, linguiform, flush with elytra, medially impressed. Elytral base weakly bisinuate, edge oblique, humeral angles rounded, sides straight from base to apical 1/2 of declivity then rounded to apex. Disc longer than declivity, distinctly separated and flat; striae impressed; interstriae shiny, densely, coarsely punctate in semicircular area from base to basal 1/4 and laterally to interstriae 6, punctures strongly confused, each bearing an erect golden hair-like seta equal in length to interstrial width, interstriae 2× width of striae, posterior 3/4 shagreened, dull, interstriae becoming densely, coarsely tuberculate apically; interstriae laterally diverging from base to declivity and narrowed on declivity. Declivity obliquely truncate, flattened, shagreened, dull; striae not impressed, impunctate; interstriae flattened, densely tuberculate and punctate, punctures dense, coarse, each bearing a short stout semi-erect scale. Posterolateral declivital margin costate, granulate. ***Legs***: procoxae contiguous; prosternal posterocoxal piece short, conical. Protibiae slender, broadest at apical 1/3; posterior faces flattened, unarmed; outer margin of apical 1/2 with six small socketed denticles. Meso- and metatibiae flattened, outer margin evenly rounded with nine socketed denticles; posterior face unarmed.

##### Etymology.

L. *setosus* = bristly. In reference to the declivity densely covered with setae. An adjective.

##### Distribution.

Vietnam.

##### Host plants.

Unknown.

**Figure 29.**
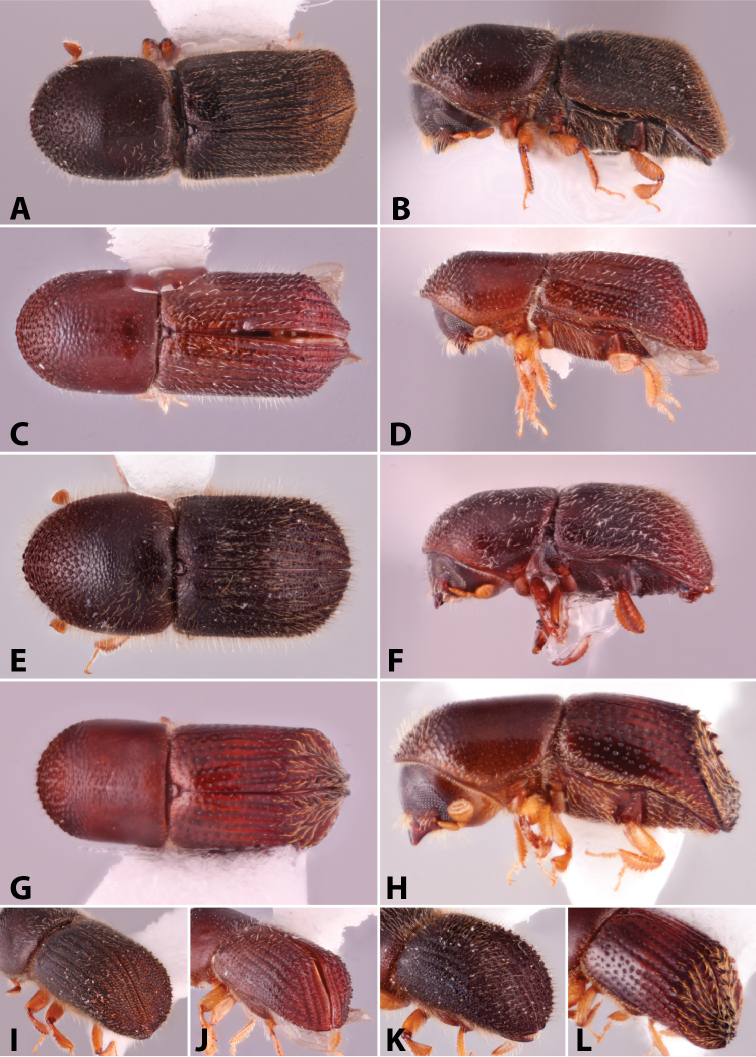
Dorsal, lateral and declivital view of *Arixyleborus setosus* holotype, 2.5 mm (**A, B, I**), *A. silvanus* holotype, 1.65–1.8 mm (**C, D, J**), *A. sittichayai* holotype, 2.3 mm (**E, F, K**), and *A. suturalis*, 1.5–1.7 mm (**G, H, L**).

#### Arixyleborus silvanus sp. nov.

Taxon classificationAnimaliaColeopteraCurculionidae

http://zoobank.org/54BBFA0F-B23E-436A-91B7-319A91607AB7

Fig. 29C, D, J

##### Type material.

***Holotype***, female, Thailand: Chiang Mai, Doi Pui, 1400 m, 20.xii.2004–10.i.2005, W. Puranasakul, ex EtOH trap (NHMUK). ***Paratypes***, female, as holotype (MSUC, 1); as previous except: 8–10.xi.2004 (QSBG, 1); as previous except: 10–31.i.2005 (RABC, 2); China: Guangxi, Shangsi, Shiwandashan, 27.iii.20017, Y. Li, ex *Liquidambar formosana* (IZAS, 1); Hainan, Wu-zhi-shan Town, 18.902N, 109.663E, 703 m, 2.xii.2016, Tian-Shang & Lv-Jia (RABC, 1).

##### Diagnosis.

1.65–1.8 mm long (mean = 1.72, n = 5); 2.6–2.7× as long as wide. This species is distinguished by its elongate form and steeply sloping declivity; posterolateral margin with a series of granules; pronotal summit distinctly anterior to middle; elytral disc with striae impressed on posterior part; and interstriae 1–3 extending to apex of declivity, armed with uniseriate granules.

*Arixyleborus silvanus* is distinguished from *A. mediosectus* by its less elongate form (2.9–3.3× as long as wide in *mediosectus*), and less elongate pronotum (1.1–1.2× longer than wide vs. 1.3× in *mediosectus*), the more coarsely granulate interstriae, and more deeply impressed striae at the apex of the elytral disc, and the presence of fine hair-like setae on the declivity rather than coarse setae. It can be further distinguished from the closely related *A. crassior* by the more elongate form (2.5× as long as wide in *A. crassior*), more stout pronotum (1.1–1.2× longer than wide vs. 1.3× in *A. crassior*), the more coarsely granulate interstriae, and weakly impressed striae at the apex of the elytral disc, and the presence of fine hair-like setae on the declivity rather than coarse setae.

##### Similar species.

*Arixyleborus crassior*, *A. mediosectus*, *A. phiaoacensis*.

##### Description

**(female).** 1.65–1.8 mm long (n = 5); 2.6–2.7× as long as wide. Body uniformly red-brown. Legs and antennae yellow-brown. ***Head***: epistoma entire, transverse, lined with a row of hair-like setae. Frons slightly convex from epistoma to upper level of eyes; surface alutaceous, shiny, sparsely punctate; punctures above epistoma large, coarse, shallow; punctures decreasing in size, coarseness, and depth from epistoma to upper level of eyes. Eyes deeply emarginate above level of antennal insertion, upper portion of eyes smaller than lower part. Submentum deeply impressed, very narrow, triangular. Scape short and thick, approximately 3/4 length of club. Pedicle as long as funicle. Antennal funicle 4-segmented, segment 1 shorter than pedicel. Club wider than long and asymmetrical, club type 1; obliquely truncate, segment 2 not visible on posterior face; segment 1 covering posterior face, its margin completely costate; segment 2 narrow, pubescent with corneous part, visible on anterior face only. ***Pronotum***: 1.33× as long as wide. In dorsal view long and rounded frontally, type 7, sides parallel in basal 2/3, rounded anteriorly; anterior margin without serrations. Pronotum with disc much longer than anterior slope, type 7, summit low. Surface shagreened, anterior 1/2 finely asperate, asperities close, arranged in concentric rings from midpoint of pronotum to anterior margin; anterolateral areas unarmed; disc minutely and sparsely punctate; glabrous. Lateral margins obliquely costate. Base weakly bisinuate; setal tuft absent. ***Elytra***: 1.53× as long as wide, 1.32× longer than pronotum. Scutellum moderately sized, linguiform, flush with elytra, flat. Elytral base weakly bisinuate, edge oblique, humeral angles rounded, sides straight from base to apical 1/2 of declivity, then rounded to apex. Disc longer than declivity, distinctly separated, shiny; striae impressed, punctures on basal 1/2 larger and deeper than those on apical 1/2; interstriae minutely, finely uniseriate punctate from base to midpoint, moderately setose, basal 1/2 of interstriae shagreened, dull, becoming sharply carinate, denticulate. Declivity obliquely truncate, strongly shagreened, dull; striae punctate, punctures large, shallow, glabrous; interstriae tuberculate, tubercles small, each bearing a fine hair-like seta, less than the distance between tubercles in length; interstriae 1–3 extending to apex of declivity, armed with uniseriate granules. Posterolateral declivital margin carinate, tuberculate to interstriae 7. ***Legs***: Protibiae slender, broadest at apical 1/3; posterior face inflated, tuberculate; outer margin of apical 1/2 with five small socketed denticles. Meso- and metatibiae flattened, outer margin evenly rounded with seven socketed denticles; posterior face unarmed.

##### Etymology.

L. *silvanus* = associated with forests. An adjective.

##### Distribution.

China (Guangxi, Hainan), Thailand.

##### Host plants.

This species is only recorded from *Liquidambar formosana* (Altingiaceae).

#### Arixyleborus sittichayai sp. nov.

Taxon classificationAnimaliaColeopteraCurculionidae

http://zoobank.org/28D53A21-7838-4E79-8D9C-C52A8EE944D9

Fig. 29E, F, K

##### Type material.

***Holotype***, female, Thailand: Nakhon Sri [Thammarat], Khao Luong [sic; = Khao Luang] N.P., 1.vi.2011, Wisut [Sittichaya], ex ET [ethanol trap] (MSUC).

##### Diagnosis.

2.3 mm long (n = 1); 2.3× as long as wide. This species is distinguished by the protibiae posterior faces flat, unarmed; antennal club as broad as tall; posterolateral carina oblique, granulate; elytral disc flat, without a transverse depression; striae not impressed on disc, feebly impressed on declivity; declivital interstriae bearing two rows of long thick semi-erect hair-like setae, setae as long as 1.5 interstrial widths.

##### Similar species.

*Arixyleborus granulifer*, *A. hirsutulus*.

##### Description

**(female).** 2.3 mm (n = 1); 2.3× as long as wide. Body uniformly dark red-brown. Legs and antennae yellow-brown. ***Head***: epistoma entire, transverse, lined with a row of hair-like setae. Frons slightly convex from epistoma to upper level of eyes; surface alutaceous, shiny, sparsely punctate; punctures above epistoma large, coarse, shallow, punctures decreasing in size, coarseness, and depth from epistoma to upper level of eyes. Eyes deeply emarginate above level of antennal insertion, upper portion of eyes smaller than lower part. Scape regularly thick, approximately as long as club. Pedicle as long as funicle. Antennal funicle 4-segmented, segment 1 shorter than pedicel. Club wider than long and asymmetrical, club type 1; obliquely truncate, segment 2 not visible on posterior face; segment 1 covering posterior face, its margin completely costate; segment 2 narrow, pubescent with corneous part, visible on anterior face only. ***Pronotum***: 1.06× as long as wide. In dorsal view long and rounded frontally, type 7, sides parallel in basal 2/3, rounded anteriorly; anterior margin without serrations. In lateral view with disc much longer than anterior slope, type 8, summit low. Surface shagreened, anterior 1/2 finely asperate, asperities close, arranged in concentric rings from midpoint of pronotum to anterior margin; anterolateral areas unarmed; disc minutely and sparsely punctate; glabrous. Lateral margins obliquely costate. Base weakly bisinuate; setal tuft absent. ***Elytra***: 1.39× as long as wide, 1.36× longer than pronotum. Elytral base weakly bisinuate, edge oblique, humeral angles rounded. Scutellum moderately sized, linguiform, flat, flush with elytra, medially impressed, sides straight from base to apical 1/2 of declivity then rounded to apex. Disc longer than declivity, distinctly separated, flat; striae impressed; interstriae shiny, densely, coarsely punctate in basal 1/4, punctures strongly confused, each bearing an erect golden-hair-like seta equal in length to interstrial width, interstriae 4× width of striae, posterior 3/4 shagreened, dull, becoming densely, coarsely tuberculate apically, interstriae straight from base to declivity and narrowed on declivity. Declivity rounded, convex, strongly shagreened, dull, sculpturing consisting of much weaker interstrial carinae and strial impression; striae feebly impressed, punctate, punctures large, shallow, glabrous; interstriae tuberculate, bearing two rows of long thick semi-erect hair-like setae, setae as long as 1.5 interstrial widths; interstriae 1–3 carinae not extending to apex of declivity. Posterolateral margin costate, granulate to interstriae 7. ***Legs***: protibiae slender, broadest at apical 1/3; posterior face flat, unarmed; outer margin of apical 1/2 with five small socketed denticles. Meso- and metatibiae flattened, outer margin evenly rounded with ten socketed denticles; posterior face unarmed.

##### Etymology.

Named for Dr. Wisut Sittichaya in recognition of his contributions to the study of bark and ambrosia beetles. Noun in genitive.

##### Distribution.

Thailand.

##### Host plants.

Unknown.

#### Arixyleborus suturalis

Taxon classificationAnimaliaColeopteraCurculionidae

(Eggers, 1936)

Fig. 29G, H, L

Xyleboricus suturalis Eggers, 1936b: 91.
Arixyleborus suturalis (Eggers): Schedl 1953b: 290.

##### Type material.

***Paratype*** (NHMW).

##### New records.

Vietnam: Dong Nai, Cat Tien N.P., 11.44221, 107.43114, 379 m, 20–22.ii.2017, VN78, A.I. Cognato, T.A. Hoang, ex FIT (MSUC, 1).

##### Diagnosis.

1.5–1.7 mm long (mean = 1.6 mm; n = 5); 2.5–3.0× as long as wide. This species is distinguished by the protibiae posterior faces inflated, granulate; antennal club wider than long; pronotum lateral margin oblique; pronotum anterior margin without serrations; posterolateral carina acute, carinate; and declivital interstriae 1 strongly elevated on apical 1/2, denticulate, denticles large.

##### Similar species.

*Arixyleborus minor*.

##### Distribution.

Indonesia (Java, Maluku), East & West Malaysia, Thailand, Vietnam*.

##### Host plants.

Polyphagous (Browne 1961b).

##### Remarks.

Browne (1961b) gives some details of brood sizes and development.

#### Arixyleborus titanus sp. nov.

Taxon classificationAnimaliaColeopteraCurculionidae

http://zoobank.org/D4670B6B-2C94-4FD1-A3DC-3D12D69B4F07

Fig. 30A, B, G

##### Type material.

***Holotype***, female, 云南西双版纳 1200–1600 公尺 1958.VII.26 采集者:王書永 [China: Yunnan, Xishuangbanna, Menghai, 1200–1600 m, 26.vii.1958, Shuyong Wang] (NMNH).

##### Diagnosis.

5.2 mm long (n = 1); 2.6× as long as wide. This species is distinguished by the protibiae posterior faces flat, unarmed; antennal club wider than long; posterolateral carina oblique, granulate; elytral disc with deep transverse saddle-like depression; and large size.

##### Similar species.

*Arixyleborus granifer*.

##### Description

**(female).** 5.2 mm (n = 1); 2.6× as long as wide. Body dark brown with red-brown declivity. Legs and antennae yellow-brown. ***Head***: epistoma entire, transverse, lined with a row of hair-like setae. Frons slightly convex from epistoma to upper level of eyes; weakly medially impressed between upper level of eyes; surface shagreened, dull, punctate; punctures above epistoma small, fine, shallow; punctures increasing in size, coarseness, and depth from epistoma to upper level of eyes; lower 1/2 of frons granulate. Eyes deeply emarginate above level of antennal insertion, upper portion of eyes smaller than lower part. Scape regularly thick, approximately 3/4 length of club. Pedicle shorter than funicle. Antennal funicle 4-segmented, segment 1 shorter than pedicel. Club wider than long and asymmetrical, club type 1; obliquely truncate, segment 2 not visible on posterior face; segment 1 covering posterior face, its margin completely costate; segment 2 narrow, corneous, visible on anterior face only. ***Pronotum***: 1.02× as long as wide. In dorsal view long and rounded frontally, type 7, sides parallel in basal 2/3, rounded anteriorly; anterior margin without serrations. In lateral view elongate with disc as long as declivity, type 7, summit moderate. Surface shiny, anterior 1/2 finely asperate, asperities close, arranged in concentric rings from midpoint of pronotum to anterior margin; anterolateral areas unarmed; disc finely, densely punctate; very long, erect hair-like setae, equal in length to 1.5× discal interstriae 1. Lateral margins obliquely costate. Base weakly bisinuate; setal tuft absent. ***Elytra***: 1.43× as long as wide, 1.27× longer than pronotum. Scutellum moderately sized, linguiform, clearly depressed below level of elytra. Elytral base weakly bisinuate, edge oblique, humeral angles rounded, sides straight from base to apical 1/2 of declivity then rounded to apex. Disc longer than declivity, indistinctly separated, shiny, median area concave, densely, finely punctate on basal 1/4; striae deeply impressed; interstrial punctures strongly confused, each bearing an erect golden hair-like seta equal in length to 3 interstrial widths, posterior 3/4 shagreened, dull, interstriae 3× width of striae, interstriae becoming sparsely tuberculate and granulate apically, interstriae laterally diverging from base to declivity and narrowed on declivity; declivity obliquely truncate, shagreened, dull; striae weakly impressed, distinctly punctate; interstriae impunctate, densely tuberculate, each tubercle bearing a long, erect golden hair-like seta equal in length to 3 interstrial widths; interstriae 1–3 inflated on apical 1/2. Posterolateral margin feebly costate, granulate to interstriae 7. ***Legs***: procoxae contiguous. Protibiae slender, broadest at apical 1/3; posterior face flat, unarmed. Meso- and metatibiae flattened, outer margin evenly rounded; posterior face unarmed.

##### Etymology.

L. *titanus* = of giants, large. In reference to its relatively large size. Noun in apposition.

##### Distribution.

China (Yunnan).

##### Host plants.

Unknown.

##### Remarks.

The holotype is card mounted with a large amount of glue. Characters on the ventral surface including the submentum, prosternal posterocoxal piece, and denticles on the outer margins of the tibia were unable to be viewed. Socketed denticles are present on all tibiae. Locality labels on the holotype are in Chinese and were translated by You Li. An English locality label has been placed on the specimen below the original locality labels.

**Figure 30.**
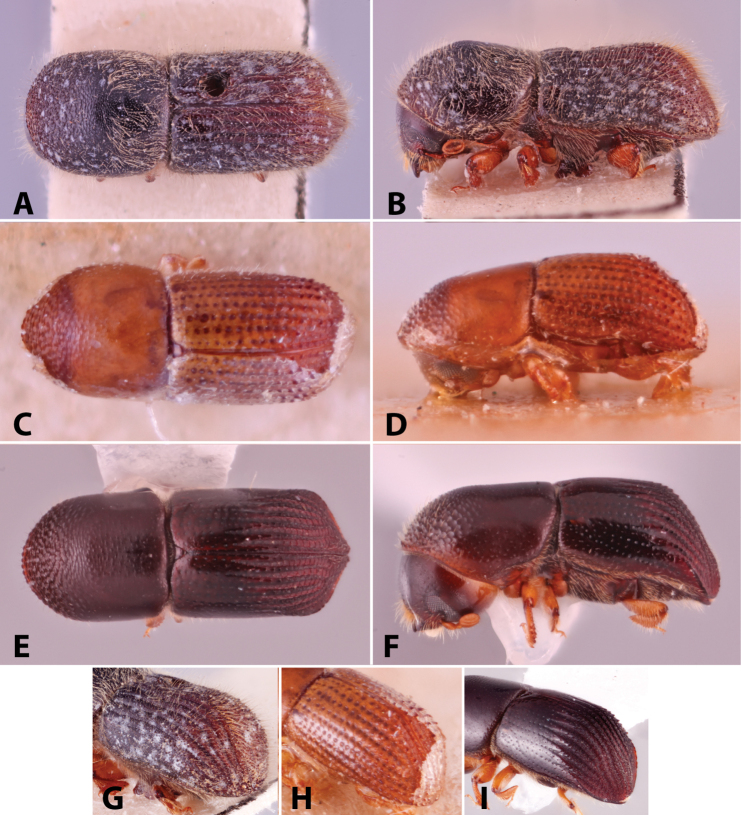
Dorsal, lateral and declivital view of *Arixyleborus titanus* holotype, 5.2 mm (**A, B, G**), *A. tuberculatus* paratype, 1.35–1.5 mm (**C, D, H**), and *A. yakushimanus*, 2.0–2.2 mm (**E, F, I**).

#### Arixyleborus tuberculatus

Taxon classificationAnimaliaColeopteraCurculionidae

(Eggers, 1940)

Fig. 30C, D, H

Xyleboricus tuberculatus Eggers, 1940: 133.
Arixyleborus tuberculatus (Eggers): Schedl 1958c: 145.

##### Type material.

***Paratype*** (NHMW).

##### Diagnosis.

1.35–1.5 mm long (mean = 1.42 mm; n = 2); 2.5–2.6× as long as wide. This species is distinguished by the protibiae posterior faces inflated, granulate; antennal club wider than long; posterolateral carina costate; pronotum lateral margin distinctly costate, nearly carinate; pronotum anterior margin elevated with a row of serrations; declivital interstriae except interstriae 1 denticulate; and minute size.

##### Similar species.

*Arixyleborus grandis*, *A. malayensis*, *A. yakushimanus*.

##### Distribution.

Indonesia (Java, Sumatra), Thailand.

##### Host plants.

Recorded from *Dalbergia* and *Parkia* (Fabaceae), and from *Cinchona* (Rubiaceae) (Kalshoven 1959b).

#### Arixyleborus yakushimanus

Taxon classificationAnimaliaColeopteraCurculionidae

(Murayama, 1958)

Fig. 30E, F, I

Xyleborus yakushimanus Murayama, 1955: 83.
Arixyleborus yakushimanus (Murayama): Nobuchi 1985: 28.

##### Type material.

***Holotype*** (NMNH).

##### New records.

China: Jiangxi, Long Nan, 12.vii.2016, Lv-Jia, Lai, S-C., ex *Cyclobalanopsis glauca* (RABC, 1). Yunnan, Xishuangbanna Sanchahe Nat. Res., 22°09.784'N, 100°52.256'E, 2186 m, 29–30.v.2008, A. Cognato (MSUC, 1). India: Bengal [Bihar], Dahura, Kurseong, 22.x.[19]33, N.C. Chatterjee (NMNH, 4). [West Bengal], Kalimpong, Samsingh, 18.iv.1934, N.C. Chatterjee, ex *Castanopsis* sp. (NMNH, 1). Laos: Vientiane, Ban Van Eue, 15.ii.1966, native collector, ex malaise trap (BPBM, 1). Taiwan: Nantou, Sun Moon Lake, 16.vi.2016, C.-S. Lin (MSUC, 2). Vietnam: Cao Bang, 22°34.118'N, 105°52.537'E, 1048 m, 12–17.iv.2014, Cognato, Smith, Pham, ex FIT (MSUC, 7). Thua Thien-Hue, Bach Ma N.P., 16.22897, 107.85349, 415 m, 15.ii.2017, VN55, A.I. Cognato, T.A. Hoang, ex 5 cm diameter (MSUC, 28; NHMUK, 2; NMNH, 2; VMNH, 4).

##### Diagnosis.

2.0–2.2 mm long (mean = 2.08 mm; n = 5); 2.5–2.75× as long as wide. This species is distinguished by the protibiae posterior faces inflated, granulate; antennal club wider than long; posterolateral carina costate; pronotum lateral margin distinctly costate, nearly carinate; pronotum anterior margin elevated with a row of serrations; strial furrows equal in width to interstrial ridges on disc; interstrial ridges glabrous or with minute setae no longer than 1/2 width of a strial furrow; interstrial ridges finely tuberculate; striae moderately impressed; declivity strongly shagreened; and moderate size.

##### Similar species.

*Arixyleborus grandis*, *A. malayensis*, *A. tuberculatus*.

##### Distribution.

China (Fujian, Jiangxi, Sichuan, Xizang, Yunnan), India* (Bihar, West Bengal), Japan, Laos*, Taiwan*, Thailand, Vietnam*.

##### Host plants.

Recorded from *Castanopsis* (Fagaceae) and *Machilus* (Lauraceae) (Yin et al. 1984).

##### Remarks.

The record of *Arixyleborus malayensis* from Doi Pui, Chiang Mai, Thailand in Beaver et al. (2014), and other unpublished records from this area, should be referred to this species. The species apparently occurs only in the north of the country and is replaced by *A. malayensis* in the central and southern regions.

### *Beaverium* Hulcr & Cognato, 2009

#### Beaverium

Taxon classificationAnimaliaColeopteraCurculionidae

Hulcr & Cognato, 2009

Beaverium Hulcr & Cognato, 2009: 25.

##### Type species.

*Xyleborus insulindicus* Eggers, 1923; original designation.

##### Diagnosis.

Large and robust species, 4.1–5.6 mm long, 2.2–2.55× as long as wide. *Beaverium* is distinguished by the declivity distinctly flattened and posterolaterally broadened, posterolateral declivital margin costate, terminating at interstriae 5; pronotal disc asperate; pronotum anterior margin with continuously elevated carina; scutellum flat, flush with elytra, mycangial tufts absent, and procoxae contiguous.

##### Similar genera.

*Ambrosiodmus*, *Fortiborus*, *Immanus*.

##### Distribution.

Distributed throughout mostly tropical regions of Asia, Australasia, and Oceania.

##### Gallery system.

This appears to have been described only in *B. insulindicus* (Eggers, 1923), a species not found in the study region. Based on observations in Fiji, Roberts (1977) notes a short radial gallery, penetrating 2–4 cm, with several longitudinal galleries parallel to the stem axis, all in the same plane, and without enlarged brood chambers.

#### Key to *Beaverium* species (females only)

**Table:** 

1	Elytral disc convex; posterolateral margin of declivity costate; smaller, 4.1–4.5 mm	*** lantanae ***
–	Elytral disc flat to concave with a transverse saddle-like depression; posterolateral margin of declivity carinate; larger, 5.0–6.0 mm	**2**
2	Declivity densely covered with long golden setae; elytral disc flat with a weak transverse impression; larger, 6.0 mm	*** latus ***
–	Declivity sparsely covered with long golden setae; elytral disc concave with a distinct transverse saddle-like depression; smaller, 5.0–5.6 mm	*** magnus ***

#### Beaverium lantanae

Taxon classificationAnimaliaColeopteraCurculionidae

(Eggers, 1930)

Fig. 31A, B, G

Xyleborus lantanae Eggers, 1930: 180.
Ambrosiodmus lantanae (Eggers): Wood and Bright 1992: 675.
Beaverium lantanae (Eggers): Beaver et al. 2014: 32.

##### Type material.

***Holotype*** (FRI).

##### New records.

Vietnam: Dong Nai, Cat Tien National Park, E of Crocodile Lake, 11°27'25"N, 107°21'7"E, 120 m, ex pan trap, 21–31.v.1999, D.C. Darling, B. Hubley (RABC, 1).

##### Diagnosis.

4.1–4.5 mm long (mean = 4.26 mm; n = 5); 2.2–2.28× as long as wide. This species is distinguished by the small size; elytral disc convex, without a transverse saddle-like depression; declivital posterolateral margins costate, never carinate; and boundary between elytral disc and declivity smoothly rounded.

##### Similar species.

*Beaverium latus*, *B. magnus*.

##### Distribution.

India (Karnataka, Nicobar Is, West Bengal), Myanmar, Thailand, Vietnam*.

##### Host plants.

Recorded from six genera in five families in India (Beeson 1961), and evidently polyphagous.

**Figure 31.**
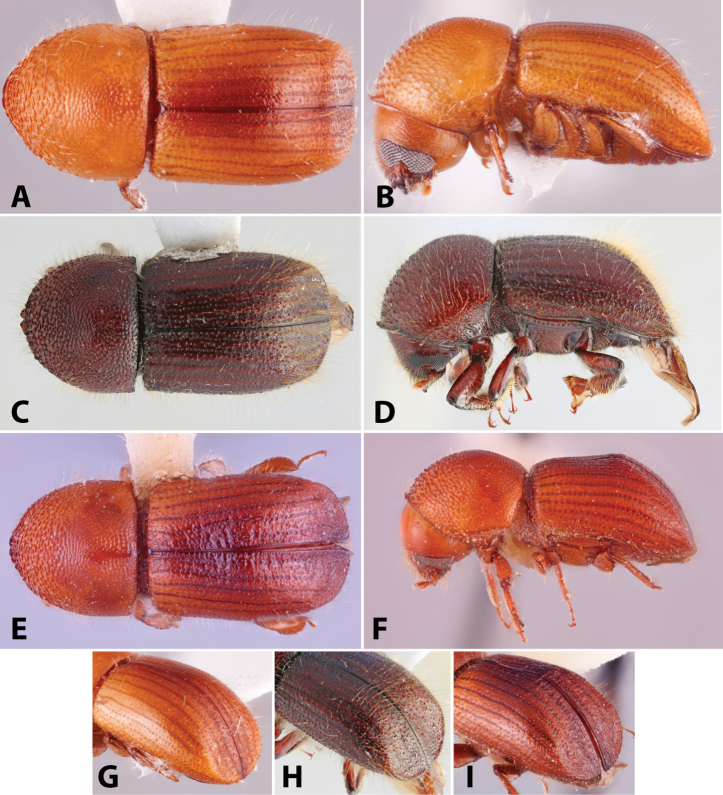
Dorsal, lateral and declivital view of *Beaverium lantanae*, 4.1–4.5 mm (**A, B, G**), *B. latus*, 6.0 mm (**C, D, H**), and *B. magnus*, 5.0–5.6 mm (**E, F, I**).

#### Beaverium latus

Taxon classificationAnimaliaColeopteraCurculionidae

(Eggers, 1923)

Fig. 31C, D, H

Xyleborus latus Eggers, 1923: 177.
Terminalinus latus (Eggers): Wood 1986: 267.
Beaverium latus (Eggers): Hulcr and Cognato 2009: 26.

##### Type material.

***Holotype*** (MCG). Not examined.

##### Diagnosis.

6.0 mm long (n = 1); 2.2× as long as wide (Sittichaya et al. 2019). This species is distinguished by the large size; declivity densely covered with long golden setae; elytral disc flat with a weak transverse impression; declivital posterolateral margins carinate; boundary between elytral disc and declivity distinct.

##### Similar species.

*Beaverium lantanae*, *B. magnus*.

##### Distribution.

‘Borneo’, Indonesia (Sumatra), East & West Malaysia, Thailand.

##### Host plants.

Recorded from *Parinari griffithiana* (Chrysobalanaceae), *Shorea balanocarpoides*, *S. leprosula*, *Shorea* sp. (Dipterocarpaceae), *Intsia palembanica* (Fabaceae), *Castanopsis sumatrana*, *Lithocarpus sundaicus* (Fagaceae) (Browne 1961b). Browne (1961b) suggests a possible preference for Dipterocarpaceae and Fagaceae hosts.

#### Beaverium magnus

Taxon classificationAnimaliaColeopteraCurculionidae

(Niisima, 1910)

Fig. 31E, F, I

Xyleborus magnus Niisima, 1910: 111.
Beaverium magnus (Niisima): Smith et al. 2018c: 841.
Xyleborus rufobrunneus var. dihingensis Eggers, 1930: 189. Smith et al. 2018c: 841.
Xyleborus chujoi Schedl, 1951a: 73. Smith et al. 2018c: 841.

##### Type material.

***Holotype***Xyleborus rufobrunneus var. dihingensis (FRI).

##### New records.

China: Chongqing, Jinfo Mtn, 9.v.2016, Tian-Shang, Lv-Jia, ex *Ficus* sp. (RABC, 1). Hong Kong, Tai Po Kau, vi.2017, J. Skelton (UFFE, 1). Jiangxi, Xin Feng, 29.vii.2016, Lai, S-C., ex *Castanopsis carlesii* (RABC, 1). Yunnan, Menglun, 750 m, 7.v.1962, Shimei Song, ex *Cassia siamea* [= *Senna siamea*] (NMNH, 1). Japan: Okinawa Pref., Iriomote-jima Island, 2.xi.2016, H. Kajimura, ex *Machilus thunbergii* (MSUC, 1). Taiwan: Nantau Co., WuCheng Village, Lien-Hun-Chih Station, 20.ix.2001, L. Stange, N. Wang, ex blacklight trap (FSCA, 1). Vietnam: Thua Thien-Hue, Bach Ma N.P., 16.22897, 107.85349, 415 m, 15.ii.2017, VN57, A.I. Cognato, T.A. Hoang, ex 5 cm diameter branch; twig (MSUC, 1).

##### Diagnosis.

5.0–5.6 mm long (mean = 5.2 mm; n = 10); 2.21–2.55× as long as wide. This species is distinguished by the moderate size; elytral disc concave with a transverse saddle-like depression; declivital posterolateral margins carinate; and boundary between elytral disc and declivity smoothly abrupt.

##### Similar species.

*Beaverium lantanae*, *B. latus*.

##### Distribution.

China (Chongqing*, Hong Kong*, Jiangxi*, Yunnan), India (Assam, West Bengal), Japan, Taiwan, Thailand, Vietnam.

##### Host plants.

Polyphagous. Recorded from *Artocarpus* (Moraceae), *Pterocarpus* (Fabaceae) (Beeson 1961), *Machilus* (Lauraceae), and *Senna* (Fabaceae).

### *Cnestus* Sampson, 1911

#### Cnestus

Taxon classificationAnimaliaColeopteraCurculionidae

Sampson, 1911

Cnestus Sampson, 1911: 383.
Tosaxyleborus Murayama, 1950: 49. Synonymy: Browne 1955: 368.

##### Type species.

*Cnestus magnus* Sampson, 1911; monotypy.

##### Diagnosis.

*Cnestus* species are typically moderate to large in size, 1.8–5.5 mm, and stout, 1.54–2.75× as long as wide. *Cnestus* is a morphologically variable genus but is distinguished by the eye feebly emarginate; lateral margin of the pronotum carinate from base to at least the midpoint; submentum depressed; procoxae narrowly separated; antennal club truncate, types 1 or 2 with segment 1 completely or almost covering the posterior face; antennal funicle 3- or 4-segmented; scutellum flat, flush with elytral surface. Most species have a mesonotal mycangium on the pronotal base.

##### Similar genera.

*Anisandrus*, *Hadrodemius*, *Xylosandrus*. *Cnestus* is closely related to *Anisandrus*, *Hadrodemius* and *Xylosandrus*, all of which possess a mesonotal mycangium and the associated dense tuft of hair-like setae at the scutellar area and pronotal base (Gohli et al. 2017; Johnson et al. 2018).

##### Distribution.

Distributed throughout Asia, Oceania and South America (Petrov and Mandelshtam 2018). One species is established in the United States (Schiefer and Bright 2004).

##### Gallery system.

The species, as far as is known, are twig and shoot-borers, and the gallery system is typical of such species with a short radial or circumferential gallery running to the middle of the stem, and longitudinal branches up and down the stem in which the brood develops.

#### Key to *Cnestus* species (females only)

**Table:** 

1	Mycangial tuft absent on pronotal base (Fig. 33H)	**2**
–	Mycangial tuft present on pronotal base (Fig. 33D)	**5**
2	Declivity convex and unarmed; anterior margin of pronotum strongly produced, extending into a process with numerous serrations; epistoma emarginate, mandibles enlarged (in lateral view protruding forward at 90° to the plane of the frons, dorsoventrally deeper than normal; in anterior view, with an upwardly directed, smooth, rounded process on the dorsal side)	**3**
–	Declivity sulcate, its margins armed with denticles or spines; anterior margin of pronotum with two large serrations; epistoma transverse; mandibles normal, not as described above	**4**
3	Smaller, 2.8–3.2 mm; pronotal base punctures fine, sparse; pronotal surface smooth, shiny; pronotum appearing narrow, sides parallel for approximately 2/3 of total length.	*** nitidipennis ***
–	Larger, 3.3–5.4 mm; pronotal base punctures coarse, dense; pronotal surface dull; pronotum appearing wide, sides parallel for approximately 1/2 of total length	*** protensus ***
4	Elytra bicolored, disc light brown, declivity and pronotum piceus; elytra with two large spines on each elytron, one at the declivital summit on interstriae 3 and a second on interstriae 5 at the lateral margin of the declivity; antennal club type 1, no sutures visible on posterior face (Fig. 2)	*** quadrispinosus ***
–	Elytra uniformly piceous; declivital interstriae 2–5 sparsely denticulate without large spines; antennal club type 2 with two sutures visible on posterior face (Fig. 2)	*** bicornioides ***
5	Elytral disc very short, less than 1/2 of elytral length, declivity obliquely truncate (Fig. 33D)	**6**
–	Elytral disc longer, more than 1/2 of elytral length, evenly curving into convex declivity (Fig. 32D)	**10**
6	Declivity bicolored with the basal 1/2 black and the apical 1/2 with a pale translucent area; declivital interstriae unarmed	*** improcerus ***
–	Declivity unicolored; declivital interstriae granulate	**7**
7	Pronotal disc sparsely and finely punctured (Fig. 32G); scutellum very small, elliptical; elytral disc approximately ten scutellum lengths; larger, 4.8–5.5 mm	*** gravidus ***
–	Pronotal disc densely and coarsely punctured (Fig. 33C); scutellum normal or very large, triangular; elytral disc 2–5 scutellum lengths; smaller, 3.6–4.4 mm	**8**
8	Scutellum very large (Fig. 33C); elytral disc 2–3 scutellum lengths	**9**
–	Scutellum of normal size (Fig. 32A); elytral disc 4–5 scutellum lengths	*** ater ***
9	Striae distinct on declivity, declivital striae 1 and 2 impressed; smaller, 3.6–3.8 mm	*** mutilatus ***
–	Striae indistinct on declivity, only declivital striae 1 impressed; larger, 4.2–4.4 mm	*** testudo ***
10	Declivital striae with much coarser, deeper punctures than on disc; declivital interstriae 2 and 3 strongly narrowed toward apex; declivital striae 1–3 impressed.	*** suturalis ***
–	Declivital striae with punctures similar to those on disc; declivital interstriae 2 and 3 not narrowed toward apex; at most declivital striae 1 and 2 impressed	*** aterrimus ***

#### Cnestus ater

Taxon classificationAnimaliaColeopteraCurculionidae

(Eggers, 1923)

Fig. 32A, B, I

Xyleborus ater Eggers, 1923: 210.
Xylosandrus ater (Eggers): Wood 1989: 177.
Cnestus ater (Eggers): Dole and Cognato 2010: 528.
Xyleborus retusiformis Schedl, 1936d: 31. Synonymy: Wood 1989: 177.

##### Type material.

***Holotype****Xyleborus retusiformis* (NHMW).

##### Diagnosis.

3.9–4.1 mm long (mean = 4.0 mm; n = 4); 1.63–1.70× as long as wide. This species is distinguished by the presence of a mesonotal mycangial tuft on the pronotal base; elytral disc 4–5× scutellum length; declivity obliquely truncate; pronotum type 1 when viewed dorsally; antennal club type 2, with two sutures visible on posterior face; antennal funicle 4-segmented; protibiae obliquely triangular; procoxae narrowly separated; declivital posterolateral margin weakly carinate from apex to declivital base along interstriae 7; declivital interstriae weakly granulate, setose with five or six rows of recumbent hair-like setae on interstriae 2 and 3; striae indistinct on declivity, striae 1 and 2 impressed; discal punctures dense, confused, surface distinctly reticulate between punctures; and body black with basal part of pronotal disc sometimes orange or brown.

##### Similar species.

*Cnestus gravidus*, *C. improcerus*, *C. mutilatus*, *C. testudo*.

##### Distribution.

‘Borneo’, China (Fujian), Indonesia (Sumatra), East & West Malaysia.

##### Host plants.

Polyphagous (Browne 1961b).

##### Remarks.

Browne (1961b) describes the gallery system and biology.

**Figure 32.**
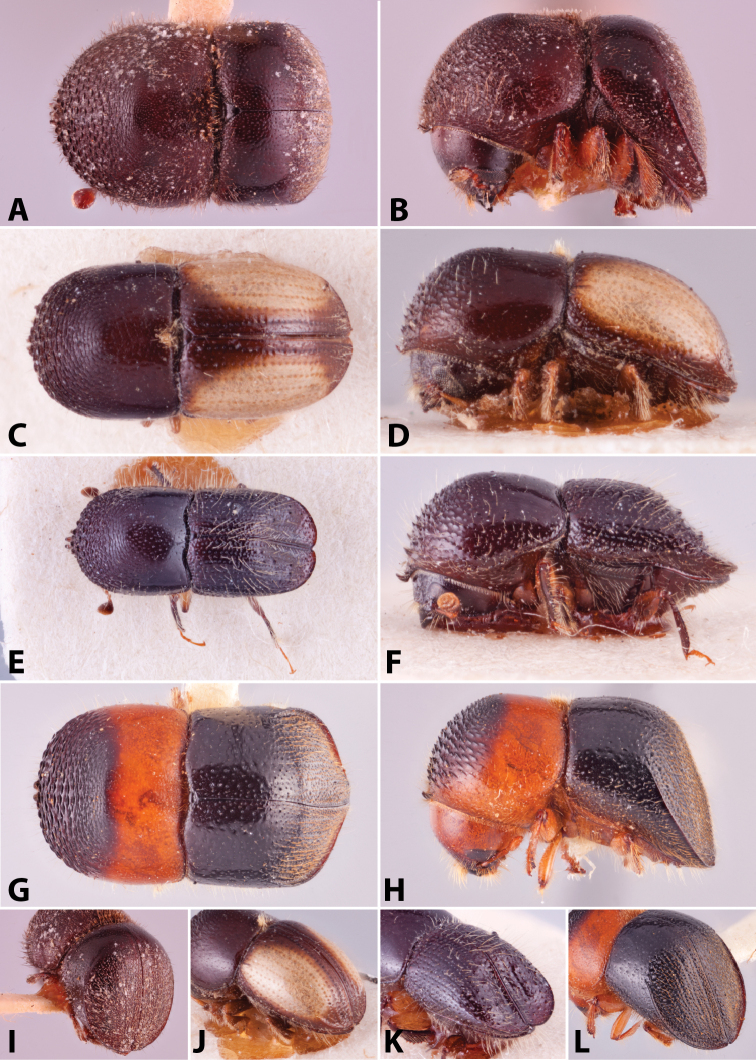
Dorsal, lateral and declivital view of *Cnestus ater* (holotype *Xyleborus retusiformis*), 3.9–4.1 mm (**A, B, I**), *C. aterrimus* (lectotype *C. pseudosuturalis*), 1.8–2.6 mm (**C, D, J**), *C. bicornioides* holotype, 3.3–3.7 mm (**E, F, K**), and *C. gravidus*, 5.0–5.5 mm (**G, H, L**).

#### Cnestus aterrimus

Taxon classificationAnimaliaColeopteraCurculionidae

(Eggers, 1927)

Fig. 32C, D, J

Xyleborus aterrimus Eggers, 1927a: 400.
Cnestus aterrimus (Eggers): Browne 1961b: 173.
Xyleborus glabripennis Schedl, 1942a: 189. Synonymy: Schedl 1958c: 145; Browne 1961b: 173.
Tosaxyleborus pallidipennis Murayama, 1950: 49. Synonymy: Smith et al. 2018b: 394.
Cnestus nitens Browne, 1955: 358. Synonymy: Schedl 1958c: 145.
Cnestus murayamai Schedl, 1962a: 207 (new name for C. pallidipennis Murayama nec Eggers 1940). Synonymy: Smith et al. 2018b: 394.
Cnestus murayamai Browne, 1963: 54 (new name for C. pallidipennis Murayama nec Eggers 1940). Synonymy: Smith et al. 2018b: 394.
Cnestus pseudosuturalis Schedl, 1964c: 315. Synonymy: Hulcr and Cognato 2013: 58.
Cnestus maculatus Browne, 1983b: 33. Synonymy: Smith et al. 2018b: 394.

##### Type material.

***Syntypes****Tosaxyleborus pallidipennis* (NMNH, 4). ***Paratype****Cnestus maculatus* (NHMUK). ***Lectotype****Cnestus pseudosuturalis* (NHMW).

##### New records.

China: Fujian, Quanzhou, 23.xi.2015, Y. Li, ex mango (UFFE, 1). Chongqing, Jinfo Mtn, 10.v.2016, Tian-Shang, Lv-Jia (RABC, 1); as previous except: Peng Shui, 10.v.2015, Tian-Shang, ex *Castanea mollissima* (RABC, 1). Hong Kong, Tai Po Kau, vi.2017, J. Skelton (MSUC, 1). Yunnan, S, Xishuangbanna, 23 km NW Jinghong, vic. Na Ban village (NNNR), 22°10'N, 100°39'E, 700–1000 m, v–vii.2009, L. Meng (NKME, 10; RABC, 3); as previous except: 20 km NW Jinghong, vic. Man Dian (NNNR), 22°07.80'N, 100°40.0'E, 740 m, rubber plantation, 23.v.2008, A. Weigel (RABC, 3); as previous except: forest, EKL, 10.x.2008; as previous except: S, Jinghong, Tian Zi garden, EKL, 15.xii.2007, A. Weigel (RABC, 2); as previous except: 37 km NW Jinghong, vic. Guo Men Shan, 22°14.48'N, 100°36.22'E, 1080 m, forest, 6.iv.2009, L. Meng (RABC, 2). Laos: Bolikhamxai, Ban nape (8 km NE), 18°21'N, 105°08'E, 600 m, 1–18.v.2001, V. Kubáň (RABC, 2). Louangnamtha, Namtha to Muang Sing, 21°09'N, 101°19'E, 900–1200 m, 5–31.v.1997, V. Kubáň (NHMB, 2). Vietnam: Hoa Binh, 1940, A. DeCooman (MNHN, 1). Lao Cai, Hoang Lien N.P., 22.35, 103.77, 1500–2000 m, 17.v.2019, VN202, S.M. Smith, A.I. Cognato, ex twigs (MSUC, 1). Tuyen Quang, Doi Can Tuyen Quang, 21.72740, 105.22742, 15.iv.2015, R.J. Rabaglia, ex funnel trap (RJRC, 1). Vinh Phuc, Dai Lai, *Acacia* hybrid plantation, alcohol lure, 11.i.2010, J. King (QDAFB, 1).

##### Diagnosis.

1.8–2.6 mm long (mean = 2.34 mm; n = 5); 2.09–2.36× as long as wide. This species is distinguished by the presence of a mesonotal mycangial tuft on the pronotal base; declivity rounded; elytra typically with a transparent area (may also be solid black); pronotum from dorsal view basic (type 1); antennal club type 1, with no sutures visible on the posterior face; antennal funicle 3-segmented; protibiae obliquely triangular; declivital striae with punctures similar to those of disc; and declivital interstriae 2 and 3 not narrowed toward apex, at most striae 1 and 2 impressed.

This species strongly resembles *C. suturalis* which has much coarser declivital strial punctures that are deeper than those of disc, interstriae 2 and 3 strongly narrowed toward apex, striae 1–3 impressed.

##### Similar species.

*Cnestus suturalis*.

##### Distribution.

China (Chongqing*, Fujian*, Hainan, Hong Kong*, Hubei, Hunan, Sichuan, Xizang, Yunnan*), Indonesia (Java, Sumatra), Japan, Laos*, West Malaysia, New Guinea, South Korea, Taiwan, Thailand, Vietnam.

##### Host plants.

Polyphagous (Browne 1961b).

##### Remarks.

Browne (1961b) describes the gallery system and biology.

#### Cnestus bicornioides

Taxon classificationAnimaliaColeopteraCurculionidae

(Schedl, 1952)

Fig. 32E, F, K

Xyleborus bicornioides Schedl, 1952a: 368.
Cnestus bicornoides (Schedl): Browne 1955: 360.

##### Type material.

***Holotype*** (NHMW).

##### New records.

China: Tibet [Xizang], Chayu, Shama, 2020 m, 21.vii.1973, ex Fagaceae sp. (NMNH, 2). S Yunnan, Xishuangbanna, 25 km NW Jinghong, vic. Zhang Zhi Chang (NNNR), 22°11.06'N, 100°39.05'E, 780 m, rubber plantation, EKL, 6.iv.2009, L. Meng (RABC, 1). Thailand: Chiangmai, Khun Chang Kian Highld Agr. Res. Stn, 18°50'23"N, 98°53'53"E, 1200–1300 m, 12.ii.2014, T. Saowaphak, ex EtOH trap (RABC, 2); as previous except: 26.ii.2014 (RABC, 2).

##### Diagnosis.

3.3–3.7 mm long (mean = 3.42 mm; n = 5); 2.36–2.75× as long as wide. This species is distinguished by the absence of a mesonotal mycangial tuft on the pronotal base; elongate body; declivity excavated; pronotum from dorsal view type 6; pronotum apex strongly produced, extending to a process with two serrations; mandibles normal; epistoma entire; elytral punctures distinct, clearly uniseriate; declivital interstriae bearing sparse erect hair-like setae; declivital interstriae 2–5 sparsely denticulate; protibiae very slender with three large narrow denticles; antennal club type 2, with two sutures visible on the posterior face; and 3-segmented antennal funicle.

##### Similar species.

*Cnestus bicornis* (from Indomalayan region), *C. quadrispinosus*.

##### Distribution.

China* (Xizang, Yunnan), India (Andaman Is, West Bengal), West Malaysia, Philippines, Thailand*.

##### Host plants.

Recorded from *Shorea* (Dipterocarpaceae), *Swietenia* (Meliaceae) (Browne 1961b) and Fagaceae.

##### Remarks.

The entries in Maiti and Saha (2004) under the name *Cnestus cruralis* (Schedl) refer to this species, which was earlier (Maiti and Saha 1986; Saha and Maiti 1996) referred to as *C. bicornioides*. The species described as *Xyleborus cruralis* Schedl belongs in the genus *Microperus* (Beaver 1998).

#### Cnestus gravidus

Taxon classificationAnimaliaColeopteraCurculionidae

(Blandford, 1898)

Fig. 32G, H, L

Xyleborus gravidus Blandford, 1898: 427.
Xylosandrus gravidus (Blandford): Wood and Bright 1992: 796.
Cnestus gravidus (Blandford): Dole and Cognato 2010: 529.

##### Type material.

***Holotype*** (NHMUK).

##### New records.

India: Arunachal Pradesh, Etalin vicinity, 28°36'56"N, 95°53'21"E, 700 m, 12–25.v.2012, L. Dembický (ZFMK, 9). Laos: Vientiane, Phou Kou Khouei, 800 m, 12–13.iv.1965, J.L. Gressitt (BPBM, 1). Vietnam: Cao Bang, Phia Oac hotel, 22°37.702'N, 105°54.5467'E, 847 m, 10–17.iv.2014, VN1, Cognato, Smith, Pham, ex in flight (MSUC, 2). NE region, Hanoi, Ba Vi Nat. Park, 16–18.vi.2016, 21°04.821'N, 105°22.034'E, G.S. Powell (MSUC, 1); as previous except: VQG Ba Vi, 400 m, 2.vi.2001 (MSUC, 1).

##### Diagnosis.

5.0–5.5 mm long (mean = 5.34 mm; n = 5); 1.83–2.0× as long as wide. This species is distinguished by the presence of a mesonotal mycangial tuft on the pronotal base; elytral disc short; declivity obliquely truncate; pronotum type 1 when viewed dorsally; antennal club type 2, with two sutures visible on posterior face; antennal funicle 4-segmented; protibiae distinctly triangular; procoxae narrowly separated; declivital posterolateral margin strongly carinate from apex to declivital base along interstriae 7; declivital interstriae granulate with a median row of long erect hair-like setae, clearly distinct from the ground vestiture; pronotal disc glabrous, shiny, sparsely punctate; and typically bicolored pronotum with apical 1/2 black and basal 1/2 orange.

##### Similar species.

*Cnestus ater*, *C. improcerus*, *C. mutilatus*, *C. testudo*.

##### Distribution.

Bangladesh, China (Hainan, Xizang, Yunnan), India (Arunachal Pradesh*, Assam, Sikkim, West Bengal), Laos, Myanmar, Nepal, Sri Lanka, Thailand, Vietnam.

##### Host plants.

Polyphagous (Beeson 1961).

#### Cnestus improcerus

Taxon classificationAnimaliaColeopteraCurculionidae

(Sampson, 1921)

Fig. 33A, B, I

Xyleborus improcerus Sampson, 1921: 33.
Xylosandrus improcerus (Sampson): Beaver 1998: 183.
Cnestus improcerus (Sampson): Dole and Cognato 2010: 529.

##### Type material.

***Holotype*** (NHMUK).

##### Diagnosis.

2.7–3.3 mm long (mean = 3.04 mm; n = 5); 1.67–1.74× as long as wide. This species is distinguished by the presence of a mesonotal mycangial tuft on the pronotal base; elytral disc very short; procoxae widely separated; declivity bicolored, with the basal 1/2 black and the apical 1/2 with a pale translucent area; declivity flat; pronotum type 1 when viewed dorsally; antennal club type 2, with two sutures visible on posterior face; antennal funicle 4-segmented; protibiae distinctly triangular; declivital posterolateral margin moderately carinate from apex to declivital base along interstriae 7; declivital interstriae punctate, setose, and ground vestiture absent.

##### Similar species.

*Cnestus ater*, *C. gravidus*, *C. mutilatus*, *C. testudo*, *Xylosandrus* spp.

##### Distribution.

‘Borneo’, Brunei, East & West Malaysia, Thailand.

##### Host plants.

Recorded from *Canarium* (Burseraceae), *Dipterocarpus* (Dipterocarpaceae), and an unidentified genus of Lauraceae (Browne 1961b).

##### Remarks.

Reported by Wood and Bright (1992) as occurring in India but no actual records have been found.

**Figure 33.**
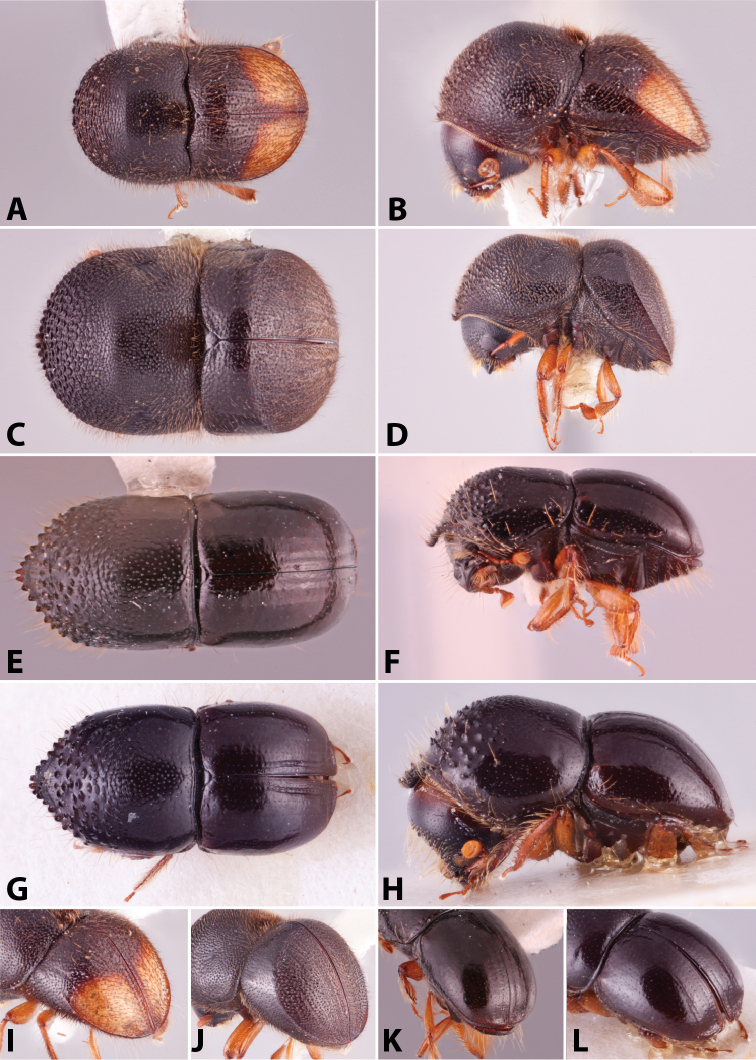
Dorsal, lateral and declivital view of *Cnestus improcerus*, 2.7–3.3 mm (**A, B, I**), *C. mutilatus*, 3.6–3.8 mm (**C, D, J**), *C. nitidipennis*, 2.8–3.2 mm (**E, F, K**), and *C. protensus*, 3.3–5.4 mm (**G, H, L**).

#### Cnestus mutilatus

Taxon classificationAnimaliaColeopteraCurculionidae

(Blandford, 1894)

Fig. 33C, D, J

Xyleborus mutilatus Blandford, 1894b: 103.
Xylosandrus mutilatus (Blandford): Wood 1989: 177.
Cnestus mutilatus (Blandford): Dole and Cognato 2010: 530.
Xyleborus sampsoni Eggers, 1930: 184. Synonymy: Wood 1989: 177.
Xyleborus banjoewangi Schedl, 1939b: 41. Synonymy: Kalshoven 1960: 63.
Xyleborus taitonus Eggers, 1939b: 118. Synonymy: Wood and Bright 1992: 799.

##### Type material.

***Holotype****Xyleborus mutilatus* (NHMUK).

##### New records.

China: Hong Kong, Tai Po Kau, vi.2017, J. Skelton, ex *Liquidambar* (MSUC, 1). Jiangsu, Nanjing, Laoshan National Park, Bacai Road, 32.09156N, 118.583701E, 15.viii.2017, Cognato, Li, Gao (MSUC, 2). Jiangxi, Nan Chang, 11.iv.2016, Lv-Jia, ex *Morus alba* (RABC, 1). Shanghai, Dongchuan, vii–viii.2017, Gao, ex trap w/ querciverol (MSUC, 4). Vietnam: Cao Bang, 22°33.118'N, 105°52.537'E, 1048 m, 12–17.vi.2014, VN9, Cognato, Smith, Pham, FIT (MSUC, 1).

##### Diagnosis.

3.6–3.8 mm long (mean = 3.76 mm; n = 5); 1.58–1.73× as long as wide. This species is distinguished by the presence of a mesonotal mycangial tuft on the pronotal base; elytral disc very short, 2× scutellum length; declivity obliquely truncate; pronotum type 1 when viewed dorsally; antennal club type 2, with two sutures visible on posterior face; antennal funicle 4-segmented; protibiae obliquely triangular; procoxae narrowly separated; declivital posterolateral margin weakly carinate from apex to declivital base along interstriae 7; declivital interstriae granulate, with recumbent hair-like setae, often a median row of long erect hair-like setae on upper part of declivity (varies geographically); interstriae 2 and 3 with three or four rows of setae; declivital striae 1 and 2 impressed; discal punctures dense, confused, surface between punctures with only traces of reticulation; and uniformly black body.

##### Similar species.

*Anisandrus ursulus*, *Cnestus ater*, *C. gravidus*, *C. improcerus*, *C. testudo*.

##### Distribution.

Throughout the Oriental region from India to Indonesia and New Guinea, and extending northwards to Japan, Korea, and Russia (Far East). Introduced and established in the United States (Schiefer and Bright 2004; Gomez et al. 2018a). Recorded in the study region from China (Anhui, Fujian, Guizhou, Hainan, Hong Kong*, Jiangsu*, Jiangxi*, Shaanxi, Shanghai*, Sichuan, Yunnan, Zhejiang), South Korea, Taiwan, Vietnam*.

##### Host plants.

Polyphagous (Wood and Bright 1992).

##### Remarks.

The biology of the species has been studied in Japan by Kajimura and Hijii (1992, 1994), in China by Tang (2000), and in USA by Stone and colleagues (Stone and Nebeker 2007; Stone et al. 2007). The associated ambrosia fungus has been described by Six et al. (2009). It is a pest of young *Castanea mollissima* (Fagaceae) trees in China (Zhejiang) (Tang 2000), but in USA appears to favor stressed host plants (Stone et al. 2007). *Cnestus mutilatus* is also strongly attracted to ethanol and has been reported to bore holes in and damage plastic gasoline containers (Carlton and Bayless 2011).

#### Cnestus nitidipennis

Taxon classificationAnimaliaColeopteraCurculionidae

(Schedl, 1951)

Fig. 33E, F, K

Xyleborus nitidipennis Schedl, 1951a: 88.
Cnestus nitidipennis (Schedl): Kalshoven 1959b: 165.

##### Type material.

***Holotype*** (NHMW).

##### New records.

China: Hainan, Jianfengling Mt., 600 m, 26.iii.1984, Shimei Song (NMNH, 1). Sichuan, Leibo, 19.iv.1964, ex *Carpinus* (NMNH, 1). S Yunnan, Xishuangbanna, 20 km NW Jinghong, vic. Man Dian (NNNR), 22°07.80'N, 100°40.05'E, 730 m, 8.vii.2008, A. Weigel (NKME, 1); as previous except: 23 km NW Jinghong, vic. Na Ban village (NNNR), 22°10'N, 100°39'E, 700–1000 m, v–vii.2009, L. Meng (RABC, 1); as previous except: 37 km NW Jinghong, vic. Guo Men Shan, 22°14.48'N, 100°36.22'E, 1080 m, forest, 28.vi.2008, L. Meng (NKME, 1). Vietnam: Lao Cai, Hoang Lien N.P., 22.35, 103.77, 1500–2000 m, 20.v.2019, VN185, S.M. Smith, A.I. Cognato, ex 1–2 cm branch (MSUC, 1).

##### Diagnosis.

2.8–3.2 mm long (mean = 3.0 mm; n = 4); 2.14–2.28× as long as wide. This species is distinguished by the uniquely emarginate epistomal margin; enlarged mandibles (in lateral view protruding forward at 90° to the plane of the frons, dorsoventrally deeper than normal; in anterior view, with an upwardly directed, smooth, rounded process on the dorsal side); absence of a mesonotal mycangial tuft on the pronotal base; pronotum from dorsal view type 6; pronotum apex strongly produced, extending to a process with numerous serrations; body glabrous, strongly shiny; declivity strongly rounded; protibiae very slender with three large, narrow denticles on outer margin; antennal club type 1, with no sutures visible on the posterior face; and 3-segmented antennal funicle.

This species is very similar to *C. protensus* and is distinguished by the smaller size, pronotal base with punctures clearly finer, sparser, surface smooth, shiny, pronotum appearing narrow, sides of pronotum parallel for approximately 2/3 total length.

##### Similar species.

*Cnestus protensus*.

##### Distribution.

China* (Fujian, Hainan, Sichuan, Yunnan), India (Arunachal Pradesh*, Sikkim), Indonesia (Java), Taiwan, Thailand, Vietnam.

##### Host plants.

Likely polyphagous. Recorded from *Eupatorium* (Asteraceae) (Kalshoven 1959b) and *Carpinus* (Betulaceae).

##### Remarks.

Both *C. nitidipennis* and *C. protensus* possess unique morphology among *Cnestus* species including the pronotal apex very strongly produced, very slender protibia, enlarged mandibles and absence of a mycangial tuft. These morphological characters are convergent with Neotropical genera such as *Sampsonius* Eggers, 1935 (Xyleborini) and *Amphicranus* Erichson, 1836 (Corthylini) (Wood 2007) which are inquilines. Further investigation of their behavior is necessary to determine if these species are also inquilines.

#### Cnestus protensus

Taxon classificationAnimaliaColeopteraCurculionidae

(Eggers, 1930)

Fig. 33G, H, L

Xyleborus protensus Eggers, 1930: 201.
Cnestus protensus (Eggers): Wood and Bright 1992: 803.
Cnestus rostratus Schedl, 1977: 502. syn. nov.

##### Type material.

***Holotype****Xyleborus protensus* (FRI). ***Holotype****Cnestus rostratus* (NHMW).

##### New records.

China: Fujian, Chong’an, Guidun, 950 m, 25.vi.1979, Fusheng Huang, ex *Machilus thunbergii* (NMNH, 1) as previous except: 1000 m, 8.v.1978, Fusheng Huang, ex evergreen broadleaf tree (NMNH, 1). Yunnan, Sutian, 2014, Tian-Shang (RABC, 3); S. Yunnan, 28 km NW Jinghong, vic. An Ma Xi Zhan (NNNR), 22°12'N, 100°38'E, 700 m, forest, EKL, 28.vi.2008, A. Weigel (RABC, 1). India: Arunachal Pradesh, Etalin vicinity, 28°36'56"N, 95°53'21"E, 700 m, 12–25.v.2012, L. Dembický (ZFMK, 1). Laos: Hua Phan, Ban Saluei, Phou Pan (Mt.), 20°12'N, 104°01'E, 1300–1900 m, 7.iv–25.v.2010, C. Holzschuh (RABC, 1).

##### Diagnosis.

3.3–5.4 mm long (mean = 4.35 mm; n = 4); 2.0–2.17× as long as wide. This species is distinguished by the uniquely emarginate epistomal margin; enlarged mandibles (in lateral view protruding forward at 90° to the plane of the frons, dorsoventrally deeper than normal; in anterior view, with an upwardly directed, smooth, rounded process on the dorsal side); absence of a mesonotal mycangial tuft on the pronotal base; pronotum from dorsal view type 6; pronotum apex strongly produced, extending to a process with numerous serrations; body glabrous, strongly shiny; declivity strongly rounded; protibiae very slender with three large, narrow denticles on outer margin; antennal club type 1, with no sutures visible on the posterior face; and 3-segmented antennal funicle.

This species is very similar to *C. nitidipennis* and is distinguished by the larger size, pronotal base with punctures clearly coarser, denser, surface mostly dull, pronotum appearing wider, sides of pronotum parallel for approximately 1/2 of the total length.

##### Similar species.

*Cnestus nitidipennis*.

##### Distribution.

China* (Yunnan*), India (Assam), Indonesia (Java), Laos*, Vietnam.

##### Host plants.

This species has only been recorded from *Machilus* (Lauraceae).

##### Remarks.

Images of the *Xyleborus protensus* holotype and the holotype of *C. rostratus* were compared. Though the two specimens differ in size (3.5 and 4.2 mm, respectively) they were clearly conspecific and *C. rostratus* is here placed in synonymy.

Both *C. nitidipennis* and *C. protensus* possess unique morphology among *Cnestus* species including the pronotal apex very strongly produced, very slender protibia, enlarged mandibles and absence of a mycangial tuft. These morphological characters are convergent with Neotropical genera that are inquilines (see remarks of *C. nitidpennis*). Further investigation of their behavior is necessary to determine if these species are also inquilines.

#### Cnestus quadrispinosus

Taxon classificationAnimaliaColeopteraCurculionidae

Sittichaya & Beaver, 2018

Fig. 34A, B, G

Cnestus quadrispinosus Sittichaya & Beaver, 2018: 32.

##### Type material.

***Holotype*** (NHMW), ***paratypes*** (MSUC, 1; NHMUK, 1; NHMW, 2; RABC, 2)

##### Diagnosis.

3.45–4.5 mm long (mean = 4.1 mm; n = 4); 2.2–2.56× as long as wide (Sittichaya and Beaver 2018). This species is distinguished from all other *Cnestus* by the presence of four large spines (two per elytron), one large spine at the declivital summit on interstriae 3 and a second large spine on interstriae 5 on the lateral margin of the declivity. It can be further distinguished by the absence of a mesonotal mycangial tuft on the pronotal base; declivity unarmed; elongate body; declivity excavated; pronotum from dorsal view type 6; pronotum apex strongly produced, extending to a process with two serrations; mandibles normal; epistoma entire; elytral punctures distinct, clearly uniseriate; interstriae bearing sparse erect hair-like setae; declivital interstriae 2–5 sparsely denticulate; protibiae obliquely triangular very slender with six or seven, narrow denticles on outer margin; antennal club type 1, with no sutures visible on the posterior face; and 3-segmented antennal funicle.

##### Similar species.

*Cnestus bicornioides*, *Cnestus bicornis* (from Indomalayan region).

##### Distribution.

Brunei, East Malaysia, Thailand.

##### Host plants.

Unknown but has been collected from dipterocarp forests (Sittichaya and Beaver 2018).

**Figure 34.**
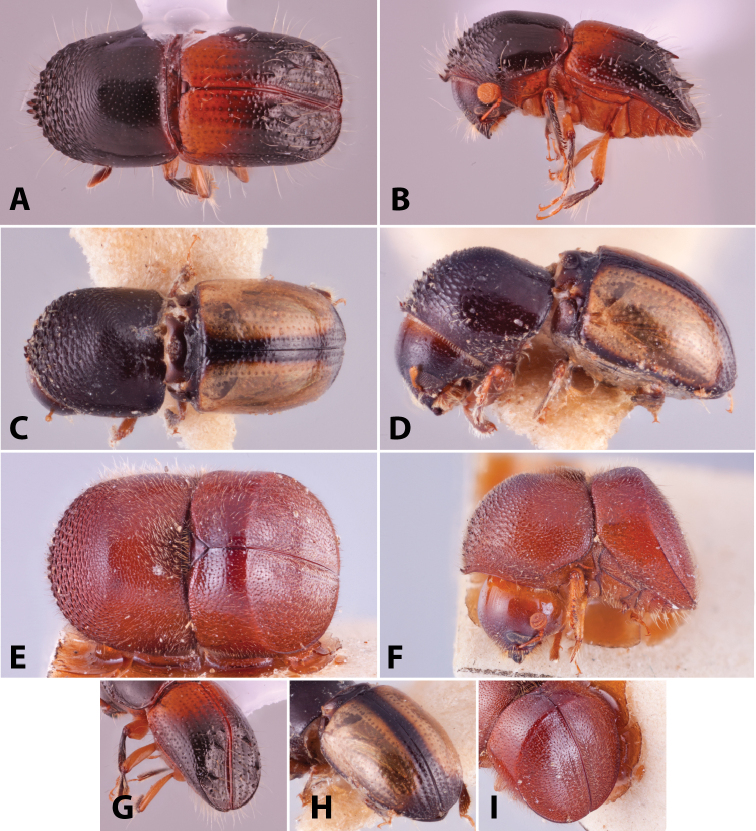
Dorsal, lateral and declivital view of *Cnestus quadrispinosus* paratype, 3.45–4.5 mm (**A, B, G**), *C. suturalis* paratype, 2.8 mm (**C, D, H**), and *C. testudo*, 4.2–4.4 mm (**E, F, I**).

#### Cnestus suturalis

Taxon classificationAnimaliaColeopteraCurculionidae

(Eggers, 1930)

Fig. 34C, D, H

Xyleborus suturalis Eggers, 1930: 200.
Cnestus suturalis (Eggers): Wood and Bright 1992: 803.

##### Type material.

***Holotype*** (FRI), ***paratype*** (NMNH, 1).

##### New records.

China: Guizhou, Guiyang, Huaxi, 8.iv.2015, Y. Li, ex in flight (UFFE, 1). Yunnan, Yulongshan mts., Ganhaizi pass, 27°06'N, 100°15'E, 3000–3500 m, 18–23.vii.1990, V. Kubáň (NHMB, 1; RABC, 1).

##### Diagnosis.

2.8 mm long (mean = 2.8 mm; n = 2); 2.55× as long as wide. This species is distinguished by the presence of a mesonotal mycangial tuft on the pronotal base; declivity rounded; elytra often with a transparent area; pronotum from dorsal view type 1; antennal club type 1, with no sutures visible on the posterior face; antennal funicle 3-segmented; protibiae obliquely triangular; declivital striae with punctures much coarser, deeper than those of disc; declivital interstriae 2 and 3 strongly narrowed toward apex; and striae 1–3 impressed.

This species strongly resembles *C. aterrimus* which has declivital strial punctures similarly sized to those of disc, interstriae 2 and 3 not narrowed toward apex, and at most striae 1 and 2 impressed.

##### Similar species.

*Cnestus aterrimus*.

##### Distribution.

China* (Guizhou, Yunnan), India (Andaman Is, Meghalaya), Indonesia (Java), Vietnam.

##### Host plants.

Recorded from *Eupatorium* (Asteraceae), *Terminalia* (Combretaceae), *Swietenia* (Meliaceae) and *Piper* (Piperaceae), and presumed polyphagous (Beeson 1961; Kalshoven 1959b).

#### Cnestus testudo

Taxon classificationAnimaliaColeopteraCurculionidae

(Eggers, 1939)

Fig. 34E, F, I

Xyleborus testudo Eggers, 1939b: 116.
Xylosandrus testudo (Eggers): Wood and Bright 1992: 801.
Cnestus testudo (Eggers): Dole and Cognato 2010: 532.

##### Type material.

***Lectotype*** (NMNH), ***paratypes*** (TARI, 3).

##### Diagnosis.

4.2–4.4 mm long (mean = 4.31 mm; n = 5); 1.54–1.62× as long as wide. This species is distinguished by the presence of a mesonotal mycangial tuft on the pronotal base; elytral disc short, 3× scutellum length; declivity obliquely truncate; pronotum type 1 when viewed dorsally; antennal club type 2, with two sutures visible on posterior face; antennal funicle 4-segmented; protibiae distinctly triangular; procoxae narrowly separated; declivital posterolateral margin weakly carinate from apex to declivital base along interstriae 7; declivital interstriae granulate, setose with recumbent ground vestiture and a median row of long erect hair-like setae; declivital striae 1 impressed; discal punctures dense, confused; and uniformly pitch black or piceous colored body with brown legs and antennae.

##### Similar species.

*Cnestus ater*, *C. gravidus*, *C. improcerus*, *C. mutilatus*.

##### Distribution.

China (Yunnan), Laos, Taiwan, Thailand, Vietnam.

##### Host plants.

Unknown.

### *Coptodryas* Hopkins, 1915

#### Coptodryas

Taxon classificationAnimaliaColeopteraCurculionidae

Hopkins, 1915

Coptodryas Hopkins, 1915a: 54.

##### Type species.

*Coptodryas confusa* Hopkins, 1915a; original designation.

##### Diagnosis.

1.8–4.0 mm, 1.88–2.71× as long as wide. *Coptodryas* is distinguished by the scutellum minute, convex, slightly raised above elytral surface or not apparent; dense tuft of setae present along elytral base associated with an elytral mycangium (*C. confusa* also has a pair of pit mycangia on the pronotal disc); elytral bases sinuate, costate; antennal club flattened, types 3 or 4, sutures gently sinuate and pubescent on anterior face, three sutures visible on posterior face; pronotal disc finely asperate (rarely punctate); pronotum from lateral view basic (type 0), or long and conical (type 5), rarely taller than basic (type 2; *C. confusa*); pronotum from dorsal view rounded (type 1) or basic and parallel sided (type 2), rarely conical (type 0; *C. confusa*); and anterior margin of pronotum with or without a row of 2–6 serrations. In addition, the procoxae are contiguous, outer margin of protibiae obliquely or distinctly triangular, armed by six or seven denticles, and posterior face flattened, unarmed.

##### Similar genera.

*Microperus*, *Schedlia*.

##### Distribution.

Species are distributed in tropical Asia and are rare in Melanesia.

##### Gallery system.

The gallery system in this genus appears to be rather variable (Browne 1961b). In *C. bella* and *C. punctipennis* (Schedl, 1953), an unbranched entrance tunnel leads to a single terminal brood chamber in the longitudinal plane. In *C. confusa*, the tunnels are simply branched and expanded in places to form small, irregular brood chambers in the longitudinal plane. In *C. quadricostata* and *C. curvidens* (Schedl, 1958), which usually breed in small diameter stems, there is a bifurcate or circumferential gallery in the transverse plane, and one or two longitudinal branches of very variable width in which the larvae develop (Browne 1961b).

##### Remarks.

*Coptodryas* is in need of further taxonomic/phylogenetic investigation given its potential polyphyly (Cognato et al. 2020b) and morphological overlap with *Microperus* (Hulcr et al. 2007).

#### Key to *Coptodryas* species (females only)

**Table:** 

1	Posterolateral margin of elytra rounded	**2**
–	Posterolateral margin of elytra carinate or costate	**3**
2	Pronotum with a pair of pit mycangia opening on the anterior slope of the elytra; elytra broadly rounded; protibiae with an evenly rounded outer margin; smaller, 1.8–2.2 mm, and stout, 2.0–2.25× as long as wide	*** confusa ***
–	Pronotum without a pair of pit mycangia; elytra acuminate, declivity gradual; protibiae distinctly triangular; larger, 2.3–2.4 mm, and elongate, 2.4–2.67× as long as wide	*** mus ***
3	Posterolateral margins of elytra costate; declivity obliquely truncate in lateral view; antennal club wider than long; larger, 3.75–4.0 mm	*** bella ***
–	Posterolateral margins of elytra carinate; declivity variously rounded in lateral view; antennal club circular or longer than wide; smaller, 1.9–3.2 mm	**4**
4	Declivital summit bearing four sharp spines that extend over the declivity	*** quadricostata ***
–	Declivital summit without spines	**5**
5	Elytral bases without a setal tuft; protibiae distinctly triangular; antennal club longer than wide	***inornata* sp. nov.**
–	Elytral bases with a dense tuft of setae extending at least to striae 3; protibiae obliquely triangular; antennal club circular	**6**
6	Elytral interstriae acutely carinate or costate; declivital face sulcate or bisulcate	**7**
–	Elytral interstriae never carinate, flat or feebly tumescent; declivital face subconvex	**9**
7	Basal 1/2 of declivity strongly sulcate, sulcate area v-shaped, margined by costate interstriae 3, 5, 6, interstriae 4 sharply carinate; larger, 2.7 mm	***carinata* sp. nov.**
–	Declivity weakly to moderately bisulcate, interstriae 2 to striae 3 weakly to moderately depressed, interstriae 4–7 carinate; smaller, 2.1–2.4 mm	**8**
8	Declivital interstriae 4 moderately tumescent and sharply carinate from base to apical 1/2 (Fig. 36C, D)	*** elegans ***
–	Declivital interstriae 4 strongly tumescent and sharply carinate from base to apical 1/4 (Fig. 35G, H)	*** concinna ***
9	Elytral discal striae punctate, interstriae impunctate; elytral disc shagreened; declivital interstriae 1–4 costate, 5 and 6 subcarinate; elongate, 2.71× as long as wide	***amydra* sp. nov.**
–	Elytral discal striae and interstriae punctate; elytral disc strongly shiny; declivital interstriae 2 and 3 depressed, remaining interstriae slightly tumescent; stout, 2.2–2.22× as long as wide	*** nudipennis ***

#### Coptodryas amydra sp. nov.

Taxon classificationAnimaliaColeopteraCurculionidae

http://zoobank.org/AFAB64B8-8AF9-4595-803E-B146C73EEC5A

Fig. 35A, B, I

##### Type material.

***Holotype***, female, Vietnam: Ninh Binh, Cuc Phuong N.P., Mac Lake, 20°15'29.0"N, 105°42'27.5"E, 155 m, 4–7.v.2009, J.B. Heppner, ex blacklight trap (FSCA).

##### Diagnosis.

1.9 mm long (n = 1); 2.71× as long as wide. This species is distinguished by the dense tuft of setae along the elytral base extending to interstriae 4; discal striae punctate, interstriae impunctate; elytral disc and declivity shagreened; declivital face subconvex; declivital interstriae 1–4 costate, 5 and 6 subcarinate; declivital posterolateral margin carinate to interstriae 7; pronotum basic (type 0) when viewed laterally, basic (type 2) when viewed dorsally; and anterior margin of the pronotum without a distinct row of serrations.

##### Similar species.

*Coptodryas carinata*, *C. concinna*, *C. elegans*, *C. nudipennis*.

##### Description

**(female).** 1.9 mm long (n = 1); 2.71× as long as wide. Pronotum, head, antennae, legs and elytral disc light brown, declivity dark brown. ***Head***: epistoma entire, transverse, with a row of hair-like setae. Frons weakly convex to upper level of eyes, shagreened, alutaceous, impunctate, glabrous. Eyes deeply emarginate just above antennal insertion, upper part smaller than lower part. Submentum narrowly triangular, slightly impressed. Antennal scape regularly thick, as long as club. Pedicel as wide as scape, shorter than funicle. Funicle 4-segmented, segment 1 shorter than pedicel. Club approximately circular, flattened, type 4; segment 1 corneous, small, convex; segment 2 larger than segment 1, narrow, transverse, corneous; segments 1–3 present on posterior face. ***Pronotum***: 1.06× as long as wide. In dorsal view basic and parallel-sided, type 2, sides parallel in basal 2/3, rounded anteriorly; anterior margin without serrations. In lateral view basic, type 0, disc flat, summit at midpoint. Anterior slope with densely spaced, broad asperities, becoming lower and more strongly transverse towards summit. Disc subshiny with dense minute punctures, glabrous, some longer hair-like setae at margins. Lateral margins obliquely costate. Base weakly bisinuate, posterior angles acutely rounded, almost subquadrate. ***Elytra***: 1.73× as long as wide, 1.63× as long as pronotum. Scutellum minute, convex, slightly raised above elytral surface. Elytral mycangium setal tuft along elytral base dense, extending to interstriae 4. Elytral base bisinuate, edge oblique, humeral angles rounded, parallel-sided in basal 4/5, narrowly rounded to apex. Disc flat, shagreened, striae not impressed, with minute shallow punctures separated by three diameters of a puncture, glabrous; interstriae flat, impunctate, glabrous. Declivity occupying approximately 2/5 of elytral length, rounded, face subconvex, strongly shagreened; striae flat, punctate, punctures much larger than those of disc and very shallow; interstriae 1–4 costate, five and six subcarinate, impunctate, feebly to moderately granulate. Posterolateral margin carinate to interstriae 7. ***Legs***: procoxae contiguous, prosternal coxal piece short, conical. Protibiae obliquely triangular, broadest at apical 1/3; posterior face smooth; apical 1/2 of outer margin with six moderate socketed denticles, their length as long as basal width. Meso- and metatibiae flattened, outer margins evenly rounded with eight and nine large socketed denticles, respectively.

##### Etymology.

G. *amydros* = indistinct. Named in reference to its uninteresting habitus. A Latinized adjective.

##### Distribution.

Vietnam.

##### Host plants.

Unknown.

**Figure 35.**
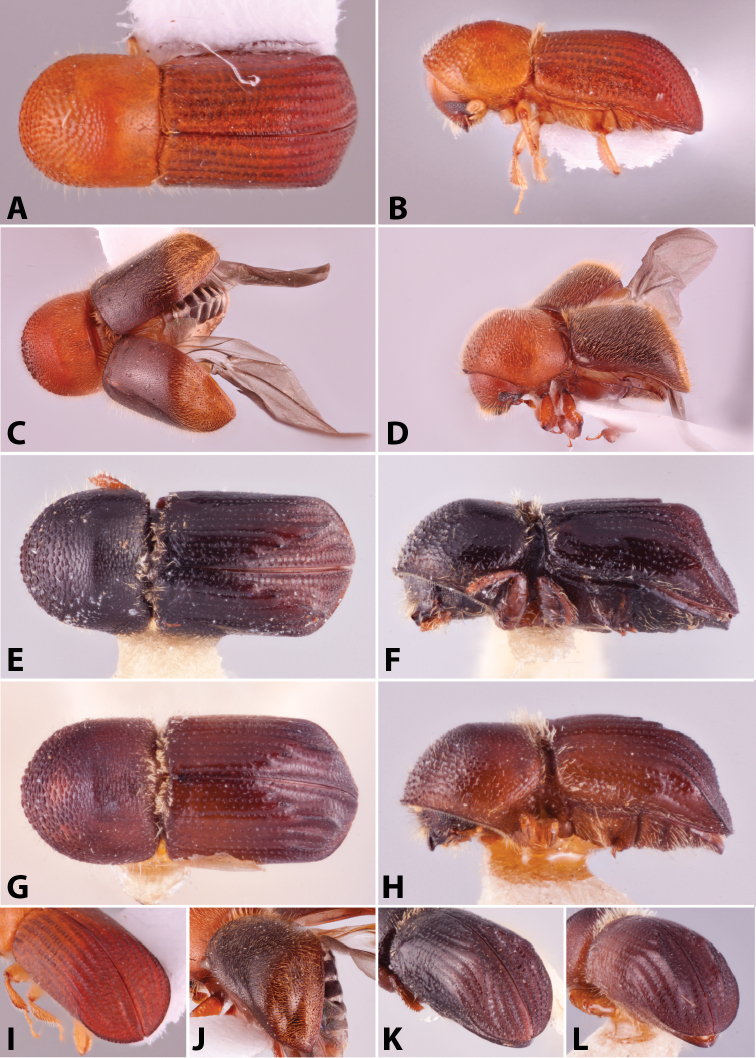
Dorsal, lateral and declivital view of *Coptodryas amydra* holotype, 1.9 mm (**A, B, I**), *C. bella*, 3.75–4.0 mm (**C, D, J**), *C. carinata* holotype, 2.7 mm (**E, F, K**), and *C. concinna*, 2.3 mm (**G, H, L**).

#### Coptodryas bella

Taxon classificationAnimaliaColeopteraCurculionidae

(Sampson, 1921)

Fig. 35C, D, J

Xyleborus bellus Sampson, 1921: 31.
Coptodryas bella (Sampson): Wood and Bright 1992: 823.

##### Type material.

***Holotype*** (NHMUK).

##### New records.

Philippines: Nueva Vizcaya, Quezon Munc., Mount Palali basecamp, 16.46228; 121.21975, 722 m, 6.vi.2017, Siler Brachymeles Expedition 4, ex light collecting (MSUC, 1).

##### Diagnosis.

3.75–4.0 mm long (mean = 3.95 mm; n = 5); 1.88–2.05× as long as wide. This species is distinguished by its large size; pronotum anterior margin with a pair of conspicuous serrations; densely setose body; declivity obliquely truncate; elytral strial and interstrial punctures confused; declivital interstriae granulate; and pronotum type 5 when laterally viewed.

##### Similar species.

*Coptodryas confusa*.

##### Distribution.

Indonesia (Maluku), East & West Malaysia, New Guinea, Philippines*, Thailand.

##### Host plants.

Recorded from *Vatica* (Dipterocarpaceae), and an unidentified genus of Euphorbiaceae (Browne 1961b).

#### Coptodryas carinata sp. nov.

Taxon classificationAnimaliaColeopteraCurculionidae

http://zoobank.org/FE30C58C-1AE0-4F84-B004-404B35321B67

Fig. 35E, F, K

##### Type material.

***Holotype***, female, 雲南:勐养 1000公尺 印度栲051 1962-V-10 采集者:宋士美 [China: Yunnan, Mengyang, 1000 m, 10.v.1962, Shimei Song, ex *Castanopsis indica*] (NMNH).

##### Diagnosis.

2.7 mm long (n = 1); 2.7× as long as wide. This species is distinguished by the dense tuft of setae along the elytral base extending to interstriae 8; body glabrous except for pronotal and elytral bases; striae and interstriae uniseriate punctate; elytral disc strongly shiny, declivity shagreened; basal 1/2 of declivity strongly sulcate, sulcate area v-shaped, margined by costate interstriae 3, 5, 6, and sharply carinate interstriae 4; apical 1/2 of declivity subconvex, interstriae costate, denticulate to apex; declivital posterolateral margin carinate to interstriae 7; protibiae obliquely triangular; pronotum rounded, robust (type 5) when viewed laterally, and basic (type 2) when viewed dorsally.

##### Similar species.

*Coptodryas amydra*, *C. concinna*, *C. elegans*, *C. nudipennis*.

##### Description

**(female).** 2.7 mm long (n = 1); 2.7× as long as wide. Body, antenna, and legs dark brown. Body glabrous except for pronotal and elytral bases. ***Head***: epistoma entire, transverse, with a row of hair-like setae. Frons weakly convex to upper level of eyes, strongly shagreened, alutaceous, punctate, punctures fine, dense, setose, setae long, erect, hair-like. Eyes deeply emarginate just above antennal insertion, upper part smaller than lower part. Submentum narrowly triangular, slightly impressed. Antennal scape regularly thick. Pedicel as wide as scape. ***Pronotum***: 0.69× as long as wide. In dorsal view basic and parallel-sided, type 2, sides parallel in basal 2/3, rounded anteriorly; anterior margin with a row of five serrations. In lateral view rounded and robust, type 5, disc flat, summit at apical 2/5. Anterior slope with densely spaced, broad asperities, becoming lower and more strongly transverse towards summit. Disc shagreened, weakly rugose, impunctate glabrous. Some longer hair-like setae at anterior and lateral margins and a dense narrow median tuft along base laterally extending to striae 3. Lateral margins obliquely costate. Base weakly bisinuate, posterior angles acutely rounded, almost subquadrate. ***Elytra***: 1.53× as long as wide, 2.22× as long as pronotum. Scutellum minute, convex, slightly raised above elytral surface. Elytral mycangium setal tuft along elytral base dense, extending to interstriae 8. Elytral base bisinuate, edge oblique, humeral angles rounded, parallel-sided in basal 2/3, then rounded to apex. Disc flat, strongly shiny, striae weakly impressed, with large deep punctures separated by less than 1–2 diameters of a puncture, glabrous; interstriae flat, minutely uniseriate punctate, punctures sparse, spaced 2–4 diameters of a puncture, glabrous. Declivity occupying approximately 2/3 of elytral length, glabrous, basal 1/2 of strongly sulcate, sulcate area v-shaped, margined by costate interstriae 3, 5, 6, and sharply carinate interstriae 4, apical 1/2 of declivity subconvex, strongly shagreened; interstriae costate and denticulate to apex; striae punctate, punctures much larger and deeper than those of disc; interstriae impunctate and densely and uniseriate granulate from base to apex. Posterolateral margin carinate to interstriae 7. ***Legs***: procoxae contiguous; prosternal coxal piece bulging. Protibiae obliquely triangular, broadest at apical 1/3; posterior face smooth; apical 1/2 of outer margin with six large socketed denticles, their length as longer than basal width. Mesotibiae flattened, outer margins evenly rounded with nine large socketed denticles.

##### Etymology.

L. *carinatus* = keeled. In reference to the profoundly large carinae on the declivity. An adjective.

##### Distribution.

China (Yunnan).

##### Host plants.

This species is known from *Castanopsis indica* (Fagaceae).

##### Remarks.

The holotype is missing the antennal funicles and club and metatibiae. Locality labels on the holotype are in Chinese and were translated by You Li. An English locality label has been placed on the specimen below the original locality labels.

#### Coptodryas concinna

Taxon classificationAnimaliaColeopteraCurculionidae

(Beeson, 1930)

Fig. 35G, H, L

Xyleborus concinnus Beeson, 1930: 214.
Coptodryas concinnus (Beeson): Wood 1989: 171.
Xyleborus flexicostatus Schedl, 1942c: 31. Synonymy: Kalshoven 1959b: 152; Wood 1989: 171.

##### Type material.

***Holotype****Xyleborus concinnus* (NHMUK), ***paratype*** (FRI, 1; NHMUK, 1). ***Holotype****Xyleborus flexicostatus* (NHMW).

##### New records.

China: Hong Kong, Tai Po Kau, vi.2017, J. Skelton (MSUC, 1).

##### Diagnosis.

2.3 mm long (mean = 2.3 mm; n = 4); 2.09–2.3× as long as wide. This species is distinguished by the dense tuft of setae along the elytral base extending to interstriae 4; body glabrous except for pronotal and elytral bases; striae and interstriae uniseriate punctate, elytral disc strongly shiny; declivity shagreened; declivity bisulcate, interstriae 2 and 3 moderately depressed, interstriae 4–7 carinate, interstriae 4 strongly tumescent and sharply carinate from base to apical 1/4; declivital posterolateral margin carinate to interstriae 7; protibiae distinctly triangular; pronotum rounded, robust (type 5) when viewed laterally and rounded (type 1) when viewed dorsally.

##### Similar species.

*Coptodryas amydra*, *C. carinata*, *C. elegans*, *C. nudipennis*.

##### Distribution.

China* (Hong Kong), India (West Bengal), Indonesia (Java), Myanmar, Thailand.

##### Host plants.

Recorded from *Albizia* (Fabaceae), *Camellia* (Theaceae), *Dimocarpus* (Sapindaceae) and *Lansium* (Meliaceae) (Beeson 1930; Kalshoven 1959b; Maiti and Saha 2004; Beaver et al. 2014).

##### Remarks.

Records of *Coptodryas elegans* (Sampson) in Beaver et al. (2014) should be referred to this species.

#### Coptodryas confusa

Taxon classificationAnimaliaColeopteraCurculionidae

Hopkins, 1915

Fig. 36A, B, I

Coptodryas confusa Hopkins, 1915a: 54.
Xyleborus cryphaloides Schedl, 1942a: 191. Synonymy: Wood and Bright 1992: 823.

##### Type material.

***Holotype****Coptodryas confusa* (NMNH).

##### Diagnosis.

1.8–2.2 mm long (mean = 2.18 mm; n = 5); 2.0–2.25× as long as wide. This species can be identified from all other species in the region by its unique mycangia that include both typical elytral mycangia with conspicuous medial tufts of setae and a pair of pit mycangia located near the pronotal base. In addition, the protibiae have evenly rounded outer margins; elytral strial and interstrial punctures confused; interstriae tuberculate; elytra setose; pronotum tall (type 2) when laterally viewed; and pronotum anterior margin unarmed by a row of serrations.

##### Similar species.

*Coptodryas bella*.

##### Distribution.

Brunei, East & West Malaysia, Philippines, Thailand.

##### Host plants.

Apparently highly host-selective and recorded only from trees of the family Dipterocarpaceae (Browne 1961b; Wood and Bright 1992).

##### Remarks.

Browne (1961b) notes that the species attacks trees of any size down to approximately 5 cm diameter, and has been known to attack newly sawn, unseasoned boards in a sawmill.

**Figure 36.**
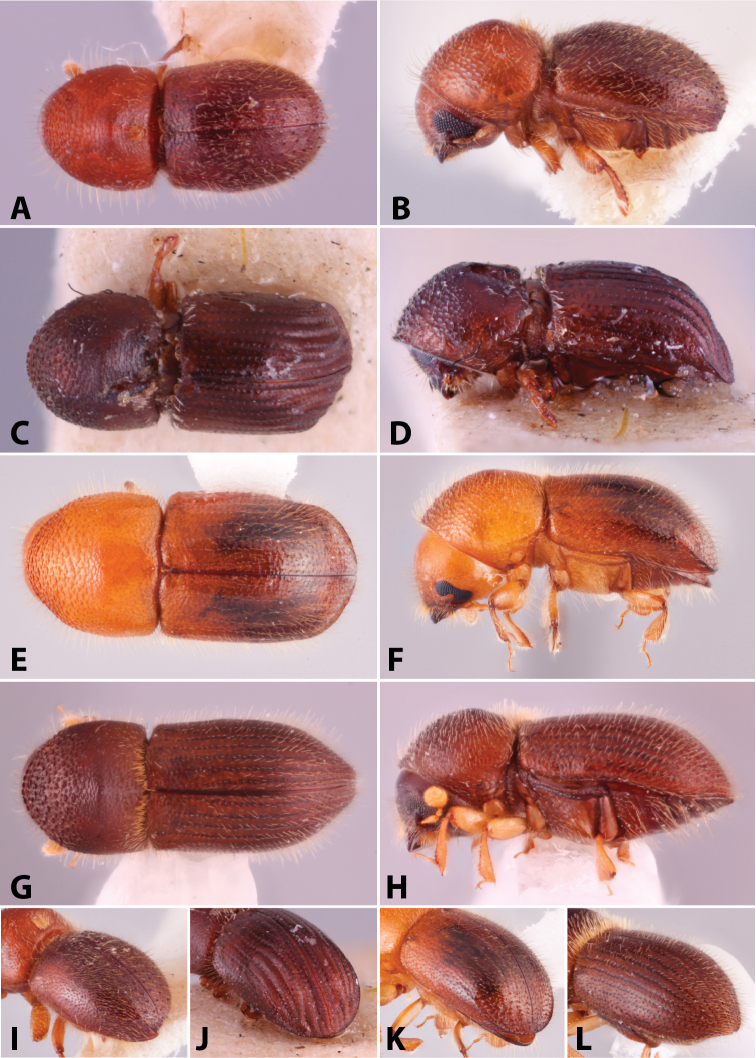
Dorsal, lateral and declivital view of *Coptodryas confusa*, 1.8–2.2 mm (**A, B, I**), *C. elegans* syntype, 2.1–2.4 mm (**C, D, J**), *C. inornata* holotype, 3.1–3.2 mm (**E, F, K**), and *C. mus*, 2.3–2.4 mm (**G, H, L**).

#### Coptodryas elegans

Taxon classificationAnimaliaColeopteraCurculionidae

(Sampson, 1923)

Fig. 36C, D, J

Xyleborus elegans Sampson, 1923: 288.
Coptodryas elegans (Sampson): Wood 1989: 171.

##### Type material.

***Syntype*** (NHMUK).

##### New records.

China: 28.iv.1938, *Litchi chinensis* (NMNH, 1).

##### Diagnosis.

2.1–2.4 mm long (mean = 2.29 mm; n = 5); 2.2–2.4× as long as wide. This species is distinguished by the dense tuft of setae along the elytral base extending to interstriae 8; body glabrous except for pronotal and elytral bases; striae and interstriae uniseriate punctate; elytral disc strongly shiny, declivity shagreened; declivity bisulcate, interstriae 2 to striae 3 weakly to moderately depressed, interstriae 4–7 carinate, interstriae 4 moderately tumescent and sharply carinate from base to apical 1/2; declivital posterolateral margin carinate to interstriae 7; protibiae obliquely triangular; pronotum rounded, robust (type 5) when viewed laterally, and rounded (type 1) when viewed dorsally.

##### Similar species.

*Coptodryas concinna*, *C. nudipennis*.

##### Distribution.

China* (no specified province), India (Madhya Pradesh, West Bengal), Indonesia (Java), Vietnam.

##### Host plants.

Recorded from three different families of trees and probably polyphagous (Beaver et al. 2014).

##### Remarks.

Records of this species in Beaver et al. (2014) should be referred to *Coptodryas concinna* (Beeson).

#### Coptodryas inornata sp. nov.

Taxon classificationAnimaliaColeopteraCurculionidae

http://zoobank.org/29DE1A5D-B4A5-4F5B-A798-45DE8356860A

Fig. 36E, F, K

##### Type material.

***Holotype***, female, Vietnam: Dong Nai, Cat Tien N.P., 11.42854, 107.42544, 148 m, 23.ii.2017, VN98, A.I. Cognato, T.A. Hoang, ex 5 cm diameter branches (MSUC). ***Paratypes***, female, as holotype (MSUC, 5; NHMUK, 5; NHMW, 5; NMNH, 5; VMNH, 5).

##### Diagnosis.

3.1–3.2 mm long (mean = 3.14 mm; n = 5); 2.38–2.46× as long as wide. This species is distinguished by the lack of a tuft of setae along the elytral base; declivity rounded; elytra shiny; striae and interstriae distinct; interstrial punctures confused; body lightly setose; antennal club as broad as tall; protibiae distinctly triangular; pronotum basic (type 0) when viewed laterally, basic (type 2) when viewed dorsally; and anterior margin of the pronotum without a row of serrations.

This species strongly resembles *Xylosandrus formosae* which also lacks a distinct mycangial tuft (at the base of the pronotum) and both have triangular protibia. *Coptodryas inornata* is distinguished by the reduced scutellum, antennal club type 3 (as described for genus), and elytral base bisinuate and costate.

##### Similar species.

*Microperus fulvulus*, *Xylosandrus formosae*.

##### Description

**(female).** 3.1–3.2 mm long (mean = 3.14 mm; n = 5); 2.38–2.46× as long as wide. Pronotum, head, antennae, and legs light brown, elytra darker brown. ***Head***: epistoma entire, transverse, with a row of hair-like setae. Frons weakly convex to upper level of eyes, alutaceous, subshiny, punctate, punctures large, shallow, setose; punctures bearing a long, erect hair-like seta. Eyes shallowly emarginate just above antennal insertion, upper part smaller than lower part. Submentum distinctly triangular, flat, flush with genae. Antennal scape regularly thick, as long as club. Pedicel as wide as scape, shorter than funicle. Funicle 4-segmented, segment 1 shorter than pedicel. Club longer than wide, flattened, type 4; segment 1 corneous, small, convex; segment 2 larger than segment 1, narrow, transverse, corneous; segments 1–3 present on posterior face. ***Pronotum***: 0.72× as long as wide. In dorsal view basic and parallel-sided, type 2, sides parallel in basal 2/3, rounded anteriorly; anterior margin without serrations. In lateral view basic, type 0, disc flat, summit at midpoint. Anterior slope with densely spaced, broad asperities, becoming lower and more strongly transverse towards summit. Disc shiny with dense minute punctures, densely setose, setae short erect hair-like, some longer hair-like setae at margins. Lateral margins obliquely costate. Base weakly bisinuate, posterior angles acutely rounded, almost subquadrate. ***Elytra***: 1.4× as long as wide, 1.96× as long as pronotum. Scutellum minute, convex, slightly raised above elytral surface. Elytral mycangium setal tuft of absent. Elytral base bisinuate, edge oblique, humeral angles rounded, parallel-sided in basal 2/3, then rounded to apex. Disc flat, shiny, striae not impressed, with small shallow punctures separated by 2–3 diameters of a puncture, setose, setae short, recumbent, hair-like; interstriae flat, minutely and confusedly punctate, setose, setae 2× as long as strial setae, erect, hair-like. Declivity occupying approximately 2/5 of elytral length, rounded, face convex, strongly shiny; striae flat, setose, setae as described for disc, punctate, punctures similar in size to those of disc; interstriae 1–3 parallel, interstriae densely covered with long, erect hair-like setae; interstriae impunctate, densely and uniformly uniseriately granulate from base to apex, setose, setae as described for disc. Posterolateral margin distinctly carinate to interstriae 7. ***Legs***: procoxae contiguous; prosternal coxal piece short, inconspicuous. Protibiae distinctly triangular, broadest at apical 1/4; posterior face smooth; apical 1/2 of outer margin with six or seven large socketed denticles, their length longer than basal width. Meso- and metatibiae flattened; outer margins evenly rounded with 9–11 and eight large socketed denticles, respectively.

##### Etymology.

L. *inornatus* = unadorned. In reference to the atypical unsculptured declivity. An adjective.

##### Distribution.

Vietnam.

##### Host plants.

Unknown.

#### Coptodryas mus

Taxon classificationAnimaliaColeopteraCurculionidae

(Eggers, 1930)

Fig. 36G, H, L

Xyleborus mus Eggers, 1930: 203.
Microperus mus (Eggers): Saha and Maiti 1984: 3.
Coptodryas mus (Eggers): Wood and Bright 1992: 825.

##### Type material.

***Holotype*** (FRI).

##### New records.

China: Guizhou, Pingtang, 7.vi.1978, Luyi Luo, ex *Carya* sp. (NMNH, 2); as previous except: 5.xii.1978 (NMNH, 1). Vietnam: Cao Bang, 22°36.454'N, 105°52.083'E, 1661 m, 15.iv.2014, VN38, Cognato, Smith, Pham, ex 1–3 cm diameter branch/twigs (MSUC, 1).

##### Diagnosis.

2.3–2.4 mm long (mean = 2.4 mm; n = 5); 2.4–2.67× as long as wide. This species is distinguished by its elongate form with acuminate elytral apex and gradual declivity; elytra shagreened; striae and interstriae distinct, interstrial punctures confused; body moderately setose; antennal club as long as wide; protibiae distinctly triangular; pronotum basic (type 0) when viewed laterally, basic (type 2) when viewed dorsally; and pronotum anterior margin with a row of six equally sized serrations.

##### Similar species.

*Tricosa metacuneolus*.

##### Distribution.

Bangladesh, China* (Guizhou), India (Sikkim, West Bengal), Vietnam*.

##### Host plants.

Recorded from *Gmelina* (Lamiaceae) and *Michelia* (Magnoliaceae) (Maiti and Saha 2004).

#### Coptodryas nudipennis

Taxon classificationAnimaliaColeopteraCurculionidae

(Schedl, 1951)

Fig. 37A, B, E

Xyleborus nudipennis Schedl, 1951a: 63.
Coptodryas nudipennis (Schedl): Hulcr et al. 2007: 579.

##### Type material.

***Holotype*** (NHMW).

##### Diagnosis.

2.0–2.2 mm long (mean = 2.1 mm; n = 5); 2.2–2.22× as long as wide. This species is distinguished by the dense tuft of setae along the elytral base extending to interstriae 6; body glabrous except for pronotal and elytral bases; striae and interstriae uniseriate; elytral disc strongly shiny; declivity shagreened; declivital face subconvex, interstriae 2 and 3 depressed, remaining interstriae slightly tumescent; pronotum rounded and robust, type 5, when viewed laterally, rounded, type 1, when viewed dorsally; and anterior margin of the pronotum without a distinct row of serrations.

##### Similar species.

*Coptodryas amydra*, *C. carinata*, *C. concinna*, *C. elegans*.

##### Distribution.

Indonesia (Java), East & West Malaysia, Sri Lanka, Thailand.

##### Host plants.

Recorded from *Camellia* (Theaceae), *Cinnamomum* (Lauraceae), and *Lansium* (Meliaceae) in Java by Kalshoven (1959b) and probably polyphagous.

**Figure 37.**
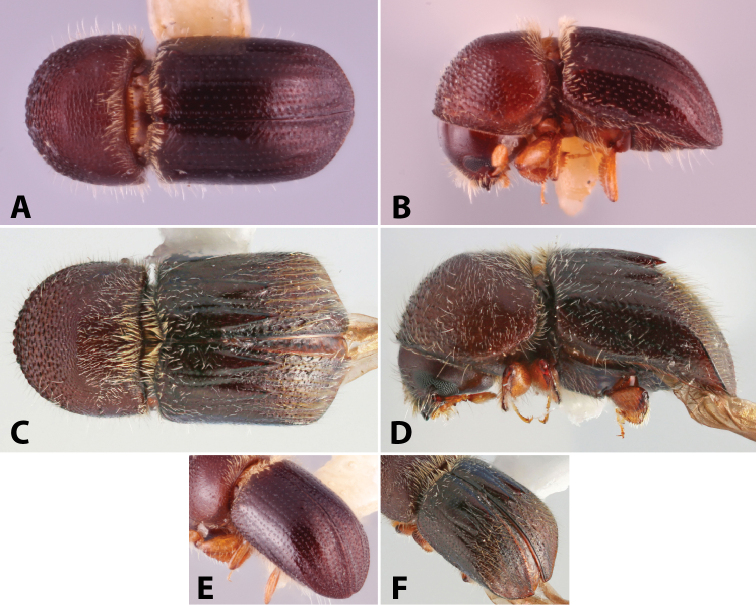
Dorsal, lateral and declivital view of *Coptodryas nudipennis*, 2.0–2.2 mm (**A, B, E**), and *C. quadricostata*, 3.0 mm (**C, D, F**).

#### Coptodryas quadricostata

Taxon classificationAnimaliaColeopteraCurculionidae

(Schedl, 1942)

Fig. 37C, D, F

Xyleborus quadricostatus Schedl, 1942c: 30.
Coptodryas quadricostata (Schedl): Wood and Bright 1992: 826.

##### Type material.

***Lectotype*** (NHMW). Not examined.

##### Diagnosis.

3.0 mm long (n = 1); 2.0× as long as wide (Sittichaya et al. 2019). This species is distinguished by the unique declivital summit bearing four sharp spines that extend beyond the over the declivity.

##### Similar species.

None.

##### Distribution.

‘Borneo’, Indonesia (Java), East & West Malaysia, Thailand.

##### Host plants.

Recorded from *Campnosperma* (Anacardiaceae), *Garcinia* (Clusiaceae), *Shorea leprosula*, *S. parvifolia* (Dipterocarpaceae), and *Elaeocarpus* (Elaeocarpaceae) (Sittichaya et al. 2019).

##### Remarks.

Browne (1961b) notes that the species attacks small branches 1–5 cm in diameter. The gallery system usually encircles the stem and has one or two longitudinal branches in which the larvae develop (Browne 1961b).

### *Cryptoxyleborus* Wood & Bright, 1992

#### Cryptoxyleborus

Taxon classificationAnimaliaColeopteraCurculionidae

Wood & Bright, 1992

Cryptoxyleborus Wood & Bright, 1992: 828.
Cryptoxyleborus Schedl, 1937a: 550. Unavailable name (see Alonso-Zarazaga and Lyal 2009).

##### Type species.

*Cryptoxyleborus naevus* Schedl, 1937a; original designation.

##### Diagnosis.

1.75–4.4 mm, and elongate, 3.0–4.17× as long as wide, with elytral apex attenuate or acuminate. *Cryptoxyleborus* is recognized by the distinctive pit mycangia located on the elytra either near the scutellum or along the base (two species without pit mycaniga); scutellum is on the anterior slope and appears absent when viewed dorsally; protibiae slender and rugose on the posterior face; and procoxae contiguous.

##### Similar genera.

*Fraudatrix*, *Tricosa*, *Xyleborinus*.

##### Distribution.

Occurring in tropical Asia and New Guinea, possibly introduced to Australia.

##### Gallery system.

This consists of an unbranched entrance tunnel leading to a single terminal brood chamber in the longitudinal plane (Browne 1961b). The brood chamber is enlarged by the larvae as they develop.

##### Remarks.

All species of *Cryptoxyleborus* with known hosts only attack trees of the family Dipterocarpaceae (Beaver and Hulcr 2008). Monophyly of *Cryptoxyleborus* is in question (Cognato et al. 2020b).

#### Key to *Cryptoxyleborus* species (females only)

**Table:** 

1	Elytra without mycangial pits (Fig. 38C); antennal funicle 3-segmented; minute, 1.4–2.0 mm	**2**
–	Elytra with mycangial pits on basal slope or near scutellum on dorsal surface (Fig. 38A); antennal funicle 4-segmented; small to large, 2.15–4.4 mm	**3**
2	Larger, 2.0 mm and elongate, 3.3× as long as wide; elytral interstriae reticulate–punctate, punctures confused and very dense at the base of the disc	*** confusus ***
–	Smaller, 1.4 mm and stout, 2.55× as long as wide; elytral interstriae distinctly seriate punctate, punctures not densely placed at the base	*** percuneolus ***
3	Pit mycangia present on dorsal elytral surface near scutellum (Fig. 38A)	**4**
–	Pit mycangia present on basal slope of elytra (Fig. 39C)	**5**
4	Mycangial pits subtriangular; elytral apex truncate when viewed from behind, forming a small approximately oval, impunctate, flattened facet	*** barbieri ***
–	Mycangial pits subcircular; elytral apex attenuate, lacking a flattened, apical facet	*** subnaevus ***
5	Elytral disc with a transverse, saddle-like depression in basal 1/2; interstriae bearing strongly hooked tubercles from basal 1/3 (Fig. 38F)	*** eggersi ***
–	Elytral disc without a transverse, saddle-like depression; interstriae bearing weakly hooked tubercles from at or behind elytral midpoint (Fig. 39A)	**6**
6	Mycangial openings in elytra base comprised of four round pits	*** quadriporus ***
–	Mycangial openings in elytra base comprised of two transverse slits	**7**
7	Smaller, 2.35–2.6 mm; elytral vestiture uniseriate on all discal interstriae (except at base)	*** stenographus ***
–	Larger, 3.2–3.3 mm; elytral vestiture irregularly biseriate on discal interstriae 2–4	*** turbineus ***

#### Cryptoxyleborus barbieri

Taxon classificationAnimaliaColeopteraCurculionidae

Schedl, 1953

Fig. 38A, B, I

Cryptoxyleborus barbieri Schedl, 1953a: 128.

##### Type material.

***Lectotype*** (NHMW).

##### Diagnosis.

2.15–2.5 mm long (mean = 2.35 mm; n = 4); 3.31–3.57× as long as wide. This species is distinguished by the pair of subtriangular mycangial pits close to the scutellum on the dorsal elytral surface; antennal funicle 4-segmented; and elytral apex truncate when viewed from behind, forming a small approximately oval, impunctate, flattened facet.

##### Similar species.

*Cryptoxyleborus subnaevus*.

##### Distribution.

Brunei, Vietnam.

##### Host plants.

Unknown.

**Figure 38.**
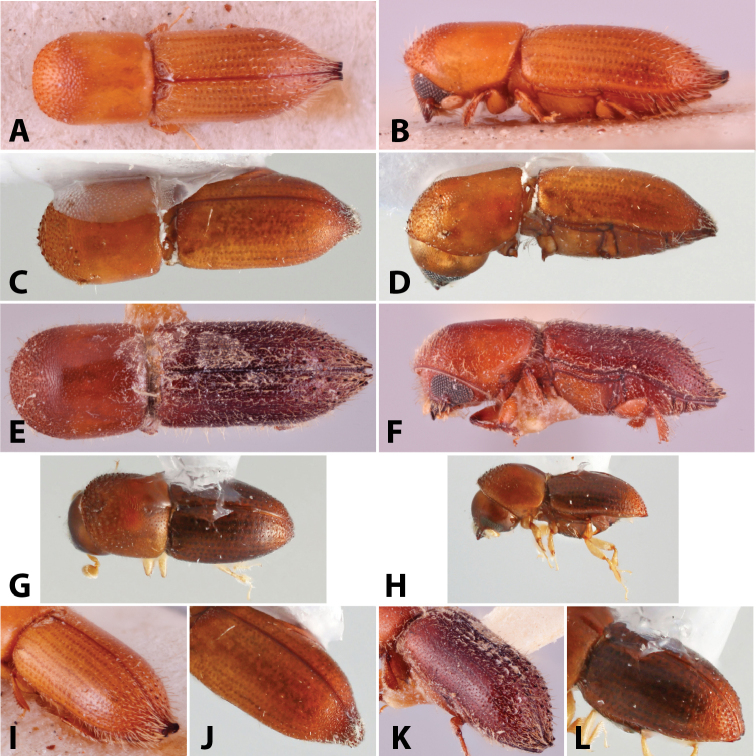
Dorsal, lateral and declivital view of *Cryptoxyleborus barbieri* lectotype, 2.15–2.5 mm (**A, B, I**), *C. confusus*, 2.0 mm (**C, D, J**), *C. eggersi* paralectotype, 3.5–4.4 mm (**E, F, K**), and *C. percuneolus*, 1.4 mm (**G, H, L**).

#### Cryptoxyleborus confusus

Taxon classificationAnimaliaColeopteraCurculionidae

Browne, 1950

Fig. 38C, D, J

Cryptoxyleborus confusus Browne, 1950: 644.

##### Type material.

***Holotype*** (NHMUK).

##### Diagnosis.

2.0 mm long (n = 1); 3.3× as long as wide (Sittichaya et al. 2019). This species is distinguished by its small size; elytral pit mycangia absent; antennal funicle 3-segmented; elytral interstriae reticulate–punctate, punctures confused and very densely placed at the base of the disc.

##### Similar species.

*Cryptoxyleborus percuneolus*, *Fraudatrix simplex*.

##### Distribution.

Brunei, Indonesia (Sumatra), East & West Malaysia, Thailand.

##### Host plants.

Only recorded from *Shorea* (Dipterocarpaceae) (Browne 1961b; Beaver and Hulcr 2008).

##### Remarks.

Browne (1961b) notes that the gallery system differs from the usual pattern found in *Cryptoxyleborus*. In this species a surface brood chamber is excavated between bark and wood in which most of the larvae develop. However, there are also more deeply penetrating tunnels into the wood. Brood sizes ranged from 18–39 (Browne 1961b).

#### Cryptoxyleborus eggersi

Taxon classificationAnimaliaColeopteraCurculionidae

Schedl, 1936

Fig. 38E, F, K

Cryptoxyleborus eggersi Schedl, 1936c: 60.
Cryptoxyleborus dryobalanopsis Schedl, 1942a: 184. Synonymy: Bright and Skidmore 1997: 4, 175.
Xyleborus eggersianus Schedl, 1960b: 110 (unnecessary new name for X. eggersi (Schedl 1936 nec Beeson 1930)).

##### Type material.

***Lectotype****Cryptoxyleborus eggersi* (NMNH), ***paralectotype*** (NHMW).

##### New records.

Laos: Kham Mouan, Ban Khoun Ngeun, 18°07'N, 104°29'E, ~ 200 m, 24–29.iv.2001, P. Pacholátko (RABC, 1). Philippines: v.1958, H. Milliron (BPBM, 1).

##### Diagnosis.

3.5–4.4 mm long (mean = 3.85 mm; n = 4); 3.14–3.5× as long as wide. This species is distinguished by its large size; large and broad mycangial pits on the basal slope of elytra; elytral disc with a transverse saddle-like depression; declivital interstriae bearing strongly hooked tubercles from basal 1/3; antennal funicle 4-segmented.

##### Similar species.

*Cryptoxyleborus quadriporus*, *C. stenographus*, *C. turbineus*.

##### Distribution.

Brunei, Laos*, East & West Malaysia, Philippines, Vietnam.

##### Host plants.

Recorded from *Balanocarpus*, *Dipterocarpus*, *Dryobalanops* and *Shorea* (Dipterocarpaceae) (Beaver and Hulcr 2008).

##### Remarks.

A lectotype for the species was designated by Anderson and Anderson (1971: 12) as well as by Schedl who designated it a “holotype” (1979: 87). The citation by Schedl is invalid and unnecessary. Wood and Bright (1992: 828) mistakenly cited the Schedl designation. The lectotype is in NMNH and a paralectotype is in NHMW.

#### Cryptoxyleborus percuneolus

Taxon classificationAnimaliaColeopteraCurculionidae

(Schedl, 1951)

Fig. 38G, H, L

Xyleborus percuneolus Schedl, 1951a: 85.
Xyleborinus percuneolus (Schedl): Wood and Bright 1992: 809.
Cryptoxyleborus percuneolus (Schedl): Beaver and Hulcr 2008: 145.

##### Type material.

***Lectotype*** (NHMW).

##### Diagnosis.

1.4 mm long (n = 1); 2.55× as long as wide (Sittichaya et al. 2019). This species is distinguished by its minute size; elytral pit mycangia absent; antennal funicle 3-segmented; elytral interstriae distinctly seriate punctate, without very densely placed punctures at the base.

##### Similar species.

*Cryptoxyleborus confusus*, *Fraudatrix simplex*.

##### Distribution.

Indonesia (Java), East Malaysia, Thailand.

##### Host plants.

No host records are known but hosts are presumably similar to other *Cryptoxyleborus* which are specific to Dipterocarpaceae (Beaver and Hulcr 2008).

##### Remarks.

One gallery system investigated consisted of an unbranched entrance tunnel leading to a single terminal brood chamber enlarged in the longitudinal plane, with multiple tunnels extending further into the wood (Beaver and Hulcr 2008).

#### Cryptoxyleborus quadriporus

Taxon classificationAnimaliaColeopteraCurculionidae

Beaver, 1990

Fig. 39A, B, I

Cryptoxyleborus quadriporus Beaver, 1990: 281.

##### Type material.

***Holotype*** (NHMUK).

##### New records.

Thailand: Chiang Mai, Fang, 12–19.iv.1958, T.C. Maa (BPBM, 1).

##### Diagnosis.

3.2–3.3 mm long (n = 2); 3.2–3.4× as long as wide. This species is distinguished by the two distinctive pairs of round mycangial pits along the basal slope of elytra; and antennal funicle 4-segmented.

##### Similar species.

*Cryptoxyleborus eggersi*, *C. stenographus*, *C. turbineus*.

##### Distribution.

Thailand.

##### Host plants.

Unknown.

##### Remarks.

Only two specimens of this species are known, both from Chiang Mai, Thailand.

**Figure 39.**
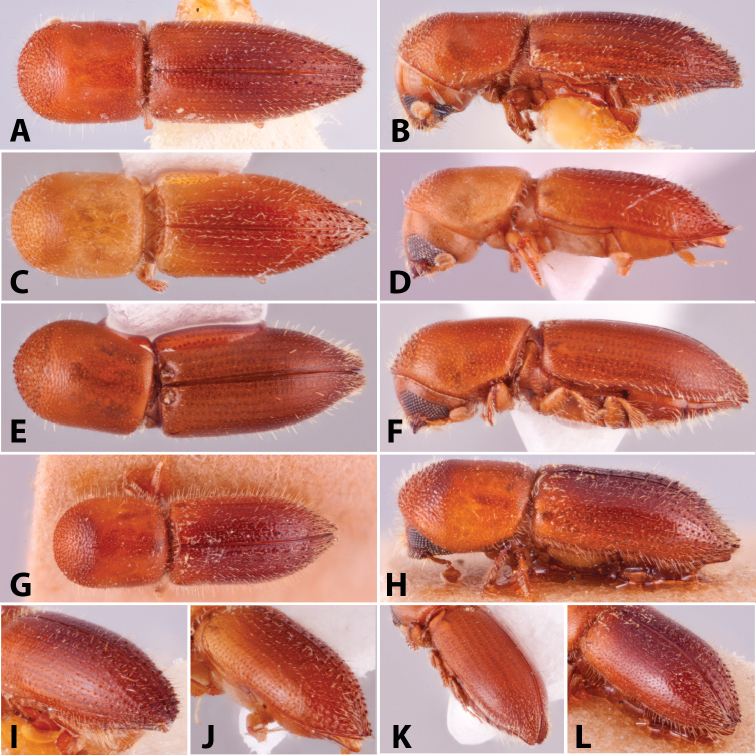
Dorsal, lateral and declivital view of *Cryptoxyleborus quadriporus* holotype, 3.2–3.3 mm (**A, B, I**), *C. stenographus*, 2.35–2.6 mm (**C, D, J**), *C. subnaevus*, 2.2–2.7 mm (**E, F, K**), and *C. turbineus*, 3.2–3.3 mm (**G, H, L**).

#### Cryptoxyleborus stenographus

Taxon classificationAnimaliaColeopteraCurculionidae

(Schedl, 1971)

Fig. 39C, D, J

Xyleborus stenographus Schedl, 1971b: 383.
Cryptoxyleborus stenographus (Schedl): Wood and Bright 1992: 829.

##### Type material.

***Holotype*** (NHMW).

##### New records.

Laos: Sekong, ~ 12 km S Sekong, Taofaek waterfall, 15°14.7'N, 106°45.1'E, 118 m, at light, 12.v.2010, J. Hájek, (MHNP, 1).

##### Diagnosis.

2.35–2.6 mm long (mean = 2.46 mm; n = 4); 3.0–3.33× as long as wide. This species is distinguished by the elytral apex acuminate; two mycangial pits broad and narrow on basal slope of elytra; declivital interstriae denticulate; and antennal funicle 4-segmented.

This species most closely resembles *C. turbineus* and is distinguished by the smaller size and by the elytral vestiture uniseriate on all discal interstriae (except at base).

##### Similar species.

*Cryptoxyleborus eggersi*, *C. quadriporus*, *C. turbineus*.

##### Distribution.

Indonesia (Sumatra), Laos*, Thailand.

##### Host plants.

Unknown.

#### Cryptoxyleborus subnaevus

Taxon classificationAnimaliaColeopteraCurculionidae

Schedl, 1937

Fig. 39E, F, K

Cryptoxyleborus subnaevus Schedl, 1937a: 552.

##### Type material.

***Lectotype*** (NHMW).

##### New records.

Laos: Vientiane, Gi Sion vill. De Tha Ngone, 28.ii.1965, J.L. Gressitt, light trap (BPBM, 1); as previous except: 26 km SW of Ban Me Thuot, 855 m, 20.v.1960, light trap (BPBM, 1). Vietnam: Cuc Phuong N.P., 20°15.586'N, 105°42.320'E, 147 m, 30.iv–1.v.2005, A. Kun (HNHM, 1).

##### Diagnosis.

2.2–2.7 mm long (mean = 2.46 mm; n = 5); 3.29–4.17× as long as wide. This species is distinguished by the pair of subcircular mycangial pits close to the scutellum on the dorsal elytral surface; antennal funicle 4-segmented; and attenuate elytral apex.

##### Similar species.

*Cryptoxyleborus barbieri*.

##### Distribution.

Australia, ‘Borneo’, Brunei, India (Kerala), Indonesia (Kalimantan, Sumatra), Laos*, East & West Malaysia, Myanmar, New Guinea, Philippines, Thailand, Vietnam*.

##### Host plants.

Recorded from *Dipterocarpus*, *Dryobalanops*, *Pentacme*, and *Shorea* (Dipterocarpaceae) (Beaver and Hulcr 2008).

#### Cryptoxyleborus turbineus

Taxon classificationAnimaliaColeopteraCurculionidae

(Sampson, 1923)

Fig. 39G, H, L

Xyleborus turbineus Sampson, 1923: 288.
Cryptoxyleborus turbineus (Sampson): Schedl 1937a: 551.

##### Type material.

***Syntype*** (NHMUK).

##### Diagnosis.

3.2–3.3 mm long (mean = 3.26 mm; n = 4); 3.2–3.3× as long as wide. This species is distinguished by the elytral apex acuminate; two mycangial pits broad and narrow on basal slope of elytra; declivital interstriae denticulate; and antennal funicle 4-segmented.

This species most closely resembles *C. stenographus* and is distinguished by the larger size and elytral vestiture irregularly biseriate on discal interstriae 2–4.

##### Similar species.

*Cryptoxyleborus eggersi*, *C. quadriporus*, *C. stenographus*.

##### Distribution.

India (Jharkhand, Odisha, West Bengal), Myanmar, Philippines, Thailand, Vietnam.

##### Host plants.

Recorded from *Pentacme* and *Shorea* (Dipterocarpaceae) (Beeson 1930).

### *Cyclorhipidion* Hagedorn, 1912

#### Cyclorhipidion

Taxon classificationAnimaliaColeopteraCurculionidae

Hagedorn, 1912

Cyclorhipidion Hagedorn, 1912b: 355.
Terminalinus Hopkins, 1915a: 10. Synonymy: Wood and Bright 1992: 697.
Notoxyleborus Schedl, 1934b: 84. Synonymy: Smith et al. 2020: 39.
Kelantanius Nunberg, 1961: 621. Synonymy: Wood 1986: 83.

##### Type species.

*Cyclorhipidion pelliculosum* Hagedorn, 1912b; original designation.

##### Diagnosis.

1.7–5.0 mm, very stout to very elongate (2.19–3.67× as long as wide) with elytral apex entire and variable declivital forms. *Cyclorhipidion* is a morphologically variable genus. However species can largely be distinguished by their distinctive appearance with most of body covered with dense pubescence and very abundant minute punctures, elytral disc with confused interstrial punctures, pronotum and elytra rounded, typically with no conspicuous edges or carinae, antennal club flattened, type 3 (types 4 and 5 rare), visible scutellum, protibiae semi-circular with evenly rounded outer edge (rarely obliquely triangular), procoxae contiguous and lack of mycangial tufts. Several species have obliquely truncate or truncate declivities.

*Fraudatrix* and *Truncaudum* are very similar to small *Cyclorhipidion* species and are distinguished by the obliquely truncate type 2 antennal club. *Tricosa* is also similar and is distinguished by the distinctly triangular protibiae.

##### Similar genera.

*Anisandrus*, *Dryoxylon*, *Fraudatrix*, *Tricosa*, *Truncaudum*.

##### Distribution.

Occurring in temperate and tropical forests worldwide with the exception of South America. Three species have been introduced to the United States. (Hoebeke et al. 2018).

##### Gallery system.

Usually consists of an unbranched entrance tunnel leading to a single narrow brood chamber, which may be quite large, in the longitudinal plane (Browne 1961b; Hulcr and Cognato 2013). However, in *C. perpilosellum*, the gallery system has a few branches in the horizontal plane with small, irregular brood chambers (Browne 1961b).

##### Remarks.

Some species of *Cyclorhipidion* have a strong host preference for trees of the family Fagaceae. These species occur especially in areas where this family is abundant in the forests (Beaver et al. 2014).

#### Key to *Cyclorhipidion* species (females only)

**Table:** 

1	Elytral disc with a median shallow saddle-like impression (Fig. 42F)	**2**
–	Elytral disc without a median shallow saddle-like impression (Fig. 42H)	**3**
2	Elytral apex armed by two or three pairs of large sharp spines, their length longer than basal width; declivital face steep, flat, unarmed by tubercles, one or two small granules on declivital interstriae 1 and 3 near upper margin	*** miyazakiense ***
–	Elytral apex armed by two pairs moderate teeth, their apices blunt, and their length no longer than basal width; declivital face steeply convex, tuberculate and granulate	*** armiger ***
3	Declivity moderately to strongly sulcate between suture and interstriae 3 (Fig. 42C); interstriae 1 unarmed	**4**
–	Declivity convex or flattened; interstriae 1 armed by granules, denticles or tubercles (Fig. 42G)	**5**
4	Declivity sulcate to interstriae 3; interstriae 3 bearing five tubercles along its length; smaller, 2.1–2.3 mm	*** japonicum ***
–	Declivity sulcate to interstriae 4; interstriae 3 only armed by two granules near base; larger, 2.5 mm	*** neocavipenne ***
5	Anterior margin of pronotum with a row of serrations (Fig. 45A)	**6**
–	Anterior margin of pronotum without a row of serrations (Fig. 45G)	**8**
6	Antennal club circular, type 5, lacking sutures on anterior and posterior faces (Fig. 3)	*** sisyrnophorum ***
–	Antennal club wider than long, type 3, with three sutures visible on anterior face and 2–3 sutures visible on posterior face (Fig. 3)	**7**
7	Anterior margin of pronotum serrate with serrations on a short continuously elevated recurved carina; posterolateral margin of elytra carinate to interstriae 7; larger, 5.5 mm	*** vigilans ***
–	Anterior margin of pronotum serrate and without a carina; posterolateral margin of elytra rounded; smaller, 3.5–4.1 mm	*** pruinosum ***
8	Eyes almost entire; declivity with scale-like setae	*** fouqueti ***
–	Eyes moderately to deeply emarginate; declivity with hair-like setae	**9**
9	Declivital interstriae 2 armed, bearing granules, denticles or tubercles	**11**
–	Declivital interstriae 2 unarmed by granules, denticles or tubercles (excluding apical margin)	**22**
10	Declivity obliquely truncate or truncate, separation between disc and declivity abrupt (Fig. 45G)	**11**
–	Declivity variably rounded (gradually, evenly or steeply), separation between disc and declivity gradual (Fig. 44E)	**15**
11	Declivity truncate, declivital margins forming a costate and tuberculate circumdeclivital ring (Fig. 45G)	**12**
–	Declivity obliquely truncate, declivital margins costate and granulate or tuberculate to interstriae 7, never forming a circumdeclivital ring (Fig. 40C)	**13**
12	Declivital face rugose and coarsely sculptured, distinctly sulcate on basal 1/2, striae 1 more deeply impressed than striae 2 or 3; interstriae 1 inflated on apical 1/3 and interstriae 2 and 3 flat	***truncaudinum* sp. nov.**
–	Declivital face smooth, feebly sulcate on basal 1/4; striae clearly, uniformly impressed; and interstriae inflated	*** umbratum ***
13	Declivity strongly shiny; pronotum wider than long; more elongate, 3.13× as long as wide	***amputatum* sp. nov.**
–	Declivity shagreened and dull; pronotum longer than wide; stouter, 2.54–2.83× as long as wide	**14**
14	Pronotum subquadrate from dorsal view (type 3); declivital interstrial punctures replaced by a single row of tubercles.	***muticum* sp. nov.**
–	Pronotum basic from dorsal view (type 2) with rounded anterior margin; declivital interstriae densely covered in multiseriate rows of tubercles	*** circumcisum ***
15	Base of elytral disc with seriate striae and confused interstriae; strial punctures larger than interstrial punctures (Fig. 41C)	**16**
–	Base of elytral disc with confused striae and interstriae; strial punctures as large as interstrial punctures (Fig. 44C)	**19**
16	Declivity sulcate between suture and striae 1; tubercles on interstriae 2 larger than those of interstriae 1 and 3; pronotal disc coarsely punctate; larger size, 4.1–4.2 mm	*** ohnoi ***
–	Declivity convex; tubercles on interstriae 1 larger than those of interstriae 2 and 3; pronotal disc finely punctate; smaller size, 2.5–3.1 mm	**17**
17	Declivity very steeply rounded; granules present on no more than apical 1/3 of declivity	***tenuigraphum* , in part**
–	Declivity gradually rounded; granules present along entire length of interstriae 2	**18**
18	Elytral apex and posterolateral margin armed with alternating spines and denticles, a single spine on each interstria and a smaller denticle on each stria from suture to interstriae 7; elytral interstriae tuberculate with three large equally spaced tubercles along interstriae 1 and 3, those of interstriae 3 smaller	***denticauda* sp. nov.**
–	Elytral apex and posterolateral margin granulate; declivital interstriae armed with a row of moderately spaced uniseriate granules	*** pilipenne ***
19	Declivital slope strongly and evenly rounded (Fig. 44B); smaller, 2.5–3.0 mm, and stout, 2.08–2.31× as long as wide	*** perpilosellum ***
–	Declivital slope gentle, gradual (Fig. 46H); larger, 3.25–4.1 mm, and elongate, 2.58–2.73× as long as wide	**20**
20	Posterolateral margin of elytra granulate; declivital striae weakly impressed; granules on interstriae 1–3 approximately equal in size; larger, 3.9–4.1 mm	***petrosum* sp. nov.**
–	Posterolateral margin of elytra costate and granulate; declivital striae not impressed, punctures small, indistinct; granules on interstriae 1 larger than those of 2 or 3; smaller, 3.25 mm	*** xyloteroides ***
21	Declivity truncate, surrounded by circumdeclivital carina; interstriae 3 unarmed; anterior margin of pronotum subquadrate; larger, 4.2 mm	***amasoides* sp. nov.**
–	Declivity rounded or obliquely truncate; interstriae 3 armed by granules, denticles or tubercles; anterior margin of pronotum rounded; smaller, 1.65–4.1 mm	**22**
22	Declivity at least 1/3 of total elytral length evenly or gradually rounded (Fig. 44H)	**23**
–	Declivity approximately 1/4 of total elytral length, very steep (Fig. 43H)	**24**
23	Declivity evenly rounded and convex; posterolateral margin of elytra rounded and granulate; declivital interstriae 1 with two large tubercles in median area; submentum deeply depressed below genae; smaller, 2.1 mm	***obesulum* sp. nov.**
–	Declivity gradually rounded; posterolateral margin of elytra carinate and granulate to interstriae 7; declivital interstriae 1 armed by a large denticle near the base and a small spine near the apex with the area in between appearing concave; submentum not depressed, flat, flush with genae; larger, 2.7–3.5 mm	*** pruinosulum ***
24	Declivital interstriae 1 with one row of seriate setae	**25**
–	Declivital interstriae 1 with two or three rows of confused setae	**26**
25	Declivity obliquely truncate and flattened; pronotal anterior slope short, pronotal summit approximately at apical 25%; smaller, 1.65–1.8 mm	***xeniolum* sp. nov.**
–	Declivity steeply rounded and weakly convex or concave (atypical and rare individuals); pronotal anterior slope moderate, pronotal summit approximately at apical 35–45%; larger, larger, 1.9–2.2 mm	*** bodoanum ***
26	Declivital interstriae 2 setae uniseriate, in one row on apical 1/2	**27**
–	Declivital interstriae 2 setae biseriate, confused on apical 1/2	**28**
27	Declivital interstriae 1 with three rows of confused setae; larger, 3.2–3.5 mm	*** pelliculosum ***
–	Declivital interstriae 1 with two rows of confused setae; smaller, 2.75–3.0 mm	*** inarmatum ***
28	Declivital interstriae 1 with three rows of confused setae	*** distinguendum ***
–	Declivital interstriae 1 with two rows of confused setae	***tenuigraphum* , in part**

#### Cyclorhipidion amasoides sp. nov.

Taxon classificationAnimaliaColeopteraCurculionidae

http://zoobank.org/50F37A06-A04F-41D5-8CC7-DFD64ACB9525

Fig. 40A, B, I

##### Type material.

***Holotype***, female, India: Arunachal Pradesh, Hunli vicinity, 28°19'32"N, 95°57'31"E, 1300 ± 100 m, 26.v.2012, L. Dembický (ZFMK).

##### Diagnosis.

4.2 mm long (n = 1); 2.8× as long as wide. This species is distinguished by the large size; declivity truncate; pronotum subquadrate from dorsal view (type 3); declivital face with three striae; declivity strongly tumescent from apex to basal 1/4 and laterally from sutural margin to striae 2; declivital interstriae 1 coarsely granulate, interstriae 2–4 minutely punctate, and surface shiny.

##### Similar species.

*Amasa* spp., *Cyclorhipidion amputatum*, *C. circumcisum*, *C. muticum*, *C. truncaudinum*, *C. umbratum*, all of which are large and have an obliquely truncate or truncate declivity.

##### Description

**(female).** 4.2 mm long (n = 1); 2.8× as long as wide. Head and body dark red-brown. Legs and antennae light brown. ***Head***: epistoma entire, transverse, with a row of hair-like setae. Frons weakly convex to upper level of eyes; surface shiny, impunctate, alutaceous, feebly rugose. Eyes shallowly emarginate just above antennal insertion, upper part smaller than lower part. Antennal scape regularly thick, as long as club. Pedicel as wide as scape, shorter than funicle. Funicle 4-segmented, segment 1 shorter than pedicel. Club approximately circular and flat, type 3; segment 1 corneous, transverse on anterior face, occupying approximately basal 1/4; segment 2 broad, corneous; segments 1 and 2 present on posterior face. ***Pronotum***: 0.89× as long as wide. In dorsal view subquadrate, sides convex, type 3, narrowly rounded anteriorly; anterior margin without serrations. In lateral view elongate with disc longer than anterior slope, type 7, disc flat, summit at apical 2/5. Anterior slope shagreened, with densely spaced, fine, narrow asperities, becoming lower and more strongly transverse towards summit, bearing long, fine, semi-recumbent hair-like setae. Disc shiny, densely, finely punctate, glabrous. Lateral margins obliquely costate. Base transverse, posterior angles broadly rounded. ***Elytra***: 1.74× as long as wide, 1.9× as long as pronotum. Scutellum large, broad, linguiform, flush with elytra, flat, shiny. Elytral base transverse, edge oblique, humeral angles rounded, parallel-sided in basal 4/5, then sharply angulate to apex. Disc ascending posteriorly, shiny, striae and interstriae densely setose, setae short, recumbent, hair-like; striae and interstriae laterally diverging from base to declivital summit; striae not impressed, punctures separated by 1–4 diameters of a puncture; interstriae flat, punctate, punctures minute, 1/2 size of strial punctures, strongly confused. Declivity occupying 1/3 of elytra, truncate, face strongly tumescent from apex to basal 1/4 and laterally from sutural margin to striae 2, strongly shiny; three striae present, striae not impressed, equidistant, strial punctures shiny, moderately sized, shallow, much larger than on disc, punctures irregular, variably spaced by 1–4 diameters of a puncture; interstriae setose, setae sparse, short, erect hair-like; interstriae 1 impunctate, coarsely granulate, granules increasing in size apically, interstriae 2–4 punctate, punctures minute, strongly confused, less than 1/2 size of strial punctures. Posterolateral margin forming a circumdeclivital carina; carina feebly rugose, setose, setae short, fine. ***Legs***: procoxae contiguous. Protibiae semi-circular with evenly rounded outer edge, broadest at apical 1/3; posterior face smooth; apical 1/3 of outer margin with nine moderate socketed denticles, their length approximately as long as basal width. Meso- and metatibiae broad, flattened; outer margin evenly rounded with 13 and 11 moderate socketed denticles, respectively.

##### Etymology.

In reference to the likeness to *Amasa*. Noun in apposition.

##### Distribution.

India (Arunachal Pradesh).

##### Host plants.

Unknown.

##### Remarks.

The holotype is card mounted and ventral characters could not be examined. This species exhibits strong morphological convergence with *Amasa*. It is distinguished from *Amasa* by the type 3 antennal club with transverse sutures, subquadrate pronotum (type 3) that lacks serrations on anterior margin and the elytral disc densely setose with strial and interstrial punctures minute and strongly confused.

**Figure 40.**
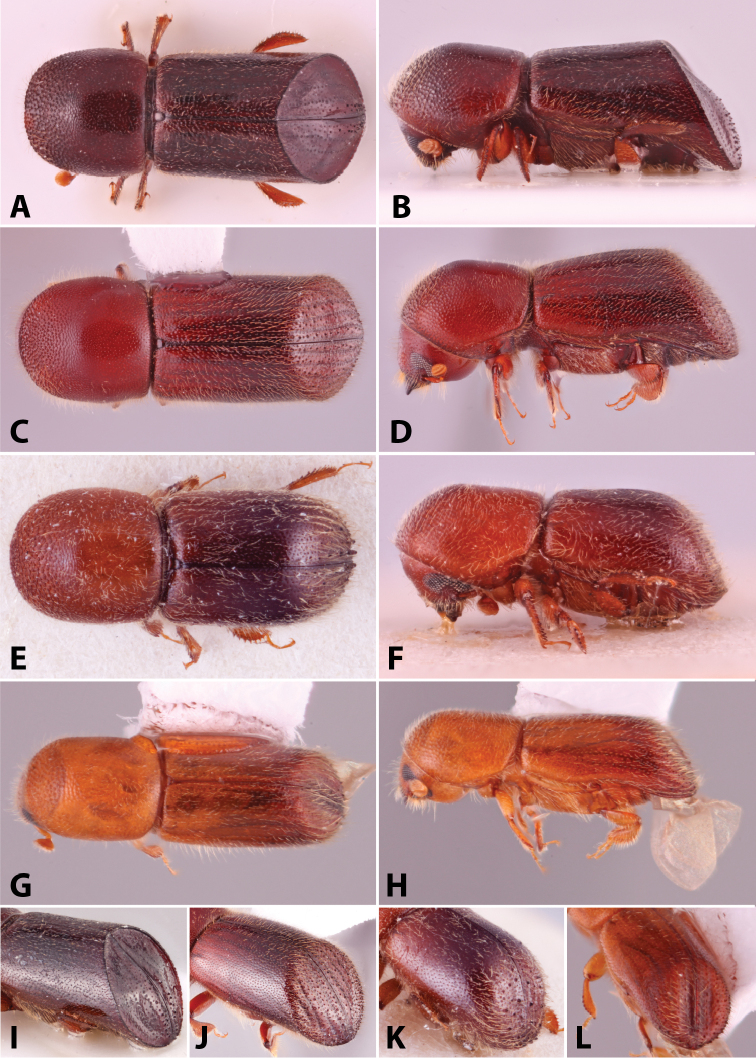
Dorsal, lateral and declivital view of *Cyclorhipidion amasoides* holotype, 4.2 mm (**A, B, I**), *C. amputatum* holotype, 5.0 mm (**C, D, J**), *C. armiger* lectotype, 2.6–3.3 mm (**E, F, K**), and *C. bodoanum*, 1.9–2.2 mm (**G, H, L**).

#### Cyclorhipidion amputatum sp. nov.

Taxon classificationAnimaliaColeopteraCurculionidae

http://zoobank.org/8B0B3F73-6E9E-4E7D-A89F-E5AA01B718BA

Fig. 40C, D, J

##### Type material.

***Holotype***, female, Vietnam: Cao Bang, 22°36.454'N, 105°52.083'E, 1661 m, 15.iv.2014, VN39, Cognato, Smith, Pham, ex 3–6 cm branches '(MSUC). ***Paratype***, female, as holotype except: 22°36.454'N, 105°52.083'E, 1661 m, 17.iv.2014, VN40, ex 3 pieces “firewood” (VMNH).

##### Diagnosis.

5.0 mm long (n = 1); 3.13× as long as wide. This species is distinguished by the large size; obliquely truncate declivity with rounded margins; pronotum wider than long and subquadrate from dorsal view (type 3); declivital interstriae punctures replaced by sparse, small, confused tubercles; declivital strial punctures large, distinct; declivital face appearing convex and strongly shiny; declivital striae clearly, uniformly impressed and interstriae inflated on apical 1/2.

##### Similar species.

*Cyclorhipidion amasoides*, *C. circumcisum*, *C. muticum*, *C. truncaudinum*, *C. umbratum*, all of which are large and have an obliquely truncate or truncate declivity.

##### Description

**(female).** 5.0 mm long (n = 1); 3.13× as long as wide. Body, antennae, and legs red. ***Head***: epistoma entire, transverse, with a row of hair-like setae. Frons weakly convex to upper level of eyes; surface subshiny, impunctate, alutaceous, rugose. Eyes shallowly emarginate just above antennal insertion, upper part smaller than lower part. Submentum narrow, triangular, deeply impressed. Antennal scape regularly thick, as long as club. Pedicel as wide as scape, shorter than funicle. Funicle 4-segmented, segment 1 as long as pedicel. Club approximately circular and flat, type 3; segment 1 corneous, transverse on anterior face, occupying approximately basal 2/5; segment 2 narrow, corneous; segments 1 and 2 present on posterior face. ***Pronotum***: 0.96× as long as wide. In dorsal view subquadrate, sides convex, type 3, narrowly rounded anteriorly; anterior margin without serrations. In lateral view tall, type 2, disc flat, summit at midpoint. Anterior slope shagreened, with densely spaced, fine, narrow asperities, becoming lower and more strongly transverse towards summit, bearing long, fine, semi-recumbent, hair-like setae. Disc subshiny, alutaceous, densely, finely punctate, finely setose, setae short, erect, hair-like. Lateral margins obliquely costate. Base transverse, posterior angles broadly rounded. ***Elytra***: 1.81× as long as wide, 1.89× as long as pronotum. Scutellum large, broad, linguiform, shiny, flush with elytra, flat. Elytral base transverse, edge oblique, humeral angles rounded, parallel-sided in basal 4/5, then sharply angulate to apex. Disc flat, shiny, striae and interstriae densely setose, setae long, semi-recumbent, hair-like, striae and interstriae strongly confused, indistinguishable; striae and interstriae not impressed, minutely punctate, punctures strongly confused, separated by 2–5 diameters of a puncture. Declivity occupying 1/3 of elytra, obliquely truncate, face convex, strongly shiny; five striae present, striae distinctly and uniformly impressed, striae 2 equidistant between 1 and 3, strial punctures large, distinct, subcontiguous to separated by two diameters of a puncture, subshiny, much larger than on disc; interstriae inflated on apical 1/2 of declivity, interstriae setose, setae dense, long, semi-erect hair-like; interstriae impunctate, coarsely tuberculate, tubercles sparse, small, strongly confused, variably sized. Posterolateral margin forming a circumdeclivital costa extending laterally to interstriae 7; costa granulate, setose, setae long, erect, fine, hair-like. ***Legs***: procoxae contiguous; prosternal coxal piece tall and pointed. Protibiae semi-circular with evenly rounded outer margin, broadest at apical 1/3; posterior face smooth; apical 1/3 of outer margin with ten moderate socketed denticles, their length approximately as long as basal width. Meso- and metatibiae broad, flattened; outer margin evenly rounded with 15 moderate socketed denticles.

##### Etymology.

L. *amputatus* = cut away, lopped off. In reference to the chopped appearance of the elytra. An adjective.

##### Distribution.

Vietnam.

##### Host plants.

Unknown.

##### Remarks.

The holotype is card mounted and ventral characters could not be examined.

#### Cyclorhipidion armiger

Taxon classificationAnimaliaColeopteraCurculionidae

(Schedl, 1953) comb. nov.

Fig. 40E, F, K

Xyleborus armiger Schedl, 1953c: 28.

##### Type material.

***Lectotype*** (NHMW).

##### New records.

China: Jiangxi, Longnan County, Jiulianshan, 24.541347; 114.460357, 613 m, 03.vii.2018, Lv-Jia & SC Lai, ex Anacardiaceae (LYLC, 1). S. Yunnan, Xishuangbanna, 23 km NW Jinghong, vic. Na Ban village (NNNR), 22°10'N, 100°39'E, 700–1000 m, Div. Fallen, v.–vii.2009, L. Meng (NKME, 1; RABC, 1); as previous except: 28 km NW Jinghong, vic. An Ma Xi Zhan (NNNR), 22°12'N, 100°38'E, 700 m, forest, EKL, 28.vi.2008, A. Weigel (MSUC, 1). Taiwan: Ilan Co., Yunshan, Fushan Res. Center-TFRI, 2.iii.2015, LJ Wang, ex log (RABC, 1). Thailand: Chiangmai, Fang, 12–19.iv.1958, T.C. Maa (BPBM, 1). Vietnam: Ha Tay, Ba Vi N.P. (lake lodge), 196 m, 3–4.vii.2008, J.B. Heppner (FSCA, 1). Thua Thien-Hue, Bach Ma N.P., 16.19831, 107.85639, 1386 m, 17–18.ii.2017, VN70, A.I. Cognato, T.A. Hoang, ex 3 cm branch (MSUC, 1). Vinh Phuc, Tam Dao, 985 m, 1–7.v.2012, J.B. Heppner (FSCA, 1).

##### Diagnosis.

2.6–3.3 mm long (mean = 2.89 mm; n = 9); 2.5–2.71× as long as wide.

This species is distinguished by the elytral disc with a median shallow saddle-like impression; declivital interstriae 2 granulate; declivity very steep, posterolateral margin feebly costate; elytral apex bearing four denticles, one on each interstriae 1 and 2; declivital face bearing four equally sized and spaced tubercles along interstriae 1; and striae slightly impressed.

##### Similar species.

*Cyclorhipidion miyazakiense*, *C. obesulum*, *C. xyloteroides*.

##### Distribution.

China (Fujian, Jiangxi*, Sichuan, Yunnan), Thailand*, Taiwan*, Vietnam*.

##### Host plants.

Recorded only from an unknown genus of Anacardiaceae.

##### Remarks.

The holotype of was examined and is here transferred to *Cyclorhipidion* because of the following characters: most of body covered with dense pubescence and discal strial and interstrial punctures strongly confused, pronotum and elytra rounded, with no conspicuous edges or carinas, semi-circular with evenly rounded outer edge, antennal club type 3, visible scutellum and lack of mycangial tufts.

#### Cyclorhipidion bodoanum

Taxon classificationAnimaliaColeopteraCurculionidae

(Reitter, 1913)

Fig. 40G, H, L

Xyleborus bodoanus Reitter, 1913: 82.
Cyclorhipidion bodoanum (Reitter): Bussler and Immler 2007: 5.
Xyleborus punctulatus Kurentzov, 1948: 52. Synonymy: Knížek 2011: 245.
Xyleborus californicus Wood, 1975b: 399. Synonymy: Knížek 2011: 245.
Xyleborus misatoensis Nobuchi, 1981a: 146. syn. nov.

##### Type material.

***Holotype****Xyleborus misatoensis* (NIAES).

##### New records.

China: Fujian, Chong’an, Guidun, 1200 m, 7.v.1978, host: *Cyclobalanopsis glauca* [= *Quercus glauca*] (NMNH, 1). Guizhou, Guiyang, Huaxi, 11.vi.2016, Y. Li, ex ethanol trap (MSUC, 1). Hong Kong, Tai Po Kau, vi.2017, J. Skelton (MSUC, 1). Jiangxi, Xunwu, Xingshan, 10.x.2018, Y. Li, ex Fagaceae log (MSUC, 1). S Yunnan, Xishuangbanna, 23 km NW Jinghong, vic. Na Ban village (NNNR), 22°10'N, 100°39'E, 700–1000 m, v–vii.2009, L. Meng (NKME, 4); as previous except: 25 km NW Jinghong, vic. Zhong Zhi Chang (NNNR), 22°11.08'N, 100°39.05'E, 780 m, rubber plantation, 6.iv.2009, L. Meng (NKME, 1); as previous except: 28 km NW Jinghong, vic. An Ma Xi Zhan (NNNR), 22°12'N, 100°38'E, 700 m, forest, 5.iv.2009, L. Meng (RABC, 3). Taiwan: Yilan Co., Fushan, v.2009, [no collector], ex sticky trap (RABC, 1). Vietnam: Bac Giang, Tay Yen Tu Nat. Res. 6 km SW Than Son, 21°10.83'N, 106°43.43'E, 200 m, 19–20.v.2015, A. Weigel (NKME, 1; RABC, 1).

##### Diagnosis.

1.7–2.2 mm long (mean = 2.03 mm; n = 4); 2.76–3.14× as long as wide. This species is distinguished by the short, steep declivity that is approximately 25% of total elytral length, armed with large tubercles on interstriae 1 and 3, interstriae 2 always unarmed; posterolateral margins rounded; and declivital interstriae 1 and 2 setae uniseriate (Table 1).

##### Similar species.

This species is a part of a challenging species group consisting of *C. distinguendum*, *C. inarmatum*, *C. pelliculosum*, *C. tenuigraphum* and *C. xeniolum* (Table 1).

##### Distribution.

China (Fujian*, Heilongjiang, Hong Kong*, Guizhou*, Jiangxi*, Yunnan*), Japan, Laos, South & North Korea, Russia (Far East), Taiwan*, Thailand, Vietnam*. Introduced to Europe, USA (Wood 1975; Vandenberg et al. 2000; Kirkendall and Faccoli 2010; Gomez et al. 2018a).

##### Host plants.

Like a number of other species of *Cyclorhipidion*, the species has a clear preference for trees in the family Fagaceae, and most records are from *Quercus*, with rare attacks on *Castanea* (Nobuchi 1981a, Bussler and Immler 2007). Also recorded from *Pinus* (Pinaceae) and *Populus* (Salicaceae) (Lightle et al. 2007).

##### Remarks.

The holotype of *Xyleborus misatoensis* was compared to specimens of *C. bodoanum* from the United States and Asia. The specimens were found to be conspecific and *X. misatoensis* is here placed in synonymy.

McPherson et al. (2008) note that the species attacks *Quercus* previously attacked by pathogenic fungi, resulting in the spread of decay fungi, and increased tree mortality.

**Table 1.** Diagnostic characters for *Cyclorhipidion* species near *C. pelliculosum*.

Species	Declivital interstriae 1 setae	Declivital interstriae 2 setae	Lateral profile of declivity	Declivital interstriae 2 granulate	Total length (mm)
* xeniolum *	uniseriate	uniseriate	obliquely truncate	unarmed	1.65–1.8
* bodoanum *	uniseriate	uniseriate	steeply rounded	unarmed	1.7–2.2
* inarmatum *	2 rows, confused	uniseriate	steeply rounded	unarmed	2.8–3.0
* tenuigraphum *	2 rows, confused	2 rows, confused	steeply rounded	often on apical third	2.7–3.0
* pelliculosum *	3 rows, confused	uniseriate	steeply rounded	unarmed	3.2–3.5
* distinguendum *	3 rows, confused	2 rows, confused	steeply rounded	unarmed	2.5–3.1

#### Cyclorhipidion circumcisum

Taxon classificationAnimaliaColeopteraCurculionidae

(Sampson, 1921)

Fig. 41A, B, I

Xyleborus circumcisus Sampson, 1921: 30.
Cyclorhipidion circumcisum (Sampson): Wood and Bright 1992: 698.
Xyleborus obtusus Eggers, 1923: 172. Synonymy: Browne 1959: 97.
Xyleborus subobtusus Schedl, 1942a: 192. Synonymy: Beaver 2011: 283.

##### Type material.

***Holotype****Xyleborus circumcisus* (NHMUK).

##### Diagnosis.

3.3–3.5 mm long (mean = 3.43 mm; n = 5); 2.54–2.83× as long as wide. This species is distinguished by the large size; declivity obliquely truncate, strongly shagreened and dull; pronotum longer than wide and basic from dorsal view (type 2); declivital interstriae densely covered in multiseriate rows of tubercles.

##### Similar species.

*Cyclorhipidion amasoides*, *C. amputatum*, *C. muticum*, *C. truncaudinum*, *C. umbratum*, all of which are large and have an obliquely truncate or truncate declivity.

##### Distribution.

Indonesia (Java, Sumatra), East & West Malaysia, Philippines, Thailand.

##### Host plants.

The species has a strong preference for Fagaceae (*Castanopsis*, *Lithocarpus*, *Quercus*) (Browne 1961b). There are single records from *Canarium* (Burseraceae), and an unidentified tree of the same family (Browne 1986; Ohno 1990).

**Figure 41.**
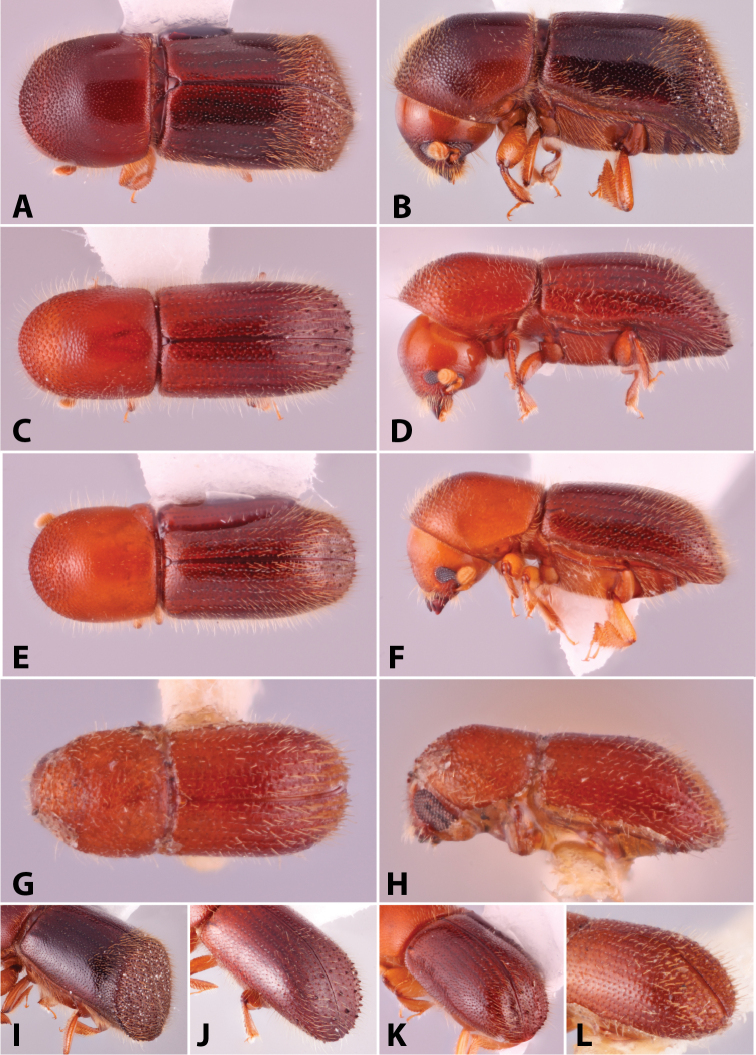
Dorsal, lateral and declivital view of *Cyclorhipidion circumcisum*, 3.3–3.5 mm (**A, B, I**), *C. denticauda* holotype, 2.95–3.1 mm (**C, D, J**), *C. distinguendum*, 2.5–3.1 mm (**E, F, K**), and *C. fouqueti* lectotype, 1.8 mm (**G, H, L**).

#### Cyclorhipidion denticauda sp. nov.

Taxon classificationAnimaliaColeopteraCurculionidae

http://zoobank.org/180741D4-6A5F-416B-A1D3-CEE2447142A3

Fig. 41C, D, J

##### Type material.

***Holotype***, female, Vietnam: Cao Bang, 22°34.5'N, 105°52.4'E, ~1080 m, 14.iv.2014, VN20, Cognato, Smith, Pham, ex branches (MSUC). ***Paratype***, female, China: Jiangxi, Jinggang Shan Mts., Xiangzhou vill. env., 26°35.5'N, 114°16.0'E, 374 m, (rice fields, forested stream valley), 26.iv.2011, M. Fikáček & J. Hájek (RABC).

##### Diagnosis.

2.95–3.1 mm long (mean = 3.02 mm; n = 2); 2.81–3.1× as long as wide. This species is distinguished by the declivital slope gentle, gradual; separation between the smooth, shiny elytral disc and shagreened declivity gradual, not sharply distinct; declivital striae weakly impressed, punctures large, distinct; declivital interstriae tuberculate with three large equally spaced tubercles along interstriae 1 and 3, those of interstriae 3 smaller; interstriae 2 granulate near base, interstriae 4 granulate along its length; and elytral apex and posterolateral margin armed with alternating spines and denticles, a single spine on each interstriae and a smaller denticle on each striae from suture to interstriae 7.

##### Similar species.

*Cyclorhipidion ohnoi*, *C. petrosum*, *C. pilipenne*.

##### Description

**(female).** 2.95–3.1 mm long (mean = 3.02 mm; n = 2); 2.81–3.1 × as long as wide. Body red. Legs and antennae light brown. ***Head***: epistoma entire, transverse, with a row of hair-like setae. Frons weakly convex to upper level of eyes; surface subshiny, impunctate, alutaceous, rugose. Eyes shallowly emarginate just above antennal insertion, upper part smaller than lower part. Submentum narrow, triangular, shallowly impressed. Antennal scape regularly thick, as long as club. Pedicel as wide as scape, shorter than funicle. Funicle 4-segmented, segment 1 as long as pedicel. Club approximately circular and flat, type 3; segment 1 corneous, transverse on anterior face, occupying approximately basal 2/5; segment 2 narrow, corneous; segments 1 and 2 present on posterior face. ***Pronotum***: 1.24× as long as wide. In dorsal view long and rounded frontally, type 7, sides parallel in basal 3/4, rounded anteriorly; anterior margin without serrations. In lateral view elongate with disc much longer than anterior slope, type 7, disc flat, summit at apical 2/5. Anterior slope shagreened, with densely spaced, fine, narrow asperities, becoming lower and more strongly transverse towards summit, bearing long, fine, semi-recumbent, hair-like setae. Disc subshiny, alutaceous, densely, finely punctate, finely setose, setae short, erect, hair-like. Lateral margins obliquely costate. Base transverse, posterior angles broadly rounded. ***Elytra***: 1.92× as long as wide, 1.54× as long as pronotum. Scutellum large, broad, linguiform, flush with elytra, flat, shiny. Elytral base transverse, edge oblique, humeral angles rounded, parallel-sided in basal 4/5, then broadly rounded to apex. Disc flat, strongly shiny, striae setose, setae short, semi-recumbent, hair-like, interstriae glabrous, striae not impressed, punctures uniseriate, spaced by two or three diameters of a puncture, interstriae minutely punctate, punctures 1/2 size of strial punctures, strongly confused, separated by five diameters of a puncture. Declivity occupying 2/5 of elytra, declivital slope gradual, rounded, strongly shagreened, separation between the smooth, shiny disc and shagreened declivity gradual, not sharply distinct; six striae present, striae 2 equidistant between 1 and 3, striae weakly impressed, punctures large, shallow, distinct, subcontiguous to spaced one diameter of a puncture, shagreened, much larger than on disc, glabrous; interstriae feebly convex, interstriae setose, setae dense, very long, erect hair-like; interstriae 1 laterally broadened from declivital summit to apical 1/3 then narrowed to apex, interstriae impunctate, tuberculate with three large equally spaced tubercles along interstriae 1 and three smaller tubercles on interstriae 3, interstriae 4 granulate, interstriae 2 denticulate near summit. Apex and posterolateral margin armed with alternating spines and denticles, a single spine on each interstria and a smaller denticle on each stria from suture to interstriae 7. ***Legs***: procoxae contiguous; prosternal coxal piece tall, conical. Protibiae semi-circular with evenly rounded outer edge, broadest at apical 1/3; posterior face smooth; apical 1/3 of outer margin with seven moderate socketed denticles, their length approximately as long as basal width. Meso- and metatibiae broad, flattened; outer margin evenly rounded with ten and 12 moderate socketed denticles, respectively.

##### Etymology.

L. *dentis* = tooth; *cauda* = tail. In reference to the declivity which is adorned with spines and denticles. Noun in apposition.

##### Distribution.

China (Jiangxi), Vietnam.

##### Host plants.

Unknown.

#### Cyclorhipidion distinguendum

Taxon classificationAnimaliaColeopteraCurculionidae

(Eggers, 1930)

Fig. 41E, F, K

Xyleborus distinguendus Eggers, 1930: 205.
Cyclorhipidion distinguendum (Eggers): Maiti and Saha 2004: 105.
Xyleborus fukiensis Eggers, 1941b: 225. syn. nov.
Xyleborus ganshoensis Murayama, 1952: 16. syn. nov.

##### Type material.

***Neotype****Xyleborus distinguendus* (FRI). ***Holotype****Xyleborus fukiensis* (ZMFK). ***Holotype****Xyleborus ganshoensis* (NMNH).

##### New records.

China: Beijing, 15.iv.1980, Peiyu Yu (NMNH, 1). Fujian, Chong’an, Guidun, 1500 m, 7.v.1978, ex *Cyclobalanopsis glauca* [= *Quercus glauca*] (NMNH, 2). Hong Kong, Tai Po Kau, vi.2017, J. Skelton, ex *Castanopsis* (MSUC, 2). Jiangxi, Long Nan, 12.vii.2016, Lv-Jia, Lai, S-C., ex *Cyclobalanopsis glauca* (RABC, 1). Yunnan, Xishuangbanna, Sanchahe Nat. Res., 22°09.784'N, 100°52.256'E, 2186 m, 29.v.2008, A.I. Cognato (MSUC, 4); S Yunnan, Xishuangbanna, 23 km NW Jinghong, vic. Na Ban village (NNNR), 22°10'N, 100°39'E, 700–1000 m, v–vii.2009, L. Meng (NKME, 1). Japan: Tsukuba, 27.viii.1980, S.L. Wood, ex *Abies firma* (NMNH, 1). Taiwan: Fushan, iii.2015, J. Hulcr, ex *Pasania* [= *Lithocarpus*] (UFFE, 1). Taichung, Heping Dist., 29.iv.2014, C.-S. Lin (MSUC, 1). Tai Pei Co., Noi Dong logging road, 850 m, 19.ii.2004, Chun Lin Li, ex flight intercept trap (MFNB, 1). Vietnam: Cao Bang, 22°34.5'N, 105°52.4'E, ~ 1080 m, VN 20, Cognato, Smith, Pham, ex branches (MSUC, 4). Lao Cai, Hoang Lien N.P., 22.35, 103.77, 1500 m, 17.v.2019, VN161, S.M. Smith, A.I. Cognato, ex branch; 5 cm (MSUC, 1). Thua Thien-Hue, Bach Ma N.P., 16.19718, 107.86002, 1409 m, 14.ii.2017, VN50, A.I. Cognato, T.A. Hoang, ex Fagaceae, 4 cm branch and twigs (MSUC, 2).

##### Diagnosis.

2.5–3.11 mm long (mean = 2.78 mm; n = 15); 2.6–3.0× as long as wide. This species is distinguished by the short, steep declivity that is approximately 25% of total elytral length, armed with large tubercles on interstriae 1 and 3, interstriae 2 always unarmed; posterolateral margins rounded; and declivital interstriae 1 setae in three confused rows and interstriae 2 setae in two confused rows (Table 1). This species is highly morphologically variable. See remarks below.

##### Similar species.

This species is a part of a challenging species group consisting of *C. bodoanum*, *C. inarmatum*, *C. pelliculosum*, *C. tenuigraphum* and *C. xeniolum* (Table 1).

##### Distribution.

China (Beijing*, Fujian, Hong Kong*, Jiangxi*, Yunnan*), India (Uttarakhand), Japan, Nepal, South Korea, Taiwan, Thailand, Vietnam*. Recently established in the United States (Hoebeke et al. 2018) and France (Dodelin 2018) as *C. fukiense*.

##### Host plants.

Recorded from *Castanea*, *Lithocarpus*, and *Quercus* (Fagaceae), and probably with a close association with Fagaceae (Beaver et al. 2014).

##### Remarks.

Images of the *X. distinguendus* holotype were examined and compared to the *X. fukiensis* and *X. ganshoensis* holotypes and were found to be conspecific. Both *X. fukiensis* and *X. ganshoensis* are here placed in synonymy.

The declivity of *C. distinguendum* is highly morphologically variable in regard to numerous key features that are routinely used to diagnose other xyleborine species and each elytron of an individual often has a different arrangement of tubercles. When trying to identify *C. distinguendum* note that the characters listed above in the diagnosis are the only way to reliably identify the species. Characters such as surface luster, the number, size, and position of interstrial tubercles, declivital puncture size, interstrial convexity and strial impression are all highly variable. Variation in all these characters can be found in individuals from one locality and even from a single host (Smith, pers. obs.). Individuals exhibiting such variation were monophyletic in a molecular phylogeny using COI and CAD (Cognato et al. 2020b). Examples of variation in the declivity include: A. The declivital surface shiny, opalescent or shagreened; B. Strial and interstrial puncture size range from equal widths to those of the striae 2× as large as those of interstriae; C. Interstriae 2 flat to depressed (typically depressed); D. Interstriae 1 and 3 flat to convex (typically convex); E. Interstriae 1 tuberculate with 1–3 tubercles and granules varying in both number and position; F. Interstriae 3 with 2–5 tubercles and often granules, all of which vary in position. G. Surface slightly to deeply impressed between striae 1 and 2.

#### Cyclorhipidion fouqueti

Taxon classificationAnimaliaColeopteraCurculionidae

(Schedl, 1937)

Fig. 41G, H, L

Xyleborus fouqueti Schedl, 1937b: 15.
Cyclorhipidion fouqueti (Schedl): Wood and Bright 1992: 699.

##### Type material.

***Lectotype*** (NHMW).

##### Diagnosis.

1.8 mm long (n = 1); 2.57× as long as wide. This species is distinguished by its minute size; eyes that are nearly as large as the head and very weakly emarginated; and declivity bearing scale-like setae.

##### Similar species.

None.

##### Distribution.

Vietnam.

##### Host plants.

Unknown.

##### Remarks.

This tiny species is only known from the lectotype specimen which is point mounted with an excessive amount of glue. This mounting prevented the examination of most antennal and ventral characters, including the legs. It is likely that this species belongs in a different genus, potentially *Tricosa*, but these characters will need to be examined before the species can be transferred.

#### Cyclorhipidion inarmatum

Taxon classificationAnimaliaColeopteraCurculionidae

(Eggers, 1923)

Fig. 42A, B, I

Xyleborus inarmatus Eggers, 1923: 209.
Cyclorhipidion inarmatum (Eggers): Beaver et al. 2014: 39.
Xyleborus vagans Schedl, 1977: 504. syn. nov.

##### Type material.

***Lectotype****Xyleborus inarmatus* (NMNH). ***Holotype****Xyleborus vagans* (NHMW).

##### New records.

Bhutan: Thimpu, km 125 Phuntsholing, 2300 m, 24.v.1972, Nat.-Hist. Mus Basel, Bhutan Expedition (NHMB, 1) [misdetermined by Schedl as *Xyleborus corporaali* Eggers]. China: Yunnan, Lijiang, v.1975, Zhizhong Zhang, ex *Pistacia weinmannifolia* (NMNH, 1). Laos: NE, Houa Phan, Ban Saluei – Phou Pane Mt., 20°12–13.5'N, 103°59.5–104°01'E, 1340–1780 m, 15.iv–15.v.2008, Lao collectors (MNHP, 1).

##### Diagnosis.

2.8–3.0 mm long (mean = 2.9 mm; n = 3); 2.8–3.0× as long as wide. This species is distinguished by the short, steep declivity that is approximately 25% of total elytral length, armed with large tubercles on interstriae 1 and 3, interstriae 2 always unarmed; posterolateral margins rounded; and declivital interstriae 1 setae in two confused rows, interstriae 2 setae uniseriate (Table 1).

##### Similar species.

This species is a part of a challenging species group consisting of *C. bodoanum*, *C. distinguendum*, *C. pelliculosum*, *C. tenuigraphum* and *C. xeniolum* (Table 1).

##### Distribution.

Bhutan*, China* (Yunnan), India (Himachal Pradesh, West Bengal), Indonesia (Sumatra), Laos*, Myanmar, Thailand, Vietnam.

##### Host plants.

Recorded from *Castanopsis* and *Quercus* (Fagaceae), and probably with a close association with Fagaceae (Beaver et al. 2014).

##### Remarks.

The holotype of *Xyleborus vagans* was compared with the lectotype of *X. inarmatum* and was found to be conspecific. *Xyleborus vagans* is slightly smaller than *X. inarmatum* but the specimens are identical in every other way. The specimens were found to be conspecific and *X. vagans* is here placed in synonymy.

**Figure 42.**
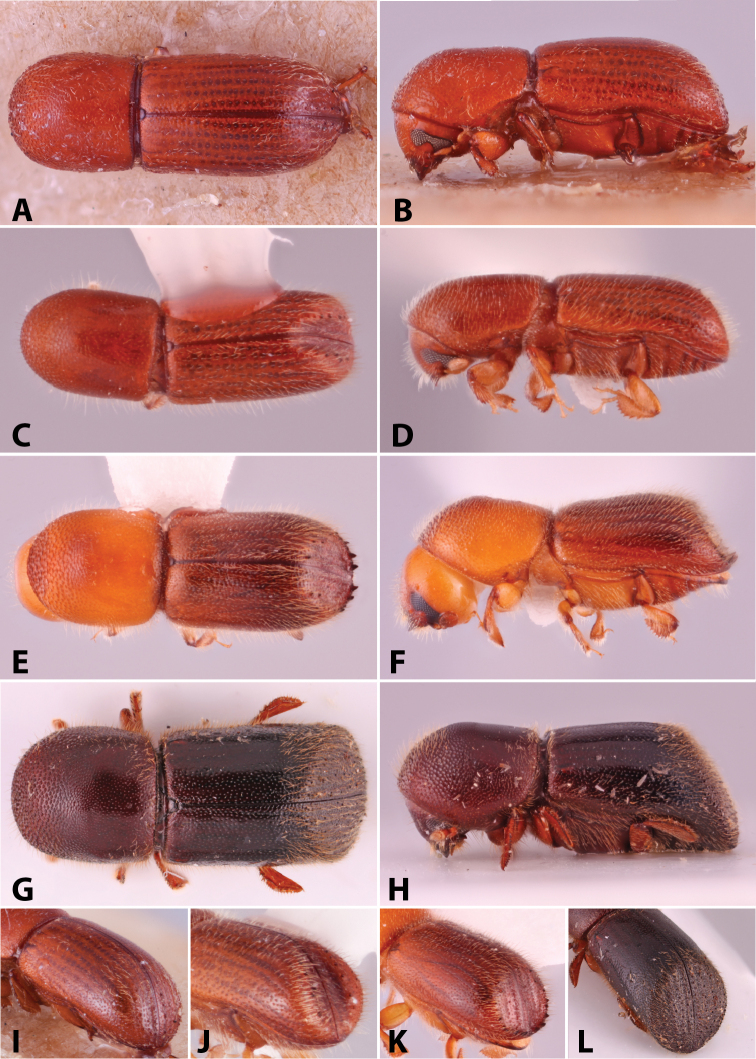
Dorsal, lateral and declivital view of *Cyclorhipidion inarmatum* lectotype, 2.8–3.0 mm (**A, B, I**), *C. japonicum*, 2.1–2.3 mm (**C, D, J**), *C. miyazakiense*, 2.6–3.0 mm (**E, F, K**), *C. muticum* holotype, 4.0 mm (**G, H, L**).

#### Cyclorhipidion japonicum

Taxon classificationAnimaliaColeopteraCurculionidae

(Nobuchi, 1981)

Fig. 42C, D, J

Xyleborus japonicus Nobuchi, 1981a: 153.
Cyclorhipidion japonicum (Nobuchi): Smith et al. 2018b: 394.

##### Type material.

***Holotype*** (NIAES).

##### New records.

China: [unspecificed province], northeastern China, DB07, A56, Wang (RJRC, 1). S. Yunnan, Xishuangbanna, 28 km NW Jinghong, vic. An Ma Xi Zhan (NNNR), 22°12'N, 100°38'E, 700 m, forest, 25.iii.2009, L. Meng (RABC, 1); as previous except: 23 km NW Jinghong, vic. Na Ban (NNNR), 22°09.49'N, 100°39.92'E, 730 m, rubber plantation, 15.vi.2008, A. Weigel (RABC, 1). Thailand: Chiang Mai, Doi Pui, 1400 m, 18–22.x.2004, W. Puranasakul, ex EtOH trap (RABC, 2); as previous except: 6–27.vi.2005 (RABC, 1). [Chaiyaphum], Phu Khieo N.P., branch, vii.2005, Hulcr et al. (RABC, 1).

##### Diagnosis.

2.1–2.3 mm long (mean = 2.2 mm; n = 4); 2.81–3.5× as long as wide. This species is distinguished by the small size; declivity obliquely truncate, moderately to strongly sulcate; pronotum elongate from dorsal view (type 9); declivity laterally sulcate to interstriae 3, interstriae 3 bearing five tubercles along its length.

##### Similar species.

*Cyclorhipidion neocavipenne*, *C. xeniolum*.

##### Distribution.

China* (Yunnan), Japan, Russia (Far East), South Korea, Thailand*.

##### Host plants.

Recorded only from *Castanopsis* and *Quercus* (Fagaceae) (Nobuchi 1981a).

#### Cyclorhipidion miyazakiense

Taxon classificationAnimaliaColeopteraCurculionidae

(Murayama, 1936)

Fig. 42E, F, K

Xyleborus miyazakiensis Murayama, 1936: 144.
Cyclorhipidion miyazakiense (Murayama): Smith et al. 2018b: 395.
Xyleborus armipennis Schedl, 1953c: 27. Synonymy: Smith et al. 2018b: 395.
Xyleborus wakayamensis Nobuchi, 1981a: 144. Synonymy: Smith et al. 2018b: 395.

##### Type material.

***Lectotype****Xyleborus armipennis* (NHMW). ***Holotype****Xyleborus wakayamensis* (NIAES).

##### New records.

China: Fukien [Fujian], Shaowu, Tachuland, 4.v.1943, T.C. Maa (BPBM, 1). N. Guangxi reg., Miaoershan, S slope, 1300–200 m, 25–28.vi.1997, Bolm (NHMB, 2; RABC, 1). Japan: Okinawa, Yona, J. Hulcr, ex *Castanopsis* (UFFE, 1). Vietnam: Lao Cai, Hoang Lien N.P., 22.35, 103.77, 1500 m, 17.v.2019, VN156, S.M. Smith, A.I. Cognato, ex 4 cm branch (MSUC, 1).

##### Diagnosis.

2.6–3.0 mm long (mean = 2.87 mm; n = 4); 2.5–2.8× as long as wide. This species is distinguished by the elytral disc with a shallow median saddle-like impression; declivity very steep; declivital posterolateral margin carinate to interstriae 5; elytral apex bearing two single triangular spines at interstriae 1 and 3 that are at least the width of an interstria (additional smaller denticles may be present along posterolateral margin); and declivital face unarmed by tubercles.

##### Similar species.

*Cyclorhipidion armiger*, *C. obesulum*, *C. xyloteroides*.

##### Distribution.

China (Fujian, Guangxi*, Sichuan), Japan, Thailand, Vietnam*.

##### Host plants.

Recorded only from *Castanopsis* and *Quercus* (Fagaceae) (Murayama 1936; Beaver et al. 2014).

#### Cyclorhipidion muticum sp. nov.

Taxon classificationAnimaliaColeopteraCurculionidae

http://zoobank.org/FAA958B6-3DF5-4D13-97EB-6B812B8FD967

Fig. 42G, H, L

##### Type material.

***Holotype***, female, India: Arunachal Pradesh, Etalin vicinity, 28°36'56"N, 95°53'21"E, 700 m, 12–25.v.2012, L. Dembický (ZFMK). ***Paratypes***, female, as holotype (ZFMK, 2).

##### Diagnosis.

4.0 mm long (n = 3); 2.67× as long as wide. This species is distinguished by the large size; declivity obliquely truncate, strongly shagreened and dull; pronotum longer than wide subquadrate from dorsal view (type 3); and declivital interstrial punctures replaced by a single row of tubercles.

##### Similar species.

*Cyclorhipidion amasoides*, *C. amputatum*, *C. circumcisum*, *C. truncaudinum*, *C. umbratum*, all of which are large and have an obliquely truncate or truncate declivity.

##### Description

**(female).** 4.0 mm long (n = 3); 2.67× as long as wide. Body dark red-brown. Legs and antennae dark brown. ***Head***: epistoma entire, transverse, with a row of hair-like setae. Frons weakly convex to upper level of eyes; surface shagreened, impunctate, alutaceous, moderately rugose. Eyes deeply emarginate just above antennal insertion, upper part smaller than lower part. Antennal scape regularly thick, as long as club. Pedicel as wide as scape, shorter than funicle. Funicle 4-segmented, segment 1 shorter than pedicel. Club approximately circular and flat, type 3; segment 1 corneous, transverse on anterior face, occupying approximately basal 1/3; segment 2 narrow, corneous; segments 1–3 present on posterior face. ***Pronotum***: 1.05× as long as wide. In dorsal view subquadrate, sides convex, type 3, narrowly rounded anteriorly; anterior margin without serrations. In lateral view elongate with disc much longer than anterior slope, type 7, disc flat, summit at apical 2/5. Anterior slope shagreened, with densely spaced, fine, narrow asperities, becoming lower and more strongly transverse towards summit, bearing long, fine, semi-recumbent, hair-like setae. Disc shagreened, alutaceous, densely, finely punctate, setose, setae dense, long, fine hair-like. Lateral margins obliquely costate. Base transverse, posterior angles narrowly rounded. ***Elytra***: 1.68× as long as wide, 1.6× as long as pronotum. Scutellum large, broad, linguiform, shiny, flush with elytra, flat. Elytral base transverse, edge oblique, humeral angles rounded, parallel-sided in basal 4/5, then sharply angulate to apex. Disc ascending posteriorly, shiny, basal 1/5 shagreened, striae and interstriae densely setose, setae long, semi-recumbent, hair-like, striae and interstriae strongly confused, indistinguishable; striae and interstriae not impressed, punctures strongly confused, separated by 2–5 diameters of a puncture. Declivity occupying 1/4 of elytra, obliquely truncate, face weakly convex, strongly shagreened, densely setose; six striae present, striae weakly impressed, striae 2 equidistant between 1 and 3, strial punctures large, shallow, subcontiguous, shagreened, much larger than on disc; interstriae weakly convex, interstriae very densely setose, setae long, semi-erect hair-like; interstriae impunctate, uniseriate tuberculate, tubercles numerous, moderately large and irregularly spaced. Posterolateral margin costate, granulate, extending laterally to interstriae 7; setose, setae long, fine, erect, hair-like. ***Legs***: procoxae contiguous. Protibiae semi-circular with evenly rounded outer edge, broadest at apical 1/3; posterior face smooth; apical 1/3 of outer margin with 9–11 moderate socketed denticles, their length approximately as long as basal width. Meso- and metatibiae broad, flattened; outer margin evenly rounded with 16 moderate socketed denticles.

##### Etymology.

L. *muticus* = maimed, broken. In reference to the truncate declivity. An adjective.

##### Distribution.

India (Arunachal Pradesh).

##### Host plants.

Unknown.

##### Remarks.

The type series is card mounted and ventral characters could not be examined.

#### Cyclorhipidion neocavipenne

Taxon classificationAnimaliaColeopteraCurculionidae

(Schedl, 1977)

Fig. 43A, B, I

Xyleborus neocavipennis Schedl, 1977: 503.
Cyclorhipidion neocavipenne (Schedl): Wood and Bright 1992: 700.

##### Type material.

***Holotype*** (NHMW).

##### New record.

Thailand: Chiang Mai, Doi Pui, 1400 m, 2004, W. Puranasakul (RABC, 2).

##### Diagnosis.

2.5 mm long (n = 1); 3.13× as long as wide. This species is distinguished by the small size; declivity obliquely truncate and moderately sulcate; pronotum elongate from dorsal view (type 9); declivity sulcate to interstriae 4; and declivity only armed by two granules near base of interstriae 3.

##### Similar species.

*Cyclorhipidion japonicum*, *C. xeniolum*.

##### Distribution.

Thailand*, Vietnam.

##### Host plants.

Unknown.

**Figure 43.**
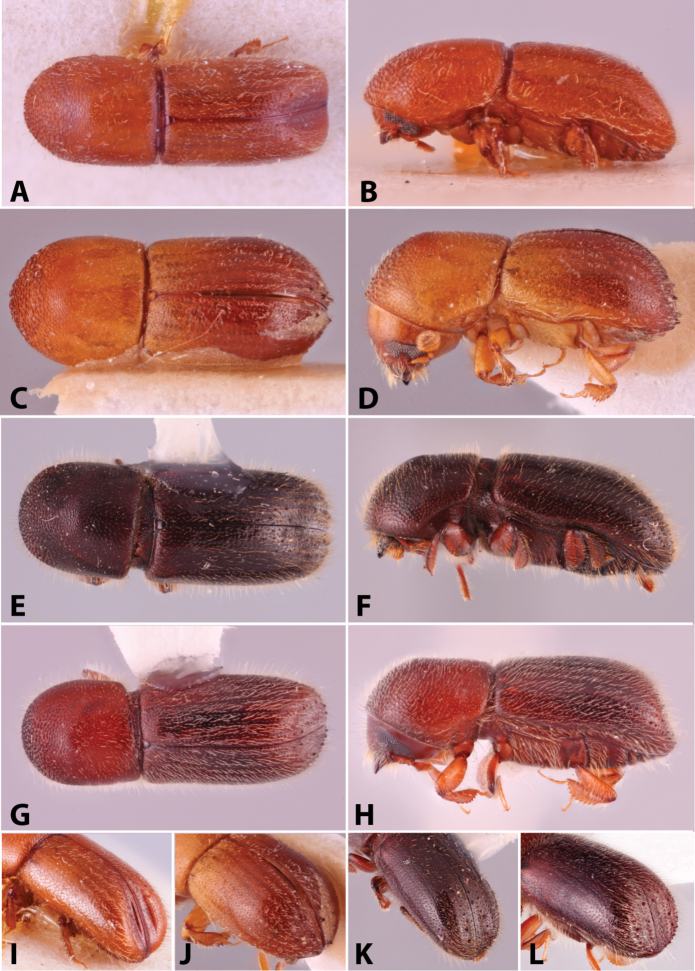
Dorsal, lateral and declivital view of *Cyclorhipidion neocavipenne* holotype, 2.5 mm (**A, B, I**), *C. obesulum* holotype, 2.1 mm (**C, D, J**), *C. ohnoi* holotype, 4.1–4.2 mm (**E, F, K**), and *C. pelliculosum*, 3.2–3.5 mm (**G, H, L**).

#### Cyclorhipidion obesulum sp. nov.

Taxon classificationAnimaliaColeopteraCurculionidae

http://zoobank.org/56977393-2740-45A5-B9F2-1130873A85EA

Fig. 43C, D, J

##### Type material.

***Holotype***, female, China: S. Yunnan, Xishuangbanna, 23 km NW Jinghong, vic. Na Ban village (NNNR), 22°10'N, 100°39'E, 700–1000 m, v–vii.2009, L. Meng (NKME).

##### Diagnosis.

2.1 mm long (n = 1); 2.45× as long as wide. This species is distinguished by the elytral disc convex; declivity rounded; and elytral apex bearing a single, strong triangular spine at the end of interstriae 2.

##### Similar species.

*Cyclorhipidion armiger*, *C. miyazakiense*, *C. xyloteroides*.

##### Description

**(female).** 2.1 mm long (n = 1); 2.45× as long as wide. Appearing bicolored: body, antennae, legs, elytral base light brown, remainder of declivity darker brown. ***Head***: epistoma entire, transverse, with a row of hair-like setae. Frons weakly convex to upper level of eyes; surface subshiny, impunctate, alutaceous, finely rugose. Eyes deeply emarginate just above antennal insertion, upper part smaller than lower part. Submentum narrow, triangular, deeply impressed. Antennal scape regularly thick, as long as club. Pedicel as wide as scape, shorter than funicle. Funicle 4-segmented, segment 1 shorter than pedicel. Club approximately circular and flat, type 3; segment 1 corneous, transverse on anterior face, occupying approximately basal 1/3; segment 2 narrow, corneous; segments 1 and 2 present on posterior face. ***Pronotum***: 1.17× as long as wide. In dorsal view long and rounded frontally, type 7, sides parallel in basal 1/2, rounded anteriorly; anterior margin without serrations. In lateral view elongate with disc much longer than anterior slope, type 7, disc flat, summit at apical 2/5. Anterior slope shagreened, with densely spaced, fine, narrow asperities, becoming lower and more strongly transverse towards summit, bearing long, fine, semi-recumbent, hair-like setae. Disc shiny, densely, finely punctate, finely setose, setae short, erect, hair-like. Lateral margins obliquely costate. Base transverse, posterior angles broadly rounded. ***Elytra***: 1.2× as long as wide, 1.03× as long as pronotum. Scutellum large, broad, linguiform, flush with elytra, flat, shiny. Elytral base transverse, edge oblique, humeral angles rounded, parallel-sided in basal 2/3, then broadly rounded to apex. Disc convex, shiny, striae and interstriae densely setose, setae very short, semi-recumbent, hair-like, striae and interstriae strongly confused, indistinguishable; striae and interstriae not impressed, minutely punctate, punctures strongly confused, separated by one diameter of a puncture. Declivity occupying 1/3 of elytra, declivital slope gradual, rounded, shagreened, separation between the smooth, shiny disc and shagreened declivity gradual, not sharply distinct; three striae present, striae 2 equidistant between 1 and 3, striae not impressed, punctures small, shallow, distinct, spaced by one diameter of a puncture, shagreened, larger than on disc; interstriae feebly convex, setose, setae dense, long, semi-erect hair-like, apically increasing in length and thickness, each interstriae with two rows; interstriae 1 laterally broadened from declivital summit to midpoint then narrowed to apex, minutely punctate, punctures strongly confused, interstriae 1 with two large tubercles in median area (variable placement on each elytron), interstriae 2 unarmed, interstriae 3 with two equally spaced tubercles on apical 1/2; apex bearing a single, strong triangular spine at apex of interstriae 2. Posterolateral margin round, granulate. ***Legs***: procoxae contiguous; prosternal coxal piece flat and inconspicuous. Protibiae semi-circular with evenly rounded outer edge, broadest at apical 1/3; posterior face smooth; apical 1/3 of outer margin with six moderate socketed denticles, their length approximately as long as basal width. Meso- and metatibiae broad, flattened; outer margin evenly rounded with eight moderate socketed denticles.

##### Etymology.

L. *obesus* = stout, plump; -*ulum* = diminutive suffix. An adjective.

##### Distribution.

China (Yunnan).

##### Host plants.

Unknown.

#### Cyclorhipidion ohnoi

Taxon classificationAnimaliaColeopteraCurculionidae

(Browne, 1980)

Fig. 43E, F, K

Xyleborus ohnoi Browne, 1980a: 375.
Cyclorhipidion ohnoi (Browne): Beaver and Liu 2010: 24.

##### Type material.

***Holotype*** (NHMUK).

##### New records.

Taiwan: Fushan, iii.2015, J. Hulcr, ex *Lithocarpus* (UFFE, 1).

##### Diagnosis.

4.1–4.2 mm long (mean = 4.15 mm; n = 2); 2.73–2.8× as long as wide. This species is distinguished by its large size; pronotal disc coarsely and densely punctured, strongly shagreened; elytra shiny; declivity impressed between suture and striae 1, interstriae 2 convex; and declivital interstriae 1 sparsely granulate, interstriae 2 and 3 each with a row of widely spaced large tubercles, those on interstriae 2 larger.

##### Similar species.

*Cyclorhipidion denticauda*, *C. petrosum*, *C. pilipenne*.

##### Distribution.

Taiwan.

##### Host plants.

Recorded only from *Quercus* (Beaver and Liu 2010) and *Lithocarpus* (Fagaceae).

#### Cyclorhipidion pelliculosum

Taxon classificationAnimaliaColeopteraCurculionidae

(Eichhoff, 1878)

Fig. 43G, H, L

Xyleborus pelliculosus Eichhoff, 1878a: 392.
Cyclorhipidion pelliculosum (Eichhoff): Hulcr and Cognato 2010a: 12.
Xyleborus seiryorensis Murayama, 1930: 25. Synonymy: Knížek 2011: 243.
Xyleborus quercus Kurentzov, 1948: 51. Synonymy: Knížek 2011: 243.
Xyleborus starki Nunberg, 1956: 209 (new name for X. quercus Kurentzov, 1948 nec Hopkins 1915). Synonymy: Knížek 2011: 243.

##### Type material.

***Syntypes****Xyleborus seiryorensis* (NMNH, 3).

##### New records.

Taiwan: Ilan Co., Fushan, 2000 m, 27.vi.1995, A. Warneke, ex light trap (RABC, 1); as previous except: 26.vii.1995 (RABC, 1).

##### Diagnosis.

3.2–3.5 mm long (mean = 3.3 mm; n = 5); 2.67–3.0× as long as wide. This species is distinguished by the short, steep declivity that is approximately 25% of total elytral length, armed with large tubercles on interstriae 1 and 3, interstriae 2 always unarmed; posterolateral margins rounded; and declivital interstriae 1 setae in three confused rows, interstriae 2 setae uniseriate (Table 1).

##### Similar species.

This species is a part of a challenging species group consisting of *C. bodoanum*, *C. distinguendum*, *C. inarmatum*, *C. tenuigraphum*, and *C. xeniolum* (Table 1).

##### Distribution.

China (Shanxi, Sichuan), Japan, South & North Korea, Russia (Far East), Taiwan*. Imported to USA (Atkinson et al. 1990).

##### Host plants.

Most records are from *Castanopsis* and *Quercus* (Fagaceae), but the species has also been recorded from *Acer* (Aceraceae), *Juglans* (Juglandaceae) and from *Alnus* and *Betula* (Betulaceae) (Mandelshtam et al. 2018).

#### Cyclorhipidion perpilosellum

Taxon classificationAnimaliaColeopteraCurculionidae

(Schedl, 1935)

Fig. 44A, B, I

Xyleborus perpilosellus Schedl, 1935a: 402.
Cyclorhipidion perpilosellum (Schedl): Wood and Bright 1992: 701.
Xyleborus punctatopilosus Schedl, 1936b: 532. Synonymy: Bright and Skidmore 1997: 4, 151.

##### Type material.

***Lectotype****Xyleborus perpilosellus* (NHMW).

##### New records.

China: S Yunnan, Xishuangbanna, 23 km NW Jinghong, vic. Na Ban village (NNNR), 22°10'N, 100°39'E, 700–1000 m, v–vii.2009, L. Meng (RABC, 2); Xishuangbanna Sanchahe Nat. Res., 22°09.784'N, 100°52.256'E, 2186 m, 29–30.v.2008, A. Cognato, ex *Quercus* (MSUC, 2); as previous except: Simao, 1380 m, 22.vi.1978, Fanjie Zeng, ex Fagaceae (NMNH, 1). India: Arunachal Pradesh, Etalin vicinity, 28°36'56"N, 95°53'21"E, 700 m, 12–25.v.2012, L. Dembický (ZFMK, 1). Laos: C, Kham Mouan, Ban Khoun Ngeun, 18°07'N, 104°29'E, 24–29.iv.2001, P. Pacholátko (RABC, 1); 10 km N Luang-Prabang, Mekhong river, 240 km N Vientiane, hills c. 250 m, poor settlem[ent], prim[ary] veget[ation] lux, iv.1993, Insomsay Somsy (MFNB, 1). Vietnam: NE region, Bac Giang, Tay Yen Tu Nature Res., 10.vi.2016, at light, 21°11.6'N, 106°45.232'E, G.S. Powell (MSUC, 2). Cao Bang, 22°34.118'N, 105°52.537'E, 1048 m, 12–17.iv.2014, VN9, Cognato, Smith, Pham, ex FIT (MSUC, 1). [Ninh Binh], Cuc Phuong N.P., 20°15.586'N, 105°42.320'E, 147 m, 30.iv–1.v.2005, A. Kun (HNHM, 1). Vinh Phuc, Tam Dao, 930 m, 24–31.viii.2015, J.B. Heppner (FSCA, 1)

##### Diagnosis.

2.5–3.0 mm long (mean = 2.74 mm; n = 5); 2.08–2.31× as long as wide. This species is distinguished by its very stout body; pronotum rounded from dorsal view (type 1); and lack of serrations on pronotum anterior margin; and uniseriate row of sparse, large tubercles on the declivity.

##### Similar species.

*Cyclorhipidion pruinosum*, *C. sisyrnophorum*.

##### Distribution.

‘Borneo’, China (Hainan, Yunnan*), India* (Arunachal Pradesh), Indonesia (Java), Laos*, West Malaysia, New Guinea, Philippines, Thailand, Vietnam*.

##### Host plants.

Recorded from *Castanopsis*, *Lithocarpus*, and *Quercus* (Fagaceae), and probably closely associated with that family.

##### Remarks.

The gallery system has few branches, and small, rather irregular brood chambers in the longitudinal plane (Browne 1961b).

**Figure 44.**
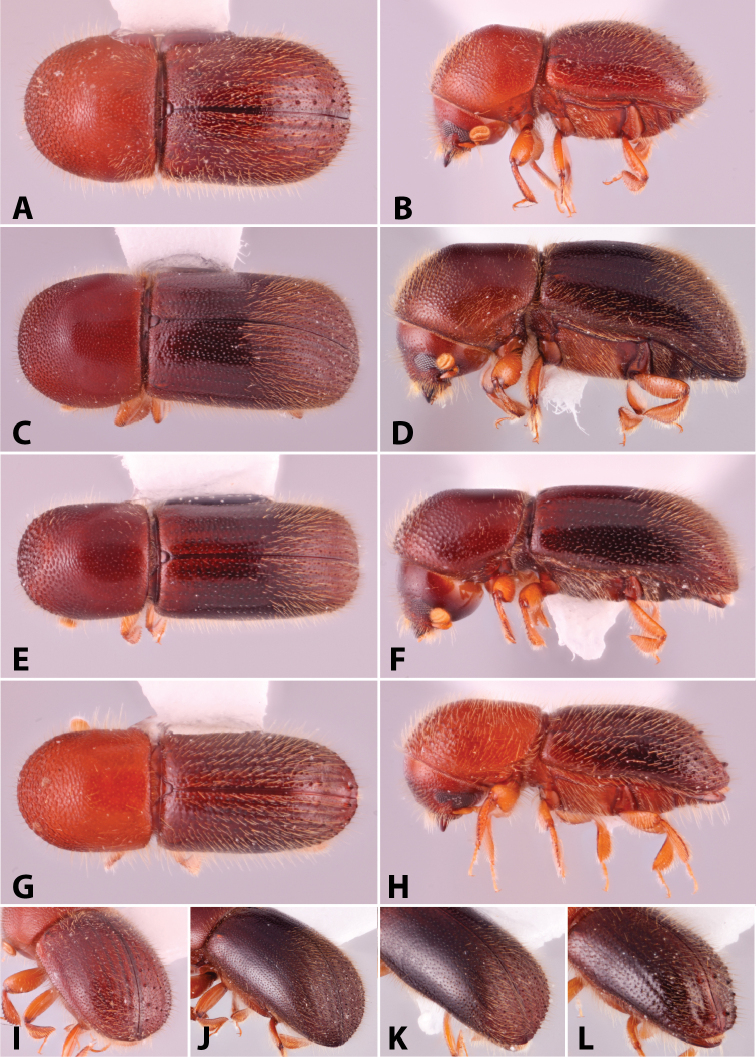
Dorsal, lateral and declivital view of *Cyclorhipidion perpilosellum*, 2.5–3.0 mm (**A, B, I**), *C. petrosum* holotype, 3.9–4.1 mm (**C, D, J**), *C. pilipenne*, 2.5–3.0 mm (**E, F, K**), and *C. pruinosulum*, 2.7–3.5 mm (**G, H, L**).

#### Cyclorhipidion petrosum sp. nov.

Taxon classificationAnimaliaColeopteraCurculionidae

http://zoobank.org/B910C3A1-D70B-447C-AD15-13B62547974F

Fig. 44C, D, J

##### Type material.

***Holotype***, female, Vietnam: Cao Bang, 22°34.118'N, 105°52.537'E, 1048 m, 12–17.iv.2014, VN9, Cognato, Smith, Pham, ex FIT (MSUC). ***Paratypes***, female, Laos: NE, Hua Phan, Ban Saluei, Phu Pan (Mt.), 20°12'N, 104°01'E, 1300–1900 m, 27.iv–1.vi.2011, C. Holzschuh (NHMUK, 1). Thailand: Chiang Mai, Doi Pui, 6.viii.2002, R.A. Beaver, K. Koivisto (RABC, 3); as previous except: 1400 m, 16–20.viii.2004, W. Puranasakul, ex flight intercept trap (NHMUK, 1); as previous except: 27.ix–1.x.2004 (QSBG, 2); as previous except: 29.xi–3.xii.2004 (RABC, 2); as previous except: ex chestnut (RABC, 2); as previous except: 1200–1300 m, 28.vi.2014, S. Sanguansub et al., ex Fagaceae branch (SSC, 2; RABC, 1); as previous except: xii.2004, J. Hulcr, ex *Castanopsis*, uffeID 6603 (UFFE, 1); as previous except: Doi Inthanon, 900 m, 28.vii.2004, A.I. Cognato (RABC, 1); as previous except: Omkoi Wildlife Sanctuary, 28.vi.2013, C. Bateman, uffeID 11757 (UFFE, 8); as previous except uffeID 11758 (UFFE, 2). Loei, Phu Hin Rongkla N. Park Huai Man Daeng Naoi @ trail, 16°57'N, 101°03'E, 14.xii.2002–17.i.2003, G.W. Courtney, ex malaise trap (MSUC, 1). Vietnam: Cao Bang, 22°34.118'N, 105°52.537'E, 1048 m, 12–17.iv.2014, VN9, Cognato, Smith, Pham, ex FIT (MSUC, 1; NHMUK, 1; NMNH, 2).

##### Diagnosis.

3.9–4.1 mm long (mean = 4.02 mm; n = 5); 2.58–2.73× as long as wide. This species is distinguished by large size; declivital slope gentle, gradual; separation between the smooth, shiny elytral disc and shagreened declivity gradual, not sharply distinct; declivital striae weakly impressed, strial punctures small, indistinct; declivital interstriae armed with a row of somewhat confused dense granules; and elytral apex and posterolateral margin armed with granules.

##### Similar species.

*Cyclorhipidion denticauda*, *C. ohnoi*, *C. pilipenne*.

##### Description

**(female).** 3.9–4.1 mm long (mean = 4.02 mm; n = 5); 2.58–2.73× as long as wide. Body dark red-brown. Legs and antennae light brown. ***Head***: epistoma entire, transverse, with a row of hair-like setae. Frons weakly convex to upper level of eyes; surface subshiny, impunctate, alutaceous, rugose. Eyes deeply emarginate just above antennal insertion, upper part smaller than lower part. Submentum narrow, triangular, deeply impressed. Antennal scape regularly thick, as long as club. Pedicel as wide as scape, shorter than funicle. Funicle 4-segmented, segment 1 shorter than pedicel. Club approximately circular and flat, type 3; segment 1 corneous, transverse on anterior face, occupying approximately basal 2/5; segment 2 narrow, corneous; segments 1 and 2 present on posterior face. ***Pronotum***: 0.89× as long as wide. In dorsal view subquadrate, sides convex, type 3, narrowly rounded anteriorly; anterior margin without serrations. In lateral view elongate with disc much longer than anterior slope, type 7, disc flat, summit at apical 2/5. Anterior slope shagreened, with densely spaced, fine, narrow asperities, becoming lower and more strongly transverse towards summit, bearing long, fine, semi-recumbent, hair-like setae. Disc subshiny, alutaceous, densely, finely punctate, finely setose, setae short, erect, hair-like. Lateral margins obliquely costate. Base transverse, posterior angles broadly rounded. ***Elytra***: 1.82× as long as wide, 2.05× as long as pronotum. Scutellum large, broad, linguiform, shiny, flush with elytra, flat. Elytral base transverse, edge oblique, humeral angles rounded, parallel-sided in basal 4/5, then broadly rounded to apex. Disc flat, shiny, striae and interstriae densely setose, setae long, semi-recumbent, hair-like, striae and interstriae strongly confused, indistinguishable; striae and interstriae not impressed, minutely punctate, punctures strongly confused, separated by 2–5 diameters of a puncture. Declivity occupying 2/5 of elytra, declivital slope gradual, rounded, strongly shagreened, separation between the smooth, shiny disc and shagreened declivity gradual, not sharply distinct; six striae present, striae 2 equidistant between 1 and 3, striae weakly impressed, punctures small, shallow, indistinct, subcontiguous, shagreened, much larger than on disc; interstriae feebly convex, interstriae setose, setae dense, long, semi-erect hair-like; interstriae impunctate, coarsely granulate, granules dense, confused, variably sized. Posterolateral margin rounded, granulate. ***Legs***: procoxae contiguous; prosternal coxal piece tall and pointed. Protibiae semi-circular with evenly rounded outer edge, broadest at apical 1/3; posterior face smooth; apical 1/3 of outer margin with 11 moderate socketed denticles, their length approximately as long as basal width. Meso- and metatibiae broad, flattened; outer margin evenly rounded with 14 moderate socketed denticles.

##### Etymology.

L. *petrosus* = rocky, stony. In reference to the granular declivity. An adjective.

##### Distribution.

Laos, Thailand, Vietnam.

##### Host plants.

This species has only been recorded from *Castanopsis* (Fagaceae).

#### Cyclorhipidion pilipenne

Taxon classificationAnimaliaColeopteraCurculionidae

(Eggers, 1940)

Fig. 44E, F, K

Xyleborus pilipennis Eggers, 1940: 140.
Cyclorhipidion pilipenne (Eggers): Wood and Bright 1992: 701.

##### Type material.

***Paratype*** (NMNH).

##### New records.

China: S Yunnan, Xishuangbanna, 23 km NW Jinghong, vic. Na Ban village (NNNR), 22°10'N, 100°39'E, 700–1000 m, v–vii.2009, L. Meng (RABC, 2); Xishuangbanna Sanchahe Nat. Res., 22°09.784'N, 100°52.256'E, 2186 m, 29–30.v.2008, A. Cognato, ex *Quercus* (MSUC, 1). Vietnam: Cao Bang, 22°36.454'N, 105°52.083'E, 1661 m, 15.iv.2014, VN39, Cognato, Smith, Pham, 3–6 cm branches (MSUC, 10). Vietnam: Lao Cai, Hoang Lien N.P., 22.35, 103.77, 1500–2000 m, 19.v.2019, VN180, S.M. Smith, A.I. Cognato, ex 5 cm branch (MSUC, 17). Thua Thien-Hue, Bach Ma N.P., 16.20089, 107.84824, 919 m, 16.ii.2017, VN67, A.I. Cognato, T.A. Hoang, ex 2 cm diameter & 8 cm diameter (MSUC, 2).

##### Diagnosis.

2.5–3.0 mm long (mean = 2.86 mm; n = 5); 2.78–3.0× as long as wide. This species is distinguished by moderate size; declivital slope gentle, gradual; separation between the smooth, shiny elytral disc and shagreened declivity gradual, not sharply distinct; declivital striae weakly impressed, punctures small, indistinct; declivital interstriae armed with a row of moderately spaced uniseriate granules; and elytral apex and posterolateral margin granulate.

##### Similar species.

*Cyclorhipidion denticauda*, *C. ohnoi*, *C. petrosum*.

##### Distribution.

China* (Yunnan), Indonesia (Java), Thailand, Vietnam*.

##### Host plants.

Recorded only from *Castanopsis* (Beaver et al. 2014) and *Quercus* (Fagaceae).

#### Cyclorhipidion pruinosulum

Taxon classificationAnimaliaColeopteraCurculionidae

(Browne, 1979)

Fig. 44G, H, L

Xyleborus pruinosulus Browne, 1979 (in Beaver and Browne 1979): 611.
Cyclorhipidion pruinosulum (Browne): Beaver 1995a: 203.

##### Type material.

***Holotype*** (NHMUK).

##### New records.

Vietnam: Dong Nai, Cat Tien N.P., 11.40817, 107.38098, 134 m, 20–22.ii.2017, VN81, A.I. Cognato, T.A. Hoang, ex FIT (MSUC, 2).

##### Diagnosis.

2.7–3.5 mm long (mean = 2.92 mm; n = 5); 2.5–2.9× as long as wide. This species is distinguished by its unique elytral sculpturing: declivital interstriae 1 armed by a large denticle near the base and a small spine near the apex with the area in between appearing concave.

##### Similar species.

None.

##### Distribution.

Brunei, East & West Malaysia, Thailand, Vietnam*.

##### Host plants.

Unknown.

#### Cyclorhipidion pruinosum

Taxon classificationAnimaliaColeopteraCurculionidae

(Blandford, 1896)

Fig. 45A, B, I

Xyleborus pruinosus Blandford, 1896b: 214.
Cyclorhipidion pruinosum (Blandford): Wood and Bright 1992: 701.
Xyleborus arcticollis Blandford, 1896b: 217. Synonymy: Browne 1955: 352.
Xyleborus decipiens Eggers, 1923: 182. Synonymy: Browne 1955: 352.

##### Type material.

***Holotype****Xyleborus pruinosus* (NHMUK).

##### Diagnosis.

3.5–4.1 mm long (mean = 3.9 mm; n = 5); 2.19–2.56× as long as wide. This species is distinguished by its stout body; anterior pronotum margin with a row of 5–7 serrations; antennal club type 3; and elytral interstriae granulate.

##### Similar species.

*Cyclorhipidion perpilosellum*, *C. sisyrnophorum*.

##### Distribution.

Chagos Is, Indonesia (Sumatra), East & West Malaysia, Philippines, Thailand.

##### Host plants.

Polyphagous, but with a distinct preference for trees of the family Burseraceae (Browne 1961b).

##### Remarks.

Browne (1961b) describes the gallery system and aspects of the biology.

**Figure 45.**
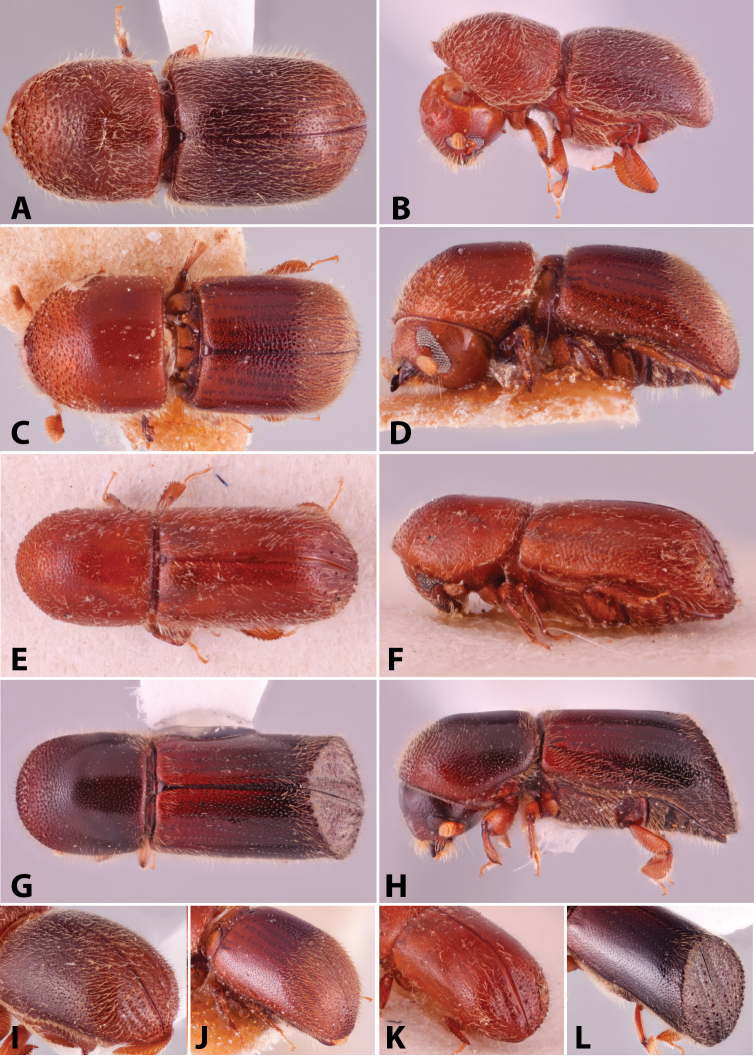
Dorsal, lateral and declivital view of *Cyclorhipidion pruinosum*, 3.5–4.1 mm (**A, B, I**), *C. sisyrnophorum*, 3.3–4.0 mm (**C, D, J**), *C. tenuigraphum*, 2.7–3.0 mm (**E, F, K**), and *C. truncaudinum* holotype, 3.9–4.1 mm (**G, H, L**).

#### Cyclorhipidion sisyrnophorum

Taxon classificationAnimaliaColeopteraCurculionidae

(Hagedorn, 1910)

Fig. 45C, D, J

Xyleborus sisyrnophorus Hagedorn, 1910a: 7.
Cyclorhipidion sisyrnophorum (Hagedorn): Wood and Bright 1992: 703.

##### Type material.

***Holotype*** (SDEI).

##### New records.

India: N. Andaman, C.F.C. Beeson, 18.iii.193 [*sic*], ex unknown wood (NMNH, 3).

##### Diagnosis.

3.3–4.0 mm long (mean = 3.61 mm; n = 8); 2.2–2.5× as long as wide. This species is distinguished by the type 5 antennal club which lacks visible sutures on both the anterior and posterior faces; anterior margin of pronotum with a distinct row of five serrations; and declivital interstriae 2 granulate and stout form.

##### Similar species.

*Cyclorhipidion perpilosellum*, *C. pruinosum*.

##### Distribution.

‘Borneo’, India (Andaman Is), Indonesia (Sumatra), East & West Malaysia.

##### Host plants.

Recorded from *Dryobalanops* (Dipterocarpaceae), *Xerospermum* (Sapindaceae) and an unidentified species of Burseraceae (Beeson 1961; Browne 1961b). Presumably polyphagous.

#### Cyclorhipidion tenuigraphum

Taxon classificationAnimaliaColeopteraCurculionidae

(Schedl) stat. res.

Fig. 45E, F, K

Xyleborus tenuigraphus Schedl, 1953c: 29.
Cyclorhipidion tenuigraphus (Schedl): Beaver and Liu 2010: 24 (as a synonym of C. fukiense).

##### Type material.

***Lectotype****Xyleborus tenuigraphus* (NHMW).

##### New records.

China: Yunnan, Lijiang, v.1975, Zhizhong Zhang, ex *Pistacia weinmannifolia* (NMNH, 1). India: Assam, 4 mi N. Cherrapunji, 1378 m, 3.x.1961, E.S. Rose, D.Q. Cavagnaro (CASC, 1). Vietnam: Cao Bang, 22°34.118'N, 105°52.537'E, 1048 m, 12–17.iv.2014, VN9, Cognato, Smith, Pham, ex FIT (MSUC, 1).

##### Diagnosis.

2.7–3.0 mm long (mean = 2.84 mm; n = 4); 2.5–3.0× as long as wide. This species is distinguished by the short, steep declivity that is approximately 25% of total elytral length, armed with large tubercles on interstriae 1 and 3, interstriae 2 granulate on apical 1/3; posterolateral margins rounded; and declivital interstriae 1 and 2 setae in two confused rows (Table 1).

##### Similar species.

This species is a part of a challenging species group consisting of *C. bodoanum*, *C. distinguendum*, *C. inarmatum*, *C. pelliculosum*, and *C. xeniolum* (Table 1).

##### Distribution.

China (Fujian, Yunnan*), India* (Assam), Vietnam*.

##### Host plants.

This species has only been recorded from *Pistacia* (Anacardiaceae).

##### Remarks.

In his description Schedl lists the species as 2.3 mm long. The lectotype is 2.7 mm long. *Xyleborus tenuigraphum* was previously considered a synonym of *X. fukiense* by Beaver and Liu (2010) based on comparison between the lectotype, a specimen of *C. fukiense* compared to the holotype by Schedl, and an additional specimen with the same locality data as the homotype but identified by Schedl as *X. tenuigraphum*. Schedl clearly erred in his identification of his homotype specimen as the two species are distinguished by the characters listed above in the diagnosis and Table 1.

#### Cyclorhipidion truncaudinum sp. nov.

Taxon classificationAnimaliaColeopteraCurculionidae

http://zoobank.org/383E3A7E-27DC-4714-8B8E-AD909DDF756F

Fig. 45G, H, L

##### Type material.

***Holotype***, female, Vietnam: Cao Bang, 22°36.454'N, 105°52.083'E, 1661 m, 15.iv.2014, VN39, Cognato, Smith, Pham, ex 3–6 cm branches (MSUC). ***Paratypes***, female, as holotype (MSUC, 1; VMNH, 1).

##### Diagnosis.

4.0 mm long (mean = 4.0 mm; n = 3); 2.67–2.86× as long as wide. This species is distinguished by the large size; truncate declivity surrounded by a granulate circumdeclivital costa; pronotum subquadrate from dorsal view (type 3); declivital interstrial punctures replaced by a single row of tubercles; declivital strial punctures large, distinct; declivital face with three striae, distinctly sulcate on basal 1/2, surface rugose, coarsely sculptured and appearing undulating; declivital striae impressed, striae 1 more deeply impressed; and interstriae 1 inflated on apical 1/3 and interstriae 2 and 3 flat.

##### Similar species.

*Cyclorhipidion amasoides*, *C. amputatum*, *C. circumcisum*, *C. muticum*, *C. umbratum*, all of which are large and have an obliquely truncate or truncate declivity.

##### Description

**(female).** 4.0 mm long (mean = 4.0 mm; n = 3); 2.67–2.86× as long as wide. Body dark red-brown. Legs and antennae dark brown. ***Head***: epistoma entire, transverse, with a row of hair-like setae. Frons weakly convex to upper level of eyes; surface subshiny, impunctate, alutaceous, rugose. Eyes deeply emarginate just above antennal insertion, upper part smaller than lower part. Submentum narrow, triangular, deeply impressed. Antennal scape regularly thick, as long as club. Pedicel as wide as scape, shorter than funicle. Funicle 4-segmented, segment 1 as long as pedicel. Club approximately circular and flat, type 3; segment 1 corneous, transverse on anterior face, occupying approximately basal 2/5; segment 2 narrow, corneous; segments 1 and 2 present on posterior face. ***Pronotum***: 0.95–0.97× as long as wide. In dorsal view subquadrate, sides convex, type 3, narrowly rounded anteriorly; anterior margin without serrations. In lateral view elongate with disc much longer than anterior slope, type 7, disc flat, summit at apical 2/5. Anterior slope shagreened, with densely spaced, fine, narrow asperities, becoming lower and more strongly transverse towards summit, bearing long, fine, semi-recumbent, hair-like setae. Disc subshiny, alutaceous, densely, finely punctate, finely setose, setae short, erect, hair-like. Lateral margins obliquely costate. Base transverse, posterior angles broadly rounded. ***Elytra***: 1.62–1.87× as long as wide, 1.7–1.92× as long as pronotum. Scutellum large, broad, linguiform, shiny, flush with elytra, flat. Elytral base transverse, edge oblique, humeral angles rounded, parallel-sided in basal 4/5, then sharply angulate to apex. Disc flat, shiny, striae and interstriae densely setose, setae long, semi-recumbent, hair-like, striae and interstriae strongly confused, indistinguishable; striae and interstriae not impressed, minutely punctate, punctures strongly confused, separated by 2–5 diameters of a puncture. Declivity occupying 1/3 of elytra, truncate, face alutaceous, subshiny, appearing undulating, sulcate on basal 1/2; three striae present, striae distinctly impressed, striae 1 more deeply than 2 or 3, strial punctures large, distinct, subcontiguous, shagreened, much larger than on disc; interstriae setose, setae dense, long, semi-erect hair-like; interstriae convex, impunctate, coarsely uniseriate tuberculate, tubercles increasing in size apically; interstriae 1 strongly convex on apical 1/3. Posterolateral margin forming a circumdeclivital carina; carina granulate, setose, setae long, fine, erect. ***Legs***: procoxae contiguous; prosternal coxal piece tall, conical. Protibiae semi-circular with evenly rounded outer edge, broadest at apical 1/3; posterior face smooth; apical 1/3 of outer margin with ten moderate socketed denticles, their length approximately as long as basal width. Meso- and metatibiae broad, flattened; outer margin evenly rounded with 15 moderate socketed denticles.

##### Etymology.

In reference to the likeness to *Truncaudum*. An adjective.

##### Distribution.

Vietnam.

##### Host plants.

Recorded from *Lithocarpus* (Fagaceae).

#### Cyclorhipidion umbratum

Taxon classificationAnimaliaColeopteraCurculionidae

(Eggers, 1941)

Fig. 46A, B, I

Xyleborus umbratus Eggers, 1941b: 223.
Cyclorhipidion umbratum (Eggers): Wood and Bright 1992: 704.

##### Type material.

***Holotype*** (ZMFK).

##### New records.

China: Fujian, Jianyang, 2.v.1978, Fusheng Huang, ex *Lithocarpus dealbatus* (NMNH, 3).

##### Diagnosis.

3.8–4.1 mm long (mean = 4.0 mm; n = 4); 2.53–2.86× as long as wide. This species is distinguished by the large size; truncate declivity surrounded by a granulate circumdeclivital costa; pronotum subquadrate from dorsal view (type 3); declivital interstrial punctures replaced by a single row of tubercles; declivital strial punctures large, distinct; declivital face with three striae, feebly sulcate on basal 1/4, surface smooth; and declivital striae clearly, uniformly impressed; and interstriae inflated.

##### Similar species.

*Cyclorhipidion amasoides*, *C. amputatum*, *C. circumcisum*, *C. muticum*, and *C. truncaudinum*, all of which are large and have an obliquely truncate or truncate declivity.

##### Distribution.

China (Fujian).

##### Host plants.

This species is only known from *Lithocarpus* (Fagaceae).

**Figure 46.**
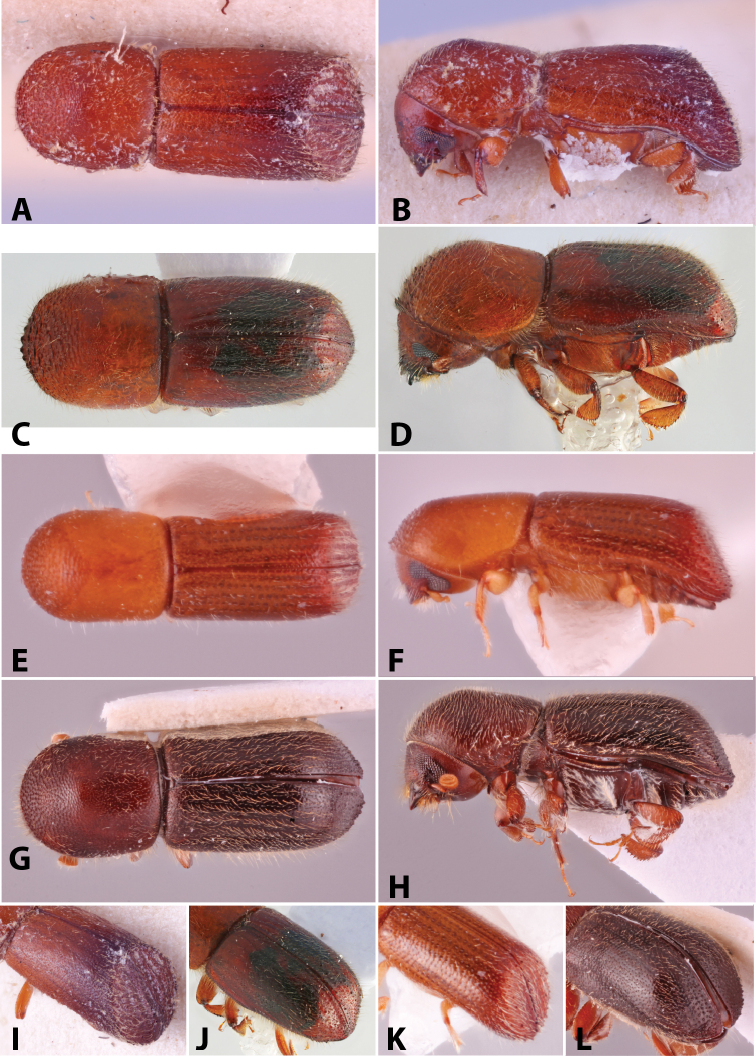
Dorsal, lateral and declivital view of *Cyclorhipidion umbratum* holotype, 3.8 mm (**A, B, I**), *C. vigilans*, 5.5 mm (**C, D, J**), *C. xeniolum* holotype, 1.65–1.8 mm (**E, F, K**), and *C. xyloteroides*, 3.25 mm (**G, H, L**).

#### Cyclorhipidion vigilans

Taxon classificationAnimaliaColeopteraCurculionidae

(Schedl, 1939)

Fig. 46C, D, J

Xyleborus vigilans Schedl, 1939b: 43.
Cyclorhipidion vigilans (Schedl): Wood and Bright 1992: 704.

##### Type material.

***Lectotype*** (NHMW). Not examined.

##### Diagnosis.

5.5 mm long (mean = 5.5 mm; n = 5); 2.45–2.48× as long as wide (Sittichaya et al. 2019). This species is distinguished by the large size; anterior margin of pronotum with a short continuously elevated recurved carina armed with 4–6 medium sized serrations; declivital interstriae 2 granulate; and rounded declivity.

##### Similar species.

*Fortiborus* spp.

##### Distribution.

Indonesia (Java), East & West Malaysia, Thailand.

##### Host plants.

Recorded only from *Horsfieldia* (Myristicaceae) (Schedl 1939b).

#### Cyclorhipidion xeniolum sp. nov.

Taxon classificationAnimaliaColeopteraCurculionidae

http://zoobank.org/62B53B84-5131-4E75-B0A3-7AF0430C8B88

Fig. 46E, F, K

##### Type material.

***Holotype***, female, China: Yunnan, Xishuangbanna, Sanchahe Nat. Res., 22°09.784'N, 100°52.256'E, 2186 m, 29–30.v.2008, A.I. Cognato (IZAS). ***Paratypes***, female, Thailand: Chiang Mai, Doi Pui, 1400 m, 20–24.xii.2004, W. Puranasakul, ex flight intercept trap (QSBG, 1); as previous except: 21.ii–14.iii.2005, ex EtOH trap (QSBG, 1); as previous except: 14.iii–4.iv.2005 (RABC, 1); as previous except: ii. 2005, ex *Castanopsis* sp. (RABC, 2).

##### Diagnosis.

1.65–1.8 mm long (mean = 1.71 mm; n = 5); 3.09–3.4× as long as wide. This species is distinguished by the short, obliquely truncate and flat declivity that is approximately 25% of total elytral length, armed with large tubercles on interstriae 1 and 3; interstriae 2 always unarmed; posterolateral margins rounded; declivital interstriae 1 and 2 setae uniseriate (Table 1).

##### Similar species.

This species is a part of a challenging species group consisting of *C. bodoanum*, *C. distinguendum*, *C. inarmatum*, *C. pelliculosum* and *C. tenuigraphum* (Table 1).

##### Description

**(female).** 1.65–1.8 mm long (mean = 1.71 mm; n = 5); 3.09–3.4 × as long as wide. Body, antennae, and legs light brown. Elytra slightly darker than rest of body. ***Head***: epistoma entire, transverse, with a row of hair-like setae. Frons weakly convex to upper level of eyes; surface subshiny, impunctate, alutaceous, finely rugose. Eyes deeply emarginate just above antennal insertion, upper part smaller than lower part. Submentum narrow, triangular, deeply impressed. Antennal scape short and thick, shorter than club. Pedicel narrower than scape, as long as funicle. Funicle 3-segmented, segment 1 shorter than pedicel. Club approximately circular and flat, type 3; segment 1 corneous, transverse on anterior face, occupying approximately 2/5 of club; segment 2 narrow, soft; segments 1 and 2 present on posterior face. ***Pronotum***: 1.32× as long as wide. In dorsal view very elongate, rounded frontally, type 9, sides parallel on basal 3/4; anterior margin without serrations. In lateral view elongate with disc much longer than anterior slope, type 8, disc flat, summit at apical 1/4. Anterior slope shagreened, with densely spaced, fine, narrow asperities, becoming lower and more strongly transverse towards summit, bearing long, fine, semi-recumbent, hair-like setae. Disc subshiny, alutaceous, densely, finely punctate, finely setose, setae short, erect, hair-like. Lateral margins obliquely costate. Base transverse, posterior angles broadly rounded. ***Elytra***: 1.79× as long as wide, 1.35× as long as pronotum. Scutellum moderate, broad, linguiform, flush with elytra, flat, shiny. Elytral base transverse, edge oblique, humeral angles rounded, parallel-sided in basal 4/5, then broadly rounded to apex. Disc flat, shiny, striae and interstriae moderately setose, setae long, semi-recumbent, hair-like, striae not impressed, punctures large, uniseriate, spaced by one diameter of a puncture; interstriae not impressed, minutely punctate, punctures less than 1/2 size of strial punctures, strongly confused, separated by more than five diameters of a puncture. Declivity occupying 1/4 of elytra, obliquely truncate, declivital slope very steep, flat, weakly medially concave between suture and interstriae 3, strongly shagreened, separation between the smooth, shiny disc and shagreened declivity distinct; three striae present, striae 2 closer to striae 1 than striae 3, striae weakly impressed, punctures very large, shallow, subcontiguous, shagreened, much larger than on disc; interstriae 1 and 3 feebly convex, interstriae 2 flat, interstriae minutely punctate, punctures seriate, interstriae 1 and 2 bearing a single row of setae on declivital face, interstriae 3 and 4 bearing two rows of setae, setae long, semi-erect; interstriae 1 with three tubercles, two on apical 1/4 and one on basal 1/3, interstriae 3 with three equally spaced tubercles, one at base, midpoint and on apical 1/4, interstriae 2 unarmed. Posterolateral margin rounded, granulate, extending to interstriae 7. ***Legs***: procoxae contiguous; prosternal coxal piece tall and pointed. Protibiae semi-circular with evenly rounded outer edge, broadest at apical 1/3; posterior face smooth; apical 1/3 of outer margin with six moderate socketed denticles, their length approximately as long as basal width. Meso- and metatibiae broad, flattened; outer margin evenly rounded with eight and seven moderate socketed denticles, respectively.

##### Etymology.

L. *xenium* = a gift to a guest; -*olum* = diminutive suffix. In reference to AIC’s appreciation of finding such a dainty species. A noun in apposition.

##### Distribution.

China (Yunnan), Thailand.

##### Host plants.

Known only from *Castanopsis* (Fagaceae).

#### Cyclorhipidion xyloteroides

Taxon classificationAnimaliaColeopteraCurculionidae

(Eggers, 1939)

Fig. 46G, H, L

Xyleborus xyloteroides Eggers, 1939b: 120.
Cyclorhipidion xyloteroides (Eggers): Beaver and Liu 2010: 25.

##### Type material.

***Holotype*** (TARI).

##### New records.

Taiwan: Taichung Co., Dasyueshan Natl Forest, ex EtOH trap, 11.viii.2013, C-S. Lin (RABC, 1). Chiayi Co., Alishan, 2400 m 12–16.vi.1965, T. Maa, K.S. Lin (BPBM, 1). Ilan Co., Fushan, 2000 m, 27.vi.1995, A. Warneke, ex light trap (RABC, 1); as previous except: 26.vii.1995 (RABC, 1).

##### Diagnosis.

3.25 mm long (n = 1); 2.7× as long as wide. This species is distinguished by the elytral disc convex; declivity convex; posterolateral margin costate with a row of larger granules; elytral apex granulate; moderately large size; pronotal disc shiny, finely, densely punctured; declivital striae not impressed, both striae and interstriae punctures granulate, and of similar size, a row of slightly larger granules on interstriae 1 and 3, and a weaker row on interstriae 2; and declivital vestiture very fine and short.

##### Similar species.

*Cyclorhipidion armiger*, *C. miyazakiense*, *C. obesulum*.

##### Distribution.

Taiwan.

##### Host plants.

Unknown.

### *Debus* Hulcr & Cognato, 2010

#### Debus

Taxon classificationAnimaliaColeopteraCurculionidae

Hulcr & Cognato, 2010

Debus Hulcr & Cognato, 2010a: 13.

##### Type species.

*Xyleborus emarginatus* Eichhoff, 1878; original designation.

##### Diagnosis.

2.2–5.4 mm, 2.68–3.85× as long as wide. *Debus* is distinguished by the pronotal disc flat and elongate, pronotum from dorsal view long, rounded frontally (type 9, rarely type 7); elytral apex emarginate (except *D. adusticollis* in our region); elytra typically strongly excavated and explanate; first declivital interstriae broadened, laterally displacing strial punctures; protibiae distinctly triangular with fewer than six large denticles on lateral margin. In addition, mycangial tufts are absent, procoxae are contiguous and scutellum flat and flush with the elytra.

##### Similar genera.

*Streptocranus*.

##### Distribution.

Common in tropical forests throughout South Asia to the far reaches of the Pacific Ocean.

##### Gallery system.

This usually has a transverse surface gallery between the bark and wood, part of which is expanded by the larvae into a brood chamber in which many of them develop. Further branching tunnels penetrate directly into the wood. These too develop brood chambers in the longitudinal plane. Brood development proceeds normally in the wood, if the tree is debarked. In some species (e.g., *D. adusticollis*), surface galleries and brood chambers have not been observed (Kalshoven 1959b; Browne 1961b).

#### Key to *Debus* species (females only)

**Table:** 

1	Elytral apex never explanate; elytral apices not prolonged beyond abdominal apex (Fig. 47A, G)	**2**
–	Elytral apex explanate; elytral apices produced beyond the abdominal apex with posterolateral extensions (Fig. 47C)	**4**
2	Elytral apex entire; declivital sulcus deep	*** adusticollis ***
–	Elytral apex emarginate; declivital sulcus shallow	**3**
3	Declivity minutely, finely punctate; smaller, 2.5–3.2 mm and more elongate, 3.2–3.6× as long as wide	*** pumilus ***
–	Declivity densely, coarsely punctate; larger, 3.9–4.6 mm, and stouter, 2.7–2.9 × as long as wide.	*** detritus ***
4	Posterolateral extensions of elytra short, less than the width of apical emargination; declivity shallowly excavated (Fig. 48G)	**5**
–	Posterolateral extensions of elytra long, at least as long as width of apical emargination; declivity deeply excavated (Fig. 47C)	**6**
5	Declivity impunctate except for a single row of punctures running from the upper margin to the inner margin of the second declivital spine and thence to the apical emargination	*** shoreae ***
–	Declivity clearly, confusedly punctate	*** emarginatus ***
6	Elytra distinctly tapering apically from 1/3 length from base, a slight lateral constriction just behind second declivital teeth; length 3.3–5.4 mm	*** amphicranoides ***
–	Elytra weakly tapering only in posterior 1/3 or less, lacking a lateral constriction; usually smaller, not more than 4.0 mm	**7**
7	Larger species, 3.7–3.9 mm; upper pair of spines on declivity short, conical, separated from lower pair by approximately the same distance as the second pair from the elytral apex	*** birmanus ***
–	Smaller species, 2.2–2.5 mm; upper pair of spines on declivity longer, more sharply pointed, usually separated from the lower pair by a shorter distance than between the lower pair and the elytral apex	*** quadrispinus ***

#### Debus adusticollis

Taxon classificationAnimaliaColeopteraCurculionidae

(Motschulsky, 1863)

Fig. 47A, B, I

Tomicus adusticollis Motschulsky, 1863: 514.
Debus adusticollis (Motschulsky): Hulcr and Cognato 2010a: 14.
Xyleborus vestitus Schedl, 1931: 341. Synonymy: Wood 1989: 176.

##### Type material.

***Holotype*** (ZMMU). Not examined.

##### New records.

Laos: Vientiane, Ban Van Eue, 15.ii.1966, native collector, ex malaise trap (BPBM, 2).

##### Diagnosis.

2.2–2.7 mm (mean = 2.52 mm; n = 5); 3.57–3.85× as long as wide. This species is distinguished by the elytral apex entire, never explanate, appearing flat and broad; declivital sulcus deep; and small size.

##### Similar species.

*Debus detritus*, *D. pumilus*.

##### Distribution.

Brunei, China (Yunnan), Indonesia (Java), Laos*, East & West Malaysia, Philippines, Sri Lanka, Thailand.

##### Host plants.

Polyphagous (Browne 1961b).

**Figure 47.**
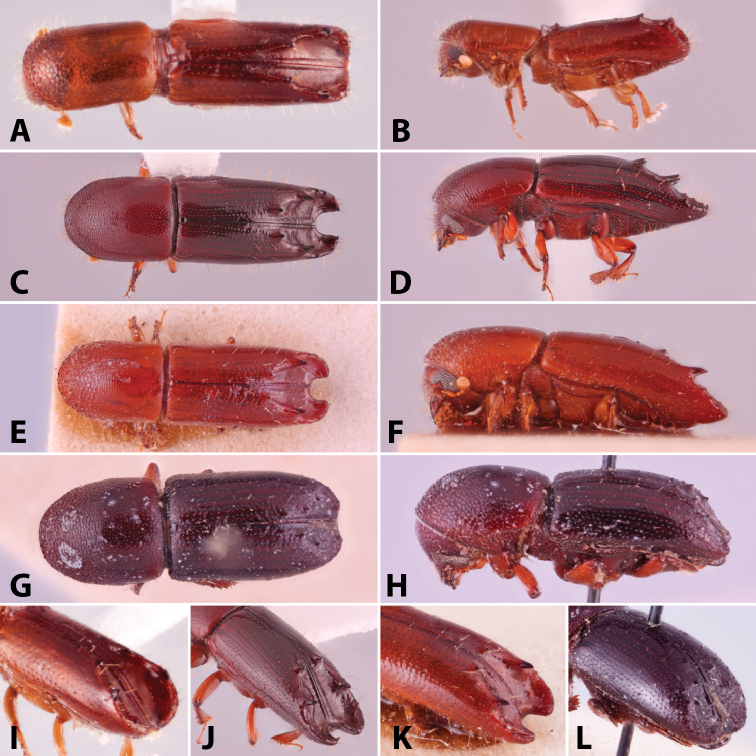
Dorsal, lateral and declivital view of *Debus adusticollis*, 2.2–2.7 mm (**A, B, I**), *D. amphicranoides*, 3.3–5.4 mm (**C, D, J**), *D. birmanus* paratype, 3.7–3.9 mm (**E, F, K**), and *D. detritus* holotype, 3.9–4.6 mm (**G, H, L**).

#### Debus amphicranoides

Taxon classificationAnimaliaColeopteraCurculionidae

(Hagedorn, 1908)

Fig. 47C, D, J

Xyleborus amphicranoides Hagedorn, 1908: 379.
Debus amphicranoides (Hagedorn): Hulcr 2010: 107.
Xyleborus amphicranoides latecavatus Eggers, 1927b: 95. Synonymy: Wood and Bright 1992: 711.
Xyleborus amphicranoides parvior Browne, 1981b: 601. Synonymy: Wood and Bright 1992: 711.

##### Type material.

***Syntypes****Xyleborus amphicranoides* (SDEI, 2). ***Lectotype****Xyleborus a. latecavatus* (NMNH).

##### New records.

China: S Yunnan, Xishuangbanna, 20 km NW Jinghong, vic. Man Dian (NNNR), 22°07.80'N, 100°40.05'E, 730 m, forest, 6.vi.2008, A. Weigel (RABC, 1). Vietnam: Ninh Binh, Cuc Phuong N.P., 7.iii.2018, 20.34932, 105.59669, 431 m, A.I. Cognato, S.M. Smith, VN 130, ex 8 cm diameter bole (MSUC, 1).

##### Diagnosis.

3.3–5.4 mm long (mean = 4.26 mm; n = 9); 3.23–3.6× as long as wide. This species is distinguished by the posterolateral extensions of elytra long, as long as width of apical emargination; apex of posterolateral extensions with a denticle; declivity strongly excavated; and large size.

##### Similar species.

*Debus birmanus*.

##### Distribution.

China* (Yunnan), Indonesia (Java, Mentawai Is, Sumatra, Sulawesi), Laos, East & West Malaysia, Philippines, Thailand, Vietnam*.

##### Host plants.

Polyphagous (Browne 1961b; Ohno 1990).

##### Remarks.

*Xyleborus amphicranoides parvior* has been considered to be a synonym of *D. amphicranoides*. As noted by Browne (1981b) in his description, the species is morphologically identical to *D. amphicranoides* but smaller in size, 3.2–3.4 mm long (Browne 1981b). Additional specimens from China and Thailand (RABC) measure 3.3–3.8 mm (mean = 3.55, n = 2), 3.39–3.4× as long as wide. Typical *Debus amphicranoides* are larger, 4.8–5.4 mm long (mean = 4.97 mm; n = 5); 3.23–3.6× as long as wide. The species are not diagnosable from each other except in body length. It is possible that they are different species but further investigation with DNA sequence data will be necessary to resolve species limits.

#### Debus birmanus

Taxon classificationAnimaliaColeopteraCurculionidae

(Eggers, 1930)

Fig. 47E, F, K

Xyleborus birmanus Eggers, 1930: 200.
Debus birmanus (Eggers): Hulcr 2010: 108.

##### Type material.

***Holotype*** (FRI), ***paratype*** (NHMW, 1).

##### Diagnosis.

3.7–3.9 mm long (mean = 3.8 mm; n = 2); 3.25–3.36× as long as wide. This species is distinguished by the posterolateral extensions of elytra long, as long as width of apical emargination; apex of posterolateral extensions unarmed by a denticle; declivity strongly excavated; and moderate size.

##### Similar species.

*Debus amphicranoides*.

##### Distribution.

West Malaysia, Myanmar, Thailand.

##### Host plants.

Polyphagous (Beeson 1930; Browne 1961b).

#### Debus detritus

Taxon classificationAnimaliaColeopteraCurculionidae

(Eggers, 1927)

Fig. 47G, H, L

Xyleborus detritus Eggers, 1927a: 402.
Debus detritus (Eggers): Beaver 2011: 284.
Xyleborus maniensis Browne, 1981a: 130. Synonymy: Beaver 2011: 284.

##### Type material.

***Holotype****Xyleborus detritus* (NHMW). ***Holotype****Xyleborus maniensis* (NHMUK).

##### Diagnosis.

3.9–4.6 mm long (mean = 4.22 mm; n = 5); 2.69–2.93× as long as wide. This species is distinguished by the elytral apex emarginate, never explanate, appearing flat and broad; declivital sulcus shallow; and large size.

##### Similar species.

*Debus adusticollis*, *D. pumilus*.

##### Distribution.

Indonesia (Java), East Malaysia, Thailand.

##### Host plants.

Unknown.

#### Debus emarginatus

Taxon classificationAnimaliaColeopteraCurculionidae

(Eichhoff, 1878)

Fig. 48A, B, I

Xyleborus emarginatus Eichhoff, 1878a: 392.
Debus emarginatus (Eichhoff): Hulcr and Cognato 2010a: 14.
Xyleborus exesus Blandford, 1894b: 119. Synonymy: Hulcr 2010: 111.
Ips cinchonae Veen, 1897: 135. Synonymy: Kalshoven 1959a: 96.
Xyleborus cordatus Hagedorn, 1910a: 12. Synonymy: Schedl 1942c: 6.
Xyleborus palmeri Hopkins, 1915a: 54. Synonymy: Hulcr 2010: 111.
Xyleborus terminaliae Hopkins, 1915a: 54. Synonymy: Hulcr 2010: 110.
Xyleborus emarginatus semicircularis Schedl, 1973: 92. Synonymy: Wood 1989: 176.

##### Type material.

***Syntype****Xyleborus emarginatus* (MIZ). ***Syntypes****Xyleborus exesus* (NHMUK, 2). ***Holotype****Xyleborus palmeri* (NMNH). ***Holotype****Xyleborus terminaliae* (NMNH).

##### New records.

China: N Guangxi reg., Miaoershan, S slope, 1300–2000 m, 25–28.vi.1997, Bolm (RABC, 1).

##### Diagnosis.

3.3–3.6 mm long (mean = 3.48 mm; n = 4); 2.83–3.0× as long as wide. This species is distinguished by the posterolateral extensions of elytra short, less than the width of apical emargination, and declivity shallowly excavated; declivity clearly, confusedly punctate.

This species is very similar to *D. shoreae* and is distinguished by the punctation of the declivity.

##### Similar species.

*Debus quadrispinus*, *D. shoreae*.

##### Distribution.

From India and southern China through southeast Asia, the Philippines and Indonesia to New Guinea and the Solomon Islands in the East, northwards to Japan. Recorded in the study region from China (Fujian, Guangxi*, Guizhou, Hubei, Hunan, Shaanxi, Shanxi, Sichuan, Xizang, Yunnan), India (Nicobar Is), Laos, Taiwan, Thailand, Vietnam.

##### Host plants.

Strongly polyphagous (e.g., Browne 1961b; Ohno 1990; Wood and Bright 1992).

##### Remarks.

Browne (1961b) provides further information on the habits of the species.

**Figure 48.**
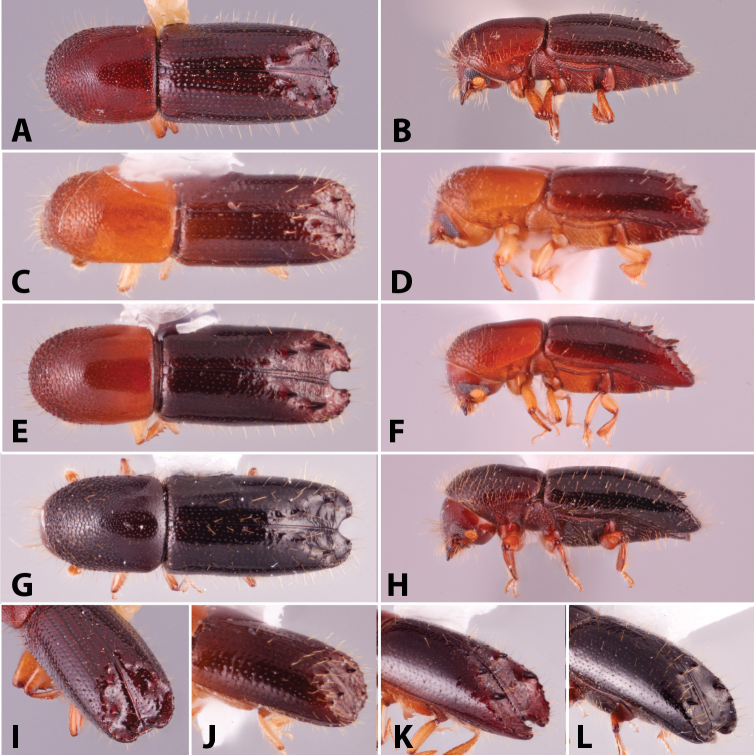
Dorsal, lateral and declivital view of *Debus emarginatus*, 3.3–3.6 mm (**A, B, I**), *D. pumilus*, 2.5–3.2 mm (**C, D, J**), *D. quadrispinus*, 2.2–2.5 mm (**E, F, K**), and *D. shoreae*, 3.0–3.8 mm (**G, H, L**).

#### Debus pumilus

Taxon classificationAnimaliaColeopteraCurculionidae

(Eggers, 1923)

Fig. 48C, D, J

Xyleborus pumilus Eggers, 1923: 209.
Debus pumilus (Eggers): Hulcr and Cognato 2010a: 15.
Xyleborus cylindricus Eggers, 1927b: 94. Synonymy: Hulcr and Cognato 2010a: 15.
Xyleborus neocylindricus Schedl, 1942a: 196. Synonymy: Beaver 2011: 284.
Ips kelantanensis Browne, 1955: 345. Synonymy: Beaver 1995a: 199 (as synonym of X. cylindricus)
Xyleborus ipidia Schedl, 1972a: Synonymy: Hulcr and Cognato 2010a: 15.
Xyleborus planodeclivis Browne, 1974: 70. Synonymy: Schedl 1980: 122 (as synonym of Xyleborus ipidia).

##### Type material.

***Holotype****Ips kelantanensis* (NHMUK). ***Lectotype****Xyleborus cylindricus* (NMNH). ***Paratype****Xyleborus ipidia* (NHMW). ***Holotype****Xyleborus neocylindricus* (NHMW). ***Holotype****Xyleborus planodeclivis* (NHMUK). ***Lectotype****Xyleborus pumilus* (NMNH).

##### New records.

China: S Yunnan, 28 km NW Jinghong, vic. An Ma Xi Zhan (NNNR), 22°12'N, 100°38'E, 700 m, forest, EKL, 30.x.2008, A. Weigel (RABC, 1). India: Arunachal Pradesh, Etalin vicinity, 28°36'56"N, 95°53'21"E, 700 m, 12–25.v.2012, L. Dembický (ZFMK, 5); as previous except: ex FIT (ZFMK, 3). Laos: Vientiane, Ban Van Eue, 30.xi.1965, native collector (BPBM, 2). Vietnam: Ninh Binh, Cuc Phuong N.P., 20.34932, 105.59669, 5.iii.2018, 431 m, A.I. Cognato, S.M. Smith, VN 113a, ex *Terminalia myriocarpa*; large tree-fall trunk 8 cm diameter (MSUC, 3).

##### Diagnosis.

2.5–3.2 mm long (mean = 2.78 mm; n = 5); 3.25–3.57× as long as wide. This species is distinguished by the elytral apex emarginate, never explanate, appearing flat and broad; declivital sulcus shallow; and small size.

##### Similar species.

*Debus adusticollis*, *D. detritus*.

##### Distribution.

Australia, China (Xizang, Yunnan*), Fiji, India (Andaman Is, Arunachal Pradesh*, Assam, West Bengal), Indonesia (Java, Maluku, Sumatra), Laos, East & West Malaysia, Myanmar, New Guinea, Philippines, Solomon Islands, Sri Lanka, Thailand, Vietnam.

##### Host plants.

Polyphagous. Browne (1961b) and Hulcr and Cognato (2013) suggest a strong preference for Moraceae, but the species has also been recorded from many other families.

#### Debus quadrispinus

Taxon classificationAnimaliaColeopteraCurculionidae

(Motschulsky, 1863) comb. nov.

Fig. 48E, F, K

Tomicus quadrispinus Motschulsky, 1863: 514.
Xyleborus quadrispinus (Motschulsky): Wood 1969: 120; Mandelshtam and Nikitsky 2010: 17.
Xyleborus fallax Eichhoff, 1878a: 392. syn. nov.
Xyleborus amphicranulus Eggers, 1923: 204. Synonymy: Schedl 1970b: 224.
Xyleborus fastigatus Schedl, 1935a: 402. Synonymy: Hulcr and Cognato 2010a: 15.

##### Type material.

***Holotype****Tomicus quadrispinus* (ZMMU). ***Syntype****Xyleborus fallax* (MIZ).

##### New records.

China: Jiangxi, Long Nan, 12.vii.2016, Lv-Jia, Lai, S-C., ex *Cyclobalanopsis glauca* (RABC, 1). S Yunnan, Xishuangbanna, 20 km NW Jinghong, vic. Man Dian (NNNR), 22°07.80'N, 100°40.05'E, 730 m, forest, 6.vi.2008, A. Weigel (NKME, 1); as previous except: 6.iv.2009, L. Meng (NKME, 1). Laos: Khamnouane, Phon Tiou, 10.vi.1965 (BPBM, 1). Vientiane, Ban Van Eue, 15.xii.1965, native collector (BPBM, 1). Philippines: Mindanao, Zamboanga, Kab 1.x.1932, H.C. Muzzall (NMNH, 1).

##### Diagnosis.

2.2–2.5 mm long (mean = 2.36 mm; n = 5); 3.67–3.83× as long as wide. This species is distinguished by the posterolateral extensions of elytra long, as long as width of apical emargination; declivity deeply excavated; small size; and typically bicolored appearance, with light brown pronotum and dark brown elytra.

##### Similar species.

*Debus emarginatus*, *D. shoreae*.

##### Distribution.

China* (Jiangxi, Yunnan), India (Assam), Indonesia (Enggano Is, Java, Maluku, Mentawai Is, Sulawesi, Sumatra), Laos*, East & West Malaysia, Myanmar, Nepal, New Guinea, Philippines*, Solomon Islands, Thailand, Vietnam.

##### Host plants.

Strongly polyphagous (e.g., Browne 1961b; Ohno 1990; Wood and Bright 1992).

##### Remarks.

Browne (1961b) provides further information on the habits of the species (as *X. fallax*). Photographs of the holotype of *Tomicus quadrispinus*Motschulsky (1863) at ZMMU were taken and shared with the authors by Alexander Petrov. The species was found to be conspecific to *Xyleborus fallax* Eichhoff (1878). *Tomicus quadrispinus* has priority and thus *Xyleborus fallax* is here placed in synonymy.

#### Debus shoreae

Taxon classificationAnimaliaColeopteraCurculionidae

(Stebbing, 1907)

Fig. 48G, H, L

Tomicus shoreae Stebbing, 1907: 39.
Xyleborus shoreae (Stebbing): Hulcr 2010: 109 (as synonym of Debus fallax (Eichhoff)).
Debus shoreae (Stebbing): Beaver et al. 2014: 44.
Tomicus assamensis Stebbing, 1909: 17. Synonymy: Beeson 1930: 259.

##### Type material.

***Holotype****Tomicus shoreae* (FRI).

##### New records.

China: Sichuan, Leibo, 800 m, 20.iv.1964, Fusheng Huang, ex fir (NMNH, 1). India: Arunachal Pradesh, Etalin vicinity, 28°36'56"N, 95°53'21"E, 700 m, 12–25.v.2012, L. Dembický (ZFMK, 3). Vietnam: Cao Bang, 22°36.3'N, 105°52.6'E, 1435–1601 m, 13–17.iv.2014, VN16, Cognato, Smith, Pham, ex FIT (MSUC, 4).

##### Diagnosis.

3.0–3.8 mm long (mean = 3.34 mm; n = 5); 2.92– 3.17× as long as wide. This species is distinguished by the posterolateral extensions of elytra short, less than the width of apical emargination and declivity shallowly excavated; and declivity impunctate except for a single row of punctures running from the upper margin to the inner margin of the second declivital spine and thence to the apical emargination.

This species is very similar to *D. emarginatus* and is distinguished by the punctation of the declivity.

##### Similar species.

*Debus emarginatus*, *D. quadrispinus*.

##### Distribution.

China (Guangxi, Sichuan*), India (Arunachal Pradesh*, Assam, Uttarakhand, Uttar Pradesh, West Bengal), Indonesia (Java, Sumatra), Laos, East Malaysia, Myanmar, Nepal, New Guinea, Thailand, Vietnam*.

##### Host plants.

Polyphagous, possibly with a preference for Dipterocarpaceae (Beaver et al. 2014).

##### Remarks.

Hulcr (2010) placed *D. shoreae* in synonymy with *D. quadrispinus* (as *D. fallax*) based on an illustration by Maiti and Saha (2004) as examination of type specimens was not possible. Maiti and Saha described the species as very morphologically similar to *D. quadrispinus* (as *D. fallax*). The illustration clearly shows the diagnostic declivital puncturation and other features as described above which are distinct from *D. quadrispinus*. Two specimens of *D. shoreae* collected from the type locality determined by C.F.C. Beeson (MSUC) were also examined and are clearly distinct from those of *D. quadrispinus*.

### *Diuncus* Hulcr & Cognato, 2009

#### Diuncus

Taxon classificationAnimaliaColeopteraCurculionidae

Hulcr & Cognato, 2009

Diuncus Hulcr & Cognato, 2009: 28.

##### Type species.

*Xyleborus papatrae* Schedl, 1972a; original designation.

##### Diagnosis.

Small to moderately sized (1.5–3.0 mm) stout species (1.33–2.78× as long as wide). *Diuncus* species are distinguished by the antennal club truncate, type 1, segment 1 corneous and dominant on both sides; pronotum stout, with 4–6 serrations on anterior margin; pronotum from lateral view rounded, robust (type 5), from dorsal view rounded (type 1), rarely conical and angulate (type 6); declivity flat and broad, margins broadened and distinctly carinate, declivital base often armed with one or two pairs of denticles; protibiae obliquely triangular, with 3–5 large denticles, denticles distinctly longer than wide; scutellum visible and flush with the elytra; mycangial tufts absent; and procoxae contiguous.

##### Similar genera.

*Ancipitis*, *Leptoxyleborus*, *Xylosandrus*.

##### Distribution.

Found in tropical Asia and Oceania, rare in Africa.

##### Gallery system.

The gallery systems in *Diuncus* vary depending on the species and the size of the breeding material. There may be an entrance tunnel leading to a terminal brood chamber in the longitudinal plane (*D. ciliatoformis*); the gallery may branch in three dimensions and either have very irregular brood chambers (*D. mucronatus*), or lack brood chambers (*D. javanus*); in small stems, there are longitudinal branches in the center of the stem (*D. haberkorni*).

##### Remarks.

*Diuncus* species are usually mycocleptic, making use of the ambrosia fungi of other ambrosia beetles, and lack mycangia (Hulcr and Cognato 2010b). However, some species also occur alone (Hulcr and Cognato 2009).

#### Key to *Diuncus* species (females only)

**Table:** 

1	Elytral summit unarmed (Fig. 50D)	**2**
–	Elytral summit armed by denticles (denticles may be present posterior to saddle-like impression) (Figs 50B, 51B)	**3**
2	Declivity densely covered by recumbent setae on both the striae and interstriae, setae short, as long as one interstrial width; declivital striae 1 moderately impressed; 1.65–1.95 mm	*** ciliatoformis ***
–	Declivity nearly glabrous, interstriae 2 and 4 with a sparse row of very long semi-erect hair-like setae, setae longer than the width of two interstriae; declivital striae 1 shallowly impressed 1.5–1.7 mm	*** justus ***
3	Elytral summit transversely impressed with a saddle-like depression (Fig. 50B)	**4**
–	Elytral summit convex, without a saddle-like depression (Fig. 49D)	**5**
4	Declivital interstrial setae thick, scale-like, in uniseriate rows; and striae glabrous	*** javanus ***
–	Declivital interstrial setae finer, almost hair-like, in two or three confused rows on interstriae 2–4; and striae setose, setae similar to those of interstriae	*** dossuarius ***
5	Declivity with uniseriate rows of small denticles along the entire length of interstriae 3, 5, 6; elytra and pronotum bicolored, darker on the apical areas	*** corpulentus ***
–	Declivity armed only at summit of interstriae 1 and 3; elytra and pronotum unicolored	**6**
6	Declivity flat; declivital summit armed by two pairs of minute sharp denticles	*** quadrispinulosus ***
–	Declivity appearing bisulcate; declivital summit armed by two pairs of large oblique denticles	**7**
7	Pronotum longer than wide; declivity appearing strongly bisulcate; interstriae 1 and 3–6 clearly convex giving the declivity a rugged appearance	*** mucronatus ***
–	Pronotum as long as wide; declivity appearing weakly bisulcate; interstriae 1 and 3–6 flat to weakly convex giving the declivity a finely sculptured appearance	**8**
8	Smaller, 1.5 mm; pronotum conical frontally and angulate (type 6) in dorsal view; pronotal summit anterior to midpoint	*** mucronatulus ***
–	Larger, 1.9–2.8 mm; pronotum rounded (type 1) in dorsal view; pronotal summit at midpoint	*** haberkorni ***

#### Diuncus ciliatoformis

Taxon classificationAnimaliaColeopteraCurculionidae

(Schedl, 1953) stat. res.

Fig. 49A, B, I

Xyleborus ciliatoformis Schedl, 1953d: 81.
Diuncus ciliatoformis (Schedl): Hulcr and Cognato 2009: 32.

##### Type material.

***Lectotype*** (NHMW).

##### New records.

China: Chongqing, Pengshui, 11.viii.2016, Tian-Shang (RABC, 1); Guizhou, Guiyang, East temple, viii.2015, Su, T-L. (RABC, 1).

##### Diagnosis.

1.65–1.95 mm long (mean = 1.78 mm; n = 5); 2.36–2.62× as long as wide. This species is distinguished by the minute size; unarmed declivity; moderately impressed declivital striae 1; declivity densely covered by recumbent setae on the striae and interstriae; and lateral margin of the protibiae armed with four denticles.

##### Similar species.

*Diuncus justus*.

##### Distribution.

China* (Chongqing, Guizhou), East & West Malaysia, New Guinea, Taiwan, Thailand.

##### Host plants.

Recorded from *Shorea*, *Vatica* (Dipterocarpaceae) and *Lithocarpus* (Fagaceae). Browne (1961b) suggests a preference for Dipterocarpaceae.

##### Remarks.

This species had previously been considered a synonym of *D. justus* by Hulcr and Cognato (2013). It is here removed from synonymy and reinstated as a distinct species. It is distinguished from *D. justus* by the moderately impressed declivital striae 1 and the declivity densely covered by recumbent setae on the striae and interstriae.

**Figure 49.**
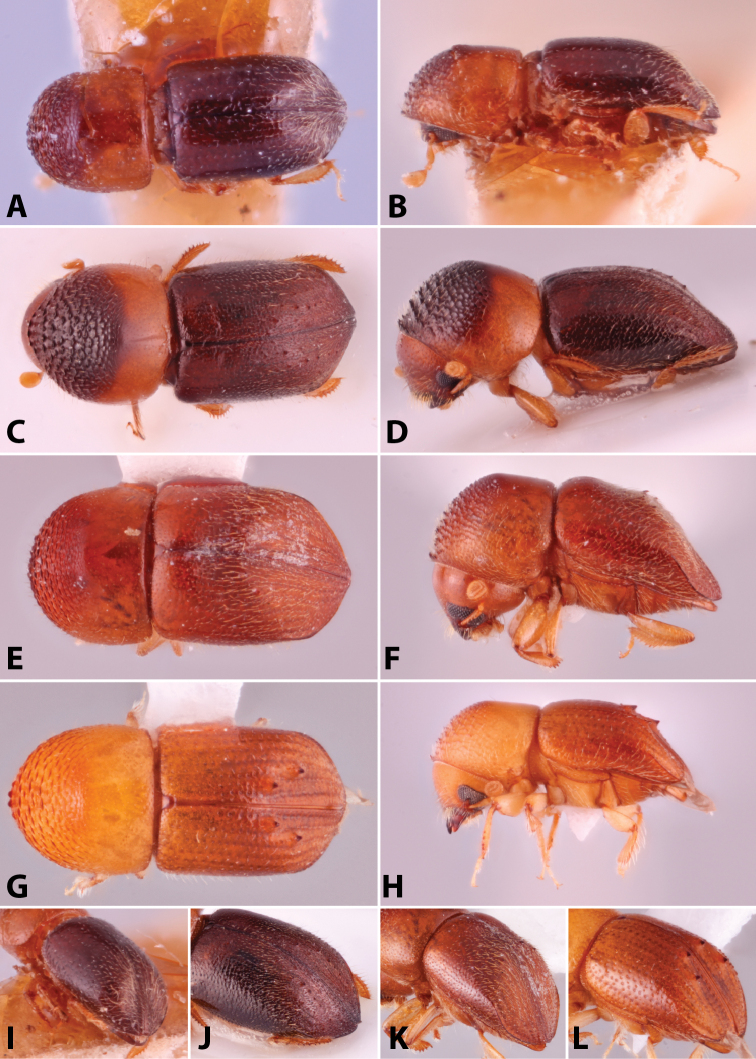
Dorsal, lateral and declivital view of *Diuncus ciliatoformis* lectotype, 1.65–1.95 mm (**A, B, I**), *D. corpulentus*, 1.6–3.2 mm (**C, D, J**), *D. dossuarius*, 2.6 mm (**E, F, K**), and *D. haberkorni*, 1.9–2.8 mm (**G, H, L**).

#### Diuncus corpulentus

Taxon classificationAnimaliaColeopteraCurculionidae

(Eggers, 1930)

Fig. 49C, D, J

Xyleborus corpulentus Eggers, 1930: 198.
Diuncus corpulentus (Eggers): Hulcr and Cognato 2009: 30.

##### Type material.

***Holotype*** (FRI).

##### New records.

China: Hainan, Wu-zhi-shan Town, 18.902N, 109.663E, 703 m, 2.xii.2016, Tian-Shang & Lv-Jia (RABC, 1). S-Yunnan, Xishuangbanna, 37 km NW Jinghong, vic. Guo Men Shan, 22°14.48'N, 100°36.22'E, 1080 m, 10.x.2008, UWP MF, L. Meng (RABC, 1); as previous except: 23 km NW Jinghong, vic. Na Ban (NNNR), 22°09.49'N, 100°39.92'E, 730 m, second[ary] for[est], 6.vi.2008, GS, A. Weigel (RABC, 1). Xishuangbanna, 23 km NW Jinghong, vic. Na Ban village (NNNR), 22°10'N, 100°39'E, 700–1000 m, v–vii.2009, L. Meng (RABC, 1). India: Arunachal Pradesh, Hunli, 28°19'32"N, 95°57'31"E, 1300 ±100 m, 26.v–1.vi.2012, L. Dembický, ex FIT (ZFMK, 1). Assam-Arunachal Pradesh border, Bhalukpong, 27°00'48"N, 92°39'08"E, 150 m, 1–8.v.2012, L. Dembický, ex FIT (ZFMK, 2). Meghalaya, 3 km E Tura, 25°30'N, 90°14'E, 1150 m, 4.v.1999, Dombický & Pacholátko (RABC, 1). Laos: Vientiane, Ban Van Eue, 15–31.v.1965, native collector (BPBM, 1). Taiwan: Nantou, Sun Moon Lake, 8.vii.2016, C.-S. Lin (MSUC, 1). Vietnam: Lao Cai, Hoang Lien N.P., 22.35, 103.77, 1500 m, 17.v.2019, VN152, S.M. Smith, A.I. Cognato, ex branch; 1–3 cm (MSUC, 2). Ninh Binh, Cuc Phuong N.P., 20.33296, 105.61259, 7.iii.2018, 279 m, A.I. Cognato, S.M. Smith, VN 147, ex 3–4 cm diameter branch from tree fall; red latex (MSUC, 1).

##### Diagnosis.

1.6–3.2 mm long (mean = 2.66 mm; n = 5); 1.33–2.31× as long as wide. This species is distinguished by the elytral disc convex; declivital summit armed by three denticles along interstriae 2; declivital interstriae 3, 5, and 6 bearing a uniseriate row of denticles along its length; interstrial setae minute, strongly confused, recumbent, as long as length between setae; and bicolored elytra and pronotum that are darker at the apical areas.

##### Similar species.

*Diuncus dossuarius*, *D. javanus*.

##### Distribution.

China (Hainan, Xizang, Yunnan*), India (Andaman Is, Arunachal Pradesh*, Assam, Meghalaya*, West Bengal), Laos, Nepal, Taiwan, Thailand, Vietnam*.

##### Host plants.

Polyphagous (Beeson 1930; Maiti and Saha 2004). Hulcr and Cognato (2009) found it in association with *Hadrodemius globus* in Thailand.

#### Diuncus dossuarius

Taxon classificationAnimaliaColeopteraCurculionidae

(Eggers, 1923)

Fig. 49E, F, K

Xyleborus dossuarius Eggers, 1923: 187.
Diuncus dossuarius (Eggers): Hulcr and Cognato 2009: 30.

##### Type material.

***Paratype*** (NHMUK).

##### Diagnosis.

2.6 mm long (mean = 2.6 mm; n = 5); 2.0–2.17× as long as wide. This species is distinguished by the elytral summit transversely impressed with a saddle-like depression; declivital base armed by two pairs of denticles, one pair on interstriae 2 and the other on interstriae 3; bicolored elytra and pronotum that are darker on the apical areas; interstrial setae recumbent, finer, almost hair-like, in two or three confused rows on interstriae 2–4; and striae setose, setae similar to those of interstriae.

##### Similar species.

*Diuncus corpulentus*, *D. javanus*.

##### Distribution.

Brunei, Philippines, Vietnam.

##### Host plants.

Recorded from *Swietenia*, *Toona* (Meliaceae), and *Ficus* (Moraceae) (Schedl 1966a; Nobuchi 1979).

#### Diuncus haberkorni

Taxon classificationAnimaliaColeopteraCurculionidae

(Eggers, 1920)

Fig. 49G, H, L

Xyleborus haberkorni Eggers, 1920: 43.
Diuncus haberkorni (Eggers): Hulcr and Cognato 2009: 31.
Xyleborus approximatus Schedl, 1951a: 77. Synonymy: Hulcr and Cognato 2013: 80.
Xyleborus taichuensis Schedl, 1952b: 64. Synonymy: Beaver and Liu 2010: 26.
Xyleborus potens Schedl, 1964a: 298. Synonymy: Schedl 1975e: 35 (as synonym of X. approximatus).

##### Type material.

***Lectotype****Xyleborus haberkorni* (NMNH). ***Paratype****Xyleborus taichuensis* (NHMW).

##### New records.

China: Fujian, Fuan, Shuyang, 2.x.2018, A. Ernstsons, ex EtOH trap (MSUC, 1). Guangdong, Shenzhen, 11.iv.2018, Y. Li (UFFE, 1). Guangxi, Shiwandashan, 25.iii.2018, Y. Li (UFFE, 1). Hong Kong, Kadoorie Farm, vi.2017, J. Skelton (UFFE, 1). Jiangxi, Gan Zhou, 5.vii.2015, Lv-Jia (RABC, 1); as previous except: Nan Chang, 22.vi.2016, ex *Cinnamomum camphora* (RABC, 1); as previous except: 18.vi.2016, ex *Ligustrum lucidum* (RABC, 1). Yunnan, Xishuangbanna, 23 km NW Jinghong, vic. Na Ban village (NNNR), 22°10'N, 100°39'E, 700–1000 m, v–vii. 2009, L. Meng (NKME, 1); as previous except: 25 km NW Jinghong, vic. Zhang Zhi Chang (NNNR), 22°11.06'N, 100°39.05'E, 780 m, rubber plantation, EKL, 15.vi.2008, A. Weigel (NKME, 1); as previous except: 28 km NW Jinghong, vic. An Ma Xi Zhan (NNNR), 22°12'N, 100°38'E; 700 m, forest, EKL, 30.x.2008 (NKME, 2). India: Assam-Arunachal Pradesh border, Bhalukpong, 27°00'48"N, 92°39'08"E, 150 m, 1–8.v.2012, L. Dembický, ex FIT (ZFMK, 1). Laos: Vientiane, ii.1965, J.L. Gressitt, ex light trap (BPBM, 1). Vientiane, Ban Van Eue, 30.iii.1967, native collector (BPBM, 1). Vietnam: Dong Nai, Cat Tien N.P., 11.40817, 107.38098, 134 m, 20–22.ii.2017, VN81, A.I. Cognato, T.A. Hoang, ex FIT (MSUC, 14). Ninh Binh, Doi Vac, Cuc Phuong, 10–16.ix.2013, J.B. Heppner (FSCA, 1). Yen Bai, Mau A, 21.88226, 104.68040, 15.iv.2015, R.J. Rabaglia, ex funnel trap (RJRC, 1).

##### Diagnosis.

1.9–2.8 mm long (mean = 2.28 mm; n = 5); 2.11–2.38× as long as wide. This species is distinguished by the elytral summit armed by two pairs of large denticles, one pair on interstriae 2 and the other on interstriae 3; pronotum approximately as long as wide, summit at midpoint, basal 1/2 punctate; declivity appearing weakly bisulcate; and interstriae 1 and 3–6 flat to weakly convex giving the declivity a finely sculptured appearance.

##### Similar species.

*Diuncus mucronatus*, *D. mucronatulus*, *D. quadrispinulosus*.

##### Distribution.

Bangladesh, China (Fujian*, Guangdong*, Guangxi*, Hainan, Hong Kong*, Jiangxi*, Yunnan*), India (Andaman Is, Assam, Arunachal Pradesh*, Tamil Nadu, Uttarakhand, West Bengal), Indonesia (Java), Japan (Ryukyu Is), East & West Malaysia, New Guinea, South Korea, Sri Lanka, Taiwan, Thailand, Vietnam. Imported to Africa (South Africa, Tanzania).

##### Host plants.

Polyphagous (Beeson 1930; Browne 1961b). The species is sometimes associated with other xyleborines (Beaver and Browne 1979; Hulcr and Cognato 2010b), but may also occur alone (Hulcr and Cognato 2009).

#### Diuncus javanus

Taxon classificationAnimaliaColeopteraCurculionidae

(Eggers, 1923)

Fig. 50A, B, I

Xyleborus javanus Eggers, 1923: 188.
Diuncus javanus (Eggers): Hulcr and Cognato 2009: 32.
Xyleborus perdix Schedl, 1939a: 351. Synonymy: Schedl 1960b: 109.

##### Type material.

***Lectotype****Xyleborus javanus* (NMNH).

##### Diagnosis.

2.5–2.7 mm long (mean = 2.62 mm; n = 5); 2.08–2.25× as long as wide. This species is distinguished by the elytral summit transversely impressed with a saddle-like depression; declivital base armed by two pairs of denticles, one pair on interstriae 2 and the other on interstriae 3; bicolored elytra and pronotum that are darker on the apical areas; declivital interstrial setae recumbent, thick, scale-like, in uniseriate rows; and striae glabrous.

##### Similar species.

*Diuncus corpulentus*, *D. dossuarius*.

##### Distribution.

Brunei, Indonesia (Java, Sumatra, Sulawesi), East & West Malaysia, Philippines, Thailand.

##### Host plants.

Polyphagous (Kalshoven 1959b; Browne 1961a).

**Figure 50.**
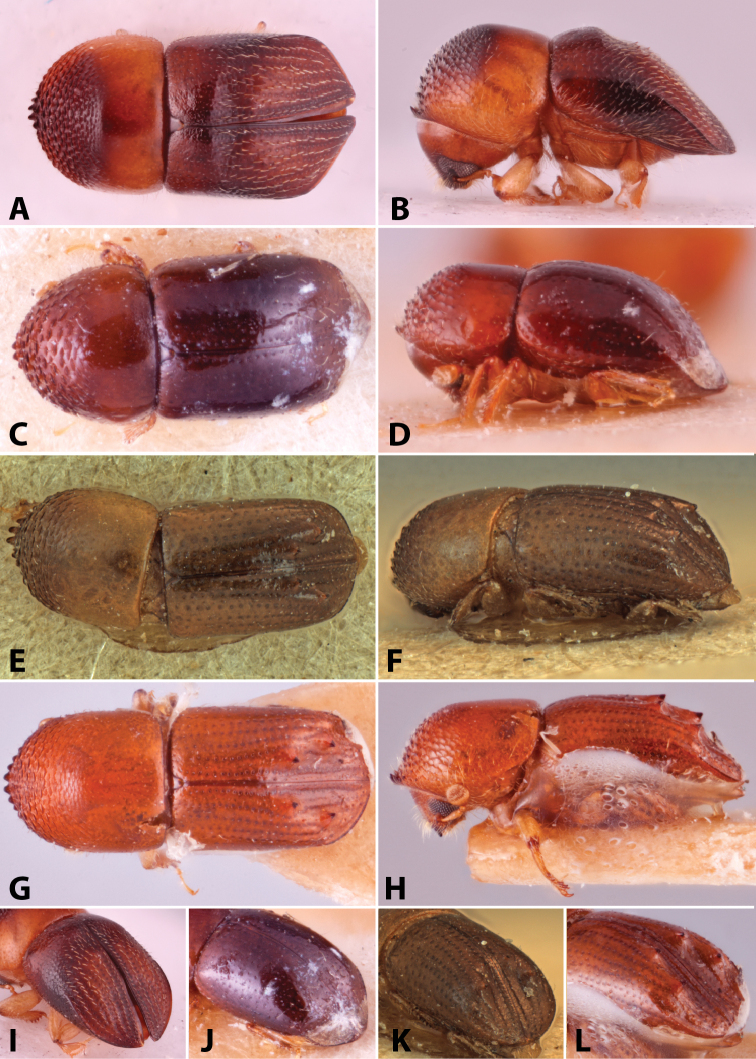
Dorsal, lateral and declivital view of *Diuncus javanus*, 2.5–2.7 mm (**A, B, I**), *D. justus*, 1.5–1.7 mm (**C, D, J**), *D. mucronatulus* holotype, 1.5 mm (**E, F, K**), and *D. mucronatus*, 2.0–2.5 mm (**G, H, L**).

#### Diuncus justus

Taxon classificationAnimaliaColeopteraCurculionidae

(Schedl, 1931)

Fig. 50C, D, J

Xyleborus justus Schedl, 1931: 339.
Diuncus justus (Schedl): Hulcr and Cognato 2009: 32.
Xyleborus marginicollis Schedl, 1936c: 64. Synonymy: Hulcr 2010: 107.
Xyleborus ciliatus Eggers, 1940: 141. Synonymy: Hulcr and Cognato 2013: 81.
Xyleborus apiculatus Schedl, 1942a: 190. Synonymy: Hulcr and Cognato 2013: 81.

##### Type material.

***Holotype****Xyleborus justus* (NMNH). ***Holotype****Xyleborus marginicollis* (NHMW).

##### New records.

Vietnam: Cao Bang, 22°34.5'N, 105°52.4'E, ~ 1080 m, 14.iv.2014, VN28, Cognato, Smith, Pham, ex *Cunninghamia* branches (MSUC, 1).

##### Diagnosis.

1.5–1.7 mm long (mean = 1.65 mm; n = 5); 2.36–2.5× as long as wide. This species is distinguished by the minute size; unarmed declivity; declivital striae 1 shallowly impressed; nearly glabrous appearance; and lateral margin of the protibiae armed with four denticles.

##### Similar species.

*Diuncus ciliatoformis*.

##### Distribution.

Australia, China (Fujian), Indonesia (Java), East & West Malaysia, New Guinea, Vietnam*.

##### Host plants.

This species has only been recorded from *Cunninghamia* (Cupressaceae).

##### Remarks.

The synonymy of *Xyleborus apiculatus*, *X. ciliatus*, and *X. marginicollis* with *Diuncus justus* needs to be reassessed using information from DNA as well as morphology.

#### Diuncus mucronatulus

Taxon classificationAnimaliaColeopteraCurculionidae

(Eggers, 1930)

Fig. 50E, F, K

Xyleborus mucronatulus Eggers, 1930: 199.
Diuncus mucronatulus (Eggers): Hulcr and Cognato 2009: 33.

##### Type material.

***Holotype*** (FRI).

##### Diagnosis.

1.5 mm long; 2.37× as long as wide. This species is distinguished by its minute size; pronotum conical frontally and angulate (type 6) in dorsal view; pronotal summit at anterior 3/8, basal 5/8 punctate; elytral summit armed by two pairs of large denticles, one pair on interstriae 2, the other on interstriae 3; pronotum as long as wide; declivity appearing weakly bisulcate; and interstriae 1 and 3–6 flat to weakly convex giving the declivity a finely sculptured appearance.

##### Similar species.

*Diuncus haberkorni*, *D. mucronatus*, *D. quadrispinulosus*.

##### Distribution.

India (West Bengal). The inclusion of Indonesia (‘Borneo’, Java) , Malaysia and Thailand in the distribution by Maiti and Saha (2004) is in error.

##### Host plants.

Recorded only from *Mesua* (Calophyllaceae) (Beeson 1930).

##### Remarks.

The species was found associated with *Xylosandrus mesuae* (Eggers) (Beeson 1930).

#### Diuncus mucronatus

Taxon classificationAnimaliaColeopteraCurculionidae

(Eggers, 1923)

Fig. 50G, H, L

Xyleborus mucronatus Eggers, 1923: 191.
Diuncus mucronatus (Eggers): Hulcr and Cognato 2009: 34.

##### Type material.

The holotype was destroyed in the bombing of UHZM in World War II (Wood and Bright 1992).

##### New records.

China: Guizhou, Guiyang, Huaxi, 25.x.2015, Y. Li, ex trap baited with ipsenol + EtOH (MSUC, 1). Hong Kong, Tai Po Kau, vi.2017, J. Skelton (MSUC, 4). Jiangsu, Nanjing, Laoshan National Park, Bacai Road, 32.09156N, 118.583701E, 15.viii.2017, Cognato, Li, Gao, ex *Populus* (MSUC, 2). Vietnam: Cao Bang, 22°34.118'N, 105°52.537'E, 1048 m, 12.iv.2014, VN13, Cognato, Smith, Pham, ex large felled *Pinus* sp. (MSUC, 1).

##### Diagnosis.

2.0–2.5 mm long (mean = 2.26 mm; n = 5); 2.33–2.78× as long as wide. This species is distinguished by the elytral summit armed by two pairs of large denticles, one pair on interstriae 2 and the other on interstriae 3; pronotum longer than wide; declivity appearing strongly bisulcate; declivital interstriae 1 and 3–6 clearly convex giving the declivity a rugged appearance.

##### Similar species.

*Diuncus haberkorni*, *D. mucronatulus*, *D. quadrispinulosus*.

##### Distribution.

China* (Guizhou*, Hong Kong*, Jiangsu*), Indonesia (Java), Japan, East & West Malaysia, New Guinea, Philippines, Thailand, Vietnam*.

##### Host plants.

Polyphagous (Browne 1961b).

#### Diuncus quadrispinosulus

Taxon classificationAnimaliaColeopteraCurculionidae

(Eggers, 1923)

Fig. 51A, B, C

Xyleborus quadrispinosulus Eggers, 1923: 189.
Diuncus quadrispinosulus (Eggers): Hulcr and Cognato 2009: 34.
Xyleborus parvispinosus palembangensis Schedl, 1939b: 43. Synonymy: Schedl 1958c: 147.
Xyleborus parvispinosus Schedl, 1951a: 78. Synonymy: Schedl 1958c: 147.

##### Type material.

***Holotype****Xyleborus quadrispinosulus* (MCG).

##### New records.

Thailand: Narathiwat, Hala-Bala Wildlife Sanct., 5°47'44"N, 101°50'07"E, lowland TRF [tropical rain forest], 1.ii.2015, W. Sittichaya (RABC, 1). Vietnam: N [Tuyen Quang], 160 km NNW Hanoi, NE env. of Na Hang, 150–200 m, 3–13.vi.1996, A. Napolov & I. Roma (RABC, 2).

##### Diagnosis.

1.8–1.9 mm long (mean = 1.82 mm; n = 5); 2.25–2.57× as long as wide. This species is distinguished by the elytral summit armed by two pairs of minute denticles, one pair on interstriae 2 and the other on interstriae 3.

##### Similar species.

*Diuncus haberkorni*, *D. mucronatus*, *D. mucronatulus*.

##### Distribution.

Indonesia (Java, Sumatra), East & West Malaysia, Myanmar, New Guinea, Thailand, Vietnam*.

##### Host plants.

Polyphagous (Browne 1961b).

**Figure 51.**
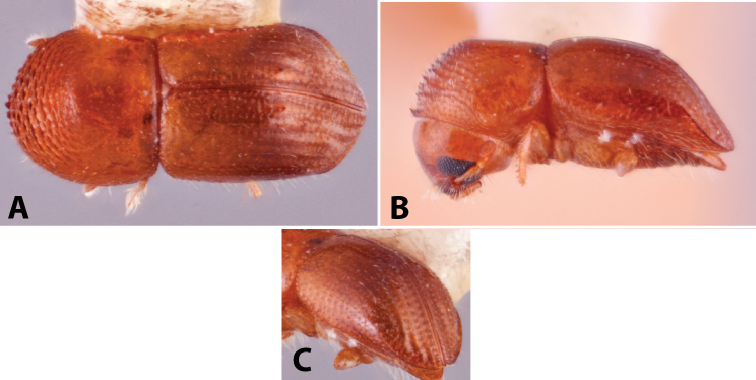
Dorsal, lateral and declivital view of *Diuncus quadrispinulosus*, 1.8–1.9 mm (**A–C**).

#### Dryoxylon

Taxon classificationAnimaliaColeopteraCurculionidae

Bright & Rabaglia, 1999

Dryoxylon Bright & Rabaglia, 1999: 333.

##### Type species.

*Xyleborus onoharaensis* Murayama, 1934; original designation.

##### Diagnosis.

2.2–2.4 mm and elongate (3.14–3.43× as long as wide). *Dryoxylon* is most easily distinguished by the anterior margin of pronotum in lateral view evenly arched, summit not elevated or evident; anterior margin of pronotum weakly emarginated at middle; declivity distinctly moderately sulcate; declivital face and lateral margins unarmed; submentum not impressed; comparatively few socketed denticles on the outer margin of the pro- (five), meso- (six) and metatibiae (five); scutellum flat, flush with elytra; procoxae narrowly separated; mycangial tufts absent; and elytra unarmed.

##### Similar genera.

*Dryoxylon* is superficially similar to *Cyclorhipidion* which also has elongate species with a setose declivity but is distinguished by the unique pronotum described above. *Dryoxylon* may also be confused with Dryocoetini because of the reduced number of socketed denticles on the pro- and metatibiae (five).

##### Distribution.

Known only from China, Japan and South Korea. Introduced and established in USA.

##### Gallery system.

Unknown. The biology of the only species in the genus, *D. onoharaense* has been investigated in the USA. Bright and Rabaglia (1999) reported *D. onoharaense* in the xylem associated with other xyleborines, but galleries solely containing this species were not found. Bateman et al. (2015) examined the fungal associates of *D. onoharaense* in Florida. The authors were unable to locate a mycangium or isolate fungi from the species. This suggests that the species is not engaged in typical fungus farming but may be entering established galleries of other ambrosia beetles rather than establishing their own, similar to the Neotropical genus *Sampsonius*. The species could also be mycocleptic similar to *Diuncus* which steal fungi from nearby galleries (Bateman et al. 2015; Hulcr and Cognato 2010b).

##### Remarks.

*Dryoxylon* was originally placed in the Dryocoetini. Molecular data clearly indicates that this genus belongs in the Xyleborini (Jordal et al. 2000; Jordal 2002; Gohli et al. 2017) into which it was transferred by Alonso-Zarazaga and Lyal (2009).

#### Dryoxylon onoharaense

Taxon classificationAnimaliaColeopteraCurculionidae

(Murayama, 1934)

Fig. 52

Xyleborus onoharaensis Murayama, 1934: 293.
Dryoxylon onoharaensum (Murayama): Bright and Rabaglia 1999: 333.
Dryoxylon onoharaensis (Murayama): Bright and Skidmore 2002: 95.
Dryoxylon onoharaense (Murayama): Alonso-Zarazaga and Lyal 2009: 100.

##### Type material.

***Holotype*** (NMNH).

##### New records.

China: Guizhou, Guiyang, vi.2015, Y. Li, ex ethanol trap (UFFE, 1); as previous except: ix.2015 (UFFE, 1). Sichuan, Leibo, 800 m, 20.iv.1964, F. Huang, ex Cupressaceae 119 (NMNH, 1).

##### Diagnosis.

2.2–2.4 mm long (mean = 2.36 mm; n = 5); 3.14–3.43× as long as wide. As described for the genus. This species is most easily distinguished by the anterior margin of pronotum in lateral view evenly arched, summit not elevated or evident; anterior margin of pronotum weakly emarginated at middle; declivity distinctly moderately deeply sulcate; declivital face and lateral margins unarmed; and comparatively few socketed denticles on the outer margin of the pro- (five), meso- (six) and metatibiae (five).

##### Similar species.

Small *Cyclorhipidion* spp.

##### Distribution.

China* (Guizhou, Sichuan), Japan, South Korea. Introduced and established in USA (Rabaglia and Bright 1999; Gomez et al. 2018a).

##### Host plants.

*Abies* (Pinaceae), *Acer* (Sapindaceae) (Bright and Rabaglia 1999), *Liriodendron tulipifera* (Magnoliaceae) (Atkinson 2018), *Populus* (Salicaceae) (Coyle et al. 2005), *Quercus* (Fagaceae) (Murayama 1934).

##### Remarks.

This species has been collected from both coniferous and angiosperm hosts.

**Figure 52.**
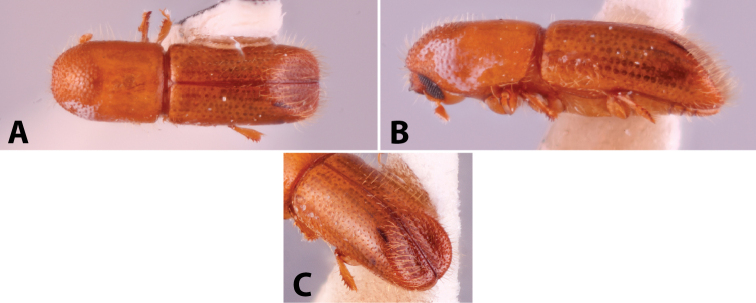
Dorsal, lateral and declivital view of *Dryoxylon onoharaense*, 2.2–2.4 mm (**A–C**).

### *Eccoptopterus* Motschulsky, 1863

#### Eccoptopterus

Taxon classificationAnimaliaColeopteraCurculionidae

Motschulsky, 1863

Eccoptopterus Motschulsky, 1863: 515.
Platydactylus Eichhoff, 1886: 25. Preoccupied by Goldfuss 1820.
Eurydactylus Hagedorn, 1909: 733. (new name for Platydactylus Eichhoff, 1866 nec Goldfuss 1820). Synonymy: Hagedorn 1910b: 110.

##### Type species.

*Eccoptopterus sexspinosus* Motschulsky, 1863 = *Scolytus spinosus* Olivier, 1800; monotypy.

##### Diagnosis.

2.5–4.2 mm and stout (2.06–2.3× as long as wide). *Eccoptopterus* is distinguished by the robust pronotum which is almost as large or larger than abdomen; pronotal base bearing a dense tuft of setae; pronotal disc asperate; elytra excavated with denticles around the margins and by the metatibiae conspicuously enlarged and flattened. In addition, the scutellum is flush with elytra and flat, and procoxae are contiguous.

##### Similar genera.

*Eccoptopterus* is morphologically very distinctive and is not similar to other genera.

##### Distribution.

Throughout the tropical regions of Africa and Asia to New Guinea, Australia, the Solomon Islands and Samoa.

##### Gallery system.

The radial entrance gallery leads to several branches in various planes, not penetrating more than 3–4 cm. In small diameter stems, the branches may be longitudinal. Enlarged brood chambers are absent.

#### Key to *Eccoptopterus* species (females only)

**Table:** 

1	Declivity bearing more than three spines on each elytral margin; declivital armature consisting of two large spines closest to suture on declivital summit and many smaller, uniform-sized denticles on declivital margin	*** limbus ***
–	Declivity bearing three spines on each elytral margin; largest spine near the declivital summit	*** spinosus ***

#### Eccoptopterus limbus

Taxon classificationAnimaliaColeopteraCurculionidae

Sampson, 1911

Fig. 53A, B, E

Eccoptopterus limbus Sampson, 1911: 381.
Xyleborus auratus Eggers, 1923: 193. Synonymy: Wood and Bright 1992: 821.
Xyleborus squamulosus Eggers, 1923: 193. Synonymy: Eggers 1927a: 407.
Xyleborus squamulosus duplicatus Eggers, 1923: 193. Synonymy: Browne 1955: 351; Wood 1989: 172.

##### Type material.

***Holotype****Eccoptopterus limbus* (NHMUK). ***Lectotype****Xyleborus auratus* (NMNH). ***Lectotype****Xyleborus squamulosus* (NMNH). ***Lectotype****Xyleborus squamulosus duplicatus* (NMNH).

##### Diagnosis.

3.5–4.2 mm long (mean = 3.73 mm; n = 5); 2.1–2.3× as long as wide. This species is distinguished by the presence of more than three spines on each elytral margin, declivital armature consists of two large spines closest to suture on declivital summit and many smaller, uniformly sized denticles on declivital margin.

##### Similar species.

*Eccoptopterus spinosus*.

##### Distribution.

China (Yunnan), Indonesia (Java, Sumatra, Sunda Is), East & West Malaysia, New Guinea, Thailand.

##### Host plants.

Polyphagous (Browne 1961b).

##### Remarks.

Elytral vestiture of this species is quite variable. In Asian specimens the declivity is covered with dense flattened scales while specimens from Papua New Guinea are covered by long setae. The shape, density and color of the scales are quite variable (Hulcr and Cognato 2013).

**Figure 53.**
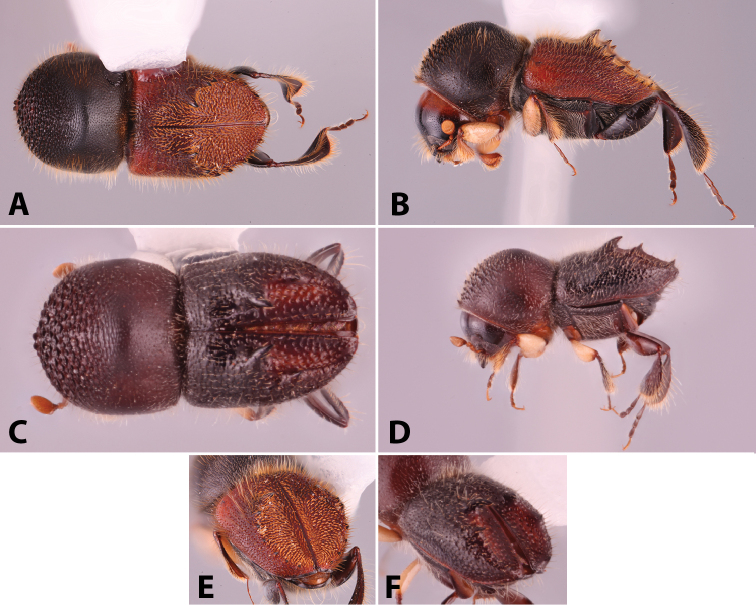
Dorsal, lateral and declivital view of *Eccoptopterus limbus*, 3.5–4.2 mm (**A, B, E**), and *E. spinosus*, 2.5–3.7 mm (**C, D, F**).

#### Eccoptopterus spinosus

Taxon classificationAnimaliaColeopteraCurculionidae

(Olivier, 1800)

Fig. 53C, D, F

Scolytus spinosus Olivier, 1800: 9.
Eccoptopterus spinosus (Olivier): Schedl 1962a: 201.
Eccoptopterus sexspinosus Motschulsky, 1863: 515. Synonymy: Schedl 1962a: 201.
Xyleborus abnormis Eichhoff, 1869: 282. Synonymy: Eichhoff 1876b: 379.
Platydactylus gracilipes Eichhoff, 1886: 25. Synonymy: Hulcr and Cognato 2013: 87.
Xyleborus sexspinosus multispinosus Hagedorn, 1908: 377. Synonymy: Schedl 1963a: 92.
Xyleborus collaris Eggers, 1923: 194. Schedl 1970b: 225 [as synonym of E. gracilipes]
Eccoptopterus sagittarius Schedl, 1939b: 41. Synonymy: Hulcr and Cognato 2013: 87.
Eccoptopterus sexspinosus pluridentatus Schedl, 1942c: 49. Synonymy: Kalshoven 1959a: 96. [as synonym of multispinosus]
Xyleborus eccoptopterus Schedl, 1951b: 154. Synonymy: Beaver 1987: 67.

##### Type material.

***Lectotype****Xyleborus collaris* (NMNH).

##### New records.

China: S Yunnan, Xishuangbanna, 23 km NW Jinghong, vic. Na Ban village (NNNR), 22°10'N, 100°39'E, 700–1000 m, v–vii.2009, L. Meng (RABC, 2); as previous except: 25 km NW Jinghong, vic. Zhang Zhi Chang (NNNR), 22°11.06'N, 100°39.05'E, 780 m, rubber plantation, EKL, 12.v.2008, A. Weigel (RABC, 1); as previous except: 6.iv.2009, L. Meng (NKME, 3); as previous except: 37 km NW Jinghong, vic. Guo Men Shan, 22°14.48'N, 100°36.22'E; 1080 m, forest, 6.iv.2009, L. Meng (NKME, 5). Laos: Attapeu, Annam Highlands Mountains, Dong Amphan NBCA, Nong Fa (crater lake) env., 15°05.9'N, 107°25.6'E, c.1160 m, 30.iv–6.v.2010, J. Hájek (MNHP, 2). [Bolikhamxai], Nam Kading, nr. Pak Kading, 21.iv.1965, J. L. Gressitt (BPBM, 1). Vietnam: Dong Nai, Cat Tien N.P., 11.42232, 107.42834, 128 m, 19.ii.2017, VN74, A.I. Cognato, T.A. Hoang, ex porch light (MSUC, 47). Tonkin, Hoa-Binh, 1934, A. De Cooman (MNHN, 5).

##### Diagnosis.

2.5–3.7 mm long (mean = 2.9 mm; n = 5); 2.06–2.27× as long as wide. This species is distinguished by the presence of three spines on each elytral margin, with the largest spine near the declivital summit.

##### Similar species.

*Eccoptopterus limbus*.

##### Distribution.

Throughout the tropical regions of Africa and Asia to New Guinea, Australia, the Solomon Islands and Samoa. Recorded in the study region from Cambodia, China* (Yunnan), India (Andaman Is, Assam, Maharashtra, Sikkim, Tamil Nadu, West Bengal), Laos*, Myanmar, Taiwan, Thailand, Vietnam.

##### Host plants.

Polyphagous (Browne 1961a; Schedl 1963a).

##### Remarks.

Following Bousquet (2018), we date the original description of the species to 1800 rather than the usually cited 1795.

*Eccoptopterus spinosus* is a morphologically variable species and represents a species complex that will require a more detailed investigation to address. COI sequences from specimens collected from Ghana, Papua New Guinea, Indonesia (Java), Taiwan, Vietnam differed from 12–18% between sites and CAD varied by 2–7% (Cognato et al. 2020b).

### *Euwallacea* Hopkins, 1915

#### Euwallacea

Taxon classificationAnimaliaColeopteraCurculionidae

Hopkins, 1915

Euwallacea Hopkins, 1915a: 54.
Wallacellus Hulcr & Cognato, 2010a: 27. Synonymy: Storer et al. 2015.

##### Type species.

*Xyleborus wallacei* Blandford, 1896b; original designation.

##### Diagnosis.

1.8–5.7 mm, 2.08–3.6× as long as wide. *Euwallacea* is distinguished by a combination of homoplastic characters which include the pronotum typically tall with inflated anterolateral corners, appearing subquadrate to quadrate in dorsal profile (types 3, 4, 8), less commonly with rounded anterior margin (types 2, 4, 7); anterior margin of pronotum unarmed; pronotal disc alutaceous; declivital posterolateral margin with prominent costa or carina; elytral discal interstrial punctures seriate; declivity typically with very sparse setae; and antennal club truncate (type 2) or flattened (type 3), circular or taller than wide. In addition, the scutellum is flush with elytra and flat, mycangial tufts are absent, lateral margin of pronotum obliquely costate, and procoxae are contiguous.

##### Similar genera.

*Fortiborus*, *Planiculus*, *Xylosandrus*.

##### Distribution.

Found throughout tropical South Asia and Oceania, rare in temperate East Asia. Six species, including three in the *Euwallacea fornicatus* species complex, have been introduced to North America (Gomez et al. 2018a, 2018b).

##### Gallery system.

This consists of branched tunnels, either in one horizontal plane or extending into three dimensions and penetrating deeply into the wood. Brood chambers are absent. In small diameter stems the galleries may be longitudinal.

##### Remarks.

*Euwallacea* species are in need of further taxonomic/phylogenetic investigation given evidence of several non-monophyletic species (Cognato et al. 2020b).

#### Key to *Euwallacea* species (females only)

**Table:** 

1	Elytra as long as wide	*** aplanatus ***
–	Elytra longer than wide	**2**
2	Declivital interstriae 1 laterally broadened from declivital summit to apical 1/3 then narrowed to apex, with a large tubercle on apical 1/3	*** similis ***
–	Declivital interstriae 1 uniform in width	**3**
3	Protibiae obliquely or distinctly triangular	**4**
–	Protibiae semi-circular with evenly rounded outer edge	**10**
4	Anterior margin of pronotum rounded, elongate, type 7 in dorsal view (Fig. 56C)	**5**
–	Anterior margin of pronotum subquadrate or quadrate, types 3 or 4 in dorsal view (Fig. 55E)	**7**
5	Very elongate, 3.6× as long as wide	*** luctuosus ***
–	Less elongate, 2.5–3.0× as long as wide	**6**
6	Posterolateral margin of declivity acutely carinate; declivital face sulcate armed only by one transverse row of four large granules at declivital summit, one on interstriae 1 and 3; larger, 2.5–2.75 mm	*** semiermis ***
–	Posterolateral margin of declivity costate; declivital face convex, without a transverse row of granules at declivital summit, granules on declivital face; smaller, 2.4 mm	***subalpinus* sp. nov.**
7	Protibiae with 7–9 socketed denticles on outer margins; very large, 4.6–5.7 mm	*** gravelyi ***
–	Protibiae with 4–6 socketed denticles on outer margins; moderate to large, 2.8–4.6 mm	**8**
8	Strial punctures much larger on declivity than on disc; declivity typically opalescent	*** andamanensis ***
–	Strial punctures on declivity and disc approximately equal in size; declivity strongly shiny	**9**
9	Declivity gradual, occupying apical ~40% of elytra; larger, 3.9–4.6 mm and less elongate, 2.54–2.79× as long as wide	*** destruens ***
–	Declivity very steep, occupying apical ~20% of elytra; smaller, 3.4–3.9 mm and more elongate, 2.77–2.83× as long as wide	*** sibsagaricus ***
10	Anterior margin of pronotum rounded, basic, type 2 in dorsal view (Fig. 57C)	**11**
–	Anterior margin of pronotum subquadrate or quadrate, types 3 or 4 in dorsal view (Fig. 59E)	**15**
11	Posterolateral margin of declivity granulate and carinate or costate	**12**
–	Posterolateral margin of declivity carinate or costate and never granulate	**19**
12	Posterolateral margin of declivity costate; smaller, 1.8–1.9 mm	*** minutus ***
–	Posterolateral margin of declivity carinate; larger, 2.4–4.2 mm	**9**
13	Elytral bases oblique, unarmed; posterolateral margin of declivity acutely carinate, elevated, giving the apical 1/3 of declivity transversely impressed appearance; larger, 4.2 mm	***neptis* sp. nov.**
–	Elytral bases weakly carinate, granulate; posterolateral margin of declivity moderately carinate, declivity convex, not transversely impressed; smaller, 2.4–3.0 mm	**14**
14	Strial punctures the same color as interstriae; distributed in submontane forests in northern India	*** malloti ***
–	Strial punctures much darker colored than interstriae; distributed in lowland forests in Vietnam	***geminus* sp. nov.**
15	Posterolateral margin of declivity costate and granulate (Fig. 59I)	*** velatus ***
–	Posterolateral margin of declivity carinate, never granulate (Fig. 55I)	**16**
16	Declivital interstriae 1 unarmed; tubercles on interstriae large	*** funereus ***
–	Declivital interstriae 1 bearing a few granules or tubercles; granules or tubercles on interstriae small	**17**
17	Elytral bases weakly carinate; smaller, 2.8–2.9 mm and stouter, 2.24–2.33× as long as wide	***testudinatus* sp. nov.**
–	Elytral bases oblique; larger, 3.5–4.1 mm, and more elongate, 2.4–2.73× as long as wide	**18**
18	Tubercles on declivital interstriae 2 extending from base to apex; declivity gradually sloped; declivital strial punctures shallow, giving the declivity a smooth appearance; smaller, 3.5–3.9 mm	*** interjectus ***
–	Tubercles on declivital interstriae 2 mostly absent from the apical 1/2; declivity steeply sloped; declivital strial punctures deep, giving the declivity a rugged appearance; larger, 3.9–4.1 mm	*** validus ***
19	Larger, 3.1–3.3 mm; declivital face flattened; declivital striae 1 more deeply impressed than striae 2 or 3; declivity opalescent and shagreened	*** semirudis ***
–	Smaller, 2.2–2.8 mm; declivital face convex or weakly concave; declivital striae 1 as impressed or less impressed than striae 2 and 3; declivity shiny	**20**
20	Declivital face weakly concave; declivital striae 1 not impressed; elongate, 2.75–3.25× as long as wide	*** piceus ***
–	Declivital face convex; declivital striae 1–3 equally impressed; stout, 2.2–2.55	***fornicatus* species complex (see Table 2)**

**Table 2.** Comparative table of measurements (mm) for the *Euwallacea fornicatus* species complex from Smith et al. (2019). Measurements for total length, pronotal and elytral width, length/width ratios are measured in dorsal view while pronotal and elytral length are measured in lateral view on a diagonal (Gomez et al. 2018b).

Species	Total length (mm)	Length/width ratio	Elytral length (mm)	Pronotal length (mm)	Elytron width (mm)	Pronotal width (mm)	# Protibial denticles
* fornicatior *	2.2–2.37	2.15–2.3	1.4–1.46	1.02–1.06	0.48–0.52	1.0–1.06	6–7
* fornicatus *	2.6–2.7	2.25–2.36	1.44–1.72	1.02–1.16	0.48–0.62	1.0–1.14	8–9
* kuroshio *	2.4–2.8	2.17–2.4	1.5–1.82	1.08–1.16	0.52–0.56	1.06–1.16	8–11
* perbrevis *	2.3–2.5	2.46–2.55	1.42–1.68	1.04–1.16	0.48–0.56	1.02–1.14	7–10

#### Euwallacea andamanensis

Taxon classificationAnimaliaColeopteraCurculionidae

(Blandford, 1896)

Fig. 54A, B, I

Xyleborus andamanensis Blandford, 1896b: 222.
Euwallacea andamanensis (Blandford): Wood 1989: 172.
Xyleborus noxius Sampson, 1913: 445. Synonymy: Wood and Bright 1992: 686.
Xyleborus siobanus Eggers, 1923: 186. Synonymy: Schedl 1958c: 150.
Xyleborus burmanicus Beeson, 1930: 210. Synonymy: Schedl 1970b: 224.
Xyleborus intextus Beeson, 1930: 211. Synonymy: Wood 1989: 172.
Xyleborus senchalensis Beeson, 1930: 212. Synonymy: Wood 1989: 172.
Xyleborus granulipennis Eggers, 1930: 194. Synonymy: Wood 1989: 172.
Xyleborus talumalai Browne, 1966: 248. Synonymy: Hulcr and Cognato 2013: 90.

##### Type material.

***Holotype****Xyleborus burmanicus* (FRI). ***Holotype****Xyleborus granulipennis* (FRI). ***Paratype****Xyleborus intextus* (MSUC, 2). ***Holotype****Xyleborus noxius* (NHMUK). ***Paratype****Xyleborus senchalensis* (MSUC, 1). ***Paratype****Xyleborus talumalai* (NHMUK).

##### New records.

China: Hong Kong, Kadoorie Farm, vi.2017, J. Skelton (MSUC, 1). S Yunnan, Xishuangbanna, 29 km NW Jinghong, vic. Da Nuo You NNNR, 22°12.41'N, 100°38.29'E, 790 m, fallow GF, 23.v.2008, A. Weigel (NKME, 1). India: Assam, Bhalukpong, 27°02'N, 92°35'E, 150 m, 26.v–3.vi.2006, L. Dombický (ZFMK, 1). Meghalaya, 3 km E Tura, 25°30'N, 90°14'E, 1150 m, 4.v.1999, Dombický & Pacholátko (RABC, 1). Japan: Kagoshima Pref., Tarumizu Oonohara, broadleaf forest, 425 m, 3.vii.2000, Yoshikazu Sato, ex EtOH baited trap (RJRC, 1). Laos: Bolikhamxai, Ban Nape (8 km NE), 18°21'N, 104°29'E, ~ 600 m, 1–18.v.2001, V. Kubáň (NHMB, 1; RABC, 1); Kham Mouan, Ban Khoun Ngeun, 18°07'N, 104°29'E, ~ 200 m, 24–29.iv.2001, P. Pacholátko (NHMB, 2; RABC, 1). Vietnam: Dong Nai, Cat Tien N.P., 11.46050, 107.37375, 379 m, 20.ii.2017, VN76, A.I. Cognato, T.A. Hoang, ex 8 cm diameter liana (MSUC, 63); as previous except 11°25'44"N, 107°25'44"E, 120 m, 26–31.v.1999, B. Hubley, D. Currie, VIET1H95-99 041, ex flight intercept (SEMC, 1). Thua Thien-Hue, Bach Ma N.P., 16.22897, 107.85349, 415 m, 15.ii.2017, VN57, A.I. Cognato, T.A. Hoang, ex 5 cm diameter branch; twig (MSUC, 4).

##### Diagnosis.

2.8–3.4 mm long (mean = 3.12 mm; n = 5); 2.5–2.91× as long as wide. This species is distinguished by its slender form; declivital posterolateral margin costate and granulate; pronotum appearing subquadrate when viewed dorsally (type 3); protibiae outer margins distinctly triangular bearing five or fewer large acute denticles; declivital strial punctures much larger on the declivity than on the disc; declivity broadly rounded; and declivital surface often appearing opalescent.

##### Similar species.

*Euwallacea fornicatus* species complex (*E. fornicatior*, *E. fornicatus*, *E. kuroshio*, *E. perbrevis*), *E. geminus*, *E. malloti*, *E. neptis*, *E. semirudis*, *E. testudinatus*, *E. velatus*.

##### Distribution.

Bangladesh, Federated States of Micronesia, China* (Hong Kong*, Jiangxi, Yunnan), Indonesia (Buru I., Java, Mentawai Is, Sumatra), India (Andaman Is, Assam, Bihar, Madhya Pradesh, Maharashtra, Meghalaya, Tamil Nadu, West Bengal), Japan*, Laos*, West Malaysia, Myanmar, New Guinea, Thailand, Vietnam.

##### Host plants.

Polyphagous (Beeson 1930, Browne 1961b).

##### Remarks.

This species as currently defined represents a species complex and is in need of revision (Cognato et al. 2020b).

Stouthamer et al. (2017) suggest that the synonymy of *X. talumalai* (Genbank number KU727039) with this species needs reinvestigation given the occurrence of the two species in two different clades with substantially different COI sequences. However, the findings of Stouthamer et al. are incorrect because they are based on a misidentified specimen of *E. talumalai* which is actually *E. velatus*. The DNA voucher (Euw.and 1, MSUC) and an additional specimen from the same collecting event in Thailand were examined by SMS and AIC; both specimens exhibited morphology consistent with the *E. velatus* type.

**Figure 54.**
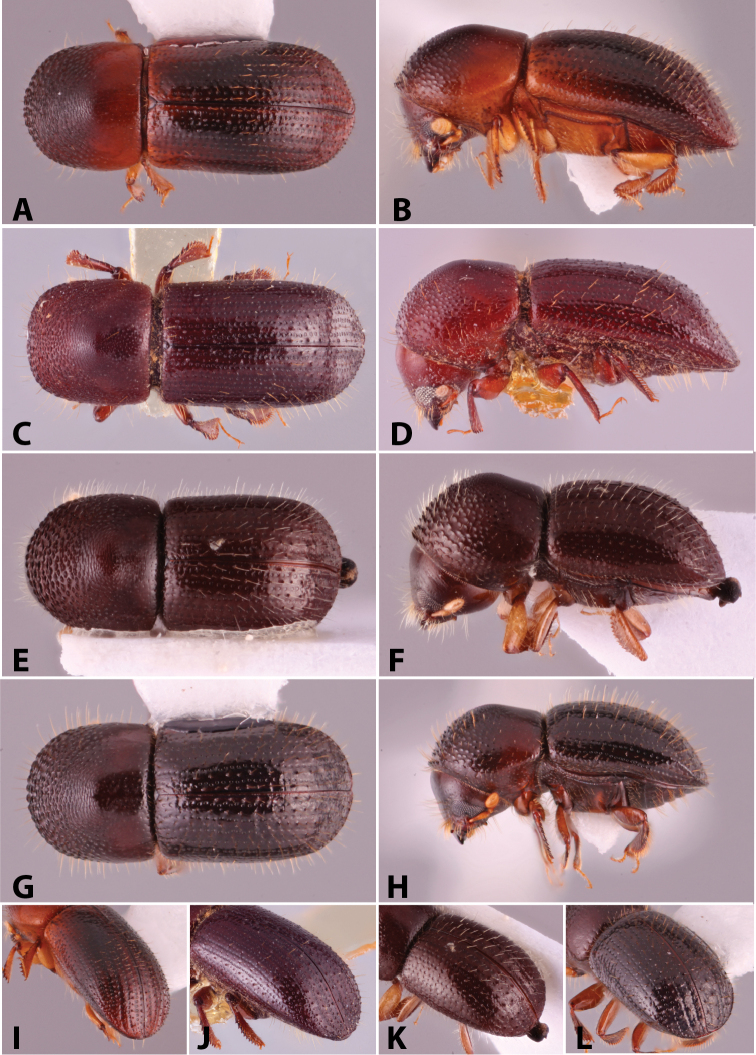
Dorsal, lateral and declivital view of *Euwallacea andamanensis*, 2.8–3.4 mm (**A, B, I**), *E. destruens*, 3.9–4.6 mm (**C, D, J**), *E. fornicatior*, 2.20–2.37 mm (**E, F, K**), and *E. fornicatus*, 2.6–2.7 mm (**G, H, L**).

#### Euwallacea aplanatus

Taxon classificationAnimaliaColeopteraCurculionidae

(Wichmann, 1914)

Xyleborus aplanatus Wichmann, 1914: 412.
Euwallacea aplanatus (Wichmann): Wood and Bright 1992: 686.

##### Type material.

***Syntypes***, possibly in Indian Museum, Kolkata, India (M. Alonso-Zarazaga pers. comm. June 2020). Not examined.

##### Diagnosis.

4.0 mm. The morphology of the species was insufficiently described in the species description. However, the species is unique in having stout elytra that are as long as wide. No other *Euwallacea* spp. have such stout elytra.

##### Similar species.

None.

##### Distribution.

India (Assam).

##### Host plants.

Unknown.

##### Remarks.

The location of the type specimens was listed as unknown (Wood and Bright 1992). Based on the description the species probably belongs in *Euwallacea*. Specimens matching the species description have not been located.

#### Euwallacea destruens

Taxon classificationAnimaliaColeopteraCurculionidae

(Blandford, 1896)

Fig. 54C, D, J

Xyleborus destruens Blandford, 1896b: 221.
Euwallacea destruens (Blandford): Wood 1989: 173.
Xyleborus barbatus Hagedorn, 1910a: 11. Synonymy: Bright and Skidmore 1997: 4, 149.
Xyleborus barbatulus Schedl, 1934b: 86. Synonymy: Bright and Skidmore 1997: 4, 149.
Xyleborus pseudobarbatus Schedl, 1942a: 193. Synonymy: Wood 1989: 173.
Xyleborus nandarivatus Schedl, 1950a: 53. Synonymy: Wood 1989: 173.
Xyleborus procerrimus Schedl, 1969a: 214. Synonymy: Hulcr and Cognato 2013: 92.

##### Type material.

***Syntype****Xyleborus barbatus* (SDEI). ***Syntype****Xyleborus destruens* (NHMUK).

##### Diagnosis.

3.9–4.6 mm long (mean = 4.19 mm; n = 6); 2.54–2.79× as long as wide. This species is distinguished by its large body size and elongate form; protibiae distinctly triangular with 4–6 denticles in the apical 1/2; declivity commencing at posterior 1/3, steeper than in *E. gravelyi*, and usually appearing concave in lateral view.

##### Similar species.

*Euwallacea gravelyi*.

##### Distribution.

From the Andaman Islands, and Southwest China, through Southeast Asia to Malaysia, Indonesia and the Philippines to New Guinea, Australia and the Pacific islands. Recorded in the study region from China (Yunnan), India (Andaman Is), Taiwan, Thailand, Vietnam.

##### Host plants.

Polyphagous (Browne 1961b).

##### Remarks.

The species is an important pest of teak (*Tectona grandis*) (Lamiaceae) in Java and other areas where there is only a short or no dry season (Browne 1968a; Kalshoven 1962).

#### Euwallacea fornicatior

Taxon classificationAnimaliaColeopteraCurculionidae

(Eggers)

Fig. 54E, F, K

Xyleborus fornicatior Eggers, 1923: 184.
Euwallacea fornicatior (Eggers): Wood and Bright 1992: 690 (as a synonym of E. fornicatus).
Xyleborus schultzei Schedl, 1951a: 68. Smith et al. 2019b: 6.

##### Type material.

***Holotype****Xyleborus fornicatior* (NMNH). ***Lectotype****Xyleborus schultzei* (NHMW).

##### Diagnosis.

2.2–2.37 mm long (mean = 2.3 mm; n = 5); 2.15–2.35× as long as wide. This species is distinguished by the pronotum basic (type 2) when viewed dorsally, anterior margin appearing rounded; declivity rounded; declivital face convex; protibiae outer margins rounded with six or seven socketed denticles, denticles small, their sockets small; declivital surface shiny; declivital interstriae bearing sparse small granules; and declivital posterolateral margin costate. This species is part of the *Euwallacea fornicatus* species complex and the most reliable method to ensure accurate identification of these species is through generation of COI barcoding sequences (Gomez et al. 2018b; Smith et al. 2019b). Specimens of *E. fornicatior* can be morphologically diagnosed through a combination of overlapping elytral and pronotal measurements and number of socketed denticles on the protibiae given in Table 2.

This species is nearly identical to *E. geminus* and *E. malloti* and can be separated by the elytral bases rounded and posterolateral declivital margin carinate and never granulate.

##### Similar species.

This species is part of the *Euwallacea fornicatus* species complex along with *E. fornicatus*, *E. kuroshio* and *E. perbrevis* from which it is difficult to distinguish. The species is also similar to *E. andamanensis*, *E. geminus*, *E. malloti*, *E. neptis*, *E. semirudis*, *E. testudinatus*, and *E. velatus*.

##### Distribution.

China (Sichuan), Federated States of Micronesia, India (Assam, Kerala, Tamil Nadu), Indonesia (Java, Sulawesi), East & West Malaysia, New Guinea, Philippines, Singapore, Sri Lanka, Taiwan, and Thailand (Smith et al. 2019b).

##### Host plants.

Recorded from *Albizzia* and *Tephrosia* (Fabaceae), durian (*Durio zibethinus*) (Malvaceae), breadfruit (*Artocarpus altilis*) (Moraceae) and tea (*Camellia sinensis*) (Theaceae) (Smith et al. 2019b).

##### Remarks.

Due to longstanding confusion of *E. fornicatior* with *E. fornicatus* and *E. perbrevis* it is quite difficult to unravel the published accounts of the biology of each species. All three species occur sympatrically on Sri Lanka where most of the natural history studies were undertaken. See the discussion on the identity of the tea shot hole borer in Smith et al. (2019b).

#### Euwallacea fornicatus

Taxon classificationAnimaliaColeopteraCurculionidae

(Eichhoff, 1868)

Fig. 54G, H, L

Xyleborus fornicatus Eichhoff, 1868b: 151.
Euwallacea fornicatus (Eichhoff): Wood 1989: 173.
Xyleborus fornicatus fornicatus Eichhoff, 1868: Beeson 1930: 234.
Xyleborus whitfordiodendrus Schedl, 1942a: 189. Synonymy: Wood 1989: 173; Smith et al. 2019b: 6.
Xyleborus tapatapaoensis Schedl, 1951b: 152. Synonymy: Wood 1989: 173.

##### Type material.

***Lectotype****Xyleborus fornicatus* (MIZ). ***Lectotype****Xyleborus tapatapaoensis* (NHMW). ***Lectotype****Xyleborus whitfordiodendrus* (NHMW).

##### Diagnosis.

2.6–2.7 mm long (mean = 2.66 mm; n = 5); 2.25–2.36× as long as wide. This species is distinguished by the pronotum basic (type 2) when viewed dorsally, anterior margin appearing rounded; declivity rounded; declivital face convex; protibiae outer margins rounded with 8 or 9 socketed denticles, denticles small, their sockets small; declivital surface shiny; declivital interstriae bearing sparse small granules; declivital posterolateral margin costate. This species is part of the *Euwallacea fornicatus* species complex and the most reliable method to ensure accurate identification of these species is through generation of COI barcoding sequences (Gomez et al. 2018b; Smith et al. 2019b). Specimens of *E. fornicatus* can be morphologically diagnosed through a combination of overlapping elytral and pronotal measurements and number of socketed denticles on the protibiae given in Table 2.

This species is nearly identical to *E. geminus* and *E. malloti* and can be separated by the elytral bases rounded and posterolateral declivital costa carinate and never granulate.

##### Similar species.

This species is part of the *Euwallacea fornicatus* species complex along with *E. fornicatior*, *E. kuroshio* and *E. perbrevis* from which it is difficult to distinguish. The species is also similar to *E. andamanensis*, *E. geminus*, *E. malloti*, *E. neptis*, *E. semirudis*, *E. testudinatus*, *E. velatus*, and *Xylosandrus formosae*.

##### Distribution.

China (Chongqing, Guizhou, Hong Kong, Yunnan), India (Uttar Pradesh), Japan (Bonin Is, Okinawa), East Malaysia, Samoa, Sri Lanka, Taiwan, Thailand, and Vietnam. This species has been introduced into Israel, South Africa, and the United States (California) (cited as PSHB and/or *E. whitfordiodendrus*; Stouthamer et al. 2017, Gomez et al. 2018b). Distribution records published prior to Smith et al. (2019b) may not reflect actual species distribution.

##### Host plants.

Strongly polyphagous and has been reported from *Sambucus* (Adoxaceae), *Liquidambar* (Altingiaceae), *Schinus* (Anacardiaceae), *Alnus* (Betulaceae), *Cunninghamia* (Cupressaceae), *Ricinus* (Euphorbiaceae), *Acacia*, *Albizia*, *Bauhinia*, *Callerya*, *Erythrina*, *Robinia* (Fabaceae), *Carya*, *Quercus* (Fagaceae), *Juglans* (Juglandaceae), *Persea*, *Umbellaria* (Lauraceae), *Magnolia* (Magnoliaceae), *Ochroma* (Malvaceae), *Ficus*, *Milicia*, *Morus* (Moraceae), *Eucalyptus* (Myrtaceae), *Fraxinus* (Oleaceae), *Platanus* (Platanaceae), *Prunus* (Rosaceae), *Populus*, *Salix* (Salicaceae), *Acer* (Sapindaceae), *Ailanthus* (Simaroubaceae), and *Ulmus* (Ulmaceae) (Smith et al. 2019b).

##### Remarks.

This species is commonly known as the Polyphagous Shot Hole Borer (PSHB) and has been referred to as this and its synonym *E. whitfordiodendrus* in numerous publications before the species complex was reassessed by Smith et al. (2019b) (e.g., Cooperband et al. 2016; Stouthamer et al. 2017; Papp et al. 2018; Gomez et al. 2018b). Due to longstanding confusion of *E. fornicatus* with *E. fornicatior* and *E. perbrevis* it is quite difficult to unravel the published accounts of the biology of each species. All three species occur sympatrically on Sri Lanka where most of the natural history studies were undertaken. See the discussion on the identity of the tea shot hole borer in Smith et al. (2019b).

Various aspects of the biology of the species are described by Mendel et al. (2012), Eskalen et al. (2013), Freeman et al. (2013), O’Donnell et al. (2015)Chen et al. (2016)Cooperband et al. (2016), Stouthamer et al. (2017).

#### Euwallacea funereus

Taxon classificationAnimaliaColeopteraCurculionidae

(Lea, 1910)

Fig. 55A, B, I

Xyleborus funereus Lea, 1910: 139.
Ambrosiodmus funereus (Lea): Wood 1989: 169.
Euwallacea funereus (Lea): Hulcr and Cognato 2010a: 16.
Xyleborus nepos Eggers, 1923: 198. Synonymy: Schedl 1933: 103.
Xyleborus nepos robustus Schedl, 1933: 103. Synonymy: Wood 1989: 169–170.
Xyleborus signatus Schedl, 1949: 278. Synonymy: Wood 1975a: 23.

##### Type material.

***Lectotype****Xyleborus nepos* (NMNH).

##### Diagnosis.

3.45–3.7 mm long (mean = 3.65 mm; n = 5); 2.46–2.55× as long as wide. This species is distinguished by the pronotum appearing subquadrate (type 3) from dorsal view; outer margin of protibiae round; declivital interstriae 1 unarmed; declivital posterolateral margin carinate, never granulate; and moderately large size.

##### Similar species.

*Euwallacea interjectus*, *E. validus*.

##### Distribution.

Australia, India (Andaman Is, Nicobar Is), Indonesia (Java, Sumatra, Sumbawa, Sulawesi, Ternate), East Malaysia, New Guinea, Philippines, Solomon Islands, Taiwan, Thailand.

##### Host plants.

Polyphagous (Kalshoven 1959b).

##### Remarks.

Kalshoven (1959b) gives details of brood sizes at various stages of development of the gallery system.

**Figure 55.**
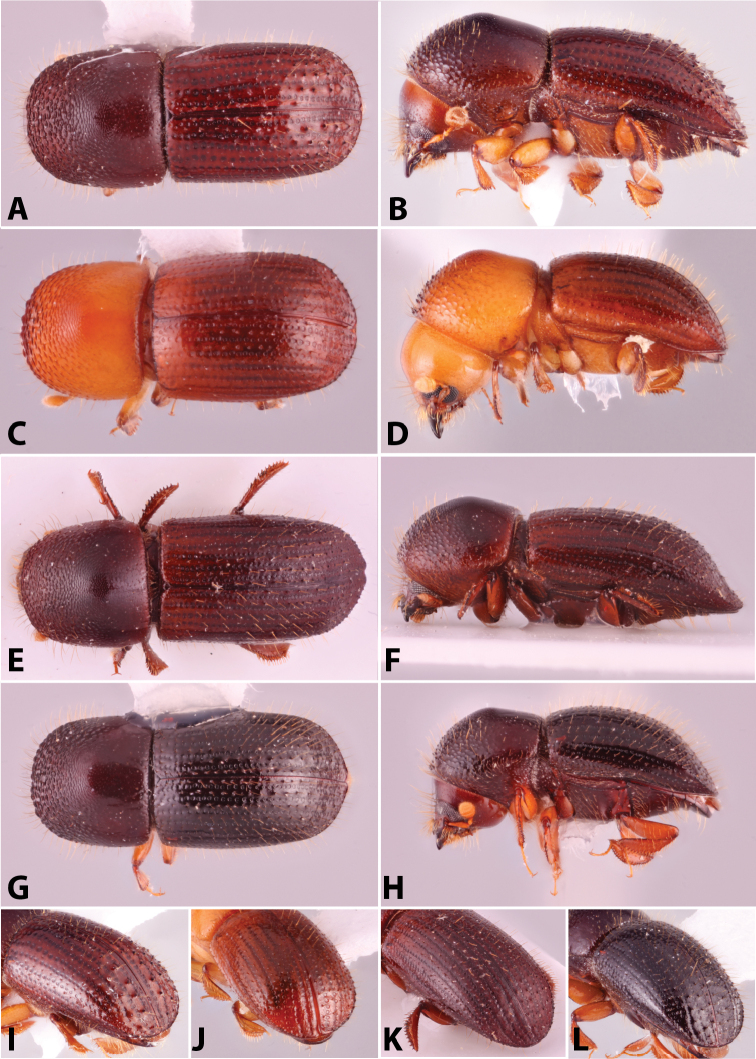
Dorsal, lateral and declivital view of *Euwallacea funereus*, 3.45–3.7 mm (**A, B, I**), *E. geminus* holotype, 2.7–2.8 mm (**C, D, J**), *E. gravelyi*, 4.6–5.7 mm (**E, F, K**), and *E. interjectus*, 3.5–3.9 mm (**G, H, L**).

#### Euwallacea geminus sp. nov.

Taxon classificationAnimaliaColeopteraCurculionidae

http://zoobank.org/BADB9195-959E-47C7-87B9-0C0F57279F34

Fig. 55C, D, J

##### Type material.

***Holotype***, female, Vietnam: Dong Nai, Cat Tien N.P., 11.44221, 107.43114, 379 m, 20.ii.2017, VN79, A.I. Cognato, T.A. Hoang, ex 4 cm diameter branch (MSUC). ***Paratypes***, female, as holotype (MSUC, 1; NHMUK, 1; NMNH, 2; VNMN, 2).

##### Diagnosis.

2.7–2.8 mm long (mean = 2.72 mm; n = 5); 2.33–2.35× as long as wide. This species is distinguished by the pronotum basic (type 2) when viewed dorsally, anterior margin appearing rounded; elytral bases weakly costate, granulate; declivity rounded; declivital face convex; protibiae outer margins rounded with at least eight socketed denticles, denticles small, their sockets small; declivital surface shiny; declivital interstriae bearing sparse small granules; posterolateral declivital margin carinate and granulate; and elytral bases weakly carinate and granulate.

This species is identical to *E. malloti* and can only reliably be identified with molecular data. Without molecular data it can be distinguished by its distribution in lowland forests (*E. malloti* primarily occurs in submontane forests of the Himalayas) and by the often unique coloration of the elytral striae which are dark brown in mature specimens making the punctures appear very distinctive and clear to see (*E. malloti* strial punctures not colored differently from the rest of the elytra). This species is also nearly identical to the *E. fornicatus* species complex and can be separated by the elytral bases weakly carinate and posterolateral declivital margin costate and granulate.

##### Similar species.

*Euwallacea fornicatus* species complex (*E. fornicatior*, *E. fornicatus*, *E. kuroshio*, *E. perbrevis*), *E. andamanensis*, *E. malloti*, *E. semirudis*, *E. neptis*, *E. testudinatus*, *E. velatus*.

##### Description

**(female).** 2.7–2.8 mm long (mean = 2.72 mm; n = 5); 2.33–2.35× as long as wide. Pronotum, head, antennae, and legs light brown, elytra darker, red-brown. ***Head***: epistoma entire, transverse, with a row of hair-like setae. Frons weakly convex to upper level of eyes; surface strongly shiny, median 2/3 smooth, impunctate, lateral 1/3 sparsely and finely punctate, setose; punctures bearing a long, erect hair-like seta. Eyes shallowly emarginate just above antennal insertion, upper part smaller than lower part. Submentum narrow, triangular, slightly impressed. Antennal scape long and slender, as long as club. Pedicel as wide as scape, shorter than funicle. Funicle 4-segmented, segment 1 as long as pedicel. Club longer than wide, flat, type 3; segment 1 corneous, transverse on anterior face, occupying approximately basal 1/4; segment 2 narrow, corneous; segments 1–3 present on posterior face. ***Pronotum***: 0.93× as long as wide. In dorsal view basic and parallel-sided, sides parallel in basal 3/4, rounded anteriorly; anterior margin without serrations. In lateral view tall, type 2, disc flat, summit at basal 2/5. Anterior slope with densely spaced, broad asperities, becoming lower and more strongly transverse towards summit. Disc strongly shiny with sparse, minute punctures, some longer hair-like setae at margins. Lateral margins obliquely costate. Base transverse, posterior angles acutely rounded. ***Elytra***: 1.24× as long as wide, 1.3× as long as pronotum. Scutellum broad, moderately sized, linguiform, shiny, flush with elytra, flat. Elytral base transverse, edge weakly costate and granulate, humeral angles rounded, parallel-sided in basal 3/4, then broadly rounded to apex. Disc convex, shiny, striae not impressed, with large, shallow punctures separated by one diameter of a puncture, glabrous; interstriae flat, impunctate, granulate, granules sparse and widely spaced, each granule with a long, erect seta. Declivity occupying approximately 1/2 of elytra, rounded, declivital face convex, shiny; striae not impressed, strial punctures much larger and deeper than those of disc; interstriae granulate, granules as described for disc, interstriae weakly laterally broadened from declivital summit to apical 1/3 then narrowed to apex. Posterolateral margin carinate, granulate. ***Legs***: procoxae contiguous; prosternal coxal piece tall, pointed. Protibiae broad, semi-circular, with rounded outer margin, broadest at apical 1/3; posterior face smooth; apical 1/2 of outer margin with eight large socketed denticles, their length longer than basal width. Meso- and metatibiae flattened; outer margin evenly rounded with 11 small socketed denticles.

##### Etymology.

L. *geminus* = twin. In reference to this species the very close morphology to *E. malloti*. An adjective.

##### Distribution.

Vietnam.

##### Host plants.

Unknown.

#### Euwallacea gravelyi

Taxon classificationAnimaliaColeopteraCurculionidae

(Wichmann, 1914) stat. res.

Fig. 55E, F, K

Xyleborus gravelyi Wichmann, 1914: 411.
Euwallacea gravelyi (Wichmann): Saha and Maiti 1996: 815.
Xyleborus ovalicollis Eggers, 1930: 193. Synonymy: Saha and Maiti 1996: 815.
Xyleborus barbatomorphus Schedl, 1951a: 72. syn. nov.

##### Type material.

***Holotype****Xyleborus barbatomorphus* (NHMW), ***paratype*** (NHMW, 1). ***Holotype****Xyleborus ovalicollis* (FRI).

##### New records.

Bhutan: W. Paro distr., Gedu, 2100 m, 17–26.vi.1988, C. Holzschuh (RABC, 1). China: Yunnan, Lincang, Genma, 12.xii.2018, Y. Li, ex rubber tree (MSUC, 1). India: Arunachal Pradesh, 0.3 km SSE of Dirang, 27°20'32"N, 92°16'17"E, 1550 m, 27.iv–1.v.2008, H. Podskalská & P. Šipek (NHMP, 1); as previous except: Etalin vicinity, 28°36'56"N, 95°53'21"E, 700 m, L. Dembický, 12–25.v.2012 (ZFMK, 2). Meghalaya, Nokrek N.P., 3 km S Darbokgiri, 25°27'N, 90°19'E, 1400 m, 26.iv.1999, Dombický, Pacholátko (RABC, 1). Laos: Bolikhamzai, Ban Nape (8 km NE), 18°21'N, 105°08'E, 600 m, 1–18.v.2001, V. Kuban (NHMB, 6; RABC, 3). Champasak, Bolavens Plateau, waterfall ~ 2 km E Tao Katamtok, 15°08.1'N, 106°38.8'E, 415 m, 10–12.v.2010, J. Hájek (NHMP, 1). Taiwan: Nantou, Sun Moon Lake, C.-S. Lin 15.v.2014 (MSUC, 1). Thailand: Chiang Mai, Doi Pui, 1400 m, 6–10.vi.2005, W. Puranasakul (RABC, 1). Vietnam: Tonkin, Hoa-Binh, 1940, A. De Cooman (MNHN, 1).

##### Diagnosis.

4.6–5.7 mm long (mean = 5.21 mm; n = 8); 2.6–2.75× as long as wide. This species is distinguished by its large size and elongate form; protibiae less distinctly triangular than *E. destruens*, and with 7–9 denticles in apical 1/2; declivity commencing at approximately midpoint, evenly curved from disc into declivity; and declivity usually appearing flat in lateral view.

##### Similar species.

*Euwallacea destruens*.

##### Distribution.

Bhutan*, China* (Yunnan), India (Arunachal Pradesh*, Assam, Meghalaya*, West Bengal), Laos*, Myanmar, Taiwan, Thailand*, Vietnam*.

##### Host plants.

Polyphagous (Saha and Maiti 1996).

##### Remarks.

This species was included in *Xyleborus* by Wood and Bright (1992) but was transferred to *Euwallacea* by Saha and Maiti (1996) with *Xyleborus ovalicollis* as a synonym. The location of the two syntypes of *E. gravelyi* is not known, but Wichmann’s description is sufficiently detailed for us to be able to confirm the synonymy given by Saha and Maiti (1996). Maiti and Saha (2004) included both species as synonyms of *Euwallacea wallacei* (Blandford), presumably following the placement of *E. ovalicollis* as a synonym of *E. wallacei* by Schedl (1970a) and Wood (1989). However, *E. wallacei* is a distinct species only superficially similar to *E. gravelyi*. In *E. gravelyi*, the eyes are of normal size, and the upper part is smaller than the lower; in *E. wallacei*, the eyes are unusually large and extend onto the frons, the upper and lower parts are of equal size. In *E. gravelyi*, the protibiae bears 7–9 small denticles in the apical 1/2; in *E. wallacei*, there are only five large denticles.

*Xyleborus barbatomorphus* was given as a synonym of *E. wallacei* by Beaver et al. (2014), but is in fact conspecific with *E. gravelyi* and is here placed in synonymy. *E. wallacei* is not known to be present in the area covered by this study.

#### Euwallacea interjectus

Taxon classificationAnimaliaColeopteraCurculionidae

(Blandford, 1894)

Fig. 55G, H, L

Xyleborus interjectus Blandford, 1894c: 576.
Euwallacea interjectus (Blandford): Saha and Maiti 1984: 2.
Xyleborus pseudovalidus Eggers, 1925: 159. Synonymy: Schedl 1958a: 155.

##### Type material.

***Holotype****Xyleborus interjectus* (NHMUK). ***Syntype****Xyleborus pseudovalidus* (NHMP).

##### New records.

China: Chongqing, Jinfo Mtn, Tian-Shang, Lv-Jia, ex *Ficus* sp. (RABC, 2). Hong Kong, Kadoorie Farm, vi.2017, J. Skelton (UFFE, 1). Jiangsu, Nanjing, Laoshan National Park, Bacai Road, 32.09156N, 118.583701E, 15.viii.2017, Cognato, Li, Gao, ex paper mulberry (MSUC, 5). Jiangxi, Jiu Jiang, 22.viii.2016, Lv-Jia, Tian-Shang, ex *Liquidambar formosana* (RABC, 1). India: Arunachal Pradesh, Etalin vicinity, 28°36'56"N, 95°53'21"E, 700 m, L. Dembický, 12–25.v.2012 (ZFMK, 53). Laos: Bolikhamxai, Ban Nape (8 km NE), 18°21'N, 105°08'E, 600 m, 1–18.v.2001, V. Kubáň (NHMB, 2). NE, Houa Phan, Ban Saluei, Phou Pan Mt., 20°12'N, 104°01'E, 1300–1900 m, 7.iv–25.v.2010, C. Holzschuh (NHMUK, 2); as previous except: 27.iv–1.vi.2011 (NHMUK, 12; RABC, 4). Kham Mouan, Ban Khun Ngeun, 18°07'N, 104°29'E, ~ 200 m, 24–29.iv.2001, Pacholátko (NHMB, 1). Oudomxai, Oudomxai, 17 km NE, 20°45'N, 102°09'E, ~ 1100 m, 1–9.v.2002, V. Kubáň (NHMB, 1). Vientiane, Ban Van Eue, 15.ii.1965, native collector (BPBM, 2).

##### Diagnosis.

3.5–3.9 mm long (mean = 3.78 mm; n = 5); 2.4–2.64× as long as wide. This species is distinguished by the pronotum appearing subquadrate (type 3) from dorsal view; outer margin of protibiae round; declivital interstriae 1 granulate; declivital posterolateral margin carinate, never granulate; and moderately large size.

It can be further separated from the strongly morphologically similar species *E. validus* by the gradually sloped declivity; declivital strial punctures shallow giving the declivity a smooth appearance; and tubercles on interstriae 2 extending from base to apex.

##### Similar species.

*Euwallacea funereus*, *E. validus*, *E. velatus*.

##### Distribution.

From the Indian subcontinent, China and South Korea through Southeast Asia and Indonesia to the Philippines, New Guinea and Solomon Islands. Introduced to North America, Hawaii and South America (Argentina) (Halbert 2011; Cognato et al. 2015; Gomez et al. 2018; Landi et al. 2019). Recorded in the study region from Bangladesh, China (Anhui, Chongqing*, Fujian, Gansu, Guangdong, Guizhou, Hainan, Hong Kong*, Hubei, Hunan, Jiangsu*, Jiangxi*, Sichuan, Yunnan, Xizang), India (Andaman Is, Arunachal Pradesh* Assam, Kerala, Madhya Pradesh, Maharashtra, Meghalaya, Sikkim, Tamil Nadu, Uttarakhand, West Bengal), Laos*, Myanmar, Nepal, Taiwan, Thailand, Vietnam.

##### Host plants.

Polyphagous (Beeson 1930; Browne 1961b).

#### Euwallacea kuroshio

Taxon classificationAnimaliaColeopteraCurculionidae

Gomez & Hulcr, 2018

Fig. 56A, B, I

Euwallacea kuroshio Gomez & Hulcr, 2018 (in Gomez et al. 2018b): 9.

##### Type material.

***Holotype*** (NMNH).

##### Diagnosis.

2.4–2.8 mm long (mean = 2.6 mm; n = 5); 2.17–2.4× as long as wide. This species is distinguished by the pronotum basic (type 2) when viewed dorsally, anterior margin appearing rounded; declivity rounded; declivital face convex; protibiae outer margins rounded with 8–11 socketed denticles, denticles small, their sockets small; declivital surface shiny; interstriae bearing sparse small granules; and posterolateral declivital margin costate. This species is part of the *Euwallacea fornicatus* species complex and the most reliable method to ensure accurate identification of these species is through generation of COI barcoding sequences (Gomez et al. 2018b; Smith et al. 2019b). Specimens of *E. kuroshio* can be morphologically diagnosed through a combination of overlapping elytral and pronotal measurements and number of socketed denticles on the protibiae given in Table 2.

This species is nearly identical to *E. geminus* and *E. malloti* and can be separated by the elytral bases rounded and posterolateral declivital costa carinate and never granulate.

##### Similar species.

This species is part of the *Euwallacea fornicatus* species complex along with *E. fornicatior*, *E. fornicatus* and *E. perbrevis* from which it is difficult to distinguish. The species is also similar to *E. andamanensis*, *E. geminus*, *E. malloti*, *E. neptis*, *E. semirudis*, *E. testudinatus*, *E. velatus*, and *Xylosandrus formosae*.

##### Distribution.

This species is reported in the study region from Indonesia, Japan (Okinawa), and Taiwan. It has been introduced to Mexico and the United States (California) (Stouthamer et al. 2017; Gomez et al. 2018a; Smith et al. 2019b).

##### Host plants.

This species is polyphagous and reported from *Sambucus* (Adoxaceae), *Liquidambar* (Altingiaceae), *Schinus*, *Searsia* (Anacardiaceae), *Ambrosia*, *Baccharis* (Asteraceae), *Alnus* (Betulaceae), *Ricinus* (Euphorbiaceae), *Quercus* (Fagaceae), *Juglans*, *Pterocarya* (Juglandaceae), *Cassia*, *Persea* (Lauraceae), *Ficus* (Moraceae), *Eucalyptus* (Myrtaceae), *Magnolia* (Magnoliaceae), *Fraxinus* (Oleaceae), *Platanus* (Platanaceae), *Populus*, *Salix* (Salicaceae), *Nicotiana* (Solanaceae), *Tamarix* (Tamaricaceae) (Smith et al. 2019b).

##### Remarks.

This species is commonly known as the Kuroshio Shot Hole Borer (KSHB) and has been referred to as this in publications before the species was formally described (e.g., Stouthamer et al. 2017).

**Figure 56.**
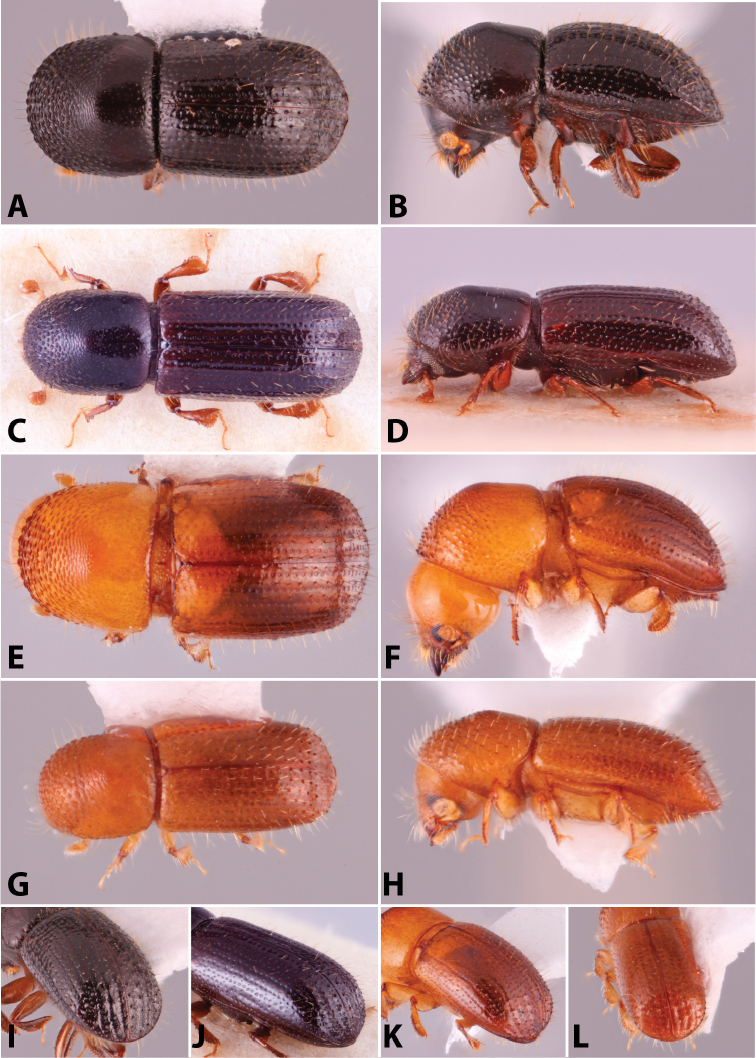
Dorsal, lateral and declivital view of *Euwallacea kuroshio*, 2.4–2.8 mm (**A, B, I**), *E. luctuosus* holotype, 3.6 mm (**C, D, J**), *E. malloti*, 2.4–3.0 mm (**E, F, K**), and *E. minutus*, 1.8–1.9 mm (**G, H, L**).

#### Euwallacea luctuosus

Taxon classificationAnimaliaColeopteraCurculionidae

(Eggers, 1939)

Fig. 56C, D, J

Xyleborus luctuosus Eggers, 1939a: 13.
Euwallacea luctuosus (Eggers): Wood and Bright 1992: 691.

##### Type material.

***Holotype*** (NHRS).

##### Diagnosis.

3.6 mm long (n = 1); 3.6× as long as wide. This species is the most slender of the *Euwallacea* species. It can be further recognized by the posterolateral margins of declivity weakly costate; pronotum from dorsal view elongate, anterior margin rounded (type 7); and outer margin of protibiae obliquely triangular and bearing seven large denticles, their bases contiguous.

##### Similar species.

*Euwallacea sibsagaricus*, *E. subalpinus*, *Heteroborips tristis*.

##### Distribution.

Myanmar.

##### Host plants.

Unknown.

#### Euwallacea malloti

Taxon classificationAnimaliaColeopteraCurculionidae

(Eggers, 1930)

Fig. 56E, F, K

Xyleborus malloti Eggers, 1930: 192.
Euwallacea malloti (Eggers): Wood and Bright 1992: 692.

##### Type material.

***Holotype*** (FRI).

##### New records.

India: Uttarakhand, Dehradun, Forest Research Institute, 30°20'24"N, 78°0'14"E, 2223’, 16–26.i.2017, A.I. Cognato, ex small branch of *Melia dubia* (MSUC, 5; NHMUK, 1; NMNH, 1).

##### Diagnosis.

2.4–3.0 mm long (mean = 2.62 mm; n = 5); 2.08–2.7× as long as wide. This species is distinguished by the pronotum basic (type 2) when viewed dorsally, anterior margin appearing rounded; declivity rounded; declivital face convex; protibiae outer margins rounded with at least seven socketed denticles, denticles small, their sockets small; declivital surface shiny; declivital interstriae bearing sparse small granules, posterolateral declivital margin carinate and granulate; and elytral bases weakly carinate and granulate.

This species is identical to *Euwallacea geminus* and can only reliably be identified with molecular data. Without molecular data it can be distinguished by its distribution primarily in submontane forests of the Himalayas (*E. geminus* occurs in lowland forests in Vietnam) and by the elytral strial punctures not colored differently from the rest of the elytra (*E. geminus* typically has unique coloration of the elytral striae which are dark brown in mature specimens making the punctures appear very distinctive and clear to see. This species is nearly identical to *E. fornicatus* species complex and can be separated by the elytral bases weakly carinate and posterolateral declivital costa granulate.

##### Similar species.

*Euwallacea fornicatus* species complex (*E. fornicatior*, *E. fornicatus*, *E. kuroshio*, *E. perbrevis*), *E. andamanensis*, *E. geminus*, *E. neptis*, *E. semirudis*, *E. testudinatus*, *E. velatus*.

##### Distribution.

India (Meghalaya, Tamil Nadu, Uttarakhand, West Bengal).

##### Host plants.

Recorded from *Mallotus* (Euphorbiaceae), *Phoebe* (Lauraceae), *Tinospora* (Menispermaceae), *Eugenia* (Myrtaceae) (Maiti and Saha 2004), and *Melia* (Meliaceae).

#### Euwallacea minutus

Taxon classificationAnimaliaColeopteraCurculionidae

(Blandford, 1894)

Fig. 56G, H, L

Xyleborus minutus Blandford, 1894b: 116.
Planiculus minutus (Blandford): Beaver and Liu 2010: 29.
Wallacellus minutus (Blandford): Beaver et al. 2014: 61.
Euwallacea minutus (Blandford): Storer et al. 2015: 395.
Xyleborus breviusculus Schedl, 1942a: 196. Synonymy: Schedl 1958c: 147.
Xyleborus pernitidus Schedl, 1954a: 152. Synonymy: Schedl 1958c: 147.

##### Type material.

***Syntypes****Xyleborus minutus* (NHMUK).

##### New records.

China: Jiangxi, Xunwu, Xingshan, 6.ix.2018, Y. Li, ex Fagaceae log (UFFE, 1). Laos: Vientiane, Ban Van Eue, 15.viii.1966, native collector (BPBM, 1). Philippines: Calmarines Norte, Mount Labo, Basecamp, 14°04.546'N, 122°46.146'E, 237 m, 5.vi.2016, Siler Brachymeies Expedition 2, ex pan traps, Department of Recent Invertebrates OMNH-66417 (OMNH, 1). Vietnam: Cao Bang, 22°34.118'N, 105°52.537'E, 1048 m, 12–17.iv.2014, VN9, Cognato, Smith, Pham, ex FIT (MSUC, 1). Thua Thien-Hue, Bach Ma N.P., 16.22897, 107.85349, 415 m, 15.ii.2017, VN60, A.I. Cognato, T.A. Hoang, ex 4 cm diameter branch (MSUC, 2).

##### Diagnosis.

1.8–1.9 mm long (mean = 1.87 mm; n = 3); 2.57–2.71× as long as wide. This species is distinguished by its minute size; short, steep declivity with two transverse rows of granules on each interstriae at declivital summit; pronotum from dorsal view elongate (type 7); and pronotal asperities small, coarse.

##### Similar species.

*Euwallacea semiermis*.

##### Distribution.

Brunei, China (Chongqing, Jiangxi*, Yunnan), Indonesia (Java), Japan, Korea, Laos, East & West Malaysia, Philippines*, Solomon Islands, Taiwan, Thailand, Vietnam*.

##### Host plants.

Polyphagous (Browne 1961b; Beaver and Browne 1979; Choo and Woo 1985).

#### Euwallacea neptis sp. nov.

Taxon classificationAnimaliaColeopteraCurculionidae

http://zoobank.org/9515C889-C9C5-492A-B399-BCD8FF84B4AB

Fig. 57A, B, I

##### Type material.

***Holotype***, female, India: Darjeeling, Rangirum, 6000 ft, J.C.M. Gardner, 5.ix.1929, ex misc. timber (NMNH).

##### Diagnosis.

4.2 mm long (n = 1); 2.8× as long as wide. This species is distinguished by the pronotum basic (type 2) when viewed dorsally, anterior margin appearing rounded; and elytral bases rounded, never granulate; declivity gradual, declivital face flat, opalescent; declivital striae impressed, strial punctures large; posterolateral declivital margin elevated, acutely carinate, giving the apical 1/3 of declivity a transversely impressed appearance; and large size.

##### Similar species.

*Euwallacea fornicatus* species complex (*E. fornicatior*, *E. fornicatus*, *E. kuroshio*, *E. perbrevis*), *E. andamanensis*, *E. geminus*, *E. malloti*, *E. semirudis*, *E. testudinatus*, *E. velatus*.

##### Description

**(female).** 4.2 mm long (n = 1); 2.8× as long as wide. Body dark red-brown. Legs and antennae light brown. ***Head***: epistoma entire, transverse, with a row of hair-like setae. Frons weakly convex to upper level of eyes; surface strongly shiny, sparsely, finely punctate, setose; punctures bearing a long, erect hair-like seta. Eyes deeply emarginate just above antennal insertion, upper part smaller than lower part. Submentum narrow, triangular, slightly impressed. Antennal scape long and slender, longer than club. Pedicel as wide as scape, shorter than funicle. Funicle 4-segmented, segment 1 longer than pedicel. Club longer than wide, flat, type 3; segment 1 corneous, transverse on anterior face, occupying approximately basal 1/4; segment 2 narrow, corneous; segments 1–3 present on posterior face. ***Pronotum***: 0.92× as long as wide. In dorsal view basic and parallel-sided, sides parallel in basal 1/2, rounded anteriorly; anterior margin without serrations. In lateral view tall, type 2, disc flat, summit at basal 2/5. Anterior slope with densely spaced, broad asperities, becoming lower and more strongly transverse towards summit. Disc strongly shiny with sparse, minute punctures, some longer hair-like setae at margins. Lateral margins obliquely costate. Base transverse, posterior angles acutely rounded, almost quadrate. ***Elytra***: 1.7 × as long as wide, 1.83× as long as pronotum. Scutellum moderately sized, linguiform, flush with elytra, flat, shiny. Elytral base transverse, edge oblique and unarmed by granules, humeral angles rounded, parallel-sided in basal 3/4, then broadly rounded to apex. Disc convex, shiny, striae not impressed, with large, shallow punctures separated by less than one diameter of a puncture, glabrous; interstriae flat, very sparsely finely punctate, punctures 1/3 size of strial punctures, each with a long, erect seta. Declivity occupying approximately 2/5 of elytra, gradual, face flat, opalescent, apical 1/3 appearing transversely impressed; striae impressed, strial punctures much larger and deeper than those of disc; interstriae 2–4 with three widely spaced tubercles on basal 1/2, apical 1/2 unarmed. Posterolateral margin elevated, acutely carinate and granulate to interstriae 7. ***Legs***: procoxae contiguous; prosternal coxal piece tall, pointed. Protibiae broad, semi-circular, with rounded outer margin; posterior face smooth; apical 1/3 of outer margin with eight small, widely spaced socketed denticles, their length shorter longer than basal width. Meso- and metatibiae flattened; outer margin evenly rounded with 12 small socketed denticles.

##### Etymology.

L. *neptis* = granddaughter. In reference to its similarity to several *Euwallacea* species. Noun in apposition.

##### Distribution.

India (West Bengal).

##### Host plants.

Unknown.

**Figure 57.**
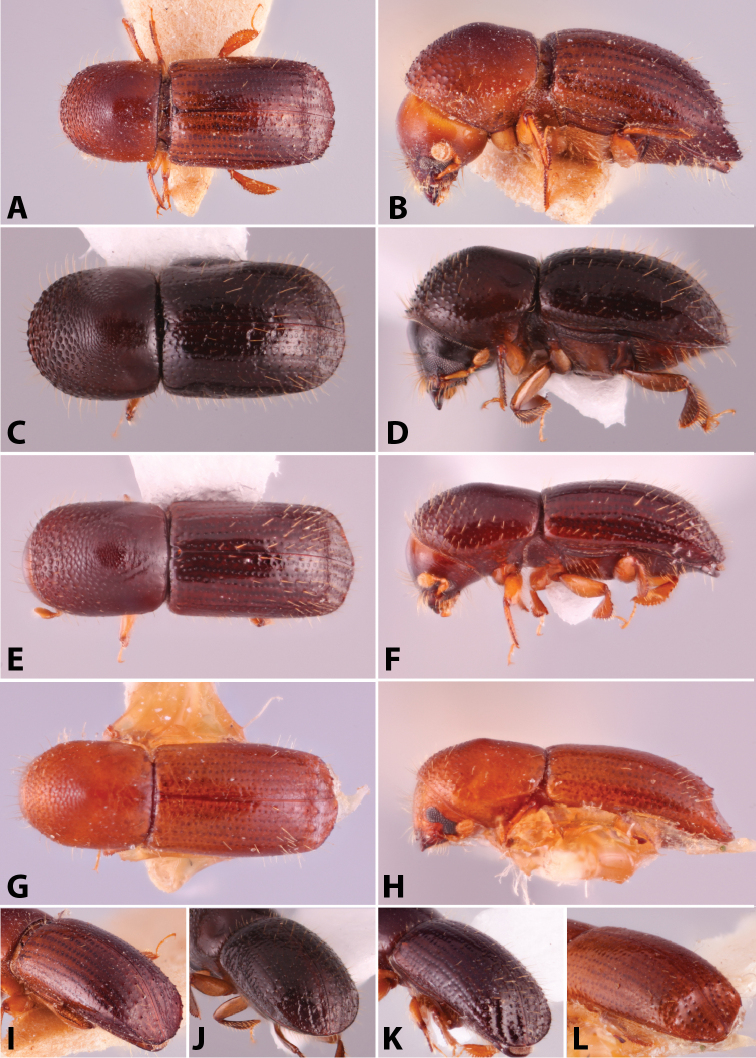
Dorsal, lateral and declivital view of *Euwallacea neptis* holotype, 4.2 mm (**A, B, I**), *E. perbrevis*, 2.3–2.5 mm (**C, D, J**), *E. piceus*, 2.2–2.6 mm (**E, F, K**), and *E. semiermis* lectotype, 2.5–2.75 mm (**G, H, L**).

#### Euwallacea perbrevis

Taxon classificationAnimaliaColeopteraCurculionidae

(Schedl, 1951)

Fig. 57C, D, J

Xyleborus perbrevis Schedl, 1951a: 59.
Euwallacea perbrevis (Schedl): Wood 1989: 173 (as a synonym of E. fornicatus).
Xyleborus molestulus Wood, 1975b: 400. syn. nov.

##### Type material.

***Holotype****Xyleborus perbrevis* (NHMW). ***Holotype****Xyleborus molestulus* (NMNH).

##### Diagnosis.

2.3–2.5 mm long (mean = 2.44 mm; n = 5); 2.46–2.55× as long as wide. This species is distinguished by the pronotum basic (type 2) when viewed dorsally, anterior margin appearing rounded; declivity rounded; declivital face convex; protibiae outer margins rounded with 7–10 socketed denticles, denticles small, their sockets small; declivital surface shiny; interstriae bearing sparse small granules; and posterolateral declivital margin costate. This species is part of the *Euwallacea fornicatus* species complex and the most reliable method to ensure accurate identification of these species is through generation of COI barcoding sequences (Gomez et al. 2018b; Smith et al. 2019b). Specimens of *E. perbrevis* can be morphologically diagnosed through a combination of overlapping elytral and pronotal measurements and number of socketed denticles on the protibiae given in Table 2.

This species is nearly identical to *E. geminus* and *E. malloti* and can be separated by the elytral bases rounded and posterolateral declivital costa carinate and never granulate.

##### Similar species.

This species is part of the *Euwallacea fornicatus* species complex along with *E. fornicatior*, *E. fornicatus*, *E. kuroshio* from which it is difficult to distinguish. The species is also similar to *E. andamanensis*, *E. geminus*, *E. malloti*, *E. neptis*, *E. semirudis*, *E. testudinatus*, *E. velatus*, and *Xylosandrus formosae*.

##### Distribution.

This species occurs in American Samoa, Australia, Brunei, China (Hainan), Fiji, Indonesia (Java), Japan (Okinawa), East & West Malaysia, New Guinea, Palau, Philippines, Réunion, Singapore, Sri Lanka, Taiwan, Thailand, Timor Leste, Vietnam, and introduced in the United States (Florida and Hawaii) (Gomez et al. 2018b), Costa Rica and Panama (Kirkendall and Ødegaard 2007, reported as *E. fornicatus*) (Smith et al. 2019b).

##### Host plants.

The species is strongly polyphagous and has been recorded from 13 families: *Avicennia* (Acanthaceae), *Mangifera* (Anacardiaceae), *Annona* (Annonaceae), *Cyathocalyx*, *Xylopia* (Annonaceae), *Bursera*, *Protium* (Burseraceae), *Terminalia* (Combretaceae), *Aleurites* (Euphorbiaceae), *Acacia*, *Albizia*, *Erythrina*, *Lysiloma* (Fabaceae), *Theobroma* and *Trichospermum* (Malvaceae), *Cedrela* (Meliaceae), *Artocarpus*, *Brosimum* (Moraceae), *Myristica* (Myristicaceae), *Citrus* (Rutaceae), *Casearia* (Salicaceae), *Litchi* (Sapindaceae), and *Camellia sinensis* (Theaceae) (Smith et al. 2019b).

##### Remarks.

*Xyleborus molestulus* Wood was described from specimens collected in the Panama Canal Zone and western Panama in 1963. Wood (1982: 775) later transferred the species to the endemic Neotropical genus *Theoborus* Hopkins, 1915 presumably because of similar morphological features. In 1982 Wood reported *Xyleborus fornicatus* from the Canal Zone from specimens collected in 1979. Based on a recent revision of the *E. fornicatus* species complex, *E. perbrevis* was recognized as the species of the complex occurring in Panama (Gomez et al. 2018b, Smith et al. 2019b). The *X. molestulus* and *E. perbrevis* holotypes and specimens collected from the Canal Zone (MSUC) are identical.

*Euwallacea perbrevis* was previously thought to be a synonym of *E. fornicatus* (Wood 1989; Gomez et al. 2018b) but a subsequent reanalysis of the complex by Smith et al. (2019b) showed that the species is a distinct lineage. This species is commonly known as the Tea Shot Hole Borer (TSHB) and has been referred to as this, as well as *E. fornicatus*, which it was misidentified as in numerous publications before the species complex was reassessed by Smith et al. (2019b). Due to longstanding confusion of *E. perbrevis* with *E. fornicatior* and *E. fornicatus* it is quite difficult to untangle the published accounts of the biology of each species. All three species occur sympatrically on Sri Lanka where most of the natural history studies were undertaken and where *E. perbrevis* is a serious pest of tea plantations. See the discussion on the identity of the tea shot hole borer in Smith et al. (2019b).

Various aspects of the biology of the species are described by Freeman et al. (2013), O’Donnell et al. (2015), Chen et al. (2016), Cooperband et al. (2016), Stouthamer et al. (2017) and Lynn et al. (2020).

#### Euwallacea piceus

Taxon classificationAnimaliaColeopteraCurculionidae

(Motschulsky, 1863)

Fig. 57E, F, K

Anodius piceus Motschulsky, 1863: 512.
Euwallacea piceus (Motschulsky): Wood and Bright 1992: 692.
Wallacellus piceus (Motschulsky): Hulcr and Cognato 2010a: 29.
Euwallacea piceus (Motschulsky): Storer et al. 2015: 396.
Xyleborus indicus Eichhoff, 1878a: 392. Synonymy: Wood 1969: 117.
Xyleborus imitans Eggers, 1927a: 404. Synonymy: Wood 1969: 117.
Xyleborus indicus subcoriaceus Eggers, 1927b: 92. Synonymy: Schedl 1959: 504.
Xyleborus samoensis Beeson, 1929: 237. Synonymy: Wood 1960: 63.

##### Type material.

***Lectotype****Xyleborus imitans* (NMNH). ***Holotype****Xyleborus samoensis* (NHMUK), ***paratype*** (FRI).

##### New records.

India: Arunachal Pradesh, Etalin vicinity, 28°36'56"N, 95°53'21"E, 700 m, 12–25.v.2012, L. Dembický (ZFMK, 2). Assam, Bhalukpong, 27°02'N, 92°35'E, 150 m, 26.v–3.vi.2006, L. Dombický (NHMUK, 1). Meghalaya, Nokrek N.P., 3 km S Darbokgiri, 25°27'N, 90°19'E, 1400 m, 26.iv.1999, Dombický, Pacholátko (RABC, 1). Laos: Vientiane, Ban Van Eue, 31.xi.1965, native collector (BPBM, 1); same as previous except 30.xi.1966 (BPBM, 1).

##### Diagnosis.

2.2–2.6 mm long (mean = 2.36 mm; n = 5); 2.75–3.25× as long as wide. This species is distinguished by declivital interstriae parallel, granulate, granules uniform in size; small body size, elongate form; declivital striae 1 not impressed; elytral apex entire; and dark brown to black color.

##### Similar species.

*Euwallacea similis*, *Planiculus* spp.

##### Distribution.

Throughout the Oriental region from the Indian subcontinent through Southeast Asia, Indonesia to New Guinea and the Western Pacific islands; tropical Africa, Madagascar and the Seychelles. Recorded in the study region from Bangladesh, India (Andaman Is, Arunachal Pradesh*, Assam*, Meghalaya*, Nicobar Is, West Bengal), Laos, Myanmar, Taiwan, Thailand, Vietnam.

##### Host plants.

Polyphagous (Browne 1961b; Schedl 1963a).

##### Remarks.

The species has some potential as a pest because of its deeply penetrating galleries and very wide host range (Browne 1961a; Schedl 1963a).

#### Euwallacea semiermis

Taxon classificationAnimaliaColeopteraCurculionidae

(Schedl, 1934)

Fig. 57G, H, L

Xyleborus semiermis Schedl, 1934c: 89.
Euwallacea semiermis (Schedl): Beaver et al. 2014: 49.

##### Type material.

***Lectotype*** (NHMW).

##### Diagnosis.

2.5–2.75 mm long (mean = 2.62 mm; n = 3); 2.78–2.89× as long as wide. This species is distinguished by its small size; short, steep, sulcate declivity armed only by one transverse row of four granules, one on interstriae 1 and three at declivital summit; pronotum from dorsal view elongate (type 7); and pronotal asperities fine, minute.

##### Similar species.

*Euwallacea minutus*.

##### Distribution.

Indonesia (Java), Thailand.

##### Host plants.

Recorded only from *Schefflera* (Araliaceae) (Kalshoven 1959b).

#### Euwallacea semirudis

Taxon classificationAnimaliaColeopteraCurculionidae

(Blandford, 1896) stat. res.

Fig. 58A, B, I

Xyleborus semirudis Blandford, 1896b: 210.
Euwallacea semirudis (Blandford): Wood 1989: 173.
Xyleborus sereinuus Eggers, 1923: 187. Synonymy: Kalshoven 1959b: 139.
Xyleborus dubius Eggers, 1923: 199. Synonymy: Kalshoven 1959b: 139.
Xyleborus hybridus Eggers, 1927b: 90. Synonymy: Kalshoven 1959b: 139.
Xyleborus interruptus Eggers, 1940: 139. Synonymy: Schedl 1958c: 151.
Xyleborus neohybridus Schedl, 1942a: 188. syn. nov.
Xyleborus longehirtus Nunberg, 1956: 209. Unnecessary new name for X. dubius Eggers, 1923: 199.

##### Type material.

***Holotype****Xyleborus semirudis* (NHMUK). ***Holotype****Xyleborus hybridus* (NHMW). ***Paratypes****Xyleborus interruptus* (NHMW, 3).

##### Diagnosis.

3.1–3.3 mm long (mean = 3.18 mm; n = 5); 2.21–2.29× as long as wide. This species is distinguished by the pronotum basic (type 2) when viewed dorsally, anterior margin appearing rounded; declivity gradual; declivital face flat, opalescent and shagreened; declivital striae impressed, strial punctures large; and declivital posterolateral margin carinate.

##### Similar species.

*Euwallacea fornicatus* species complex (*E. fornicatior*, *E. fornicatus*, *E. kuroshio*, *E. perbrevis*), *E. andamanensis*, *E. geminus*, *E. malloti*, *E. neptis*, *E. testudinatus*, *E. velatus*.

##### Distribution.

‘Borneo’, Brunei, Indonesia (Java, Mentawai Is, Sumatra), East & West Malaysia, Philippines, Sri Lanka, Thailand.

##### Host plants.

Polyphagous (Browne 1961b).

##### Remarks.

*Euwallacea semirudis* had previously been considered a synonym of the African species *E. xanthopus* Eichhoff by Wood (1989). Wood and Bright (1992: 696) noted that there were morphological differences between the two species in that the African specimens (*E. xanthopus*) were shiny and Asian specimens (*E. semirudis*) were partly or entirely dull, which strongly suggests that African and Asian populations represent different species. To test this we obtained COI sequences from South Africa sequenced by Stouthamer et al. (2017) (KU727034) and compared these to two individuals sequenced from Sabah (MN619944) and Papua New Guinea (HM064086). The African species differed from each Asian species by 14.6% and 13.7%, respectively while the Asian specimens differed by 16.3%. Typical intraspecific variation in xyleborines is under 10% (Cognato et al. 2020b). Given the combination of differences in appearance and distribution combined with large COI pairwise differences, we here recognize *E. semirudis* as a separate species from *E. xanthopus*. However, the data clearly show that *E. semirudis* is likely a species complex and additional investigation will be required to further delimit species.

**Figure 58.**
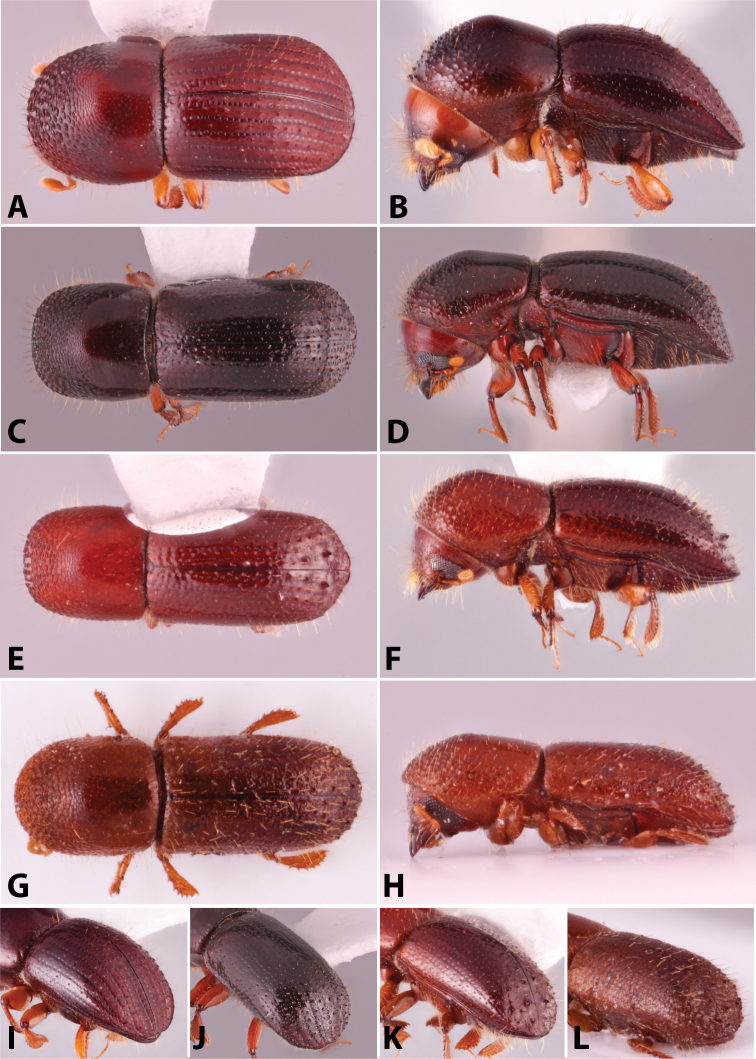
Dorsal, lateral and declivital view of *Euwallacea semirudis*, 3.1–3.3 mm (**A, B, I**), *E. sibsagaricus*, 3.4–3.9 mm (**C, D, J**), *E. similis*, 2.3–2.5 mm (**E, F, K**), and *E. subalpinus* holotype, 2.4 mm (**G, H, L**).

#### Euwallacea sibsagaricus

Taxon classificationAnimaliaColeopteraCurculionidae

(Eggers, 1930)

Fig. 58C, D, J

Xyleborus sibsagaricus Eggers, 1930: 196.
Euwallacea sibsagaricus (Eggers): Wood 1989: 173.
Xyleborus dalbergiae Eggers, 1930: 196. Synonymy: Wood 1989: 173.
Xyleborus tonkinensis Schedl, 1934a: 39. syn. nov.

##### Type material.

***Holotype****Xyleborus sibsagaricus* (FRI). ***Cotype****Xyleborus dalbergiae* (NMNH, 1). ***Holotype****Xyleborus tonkinensis* (NHMW).

##### New records.

China: Yunnan, Xishuangbanna, Jinghong City, Jinghong Farm, 21.785N, 100.790E, 677 m, 18.vii.2018, Lai S-C., Zhang L., ex *Hevea brasiliensis* (RABC, 1). India: Arunachal Pradesh, Etalin vicinity, 28°36'56"N, 95°53'21"E, 700 m, 12–25.v.2012, L. Dembický (ZFMK, 1). Meghalaya, 3 km E Tura, 25°30'N, 90°14'E, 1150 m, 4.v.1999, Dombický, Pacholátko (NHMB, 1). Vietnam: Central Tonkin, [Tuyen Quang], Chiem-Hoa, viii–ix.[no year given], H. Fruhstorfer (NHMW, 1). Tonkin, Hoa-Binh, De Cooman, 1926 (NMNH, 1); as previous except: 1940 (MNHN, 2).

##### Diagnosis.

3.4–3.9 mm long (mean = 3.58 mm; n = 5); 2.77–2.83× as long as wide. This species is distinguished by its slender form; declivital posterolateral margin costate and granulate, pronotum appearing subquadrate when viewed dorsally (type 3); protibiae outer margins distinctly triangular bearing five short broad obtuse denticles; declivital strial punctures approximately the same size on disc and declivity; declivity very steep; and declivital surface strongly shiny.

##### Similar species.

*Euwallacea luctuosus*, *E. subalpinus*, *Heteroborips tristis*.

##### Distribution.

China* (Yunnan), India (Arunachal Pradesh*, Assam, Meghalaya*, West Bengal), Indonesia (Maluku), East Malaysia, Philippines, Vietnam.

##### Host plants.

Recorded from *Ehretia* (Ehretiaceae), *Sapium* (Euphorbiaceae), *Casearia* (Salicaceae) (Beeson 1961), and *Hevea brasiliensis* (Euphorbiaceae).

##### Remarks.

Images of the *Xyleborus dalbergiae* and *X. sibsagaricus* holotypes and the holotype specimen of *X. tonkinensis* was compared to each other and found to be conspecific and *X. tonkinensis* is here placed in synonymy.

#### Euwallacea similis

Taxon classificationAnimaliaColeopteraCurculionidae

(Ferrari, 1867)

Fig. 58E, F, K

Xyleborus similis Ferrari, 1867: 23.
Wallacellus similis (Ferrari): Hulcr and Cognato 2010a: 29.
Euwallacea similis (Ferrari) Storer et al. 2015: 396.
Bostrichus ferrugineus Bohemann, 1858: 88. Preoccupied by Fabricius (1801). Synonymy: Schedl 1960a: 11.
Xyleborus parvulus Eichhoff, 1868b: 152. Synonymy: Schedl 1959: 505.
Xyleborus dilatatus Eichhoff, 1878b: 393. Synonymy: Schedl 1959: 505.
Xyleborus submarginatus Blandford, 1896b: 223. Synonymy: Eggers 1929: 48.
Xyleborus bucco Schaufuss, 1897: 212. Synonymy: Schedl 1959: 505.
Xyleborus capito Schaufuss, 1897: 215. Synonymy: Schedl 1959: 505.
Xyleborus novaguineanus Schedl, 1936b: 530. Synonymy: Wood 1989: 177.
Xyleborus dilatatulus Schedl, 1953a: 127. Synonymy: Wood 1989: 177.

##### Type material.

***Lectotype****Xyleborus bucco* (NMNH). ***Lectotype****Xyleborus capito* (NMNH).

##### New records.

China: Chongqing, Gele Mtn, 5.v.2016, Tian-Shang, Lv-Jia, ex *Broussonetia* sp. (RABC, 1). Hong Kong, Tai Po Kau, vi.2017, J. Skelton (MSUC, 1). India: Assam-Arunachal Pradesh border, Bhalukpong, 27°00'48"N, 92°39'08"E, 150 m, 1–8.v.2012, L. Dembický, ex FIT (ZFMK, 5). Laos: Bolikhamxai, Ban Nape, 18°20'N, 105°08'E, 500 m, 1–18.v.2000, P. Pachlolátoko (NHMP, 4). Khammouane, Hin Boun river, Ban Nathan, Camp de l’Agame, 17°59.645'N, 104°49.352'E, IBCFL, Operation Canopée, 7.v.2012, H.-P. Aberlenc (RABC, 2). Luang Namtha, Tong On village, 47Q 0750111, UTM 2321825, 552 m, 1.v.2005, N. Jönsson, T. Malm, B. Viklund, ex light trap (SMNH, 1). Vientiane, 10 km N Luang-Prabang, Mekhong river, 240 km N Vientiane, hills c. 250 m, poor settlem[ent], prim[ary] veget[ation] lux, iii.1993, Insomsay Somsy (MFNB, 18); as previous except: iv.1993 (MFNB, 30); Ban Van Eue, 31.xii.1965, native collector (BPBM, 2); as previous except: Vientiane city, Donchan sand dune in Mekong river, 17°57.4'N, 102°36.5'E, ~ 180 m, J. Hájek (NHMP, 1); N, 10 km N Luang Prabang, Mekong river, 240 km N. Vientiane, hilly country, sparse, settled primary vegetation, xii.1992, I. Somsy (NKME, 1). Vietnam: Bach Kan, Ba Be N.P., cabins, 255 m, 20–24.ix.2013, J.B. Heppner (FSCA, 2). [Da Lak], 10 km E of BanME Thout [*sic*] [= Buon Ma Thout], 570 m, 7.v.1960, R.E. Leech (BPBM, 1). Dong Nai, Cat Tien N.P., 11.46050, 107.37375, 379 m, 22.ii.2017, VN94, A.I. Cognato, T.A. Hoang, ex under bark; 30 cm diameter (MSUC, 91); as previous except: ecology trail, 11°26'22"N, 107°24'58"E, 120 m, 28–31.v.1999, D.C. Darling, N. Tatamic, VIET1H95-99 042, ex pan trap (SEMC, 1). Hatay, Ba Vi Nat. Pk, 455 m, 19–23.vii.2010, J.B. Heppner, Y.S. Bae (FSCA, 1). Tonkin, Hoa-Binh, 1929, A. De Cooman (MNHN, 1). Ninh Binh, Doi Vac, Cuc Phuong, 10–16.ix.2013, J.B. Heppner (FSCA, 11). Thua Thien-Hue, Bach Ma N.P., 16.22897, 107.85349, 415 m, 15.ii.2017, VN55, A.I. Cognato, T.A. Hoang, ex 5 cm diameter branch (MSUC, 1). Ninh Binh, Cuc Phuong N.P., Mac Lake, 20°15'29.0"N, 105°42'27.5"E, 155 m, 4–7.v.2009, J.B. Heppner, ex blacklight trap (FSCA, 1). Vinh Phuc, Me Linh Biodiversity Station, Dai Lai Lake, 100 m, 27–29.ix.2013, J.B. Heppner (FSCA, 3); as previous except Tam Dao (SE), 25–31.vii.2010, 985 m, J.B. Heppner (FSCA, 1). Yen Bai, Tan Huong, 21.82410, 104.89651, 30.viii.2015, Pham Thu, ex funnel trap (RJRC, 1).

##### Diagnosis.

2.3–2.5 mm long (mean = 2.42 mm; n = 5); 2.88–3.13× as long as wide. This species is distinguished by declivital interstriae 1 laterally broadened, bearing a large median tubercle and several small granules (rarely median tubercles absent); small body size and elongate form; and red brown color.

##### Similar species.

*Euwallacea piceus*, *Planiculus* spp., *Xyleborus affinis*, *X. cognatus*, *X. ferrugineus*, *X. perforans*, *X. volvulus*.

##### Distribution.

Throughout the Oriental region from the Indian subcontinent through southeast Asia and Indonesia to New Guinea, Australia, and the Pacific islands; tropical Africa, Indian Ocean islands. Recorded in the study area from Bangladesh, Cambodia, China (Chongqing*, Guangdong, Hainan, Hong Kong*, Yunnan), India (Andaman Is, Assam, Jharkhand, Karnataka, Madhya Pradesh, Nicobar Is, Sikkim, Tamil Nadu, Uttarakhand, Uttar Pradesh, West Bengal), Laos*, Myanmar, Nepal, Taiwan, Thailand, Vietnam. Introduced to the US (Rabaglia et al. 2006; Gomez et al. 2018a) and Central and South America (Wood 2007).

##### Host plants.

Strongly polyphagous (Browne 1961b; Schedl 1963a).

##### Remarks.

The biology of the species is discussed by Browne (1961a, 1968), Kalshoven (1964) and Schedl (1963a).

#### Euwallacea subalpinus sp. nov.

Taxon classificationAnimaliaColeopteraCurculionidae

http://zoobank.org/0C9C5DF6-D198-493E-846C-595B571B9896

Fig. 58G, H, L

##### Type material.

***Holotype***, female, India: Assam-Arunachal Pradesh border [Assam]: Bhalukpong, 27°00'48"N, 92°39'08"E, 150 m, 1–8.v.2012, L. Dembický, ex FIT (ZFMK).

##### Diagnosis.

2.4 mm long (n = 1); 3.0× as long as wide. This species is distinguished by its slender form; pronotum from dorsal view elongate, anterior margin rounded (type 7); outer margin of protibiae distinctly triangular, bearing five denticles, denticles not contiguous; and declivital strial punctures very large, coarse.

##### Similar species.

*Euwallacea luctuosus*, *E. sibsagaricus*.

##### Description

**(female).** 2.4 mm long (n = 1); 3.0× as long as wide. Body ferruginous. Legs and antennae light brown. ***Head***: epistoma entire, transverse, with a row of hair-like setae. Frons weakly convex to upper level of eyes; surface subshiny, alutaceous, punctate; punctures large, shallow, moderately spaced, setose; punctures bearing a long, erect hair-like seta. Eyes shallowly emarginate just above antennal insertion, upper part smaller than lower part. Antennal scape regularly thick, as long as club. Pedicel as wide as scape, shorter than funicle. Funicle 4-segmented, segment 1 shorter than pedicel. Club approximately circular, obliquely truncate, type 2; segment 1 corneous, transverse on anterior face, occupying basal 1/2, nearly covering posterior face; segment 2 narrow, corneous; segment 1 present on posterior face. ***Pronotum***: 1.26× as long as wide. In dorsal view long and rounded frontally, type 7, sides parallel in basal 3/4, rounded anteriorly; anterior margin without serrations. In lateral view elongate with disc much longer than anterior slope, type 7, disc flat, summit at apical 2/5. Anterior slope with densely spaced, low, broad asperities, becoming lower and more strongly transverse towards summit. Disc strongly shiny with sparse, minute punctures, some longer hair-like setae at margins. Lateral margins obliquely costate. Base transverse, posterior angles acutely rounded. ***Elytra***: 1.68× as long as wide, 1.34× as long as pronotum. Scutellum moderately sized, linguiform, shiny, flush with elytra, flat. Elytral base transverse, edge oblique and unarmed by granules, humeral angles rounded, parallel-sided in basal 1/2, then broadly rounded to apex. Disc flat, shiny, striae not impressed, with large, shallow punctures separated by less than one diameter of a puncture, glabrous; interstriae flat, very sparsely finely punctate, punctures 1/3 size of strial punctures, each with a long, erect seta. Declivity occupying approximately 1/3 of elytra, rounded, declivital face convex, shiny and coarsely sculptured; striae not impressed, strial punctures very large and coarse, much larger and deeper than those of disc, setose, setae short, as long as strial punctures; interstriae impunctate, interstriae 1 and 3 with three and two large tubercles, respectively, as well as several granules, interstriae 2 sparsely granulate, tubercles and granules setose, setae long, erect. Posterolateral margin costate, granulate to interstriae 7. ***Legs***: protibiae distinctly triangular, broadest at apical 1/4; posterior face smooth; apical 1/2 of outer margin with five large socketed denticles, their length much longer than basal width. Meso- and metatibiae flattened; outer margin evenly rounded with five and six large socketed denticles, respectively.

##### Etymology.

L. *sub* = under, below; *alpinus* = high mountains. In reference for the species occurrence in the foothills of the Himalayas. An adjective.

##### Distribution.

India (Assam).

##### Host plants.

Unknown.

##### Remarks.

The holotype is card mounted and ventral characters could not be examined.

#### Euwallacea testudinatus sp. nov.

Taxon classificationAnimaliaColeopteraCurculionidae

http://zoobank.org/F2B1020A-6210-4D9B-90A9-8033616322C8

Fig. 59A, B, G

##### Type material.

***Holotype***, female, China: S-Yunnan, Xishuangbanna, 23 km NW Jinghong, vic. Na Ban village (NNNR), 22°10'N, 100°39'E, 700–1000 m, v–vii.2009, L. Meng (NKME). ***Paratypes***, female, as holotype (MSUC, 1; NKME, 2; RABC, 2); as previous except: 20 km NW Jinghong, Man Dian NNNR-office, 22°07.80'N, 100°40.05'E, 740 m, LFF, 24.v.2008, A. Weigel (NKME, 1).

##### Diagnosis.

2.8–2.9 mm long (mean = 2.86 mm; n = 5); 2.24–2.33× as long as wide. This species is distinguished by the pronotum subquadrate (near type 3) in dorsal view, but only 0.89–0.97× longer than wide, and more strongly truncate anteriorly; elytra with a weak basal carina, sides parallel in basal 2/3, then gradually incurved to broadly rounded apex; declivity beginning after basal 1/4, convex, weakly flattened across interstriae 1–2 in apical 1/4, declivital strial punctures shallow, very coarse, striae 2 very weakly impressed, interstriae granulate, interstriae 2 with two or three larger denticles in apical 1/4, declivital posterolateral margin carinate, never granulate; and protibiae with 9–11 socketed denticles.

##### Similar species.

*Euwallacea fornicatus* species complex (*E. fornicatior*, *E. fornicatus*, *E. kuroshio*, *E. perbrevis*), *E. andamanensis*, *E. geminus*, *E. malloti*, *E. neptis*, *E. semirudis*, *E. velatus*.

##### Description

**(female).** 2.8–2.9 mm long (mean = 2.86 mm; n = 5); 2.24–2.33× as long as wide. Body dark brown. Legs and antennae brown. ***Head***: epistoma entire, transverse, with a row of hair-like setae. Frons weakly convex to upper level of eyes; surface strongly shiny, median 2/3 smooth, impunctate, lateral 1/3 sparsely and finely punctate, setose; punctures bearing a long, erect hair-like seta. Eyes shallowly emarginate just above antennal insertion, upper part smaller than lower part. Submentum narrow, triangular, slightly impressed. Antennal scape regularly thick, as long as club. Pedicel as wide as scape, shorter than funicle. Funicle 4-segmented, segment 1 as long as pedicel. Club longer than wide, flat, type 3; segment 1 corneous, transverse on anterior face, occupying basal 2/5; segment 2 narrow, corneous; segments 1–3 present on posterior face. ***Pronotum***: 0.89–0.97× as long as wide. In dorsal view subquadrate and parallel-sided, type 3, sides parallel in basal 4/5, narrowly rounded anteriorly; anterior margin transverse without serrations. In lateral view tall, type 2, disc flat, summit at midpoint. Anterior slope with densely spaced, large coarse asperities, becoming lower and more strongly transverse towards summit. Disc shiny, alutaceous with sparse, minute punctures, some longer hair-like setae at margins. Lateral margins obliquely costate. Base transverse, posterior angles acutely rounded. ***Elytra***: 1.46× as long as wide, 1.5× as long as pronotum. Scutellum broad, moderately sized, linguiform, flush with elytra, flat, shiny. Elytral base transverse, edge weakly carinate and unarmed by granules, humeral angles rounded, parallel-sided in basal 2/3, then gradually incurved to broadly rounded apex. Disc convex, shiny, striae not impressed, with large, shallow punctures separated by one diameter of a puncture, glabrous; interstriae flat, very sparsely finely punctate, punctures 1/3 size of strial punctures, each with a long, erect seta. Declivity occupying approximately 3/4 of elytra, gradual, face convex, shiny, weakly flattened across interstriae 1–2 in apical 1/4; declivital strial punctures shallow, very coarse, strial punctures as large as those of disc, striae 2 very weakly impressed; interstriae impunctate, granulate, interstriae 1 with one denticle, interstriae 2–4 with two or three larger denticles in apical 1/4. Posterolateral margin carinate, unarmed to interstriae 7. ***Legs***: procoxae contiguous; prosternal coxal piece short, conical. Protibiae semi-circular, with rounded outer margin; posterior face smooth; apical 1/2 of outer margin with 9–11 large socketed denticles, their length longer than basal width. Meso- and metatibiae flattened; outer margin evenly rounded with 11 small socketed denticles.

##### Etymology.

L. *testudinatus* = vaulted like a tortoise shell. Named in reference to its domed shaped elytra. An adjective.

##### Distribution.

China (Yunnan).

##### Host plants.

Unknown.

**Figure 59.**
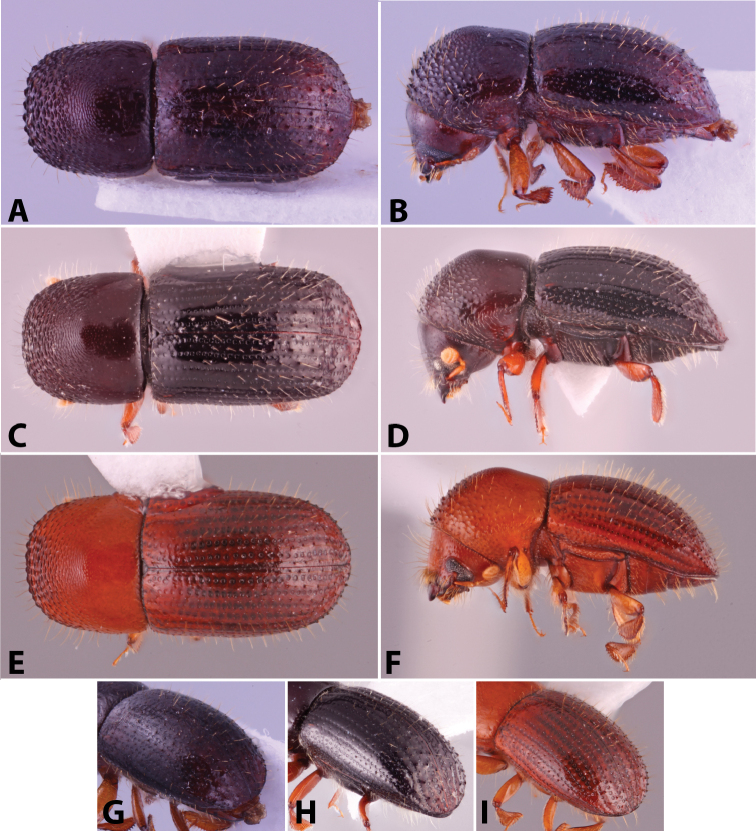
Dorsal, lateral and declivital view of *Euwallacea testudinatus* paratype, 2.8–2.9 mm (**A, B, G**), *E. validus*, 3.9–4.1 mm (**C, D, H**), and *E. velatus*, 2.8–3.6 mm (**E, F, I**).

#### Euwallacea validus

Taxon classificationAnimaliaColeopteraCurculionidae

(Eichhoff, 1876)

Fig. 59C, D, H

Xyleborus validus Eichhoff, 1876a: 202.
Euwallacea validus (Eichhoff): Wood and Bright 1992: 694.

##### Type material.

***Syntype*** (IRSNB).

##### New records.

China: Chongqing, Youyang, 11.vii.2016, Tian-Shang (RABC, 1). W. Hupeh [= Hubei], Lichuan Distr., Suisapa, 1000 m, 29.vii.[19]48, Gressitt & Djou, ex *Metasequoia glyptostroboides* (CASC, 4). Fujian, Jiangle, Longqishan Mt., 700 m, 7.viii.1991, Xiaochun Zhang coll, ex pine (BPBM, 1); as previous except: 26[?].v.1991, Hong Liu (BPBM, 1).

##### Diagnosis.

3.9–4.1 mm long (mean = 4.0 mm; n = 5); 2.5–2.73× as long as wide. This species is distinguished by the pronotum appearing subquadrate (type 3) from dorsal view; outer margin of protibiae round, declivital interstriae 1 granulate; declivital posterolateral margin carinate never granulate; and moderately large size.

It can be further separated from the strongly morphologically similar species *E. interjectus* by the declivity steeply sloped; declivital strial punctures deep giving the declivity a rugged appearance; and tubercles mostly absent from the apical 1/2 of interstriae 2.

##### Similar species.

*Euwallacea funereus*, *E. interjectus*, *E. velatus*.

##### Distribution.

China (Anhui, Chongqing*, Fujian, Hubei*, Yunnan), Japan, Nepal, South Korea, Taiwan, Vietnam. Introduced and established in USA (Wood 1975; Cognato et al. 2015; Gomez et al. 2018a).

##### Host plants.

Polyphagous attacking both gymnosperm and angiosperm trees (Wood and Bright 1992).

#### Euwallacea velatus

Taxon classificationAnimaliaColeopteraCurculionidae

(Sampson, 1913)

Fig. 59E, F, I

Xyleborus velatus Sampson, 1913: 443.
Euwallacea velatus (Sampson): Wood 1989: 173.
Xyleborus rudis Eggers, 1930: 192. syn. nov.
Xyleborus assamensis Eggers, 1930: 195. Synonymy: Wood 1989: 173.
Xyleborus asperipennis Eggers, 1934b: 27. Unnecessary new name for X. assamensis Eggers, 1930 nec Stebbing, 1909. Synonymy: Wood 1989: 173.

##### Type material.

***Holotype****Xyleborus velatus* (NHMUK), ***paratype*** (NHMW). ***Holotype****Xyleborus assamensis* (FRI), ***paratype*** (NMNH, 1; NHMW, 1). ***Paratype****Xyleborus rudis* (NHMW).

##### New records.

China: Yunnan, Xishuangbanna, Sanchahe Nat. Res., 22°09.784'N, 100°52.256'E, 2186 m, 29–30.v.2008, A.I. Cognato (MSUC, 1). India: Arunachal Pradesh, Etalin vicinity, 28°36'56"N, 95°53'21"E, 700 m, 12–25.v.2012, L. Dembický (ZFMK, 1). Vietnam: Ninh Binh, Cuc Phuong N.P., 20.33296, 105.61259, 7.iii.2018, 279 m, A.I. Cognato, S.M. Smith, VN 140, ex 3 cm diameter branch (MSUC, 4).

##### Diagnosis.

2.8–3.6 mm long (mean = 3.28 mm; n = 5); 2.31–2.57× as long as wide. This species is distinguished by the granulate posterolateral costa, pronotum appearing subquadrate (type 3) when viewed dorsally; protibiae outer margins rounded, bearing nine small socketed denticles, sockets small; declivital posterolateral margin costate and granulate; declivity rounded and convex; and elytral bases rounded, never weakly costate, or granulate.

##### Similar species.

*Euwallacea fornicatus* species complex (*E. fornicatior*, *E. fornicatus*, *E. kuroshio*, *E. perbrevis*), *E. andamanensis*, *E. geminus*, *E. interjectus*, *E. malloti*, *E. neptis*, *E. semirudis*, *E. testudinatus*, *E. validus*.

##### Distribution.

China (Xizang, Yunnan*), India (Andaman Is, Arunachal Pradesh*, Assam, Meghalaya, Nagaland, Sikkim, Uttar Pradesh, West Bengal), Laos, Myanmar, Nepal, Thailand, Vietnam*.

##### Host plants.

Polyphagous (Maiti and Saha 2004).

##### Remarks.

*Xyleborus rudis* was considered a synonym of the African species *E. xanthopus* by Wood (1989). Examination of the holotype and paratype revealed that this species is clearly conspecific with *E. velatus* and bears little resemblance to either *E. xanthopus* or its close Asian relative *E. semirudis* (Fig. 58A, B, I). *Xyleborus rudis* is here placed in synonymy with *E. velatus*.

### *Fortiborus* Hulcr & Cognato, 2010

#### Fortiborus

Taxon classificationAnimaliaColeopteraCurculionidae

Hulcr & Cognato, 2010

Fortiborus Hulcr & Cognato, 2010a: 17.

##### Type species.

*Phloeosinus major* Stebbing, 1909; original designation.

##### Diagnosis.

*Fortiborus* species are among the largest xyleborines in Southeast Asia (4.8–6.6 mm and 2.52–3.06× as long as wide). *Fortiborus* is distinguished by the robust pronotum; declivity flattened and broadened laterally, apex angulate; anterior edge of pronotum extended anteriad, bearing a distinct row of serrations; antennal club distinctly pubescent, type 4; eyes very large, deeply emarginate; scutellum flat, flush with elytra; procoxae contiguous; and mycangial tufts absent.

*Fortiborus* is similar to some large *Euwallacea* species except the margin of segment 1 of antennal club is concave and recurved; anterior edge of pronotum produced anteriad, bearing row of serrations; and protibiae rounded, with seven or more denticles.

##### Similar genera.

*Euwallacea*, *Xyleborus*.

##### Distribution.

Found throughout Southeast Asia and Oceania.

##### Gallery system.

The galleries are regularly branched in one transverse plane and are without brood chambers (Browne 1961b).

##### Remarks.

The species of this genus are all closely associated with Dipterocarpaceae and are not definitely known to breed in other families of trees.

#### Key to *Fortiborus* species (females only)

**Table:** 

1	Larger, over 6.0 mm; elytral discal interstriae impunctate; boundary between disc and declivity distinct (Fig. 60E); elytral tubercles large, distinct	*** pseudopilifer ***
–	Smaller, under 6.0 mm; elytral discal interstriae punctate; boundary between disc and declivity indistinct (Fig. 60C); elytral tubercles small, granulate	**2**
2	Declivity evenly rounded, posterolateral costa of the elytra uniformly thick to its dorsal end on interstriae 7 (Fig. 60A, G); declivity appearing very broad and rounded in dorsal view; interstriae 2 punctures biseriate on disc, uniseriate on declivity; smaller, 4.8–5.1 mm	*** macropterus ***
–	Declivity steeper, posterolateral costa of the elytra thickens and becomes more prominent at its dorsal end on interstriae 7 (Fig. 60C, H); declivity appears very broad and flat in dorsal view; interstriae 2 punctures confused; larger, 5.2–6.0 mm	*** major ***

#### Fortiborus macropterus

Taxon classificationAnimaliaColeopteraCurculionidae

(Schedl, 1935)

Fig. 60A, B, G

Xyleborus macropterus Schedl, 1935b: 271.
Fortiborus macropterus (Schedl): Beaver et al. 2014: 51.

##### Type material.

***Lectotype*** (NHMW).

##### Diagnosis.

4.8–5.1 mm long (mean = 4.93 mm; n = 3); 2.55–3.06× as long as wide. This species is distinguished by the small size; conspicuously angulate elytral apex; posterolateral declivital costa weakly elevated and asperate; declivity appearing very broad and rounded; tubercles on declivital interstriae uniformly sized, present from base to apex; and discal interstrial punctures uniseriate.

##### Similar species.

*Fortiborus major*.

##### Distribution.

‘Borneo’, Indonesia (Sumatra), East & West Malaysia, Philippines, Thailand.

##### Host plants.

Apart from a single record from Sapotaceae, recorded only from various genera of Dipterocarpaceae (*Balanocarpus*, *Dipterocarpus*, *Dryobalanops*, *Hopea*, *Shorea*, *Vatica*) (Browne 1961b; Ohno 1990).

**Figure 60.**
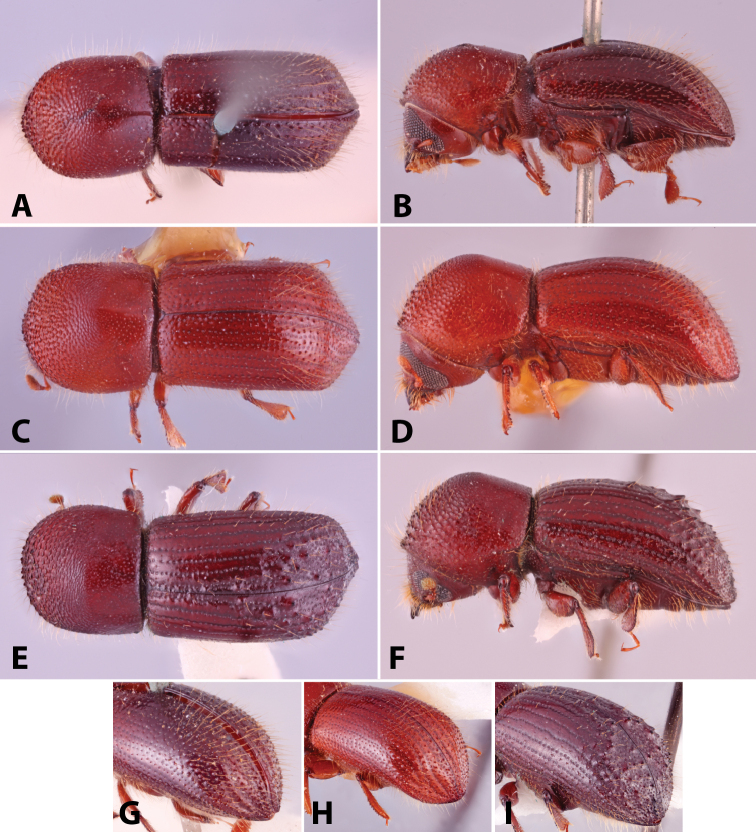
Dorsal, lateral and declivital view of *Fortiborus macropterus* lectotype, 4.8–5.1 mm (**A, B, G**), *F. major*, 5.2–6.0 mm (**C, D, H**), and *F. pseudopilifer*, 6.6 mm (**E, F, I**).

#### Fortiborus major

Taxon classificationAnimaliaColeopteraCurculionidae

(Stebbing, 1909)

Fig. 60C, D, H

Phloeosinus major Stebbing, 1909: 19.
Xyleborus major (Stebbing): Stebbing 1914: 590.
Notoxyleborus major (Stebbing): Maiti and Saha 1986: 100.
Fortiborus major (Stebbing): Hulcr and Cognato 2010a: 17.
Xyleborus siclus Schedl, 1936d: 26. Synonymy: Hulcr and Cognato 2010a: 18.

##### Type material.

***Holotype****Phloeosinus major* (FRI). ***Allotype****Xyleborus siclus* (NHMUK).

##### New records.

India: Andaman Islands, N. Andaman, 3.xi.1930, C.F.C. Beeson, *Dipterocarpus turbinatus* (NMNH, 1). [West] Bengal, Chilapata forest, Buxa, 1.ix.1915, C.F.C. Beeson, *Shorea robusta* bark (NMNH, 1).

##### Diagnosis.

5.2–6.0 mm long (mean = 5.58 mm; n = 5); 2.52–2.71× as long as wide. This species is distinguished by the moderate size; conspicuously angulate elytral apex; posterolateral declivital costa conspicuously elevated and asperate, making the declivity appear very broad and flat; declivital interstrial granules uniformly sized, present from base to apex; and discal interstrial punctures confused.

##### Similar species.

*Fortiborus macropterus*.

##### Distribution.

‘Borneo’, India (Andaman Is*, Assam, West Bengal*), Indonesia (Mentawai Is, Sumatra), New Guinea, Thailand.

##### Host plants.

This species is also closely associated with Dipterocarpaceae (Browne 1961b). There are records of single specimens taken from three other families (Ohno 1990), but they may not have been breeding.

#### Fortiborus pseudopilifer

Taxon classificationAnimaliaColeopteraCurculionidae

(Schedl, 1936)

Fig. 60E, F, I

Xyleborus pseudopilifer Schedl, 1936a: 11.
Fortiborus pseudopilifer (Schedl): Smith et al. 2018c: 841.

##### Type material.

***Paratype*** (NHMUK).

##### Diagnosis.

6.6 mm long, (n = 1); 2.64× as long as wide. This species is distinguished by the large size; unique declivital sculpturing marked by a distinct boundary between disc and declivity; all interstriae bearing a series of 1–3 moderate to large tubercles at declivital summit and all interstriae bearing 3–8 irregularly spaced small to moderately sized tubercles; declivital punctures strongly confused; and discal interstriae impunctate.

##### Similar species.

*Euwallacea wallacei*, from which it is distinguished by its very large eyes.

##### Distribution.

‘Borneo’, Indonesia (Sumatra), East & West Malaysia, Philippines, Thailand, Vietnam.

##### Host plants.

Unknown, but probably associated with Dipterocarpaceae like other *Fortiborus* species.

### *Fraudatrix* Cognato, Smith & Beaver, 2020

#### Fraudatrix

Taxon classificationAnimaliaColeopteraCurculionidae

Cognato, Smith & Beaver, 2020

Fraudatrix Cognato, Smith & Beaver, 2020 (in Cognato et al. 2020a): 544.

##### Type species.

*Xyleborus melas* Eggers, 1927b; original designation.

##### Diagnosis.

1.75–2.5 mm and 2.86–3.33× as long as wide. *Fraudatrix* is distinguished by the following combination of characters: antennal funicle 2-segmented, antennal club type 2 with one suture visible on the posterior face; protibiae obliquely triangular with six or fewer denticles on outer margin, posterior face flattened and unarmed; scutellum small, flush with elytral surface; mycangial tufts absent, elytra attenuate and pronotal disc longer than anterior slope (Cognato et al. 2020a).

*Fraudatrix* most closely resembles *Cryptoxyleborus* and *Tricosa* with which it shares an attenuate appearance and small size. It is distinguished from *Cryptoxyleborus* by the following diagnostic characters (*Fraudatrix* given first): scutellum visible vs. scutellum not apparent, antennal club truncate and type 2 vs. flattened and type 4, antennal funicle 2-segmented vs. 3 or 4-segmented, no more than one suture visible on the posterior face vs. three sutures visible. *Fraudatrix* is also similar to *Stictodex* with which it shares a type 2 antennal club and obliquely triangular protibia. *Stictodex* is easily distinguished from *Fraudatrix* by the following combination of characters (*Stictodex* given first): larger size and stouter form (2.4–3.3 mm long; 2.54–2.89× as long as wide), antennal club very broad, protibiae with 6–8 denticles on outer margin and posterior face inflated and granulate, elytra with first and second interstriae divergent, broadest at elytral summit, and declivity truncate or broadly rounded (Cognato et al. 2020a).

##### Similar genera.

*Cryptoxyleborus*, *Tricosa*.

##### Distribution.

Throughout the Oriental and Australian regions.

##### Gallery system.

Only the gallery of *F. cuneiformis* has been described. The system has branched tunnels with small brood chambers in the longitudinal plane (Browne 1961b).

##### Remarks.

*Fraudatrix* species appear to be quite rare. Species are known from very few specimens.

#### Key to *Fraudatrix* species (females only)^1^

**Table:** 

1	Pronotum anterior margin subquadrate	* simplex *
–	Pronotum anterior margin rounded	2
2	Declivital strial punctures distinct, nearly as large as interstrial granules	* melas *
–	Declivital strial punctures indistinct, much smaller than interstrial granules	* cuneiformis *

#### Fraudatrix cuneiformis

Taxon classificationAnimaliaColeopteraCurculionidae

(Schedl, 1958)

Fig. 61A, B, G

Xyleborus cuneiformis Schedl, 1958b: 104.
Fraudatrix cuneiformis (Schedl): Cognato et al. 2020a: 545.

##### Type material.

***Lectotype*** (NHMW).

##### Diagnosis.

1.9–2.15 mm long (mean = 2.02 mm; n = 5); 2.86–3.07× as long as wide. This species is distinguished by the anterior margin of the pronotum rounded; declivital strial punctures indistinct; interstrial granules large, distinct; elytral apex narrowly attenuate; and stouter form (Cognato et al. 2020a).

##### Similar species.

*Coptodryas mus*, *Tricosa metacuneolus*.

##### Distribution.

Brunei, East & West Malaysia, Singapore, Taiwan.

##### Host plants.

Recorded only from two species of *Shorea* (Dipterocarpaceae) (Schedl 1958b; Cognato et al. 2020a).

##### Remarks.

The gallery system has branched tunnels with small brood chambers in the longitudinal plane (Browne 1961b).

**Figure 61.**
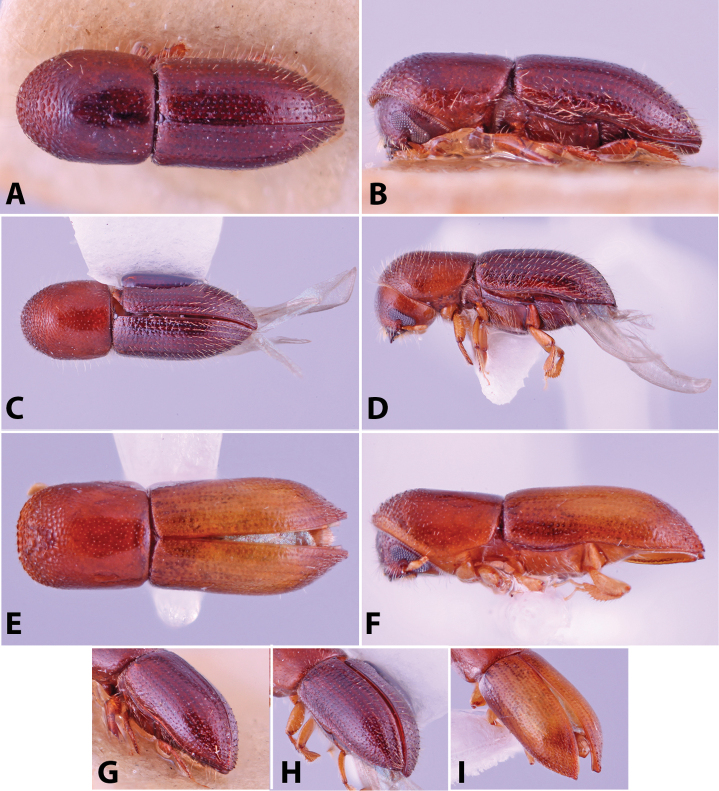
Dorsal, lateral and declivital view of *Fraudatrix cuneiformis* lectotype, 1.9–2.15 mm (**A, B, G**), *F. melas*, 2.3 mm (**C, D, H**), and *F. simplex*, 1.75–2.0 (**E, F, I**).

#### Fraudatrix melas

Taxon classificationAnimaliaColeopteraCurculionidae

(Eggers, 1927)

Fig. 61C, D, H

Xyleborus melas Eggers, 1927b
Fraudatrix melas (Eggers): Cognato et al. 2020a: 546.

##### Type material.

*Lectotype* (NMNH).

##### Diagnosis.

2.3 mm long (n = 2); 3.29× as long as wide. This species is distinguished by the anterior margin of the pronotum rounded; declivital strial punctures distinct, nearly as large as interstrial granules, each bearing a short recumbent seta; and more slender form (Cognato et al. 2020a).

##### Similar species.

*Tricosa jacula*, *T. metacuneolus*.

##### Distribution.

China (Hong Kong), Philippines.

##### Host plants.

Unknown.

#### Fraudatrix simplex

Taxon classificationAnimaliaColeopteraCurculionidae

(Browne, 1949)

Fig. 61E, F, I

Cryptoxyleborus simplex Browne, 1949: 902.
Webbia simplex (Browne): Wood and Bright 1992: 833.
Cryptoxyleborus simplex Browne: Bright and Skidmore 1997: 5, 176.
Fraudatrix simplex (Browne): Cognato et al. 2020a: 546.

##### Type material.

*Holotype* (NHMUK).

##### Diagnosis.

1.75–2.0 mm long (mean = 1.92 mm; n = 5); 3.08–3.33× as long as wide. This species is distinguished by the anterior margin of the pronotum subquadrate; short semi-recumbent interstrial setae; and minute size (Cognato et al. 2020a).

##### Similar species.

*Cryptoxyleborus confusus*, *C. percuneolus*.

##### Distribution.

Brunei, Indonesia (Sumatra), East & West Malaysia, Thailand.

##### Host plants.

Recorded from *Dipterocarpus*, *Dryobalanops*, *Hopea*, *Shorea* (Dipterocarpaceae) (Beaver and Hulcr 2008).

##### Remarks.

Browne (1961b) notes that brood size can exceed 50.

### *Hadrodemius* Wood, 1980

#### Hadrodemius

Taxon classificationAnimaliaColeopteraCurculionidae

Wood, 1980

Hadrodemius Wood, 1980: 94.

##### Type species.

*Xyleborus globus* Blandford, 1896b; original designation.

##### Diagnosis.

*Hadrodemius* species are distinguished by their large size, 4.9–7.2 mm and stout (less than 2× as long as wide) and hairy appearance; pronotal base and scutellar area ornamented with a dense tuft of hair associated with mesonotal mycangium; scutellum visible only on the basal slope of elytral bases; procoxae contiguous; and the pronotal lateral margins rounded.

##### Similar genera.

*Hadrodemius* is closely related to *Anisandrus*, *Cnestus*, and *Xylosandrus*, all of which possess a mesonotal mycangium and the associated dense tuft of hair-like setae at the scutellar area and pronotal base (Gohli et al. 2017; Johnson et al. 2018). These three genera is distinguished from *Hadrodemius* by their normal scutellum that is flush with the dorsal surface of the elytra rather than just visible only on the basal slope of the elytral bases.

##### Distribution.

*Hadrodemius* occurs in tropical areas from India in the West, through the Oriental region to New Guinea and the Solomon Islands in the East.

##### Gallery system.

Usually constructed in small stems from 1.5 – 5.0 cm diameter, it comprises a circumferential entrance gallery leading to one to several longitudinal galleries (Beaver 2010).

##### Remarks.

Further details of the biology are given by Browne (1961b), Kalshoven (1959b) and Beaver (2010).

#### Key to *Hadrodemius* species (females only)^2^

**Table:** 

1	Declivity strongly impressed, sides of impression raised and bearing tubercles or rugosities; elytral impression matte or nearly so, with fairly sparse long hair-like setae or short hair-like setae only; 6.0 – 7.2 mm	* pseudocomans *
–	Declivity weakly impressed, flat or weakly convex; if weakly impressed, sides of impression without tubercles or rugosities, although minute granules are often present, and whole declivity with dense, long, fine hair-like setae; 4.9–6.3 mm.	2
2	Declivity plano-convex, nitid, striae 1 not impressed, strial punctures less distinct, interstrial punctures finer and more closely placed, declivital face more densely hairy; elytral vestiture usually yellowish or golden; 4.9–5.8 mm	* globus *
–	Declivity plano-concave from suture to interstriae 3, nitid to matte, striae 1 at least weakly impressed, strial punctures more distinct, interstrial punctures coarser and less closely placed, declivital face less densely hairy; elytral vestiture dark brown or black; 5.0–6.3 mm	* comans *

#### Hadrodemius comans

Taxon classificationAnimaliaColeopteraCurculionidae

(Sampson, 1919)

Fig. 62A, B, G

Xyleborus comans Sampson, 1919: 109.
Hadrodemius comans (Sampson): Wood and Bright 1992: 819.
Xyleborus amorphus Eggers, 1926: 147. Synonymy: Beaver 2010: 54.
Xyleborus metacomans Eggers, 1930: 199. Synonymy: Beaver 2010: 54.

##### Type material.

*Syntype Xyleborus comans* (NHMUK). *Syntypes Xyleborus amorphus* (NHMW).

##### New records.

China: Guangdong, v.2014, Jianguo Wang (RJRC, 1); as previous except: xii.2014 (RJRC, 3). Hong Kong, Tai Po Kau, vi.2017, J. Skelton (MSUC, 1). S Yunnan, Xishuangbanna, 23 km NW Jinghong, vic. Na Ban village (NNNR), 22°10'N, 100°39'E, 700–1000 m, v–vii.2009, L. Meng (NKME, 3; RABC, 2); as previous except: 28 km NW Jinghong, vic. An Ma Xi Zhan (NNNR), 22°12'N, 100°38'E, 700 m, forest, EKL, 28.vi.2008, A. Weigel (NKME, 1); as previous except: 5.iv.2009, L. Meng (RABC, 1).

##### Diagnosis.

5.0–6.3 mm long (mean = 6.02 mm; n = 5); 1.73–1.88× as long as wide. This species is distinguished by the declivity plano-concave from suture to interstriae 3, striae 1 at least weakly impressed; entire elytra densely setose with declivital face less densely hairy and dark brown to black vestiture.

This species is similar to *H. globus* and is distinguished by the strial punctures distinct, interstrial punctures coarser and less closely placed, and vestiture darker.

##### Similar species.

*Hadrodemius globus*.

##### Distribution.

Recorded in the study region from China (Fujian, Guangdong*, Guangxi, Hainan, Hong Kong*, Hunan, Jiangxi, Sichuan, Xizang, Yunnan*, Zhejiang), India (Assam, West Bengal), Laos, Myanmar, Taiwan, Thailand, Vietnam. It also occurs in Malaysia and Indonesia West of Wallace’s line.

##### Host plants.

Polyphagous (Beaver 2010).

**Figure 62.**
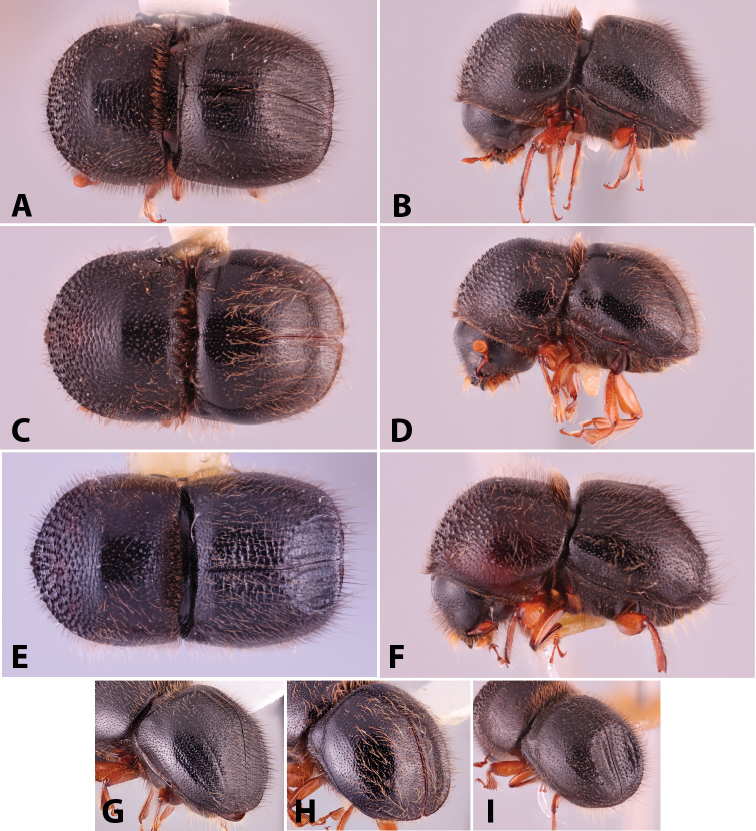
Dorsal, lateral and declivital view of *Hadrodemius comans*, 5.0–6.3 mm (**A, B, G**), *H. globus*, 4.9–5.8 mm (**C, D, H**), and *H. pseudocomans*, 6.0–7.2 mm (**E, F, I**).

#### Hadrodemius globus

Taxon classificationAnimaliaColeopteraCurculionidae

(Blandford, 1896)

Fig. 62C, D, H

Xyleborus globus Blandford, 1896b: 208.
Hadrodemius globus (Blandford): Wood 1980: 94.
Xyleborus ursus Eggers, 1923: 173. Synonymy: Browne 1961b: 111.
Xyleborus ursus fuscus Eggers, 1923: 174. Synonymy: Kalshoven 1959b: 163.
Xyleborus tomentosus Eggers, 1939a: 10. Synonymy: Beaver 2010: 54.

##### Type material.

*Holotype Xyleborus globus* (NHMUK). *Holotype Xyleborus tomentosus* (SMNH).

##### Diagnosis.

4.9–5.8 mm long (mean = 5.5 mm; n = 5); 1.77–1.83× as long as wide. This species is distinguished by the declivity plano-convex; striae 1 not impressed; and entire elytra densely setose with usually yellowish or golden vestiture.

This species is similar to *H. comans* and is distinguished by the strial punctures less distinct, interstrial punctures finer and more closely placed, declivital face more densely hairy, and vestiture lighter in color.

##### Similar species.

*Hadrodemius comans*.

##### Distribution.

Recorded in the study region from India (Kerala), Laos, Myanmar, Taiwan, Thailand, Vietnam. It also occurs in Malaysia and the Philippines, through Indonesia to New Guinea and the Solomon Islands.

##### Host plants.

Polyphagous (Beaver 2010).

#### Hadrodemius pseudocomans

Taxon classificationAnimaliaColeopteraCurculionidae

(Eggers, 1930)

Fig. 62E, F, I

Xyleborus pseudocomans Eggers, 1930: 187.
Hadrodemius pseudocomans (Eggers): Wood and Bright 1992: 819.
Xyleborus artecomans Schedl, 1953c: 24. Synonymy: Beaver 2010: 55.

##### Type material.

*Holotype Xyleborus pseudocomans* (FRI), *paratype* (NMNH, 1). *Lectotype Xyleborus artecomans* (ZMFK), *paralectotype* (ZMFK, 1).

##### New records.

China: Chongqing, Chengkou, 16.vii.2016, Tian-Shang (RABC, 1). Guangdong, iii.2014, Jianguo Wang (RJRC, 1). Guangxi A. R., Jiangidi, 25°55.6'N, 110°14.8'E, 365 m, terraced fields surrounded with shrubs and bamboo forest, 12.iv.2013, M. Ficáček, J. Hájek, J. Růžička (MNHP, 1). Hainan, Jianfengling Mt., 600 m, 26.iii.1984, Shimei Song (NMNH, 1). Jiangxi, Jinggang Shan, Jingzhushan Zhufeng, 26°31.0'N, 114°05.9'E, 640 m, stream valley, 25.iv.2011, M. Ficáček, J. Hájek (MNHP, 1). Laos: NE, Hua Phan, Ban Saluei, Phou Pan (Mt.), 20°12'N, 104°01'E, 1300–1900 m, 7.iv–25.v.2010, C. Holzschuh (NHMUK, 2); as previous except: 27.iv–1.vi.2011 (RABC, 1).

##### Diagnosis.

6.0–7.2 mm long (mean = 6.86 mm; n = 5); 1.8–1.9× as long as wide. This species is distinguished by its larger size; declivity strongly impressed, sides of impression raised and bearing tubercles or rugosities; and elytral impression bearing fairly sparse, long hairs or short hairs only.

This species is most similar to *H. comans* from which it is distinguished by the strongly impressed declivity rather than the declivity plano-concave from suture to interstriae 3.

##### Similar species.

*Hadrodemius comans*.

##### Distribution.

Brunei, China (Chongqing*, Fujian, Guangdong*, Guangxi*, Hainan, Jiangxi*, Xizang, Yunnan), India (Assam, West Bengal), Laos*, Myanmar, Thailand.

##### Host plants.

Polyphagous (Beaver 2010).

### *Heteroborips* Reitter, 1913

#### Heteroborips

Taxon classificationAnimaliaColeopteraCurculionidae

Reitter, 1913

Heteroborips Reitter, 1913: 79.

##### Type species.

*Bostrichus cryptographus* Ratzeburg, 1837; monotypy.

##### Diagnosis.

2.5–4.0 mm, 2.2–3.25× as long as wide. In this region *Heteroborips* is distinguished by the distinctive elytral mycangium appearing as a distinctly impressed area immediately adjacent to the scutellum on each elytron.

##### Similar genera.

*Tricosa*, *Xyleborinus*, *Xyleborus*.

##### Distribution.

Distributed throughout Europe and temperate Asia including the Himalayas. One species is introduced and established in USA (Hoebeke and Rabaglia 2008).

##### Gallery system.

The gallery system of *Heteroborips* is unusual among xyleborines and lies wholly between the bark and wood (Mandelshtam et al. 2019).

##### Remarks.

The genus has been recently reviewed by Mandelshtam et al. (2019).

#### Key to *Heteroborips* species (females only)

**Table:** 

1	Pronotum subquadrate (type 3) in dorsal view; apical margin of elytral mycangia distinctly raised; larger, 3.9–4.0 mm	*** tristis ***
–	Pronotum conical (type 0) in dorsal view; elytral mycangia flat, margins never raised; smaller, 2.2–3.5 mm	**2**
2	Declivity steeply sloping, occupying apical 1/4 of elytra	*** seriatus ***
–	Declivity gradually sloping, occupying at least 1/2 of elytra	**3**
3	Declivity occupying 1/2 of elytra; elytra tapering after basal 3/4 to a broadly rounded apex; smaller, 2.2–2.4 mm	***indicus* sp. nov.**
–	Declivity occupying 2/3 of elytra; elytra tapering after basal 1/3 to an angularly rounded apex; larger, 3.4–3.5 mm	***fastigatus* sp. nov.**

#### Heteroborips fastigatus sp. nov.

Taxon classificationAnimaliaColeopteraCurculionidae

http://zoobank.org/19028845-1889-40AA-BDD3-AE62EEDDE45C

Fig. 63A, B, I

##### Type material.

***Holotype***, female, India: NE, Meghalaya, Nokrak N.P., 3 km S Darbokgiri, 25°27'N, 90°19'E, 1400 m, 26.iv.1999, Dombický & Pacholátko (NHMB). ***Paratype***, female, Nepal: Annapurna Region, West Mardi Himal, Modi Khola Tal, oberh. Himalpani, 1420–1480 m, 16.v.2001, Hirthe (RABC).

##### Diagnosis.

3.4–3.5 mm long (mean = 3.45 mm; n = 2); 2.7–2.8× as long as wide. This species is distinguished by the tapering, gradually sloping form of the elytra; pronotum conical (type 0) from dorsal view, with rounded anterior margin; elytra tapering after basal 1/3 to an angularly rounded apex; declivity beginning after basal 1/4, gradually, evenly sloping to apex; declivital interstriae weakly outwardly curved in apical 1/4; interstriae granulate only in apical 1/4; and posterolateral margin weakly raised to interstriae 7, not carinate or granulate.

##### Similar species.

*Heteroborips indicus*.

##### Description

**(female).** 3.4–3.5 mm long (mean = 3.45 mm; n = 2); 2.7–2.8× as long as wide. Body, head and legs dark brown. Antennae light brown. ***Head***: epistoma entire, transverse, with a row of hair-like setae. Frons weakly convex to upper level of eyes; surface shiny, median 1/3 smooth, impunctate, lateral 2/3 densely and coarsely punctate, setose; puncture bearing a long, erect hair-like seta. Eyes very shallowly emarginate just above antennal insertion, upper part smaller than lower part. Antennal scape long and slender, as long as club. Pedicel as wide as scape, shorter than funicle. Funicle 4-segmented, segment 1 as long as pedicel. Club longer than wide, flat, type 3; segment 1 corneous, transverse on anterior face, occupying approximately basal 2/5; segment 2 narrow, corneous; segments 1–3 present on posterior face. ***Pronotum***: 1.08 × as long as wide. In dorsal view conical, type 0, sides convex, conical anteriorly; anterior margin without serrations. In lateral view tall, type 2, summit pronounced, just behind middle. Anterior slope with densely spaced, broad asperities, becoming lower and more strongly transverse towards summit. Disc subshiny, alutaceous, with moderately dense punctures, punctures setose, each bearing a very long, recumbent or semi-erect, hair-like seta, some longer hair-like setae at margins. Lateral margins obliquely costate. Base transverse, posterior angles broadly rounded. ***Elytra***: 1.9× as long as wide, 1.86× as long as pronotum. Scutellum moderately sized, narrowly linguiform, slightly raised above elytra, flat, shiny. Elytral mycangium consisting of two oblong pit mycangia immediately adjacent to scutellum, one on each elytron. Elytral base transverse, edge oblique, humeral angles rounded, parallel-sided in basal 1/3 then gradually tapering to angularly rounded apex. Disc flat shiny, striae weakly impressed, with moderately sized deep punctures separated by two diameters of a puncture, punctures setose, setae semi-erect, slightly longer than puncture diameter; interstriae flat, punctate, punctures minute and widely spaced, setose, setae longer than 2× interstriae 1 width, semi-erect, hair-like. Declivity beginning after basal 1/4, gradually, evenly sloping to apex, strongly shiny; strial punctures as large as those of disc, striae weakly impressed, punctures setose, setae like those of disc, interstriae weakly laterally broadened from declivital summit to apical 1/3 then narrowed to apex, basal 3/4 of interstriae 1–3 uniseriate punctate, punctures subequal to those of striae, apical 1/4 impunctate, punctures replaced by four granules, granules widely spaced, interstriae 4–8 impunctate and unarmed. Posterolateral margin rounded. ***Legs***: protibiae obliquely triangular, broadest at apical 1/3; posterior face smooth; apical 1/2 of outer margin with six large socketed denticles, their length much longer than basal width. Meso- and metatibiae flattened; with obliquely triangular outer margin with ten large socketed denticles.

##### Etymology.

L. *fastigatus* = sloping. In reference to the form of the elytra which slope downwards almost from the base. An adjective.

##### Distribution.

India (Meghalaya), Nepal.

##### Host plants.

Unknown.

##### Remarks.

The holotype is card mounted and ventral characters could not be examined.

**Figure 63.**
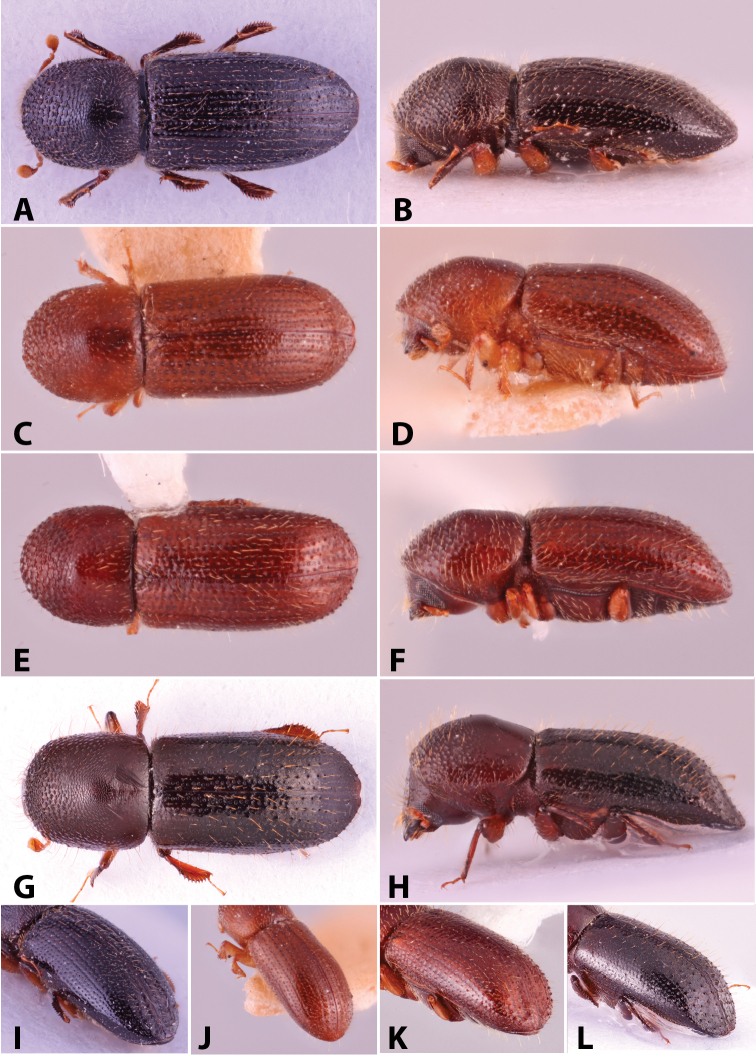
Dorsal, lateral and declivital view of *Heteroborips fastigatus* holotype, 3.4–3.5 mm (**A, B, I**), *H. indicus* holotype, 2.2–2.4 mm (**C, D, J**), *H. seriatus*, 2.5–2.9 mm (**E, F, K**), and *H. tristis*, 3.9–4.0 mm (**G, H, L**).

#### Heteroborips indicus sp. nov.

Taxon classificationAnimaliaColeopteraCurculionidae

http://zoobank.org/C5BEA9E4-D1C8-4170-9197-AB9AC625582B

Fig. 63C, D, J

##### Type material.

***Holotype***, female, India: [West] Bengal, Darjeeling, Debrepani, 6000 ft, 20.xi.1929, J.C.M. Gardner, ex *Michelia excelsa* (NMNH). ***Paratypes***, female, as holotype (NMNH, 5).

##### Diagnosis.

2.2–2.4 mm long (mean = 2.3 mm; n = 4); 2.75–3.0× as long as wide. This species is distinguished by the distinctive elytral mycangium which appears as a distinctly impressed area immediately adjacent to the scutellum on each elytron; and declivity broadly sloping, occupying 1/2 of elytra.

##### Similar species.

*Heteroborips fastigatus*, *H. seriatus*, *Tricosa cattienensis*, *T. indochinensis*.

##### Description

**(female).** 2.2–2.4 mm long (mean = 2.3 mm; n = 4); 2.75–3.0× as long as wide. Body, antennae and legs uniformly light brown. ***Head***: epistoma entire, transverse, with a row of hair-like setae. Frons flattened to upper level of eyes; surface shiny, punctate, setose; punctures dense, becoming shallower and sparser on reticulate upper part of frons. Eyes feebly emarginate just above antennal insertion, upper part smaller than lower part. Submentum flat, flush with genae, triangular. Antennal scape regularly thick, as long as club. Pedicel as wide as scape, as long as funicle. Funicle 4-segmented, segment 1 shorter than pedicel. Club longer than broad, obliquely truncate, type 2; segment 1 corneous, transverse on anterior face, occupying basal 2/5, nearly covering posterior face; segment 2 narrow, corneous; segment 1 present on posterior face. ***Pronotum***: 1.0× as long as wide. In dorsal view conical, type 0, sides convex, conical anteriorly; anterior margin without serrations. In lateral view tall, type 2, summit pronounced, just behind middle. Anterior slope with densely spaced small asperities, becoming lower and more strongly transverse towards summit. Disc alutaceous, subshiny, with sparse coarse punctures bearing short, recumbent setae, some longer hair-like setae at margins. Lateral margins obliquely costate. Base transverse, posterior angles broadly rounded. ***Elytra***: 1.88× as long as wide, 1.88× as long as pronotum. Scutellum moderately sized, linguiform, slightly raised above elytra, flat, shiny. Elytral mycangium comprised of two oblong pit mycangia immediately adjacent to scutellum, one on each elytron. Elytral base transverse, edge oblique, humeral angles rounded, parallel-sided in basal 3/4, then acuminate to apex. Disc ascending apically, shiny, striae not impressed, with moderately sized deep punctures separated by 2–4 diameters of a puncture, punctures setose, setae semi-erect, slightly longer than puncture diameter; interstriae flat, impunctate, glabrous. Declivity occupying 1/2 of elytral length, shiny, gradually rounded; strial punctures larger than on disc, striae weakly impressed, punctures setose, setae semi-erect, as long as interstriae 1 width; interstriae laterally broadened from declivital summit to apical 1/3 then narrowed to apex, basal 1/2 of interstriae 1–3 uniseriate punctate, punctures subequal to those of striae, apical 1/2 impunctate, punctures replaced by 4–7 granules, granules widely spaced, interstriae 4–8 impunctate and unarmed, setose, as described for striae. Posterolateral margin rounded. ***Legs***: procoxae contiguous. Protibiae distinctly triangular, posterior face smooth; apical 1/3 of outer margin with four or five large socketed denticles, their length longer than basal width. Meso- and metatibiae flattened; outer margin evenly rounded with eight or nine and seven or eight large socketed denticles, respectively.

##### Etymology.

L. *indicus* = of India. An adjective.

##### Distribution.

India (West Bengal).

##### Host plants.

This species has only been reported from *Michelia* (Magnoliaceae).

##### Remarks.

The entire type series is card mounted and ventral characters could not be examined.

#### Heteroborips seriatus

Taxon classificationAnimaliaColeopteraCurculionidae

(Blandford, 1894)

Fig. 63E, F, K

Xyleborus seriatus Blandford, 1894b: 111.
Heteroborips seriatus (Blandford): Mandelshtam et al. 2019: 392.
Xyleborus orientalis Eggers, 1933b: 54. Synonymy: Mandelshtam 2006: 324.
Xyleborus todo Kôno, 1938: 71. Smith et al. 2018b: 399.
Xyleborus orientalis kalopanacis Kurentzov, 1941: 187. Synonymy: Knížek 2011: 249.
Xyleborus orientalis aceris Kurentzov, 1941: 188. Synonymy: Knížek 2011: 249.
Xyleborus perorientalis Schedl, 1957: 85. Unnecessary replacement name. Synonymy: Knížek 2011: 249.

##### Type material.

***Holotype****Xyleborus orientalis* (NMNH). ***Syntypes****Xyleborus seriatus* (NHMUK).

##### Diagnosis.

2.5–2.9 mm long (mean = 2.64 mm; n = 5); 2.78–3.0× as long as wide. This species is distinguished by the unique elytral mycangium appearing as a distinctly impressed area immediately adjacent to the scutellum on each elytron; and declivity steeply sloping, occupying apical 1/4.

##### Similar species.

*Heteroborips cryptographus*, which is distributed from Europe to the Russian Far East, and *H. indicus*.

##### Distribution.

China (Shaanxi, Shanxi, Sichuan), Japan, South & North Korea, Russia (Far East, Kuril Is). Introduced and established in USA (Hoebeke and Rabaglia 2008).

##### Host plants.

Polyphagous attacking both conifers and angiosperms (Hoebeke and Rabaglia 2008).

##### Remarks.

The gallery system is unusual in lying between the bark and wood and not penetrating the wood. The parent female, larvae and pupae are all found together in communal chambers under the bark (Murayama 1955; Nakashima et al. 1992).

#### Heteroborips tristis

Taxon classificationAnimaliaColeopteraCurculionidae

(Eggers, 1930) comb. nov.

Fig. 63G, H, L

Xyleborus tristis Eggers, 1930: 194.
Euwallacea tristis (Eggers): Wood and Bright 1992: 694; Smith et al. 2018a: 138.

##### Type material.

***Neotype*** (NHMB).

##### New records.

India: Arunachal Pradesh, Etalin vicinity, 28°36'56"N, 95°53'21"E, 700 m, 12–25.v.2012, L. Dembický (ZFMK, 2). Meghalaya, 3 km E Tura, 25°30'N, 90°14'E, 1150 m, 4.v.1999, Dombický & Pacholátko (NHMB, 1; RABC, 1).

##### Diagnosis.

3.9–4.0 mm long (mean = 3.92 mm; n = 3); 2.79–3.25× as long as wide. This species can be recognized by the unique elytral mycangium appearing as a distinct impressed area immediately adjacent to the scutellum on each elytron, its posterior margin distinctly raised; large size, pronotum from dorsal view appearing subquadrate (type 3); declivity steeply sloping, occupying apical 3/4 of elytra; and elytral disc flat and transverse.

##### Similar species.

*Euwallacea luctuosus*, *E. sibsagaricus*, *E. subalpinus*.

##### Distribution.

India (Arunachal Pradesh*, Assam, Meghalaya, West Bengal).

##### Host plants.

Recorded from *Vatica* (Dipterocarpaceae) and *Macaranga* (Euphorbiaceae) (Maiti and Saha 2004).

##### Remarks.

*Xyleborus tristis* is here transferred to *Heteroborips* because of the distinct elytral mycangia adjacent to the scutellum on each elytron.

### *Immanus* Hulcr & Cognato, 2013

#### Immanus

Taxon classificationAnimaliaColeopteraCurculionidae

Hulcr & Cognato, 2013

Immanus Hulcr & Cognato, 2013: 100.

##### Type species.

*Xyleborus colossus* Blandford, 1896b; original designation.

##### Diagnosis.

This is the largest xyleborine genus with species ranging between 5.0–8.8 mm (Hulcr and Cognato 2013). *Immanus* is distinguished by its large size; robust form (1.97–2.5× as long as wide); truncate or rounded declivity; pronotum anterior margin with elevated carina or a row of 4–6 coarse asperities; pronotal disc asperate; tibial denticles reduced or absent on meso- and metatibiae; tibial edge very uneven and rugged; scutellum flat, flush with elytra; procoxae contiguous; and mycangial tufts absent.

##### Similar genera.

*Ambrosiodmus*, *Beaverium*.

##### Distribution.

Paleotropical.

##### Gallery system.

The gallery system is branched and lies in one transverse plane (Kalshoven 1959b).

##### Remarks.

The genus has been recently reviewed by Beaver et al. (2019) and an additional species has since been described (Wang et al. 2020).

#### Key to *Immanus* species (females only)

**Table:** 

1	Declivity rounded; larger, 7.0–7.8 mm	*** sarawakensis ***
–	Declivity truncate with a circumdeclivital costa; smaller, 5.0–5.5 mm	*** desectus ***

#### Immanus desectus

Taxon classificationAnimaliaColeopteraCurculionidae

(Eggers, 1923)

Fig. 64A, B, E

Xyleborus desectus Eggers, 1923: 167.
Ambrosiodmus desectus (Eggers): Wood and Bright 1992: 672.
Immanus desectus (Eggers): Beaver et al. 2014: 53.
Xyleborus desectus arduus Schedl, 1942a: 188. Synonymy: Wood and Bright 1992: 673.

##### Type material.

***Lectotype*** (NMNH).

##### New records.

Thailand: [Prachuap Khiri Khan]: Kui Buri N.P., 27.iii.2006, Dole et al., ex “Krachid” dead standing trunk (MSUC, 7). Vietnam: Tonkin, Hoa-Binh region, A. DeCooman, 1940 (MNHN, 2).

##### Diagnosis.

5.0–5.5 mm long (mean = 5.2 mm; n = 5); 2.27–2.5× as long as wide. Most closely resembles *I. colossus* (Blandford, 1896), which occurs in Papua New Guinea. *Immanus desectus* is distinguished by the smaller size; truncate declivity with a circumdeclivital costa; and two or three denticles on declivital interstriae 2 rather than a row of denticles.

##### Similar species.

*Immanus colossus* (from Papua New Guinea), *I. sarawakensis*.

##### Distribution.

Indonesia (Java), West Malaysia, Philippines, Thailand, Vietnam.

##### Host plants.

Recorded only from *Castanospermum* (Fabaceae) (Kalshoven 1959b).

**Figure 64.**
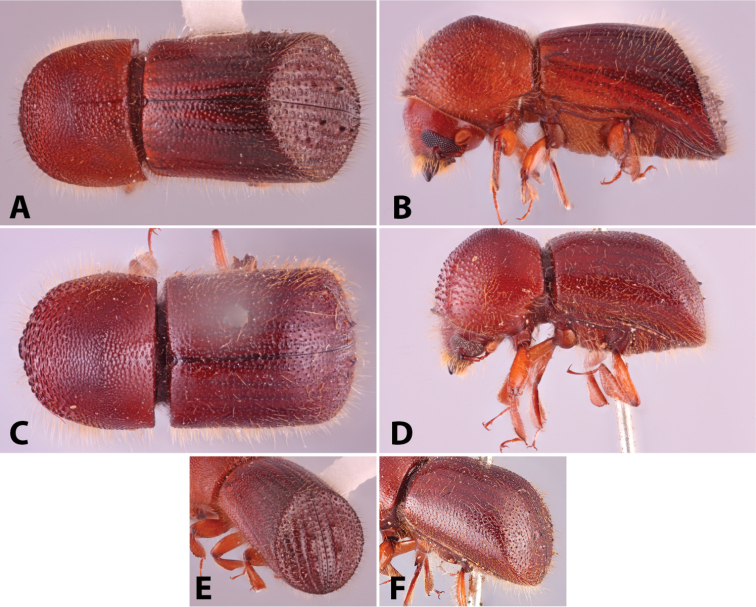
Dorsal, lateral and declivital view of *Immanus desectus*, 5.0–5.5 mm (**A, B, E**), and *I. sarawakensis* holotype, 7.0–7.8 mm (**C, D, F**).

#### Immanus sarawakensis

Taxon classificationAnimaliaColeopteraCurculionidae

(Eggers, 1923)

Fig. 64C, D, F

Xyleborus sarawakensis Eggers, 1923: 176.
Ambrosiodmus sarawakensis (Eggers): Wood and Bright 1992: 680.
Immanus sarawakensis (Eggers): Beaver et al. 2019: 385.

##### Type material.

***Holotype*** (MCG).

##### Diagnosis.

The largest species occurring in Southeast Asia, 7.0–7.8 mm long (mean = 7.26 mm; n = 5); 1.97–2.05× as long as wide. This species is distinguished by the very large size; rounded declivity; densely setose body; declivital interstriae 2 bearing a row of two or three denticles; and all declivital interstriae are slightly elevated.

##### Similar species.

*Immanus colossus* (from Papua New Guinea), *I. desectus*.

##### Distribution.

East & West Malaysia, Thailand.

##### Host plants.

Recorded from *Lophopetalum* (Celastraceae), *Parinari* (Chrysobalanaceae), *Xanthophyllum* (Polygalaceae) and an unidentified genus of Annonaceae (Browne 1961b).

### *Leptoxyleborus* Wood, 1980

#### Leptoxyleborus

Taxon classificationAnimaliaColeopteraCurculionidae

Wood, 1980

Leptoxyleborus Wood, 1980: 94.

##### Type species.

*Phloeotrogus sordicauda* Motschulsky, 1863; original designation.

##### Diagnosis.

1.9–3.6 mm, 2.15–2.52× as long as wide. *Leptoxyleborus* is distinguished by the declivity extremely flat and broad, especially laterally; posterolateral declivital margin carinate, ending at interstriae 7; surface covered with bristles or minute star-shaped scales; pronotum elongate, appearing conical and elongate from dorsal aspect (type 5); antennal club truncate, type 2, with segment 1 nearly covering the entire posterior face; submentum slightly impressed, shaped as a large triangle; protibiae narrow, with fewer than six denticles; scutellum flat, flush with elytra; procoxae contiguous; mycangial tufts absent; and elytra unarmed.

##### Similar genera.

*Ancipitis*, *Diuncus*.

##### Distribution.

Paleotropics and Oceania.

##### Gallery system.

Consists of a system of irregularly branched tunnels without brood chambers, lying more or less in one transverse plane. When the host tree has thick bark, transverse surface galleries may also be made between the bark and wood (Beeson 1930; Browne 1961b).

##### Remarks.

Hulcr and Cognato (2013) incorrectly state that the submentum is shaped as a very narrow triangle; it is shaped as a large and broad triangle.

#### Key to *Leptoxyleborus* species (females only)

**Table:** 

1	Declivital interstriae bearing uniseriate short, semi-erect scales; posterolateral declivital margin costate; smaller, 1.9–2.1 mm	*** machili ***
–	Declivital interstriae covered with minute star-shaped scales; posterolateral declivital margin carinate; larger, 2.6–3.6 mm	*** sordicauda ***

#### Leptoxyleborus machili

Taxon classificationAnimaliaColeopteraCurculionidae

(Niisima, 1910) comb. nov.

Fig. 65A, B, E

Xyleborus machili Niisima, 1910: 14.
Ancipitis machili (Niisima): Smith et al. 2018b: 393.
Xyleborus depressus Eggers, 1923: 190. Synonymy: Smith et al. 2018b: 393.
Xyleborus kojimai Murayama, 1936: 143. Synonymy: Smith et al. 2018b: 393.
Xyleborus sejugatus Schedl, 1942a: 188. Synonymy: Wood 1989: 175.

##### Type material.

***Holotype****Xyleborus depressus* (NHMW).

##### New records.

China: Jiangxi, Long Nan, 12.vii.2016, Lv-Jia, Lai, S-C., ex *Cyclobalanopsis glauca* (RABC, 1). Japan: Kagoshima Pref., Tarumizu Oonohara broadleaf forest, 425 m, 1.viii.2000, Y. Sato, ex EtOH baited trap (RJRC, 1); as previous except: 27.viii.2000 (RJRC, 1).

##### Diagnosis.

1.9–2.1 mm long (mean = 1.99 mm; n = 5); 2.22–2.53× as long as wide. The species is readily distinguished by its small size and declivital interstriae bearing uniseriate short, semi-erect scales; and posterolateral declivital margin costate.

##### Similar species.

*Ancipitis puer*, *Leptoxyleborus sordicauda*.

##### Distribution.

China* (Jiangxi), Indonesia (Sumatra), Japan*, East & West Malaysia, Solomon Islands, Thailand.

##### Host plants.

Polyphagous (Browne 1961b; Ohno 1990; Ohno et al. 1988).

##### Remarks.

This species bears striking morphological similarity to *Ancipitis* species. Upon close examination of specimens we determined that this species should be moved to *Leptoxyleborus* because of the following combination of characters: antennal club truncate, type 2, with segment 1 nearly covering the entire posterior face; segment 1 of the antennal club shorter than pedicel; scape regularly thick; protibiae obliquely truncate; elytral interstriae seriate and bearing scales; and declivital face flat.

**Figure 65.**
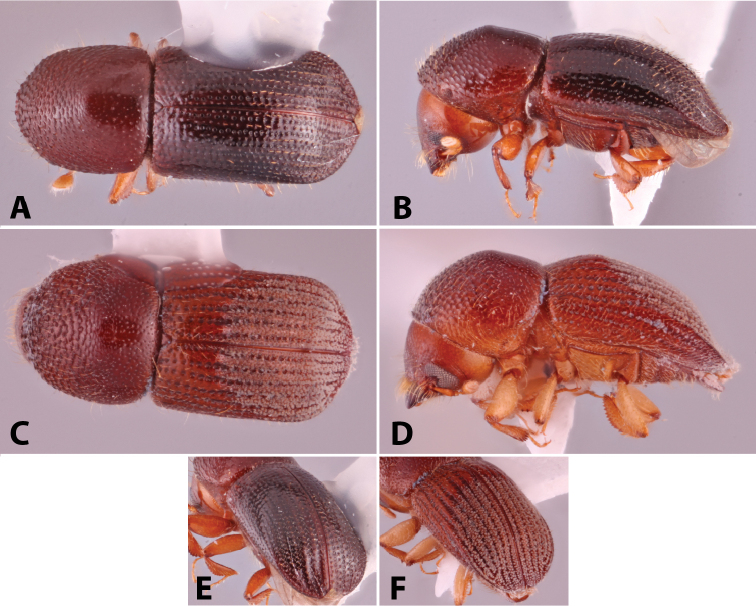
Dorsal, lateral and declivital view of *Leptoxyleborus machili*, 1.9–2.1 mm (**A, B, E**), and *L. sordicauda*, 2.6–3.6 mm (**C, D, F**).

#### Leptoxyleborus sordicauda

Taxon classificationAnimaliaColeopteraCurculionidae

(Motschulsky, 1863)

Fig. 65C, D, F

Phloeotrogus sordicauda Motschulsky, 1863: 514.
Leptoxyleborus sordicauda (Motschulsky): Wood 1980: 94.
Phloeotrogus attenuatus Motschulsky, 1863: 512. Synonymy: Wood 1969: 119.
Xyleborus concisus Blandford, 1894b: 107. Synonymy: Hulcr and Cognato 2013: 103.
Xyleborus marginatus Eggers, 1927b: 91. Synonymy: Browne 1955: 354.
Xyleborus sordicaudulus Eggers, 1927b: 91. Synonymy: Browne 1955: 354.
Xyleborus incurvus Eggers, 1930: 197. Synonymy: Wood 1989: 175.
Xyleborus sordicaudulus peguensis Eggers, 1930: 198. Synonymy: Schedl 1951a: 51.

##### Type material.

***Holotype****Xyleborus concisus* (NHMUK). ***Holotype****Xyleborus incurvus* (FRI), ***paratype*** (NMNH, 2). ***Holotype****Xyleborus marginatus* (NMNH). ***Holotype****Xyleborus sordicaudulus* (NMNH), ***paratype*** (NMNH, 1). ***Holotype****Xyleborus sordicaudulus peguensis* (FRI).

##### New records.

China: Guangxi Reg., Miaoershan, S slope, 800–1300 m, 20–27.vi.1997, Bolm (NHMB, 1). Jiangxi, Long Nan, 12.vii.2016, Lv-Jia, Lai S-C., ex *Cyclobalanopsis glauca* (RABC, 1). Vietnam: Dong Nai, Cat Tien National Park, near park headquarters, 11°25'44"N, 107°25'44"E, 120 m, 26–31.v.1999, B. Hubley, D. Currie, VIET1H95-99 041, ex flight intercept trap (SEMC, 2); as previous except: 11.42854; 107.42544, 148 m, 23.ii.2017, VN97, A.I. Cognato, T.A. Hoang, ex 5 cm diameter branch (MSUC, 23).

##### Diagnosis.

2.6–3.6 mm long (mean = 2.84 mm; n = 5); 2.15–2.5× as long as wide. This species is distinguished by the larger size, declivity extremely flat and broad; especially laterally; posterolateral declivital margin elevated, carinate; declivital surface covered with minute star-shaped scales.

##### Similar species.

*Leptoxyleborus machili*, *Ancipitis puer*, *A. punctatissimus*.

##### Distribution.

China* (Guangxi, Jiangxi), India (Andaman Is, West Bengal), Indonesia (Java, Maluku, Sumatra), Japan, East & West Malaysia, Myanmar, New Guinea, Philippines, Taiwan, Thailand, Vietnam.

##### Host plants.

Polyphagous (Browne 1961b).

##### Remarks.

The species attacks large logs, smaller stems down to approximately 3 cm diameter, and lianas (Browne 1961b). Beaver and Browne (1979) suggest that it may be particularly attracted to sappy stems.

### *Microperus* Wood, 1980

#### Microperus

Taxon classificationAnimaliaColeopteraCurculionidae

Wood, 1980

Microperus Wood, 1980: 94.

##### Type species.

*Xyleborus theae* Eggers, 1940 = *Xyleborus myristicae* Schedl, 1939b; original designation.

##### Diagnosis.

1.2–3.1 mm, 1.93–3.17× as long as wide. *Microperus* is distinguished by the scutellum either narrow, minute, convex and slightly raised above elytra or not visible; dense tuft of setae present along elytral base associated with an elytral mycangium; elytral bases sinuate (rarely transverse), costate; antennal club truncate (type 2) or flattened, types 3 or 4, sutures gently sinuate and pubescent on anterior face, 1–3 sutures visible on posterior face; pronotum from lateral view taller than basic (type 2) or with pronotal disc longer than anterior slope (type 7); pronotum from dorsal view basic and parallel sided (type 2), or subquadrate (type 3); anterior margin of pronotum without a row of serrations; and pronotal disc punctate. In addition, the procoxae are contiguous, outer margin of protibiae obliquely or distinctly triangular and armed by 6–8 denticles, and posterior face flattened, unarmed.

##### Similar genera.

*Coptodryas*, *Xyleborinus*.

##### Distribution.

Found throughout the Paleotropics, Australia and Oceania.

##### Gallery system.

The tunnels are irregularly branched, more or less in one transverse plane, and enlarged into small brood chambers in the longitudinal plane in places. In a few species (e.g., *M. corporaali*, *M. nugax*, *M. undulatus*), the brood chambers are in the transverse plane (Browne 1961b).

##### Remarks.

*Microperus* is in need of further taxonomic/phylogenetic investigation given its potential polyphyly and confusion with *Coptodryas* (Hulcr et al. 2007; Cognato et al. 2020b). Hulcr and Cognato (2013) suggest that the species may engage in communal breeding, as a result of interconnecting gallery systems.

#### Key to *Microperus* species (females only)

**Table:** 

1	Elytral disc broadly, deeply transversely impressed with a saddle-like depression from scutellum to declivital base; declivity deeply sulcate, its margins costate; elytral bases slightly emarginated from sutural margin to interstriae 4 to accommodate mycangial tuft	*** cruralis ***
–	Elytral disc either medially impressed and appearing humped, or flat, or broadly convex; declivity flat or convex its margins flat; elytral bases not emarginated	**2**
2	Declivity obliquely truncate; posterolateral declivital margin rounded and denticulate (Fig. 68B)	**3**
–	Declivity rounded; posterolateral declivital margin costate or carinate, with or without granules (Fig. 66F)	**6**
3	Declivital interstriae 2 and 3 strongly laterally broadened from base to declivital midpoint and then narrowing towards apex (Fig. 68A, I)	***latesalebrinus* sp. nov.**
–	Declivital interstriae parallel from base to apex, never laterally broadened (Fig. 69A, I)	**4**
4	Denticles on declivital summit and margins larger and more sharply acute than those on declivital face	*** kirishimanus ***
–	Denticles on declivital summit of equal size and shape as those on declivital face	**5**
5	Denticles on declivital summit as dense as those on declivital face; declivital face opalescent, subshiny	*** nudibrevis ***
–	Denticles on declivital summit denser than those on declivital face; declivital face shagreened, dull	*** perparvus ***
6	Larger, 2.55–2.95 mm	**7**
–	Smaller, 1.2–2.1 mm	**9**
7	Stout, 1.93–2.19× as long as wide; elytral posterolateral margin strongly carinate and unarmed	*** fulvulus ***
–	Elongate, 2.5–2.9× as long as wide; elytral posterolateral margin costate and granulate	**8**
8	Declivital strial punctures very large, distinct	*** chrysophylli ***
–	Declivital strial punctures small, indistinct	*** corporaali ***
9	Declivity with granules, denticles or tubercles distinctly less abundant than strial punctures (Fig. 66A)	**10**
–	Declivity with abundant granules or denticles, at least as abundant as strial punctures (Fig. 68C)	**13**
10	Elytral disc shallowly transversely impressed with a saddle-like impression (Fig. 70B, D)	**11**
–	Elytral disc without a depression (Fig. 69H)	**12**
11	Discal impression deeper, antero-posteriorly narrower, with steeper anterior and posterior slopes, strial punctures on impression with rounded granules (Fig. 70B); interstrial spines on disc behind impression stronger and backwardly hooked	***sagmatus* sp. nov.**
–	Discal impression shallower, antero-posteriorly broader, with gentler anterior and posterior slopes strial punctures on impression without granules (Fig. 70D); interstrial tubercles on disc behind impression moderate with rounded apices pointing dorsally.	*** undulatus ***
12	Declivital denticles uniformly sized; smaller, 1.7–1.8 mm	*** alpha ***
–	Declivital denticles not uniformly sized, one or two pairs of slightly larger denticles on declivital interstriae 3; larger, 1.9–2.0 mm	*** recidens ***
13	Elytral disc convex on basal 1/3, appearing humped in lateral view (Fig. 68D)	**14**
–	Elytral disc flat, never appearing humped (Fig. 69F)	**16**
14	Declivital interstriae densely covered with short semi-erect scales	*** kadoyamaensis ***
–	Declivital interstriae densely covered with long fine, erect hair-like setae	**15**
15	Antennal club flat, type 3 with two sutures visible on apical 1/3 of posterior face (Fig. 3); larger, declivity smooth, shiny; larger, 1.95–2.0 mm and more elongate, 2.79–2.86× as long as wide	***minax* sp. nov.**
–	Antennal club obliquely truncate, type 2 with segment 1 almost covering posterior face (Fig. 2); one suture visible on posterior face near apex; declivity shagreened, dull; smaller, 1.8–1.9 mm and less elongate, 2.57–2.71× as long as wide	*** nugax ***
16	Antennal club flat, type 3 with two sutures visible on apical 1/3 of posterior face (Fig. 3)	*** diversicolor ***
–	Antennal club obliquely truncate, type 2 with segment 1 almost covering posterior face (Fig. 2); one suture visible on posterior face near apex	**17**
17	Declivital interstrial granules dispersed, separated by the width of at least three granules; posterolateral margin of declivity weakly carinate and granulate; interstrial vestiture consisting of short semi-erect bristles, shorter in length than the width of an interstria; smaller, 1.2–1.7 mm	*** pometianus ***
–	Declivital interstrial granules dense, separated by the width of one granule; posterolateral margin of declivity strongly carinate; interstrial vestiture consisting of long semi-erect hair-like setae, longer in length than the width of an interstria (easily abraded); larger, 1.8–2.0 mm	*** quercicola ***

#### Microperus alpha

Taxon classificationAnimaliaColeopteraCurculionidae

(Beeson, 1923)

Fig. 66A, B, I

Xyleborus bicolor Blandford, var. α Sampson, 1923: 289.
Xyleborus alpha Beeson, 1929: 239.
Coptodryas alpha (Beeson): Wood and Bright 1992: 823.
Microperus alpha (Beeson): Hulcr 2010: 111.

##### Type material.

***Holotype*** (NHMUK).

##### New records.

China: S Yunnan, Xishuangbanna, 23 km NW Jinghong, vic. Na Ban village (NNNR), 22°10'N, 100°39'E, 700–1000 m, v–vii.2009, L. Meng (RABC, 1); Xishuangbanna tropical botanical garden, 17.vii.2014, C. Bateman, ex unknown wood (UFFE, 1); as previous except: Mengyang, 12.v.1962 (NMNH, 1). India: Arunachal Pradesh, Etalin vicinity, 28°36'56"N, 95°53'21"E, 700 m, 12–25.v.2012, L. Dembický (ZFMK, 2); as previous except: Bhalukpong, 27°00'48"N, 92°39'08"E, 150 m, 1–8.v.2012, L. Dembický (ZFMK, 1). Laos: Kham Mouan, Ban Khoun Ngeun, 18°07'N, 104°29'E, ~ 200 m, 24–29.iv.2001, P. Pacholátko (NHMB, 2). Louangphrabang, Ban Song Cha (5 km W), 20°33–4'N, 102°14'E, 1200 m, 1–16.iv.1999, V. Kubáň (RABC, 1). Oudomxai, Oudomxai, 17 km NE, 20°45'N, 102°09'E, ~ 1100 m, 1–9.v.2002, V. Kubáň (NHMB, 1). Vientiane, Ban Van Eue, 15–31.v.1965, native collector, ex light trap (BPBM, 1). Vietnam: Thua Thien-Hue, Bach Ma N.P., 16.22897, 107.85349, 415 m, 15.ii.2017, VN57, A.I. Cognato, T.A. Hoang, ex 5 cm diameter branch; twig (MSUC, 4).

##### Diagnosis.

1.7–1.8 mm long (mean = 1.74 mm; n = 5); 2.43–2.83× as long as wide. This species is distinguished by the elytral disc flat with short, steep declivity; declivital posterolateral margin carinate; declivity with sparse minor denticles, less abundant than strial punctures, and denticles uniform in size.

##### Similar species.

*Microperus recidens*.

##### Distribution.

Bangladesh, China (Guizhou, Yunnan*), India (Arunachal Pradesh*, Assam, West Bengal), Laos*, West Malaysia, Sri Lanka, Taiwan, Thailand, Vietnam.

##### Host plants.

Polyphagous (Maiti and Saha 2004).

**Figure 66.**
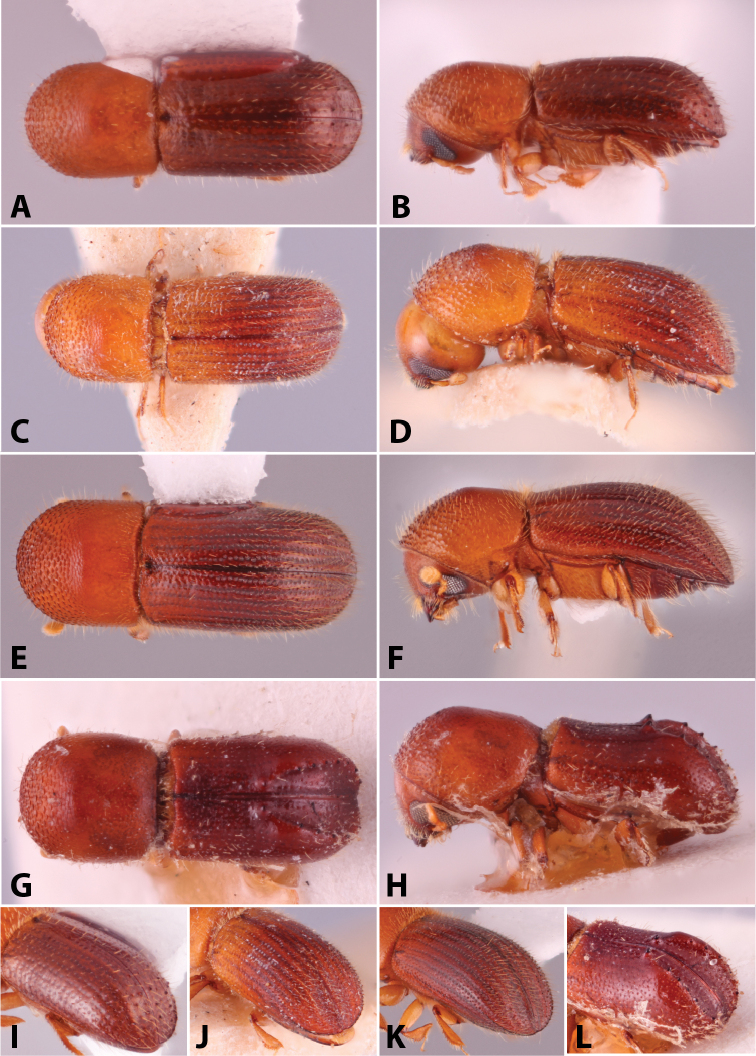
Dorsal, lateral and declivital view of *Microperus alpha*, 1.7–1.8 mm (**A, B, I**), *M. chrysophylli* paratype, 2.6–2.7 mm (**C, D, J**), *M. corporaali*, 2.55–2.9 mm (**E, F, K**), and *M. cruralis* holotype, 3.0–3.1 mm (**G, H, L**).

#### Microperus chrysophylli

Taxon classificationAnimaliaColeopteraCurculionidae

(Eggers, 1930)

Fig. 66C, D, J

Xyleborus chrysophylli Eggers, 1930: 205.
Coptodryas chrysophylli (Eggers): Wood and Bright 1992: 823.
Microperus chrysophylli (Eggers): Saha and Maiti 1996: 824.

##### Type material.

***Holotype*** (FRI), ***paratype*** (NMNH, 1).

##### New records.

China: Yunnan, Xishuangbanna, Jinghong City, Jinghong Farm, 21.785N, 100.790E, 677 m, 18.vii.2018, Lai, S-C, Zhang, L., ex *Hevea brasiliensis* (RABC, 1).

##### Diagnosis.

2.6–2.7 mm long (mean = 2.68 mm; n = 5); 2.6–2.7× as long as wide. This species is distinguished by the elytral disc flat; declivity long, gradual; large size; declivital interstriae 2 lacking granules on declivital face; declivital face strongly shagreened, weakly impressed along striae 2 and interstriae 2; declivital strial punctures small, indistinct; posterolateral costa granulate; interstriae densely covered with long erect hair-like setae, setae longer than two interstrial widths; and striae setose, setae short, semi-recumbent, as long as strial width.

This species strongly resembles *M. corporaali* and is distinguished by the less strongly sulcate declivity, declivital strial punctures very large, distinct.

##### Similar species.

*Microperus corporaali*, *M. fulvulus*.

##### Distribution.

Bangladesh, China* (Yunnan), India (West Bengal).

##### Host plants.

Recorded from *Cinnamomum* (Lauraceae), *Chrysophyllum* (Sapotaceae), (Maiti and Saha 2004), and *Hevea brasiliensis* (Euphorbiaceae).

#### Microperus corporaali

Taxon classificationAnimaliaColeopteraCurculionidae

(Eggers, 1923)

Fig. 66E, F, K

Xyleborus corporaali Eggers, 1923: 210.
Coptodryas corporaali (Eggers): Wood and Bright 1992: 823.
Microperus corporaali (Eggers): Hulcr 2010: 111.

##### Type material.

***Lectotype*** (NMNH).

##### New records.

China: Guangxi, Shangsi, 25.iii.2018, Y. Li, ex *Quercus* (UFFE, 3). Yunnan, Xishuangbanna, 20 km NW Jinghong, vic. Man Dian (NNNR), 22°07.80'N, 100°40.0'E, 730 m, forest, EK, 6.iv.2009, L. Meng (RABC, 1). Vietnam: Cao Bang, 22°34.5'N, 105°52.4'E, ~ 1080 m, 14.iv.2014, VN31, Cognato, Smith, Pham, ex emerging from bark of standing dead tree (MSUC, 7). Ninh Binh, Cuc Phuong N.P., 20.25000, 105.71495, 7.iii.2018, 158 m, A.I. Cognato, S.M. Smith, VN 150, ex 4 cm diameter living branch (MSUC, 12).

##### Diagnosis.

2.55–2.9 mm long (mean = 2.72 mm; n = 5); 2.5–2.9× as long as wide. This species is distinguished by the elytral disc flat; declivity long, gradual; large size; declivital interstriae 2 lacking granules on declivital face; declivital face strongly shagreened, weakly impressed along striae 2 and interstriae 2; declivital strial punctures small, indistinct; posterolateral costa granulate; interstriae densely covered with long erect hair-like setae, setae longer than two interstrial widths; striae setose, setae short, semi-recumbent, as long as strial width.

This species strongly resembles *M. chrysophylli* and is distinguished by the more strongly sulcate declivity, declivital strial punctures small, indistinct.

##### Similar species.

*Microperus chrysophylli*, *M. fulvulus*.

##### Distribution.

China* (Guangxi*, Yunnan*), Indonesia (Java, Sumatra), East & West Malaysia, New Guinea, Solomon Islands, Thailand, Vietnam*.

##### Host plants.

Recorded from five different families of trees, and probably polyphagous (Beaver et al. 2014).

#### Microperus cruralis

Taxon classificationAnimaliaColeopteraCurculionidae

(Schedl, 1975) comb. nov.

Fig. 66G, H, L

Xyleborus cruralis Schedl, 1975b: 456.
Coptodryas cruralis (Schedl): Beaver 1995a: 201.

##### Type material.

***Holotype*** (NHMW).

##### New records.

Cambodia: Siem Reap, Angkor Thom, 26.v.2003, J. Constant, K. Smets & P. Grootaert, ex light trap (RABC, 1). Laos: Vientiane, Gi Sion vill., de Tha Ngone, 28.ii.1965, J.L. Gressitt, ex light trap (BPBM, 1).

##### Diagnosis.

3.0–3.1 mm long (mean = 3.03 mm; n = 3); 2.5–2.82× as long as wide. This species is distinguished by its large size; elytral disc broadly and deeply transversely impressed with a saddle-like depression from scutellum to declivital base; declivity deeply sulcate, its margins lined by large tubercles on interstriae 1 and 3–6; elytral base emarginated from sutural margin to interstriae 4 to accommodate mycangial tuft, mycangial tuft setae long, very dense; and posterolateral costa absent.

##### Similar species.

*Microperus nugax*, *M. sagmatus*, *M. undulatus*.

##### Distribution.

Cambodia*, Laos*, Thailand.

##### Host plants.

Unknown.

##### Remarks.

The species is transferred to *Microperus* because of the following characters: pronotum type 2 (viewed dorsally), antennal club flat, type 3, pronotal disc punctate, and scutellum narrow, minute and convex.

#### Microperus diversicolor

Taxon classificationAnimaliaColeopteraCurculionidae

(Eggers, 1923)

Fig. 67A, B, I

Xyleborus diversicolor Eggers, 1923: 202.
Coptodryas diversicolor (Eggers): Wood and Bright 1992: 824.
Microperus diversicolor (Eggers): Hulcr and Cognato 2010a: 19.
Xyleborus myristicae Schedl, 1939b: 49. Synonymy: Hulcr and Cognato 2010a: 19.
Xyleborus theae Eggers, 1940: 144. Synonymy: Wood 1989: 171.
Xyleborus brevipilosus Eggers, 1940: 145. Synonymy: Kalshoven 1959a: 95.
Xyleborus cylindripennis Schedl, 1954a: 152. Synonymy: Wood 1989: 171.
Xyleborus atavus Schedl, 1979b: 104. Synonymy: Hulcr and Cognato 2010a: 19.

##### Type material.

***Lectotype****Xyleborus myristicae* (NHMW).

##### Diagnosis.

1.6–1.8 mm long (mean = 1.68 mm; n = 5); 2.57–2.83× as long as wide. This species is distinguished by the declivital interstriae sparsely covered with short erect bristle-like setae; elytral disc medially convex, appearing humped; antennal club type 3 with two sutures visible on posterior face; declivity short, steep; all declivital interstriae uniformly granulate from base to apex; declivital face convex; and posterolateral costa carinate.

##### Similar species.

*Microperus kadoyamaensis*, *M. minax*.

##### Distribution.

Indonesia (Java, Sumatra), East & West Malaysia, New Guinea, Philippines, Solomon Islands, Thailand.

##### Host plants.

Polyphagous (Browne 1961b).

**Figure 67.**
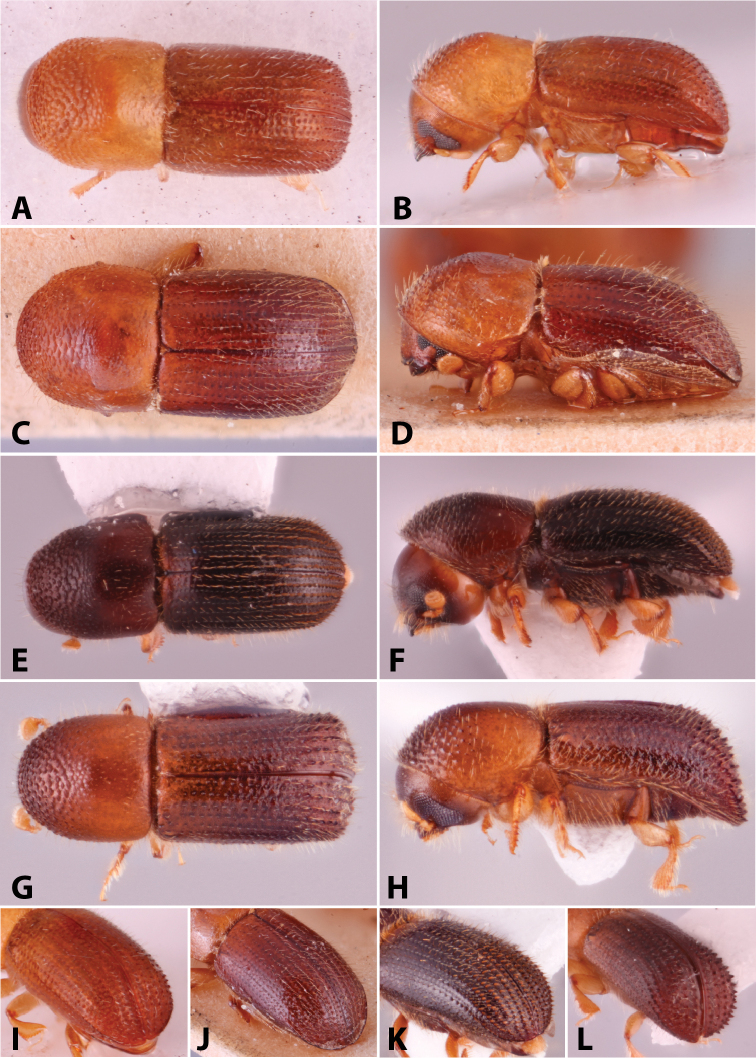
Dorsal, lateral and declivital view of *Microperus diversicolor*, 1.6–1.8 mm (**A, B, I**), *M. fulvulus* lectotype, 2.8–2.95 mm (**C, D, J**), *M. kadoyamaensis*, 1.8–2.0 mm (**E, F, K**), and *M. kirishimanus*, 1.6–1.8 mm (**G, H, L**).

#### Microperus fulvulus

Taxon classificationAnimaliaColeopteraCurculionidae

(Schedl, 1942) stat. res.

Fig. 67C, D, J

Xyleborus fulvus Schedl, 1939b: 48. Preoccupied by Murayama 1936.
Xyleborus fulvulus Schedl, 1942c: 35 (new name for X. fulvus Schedl, 1939 nec Murayama 1936).
Microperus fulvulus (Schedl): Hulcr 2010: 111 (as a synonym of Microperus corporaali Eggers).

##### Type material.

***Lectotype****Xyleborus fulvus* (NHMW), ***paralectotype*** (NHMW, 1).

##### New records.

China: Chongqing Mun., S-W Univ., viii.2015, Su, T-L., ex *Cinnamomum camphora* (RABC, 1). Sichuan, De Chang Co., roadside, vii. 2015, Su, T-L., ex *Prunus yedoensis* (RABC, 1). Thailand: Chiang Mai, Doi Pui, 28°50'23"N, 98°53'53"E, 1200–1300 m, vii.2014, S. Sanguansub et al. (RABC, 1). Chumphon, 1.iii.2010, W. Sittichaya, ex EtOH trap in durian plantn [plantation] (MSUC, 1; RABC, 1). [Chaiyaphum], Phu Khieo N.P., vii.2005, Hulcr et al. (RABC, 1). Nakhon Sri Thammarat, Namtok Yong N.P., Campgrd, 8°10.434'N, 99°44.508'E, 80 m, 8–15.vii.2008, U-prai, K., ex Malaise trap (QSBG, 1); as previous except: 29.vii–5.viii.2008 (RABC, 1); as previous except: 30–31.vii. 2008, ex pan trap (QSBG, 1). Phetchabun, Nam Nao N.P., helicopter landing ground, 16°43.156'N, 101°35.108'E, 890 m, 8–9.vii.2006, N. Hongyothi & L. Janteab, pan traps (QSBG, 1). Songkhla, Rathapum Distr., Silvic. Res. Stn, 21.ii.2009, W. Sittichaya, ex *Cinnamomum iners* branch (RABC, 1).

##### Diagnosis.

2.8–2.95 mm long (mean = 2.88 mm; n = 4), 1.93–2.19× longer than wide. This species is distinguished by the elytral disc flat; declivity long, gradual; all declivital interstriae uniformly granulate from base to apex; declivital face convex; posterolateral costa strongly carinate; interstriae densely covered with long erect hair-like setae, setae longer than two interstrial widths; striae setose, setae short, recumbent, as long as 1.5 strial widths.

This species is distinguished from the closely related *M. chrysophylli and M. corporaali* by the convex declivity and strongly carinate posterolateral costa.

##### Similar species.

*Coptodryas inornata*, *Microperus chrysophylli*, *M. corporaali*.

##### Distribution.

China* (Chongqing, Sichuan), Indonesia (Sumatra), Thailand*.

##### Host plants.

Recorded from *Cinnamomum camphora*, *C. iners* (Lauraceae), *Myristica fragrans* (Myristicaceae), and *Prunus yedoensis* (Rosaceae).

##### Remarks.

The species was incorrectly synonymized with *Microperus corporaali* (Eggers) by Hulcr (2010) based on examination of a *X. fulvus* paratype in the NMNH. Hulcr concluded that the species were morphologically identical. However, comparison of the lectotypes shows that the species are closely related but are significantly different, particularly in regard to the declivity and body size. Specific details are given in the diagnosis and key.

#### Microperus kadoyamaensis

Taxon classificationAnimaliaColeopteraCurculionidae

(Murayama, 1934)

Fig. 67E, F, K

Xyleborus kadoyamaensis Murayama, 1934: 290.
Microperus kadoyamaensis (Murayama): Hulcr et al. 2007: 580.
Xyleborus denseseriatus Eggers, 1941b: 225. syn. nov.
Xyleborus nameranus Murayama, 1954: 194. Synonymy: Smith et al. 2018b: 396.
Xyleborus pubipennis Schedl, 1974: 263. syn. nov.
Xyleborus huangi Browne, 1983b: 34. Synonymy: Beaver 2011: 285.

##### Type material.

***Syntypes****Xyleborus kadoyamaensis* (NMNH, 2). ***Holotype****Xyleborus denseseriatus* (ZMFK). ***Paratype****Xyleborus huangi* (NHMUK). ***Syntypes****Xyleborus nameranus* (NMNH, 2). ***Paratype****Xyleborus pubipennis* (NHMW).

##### New records.

China: Guangdong, Nanling N. P., 25.iii.2005, P. Grootaert (IRSNB, 1). Guangxi Reg., Miaoershan, S slope, 800–1300 m, 20–27.vi.1997, Bolm (RABC, 9). Hong Kong, Tai Po Kau, vi.2017, J. Skelton (MSUC, 1). Jiangxi, Long Nan, 12.vii.2016, Lv-Jia, Lai, S-C., ex *Cyclobalanopsis glauca* (RABC, 1); as previous except: Jinggang Shan Mts., Xiangzhu vill. env., 26°35.5'N, 114°16.0'E, 374 m, rice fields, forested stream valley, M. Fikáček, J. Hájek (NHMP, 1). Yunnan S, Xishuangbanna, 23 km NW Jinghong, vic. Na Ban (NNNR), 22°10'N, 100°39'E, 700–1000 m, v–vii.2009, L. Meng (NKME, 12; RABC, 6); as previous except: 22°09.49'N, 100°39.92'E, 730 m, second[ary]. for[est], 6.vi.2008, A. Weigel (NKME, 1). Zhejiang, Gutianshan Nat. N. Res., 29°8'18"–29°17'29"N, 118°2'14"–118°11'12"E, CSP21-SE/5 (RABC, 1). Vietnam: Cao Bang, 22°34.118'N, 105°52.537'E, 1048 m, 12–17.iv.2014, VN9, Cognato, Smith, Pham, ex FIT (MSUC, 3).

##### Diagnosis.

1.8–2.0 mm long (mean = 1.92 mm; n = 5); 2.86–3.17× as long as wide. This species is distinguished by the declivital interstriae densely covered with short semi-erect scales; elytral disc medially convex, appearing humped; declivity long, gradual; all declivital interstriae uniformly granulate from base to apex; declivital face convex; and posterolateral costa granulate.

##### Similar species.

*Microperus diversicolor*, *M. minax*, *M. quercicola*.

##### Distribution.

China (Fujian, Guangdong*, Guangxi, Hong Kong*, Hunan, Jiangxi*, Yunnan*, Zhejiang*), Japan, South Korea, Taiwan, Vietnam.

##### Host plants.

Polyphagous attacking both gymnosperm and angiosperm trees (Beaver and Liu 2010).

##### Remarks.

*Xyleborus pubipennis* was recently placed in synonymy with *Microperus parvus* (Lea, 1894) (Hulcr and Cognato 2010a). However, the type does not resemble *M. parvus* at all; the declivity is densely setose and clearly the same as that of *M. kadoyamaensis*. Hulcr and Cognato (2013) list the occurrence of *M. parvus* in Vietnam based on their synonymy of *X. pubipennis*. *Microperus parvus* occurs in Australasia not the Oriental region. The *X. denseseriatus* holotype was also examined and found to be conspecific with the syntype series of *X. kadoyamaensis*. Both *X. denseseriatus* and *X. pubipennis* are here placed in synonymy with *M. kadoyamaensis*.

#### Microperus kirishimanus

Taxon classificationAnimaliaColeopteraCurculionidae

(Murayama, 1955)

Fig. 67G, H, L

Xyleborus kirishimanus Murayama, 1955: 85.
Coptodryas kirishimanus (Murayama): Wood and Bright 1992: 825.
Microperus kirishimanus (Murayama): Beaver and Liu 2010: 28.

##### Type material.

***Syntypes*** (NMNH, 4).

##### Diagnosis.

1.6–1.8 mm long (mean = 1.69 mm; n = 5); 2.43–2.75× as long as wide. This species is distinguished by the elytral disc flat with short and steep obliquely truncate declivity; posterolateral carina denticulate; declivital interstriae straight from base to apex, never laterally broadened; denticles on declivital summit and margins larger, more sharply acute and denser than those on declivital face.

##### Similar species.

*Microperus latesalebrinus*, *M. nudibrevis*, *M. perparvus*.

##### Distribution.

Japan, Taiwan.

##### Host plants.

Recorded from *Ilex* (Aquifoliaceae), *Castanopsis*, and *Quercus* (Fagaceae) (Nobuchi 1981d).

##### Remarks.

Murayama (1955) states the type series of *Xyleborus kirishimanus* is comprised of 21 males. Given the heavily female biased sex ratio and biology of male xyleborines it is dubious that 21 males could have been collected and not a single female. This suspicion was confirmed by examination of four syntypes examined by SMS and AIC, all of which are female.

#### Microperus latesalebrinus sp. nov.

Taxon classificationAnimaliaColeopteraCurculionidae

http://zoobank.org/D22F66B1-9A41-411E-B381-00F7E7AA704F

Fig. 68A, B, I

##### Type material.

***Holotype***, female, China: Hong Kong, Tai Po Kau, 3.vi.2016, Skelton, Carlson (IZAS). ***Paratypes***, female, as holotype, SAX 235 (MSUC, 1), SAX 248 (MSUC, 1).

##### Diagnosis.

1.6 mm long (mean = 1.6 mm; n = 2); 2.67× as long as wide. This species is distinguished by the elytral disc flat with short and steep obliquely truncate declivity; posterolateral carina denticulate; and declivital interstriae 2 and 3 strongly laterally broadened from base to declivital midpoint then narrowing towards apex.

##### Similar species.

*Microperus kirishimanus*, *M. nudibrevis*, *M. perparvus*.

##### Description

**(female).** 1.6 mm long (mean = 1.6 mm; n = 2); 2.67× as long as wide. Appearing bicolored: head, anterior slope of pronotum and elytra dark brown, remainder of pronotum, antennae, and legs light brown. ***Head***: epistoma entire, transverse, with a row of hair-like setae. Frons weakly convex to upper level of eyes, subshiny, punctate; punctures large, shallow, moderately dense, glabrous; punctures in lateral areas bearing a long, erect hair-like seta. Eyes shallowly emarginate just above antennal insertion, upper part smaller than lower part. Submentum large, distinctly triangular, slightly impressed. Antennal scape short and thick, shorter than club. Pedicel as wide as scape, shorter than funicle. Funicle 4-segmented, segment 1 shorter than pedicel. Club longer than wide, obliquely truncate, type 2; segment 1 corneous, transverse on anterior face, occupying basal 2/5 club, nearly covering posterior face; segment 2 narrow, soft; segment 1 present on posterior face. ***Pronotum***: 1.0× as long as wide. In dorsal view basic and parallel-sided, sides parallel in basal 3/4, rounded anteriorly; anterior margin without serrations. In lateral view elongate with disc much longer than anterior slope, type 8, summit low, at apical 2/5. Anterior slope with densely spaced, broad asperities, becoming lower and more strongly transverse towards summit. Disc shagreened, alutaceous, finely punctate, glabrous, some moderately long hair-like setae at margins. Lateral margins obliquely costate. Base weakly bisinuate, posterior angles acutely rounded, almost subquadrate. ***Elytra***: 1.78× as long as wide, 1.78× as long as pronotum. Scutellum minute, convex, slightly raised above elytral surface. Elytral mycangium present as a dispersed median setal tuft of setae extending along elytral base. Elytral base transverse, edge oblique, humeral angles rounded, parallel-sided in basal 4/5, then narrowly rounded to apex. Disc flat, subshiny, striae not impressed, with small shallow punctures separated by one diameter of a puncture, glabrous; interstriae flat, impunctate, setose, setae short, sparse, erect-hair-like. Declivity occupying 1/3 of elytral length, truncate, its margins denticulate, strongly shagreened, dull; striae flat, glabrous, impunctate; interstriae irregularly denticulate along their lengths, denticles uniformly sized, each bearing a short erect hair-like seta, interstriae 2 and 3 strongly laterally broadened from base to midpoint and then narrowed to apex. Posterolateral margin rounded, denticulate to interstriae 7. ***Legs***: procoxae contiguous; prosternal coxal piece tall, pointed. Protibiae slender, broadest at apical 1/3; posterior face smooth; apical 1/3 of outer margin with six moderate socketed denticles, their length approximately as long as basal width Meso- and metatibiae flattened; outer margin evenly rounded with seven and eight large socketed denticles, respectively.

##### Etymology.

L. *latus* = broad; *salebra* = rough road; -*inus* = likeness. Named in reference to the wide shagreened second declivital interstriae. An adjective.

##### Distribution.

China (Hong Kong).

##### Host plants.

*Castanopsis* (Fagaceae).

**Figure 68.**
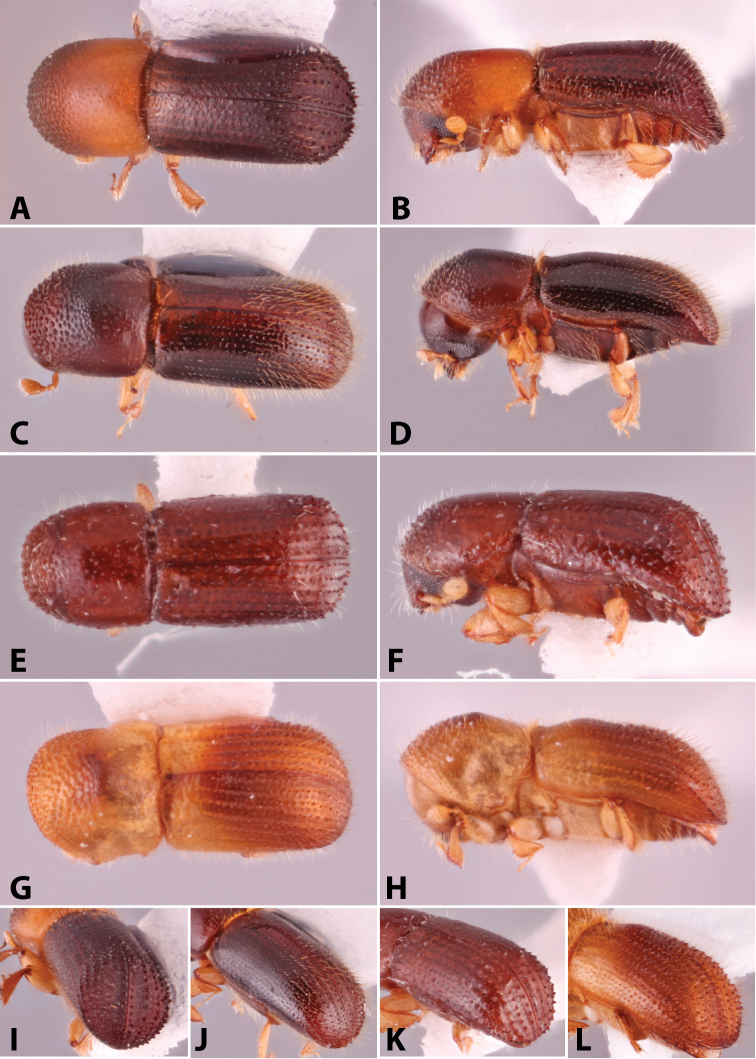
Dorsal, lateral and declivital view of *Microperus latesalebrinus* holotype, 1.6 mm (**A, B, I**), *M. minax* holotype, 1.95–2.0 mm (**C, D, J**), *M. nudibrevis*, 1.5–1.6 mm (**E, F, K**), and *M. nugax*, 1.8–1.9 mm (**G, H, L**).

#### Microperus minax sp. nov.

Taxon classificationAnimaliaColeopteraCurculionidae

http://zoobank.org/375928EE-6C48-47FB-BEEB-1ABCEA7204FE

Fig. 68C, D, J

##### Type material.

***Holotype***, female, Vietnam: Ninh Binh, Cuc Phuong N.P., 20.33296, 105.61259, 7.iii.2018, 279 m, A.I. Cognato, S.M. Smith, VN 141, ex 6 cm diameter branch (MSUC). ***Paratypes***, female, Vietnam: Ninh Binh, Cuc Phuong N.P., 20.34932, 105.59669, 431 m, 5.iii.2018, A.I. Cognato, S.M. Smith, VN 113b, ex *Terminalia myriocarpa*; large tree-fall trunk, 8 cm (NMNH, 1); Thua Thien-Hue, Bach Ma N.P., 16.19831, 107.85639, 1386 m, 17–18.ii.2017, VN69, A.I. Cognato, T.A. Hoang, ex 6 cm diameter branch (MSUC, 1).

##### Diagnosis.

1.95–2.0 mm long (mean = 1.98 mm; n = 2); 2.79–2.86× as long as wide. This species is distinguished by the declivital interstriae densely covered with long erect hair-like setae; elytral disc medially convex, appearing humped; declivity long, gradual; all declivital interstriae uniformly granulate from base to apex; declivital face convex; posterolateral carina granulate; antennal club truncate, type 2 with one suture visible on posterior face near apex; and declivity smooth, shiny.

This species is nearly identical to *M. intermedius* (Eggers, 1923) which has not been reported from the study region. *Microperus minax* is distinguished by the larger size (1.6–1.8 mm in *M. intermedius*) and the elytral disc longer, occupying 36–42% of total elytral length (30% in *M. intermedius*).

##### Similar species.

*Microperus diversicolor*, *M. kadoyamaensis*.

##### Description

**(female).** 1.95–2.0 mm long (mean = 1.98 mm; n = 2); 2.79–2.86× as long as wide. Body dark red-brown. Legs and antennae light brown. ***Head***: epistoma entire, transverse, with a row of hair-like setae. Frons weakly convex to upper level of eyes, subshiny, punctate, punctures small, shallow, moderately dense, glabrous; a few punctures in lateral areas bearing a long, erect hair-like seta. Eyes shallowly emarginate just above antennal insertion, upper part smaller than lower part. Submentum narrowly triangular, slightly impressed. Antennal scape short and thick, shorter than club. Pedicel as wide as scape, shorter than funicle. Funicle 4-segmented, segment 1 shorter than pedicel. Club longer than wide, obliquely truncate, type 2; segment 1 corneous, transverse on anterior face, occupying basal 2/5, nearly covering posterior face; segment 2 narrow, corneous; segment 1 present on posterior face. ***Pronotum***: 1.0× as long as wide. Basic and parallel-sided, type 2 in dorsal view, sides parallel in basal 2/3, rounded anteriorly; anterior margin without serrations. In lateral view elongate with disc slightly longer than anterior slope, type 7, disc flat, summit at apical 2/5. Anterior slope with densely spaced, broad asperities, becoming lower and more strongly transverse towards summit. Disc shagreened, alutaceous, impunctate, glabrous, some moderately long hair-like setae at margins. Lateral margins obliquely costate. Base transverse, posterior angles acutely rounded, almost subquadrate. ***Elytra***: 1.7× as long as wide, 1.7× as long as pronotum. Scutellum minute, convex, slightly raised above elytral surface. Elytral mycangium present as a dispersed median setal tuft of setae extending along elytral base to striae 3. Elytral base transverse, edge oblique, humeral angles rounded, parallel-sided in basal 9/10, then narrowly rounded to apex. Disc medially convex, appearing humped, shiny, striae not impressed, with small shallow punctures separated by 1–2 diameters of a puncture, setose, setae short, semi-erect, hair-like; interstriae flat, minutely punctate, setose, setae 2× as long as strial setae, erect, hair-like. Declivity occupying over 1/2 of elytral length, long, gradually rounded, face convex, shiny; striae flat, setose, setae as described for disc, impunctate; interstriae 1–3 parallel, interstriae densely covered with long, erect hair-like setae; all interstriae densely and uniformly granulate from base to apex, densely setose, setae as described for disc. Posterolateral margin carinate, granulate to interstriae 7. ***Legs***: procoxae contiguous; prosternal coxal piece tall, pointed. Protibiae slender, broadest at apical 1/3; posterior face smooth; apical 1/3 of outer margin with seven moderate socketed denticles, their length approximately as long as basal width. Meso- and metatibiae flattened; outer margin evenly rounded with 13 and 11 socketed denticles, respectively; basal two denticles longer than basal width, much larger than other denticles, remaining apical denticles very small, their length much shorter than basal width.

##### Etymology.

L. *minax* = threatening. In reference to the species habit of using live trees to host brood chambers. An invariable adjective.

##### Distribution.

Vietnam.

##### Host plants.

This species was collected from *Terminalia myriocarpa* (Combretaceae).

##### Remarks.

*Microperus minax* was collected from apparently healthy branches of living trees (SMS, AIC, personal observation) and may be an aggressive species.

#### Microperus nudibrevis

Taxon classificationAnimaliaColeopteraCurculionidae

(Schedl, 1942)

Fig. 68E, F, K

Xyleborus nudibrevis Schedl, 1942a: 195.
Coptodryas nudibrevis (Schedl): Wood and Bright 1992: 825.
Microperus nudibrevis (Schedl): Beaver et al. 2014: 55.

##### Type material.

***Holotype*** (NHMW).

##### New records.

China: Hong Kong, Tai Po Kau, vi.2017, J. Skelton (MSUC, 2). Japan: Okinawa, Yona, xi.2011, J. Hulcr, ex *Castanopsis* (MSUC, 1). Vietnam: Dong Nai, Cat Tien N.P., 11.42232, 107.42834, 128 m, 19.ii.2017, VN74, A.I. Cognato, T.A. Hoang, ex bottle trap (MSUC, 1).

##### Diagnosis.

1.5–1.6 mm long (mean = 1.53 mm; n = 5); 2.5–2.91× as long as wide. This species is distinguished by the elytral disc flat with short and steep obliquely truncate declivity; posterolateral carina strong and denticulate; declivital interstriae straight from base to apex, never laterally broadened; declivital interstriae densely granulate, granules uniformly sized and spaced from declivital summit to elytral apex; and declivital face opalescent, subshiny.

##### Similar species.

*Microperus kirishimanus*, *M. latesalebrinus*, *M. perparvus*.

##### Distribution.

China* (Hong Kong), Japan*, East & West Malaysia, Thailand, Vietnam*.

##### Host plants.

Recorded from five genera in five different families of trees, and presumably polyphagous (Beaver et al. 2014).

##### Remarks.

Browne (1961a) suggests that the female lays eggs in clusters over a considerable period, the offspring from each cluster occupying a separate brood chamber.

#### Microperus nugax

Taxon classificationAnimaliaColeopteraCurculionidae

(Schedl, 1939)

Fig. 68G, H, L

Xyleborus nugax Schedl, 1939a: 353.
Coptodryas nugax (Schedl): Wood 1989: 171.
Microperus nugax (Schedl): Hulcr 2010: 112.
Xyleborus pertuberculatus Eggers, 1940: 144. Synonymy: Kalshoven 1959a: 97.

##### Type material.

***Lectotype*** (NHMW).

##### New records.

Vietnam: Dong Nai, Cat Tien N.P., 11.42232, 107.42834, 128 m, 25.ii.2017, VN105, A.I. Cognato, T.A. Hoang, ex 10 cm diameter branch (MSUC, 2).

##### Diagnosis.

1.8–1.9 mm long (mean = 1.84 mm; n = 4); 2.57–2.71× as long as wide. This species is distinguished by the declivital interstriae densely covered with long erect hair-like setae; elytral disc medially convex, appearing humped; declivity long, gradual; all declivital interstriae uniformly granulate from base to apex; declivital face convex; posterolateral carina granulate; antennal club flat, type 3 with two sutures visible on posterior face; and declivity shagreened, dull.

##### Similar species.

*Microperus cruralis*, *M. sagmatus*, *M. undulatus*.

##### Distribution.

‘Borneo’, Brunei, Indonesia (Java, Sulawesi), East & West Malaysia, Thailand, Vietnam*.

##### Host plants.

Polyphagous (Browne 1961b).

#### Microperus perparvus

Taxon classificationAnimaliaColeopteraCurculionidae

(Sampson, 1922)

Fig. 69A, B, I

Xyleborus perparvus Sampson, 1922b: 151.
Microperus perparvus (Sampson): Maiti and Saha 1986: 97.
Coptodryas perparva (Sampson): Wood and Bright 1992: 826.
Xyleborus tsukubanus Murayama, 1954: 184. Synonymy: Beaver et al. 2008: 233.

##### Type material.

***Syntypes****Xyleborus perparvus* (NHMUK).

##### New records.

China: Hong Kong, Tai Po Kau, vi.2017, J. Skelton (MSUC, 1). Jiangxi, Long Nan, 10.vii.2016, Lv-Jia, Lai, S-C., ex *Eriobotrya japonica* (RABC, 1). Sichuan, Mt. Emei, 18.viii.2016, Tian-Shang (RABC, 1). S Yunnan, Xishuangbanna, 20 km NW Jinghong, vic. Man Dian (NNNR), 22°07.80'N, 100°40.05'E, 740 m, fallow, 18.vi.2008, A. Weigel (RABC, 1); as previous except: forest, 28.vi.2008 (RABC, 1); as previous except: 730 m, forest, EKL, 6.iv.2009, L. Meng (RABC, 6); as previous except: 23 km NW Jinghong, vic. Na Ban (NNNR), 22°09.49'N, 100°39.92'E, rubber plantation, 730 m, 15.vi.2008, A. Weigel (RABC, 2); as previous except: 25 km NW Jinghong, vic. Zhang Zhi Chang (NNNR), 22°11.06'N, 100°39.05'E, 780 m, rubber plantation, EKL, 15.vi.2008, A. Weigel (RABC, 2). Vietnam: Cao Bang, 22°36.454'N, 105°52.083'E, 1661 m, 15.iv.2014, VN38, Cognato, Smith, Pham, ex 1–3 cm diameter branch and twig (MSUC, 2). Dong Nai, Cat Tien N.P., 11.40817, 107.38098, 134 m, 22–24.ii.2017, VN81, A.I. Cognato, T.A. Hoang, ex FIT (MSUC, 1).

##### Diagnosis.

1.5–1.9 mm long (mean = 1.64 mm; n = 5); 2.71–2.86× as long as wide. This species is distinguished by the elytral disc flat with short and steep obliquely truncate declivity; posterolateral carina weak, denticulate; declivital interstriae straight from base to apex, never laterally broadened; denticles on declivital summit denser and of equal size to those on declivital face; declivital face shagreened, dull.

##### Similar species.

*Microperus kirishimanus*, *M. latesalebrinus*, *M. nudibrevis*.

##### Distribution.

Bangladesh, China (Fujian, Guizhou, Hong Kong*, Hunan, Jiangxi*, Sichuan*, Xizang, Yunnan*), India (Andaman Is, Assam, West Bengal), Indonesia (Ternate), Japan, East & West Malaysia, Myanmar, New Guinea, Solomon Islands, Taiwan, Thailand, Vietnam*.

##### Host plants.

Polyphagous, possibly with some preference for Dipterocarpaceae (Beaver and Liu 2010).

**Figure 69.**
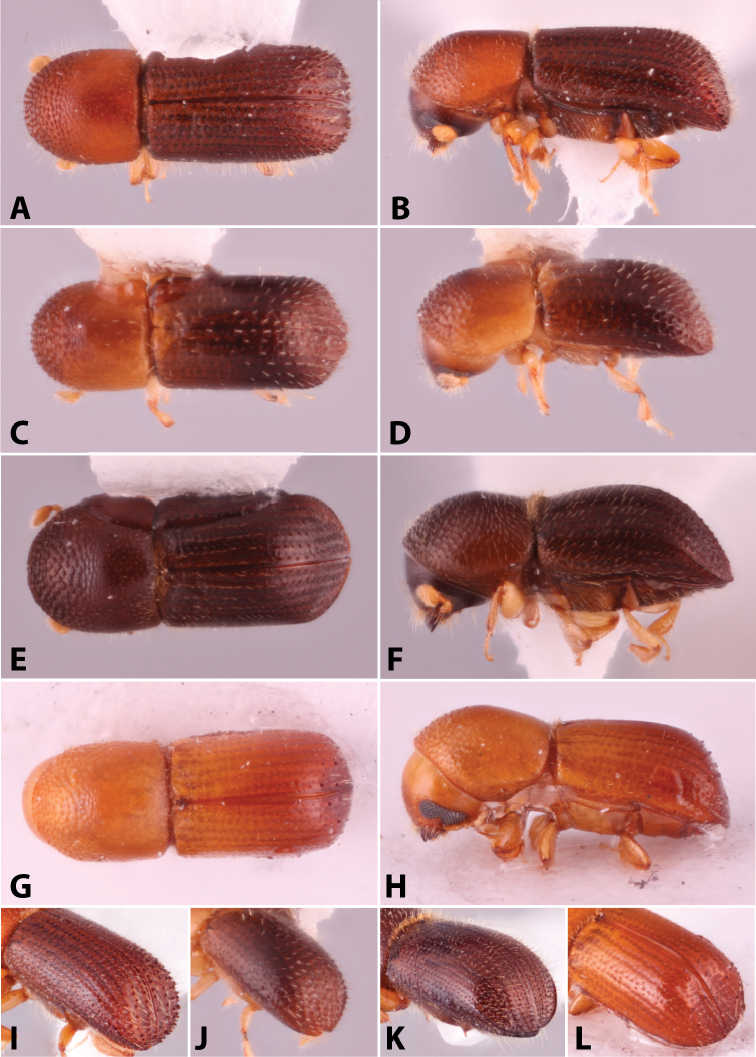
Dorsal, lateral and declivital view of *Microperus perparvus*, 1.5–1.9 mm (**A, B, I**), *M. pometianus*, 1.2–1.7 mm (**C, D, J**), *M. quercicola*, 1.8–2.0 mm (**E, F, K**), and *M. recidens*, 1.9–2.0 mm (**G, H, L**).

#### Microperus pometianus

Taxon classificationAnimaliaColeopteraCurculionidae

(Schedl, 1939)

Fig. 69C, D, J

Xyleborus pometianus Schedl, 1939a: 354.
Microperus pometianus (Schedl): Hulcr and Cognato 2010a: 21.

##### Type material.

***Lectotype*** (NHMW).

##### Diagnosis.

1.2–1.7 mm long (mean = 1.44 mm; n = 5); 2.6–3.0× as long as wide. This species is distinguished by the elytral disc flat; declivity short, steep; declivity granulate from base to apex, granules small, as abundant as strial punctures; granules dispersed, separated by the width of at least three granules; declivital surface shagreened; interstriae moderately setose, setae short semi-erect bristles, less than the width of an interstria; striae glabrous; and minute size.

##### Similar species.

*Microperus quercicola*.

##### Distribution.

East & West Malaysia, New Guinea, Philippines, Thailand.

##### Host plants.

Recorded from *Nephelium*, *Pometia*, *Xerospermum* (Sapindaceae), and an unidentified genus of Burseraceae (Schedl 1942a; Browne 1961b). An association with Sapindaceae is suggested by Browne (1961b), but there are too few records to be sure of this.

#### Microperus quercicola

Taxon classificationAnimaliaColeopteraCurculionidae

(Eggers, 1926)

Fig. 69E, F, K

Xyleborus quercicola Eggers, 1926: 146.
Microperus quercicola (Eggers): Smith et al. 2018b: 396.
Xyleborus izuensis Murayama, 1952: 16. Synonymy: Smith et al. 2018b: 396.

##### Type material.

***Holotype****Xyleborus quercicola* (NMNH).

##### New records.

China: Guizhou, Zunyi, 28.x.2015, Y. Li, ex *Cinnamomum camphora* (MSUC, 5). Hong Kong, Tai Po Kau, vi.2017, J. Skelton, ex Lauraceae (MSUC, 1). Jiangxi, Nanchang, 12.iii.2018, Y. Li (UFFE, 1). Sichuan, Chengdu, 570 m; 8.vi.1960, Huifen Yin, *Cinnamomum* 78 (NMNH, 1). Zhejiang, Fuyang, 11.iv.1984, Guangpu Shen, *Cinnamomum* sp. (NMNH, 2). Taiwan: Nantou, Huisun, 24.x.2017, Y-T Huang, J. Hulcr, ex *Diospyros morrisiana* (MSUC, 1); as previous except: Zhushan, 11.vii.2017, C.-S. Lin (MSUC, 1).

##### Diagnosis.

1.8–2.0 mm long (mean = 1.96 mm; n = 5); 2.38–2.86× as long as wide. This species is distinguished by the elytral disc flat; declivity short, steep; declivity granulate from base to apex, granules small, as abundant as strial punctures; granules dense, separated by the width of one granule; declivital surface shiny; posterolateral costa strongly carinate; interstriae densely setose, setae fine, hair-like as long as the width of an interstria; and strial punctures setose, setae recumbent, hair-like, less than a strial width.

##### Similar species.

*Microperus kadoyamaensis*, *M. pometianus*.

##### Distribution.

China* (Guizhou, Hong Kong*, Jiangxi, Sichuan, Zhejiang), Japan, Russia (Far East), South Korea, Taiwan*.

##### Host plants.

This species is polyphagous and has been recorded from *Cinnamomum* (Lauraceae) (Murayama 1952), *Diospyros* (Ebenaceae), *Fraxinus* (Oleaceae), *Carpinus* (Betulaceae) (Mandelshtam et al. 2018) and “oak trees” (Fagaceae) (Eggers 1926).

#### Microperus recidens

Taxon classificationAnimaliaColeopteraCurculionidae

(Sampson, 1923)

Fig. 69G, H, L

Xyleborus recidens Sampson, 1923: 287.
Coptodryas recidens (Sampson): Wood 1989: 171.
Microperus recidens (Sampson): Beaver et al. 2014: 56.
Xyleborus minusculus Eggers, 1923: 212. Synonymy: Eggers 1925: 154.
Xyleborus minutissimus Eggers, 1930: 204. Synonymy: Wood 1989: 171.
Xyleborus crassitarsus Schedl, 1936d: 28. Synonymy: Browne 1955: 364.
Xyleborus artegraphus Schedl, 1942c: 44. Synonymy: Hulcr and Cognato 2013: 111.
Xyleborus extensus Schedl, 1955a: 301. Synonymy: Hulcr and Cognato 2013: 111.
Xyleborus tuberculosus Browne, 1981b: 602. Synonymy: Beaver 1995a: 198.

##### Type material.

***Lectotype****Xyleborus minusculus* (NMNH). ***Syntypes****Xyleborus recidens* (NHMUK). ***Holotype***, ***paratype****Xyleborus tuberculosus* (NHMUK).

##### New records.

China: Jiangxi, Xunwu, Xingshan, 6.ix.2018, Y. Li, ex Fagaceae log (UFFE, 1). S Yunnan, Xishuangbanna, 23 km NW Jinghong, vic. Na Ban village (NNNR), 22°10'N, 100°39'E, 700–1000 m, v–vii. 2009, L. Meng (RABC, 1). Vietnam: Dong Nai, Cat Tien N.P., 11.42232, 107.42834, 128 m, 19.ii.2017, VN74, A.I. Cognato, T.A. Hoang, ex porch light (MSUC, 1).

##### Diagnosis.

1.9–2.0 mm long (mean = 1.97 mm; n = 5); 2.71–2.86× as long as wide. This species is distinguished by the elytral disc flat with short, steep declivity; posterolateral margin of elytra carinate; declivity with sparse minor denticles, much less abundant than strial punctures; and a pair of slightly larger denticles on interstriae 3.

##### Similar species.

*Microperus alpha*.

##### Distribution.

Bangladesh, ‘Borneo’, Brunei, China* (Jiangxi, Yunnan), India (Andaman Is, West Bengal), Indonesia (Engano I., Java, Maluku), East & West Malaysia, Myanmar, New Guinea, Philippines, Thailand, Vietnam*.

##### Host plants.

Polyphagous (Beeson 1961).

#### Microperus sagmatus sp. nov.

Taxon classificationAnimaliaColeopteraCurculionidae

http://zoobank.org/F8082757-4BED-4036-BE76-64A89A26FB2D

Fig. 70A, B, E

##### Type material.

***Holotype***, female, Thailand: Suranthani [= Surat Thani], durian or[chard], 01.xii.[20]10, Wisut Sittichaya, EToH-trap (MSUC). ***Paratypes***, female, Malaysia: Penang, B[atu] Ferringhi, 6.i–1.ii.1981, T. Palm (RABC, 1); Thailand: as holotype (MSUC, 1); Prachuab Khiri Khan: Kui Buri N.P., 27.iii.2008, S. Stevens et al., ex ‘krachid’ (NHMUK, 2; MSUC, 2; QSBG, 1; RABC, 2); Songkhla, Ratthapum distr., ex durian branch, 4.xi.2008, W. Sittichaya (RABC, 1; QSBG 1).

##### Diagnosis.

1.75–1.95 mm long (mean = 1.83 mm; n = 5); 2.69–2.79× as long as wide. This species is distinguished by the elytral disc shallowly transversely impressed with a saddle-like depression; elytral interstriae costate with strong interstrial spines posterior to the saddle; and declivity steep, slightly flattened.

*Microperus sagmatus* closely resembles *M. undulatus* but is distinguished by the discal impression deeper, antero-posteriorly narrower, with steeper anterior and posterior slopes, strial punctures on impression with rounded granules, interstrial spines on disc behind saddle stronger, and backwardly hooked, not pointing dorsally.

##### Similar species.

*Microperus cruralis*, *M. nugax*, *M. undulatus*.

##### Description

**(female).** 1.75–1.95 mm long (mean = 1.83 mm; n = 5); 2.69–2.79 × as long as wide. Body ferruginous. Legs and antennae light brown. ***Head***: epistoma entire, transverse, with a row of hair-like setae. Frons weakly convex to upper level of eyes, shagreened, punctate; punctures large, shallow, sparse, setose; punctures bearing a long, erect hair-like seta. Eyes shallowly emarginate just above antennal insertion, upper part smaller than lower part. Submentum large, distinctly triangular, slightly impressed. Antennal scape short and thick, as long as club. Pedicel as wide as scape, shorter than funicle. Funicle 4-segmented, segment 1 shorter than pedicel. Club longer than wide, obliquely truncate, type 2; segment 1 corneous, feebly convex on anterior face, occupying basal 1/3, nearly covering posterior face; segment 2 narrow, soft; segment 1 present on posterior face. ***Pronotum***: 1.05× as long as wide. In dorsal view subquadrate and parallel-sided, type 3, sides parallel in basal 2/3, weakly rounded anteriorly with prominent anterolateral corners; anterior margin without a row of serrations. In lateral view tall, type 2, disc flat, summit at midpoint. Anterior slope with densely spaced, broad asperities, becoming lower and more strongly transverse towards summit. Disc shagreened, alutaceous, impunctate, glabrous, some moderately long hair-like setae at margins. Lateral margins obliquely costate. Base weakly bisinuate, posterior angles acutely rounded, almost subquadrate. ***Elytra***: 1.7× as long as wide, 1.6× as long as pronotum. Scutellum minute, convex, slightly raised above elytral surface. Elytral mycangium present as a dispersed median setal tuft of setae extending along elytral base. Elytral base transverse, edge oblique, humeral angles rounded, parallel-sided in basal 4/5, then narrowly rounded to apex. Disc shiny, a moderately deep transverse saddle-like impression at midpoint, striae and interstriae flat, nearly glabrous anteriad of depression, strial punctures on impression with rounded granules, interstriae costate with strong backwardly hooked spines posteriad of depression, spines setose with long hair-like setae. Declivity occupying 1/3 of elytral length, shagreened, dull, steeply rounded, face slightly flattened; striae flat, parallel, punctate, punctures very large, shallow subcontiguous, setose, setae recumbent, as long a puncture; interstriae irregularly denticulate along their lengths, denticles small, irregularly spaced and sized, each bearing a long, erect hair-like seta, interstriae 1 and 3, weakly convex, 2 and 4 flat. Posterolateral margin carinate to interstriae 7. ***Legs***: procoxae contiguous; prosternal coxal piece tall, conical. Protibiae obliquely triangular, broadest at apical 1/3; posterior face smooth; apical 1/3 of outer margin with seven moderate socketed denticles, their length approximately as long as basal width. Meso- and metatibiae flattened; outer margin evenly rounded with nine and eight moderate to large socketed denticles, respectively.

##### Etymology.

G. *sagma* = pack-saddle. In reference to the shape of the elytra. An adjective.

##### Distribution.

West Malaysia, Thailand.

##### Host plants.

Recorded from *Durio zibethinus* (durian) (Malvaceae), and an undetermined tree, ‘krachid’.

##### Remarks.

The specimens from Thailand were included under *M. undulatus* by Beaver et al. (2014).

**Figure 70.**
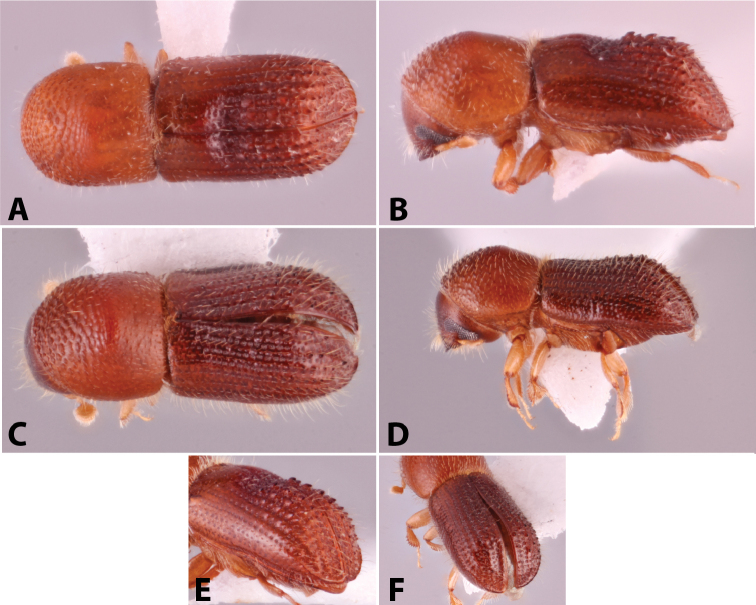
Dorsal, lateral and declivital view of *Microperus sagmatus* holotype, 1.75–1.95 mm (**A, B, E**), and *M. undulatus*, 2.0–2.1 mm (**C, D, F**).

#### Microperus undulatus

Taxon classificationAnimaliaColeopteraCurculionidae

(Sampson, 1919)

Fig. 70C, D, F

Xyleborus undulatus Sampson, 1919: 111.
Coptodryas undulata (Sampson): Wood 1989: 171.
Microperus undulatus (Sampson): Saha and Maiti 1996: 827.
Xyleborus leprosulus Schedl, 1936d: 27. Synonymy: Wood 1989: 171.

##### Type material.

***Holotype****Xyleborus undulatus* (NHMUK).

##### Diagnosis.

2.0–2.1 mm long (mean = 2.03 mm; n = 3); 2.5–2.86× as long as wide. This species is distinguished by the elytral disc shallowly transversely impressed with a saddle-like depression; elytral interstriae flat with moderate interstrial tubercles posterior to the saddle; and declivity steep, slightly flattened.

*Microperus undulatus* closely resembles *M. sagmatus* but is distinguished by the discal impression shallower, antero-posteriorly broader, with gentler anterior and posterior slopes, strial punctures on impression without granules, interstrial tubercles on disc behind saddle moderately sized with rounded apices pointing dorsally, not backwardly hooked.

##### Similar species.

*Microperus cruralis*, *M. nugax*, *M. sagmatus*.

##### Distribution.

India (West Bengal), Indonesia (Java), West Malaysia, Nepal, Thailand.

##### Host plants.

Recorded only from two species of *Shorea* (Dipterocarpaceae) (Beeson 1930; Browne 1961b).

### *Planiculus* Hulcr & Cognato, 2010

#### Planiculus

Taxon classificationAnimaliaColeopteraCurculionidae

Hulcr & Cognato, 2010

Planiculus Hulcr & Cognato, 2010a: 21.

##### Type species.

*Xyleborus bicolor* Blandford, 1894b; original designation.

##### Diagnosis.

Minute to small (1.7–2.4 mm), elongate (2.57–3.6× as long as wide) and distinctly bicolored species. *Planiculus* is distinguished by the declivity flat, slightly broadened laterally; declivital interstriae 1 laterally broadened; lateral profile of declivity gradually descending; pronotum from dorsal view long, rounded anteriad (type 9), from lateral view elongated with low summit and elongate disc (type 8); antennal club approximately circular, obliquely truncate, type 2, segment 1 corneous, large, occupying at least basal 1/2 of club, segment 2 visible on posterior face; and protibiae distinctly triangular. In addition, the procoxae are contiguous, scutellum visible, flat, flush with elytra, the tuft on pronotal base associated with mesonotal mycangium is absent.

*Planiculus* species are most easily confused with small *Euwallacea* and *Xyleborus* species but are distinguished by the declivity flat, slightly broadened laterally, with very few tubercles and smaller more elongate body, and rounded frontal margin of pronotum (type 7) that is never subquadrate (as in *Euwallacea*).

##### Similar genera.

*Euwallacea*, *Xyleborus*.

##### Distribution.

Found throughout the Paleotropics and Australasia.

##### Gallery system.

The gallery system has a few branches more or less in one transverse plane with several small brood chambers extending longitudinally. Surface galleries between the phloem and sapwood may occur in thick-barked stems (Browne 1961b).

#### Key to *Planiculus* species (females only)

**Table:** 

1	Elytral apex entire (Fig. 71A)	*** bicolor ***
–	Elytral apex emarginate (Fig. 71C)	**2**
2	Declivital interstriae 1 armed with several granules	*** limatus ***
–	Declivital interstriae 1 armed with one tubercle	*** shiva ***

#### Planiculus bicolor

Taxon classificationAnimaliaColeopteraCurculionidae

(Blandford, 1894)

Fig. 71A, B, E

Xyleborus bicolor Blandford, 1894b: 113.
Euwallacea bicolor (Blandford): Wood 1989: 172.
Planiculus bicolor (Blandford): Hulcr and Cognato 2010a: 22.
Xyleborus laevis Eggers, 1923: 201. Synonymy: Hulcr and Cognato 2010a: 22.
Xyleborus bicolor unimodus Beeson, 1929: 238. Synonymy: Wood 1989: 172.
Xyleborus rodgeri Beeson, 1930: 213. Synonymy: Wood 1989: 173.
Xyleborus rodgeri privatus Beeson, 1930: 213. Synonymy: Wood 1989: 173.
Xyleborus rameus Schedl, 1940a: 441. Synonymy: Kalshoven 1959b: 141.
Xyleborus artelaevis Schedl, 1942a: 196. Synonymy: Hulcr 2010: 113.
Xyleborus ashuensis Murayama, 1954: 193. Synonymy: Smith et al. 2018b: 396.
Xyleborus filiformis Schedl, 1975c: 364. Synonymy: Hulcr and Cognato 2010a: 22.
Xyleborus tumidus Schedl, 1975c: 371. Synonymy: Hulcr and Cognato 2010a: 22.
Xyleborus glabratulus Browne, 1983a: 560. Synonymy: Hulcr and Cognato 2010a: 22.

##### Type material.

***Syntypes****Xyleborus bicolor* (NHMUK). ***Holotype****Xyleborus bicolor unimodus* (NHMUK), ***paratypes*** (BPBM, 2). ***Holotype****Xyleborus glabratulus* (NHMUK). ***Lectotype****Xyleborus laevis* (NMNH). ***Syntype****Xyleborus rameus* (NHMW). ***Holotype****Xyleborus rodgeri* (FRI).

##### New records.

China: Hainan, Ledong, Jian Feng Natl For. Park, 18.700N, 109.080E, 133 m, 4.xii.2016, Tian-Shang, Lv-Jia (RABC, 1). Jiangxi, Long Nan, 12.vii.2016, Lv-Jia, Lai, S-C., ex *Cyclobalanopsis glauca* (RABC, 1). Laos: Bolikhamxai, Ban nape (8 km NE), 18°21'N, 105°08'E, 600 m, 1–18.v.2001, V. Kubáň (NHMB, 6; RABC, 2). NE, Houa Phan, Phou Pane mt., 20°13'09–19"N, 103°59'54"–104°00'03"E, 1480–1510 m, 22.iv–14.v.2008, V. Kubáň (RABC, 1). Kham Mouan, Ban Khun Ngeun, 18°07'N, 104°29'E, ~ 200 m, 24–29.iv.2001, Pacholátko (NHMB, 1). Louangphrabang, Thong Khan, 19°35'N, 101°58'E, ~ 750 m, 11–21.v.2002, V. Kubáň (NHMB, 7; RABC, 1). Oudomxai, Oudomxai, 17 km NE, 20°45'N, 102°09'E, ~ 1100 m, 1–9.v.2002, V. Kubáň (NHMB, 1). Vietnam: Dong Nai, Cat Tien National Park, near park headquarters, 11°25'44"N, 107°25'44"E, 120 m, 26–31.v.1999, B. Hubley, D. Currie, VIET1H95-99 041, ex flight intercept trap (SEMC, 3); as previous except: 11.42854, 107.42544, 148 m, 23.ii.2017, VN99, A.I. Cognato, T.A. Hoang, ex 2–3 cm diameter branches (MSUC, 66).

##### Diagnosis.

1.8–2.4 mm long (mean = 2.05 mm; n = 5); 2.86–3.6× as long as wide. This species is distinguished by its rounded elytral apex.

##### Similar species.

*Planiculus limatus*, *P. shiva*.

##### Distribution.

American Samoa, Bangladesh, ‘Borneo’, China (Hainan*, Jiangxi*, Yunnan), Federated States of Micronesia, Fiji, India (Andaman Is, Assam, Nicobar Is, Tamil Nadu, Uttarakhand, West Bengal), Indonesia (Java, Sumatra), Japan, Laos*, East & West Malaysia, Myanmar, Nepal, New Caledonia, New Guinea, Philippines, Samoa, Seychelles, Solomon Islands, Sri Lanka, Thailand, Vietnam*.

##### Host plants.

Polyphagous (e.g., Beeson 1961; Browne 1961b; Ohno et al. 1988).

**Figure 71.**
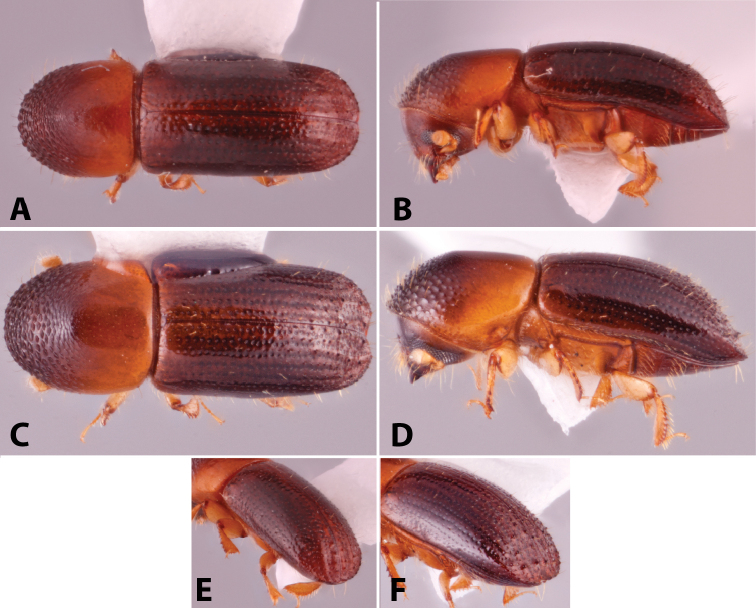
Dorsal, lateral and declivital view of *Planiculus bicolor*, 1.8–2.4 mm (**A, B, E**), and *P. limatus*, 1.7–2.2 mm (**C, D, F**).

#### Planiculus limatus

Taxon classificationAnimaliaColeopteraCurculionidae

(Schedl, 1942)

Fig. 71C, D, F

Xyleborus limatus Schedl, 1942b: 171.
Planiculus limatus (Schedl): Hulcr and Cognato 2010a: 23.
Xyleborus subemarginatus Eggers, 1940: 150. Synonymy: Hulcr and Cognato 2010a: 23.
Xyleborus subparallelus Eggers, 1940: 151. Synonymy: Hulcr and Cognato 2010a: 23.

##### Type material.

***Holotype****Xyleborus limatus* (NHMW). ***Lectotype****Xyleborus subemarginatus* (NMNH). ***Lectotype****Xyleborus subparallelus* (NMNH).

##### New records.

Japan: Okinawa Pref., Iriomote-jima Island, 2.xi.2016, H. Kajimura, ex *Machilus thunbergii* (MSUC, 1). Vietnam: Dong Nai, Cat Tien N.P., 11.46050, 107.37375, 379 m, 20.ii.2017, VN77, A.I. Cognato, T.A. Hoang, ex 45 cm diameter buttressed tree (MSUC, 45).

##### Diagnosis.

1.7–2.2 mm long (mean = 1.9 mm; n = 5); 2.57–2.83× as long as wide. This species is distinguished by the emarginate elytral apex and declivital interstriae 1 armed with several granules.

##### Similar species.

*Planiculus bicolor*, *P. shiva*.

##### Distribution.

Indonesia (Java), Japan*, East & West Malaysia, New Guinea, Philippines, Thailand, Vietnam*.

##### Host plants.

Polyphagous (Browne 1961b).

#### Planiculus shiva

Taxon classificationAnimaliaColeopteraCurculionidae

(Maiti & Saha, 1986) comb. nov.

Xyleborus shiva Maiti & Saha, 1986: 140.

##### Type material.

***Holotype*** (ZSI). Not examined.

##### Distribution.

India (Andaman Is).

##### Diagnosis.

1.85 mm long. Length/width ratio unknown. This species is distinguished by the emarginate elytral apex and “declivital interstriae 1 somewhat raised below the middle accommodating one distinct setiferous tubercle” (Maiti and Saha 1986).

##### Similar species.

*Planiculus bicolor*, *P. limatus*.

##### Host plants.

Recorded only from *Pterocymbium* (Malvaceae) (Maiti and Saha 1986).

##### Remarks.

Specimens of this species were unavailable for study. The diagnosis and measurements were taken from Maiti and Saha’s (1986) description and illustration. The authors determined *P. shiva* to be very closely related to *P. bicolor*. The species is transferred to *Planiculus* because of the following characters: declivity flat, slightly broadened laterally, with very few tubercles and smaller more elongate body, rounded frontal margin of pronotum (type 7) and bicolored body.

#### Pseudowebbia

Taxon classificationAnimaliaColeopteraCurculionidae

Browne, 1961

Pseudowebbia Browne, 1961a: 308.

##### Type species.

*Xyleborus trepanicauda* Eggers, 1923; original designation.

##### Diagnosis.

2.2–3.1 mm, elongate species, 2.4–3.1× as long as wide. *Pseudowebbia* is distinguished by the scutellum not visible; dense tuft of setae along elytral base associated with an elytral mycangium; antennal funicle 4-segmented; declivity truncate, covered with dense scales and encircled by a row of denticles; and protibiae with evenly rounded edge, lateral margin armed with seven socketed denticles, posterior face flat, unarmed.

##### Similar genera.

*Arixyleborus*, *Cyclorhipidion*, *Truncaudum*, *Webbia*.

##### Distribution.

Occurring throughout the Paleotropics.

##### Gallery system.

Described only for *P. percorthylus* (Schedl, 1935). The short entrance tunnel runs into an irregular cavity lying between the bark and wood, with or without some short side branches. In the observed systems, the gallery system does not penetrate the wood (Browne 1961b; RAB pers. obs.).

#### Pseudowebbia trepanicauda

Taxon classificationAnimaliaColeopteraCurculionidae

(Eggers, 1923)

Fig. 72

Xyleborus trepanicauda Eggers, 1923: 170.
Pseudowebbia trepanicauda (Eggers): Browne 1961a: 308.

##### Type material.

***Syntypes*** (RMNH, MCG). Not examined.

##### Diagnosis.

2.2–3.1 mm long (mean = 2.51 mm; n = 6); 2.4–3.1× as long as wide. This species is distinguished by 1–3 moderately sized denticles on declivital interstriae 2; and pronotum anterior margin basic, short, rounded and parallel-sided, when viewed dorsally (type 2).

##### Similar species.

*Arixyleborus*, truncate *Cyclorhipidion* species, *Truncaudum*, *Webbia*.

##### Distribution.

‘Borneo’, Brunei, Indonesia (Sumatra), East Malaysia, Thailand, Vietnam.

##### Host plants.

Recorded only from *Vatica* (Dipterocarpaceae) (Browne 1961a).

##### Remarks.

The number of tubercles on the declivity can be very variable.

**Figure 72.**
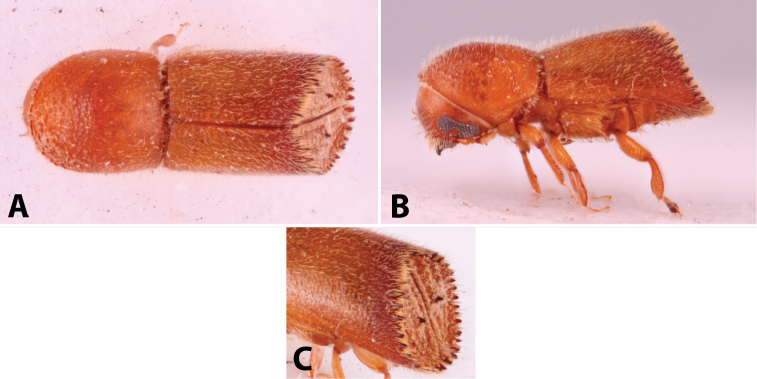
Dorsal, lateral and declivital view of *Pseudowebbia trepanicauda*, 2.2–3.1 mm (**A–C**).

### *Schedlia* Browne, 1950

#### Schedlia

Taxon classificationAnimaliaColeopteraCurculionidae

Browne, 1950

Schedlia Browne, 1950b: 641.

##### Type species.

*Xyleborus sumatranus* Hagedorn, 1908; original designation.

##### Diagnosis.

*Schedlia* species are large and stout (4.2–5.3 mm; 2.15–2.5× as long as wide) and distinguished by the scutellum absent; elytral disc minutely rugose and punctate; declivity clearly distinct from disc, obliquely truncate, impunctate, coarsely granulate to tuberculate; elytral bases costate, curved, with conspicuous medial tufts of setae denoting an elytral mycangium; antennal club flattened, type 4, pubescent; pronotum type 4 in lateral view; protibiae sickle-like, inflated and granulate on posterior face; and procoxae contiguous.

*Schedlia* can be distinguished from *Ambrosiodmus* by the lack of scutellum, and from *Coptodryas* by the declivity clearly separated from disc.

##### Similar genera.

*Ambrosiodmus*, *Coptodryas*.

##### Distribution.

Paleotropical.

##### Gallery system.

The unbranched radial entrance tunnel leads to a single large brood chamber in the longitudinal plane (Browne 1961b).

##### Remarks.

*Schedlia* species are Dipterocarpaceae specialists.

#### Key to *Schedlia* species (females only)

**Table:** 

1	Declivity without a pair of large spines on basal 1/3; smaller, 4.2–4.65 mm	*** allecta ***
–	Declivity with a pair of large spines on basal 1/3; larger, 4.8–5.3 mm	*** sumatrana ***

#### Schedlia allecta

Taxon classificationAnimaliaColeopteraCurculionidae

(Schedl, 1942)

Fig. 73A, B, E

Xyleborus allectus Schedl, 1942c: 33.
Schedlia allecta (Schedl): Browne, 1950: 642.

##### Type material.

***Holotype*** (NHMW).

##### New records.

Cambodia: Pursat, Phnom Samkos Wildlife Sanctuary, Pramsoy, forest edge, 16.xi.2005, K. Smets, I. Var, light trapping (IRSNB, 5; RABC, 1).

##### Diagnosis.

4.2–4.65 mm long (mean = 4.45 mm; n = 5); 2.15–2.5× as long as wide. This species is clearly distinguished from *S. sumatrana* by the lack of a pair of large spines on basal 1/3 of declivity; and smaller size.

##### Similar species.

*Schedlia sumatrana*.

##### Distribution.

Brunei, Cambodia*, Thailand, Vietnam.

##### Host plants.

Unknown, but likely a Dipterocarpaceae specialist.

**Figure 73.**
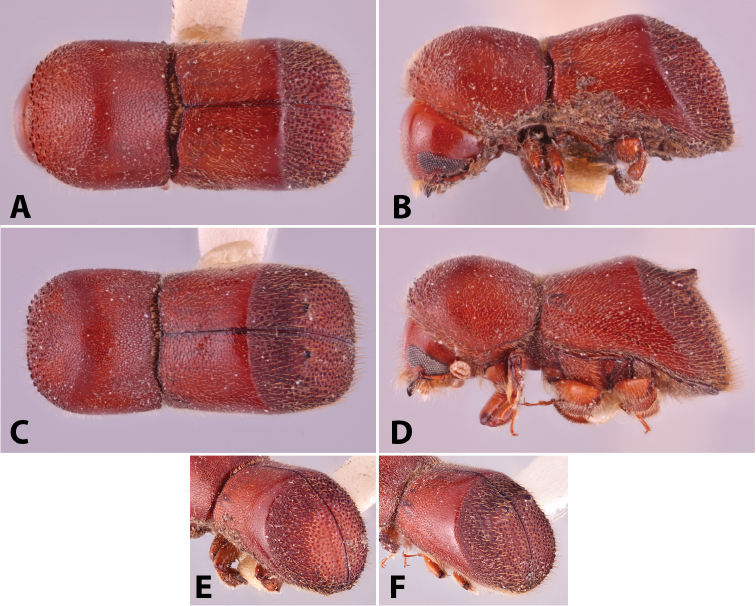
Dorsal, lateral and declivital view of *Schedlia allecta* holotype, 4.2–4.65 mm (**A, B, E**), and *S. sumatrana*, 4.8–5.3 mm (**C, D, F**).

#### Schedlia sumatrana

Taxon classificationAnimaliaColeopteraCurculionidae

(Hagedorn, 1908)

Fig. 73C, D, F

Xyleborus sumatranus Hagedorn, 1908: 381.
Schedlia sumatrana (Hagedorn): Browne 1950: 642.

##### Type material.

The holotype was destroyed in the bombing of UHZM in World War II (Wood and Bright 1992).

##### Diagnosis.

4.8–5.3 mm long (mean = 4.96 mm; n = 5); 2.18–2.41× as long as wide. This species can be diagnosed by the larger size; and a pair of large spines on basal 1/3 of declivity.

##### Similar species.

*Schedlia allecta*.

##### Distribution.

Indonesia (Sumatra), East & West Malaysia, Thailand, Vietnam.

##### Host plants.

The species has been recorded only from trees of the family Dipterocarpaceae (*Balanocarpus*, *Dipterocarpus*, *Dryobalanops*, *Hopea*, *Shorea*, *Vatica*) (Browne 1961a, c).

##### Remarks.

Browne (1961b) notes that broods tend to be small (from 8–18 individuals), and that the life cycle may be completed in 3–4 weeks.

#### Stictodex

Taxon classificationAnimaliaColeopteraCurculionidae

Hulcr & Cognato, 2013

Stictodex Hulcr & Cognato, 2013: 123.

##### Type species.

*Xyleborus dimidiatus* Eggers, 1927a: original designation.

##### Diagnosis.

Moderately sized, 2.4–3.3 mm, elongate, 2.54–2.89× as long as wide, and shiny species. *Stictodex* is distinguished by the antennal club very broad, type 2, with segment 1 straight; declivity with first and second interstriae divergent, broadest at elytral summit; declivity flat and gradually sloped; scutellum flat, flush with elytra; protibiae inflated on posterior face; and procoxae contiguous.

*Stictodex* is similar to *Arixyleborus* with which it shares a broad antennal club but it lacks the distinctive elytral ridges and furrows.

##### Similar genera.

*Arixyleborus*, *Fraudatrix*, *Xyleborus*.

##### Distribution.

Paleotropical.

##### Gallery system.

Not described.

#### Stictodex dimidiatus

Taxon classificationAnimaliaColeopteraCurculionidae

(Eggers, 1927)

Fig. 74

Xyleborus dimidiatus Eggers, 1927a: 404.
Stictodex dimidiatus (Eggers): Hulcr and Cognato 2013: 125.
Xyleborus dorsosulcatus Beeson, 1930: 219. syn. nov.
Xyleborus tunggali Schedl, 1936d: 32. Synonymy: Hulcr and Cognato 2013: 125.
Xyleborus decumans Schedl, 1953b: 301. Synonymy: Hulcr and Cognato 2013: 125.
Xyleborus cruciatus Schedl, 1973: 90. Synonymy: Hulcr and Cognato 2013: 125.

##### Type material.

***Paratype****Xyleborus dimidiatus* (NMNH,1). ***Holotype****Xyleborus dorsosulcatus* (FRI). ***Lectotype****Xyleborus tunggali* (NHMW).

##### New records.

Laos: Kham Mouan, Ban Khun Ngeun, 18°07'N, 104°29'E, ~ 200 m, 24–29.iv.2001, Pacholátko (RABC, 1). Vientiane, Ban Van Eue, 15.xii.1965, native collector, ex malaise trap (BPBM, 3); as previous except 15.ii.1966 (BPBM, 1). Vietnam: Dong Nai, Cat Tien N.P., 11.40817, 107.38098, 134 m, 20–22.ii.2017, VN81, A.I. Cognato, T.A. Hoang, ex FIT (MSUC, 2). Quang Tri, Huong Hoa distr., Huong Hoa Nature Reserve, near Cup village, 16°56'15"N, 106°34'52"E, 400 m, 6.xi.2007, G. Csorba (HNHM, 1).

##### Diagnosis.

2.4–3.3 mm long (mean = 2.96 mm; n = 5); 2.54–2.89× as long as wide. *Stictodex dimidiatus* can be readily distinguished by the antennal club very broad, and type 2, with segment 1 straight; declivity with first and second interstriae divergent, broadest at elytral summit; declivity flat and gradually sloped; scutellum flat, flush with elytra; pronotum tight around head; protibiae inflated on posterior face. Specimens of this species demonstrate an extreme morphological continuum of variation in the elytral striae (both on disc and declivity) ranging from slightly to deeply impressed.

##### Similar species.

*Arixyleborus* spp.

##### Distribution.

Indonesia (Maluku), Laos*, East & West Malaysia, Myanmar, New Guinea, Sri Lanka, Thailand, Vietnam*.

##### Host plants.

Most host records are from the Dipterocarpaceae, but other tree families are also occasionally attacked (Browne 1961b as *Xyleborus decumans* and *X. tunggali*).

##### Remarks.

**Figure 74.**
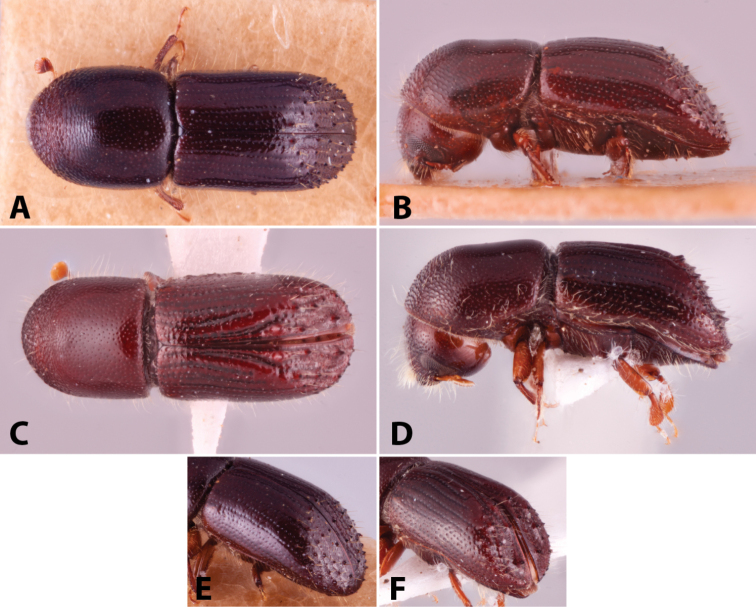
Dorsal, lateral and declivital view of *Stictodex dimidiatus*, 2.4–3.3 mm. Specimen exhibiting typical *S. dimidiatus* morphology (**A, B, E**), specimen exhibiting morphology of the synonym *X. dorsosulcatus* (**C, D, F**).

This species as currently defined is remarkably morphologically variable (Fig. 74). Further study using molecular data will be required to assess species limits.

Images of a *X. dorsosulcatus* paratype were examined. The specimen represents the most extreme declivity seen in *S. dimidiatus* with striae 1 deeply impressed (Fig. 74C, D, F). Because *X. dorsosulcatus* falls within the continuum of variation, it is here placed in synonymy with *S. dimidiatus*.

### *Streptocranus* Schedl, 1939

#### Streptocranus

Taxon classificationAnimaliaColeopteraCurculionidae

Schedl, 1939

Streptocranus Schedl, 1939b: 52.

##### Type species.

*Streptocranus mirabilis* Schedl, 1939b; monotypy.

##### Diagnosis.

The most slender and extremely elongated species (1.9–4.9 mm; 3.85–4.75× as long as wide) occurring in Southeast Asia. *Streptocranus* is distinguished by its unique subquadrate and laterally constricted pronotum (type a in lateral view; type c in dorsal view) with a flat and long pronotal disc; elytral apex divaricate and ornamented with a pair of distal processes; protibiae slender, sickle-shaped; mycangial tufts absent; scutellum flat, flush with elytra; and procoxae contiguous.

##### Similar genera.

*Debus*.

##### Distribution.

Only occurring in the Paleotropics and Oceania.

##### Gallery system.

The gallery systems of *Streptocranus* seem to be rather variable, with a few branches that may run horizontally or longitudinally, and may be irregularly widened, but without distinct brood chambers (Browne 1961b).

#### Key to *Streptocranus* species (females only)

**Table:** 

1	Elytral processes somewhat laterally compressed, much narrower in dorsal view than the space between them (Fig. 75C)	**2**
–	Elytral processes somewhat dorso-ventrally compressed, approximately as wide or wider than the space between them (Fig. 75G)	**4**
2	Elytral processes elongate, strongly tapering, strongly curved dorsad, and with an acutely pointed tip	*** bicuspis ***
–	Elytral processes short, less tapering, less strongly curved dorsad, and with a mucronate tip	**3**
3	Declivital interstriae 1 and 3 unarmed; elytral processes rounded, weakly carinate; smaller, 1.9–2.15 mm and very elongate, 4.3–4.8× as long as wide	*** fragilis ***
–	Declivital interstriae 1 and 3 with two or three granules; elytral processes subquadrate, strongly carinate; larger, 2.3 mm and less elongate, 3.8× as long as wide	***petilus* sp. nov.**
4	Large species, 4.1–4.9 mm long; dorsal margin of elytral process with a strong, inwardly-directed denticle at the level of the elytral apex	*** mirabilis ***
–	Smaller species, 2.4–3.6 mm long; dorsal margin of elytral process with a minute denticle (occasionally absent) at the level of the elytral apex	*** bicolor ***

#### Streptocranus bicolor

Taxon classificationAnimaliaColeopteraCurculionidae

Browne, 1949

Fig. 75A, B, I

Streptocranus bicolor Browne, 1949: 900.
Coptoborus bicolor (Browne): Wood and Bright 1992: 662.
Streptocranus bicolor Browne: Hulcr et al. 2007: 582.

##### Type material.

***Holotype*** (NHMUK).

##### Diagnosis.

2.4–3.6 mm long (mean = 2.69 mm; n = 5); 4.0–4.17× as long as wide. This species is distinguished by its moderate size; strongly attenuate elytra; and dorsal margin of elytral process with a minute denticle (occasionally absent) at the level of the elytral apex.

##### Similar species.

*Streptocranus fragilis*, *S. mirabilis*, *S. petilus*.

##### Distribution.

East & West Malaysia, Thailand.

##### Host plants.

Recorded from *Dryobalanops*, *Shorea* (Dipterocarpaceae), *Eugenia* (Myrtaceae), and *Palaquium* (Sapotaceae). Probably polyphagous (Beaver et al. 2014).

**Figure 75.**
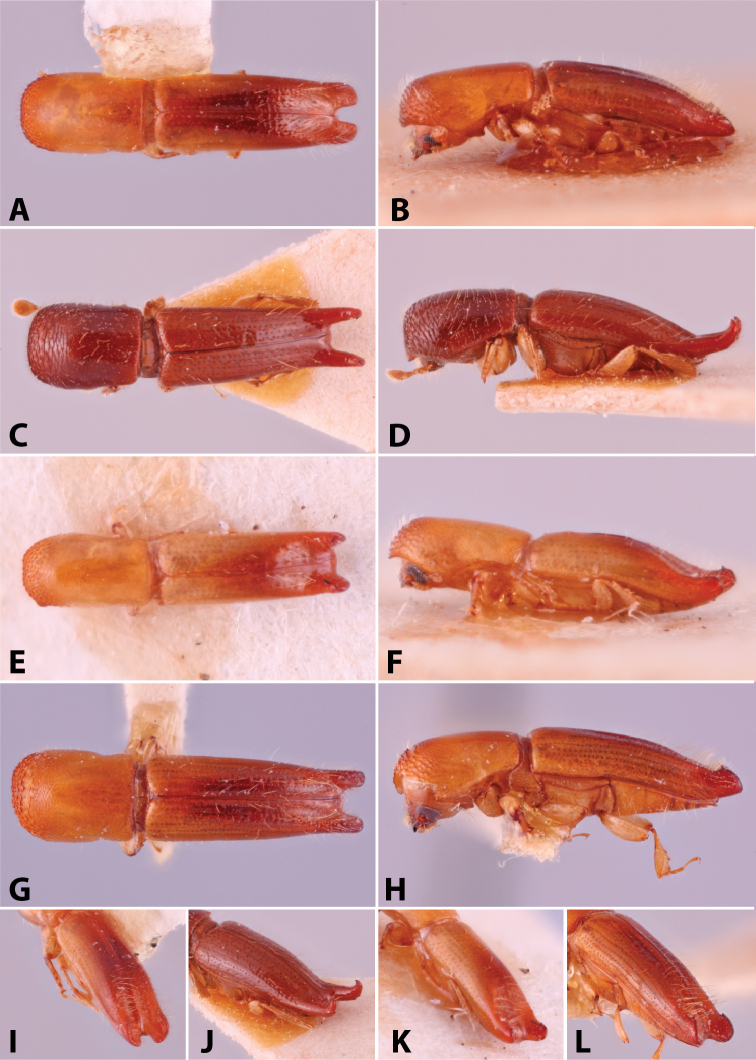
Dorsal, lateral and declivital view of *Streptocranus bicolor*, 2.4–3.6 mm (**A, B, I**), *S. bicuspis* lectotype, 2.2–3.4 mm (**C, D, J**), *S. fragilis* holotype, 1.9–2.15 mm (**E, F, K**), and *S. mirabilis* lectotype, 4.1–4.9 mm (**G, H, L**).

#### Streptocranus bicuspis

Taxon classificationAnimaliaColeopteraCurculionidae

(Eggers, 1940)

Fig. 75C, D, J

Xyleborus bicuspis Eggers, 1940: 153.
Coptoborus bicuspis (Eggers): Wood and Bright 1992: 662.
Streptocranus bicuspis (Eggers): Hulcr et al. 2007: 582.
Streptocranus recurvus Browne 1949: 898. Synonymy: Schedl 1950b: 893.

##### Type material.

***Lectotype****Xyleborus bicuspis* (NHMW), ***paralectotype*** (NMNH).

##### Diagnosis.

2.2–3.4 mm long (mean = 2.87 mm; n = 4); 3.85–4.25× as long as wide. The species is distinguished by the unique elongate elytral processes, strongly tapering, strongly curved dorsad, and with an acutely pointed tip.

##### Similar species.

None.

##### Distribution.

‘Borneo’, Indonesia (Java), West Malaysia, Thailand.

##### Host plants.

Recorded only from *Castanopsis* (Fagaceae) (Browne 1961b).

#### Streptocranus fragilis

Taxon classificationAnimaliaColeopteraCurculionidae

Browne, 1949

Fig. 75E, F, K

Streptocranus fragilis Browne, 1949: 901.
Coptoborus fragilis (Browne): Wood and Bright 1992: 663.
Streptocranus fragilis (Browne): Hulcr et al. 2007: 582.

##### Type material.

***Holotype*** (NHMUK).

##### New records.

China: Fujian, Fuzhou, 19.iv.2018, Y. Li, ex *Liquidambar formosana* (UFFE, 1). S Yunnan, Xishuangbanna, 28 km NW Jinghong, vic. An Ma Xi Zhan (NNNR), 22°12'N, 100°38'E, 700 m, forest, EKL, 5.iv.2009, L. Meng (RABC, 1).

##### Diagnosis.

1.9–2.15 mm long (mean = 2.03 mm; n = 5); 4.3–4.75× as long as wide. This species is distinguished by its small size; elytra with sides nearly parallel from base to apex; declivital interstriae 1 and 3 unarmed; and elytral distal projection short, rounded, weakly carinate.

##### Similar species.

*Streptocranus bicolor*, *S. mirabilis*, *S. petilus*.

##### Distribution.

Brunei, China* (Fujian, Yunnan), East & West Malaysia, Thailand.

##### Host plants.

Recorded from *Eugenia* (Myrtaceae), *Palaquium* (Sapotaceae) (Browne 1961b), and *Liquidambar* (Altingiaceae).

#### Streptocranus mirabilis

Taxon classificationAnimaliaColeopteraCurculionidae

Schedl, 1939

Fig. 75G, H, L

Streptocranus mirabilis Schedl, 1939b: 53.
Coptoborus mirabilis (Schedl): Wood and Bright 1992: 663.
Streptocranus mirabilis Schedl: Hulcr et al. 2007: 583.

##### Type material.

***Lectotype*** (NHMW).

##### Diagnosis.

4.1–4.9 mm long (mean = 4.45 mm; n = 3); 3.90–4.08× as long as wide. The species is the largest *Streptocranus* and is distinguished by the moderately attenuate elytra; and dorsal margin of elytral process with a strong, inwardly directed denticle at the level of the elytral apex.

##### Similar species.

*Streptocranus bicolor*, *S. fragilis*, *S. petilus*.

##### Distribution.

Indonesia (Java), West Malaysia, Thailand.

##### Host plants.

Recorded from *Mesua* (Calophyllaceae), *Quercus* (Fagaceae), and *Schoutenia* (= *Actinophora*) (Malvaceae) (Kalshoven 1959b). Probably polyphagous.

#### Streptocranus petilus sp. nov.

Taxon classificationAnimaliaColeopteraCurculionidae

http://zoobank.org/24510F58-3770-4722-BEDE-8215E9C5265A

Fig. 76

##### Type material.

***Holotype***, female, China: Yunnan, Jinghong, 24.i.2018, Shengchang Lai, ex *Hevea brasiliensis* (IZAS).

##### Diagnosis.

2.3 mm long (n = 1); 3.83× as long as wide. This species is distinguished by its small size; elytra with sides nearly parallel from base to apex; declivital interstriae 1 and 3 with two or three granules; and elytral distal projection short, subquadrate, strongly carinate.

##### Similar species.

*Streptocranus bicolor*, *S. fragilis*, *S. mirabilis*.

##### Description

**(female).** 2.3 mm long (n = 1); 3.83× as long as wide. Body light to dark brown. Legs and antennae light brown. ***Head***: Missing. ***Pronotum***: 1.48× as long as wide. In dorsal view conspicuously elongate and quadrate frontally, type c, sides tapering from summit to base; anterior margin without serrations. In lateral view conspicuously elongate and hooded frontally, type a, summit on apical 1/5. Anterior slope steep with densely spaced small asperities, becoming lower and more strongly transverse towards summit. Disc shiny, glabrous, with sparse, fine punctures. Lateral margins concave above procoxae. Base transverse, posterior angles narrowly rounded. ***Elytra***: 2.28× as long as wide, 1.5× as long as pronotum. Scutellum small, triangular, flush with elytra, flat, shiny. Elytral base transverse, edge oblique, humeral angles rounded, nearly parallel-sided along entire length, apex emarginate, each elytron with a short, subquadrate, strongly carinate distal projection that is shorter than the depth of the emargination. Disc shiny; striae irregularly seriate, not impressed, with moderately sized, shallow punctures separated by 1–3 diameters of a puncture, glabrous; interstriae flat, impunctate, glabrous. Declivity short, occupying apical 1/4, gradually rounded, shiny; striae flat, punctures as large as those of disc; interstriae laterally diverging from base to apex, interstriae 1 and 3 with two or three granules, each granule with a moderately long, erect hair. Posterolateral margin rounded. ***Legs***: procoxae contiguous; prosternal coxal piece short, inconspicuous. Protibiae slender with evenly rounded outer edge, broadest at apical 1/3; posterior face smooth; apical 1/3 of outer margin with four large socketed denticles, their length longer than basal width. Meso- and metatibiae flattened; outer margin obliquely triangular with four and five large socketed denticles, respectively.

##### Distribution.

China (Yunnan).

##### Etymology.

L. *petilus* = slender. In reference its general habitus. An adjective.

##### Host plants.

This species is only known from *Hevea brasiliensis* (Euphorbiaceae).

##### Remarks.

The head of the holotype was destroyed during fungal culturing and could not be examined.

**Figure 76.**
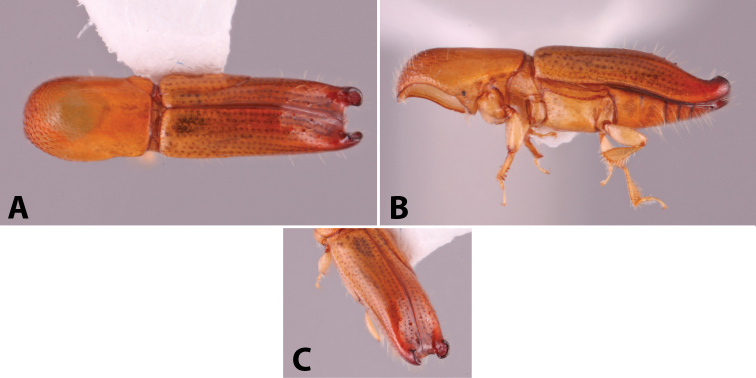
Dorsal, lateral and declivital view of *Streptocranus petilus* holotype, 2.3 mm (**A–C**).

### *Tricosa* Cognato, Smith & Beaver, 2020

#### Tricosa

Taxon classificationAnimaliaColeopteraCurculionidae

Cognato, Smith & Beaver, 2020

Tricosa Cognato, Smith & Beaver, 2020 (Cognato et al. 2020a): 547.

##### Type species.

*Xyleborus metacuneolus* (Eggers, 1940); original designation.

##### Diagnosis.

2.2–3.8 mm, 2.5–3.0× as long as wide. *Tricosa* is distinguished by the following combination of characters: antennal funicle 4-segmented; antennal club type 3 with one or two sutures visible on the posterior face; protibiae distinctly or obliquely triangular with six or fewer denticles on outer margin and posterior face flattened and unarmed; scutellum small, flush with elytra surface; mycangial tufts absent; elytra attenuate; elytral discal punctures seriate; and posterolateral costa absent (Cognato et al. 2020a).

*Tricosa* resembles *Cyclorhipidion*, *Cryptoxyleborus*, and *Fraudatrix* with which it shares either a setose and/or an attenuate appearance. *Tricosa* is most similar to *Cyclorhipidion* with which it shares a setose appearance, but is distinguished by the following diagnostic characters (*Tricosa* given first): protibiae obliquely triangular vs. semi-circular with evenly rounded outer edge; typically attenuate elytra vs. rounded, truncate or excavated; outer margin of protibiae with five or six socketed denticles vs. 6–9+; anterior margin of the pronotum typically serrate vs. unarmed (rarely serrate). *Tricosa* is distinguished from *Cryptoxyleborus* by the visible scutellum, and from *Fraudatrix* by the 4-segmented antennal funicle and antennal club type 3 with one or two sutures visible on the posterior face, and the pronotal disc being as long as or shorter than the anterior slope (Cognato et al. 2020a).

##### Similar genera.

*Cryptoxyleborus*, *Cyclorhipidion*, *Fraudatrix*.

##### Distribution.

Throughout the Oriental region and New Guinea.

##### Gallery system.

Not described.

#### Key to *Tricosa* species (females only)^3^

**Table:** 

1	Elytral discal striae and interstriae clearly uniseriate punctate	2
–	Elytral discal striae and interstriae punctures confused	3
2	Pronotum anterior margin unarmed; protibiae broad, appearing distinctly triangular	* jacula *
–	Pronotum anterior margin serrate; protibiae narrow, appearing obliquely triangular	* metacuneolus *
3	Pronotum anterior margin armed by a row of six serrations; smaller, 2.7–3.1 mm, and stouter, 2.5–2.7× as long as wide	* cattienensis *
–	Pronotum anterior margin armed by a row of eight serrations; larger, 3.2–3.4 mm, and more slender, 2.83–2.91× as long as wide	* indochinensis *

#### Tricosa cattienensis

Taxon classificationAnimaliaColeopteraCurculionidae

Cognato, Smith & Beaver, 2020

Fig. 77A, B, I

Tricosa cattienensis Cognato, Smith & Beaver, 2020 (in Cognato et al. 2020a): 548.

##### Type material.

*Holotype* (MSUC), *paratypes* (IZAS, 1; MSUC, 1; RABC, 3; UFFE, 1).

##### Diagnosis.

2.7–3.1 mm long (mean = 2.98 mm; n = 5); 2.5–2.7× as long as wide. This species is distinguished by the declivital slope gentle; declivital posterolateral margins rounded; elytral disc and declivity shiny; elytral interstriae granulate, not tuberculate; declivital striae weakly impressed; and pronotum anterior margin with a clear row of six moderate serrations.

It can be further distinguished from *T. indochinensis* by the smaller size and stouter form.

##### Similar species.

*Tricosa indochinensis*.

##### Distribution.

China (Hong Kong), Japan, Thailand, Vietnam.

##### Host plants.

Known only from *Pterocarpus* (Fabaceae), *Machilus* (Lauraceae), and a cut liana (Cognato et al. 2020a).

**Figure 77.**
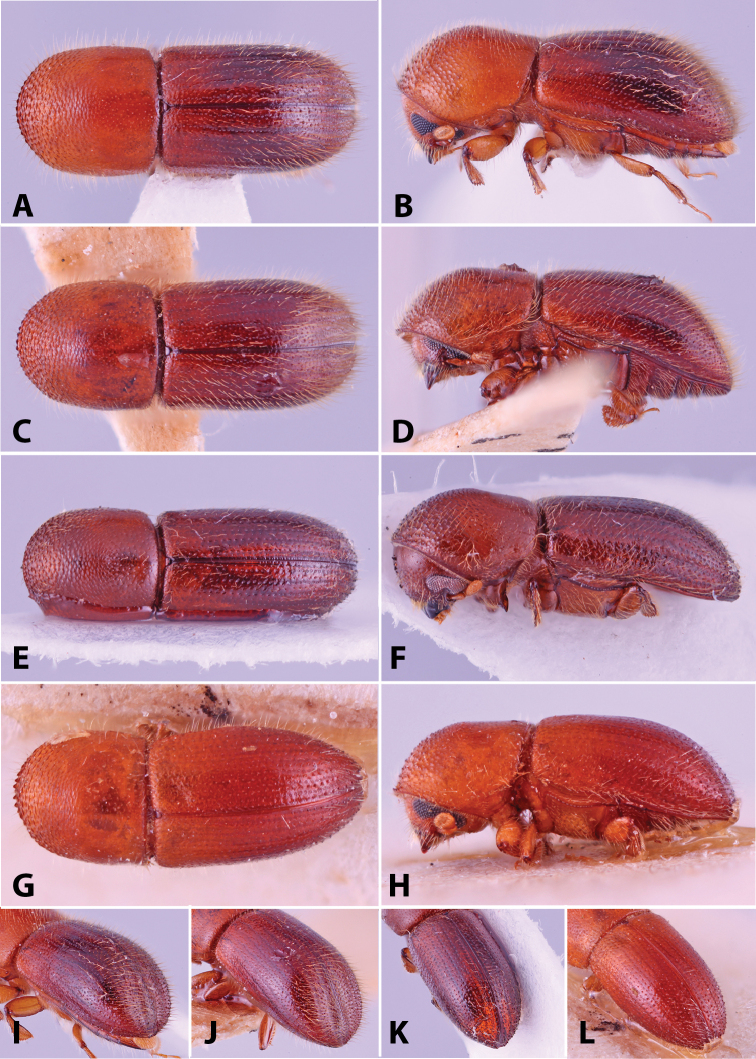
Dorsal, lateral and declivital view of *Tricosa cattienensis* holotype, 2.7–3.1 mm (**A, B, I**), *T. indochinensis* holotype, 3.2–3.4 mm (**C, D, J**), *T. jacula* holotype, 3.2 mm (**E, F, K**), and *T. metacuneolus* paratype, 2.4–2.5 mm (**G, H, L**).

#### Tricosa indochinensis

Taxon classificationAnimaliaColeopteraCurculionidae

Cognato, Smith & Beaver, 2020

Fig. 77C, D, J

Tricosa indochinensis Cognato, Smith & Beaver, 2020 (in Cognato et al. 2020a): 549.

##### Type material.

*Holotype* (NMNH), *paratypes* (IZAS, 1; NMNH, 4; RABC, 1).

##### Diagnosis.

3.2–3.4 mm long (mean = 3.32 mm; n = 5); 2.83–2.91× as long as wide. This species is distinguished by the discal interstrial punctures confused; protibiae distinctly triangular; and pronotum anterior margin with a clear row of eight moderate serrations.

This species is very similar to *T. cattienensis* and is distinguished by the larger size and narrower form (Cognato et al. 2020a).

##### Similar species.

*Heteroborips indicus*, *Tricosa cattienensis*.

##### Distribution.

China (Yunnan), India (West Bengal), Thailand.

##### Host plants.

Known only from *Pterocarpus* (Fabaceae) (Cognato et al. 2020a).

#### Tricosa jacula

Taxon classificationAnimaliaColeopteraCurculionidae

Cognato, Smith & Beaver, 2020

Fig. 77E, F, K

Tricosa jacula Cognato, Smith & Beaver, 2020 (in Cognato et al. 2020a): 549.

##### Type material.

*Holotype* (IZAS).

##### Diagnosis.

3.2 mm long (n = 1); 2.91× as long as wide. This species is distinguished by the elytral discal striae and interstriae clearly uniseriate punctate; pronotum anterior margin unarmed; and protibiae distinctly triangular (Cognato et al. 2020a).

##### Similar species.

*Fraudatrix melas*, *Tricosa metacuneolus*.

##### Distribution.

China (Guizhou).

##### Host plants.

This species has been reported from *Populus* (Salicaceae) (Cognato et al. 2020a).

#### Tricosa metacuneolus

Taxon classificationAnimaliaColeopteraCurculionidae

(Eggers, 1940)

Fig. 77G, H, L

Xyleborus metacuneolus Eggers, 1940: 150.
Tricosa metacuneola [*sic*] (Eggers): Cognato et al. 2020a: 550.
Xyleborus kaimochii Nobuchi, 1981a: 143. Synonymy: Smith et al. 2018b: 397.

##### Type material.

*Paratype Xyleborus metacuneolus* (NMNH).

##### Diagnosis.

2.4–2.5 mm long (mean = 2.46 mm; n = 5); 2.67–2.78× as long as wide. This species is distinguished by the elytra gently attenuate on apical 30%; declivital interstriae uniseriate granulate, granules numerous, spaced by a distance of less than three granule widths; and declivital striae and interstriae densely setose, strial setae 1/2 as long as those of interstriae.

##### Similar species.

*Coptodryas mus*, *Fraudatrix cuneiformis*.

##### Distribution.

Brunei, Indonesia (Java, Sulawesi), Japan, East & West Malaysia, New Guinea, Philippines, Sri Lanka, Taiwan, Thailand.

##### Host plants.

Probably polyphagous. Recorded from *Buchanania*, *Mangifera* (Anacardiaceae), *Castanopsis* (Fagaceae), *Swietenia* (Meliaceae), and *Gymnacranthera* (Myristicaceae) (Nobuchi 1981a; Beaver and Liu 2010; Cognato et al. 2020a).

### *Truncaudum* Hulcr & Cognato, 2010

#### Truncaudum

Taxon classificationAnimaliaColeopteraCurculionidae

Hulcr & Cognato, 2010

Truncaudum Hulcr & Cognato, 2010a: 24.

##### Type species.

*Xyleborus impexus* Schedl, 1942b; original designation.

##### Diagnosis.

Small to moderately sized, somewhat elongate (1.9–2.9 mm, 2.44–2.9 × as long as wide) and densely pubescent. *Truncaudum* is distinguished by the declivity obliquely or abruptly truncate; pronotum elongate without distinct serrations on anterior margin; protibiae semi-circular with evenly rounded outer margin; scutellum visible, procoxae contiguous, mycangial tufts absent.

The two species in Southeast Asia are strikingly similar to several small *Cyclorhipidion* species and is distinguished by the obliquely truncate (type 2) antennal club while those of *Cyclorhipidion* are flat and types 3, 4, 5.

##### Similar genera.

*Amasa*, *Cyclorhipidion*, *Pseudowebbia*.

##### Distribution.

Found throughout the Paleotropics and Australasia with one species occurring in Africa.

##### Gallery system.

The gallery system has a few branches, usually in the transverse plane, and at least one brood chamber in the longitudinal plane (Browne 1961b).

#### Key to *Truncaudum* species (females only)

**Table:** 

1	Declivity obliquely truncate, margins rounded; declivital interstriae 1 flat	*** agnatum ***
–	Declivity abruptly truncate, surrounded by a circumdeclivital costa margined with a row of variably tubercles; declivital interstriae 1 tumescent	***bullatum* sp. nov.**

#### Truncaudum agnatum

Taxon classificationAnimaliaColeopteraCurculionidae

(Eggers, 1923)

Fig. 78A, B, E

Xyleborus agnatus Eggers, 1923: 197.
Truncaudum agnatum (Eggers): Hulcr and Cognato 2010a: 25.
Xyleborus polyodon Eggers, 1923: 196. Synonymy: Hulcr 2010: 114.
Xyleborus gratiosus Schedl, 1942a: 199. Synonymy: Hulcr and Cognato 2010a: 25.
Xyleborus nutans Schedl, 1942a: 199. Synonymy: Bright and Skidmore 1997: 4, 151.
Xyleborus delicatus Schedl, 1955a: 300. Synonymy: Hulcr and Cognato 2010a: 25.
Xyleborus subagnatus Wood, 1992: 85. Synonymy: Hulcr and Cognato 2010a: 25.

##### Type material.

***Paralectotype****Xyleborus nutans* (NHMUK). ***Lectotype****Xyleborus polyodon* (NMNH).

##### Diagnosis.

2.1–2.9 mm long (mean = 2.4 mm; n = 5); 2.44–2.9× as long as wide and densely pubescent. This species is distinguished by the declivity obliquely truncate, margins rounded; declivital interstriae 1 flat; and large size.

This species is strikingly similar to many small *Cyclorhipidion* species and is distinguished by the characters given for the genus.

##### Similar species.

Small *Cyclorhipidion* spp.

##### Distribution.

Australia, ‘Borneo’, Federated States of Micronesia, Indonesia (Java, Maluku, Sulawesi, Sumatra), New Caledonia, New Guinea, Palau, Philippines, Solomon Islands, Thailand.

##### Host plants.

Polyphagous (Browne 1961b; Ohno 1990).

##### Remarks.

Both molecular and morphological data suggest that this is a complex of species. Further study of the complex is needed.

**Figure 78.**
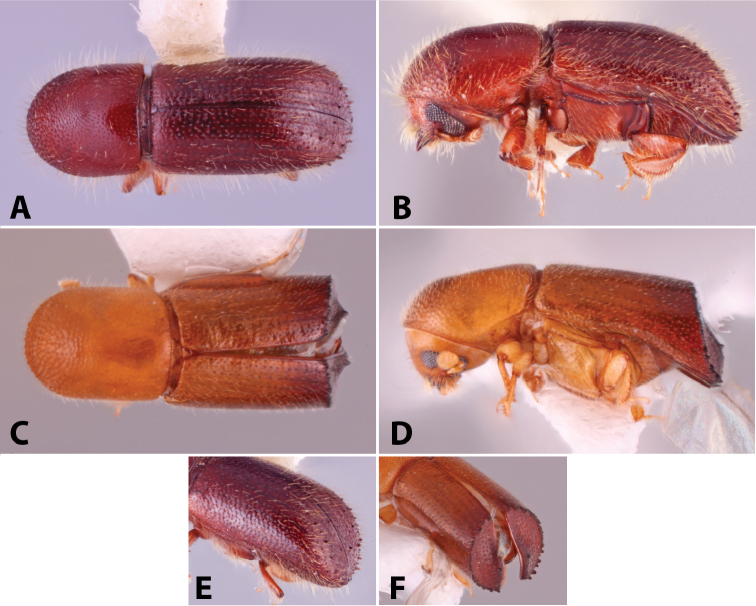
Dorsal, lateral and declivital view of *Truncaudum agnatum*, 2.1–2.9 mm (**A, B, E**), and *T. bullatum* holotype, 1.9 mm (**C, D, F**).

#### Truncaudum bullatum sp. nov.

Taxon classificationAnimaliaColeopteraCurculionidae

http://zoobank.org/10B70B6C-4F14-4995-B96C-CB06C9B20F2D

Fig. 78C, D, F

##### Type material.

***Holotype***, female, China: Fujian, Fuzhou, Qishan, Y. Li, 18.iv.2018, ex unknown twig (IZAS).

##### Diagnosis.

1.9 mm long (n = 1); 2.71× as long as wide. This species is distinguished by the declivity abruptly truncate, surrounded by a complete costa and margined with a row of variably tubercles; declivital interstriae 1 tumescent; and small size.

This species is strikingly similar to many small *Cyclorhipidion* species and is distinguished by the characters given for the genus.

##### Similar species.

*Amasa* spp., *Arixyleborus* spp., *Cyclorhipidion* spp., *Pseudowebbia* spp., *Webbia* spp.

##### Description

**(female).** 1.9 mm long (n = 1); 2.71× as long as wide. Head, pronotum, antennae and legs light brown. Elytra bicolored: elytral disc light brown, becoming darker apically, declivital face maroon. ***Head***: epistoma entire, transverse, with a row of hair-like setae. Frons weakly convex to upper level of eyes; surface shagreened, impunctate, alutaceous; granulate just above epistoma. Eyes deeply emarginate just above antennal insertion, upper part smaller than lower part. Submentum narrow, triangular, deeply impressed. Antennal scape regularly thick, as long as club. Pedicel as wide as scape, approximately as long as funicle. Funicle 4-segmented, segment 1 shorter than pedicel. Club approximately circular, obliquely truncate, type 2; segment 1 corneous, transverse on anterior face, occupying basal 2/5, nearly covering posterior face; segment 2 narrow, corneous; segment 1 present on posterior face. ***Pronotum***: 1.2× as long as wide. In dorsal view long and rounded frontally, type 7, sides parallel in basal 1/2, rounded anteriorly; anterior margin without serrations. In lateral view elongate with disc much longer than anterior slope, type 7, disc flat, summit at apical 1/4. Anterior slope with densely spaced, fine asperities, becoming lower and more strongly transverse towards summit, bearing long, fine, erect hair-like setae. Disc shiny, alutaceous with very dense, fine punctures, glabrous. Lateral margins obliquely costate. Base transverse, posterior angles narrowly rounded. ***Elytra***: 1.35× as long as wide, 1.1× as long as pronotum. Scutellum small, linguiform, shiny, flush with elytra, flat. Elytral base transverse, edge oblique, humeral angles rounded, parallel-sided in basal 3/4, then sharply angulate to apex. Disc shiny, densely setose; striae not impressed, punctures fine, shallow, separated by three diameters of a puncture; interstriae flat, finely punctate, punctures as large as those of striae, strongly confused, setose, each bearing a short, semi-erect hair-like seta. Declivity truncate, strongly shagreened, dull, almost glabrous; interstriae impunctate, interstriae 1 laterally broadened from declivital summit to apical 1/3 then narrowed to apex, tumescent, one denticle on apical 1/3; tumescent area sparsely setose, setae short, stout, erect; strial punctures very large, shallow, much larger than on disc, punctures subcontiguous with those of adjacent rows. Posterolateral margin forming a circumdeclivital carina, carina coarsely tuberculate, tubercles increasing in size from base to apex. ***Legs***: procoxae contiguous, prosternal coxal piece inconspicuous. Protibiae slender with evenly rounded outer edge, broadest at apical 1/3; posterior face smooth; apical 1/3 of outer margin with four large socketed denticles, their length longer than basal width. Meso- and metatibiae flattened; outer margin evenly rounded with seven moderate socketed denticles, their length equal to basal width.

##### Etymology.

L. *bullatus* = inflated. Named in reference to the tumescent declivity. An adjective.

##### Distribution.

China (Fujian).

##### Host plants.

Unknown.

### *Webbia* Hopkins, 1915

#### Webbia

Taxon classificationAnimaliaColeopteraCurculionidae

Hopkins, 1915

Webbia Hopkins, 1915b: 222.
Xelyborus Schedl, 1939a: 349. Synonymy: Browne 1963: 57.
Prowebbia Browne, 1962: 208. Synonymy: Browne 1972: 25.

##### Type species.

*Webbia dipterocarpi* Hopkins, 1915b; original designation.

##### Diagnosis.

1.9–3.4 mm long, 2.6–3.75× as long as wide. *Webbia* is distinguished by the scutellum not apparent; dense tuft of setae present along elytral base associated with an elytral mycangium; antennal funicle 2-segmented; protibiae slender, outer margin armed with more than nine denticles, posterior face inflated and unarmed; pronotum conspicuously elongated, rectangular in dorsal aspect, disc flat, anterolateral corners inflated (type a in dorsal view); elytra with few setae, abruptly truncated and often elaborately ornamented with large projections.

##### Similar genera.

*Amasa*, *Arixyleborus*, *Cyclorhipidion*, *Pseudowebbia*.

##### Distribution.

Throughout the Paleotropics.

##### Gallery system.

The unbranched radial entrance tunnel leads to a single large brood chamber in the longitudinal plane (Browne 1961b).

##### Remarks.

The majority of species are strongly associated with Dipterocarpaceae, but single species are specialized on Fagaceae and Sapotaceae (Browne 1961b).

#### Key to *Webbia* species (females only)

**Table:** 

1	Circumdeclivital margin carinate and unarmed by denticles or spines; declivity densely covered with thick semi-recumbent golden setae; declivity unarmed by processes	*** dasyura ***
–	Circumdeclivital margin costate and denticulate or spinose; declivity glabrous or with few fine hair-like setae; declivity armed by processes	**2**
2	Circumdeclivital margin denticulate; declivital summit with striae and interstriae flush; entire elytral disc smooth, shiny (Fig. 80E)	**3**
–	Circumdeclivital margin spinose; declivital summit with striae strongly impressed and interstriae costate; posterior 25–40% of elytral disc coarsely sculptured, shagreened, dull, anterior portions smooth and shiny (Fig. 80C)	**7**
3	Declivity with short elytral processes, as long as basal width, their apices acute (Fig. 79C)	**4**
–	Declivity with long elytral processes, spinose, longer than 1.5× their basal width, their apices bifurcate (Fig. 79A)	**5**
4	Elytral process arising from apical margin, rounded apically with a short, medially directed spine (Fig. 79C); elytral apex entire; declivity smooth and strongly shiny, striae 1 and 3 very weakly impressed, interstriae without granules; strial and interstrial punctures very fine, of the same size; smaller, 1.9–2.2 mm	*** cornuta ***
–	Elytral process arising from declivital face, short and acute (Fig. 81C); declivity appearing rugose, striae 1–3 distinctly impressed, interstriae granulate; interstrial punctures coarse, shallow, strial punctures smaller; elytral apices weakly but distinctly divaricate; larger, 2.2–3.4 mm	*** turbinata ***
5	Apical processes of elytra not strongly widened from base to apex, their upper and lower edges subparallel	*** pabo ***
–	Apical processes of elytra triangular, strongly widened from base to apex	**6**
6	Base of triangular spine elongate, occupying approximately 1/3 of declivital length; acute spine at elytral apex arising from the sutural interstriae; discal interstriae 1 denticulate, never prolonged into a short spine over the declivity	*** biformis ***
–	Base of triangular spine narrow, occupying approximately 1/4 of declivital length; acute spine at elytral apex arising from the second interstriae, distinctly separated from the suture; discal interstriae 1 prolonged into a short spine over the declivity	***diversecauda***
7	Margin of declivity with six or seven spines on each side	**8**
–	Margin of declivity with at least nine spines on each side	**9**
8	Margin of declivity with six spines on each side, lacking teeth on interstriae 2, 4, and 5, or these teeth much smaller than others; declivital face with a single vermiculate ridge on each side and a row of tubercles lateral to it	*** duodecimspinata ***
–	Margin of declivity with seven teeth on each side, lacking teeth on interstriae 2 and 4; declivital face with two strong vermiculate ridges on each side and without additional tubercles	*** quatuordecimspinata ***
9	Margin of declivity with 13–15 teeth on each side; declivital face with the vermiculate ridge on interstriae 1 strongly raised in middle of declivity, and with three or four rows of tubercles lateral to it; elytral disc shiny to upper margin of declivity, apart from grooves between marginal teeth	*** trigintispinata ***
–	Margin of declivity with nine or ten teeth on each side; declivital face with a weak ridge on interstriae 1, and two rows of tubercles lateral to it; elytral disc matte on posterior 1/4	*** dipterocarpi ***

#### Webbia biformis

Taxon classificationAnimaliaColeopteraCurculionidae

Browne, 1958

Fig. 79A, B, I

Webbia biformis Browne, 1958: 496.

##### Type material.

***Holotype*** (NHMUK), ***paratypes*** (NHMUK, 3).

##### Diagnosis.

2.4–3.0 mm long (mean = 2.67 mm; n = 3); 3.43–3.75× as long as wide. This species is distinguished by the circumdeclivital margin denticulate; declivital face bearing a large triangular spine that is as much broader at apex than base; base of spine elongate, occupying approximately 1/3 of declivital length; acute spine at elytral apex arising from the sutural interstriae; and discal interstriae 1 denticulate, never prolonged into a short spine over the declivity.

##### Similar species.

*Webbia diversicauda*, *W. pabo*.

##### Distribution.

East & West Malaysia, Thailand.

##### Host plants.

Associated with Dipterocarpaceae (*Dipterocarpus*, *Hopea*, *Shorea*) (Browne 1961b; Beaver and Browne 1975, 1979).

##### Remarks.

Brood size can be as high as 120 in a gallery (Beaver and Browne 1979).

**Figure 79.**
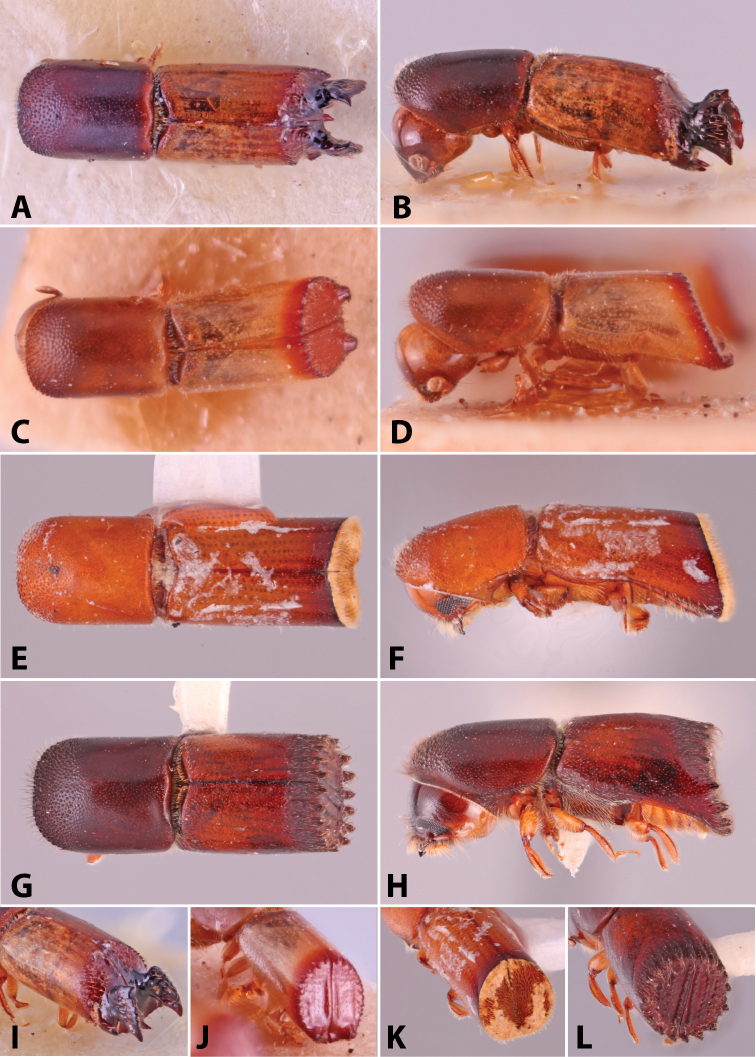
Dorsal, lateral and declivital view of *Webbia biformis* holotype, 2.4–3.0 mm (**A, B, I**), *W. cornuta*, 1.9–2.2 mm (**C, D, J**), *W. dasyura* holotype, 2.8–2.9 mm (**E, F, K**), and *W. dipterocarpi*, 3.0–3.2 mm (**G, H, L**).

#### Webbia cornuta

Taxon classificationAnimaliaColeopteraCurculionidae

Schedl, 1942

Fig. 79C, D, J

Webbia cornutus [*sic*] Schedl, 1942a: 183.

##### Type material.

***Lectotype*** (NHMW). Not examined.

##### Diagnosis.

1.9–2.2 mm long (mean = 2.02 mm; n = 5); 3.14–4.0× as long as wide. This species is distinguished by the circumdeclivital margin denticulate; declivity rather smooth and strongly shiny; declivital striae 1 and 3 very weakly impressed, declivital interstriae without granules; declivital strial and interstrial punctures very fine, of the same size, bearing short fine hair-like setae; short acute elytral process arising from declivital margin; and elytral apex entire.

##### Similar species.

*Webbia turbinata*.

##### Distribution.

‘Borneo’, East & West Malaysia, Thailand.

##### Host plants.

Associated with Dipterocarpaceae (*Dipterocarpus*, *Hopea*, *Shorea*) (Browne 1961b; Beaver and Browne 1975, 1979).

##### Remarks.

As in *W. biformis*, brood size can be large (up to 87) (Beaver and Browne 1979). The majority of the records from Thailand listed under this species by Beaver et al. (2014) should be transferred to *W. turbinata*. The records from the southern provinces of Chumphon and Nakhon Sri Thammarat are correct.

#### Webbia dasyura

Taxon classificationAnimaliaColeopteraCurculionidae

Browne, 1981

Fig. 79E, F, K

Webbia dasyurus [*sic*] Browne, 1981a: 133.

##### Type material.

***Holotype*** (NHMUK).

##### New records.

Laos: Kham Mouan, Ban Khun Ngeun, 18°07'N, 104°29'E, ~ 200 m, 24–29.iv.2001, Pacholátko (RABC, 1).

##### Diagnosis.

2.8–2.9 mm long (mean = 2.83 mm; n = 3); 2.8–2.95× as long as wide. This species is distinguished by the circumdeclivital margin carinate smooth, unarmed by granules or tubercles; declivital face densely covered with thick semi-recumbent golden setae; and declivity unarmed by any spines or processes.

##### Similar species.

None.

##### Distribution.

Laos*, East Malaysia, Philippines.

##### Host plants.

Recorded from *Dipterocarpus*, *Dryobalanops*, *Shorea* (Dipterocarpaceae) (Ohno 1990).

#### Webbia dipterocarpi

Taxon classificationAnimaliaColeopteraCurculionidae

Hopkins, 1915

Fig. 79G, H, L

Webbia dipterocarpi Hopkins, 1915b: 223.
Webbia octodecimspinatus Sampson, 1921: 32. Synonymy: Wood 1983: 650.

##### Type material.

***Holotype*** (NMNH).

##### New records.

Thailand: Trang, Khaophappha Khaochang, 200–400 m, 13.i.1964, G.A. Samuelson (BPBM, 1).

##### Diagnosis.

3.0–3.2 mm long (mean = 3.12 mm; n = 5); 2.73–2.91× as long as wide. This species is distinguished by the margin of declivity with nine or ten teeth on each side; declivital face with a weak ridge on interstriae 1, and two rows of tubercles lateral to it; and elytral disc matte on posterior 1/4.

##### Similar species.

*Webbia duodecimspinata*, *W. quatuordecimspinata*, *W. trigintispinata*.

##### Distribution.

East & West Malaysia, Philippines, Thailand*, Vietnam.

##### Host plants.

Associated with Dipterocarpaceae (*Dipterocarpus*, *Dryobalanops*, *Hopea*, *Shorea*, *Vatica*) (Browne 1961b; Beaver and Browne 1979; Ohno 1990). Recorded once each from *Bridelia* and *Macaranga* (Euphorbiaceae) (Browne 1961b).

##### Remarks.

Browne (1961b) gives some details of the biology and development period. The record from Vietnam was recorded as *W. duodevigintispinatus* Sampson by Browne (1968b). Browne later corrected the identification to *W. octodecimspinatus* in a letter to RAB (F.G. Browne, pers. comm., 18 August 1978).

#### Webbia diversicauda

Taxon classificationAnimaliaColeopteraCurculionidae

Browne, 1972

Fig. 80A, B, I

Webbia diversicauda Browne, 1972: 26.

##### Type material.

***Holotype*** (NHMUK).

##### Diagnosis.

2.75–2.9 mm long (mean = 2.83 mm; n = 5); 3.5–3.73× as long as wide. This species is distinguished by the circumdeclivital margin denticulate; declivital face bearing a large triangular spine that is much broader at apex than base; base of spine narrow, occupying approximately 1/4 of declivital length; acute spine at elytral apex arising from the second interstriae, distinctly separated from the suture; discal interstriae 1 prolonged into a short spine over the declivity.

##### Similar species.

*Webbia biformis*, *W. pabo*.

##### Distribution.

West Malaysia, Thailand.

##### Host plants.

Unknown.

**Figure 80.**
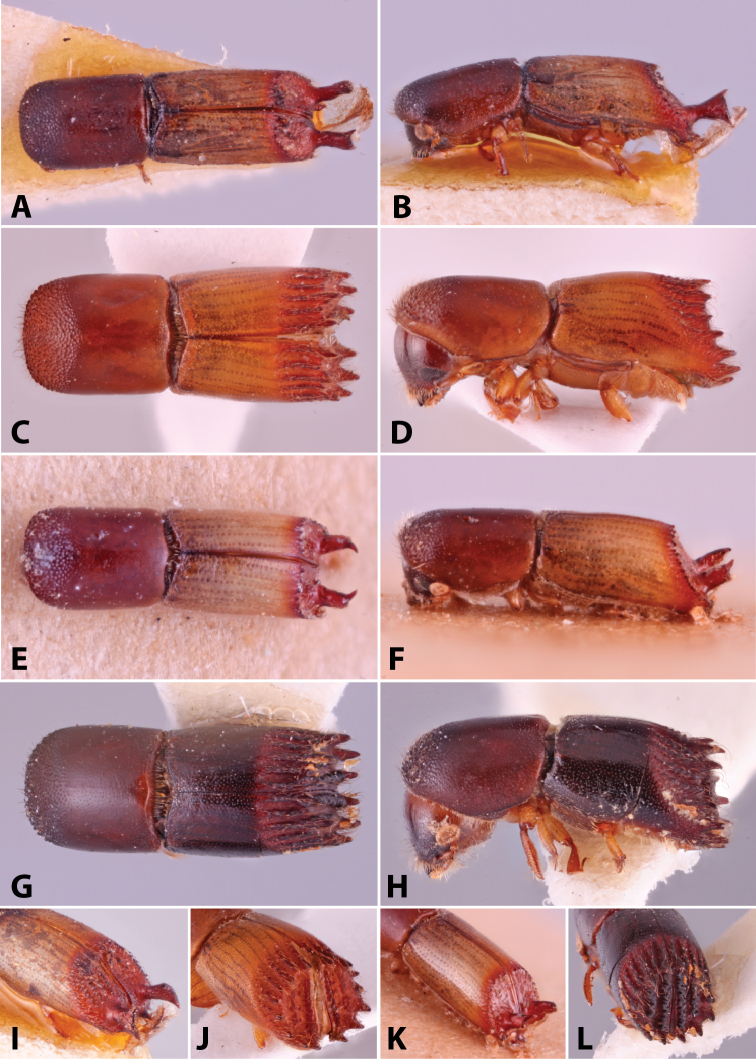
Dorsal, lateral and declivital view of *Webbia diversicauda* holotype, 2.75–2.9 mm (**A, B, I**), *W. duodecimspinata*, 3.1–3.5 mm (**C, D, J**), *W. pabo*, 2.3–2.5 mm (**E, F, K**), and *W. quatuordecimspinata*, 2.8–3.0 mm (**G, H, L**).

#### Webbia duodecimspinata

Taxon classificationAnimaliaColeopteraCurculionidae

Schedl, 1942

Fig. 80C, D, J

Webbia 12-spinatus [*sic*] Schedl, 1942a: 182.

##### Type material.

***Lectotype*** (NHMW). Not examined.

##### Diagnosis.

3.1–3.5 mm long (mean = 3.27 mm; n = 5); 2.6–2.92× as long as wide. This species is distinguished by the entire circumdeclivital margin armed with six spines on each side, lacking teeth on interstriae 2, 4, and 5, or these teeth much smaller than others; declivital face with a single vermiculate ridge on each side and a row of tubercles lateral to it.

##### Similar species.

*Webbia dipterocarpi*, *W. quatuordecimspinata*, *W. trigintispinata*.

##### Distribution.

West Malaysia, Thailand.

##### Host plants.

Associated with Dipterocarpaceae (*Dipterocarpus*, *Hopea*, *Shorea*) (Beaver and Browne 1979; Beaver et al. 2014).

##### Remarks.

A brood of 107 offspring is recorded by Beaver and Browne (1979).

#### Webbia pabo

Taxon classificationAnimaliaColeopteraCurculionidae

Sampson, 1922

Fig. 80E, F, K

Webbia pabo Sampson, 1922: 150.

##### Type material.

***Holotype*** (NHMUK).

##### Diagnosis.

2.3–2.5 mm long (mean = 2.5 mm; n = 4); 3.29–3.57× as long as wide. This species is distinguished by the circumdeclivital margin denticulate; declivital face bearing a large spine that is as broad at apex as base; and an acute spine at elytral apex arising from the sutural interstriae.

##### Similar species.

*Webbia biformis*, *W. diversicauda*.

##### Distribution.

‘Borneo’, China (Xizang, Yunnan), India (Madhya Pradesh, Uttarakhand), Indonesia (Maluku), East Malaysia, Thailand.

##### Host plants.

Associated with Dipterocarpaceae (*Anisoptera*, *Dipterocarpus*, *Dryobalanops*, *Shorea*) (Beaver and Browne 1975; Ohno 1990).

#### Webbia quatuordecimspinata

Taxon classificationAnimaliaColeopteraCurculionidae

Sampson, 1921

Fig. 80G, H, L

Webbia 14-spinatus [*sic*] Sampson, 1921: 34.
Webbia quatuordecimspinatus Schedl, 1942a: 182. Synonymy: Wood 1989: 176.
Webbia quatuordecimcostatus Schedl, 1952b: 61. Synonymy: Browne 1961a: 310.
Webbia sampsoni Nunberg, 1956: 209. Unnecessary replacement name for W. quatuordecimspinatus Schedl.

##### Type material.

***Holotype*** (NHMUK).

##### Diagnosis.

2.8–3.0 mm long (mean = 2.84 mm; n = 5); 2.8–3.0× as long as wide. This species is distinguished by the margin of declivity with seven teeth on each side, lacking teeth on interstriae 2 and 4; declivital face with two strong vermiculate ridges on each side and without additional tubercles.

##### Similar species.

*Webbia dipterocarpi*, *W. duodecimspinata*, *W. trigintispinata*.

##### Distribution.

‘Borneo’, Brunei, East & West Malaysia, Philippines, Thailand.

##### Host plants.

Associated with Dipterocarpaceae (*Dipterocarpus*, *Dryobalanops*, *Hopea*, *Shorea*), but also recorded from unidentified species of Burseraceae and Euphorbiaceae (Browne 1961b).

#### Webbia trigintispinata

Taxon classificationAnimaliaColeopteraCurculionidae

Sampson, 1922

Fig. 81A, B, E

Webbia 14-spinatus [*sic*] Sampson, 1922: 149.
Webbia 26-spinatus [*sic*] Sampson, 1922: 149. Synonymy: Browne, 1963: 57.
Webbia trigintispinatus [*sic*] Sampson. Browne, 1968b: 133.
Webbia vigintisexspinata Sampson. Corrected name.
Webbia mucronatus Eggers, 1927b: 107. syn. nov.

##### Type material.

***Holotype****Webbia trigintispinata* (NHMUK). ***Holotype****Webbia mucronatus* (NMNH).

##### New records.

Ceylon [Sri Lanka]: Galle district, Kanneliya, 250 m, 23.v.1975, S.L. Wood, collected from log (NMNH, 1). Kalutara district, Morapitiya, 250 m, 27.v.1975, S.L. Wood, misc. hosts (NMNH, 1). Vietnam: Cochinchine, F. de Thuc-Trong, 1934, Caresche (MNHN, 2).

##### Diagnosis.

3.0 mm long (mean = 3.0 mm; n = 5); 3.0× as long as wide. This species is distinguished by the margin of declivity with 13–15 teeth on each side; declivital face with the vermiculate ridge on interstriae 1 strongly raised in middle of declivity, and with three or four rows of tubercles lateral to it; and elytral disc shiny to upper margin of declivity, apart from grooves between marginal teeth.

##### Similar species.

*Webbia dipterocarpi*, *W. duodecimspinata*, *W. quatuordecimspinata*.

##### Distribution.

Cambodia, India (Andaman Is, Assam), Indonesia (Sumatra), East & West Malaysia, Philippines, Sri Lanka*, Thailand, Vietnam.

##### Host plants.

Associated with Dipterocarpaceae (*Dipterocarpus*, *Dryobalanops*, *Hopea*, *Shorea*) (Beeson 1961; Ohno 1990).

##### Remarks.

The species name *26-spinatus* is an incorrect original spelling which is here corrected to *vigintisexspinata*. The corrected name is a subjective synonym of *Webbia trigintispinata*. Images of the *W. mucronatus* holotype were examined by all authors and found to be conspecific with the *W. trigintispinata* holotype and non-type specimens. It is here placed in synonymy.

**Figure 81.**
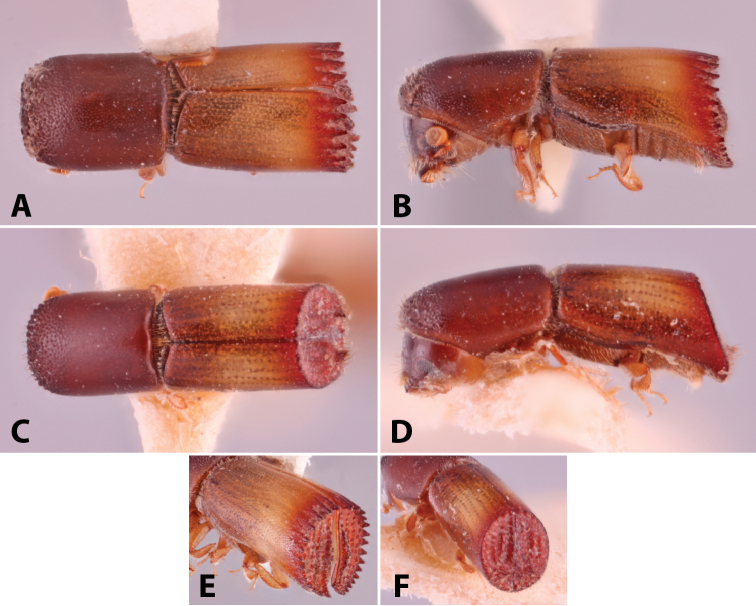
Dorsal, lateral and declivital view of *Webbia trigintispinata*, 3.0 mm (**A, B, E**), and *W. turbinata*, 2.2–3.4 mm (**C, D, F**).

#### Webbia turbinata

Taxon classificationAnimaliaColeopteraCurculionidae

Maiti & Saha, 1986

Fig. 81C, D, F

Webbia turbinatus [*sic*] Maiti & Saha, 1986: 104.

##### Type material.

***Holotype*** (FRI). Not examined.

##### New records.

Thailand: Chaiyaphum, Phu Khieo, 17.vii.2005, Hulcr et al., ex *Shorea* branch (MSUC, 1).

##### Diagnosis.

2.2–3.4 mm long; 3.14–3.2× as long as wide. This species is distinguished by the circumdeclivital margin denticulate; declivity appearing rugose, striae 1–3 distinctly impressed, interstriae granulate; declivital strial punctures coarse, shallow, hair-like setae arising from punctures rather coarse; short acute elytral process arising from declivital face, not declivital margin; and elytral apices weakly but distinctly divaricate.

##### Similar species.

*Webbia cornuta*.

##### Distribution.

India (Andaman Is), Thailand*.

##### Host plants.

Recorded from *Dipterocarpus*, *Shorea* (Dipterocarpaceae) and *Sapium* (Euphorbiaceae) (Maiti and Saha 1986).

##### Remarks.

The records of *Webbia cornuta* from Thailand (Chiang Mai and Phrae) in Beaver and Browne (1975), and those from Chaiyaphum, Chiang Mai, Phetchabun and Trat in Beaver et al. (2014) should be transferred to this species.

### *Xyleborinus* Reitter, 1913

#### Xyleborinus

Taxon classificationAnimaliaColeopteraCurculionidae

Reitter, 1913

Xyleborinus Reitter, 1913: 83.

##### Type species.

*Bostrichus saxesenii* Ratzeburg, 1837; subsequent designation: Swaine, 1918: 50.

##### Diagnosis.

Typically small (1.6–3.1 mm) and elongate (2.3–3.4× as long as wide). *Xyleborinus* is most readily distinguished by the unique scutellum and elytral mycangia: scutellum minute, conical, disconnected from elytra and mycangium which opens adjacent to scutellum. In addition, the antennal club is obliquely truncate with segment 1 corneous and dominant on both sides of the club (type 1), protibiae obliquely triangular, and procoxae contiguous.

Southeast Asian *Heteroborips* species have elytral mycangium opening adjacent to the scutellum but the scutellum is never minute, conical and disconnected from the elytra.

##### Similar genera.

*Cryptoxyleborus*, *Heteroborips*, *Microperus*, *Xyleborus*.

##### Distribution.

Widespread throughout temperate and tropical regions of the world.

##### Gallery system.

In many species, a short unbranched entrance tunnel leads to a brood chamber in the longitudinal plane; in others, such as *X. artestriatus*, the tunnel branches and there are several small brood chambers (Browne 1961b; Schedl 1963a). The brood chamber is enlarged by the larvae as they develop.

#### Key to *Xyleborinus* species (females only)

Note that granules or tubercles on the declivital summit are not considered to be on the declivital face.

**Table:** 

1	Declivital interstriae 1 unarmed on declivital face (Fig. 86A)	**2**
–	Declivital interstriae 1 armed on declivital face (Fig. 83G)	**13**
2	Only declivital interstriae 1 unarmed on declivital face	*** subspinosus ***
–	Declivital interstriae 1 and 2 unarmed on declivital face	**3**
3	Elytra parallel in basal 1/2, tapering posteriorly to attenuate apex (Fig. 86A)	**4**
–	Elytra parallel for at least basal 40%, broadly rounded to apex (Fig. 83A)	**5**
4	Larger, 2.3–2.75 mm and less elongate, 2.83–2.89× as long as wide; elytra with small denticles on interstriae 1–4 not extending anteriorly beyond the declivital summit; pronotum less elongate, 1.14× as long as wide	*** spinipennis ***
–	Smaller, 2.1–2.2 mm and more elongate, 3.14–3.23× as long as wide; elytra with small denticles on interstriae 1–4 extending anteriorly onto the disc to at least the midpoint; pronotum more elongate, 1.22× as long as wide	***cuneatus* sp. nov.**
5	Declivital face with impunctate striae	**6**
–	Declivital face with punctate striae	**8**
6	Declivity strongly sulcate between interstriae 3; interstriae 3 strongly elevated and costate; larger, 2.5–3.1 mm	*** schaufussi ***
–	Declivity flattened or weakly sulcate between interstriae 3; interstriae 3 weakly elevated; smaller, 1.6–2.0 mm	**7**
7	Declivital interstriae 3 denticles increasing in size apically, third denticle very large; denticles along interstriae 5 distinctly smaller than those on interstriae 3	***disgregus* sp. nov.**
–	Declivital interstriae 3 denticles subequal or uniform in size; denticles along interstriae 5 subequal to those on interstriae 3	***jianghuasuni* sp. nov.**
8	Very small, 1.6–1.8 mm; declivital face flattened and shagreened; discal interstriae 1 and 2 granulate, granules extending from declivital summit to midpoint of disc (Fig. 86D, J)	***subgranulatus* (in part)**
–	Larger, 2.1–3.0 mm; declivital face weakly to moderately sulcate, opalescent or shiny; discal interstriae granulate only at declivital summit (Fig. 82D, J)	**9**
9	Declivital face moderately sulcate, interstriae 3 strongly elevated and costate, bearing 4–6 pairs of long, narrow, acute spines	*** octiesdentatus ***
–	Declivital face weakly sulcate, interstriae 3 weakly elevated, bearing denticles or short spines	**10**
10	Declivital striae 1 and 2 nearly convergent, their punctures subcontiguous interstriae 2 very narrow; larger, 3.0 mm	***echinopterus* sp. nov.**
–	Declivital striae 1 and 2 distinctly separated, interstriae 2 wide, at least the width of two strial punctures; smaller, 2.1–2.7 mm	**11**
11	Declivital interstriae 3 with broad tubercles, their bases wider than their length and apices obtusely rounded (Fig. 82C)	*** artestriatus ***
–	Declivital interstriae 3 with narrow denticles or spines, their bases less than or equal to their length and apices pointed (Fig. 85G)	**12**
12	Declivital interstriae 3 feebly elevated, bearing three small pairs of sharply pointed denticles increasing in size from base to apex; declivity opalescent; smaller, 2.1–2.25 mm	*** sculptilis ***
–	Declivital interstriae weakly elevated, bearing three pairs of short, narrow, sharply pointed spines, spines equal in size; declivity strongly shiny and smooth; larger, 2.6–2.7 mm	*** speciosus ***
13	Declivital interstriae 1 and 2 granulate, interstriae 3 spinose	***ephialtodes* sp. nov.**
–	Declivital interstriae 1 armed, 2 unarmed, 3 armed	**14**
14	Elytra strongly attenuate from basal 1/2; apex acute	*** andrewesi ***
–	Elytra parallel for at least basal 2/3; apex narrowly or broadly rounded	**15**
15	Denticles of interstriae 3 larger than those of interstriae 1	**16**
–	Denticles of interstriae 3 and interstriae 1 approximately equal	**18**
16	Smaller, shorter than 2.0 mm; denticles of interstriae 1 and 3 with bluntly rounded apices; elytral apex with three (usually) pairs of large flattened tubercles; declivity strongly shiny	*** exiguus ***
–	Larger, longer than 2.6 mm; denticles of interstriae 1 and 3 spine-like with acute apices; elytral apex with 1–2 small acute tubercles; declivity shagreened, dull	**17**
17	Denticles of declivital interstriae 3 and 5 spine-like with apices slightly recurved in lateral view (Fig. 82F)	*** attenuatus ***
–	Denticles of declivital interstriae 3 and 5 spine-like with apices erect, not recurved (Fig. 86H)	***thaiphami* sp. nov.**
18	Declivital interstriae 2 unarmed along its entire length (Fig. 84G, L)	*** perpusillus ***
–	Declivital interstriae 2 armed at declivital summit and/ or disc by granules (Fig. 87A, C)	**19**
19	Declivital interstriae 1 and 3 strongly convex; bases of denticles tumescent; striae 1 nearly convergent with striae 2 on declivital face, interstriae 2 not apparent	***tritus* sp. nov.**
–	Declivital interstriae 1 and 3 weakly convex; bases of denticles never tumescent; striae 1 clearly separated from striae 2, interstriae 2 distinct	**20**
20	Larger, 2.3−2.5 mm; discal interstriae 1 and 2 unarmed (Fig. 85A, I)	*** saxesenii ***
–	Smaller, 1.6−1.8 mm; discal interstriae 1 and 2 granulate, granules extending from declivital summit up to midpoint of disc (Fig. 86D, J)	**21**
21	Discal interstriae 1 and 2 granules extending from declivital summit to apical quarter of disc (Fig. 84B, I)	***huifenyinae* sp. nov.**
–	Discal interstriae 1 and 2 granules extending from declivital summit to midpoint of disc (Fig. 86D, J)	***subgranulatus* (in part)**

#### Xyleborinus andrewesi

Taxon classificationAnimaliaColeopteraCurculionidae

(Blandford, 1896)

Fig. 82A, B, I

Xyleborus andrewesi Blandford, 1896b: 227.
Xyleborinus andrewesi (Blandford): Wood 1989: 176.
Xyleborus persphenos Schedl, 1970a: 219. Synonymy: Schedl 1975e: 34.
Xyleborus insolitus Bright, 1972: 77. Synonymy: Bright 1985: 173.
Cryptoxyleborus gracilior Browne, 1984: 101. Synonymy: Beaver 1995a: 198.

##### Type material.

***Holotype****Xyleborus andrewesi* (NHMUK). ***Holotype****Cryptoxyleborus gracilior* (NHMUK).

##### New records.

China: Fujian, Fuan, Shuyang, 29.ix.2018, A. Ernstsons, ex EtOH trap (MSUC, 3). Hong Kong, Tai Po Kau, vi.2017, J. Skelton (MSUC, 1). Vietnam: Dong Nai, Cat Tien N.P., 11.44221, 107.43114, 379 m, 20–22.ii.2017, VN78, A.I. Cognato, T.A. Hoang, ex FIT (MSUC, 26). Lao Cai, Hoang Lien N.P., 22.35, 103.77, 1500–2000 m, 19–20.v.2019, VN184, S.M. Smith, A.I. Cognato, ex FIT (MSUC, 3). Thua Thien-Hue, Bach Ma N.P., 16.25038, 107.87352, 29 m, 15.ii.2017, VN52, A.I. Cognato, T.A. Hoang, ex in flight (MSUC, 1).

##### Diagnosis.

1.9–2.0 mm long (mean = 1.92 mm; n = 5); 3.17–3.33× as long as wide. This species is distinguished by the elytra strongly attenuate with apex acute.

##### Similar species.

*Cryptoxyleborus* spp., *Xyleborinus cuneatus*.

##### Distribution.

Recorded in the study region from Bangladesh, China (Fujian*, Hong Kong*, Yunnan), India (Andaman Is, Jharkhand, Karnataka, Kerala, Madhya Pradesh, Odisha, Tamil Nadu, Uttarakhand, West Bengal), Nepal, Taiwan, Thailand, Vietnam. It also occurs in Sri Lanka and through Malaysia and Indonesia, to the Philippines and New Guinea. Presumably imported to and established in East Africa (Tanzania, Zambia) and the Seychelles. Introduced and established in the USA (Okins and Thomas 2010; Gomez et al. 2018a).

##### Host plants.

Polyphagous (Browne 1961b; Schedl 1963a).

##### Remarks.

Kalshoven (1959b) gives some details of brood sizes at different stages of development of the brood chamber. The species attacks plantation trees, but the attacks are secondary on stressed or dying host trees, and not primary on healthy trees (Maiti and Saha 2004).

**Figure 82.**
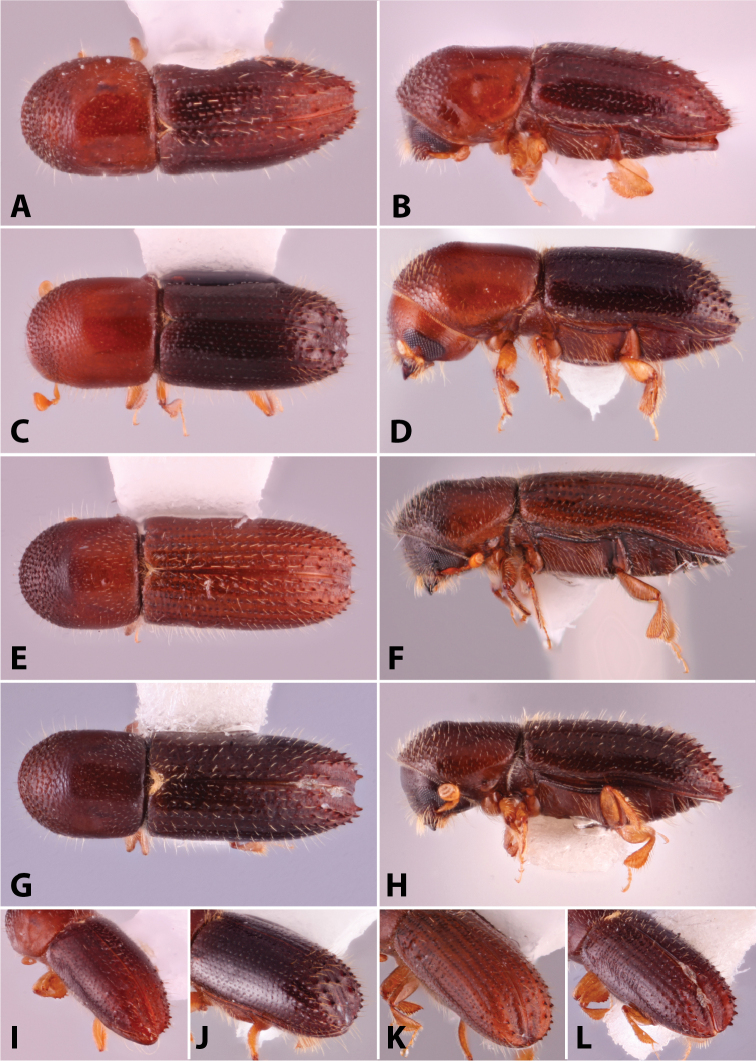
Dorsal, lateral and declivital view of *Xyleborinus andrewesi*, 1.9–2.0 mm (**A, B, I**), *X. artestriatus*, 2.3–2.8 mm (**C, D, J**), *X. attenuatus*, 2.6–2.9 mm (**E, F, K**), and *X. cuneatus* holotype, 2.1–2.2 mm (**G, H, L**).

#### Xyleborinus artestriatus

Taxon classificationAnimaliaColeopteraCurculionidae

(Eichhoff, 1878)

Fig. 82C, D, J

Xyleborus artestriatus Eichhoff, 1878b: 507.
Xyleborinus artestriatus (Eichhoff): Saha and Maiti 1984: 4.
Xyleborus laticollis Blandford, 1896b: 226. Synonymy: Schedl 1958c: 152.
Xyelborus angustior [*sic*] Eggers, 1925: 158. syn. nov.
Xyleborus rugipennis Schedl, 1953b: 303. Synonymy: Wood 1989: 176.
Xyleborus undatus Schedl, 1974: 264. syn. nov.
Xyleborus beaveri Browne, 1979 (in Beaver and Browne 1979): 603. Synonymy: Beaver and Liu 2010: 30.

##### Type material.

***Paratype****Xyelborus angustior* (NMNH). ***Holotype****Xyleborus beaveri* (NHMUK), ***paratypes*** (NHMUK, RABC). ***Holotype****Xyleborus laticollis* (NHMUK). ***Paratype****Xyleborus undatus* (NHMW).

##### New records.

Cambodia: Siem Reap, Preaj Khan temple, 26.vii.2006, O. Yothin, ex Malaise trap (IRSNB, 1). China: Hong Kong, Tai Po Kau, vi.2017, J. Skelton (MSUC, 2). S Yunnan, Xishuangbanna, 20 km NW Jinghong, vic. Man Dian (NNNR), 22°07.80'N, 100°40.05'E, 740 m, rubber plantation, 18.vi.2008, A. Weigel (RABC, 1); as previous except: 730 m, 15.vi.2008, EKL (NKME, 2); as previous except: 23 km NW Jinghong, vic. Na Ban (NNNR), 22°09.49'N, 100°39.92'E, 730 m, forest, 15.vi.2008, A. Weigel (RABC, 1); as previous except: rubber plantation (NKME, 2); as previous except: 25 km NW Jinghong, vic. Zhang Zhi Chang (NNNR), 22°11.06'N, 100°39.05'E, 780 m, rubber plant., 5.vi.2008, A. Weigel (RABC, 1); as previous except Hekou city, 3.vi.2014, Wang (RJRC, 2); as previous except Jinping city (RJRC, 1). Laos: 10 km N Luang-Prabang, Mekhong river, 240 km N Vientiane, hills c. 250 m, poor settlem[ent], prim[ary] veget[ation] lux, iii.1993, Insomsay Somsy (MFNB, 17); as previous except: iv.1993 (MFNB, 1). Wapikhamthong, Khong Sedone, Wapi, native collector, 15.iv.1967 (BPBM, 1); as previous except 30.viii.1967 (BPBM, 1). Taiwan: Nantou, Sun Moon Lake, 1.v.2014, C.-S. Lin (MSUC, 1). Vietnam: Lao Cai, Nam Tha, 22.01218, 104.37685, 9.v.2015, Pham Thu, ex funnel trap (RJRC, 1). Ninh Binh, Cuc Phuong N.P., Mac Lake, 20°15'29.0"N, 105°42'27.5"E, 155 m, 4–7.v.2009, J.B. Heppner, blacklight trap (FSCA, 1). Tuyen Quang, Doi Can Tuyen Quang, 21.72740, 105.22742, 15.iv.2015, R.J. Rabaglia, ex funnel trap (RJRC, 1). Vinh Phuc, Me Linh Biodiversity station, Dai Lai Lake, 27–29.ix.2013, 100 m, J.B. Heppner (FSCA, 1).

##### Diagnosis.

2.3–2.8 mm long (mean = 2.48 mm; n = 5); 2.3–3.13× as long as wide. This species is distinguished by the posterior margin of elytra broadly rounded; declivital face with interstriae 1 and 2 unarmed by tubercles; declivital face weakly sulcate; large body size; lateral declivital margins elevated along interstriae 3, bearing two or three pairs of large, broad, obtusely pointed tubercles of different sizes on basal 1/2 of declivity; sulcate area of declivity smooth, shiny or opalescent; and stout body.

##### Similar species.

*Xyleborinus echinopterus*, *X. ephialtodes*, *X. octiesdentatus*, *X. schaufussi*, *X. speciosus*, *X. spinipennis*.

##### Distribution.

Recorded in the study region from Bangladesh, Cambodia*, China (Chongqing, Fujian, Guangdong, Guangxi, Hainan, Hong Kong*, Shanghai, Yunnan, unknown province), India (Madhya Pradesh, Sikkim, Uttarakhand, West Bengal), Laos, Myanmar, Taiwan, Thailand, Vietnam. It also occurs in Sri Lanka, and through the Indomalayan region to New Guinea and Australia and has been introduced to the United States (Cognato et al. 2013; Gomez et al. 2018a).

##### Host plants.

Polyphagous (Wood and Bright 1992).

##### Remarks.

In this species the gallery system is branched with several small brood chambers (Browne 1961b).

The holotype of *Xyelborus angustior* is in poor condition; only the mesonotum and abdomen without elytra remain glued to the card. Eggers’ description clearly indicates that the specimen strongly resembled *X. artestriatus* before it became damaged. The species description and paratype specimen of *Xyleborus undatus* was also examined and found to be conspecific with *X. artestriatus*. Both species are here placed in synonymy with *X. artestriatus*.

#### Xyleborinus attenuatus

Taxon classificationAnimaliaColeopteraCurculionidae

(Blandford, 1894)

Fig. 82E, F, K

Xyleborus attenuatus Blandford, 1894b: 114.
Xyleborinus attenuatus (Blandford): Beaver and Liu 2010: 30.
Xyleborus alni Niisima, 1909: 160. Synonymy: Knížek 2011: 246.
Xyleborus canus Niisima, 1909: 161. Synonymy: Smith et al. 2018b: 397.

##### Type material.

***Holotype****Xyleborus attenuatus* (NHMUK). ***Syntypes*** of *Xyleborus canus* should be housed in NIAES but have not been located (Smith et al. 2018b).

##### New records.

China: Chongqing, Wu Xi, 4.viii.2015, Wang, J-G., Lv-Jia, Tian-Shang, ex *Pinus armandii* (RABC, 2). Shaanxi, Feng Xian, 20–22.v.2016, Nie, Yang (MSUC, 1). Vietnam: Cao Bang, 22°36.402'N, 105°52.397'E, 1601 m, 13.iv.2014, VN17, Cognato, Smith, Pham, ex standing stump (MSUC, 1); as previous except: 22°36.804'N, 105°51.982'E, 1831 m, 17.iv.2014, VN44, Cognato, Smith, Pham, ex fallen tree, 10 cm branch (MSUC, 1).

##### Diagnosis.

2.6–2.9 mm long (mean = 2.78 mm; n = 5); 2.9–3.25× as long as wide. This species is distinguished by the declivital face with interstriae 2 armed by granules at declivital summit, unarmed on declivital face; denticles of declivital interstriae 3 larger than those of interstriae 1; denticles pointed, spine-like, slightly incurved; denticles on interstriae 5 large, sharply pointed, spine-like, curved slightly downwards; discal interstriae 1 and 2 unarmed; declivital interstriae 2 flattened; and large size.

This species is nearly identical to *X. thaiphami* and is distinguished by the declivital interstriae 3 denticles that are incurved rather than acutely pointed and interstriae 5 denticles always down-curved.

##### Similar species.

*Xyleborinus saxesenii*, *X. subgranulatus*, *X. subspinosus*, *X. thaiphami*.

##### Distribution.

China* (Chongqing, Shaanxi), Japan, Korea, Russia (Far East), Taiwan. Introduced and established in central and northern Europe and North America (Knížek 1988; Hoebeke and Rabaglia 2007; Gomez et al. 2018a).

##### Host plants.

Previously recorded from trees in the families Betulaceae, Fagaceae and Rosaceae (Beaver and Liu 2010). Recorded here from *Pinus* (Pinaceae).

#### Xyleborinus cuneatus sp. nov.

Taxon classificationAnimaliaColeopteraCurculionidae

http://zoobank.org/48594710-73BC-4F5D-9FB7-21E84DD8977B

Fig. 82G, H, L

##### Type material.

***Holotype***, female, Thailand: Chiang Mai, Doi Pui, 1400 m, 10–31.i.2005, W. Puranasakul, ex EtOH trap (NHMUK). ***Paratypes***, female, as holotype except: 10–31.v.2005 (RABC, 1); as holotype except: 8–12.xi.2004, flight intercept trap (QSBG, 1).

##### Diagnosis.

2.1–2.2 mm long (mean = 2.13 mm; n = 3); 3.14–3.23× as long as wide. This species is distinguished by the elytra parallel-sided in basal 1/2, tapering posteriorly; declivital face with interstriae 1 and 2 unarmed by tubercles; declivital face moderately sulcate; small body size; lateral declivital margins moderately elevated, costate, bearing 4–6 pairs of large sharply pointed backwardly hooked denticles (often asymmetric); elytra with small denticles on interstriae 1–4 extending anteriorly onto the disc to at least the midpoint; strial punctures large; declivital surface shagreened, dull; and elongate body form.

This species is very similar to *X. spinipennis* and can be recognized by the smaller size and more elongate form, elytra with small denticles on interstriae 1–4 extending anteriorly onto the disc to at least the midpoint, and pronotum more elongate, 1.22× as long as wide.

##### Similar species.

*Xyleborinus andrewesi*, *X. disgregus*, *X. jianghuasuni*, *X. sculptilis*, *X. speciosus*, *X. spinipennis*.

##### Description

**(female).** 2.1–2.2 mm long (mean = 2.13 mm; n = 3); 3.14–3.23× as long as wide. Body dark red-brown. Legs and antennae light brown. ***Head***: epistoma entire, transverse, with a row of hair-like setae. Frons weakly convex to upper level of eyes, alutaceous, subshiny, sparsely punctate; punctures large, shallow, setose; punctures bearing a long, erect hair-like seta. Eyes deeply emarginate just above antennal insertion, upper part smaller than lower part. Submentum narrowly triangular, deeply impressed. Antennal scape regularly thick, as long as club. Pedicel as wide as scape, as long as funicle. Funicle 4-segmented, segment 1 shorter than pedicel. Club longer than wide, obliquely truncate, type 1; segment 1 corneous, encircling anterior face; segment 2 narrow, concave, corneous on anterior face only; sutures absent on posterior face. ***Pronotum***: 1.17× as long as wide. In dorsal view very elongate, rounded frontally, type 9, sides parallel on basal 3/4; anterior margin without serrations. In lateral view elongate with disc much longer than anterior slope, type 8, disc flat, summit prominent at apical 1/3. Anterior slope with densely spaced narrow asperities, becoming lower and more strongly transverse towards summit, bearing long, fine, semi-recumbent, hair-like setae. Disc subshiny, alutaceous, finely punctate, finely setose, setae short, erect, hair-like, some longer hair-like setae at margins. Lateral margins obliquely costate. Base transverse, posterior angles acutely rounded. ***Elytra***: 2.02× as long as wide, 1.73× as long as pronotum. Scutellum minute, conical, disconnected from elytra, surrounded by dense mycangial tuft of setae. Elytral base transverse, medially emarginate near scutellum and mycangial tuft, edge oblique, humeral angles angulate, parallel-sided in basal 1/2, then tapering to apex. Disc occupying basal 2/3, smooth on basal 1/2, shagreened and dull on apical 1/2; striae not impressed, glabrous, with moderate punctures separated by one diameter of a puncture; interstriae flat, sparsely finely uniseriate punctate, punctures 1/3 those of striae, each bearing erect, hair-like setae, setae approximately as long as width of interstriae 2; apical 1/2 of interstriae armed by granules medially and increasing in size apically, becoming large denticles at declivital base. Declivital face steeply rounded, impressed between interstriae 1 and 3, appearing bisulcate, strongly shagreened; three striae present, striae parallel, strial punctures slightly larger than on disc, glabrous; interstriae impunctate; interstriae 1 with two denticles present at base, remainder unarmed; interstriae 2 narrow, flat, impressed, unarmed along its length; interstriae 3 elevated on basal 3/4 with a row of with a row of five or six regularly spaced, strong spines. Interstrial denticles and spines setose, setae erect, hair-like, uniseriate, as long as the width between suture and interstriae 3. Posterolateral margin costate, denticulate to interstriae 7. ***Legs***: procoxae contiguous; prosternal coxal piece flat, inconspicuous. Protibiae obliquely triangular, broadest at apical 1/3; posterior face smooth; apical 1/3 of outer margin with six moderate socketed denticles, their length approximately as long as basal width. Meso- and metatibiae flattened; outer margin evenly rounded with eight and five moderate socketed denticles, respectively.

##### Etymology.

L. *cuneatus* = wedge-shaped. In reference to the shape of the beetle. An adjective.

##### Distribution.

Thailand.

##### Host plants.

Unknown.

#### Xyleborinus disgregus sp. nov.

Taxon classificationAnimaliaColeopteraCurculionidae

http://zoobank.org/52FE12D4-C53E-4DE2-A606-3A4A92B19B1F

Fig. 83A, B, I

##### Type material.

***Holotype***: female, Vietnam: Cao Bang, 22°34.118'N, 105°52.537'E, 1048 m, 12–17.iv.2014, VN9, Cognato, Smith, Pham, ex FIT (MSUC). ***Paratypes***, female, India: Arunachal Pradesh, Etalin vicinity, 28°36'56"N, 95°53'21"E, 700 m, 12–25.v.2012, L. Dembický (ZFMK, 1); Vietnam: Cao Bang, 22°33.9981'N, 105°52.591'E, 1051 m, 2–17.iv.2014, VN9, Cognato, Smith, Pham, ex FIT (MSUC, 1).

##### Diagnosis.

1.8–2.0 mm long (mean = 1.87 mm; n = 3); 3.0–3.33× as long as wide. This species is distinguished by the posterior margin of elytra broadly rounded; declivital face with interstriae 1 and 2 unarmed by tubercles; declivital face feebly sulcate; small body size; lateral declivital margins weakly elevated, bearing three pairs of sharply pointed denticles, denticles increasing in size from base to apex; denticles along interstriae 5 distinctly smaller than those on interstriae 3; sulcate area impunctate, surface strongly shagreened, dull; and elongate body form.

##### Similar species.

*Xyleborinus cuneatus*, *X. jianghuasuni*, *X. sculptilis*.

##### Description

**(female).** 1.8–2.0 mm long (mean = 1.87 mm; n = 3); 3.0–3.33× as long as wide. Body, legs and antennae light brown, elytra becoming darker apically, declivity red-brown. ***Head***: epistoma entire, transverse, with a row of hair-like setae. Frons flattened to upper level of eyes, alutaceous, subshiny, finely, sparsely punctate, setose; punctures bearing a long, erect hair-like seta. Eyes deeply emarginate just above antennal insertion, upper part smaller than lower part. Submentum narrowly triangular, deeply impressed. Antennal scape short and thick, as long as club. Pedicel as wide as scape, as long as funicle. Funicle 4-segmented, segment 1 shorter than pedicel. Club longer than wide, obliquely truncate, type 1; segment 1 corneous, encircling anterior face; segment 2 narrow, concave, corneous on anterior face only; sutures absent on posterior face. ***Pronotum*.** 1.06× as long as wide. In dorsal view very elongate, rounded frontally, type 9, sides parallel on basal 3/4; anterior margin without serrations. In lateral view elongate with disc much longer than anterior slope, type 8, disc flat, summit moderately prominent, at apical 1/3. Anterior slope with densely spaced narrow asperities, becoming lower and more strongly transverse towards summit, bearing long, fine, semi-recumbent hair-like setae. Disc subshiny, alutaceous, finely punctate, finely setose, setae short, erect, hair-like, some longer hair-like setae at margins. Lateral margins obliquely costate. Base transverse, posterior angles acutely rounded. ***Elytra***: 2.0× as long as wide, 1.88× as long as pronotum. Scutellum minute, conical, disconnected from elytra, surrounded by dense mycangial tuft of setae. Elytral base transverse, medially emarginate near scutellum and mycangial tuft, edge oblique, humeral angles angulate, parallel-sided in basal 3/4, then broadly rounded to apex. Disc occupying basal 2/3, smooth, shiny, glabrous, unarmed; striae not impressed, with moderate punctures separated by two diameters of a puncture; interstriae flat, sparsely finely uniseriate punctate, punctures 1/3 those of striae. Declivital face strongly shagreened, dull, steeply rounded, feebly sulcate between interstriae 3; three striae present, striae and interstriae impunctate; interstriae 1 and 2 flat, unarmed on face, two small denticles present at base; interstriae 3 weakly elevated with a row of three pairs of sharply pointed denticles, denticles increasing in size from base to apex. Interstrial denticles and spines setose, setae erect, hair-like, uniseriate and as long as the width between suture and interstriae 3. Posterolateral margin without a costa, denticulate from interstriae 5–6. ***Legs***: procoxae contiguous; prosternal coxal piece flat, inconspicuous. Protibiae distinctly triangular, broadest at apical 1/3; posterior face smooth; apical 1/3 of outer margin with six moderate socketed denticles, their length approximately as long as basal width. Meso- and metatibiae flattened; outer margin evenly rounded with 5–7 and seven or eight moderate socketed denticles, respectively.

##### Etymology.

L. *disgregus* = unlike, different. In reference to the interesting pattern of granules on the declivity. An adjective.

##### Distribution.

India (Arunachal Pradesh), Vietnam.

##### Host plants.

Unknown.

**Figure 83.**
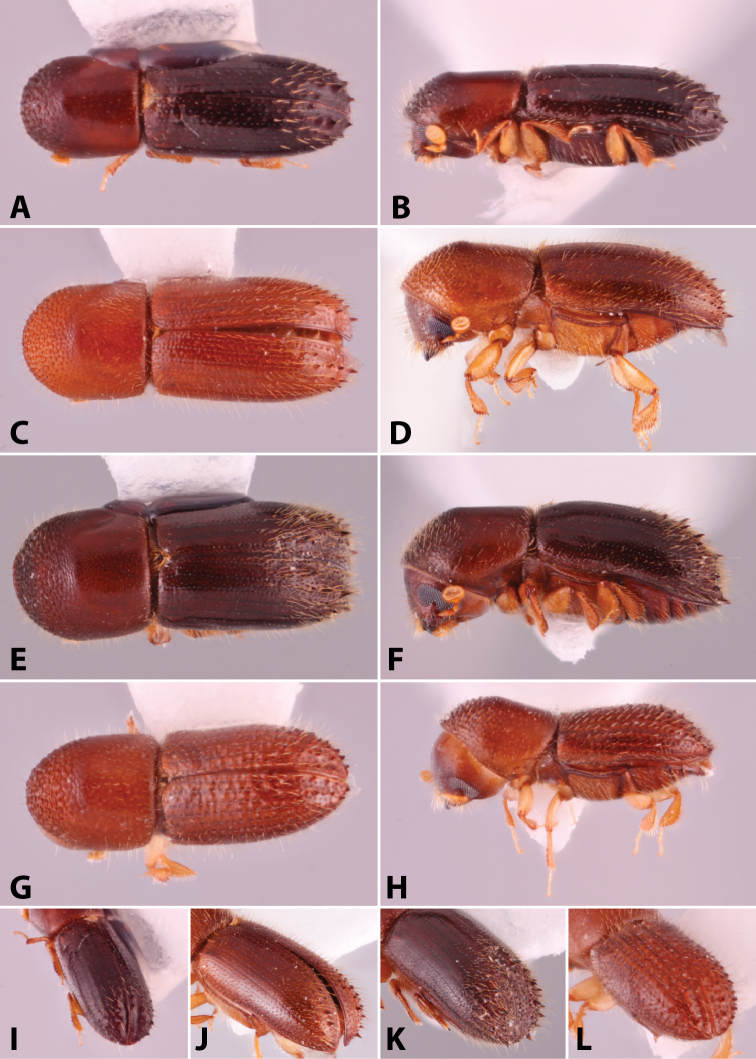
Dorsal, lateral and declivital view of *Xyleborinus disgregus* holotype, 1.8–2.0 mm (**A, B, I**), *X. echinopterus* holotype, 3.0 mm (**C, D, J**), *X. ephialtodes* holotype, 2.6 mm (**E, F, K**), and *X. exiguus*, 1.8–2.0 mm (**G, H, L**).

#### Xyleborinus echinopterus sp. nov.

Taxon classificationAnimaliaColeopteraCurculionidae

http://zoobank.org/941C16C1-E4AA-4A23-AB2E-F389651A493E

Fig. 83C, D, J

##### Type material.

***Holotype***, female, China: Hong Kong, Shing Mun, 24–28.v.2017, ex intercept trap (IZAS).

##### Diagnosis.

3.0 mm long (n = 1); 2.73× as long as wide. This species is distinguished by the posterior margin of elytra broadly rounded; declivital face with interstriae 1 and 2 unarmed by tubercles; declivital face weakly sulcate; large body size; lateral declivital margins elevated, bearing 2–4 pairs of small, narrow, sharply pointed spines of equal size (often asymmetric) on basal 2/3; sulcate area of declivity smooth, shiny; and stout body.

##### Similar species.

*Xyleborinus artestriatus*, *X. ephialtodes*, *X. octiesdentatus*, *X. schaufussi*, *X. speciosus*, *X. spinipennis*.

##### Description

**(female).** 3.0 mm long (n = 1); 2.73× as long as wide. Body, legs, and antennae uniformly light brown. ***Head***: epistoma entire, transverse, with a row of hair-like setae. Frons weakly convex to upper level of eyes, alutaceous, subshiny, finely, sparsely punctate, setose; punctures bearing a long, erect hair-like seta. Eyes shallowly emarginate just above antennal insertion, upper part smaller than lower part. Submentum large, distinctly triangular, deeply impressed. Antennal scape regularly thick, as long as club. Pedicel as wide as scape, as long as funicle. Funicle 4-segmented, segment 1 shorter than pedicel. Club longer than wide, obliquely truncate, type 1; segment 1 corneous, encircling anterior face; segment 2 narrow, concave, corneous on anterior face only; sutures absent on posterior face. ***Pronotum***: 1.02× as long as wide. In dorsal view basic and parallel-sided, sides parallel in basal 2/3, rounded anteriorly; anterior margin without serrations. In lateral view elongate, disc longer than anterior slope, type 7, summit low, on apical 2/5. Anterior slope with densely spaced narrow asperities, becoming lower and more strongly transverse towards summit, bearing long, fine, semi-recumbent, hair-like setae. Disc subshiny, alutaceous, finely punctate, finely setose, setae short, erect, hair-like, some longer hair-like setae at margins. Lateral margins obliquely costate. Base transverse, posterior angles acutely rounded. ***Elytra***: 1.69× as long as wide, 1.67× as long as pronotum. Scutellum minute, conical, disconnected from elytra, surrounded by dense mycangial tuft of setae. Elytral base transverse, medially emarginate near scutellum and mycangial tuft, edge oblique, humeral angles angulate, parallel-sided in basal 3/4, then broadly rounded to apex. Disc occupying basal 3/5, smooth, shiny, glabrous, unarmed; striae not impressed, glabrous, with moderate punctures separated by one diameter of a puncture; interstriae flat, sparsely finely uniseriate punctate, punctures 1/3 those of striae, each bearing erect hair-like setae, setae approximately as long as width of interstriae 2. Declivital face steeply rounded, weakly sulcate between interstriae 3, smooth, shiny, striae and interstriae moderately setose, setae long, semi-erect hair-like, as long or longer than the distance between suture and interstriae 3; three striae present, striae 1 and 2 subcontiguous; strial punctures as larger, deeper than on disc; interstriae 1 and 2 flat, armed by two and four denticles, respectively, on declivital base, unarmed on face; interstriae 2 very narrow; interstriae 3 forming declivital margin, distinctly elevated, 2–4 pairs of small, narrow, sharply pointed spines of equal size (often asymmetric) on basal 2/3. Posterolateral margin rounded, denticulate from interstriae 3–7. ***Legs***: procoxae contiguous, prosternal coxal piece flat, inconspicuous. Protibiae obliquely triangular, broadest at apical 1/3; posterior face smooth; apical 1/3 of outer margin with six moderate socketed denticles, their length approximately as long as basal width. Meso- and metatibiae flattened, outer margin evenly rounded with nine and seven moderate socketed denticles, respectively.

##### Etymology.

G. *echinos*- = hedgehog or sea urchin; -*pteron* = wing. In reference to the acute spines on the declivity. The last element has been Latinized as a second declension noun. A noun in apposition.

##### Distribution.

China (Hong Kong).

##### Host plants.

Unknown.

#### Xyleborinus ephialtodes sp. nov.

Taxon classificationAnimaliaColeopteraCurculionidae

http://zoobank.org/A9B9FD8C-BD45-4026-8F6F-940B40992056

Fig. 83E, F, K

##### Type material.

***Holotype***, female, China: Fujian, Zhangzhou, 14.xii.2017, Shouping Cai, Haitian Song, ex *Schima superba* (IZAS).

##### Diagnosis.

2.6 mm long (n = 1); 2.6× as long as wide. This species is distinguished by the discal interstriae confused; posterior margin of elytra broadly rounded; declivital face with interstriae 1 and 2 granulate, unarmed by tubercles; declivital face weakly sulcate; large body size; lateral declivital margins elevated, bearing five pairs of moderate, narrow, sharply pointed spines of equal size; sulcate area of declivity shagreened, dull; and stout body.

##### Similar species.

*Xyleborinus artestriatus*, *X. echinopterus*, *X. octiesdentatus*, *X. schaufussi*, *X. speciosus*, *X. spinipennis*.

##### Description

**(female).** 2.6 mm long (n = 1); 2.6× as long as wide. Body dark red-brown. Legs and antennae light brown. ***Head***: epistoma entire, transverse, with a row of hair-like setae. Frons weakly convex to upper level of eyes, alutaceous, subshiny, finely, densely punctate, setose; punctures bearing a long, erect hair-like seta. Eyes shallowly emarginate just above antennal insertion, upper part smaller than lower part. Submentum large, distinctly triangular, deeply impressed. Antennal scape regularly thick, as long as club. Pedicel as wide as scape, as long as funicle. Funicle 4-segmented, segment 1 shorter than pedicel. Club wider than long, obliquely truncate, type 1; segment 1 corneous, encircling anterior face; segment 2 narrow, concave, corneous on anterior face only; sutures absent on posterior face. ***Pronotum***: 0.9× as long as wide. Basic and parallel-sided, type 2 in dorsal view, sides parallel in basal 2/3, rounded anteriorly; anterior margin without serrations. In lateral view type 7, elongate, disc longer than anterior slope, summit low, on apical 2/5. Anterior slope with densely spaced narrow asperities, becoming lower and more strongly transverse towards summit, bearing long, fine, semi-recumbent hair-like setae. Disc subshiny, alutaceous, finely punctate, finely setose, setae short, erect hair-like, some longer hair-like setae at margins. Lateral margins obliquely costate. Base transverse, posterior angles acutely rounded. ***Elytra***: 1.62× as long as wide, 1.78× as long as pronotum. Scutellum minute, conical, disconnected from elytra, surrounded by dense mycangial tuft of setae. Elytral base transverse, medially emarginate near scutellum and mycangial tuft, edge oblique, humeral angles angulate, parallel-sided in basal 3/4, then broadly rounded to apex. Disc occupying basal 3/5, smooth, shiny, glabrous, unarmed; striae not impressed, glabrous, with small punctures separated by two diameters of a puncture; interstriae flat, sparsely finely punctate, punctures confused, 1/2 those of striae, glabrous. Declivital face shagreened, dull, steeply rounded, weakly sulcate between interstriae 3, striae and interstriae densely setose, setae long, erect hair-like, as long as the distance between suture and interstriae 3; three striae present; strial punctures as larger, deeper than on disc; interstriae 1 and 2 flat, granulate along their length; interstriae 2 very narrow; interstriae 3 forming declivital margin, distinctly elevated, bearing five pairs of moderate, narrow, sharply pointed spines of equal size. Posterolateral margin without a costa, spinose and denticulate from interstriae 3–7, apical pair largest. ***Legs***: procoxae contiguous; prosternal coxal piece tall, pointed. Protibiae obliquely triangular, broadest at apical 1/3; posterior face smooth; apical 1/3 of outer margin with seven moderate socketed denticles, their length approximately as long as basal width. Meso- and metatibiae flattened; outer margin evenly rounded with ten and eight moderate socketed denticles, respectively.

##### Etymology.

G. *ephialtes* = nightmare; -*odes* = resembling. In reference to the nightmarish long, acute spines on the declivity. A noun in apposition.

##### Distribution.

China (Fujian).

##### Host plants.

This species is only known from *Schima* (Theaceae).

#### Xyleborinus exiguus

Taxon classificationAnimaliaColeopteraCurculionidae

(Walker, 1859)

Fig. 83G, H, L

Bostrichus exiguus Walker, 1859: 260.
Xyleborinus exiguus (Walker): Maiti and Saha 1986: 109.
Xyleborus muriceus Eichhoff, 1878a: 392. Synonymy: Eggers 1925: 154.
Xyleborus diversus Schedl, 1954b: 80. syn. nov.
Xyleborus perexiguus Schedl, 1971b: 381. Synonymy: Hulcr and Cognato 2013: 142.
Xyleborus ankius Schedl, 1975c: 361. Synonymy: Hulcr and Cognato 2013: 142.

##### Type material.

***Holotype****Bostrichus exiguus* (NHMUK). ***Paralectotype****Xyleborus diversus* (NHMUK). The holotype of *Xyleborus muriceus* was destroyed in the bombing of UHZM in World War II (Wood and Bright 1992).

##### New records.

Cambodia: Kumpong Speu, Oral mountain foot, Cardamom [Mts.], 25–31.i.2006, Oul Yothin, Malaise trap (IRSNB, 1). China: Jiangxi, Shang Rao, 31.viii.2016, Lv-Jia, Lai, S-C., ex *Prunus* sp. (RABC, 1). S Yunnan, Xishuangbanna, 20 km NW Jinghong, vic. Man Dian (NNNR), 22°07.80'N, 100°40.05'E, 740 m, rubber plantation., 10.x.2008, A. Weigel (RABC, 1); as previous except: 23 km NW Jinghong, vic. Na Ban village (NNNR), 22°10'N, 100°39'E, 700–1000 m, v–vii. 2009, L. Meng (NKME, 10; RABC, 3). Laos: Vientiane, Ban Van Eue, 30.ii.1965 (BPBM, 1). Taiwan: Nantou, Sun Moon Lake, 29.xii.2012, Lin, C-S. (CSLC, 1). Vietnam: Dong Nai, Cat Tien N.P., 11.40817, 107.38098, 134 m, 20–22.ii.2017, VN81, A.I. Cognato, T.A. Hoang, ex FIT (MSUC, 2).

##### Diagnosis.

1.8–2.0 mm long (mean = 1.88 mm; n = 5); 2.57–3.0× as long as wide. This species is distinguished by the declivital face with interstriae 2 unarmed by tubercles; elytral apex attenuated, with three (usually) pairs of large flattened tubercles; and declivital interstriae flat, interstriae 2 not impressed.

##### Similar species.

*Xyleborinus huifenyinae*, *X. perpusillus*, *X. tritus*.

##### Distribution.

Recorded in the study region from India (Andaman Is), Cambodia*, China* (Jiangxi, Yunnan), Laos*, Myanmar, Nepal, Taiwan*, Thailand, Vietnam. Also recorded from American Samoa, Australia, Cook Is, Federated States of Micronesia, Fiji, Guam, Indonesia (Java, Maluku, Sulawesi, Sumatra), East & West Malaysia, Mariana Is., New Guinea, Niue I., Philippines, Samoa, Solomon Islands, Tahiti. Introduced to West Africa (Angola, Congo, Equatorial Guinea, Gabon, Ghana, Ivory Coast) (Beaver 2005, Wood and Bright 1992) and Central America (Costa Rica, Panama) (Kirkendall and Ødegaard 2007).

##### Host plants.

Polyphagous (Browne 1961b).

##### Remarks.

A paralectotype of the West African species, *Xyleborus diversus* (NHMUK), has been compared directly with the holotype of the Oriental species, *Xyleborinus exiguus* (NHMUK), and is clearly conspecific. *X. diversus* is here placed in synonymy with *X. exiguus*. Kalshoven (1959b) gives details of brood sizes in various hosts in relation to the size of the brood chamber.

#### Xyleborinus huifenyinae sp. nov.

Taxon classificationAnimaliaColeopteraCurculionidae

http://zoobank.org/55245CA8-39ED-4ECC-837B-32BF5BD2001B

Fig. 84A, B, I

##### Type material.

***Holotype***, female, China: Jiangxi, Xunwu, Xiangshan, You Li, 10.x.2018, ex Fagaceae log (IZAS).

##### Diagnosis.

1.7 mm long (n = 1); 2.83× as long as wide. This species is distinguished by declivital interstriae 2 unarmed on face (armed at summit); granules at declivital summit extending to apical quarter of disc; declivital posterolateral margin costate and denticulate; declivital face shagreened, dull; declivital interstriae flat, interstriae 2 not impressed; and denticles on interstriae 1 and 3 prominent.

This species is very similar to *X. perpusillus* and is distinguished by the shagreened declivity, posterolateral declivity margin costate and denticulate, and larger denticles on interstriae 1 and 3.

##### Similar species.

*Xyleborinus exiguus*, *X. perpusillus*, *X. tritus*.

##### Description

**(female).** 1.7 mm long (n = 1); 2.83× as long as wide. Body dark brown, pronotum lighter than elytra. Legs and antennae light brown. ***Head***: epistoma entire, transverse, with a row of hair-like setae. Frons weakly convex to upper level of eyes, alutaceous, subshiny, finely and sparsely punctate, setose; punctures bearing a long, erect hair-like seta. Eyes shallowly emarginate just above antennal insertion, upper part smaller than lower part. Submentum large, distinctly triangular, slightly impressed. Antennal scape short and thick, as long as club. Pedicel as wide as scape, as long as funicle. Funicle 4-segmented, segment 1 shorter than pedicel. Club approximately circular, obliquely truncate, type 1; segment 1 corneous, encircling anterior face; segment 2 narrow, concave, corneous on anterior face only; sutures absent on posterior face. ***Pronotum***: 1.06× as long as wide. In dorsal view long and rounded frontally, type 7, sides parallel in basal 2/3, rounded anteriorly; anterior margin without serrations. In lateral view type 7, elongate, disc much longer than anterior slope, summit low, on anterior 1/3. Anterior slope with densely spaced narrow asperities, becoming lower and more strongly transverse towards summit, bearing long, fine, semi-recumbent, hair-like setae. Disc subshiny, alutaceous, finely punctate, finely setose, setae short, erect, hair-like, some longer hair-like setae at margins. Lateral margins obliquely costate. Base transverse, posterior angles acutely rounded. ***Elytra***: 1.69× as long as wide, 1.58× as long as pronotum. Scutellum minute, conical, disconnected from elytra, surrounded by dense mycangial tuft of setae. Elytral base transverse, medially emarginate near scutellum and mycangial tuft, edge oblique, humeral angles angulate, parallel-sided in basal 3/4, then weakly rounded to apex. Disc occupying basal 2/3, smooth, shiny, interstriae 1–3 granulate on apical quarter; striae not impressed, with small punctures separated by two diameters of a puncture setose, setae recumbent, as long as the distance between punctures; interstriae flat, sparsely finely uniseriate punctate, punctures 1/3 those of striae, each bearing erect hair-like setae, setae approximately as long as width of interstriae 2. Declivital face strongly shagreened, dull, steeply rounded, feebly sulcate between interstriae 3; three parallel striae present; interstriae impunctate, flat; interstriae 1 and 3 armed by four prominent denticles along its length; interstriae 2 unarmed along its length, one small denticle at summit; interstrial denticles and spines setose, setae erect, hair-like, uniseriate and as long as the width between suture and interstriae 3. Posterolateral margin costate, tuberculate to interstriae 7. ***Legs***: procoxae contiguous; prosternal coxal piece tall, conical. Protibiae obliquely triangular, broadest at apical 1/3; posterior face smooth; apical 1/3 of outer margin with six moderate socketed denticles, their length approximately as long as basal width. Meso- and metatibiae flattened; outer margin evenly rounded with eight and seven moderate socketed denticles, respectively.

##### Etymology.

Named for Dr. Hui-Fen Yin and her contribution to the understanding of the Chinese scolytine fauna. Noun in genitive.

##### Distribution.

China (Fujian).

##### Host plants.

This species is only known from Fagaceae.

**Figure 84.**
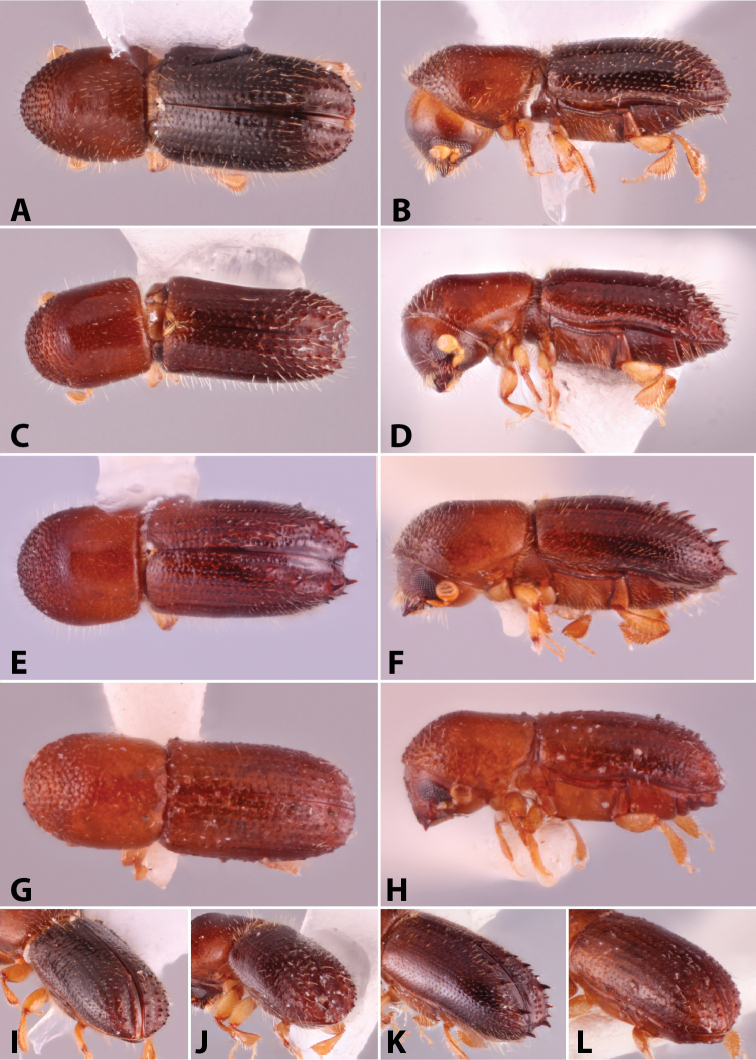
Dorsal, lateral and declivital view of *Xyleborinus huifenyinae* holotype, 1.7 mm (**A, B, I**), *X. jianghuasuni* holotype, 1.8 mm (**C, D, J**), *X. octiesdentatus*, 2.5–2.65 mm (**E, F, K**), and *X. perpusillus*, 1.6–1.9 mm (**G, H, L**).

#### Xyleborinus jianghuasuni sp. nov.

Taxon classificationAnimaliaColeopteraCurculionidae

http://zoobank.org/17D51C13-87C1-4FF2-8901-09844BE0E243

Fig. 84C, D, J

##### Type material.

***Holotype***, female, China: Yunnan, Xishuangbanna, Sanchahe Nat[ure]. Res[erve]., 22°09.784'N, 100°52.256'E, 2186 m, 29–30.v.2008, A.I. Cognato, ex *Quercus* (IZAS). ***Paratypes***, female, as holotype (MSUC, 2).

##### Diagnosis.

1.8 mm long (n = 3); 3.0× as long as wide. This species is distinguished by the posterior margin of elytra broadly rounded; declivital face with interstriae 1 and 2 unarmed by tubercles; declivital face feebly sulcate; small body size; lateral declivital margins weakly elevated, bearing three pairs of sharply pointed denticles, denticles subequal; denticles along interstriae 5 subequal to those on interstriae 3; sulcate area impunctate, surface strongly shagreened, dull; and elongate body form.

##### Similar species.

*Xyleborinus cuneatus*, *X. disgregus*, *X. sculptilis*.

##### Description

**(female).** 1.8 mm long (n = 3); 3.0× as long as wide. Body uniformly red-brown. Legs and antennae light brown. ***Head***: epistoma entire, transverse, with a row of hair-like setae. Frons weakly convex to upper level of eyes, alutaceous, subshiny, finely, sparsely punctate, setose; punctures bearing a long, erect hair-like seta. Eyes deeply emarginate just above antennal insertion, upper part smaller than lower part. Submentum narrowly triangular, deeply impressed. Antennal scape short and thick, as long as club. Pedicel as wide as scape, as long as funicle. Funicle 4-segmented, segment 1 shorter than pedicel. Club longer than wide, obliquely truncate, type 1; segment 1 corneous, encircling anterior face; segment 2 narrow, concave, corneous on anterior face only; sutures absent on posterior face. ***Pronotum***: 1.25× as long as wide. In dorsal view very elongate, rounded frontally, type 9, sides parallel on basal 3/4; anterior margin without serrations. In lateral view elongate with disc much longer than anterior slope, type 8, disc flat, summit moderately prominent, at apical 1/3. Anterior slope with densely spaced narrow asperities, becoming lower and more strongly transverse towards summit, bearing long, fine, semi-recumbent, hair-like setae. Disc subshiny, alutaceous, finely punctate, finely setose, setae short, erect, hair-like, some longer hair-like setae at margins. Lateral margins obliquely costate. Base transverse, posterior angles acutely rounded. ***Elytra***: 1.93× as long as wide, 1.54× as long as pronotum. Scutellum minute, conical, disconnected from elytra, surrounded by dense mycangial tuft of setae. Elytral base transverse, medially emarginate near scutellum and mycangial tuft, edge oblique, humeral angles angulate, parallel-sided in basal 3/4, then broadly rounded to apex. Disc occupying basal 2/3, smooth, shiny, unarmed; striae not impressed, with large punctures separated by one diameter of a puncture, setose, setae recumbent, as long as the diameter of a puncture; interstriae flat, sparsely finely uniseriate punctate, punctures 1/4 those of striae, each bearing erect hair-like setae, setae approximately as long as width of interstriae 2. Declivital face strongly shagreened, dull, steeply rounded, feebly sulcate between interstriae 3, impunctate; three parallel striae present; interstriae impunctate, flat; interstriae 1 unarmed on face, two or three denticles present at base; interstriae 2 unarmed along its length, one small denticle at base; interstriae 3 weakly elevated, bearing three pairs of sharply pointed denticles, denticles subequal; interstrial denticles setose, setae erect, hair-like, uniseriate and as long as the width between suture and interstriae 3. Posterolateral margin costate, granulate to interstriae 7. ***Legs***: procoxae contiguous; prosternal coxal piece tall and pointed. Protibiae obliquely triangular, broadest at apical 1/3; posterior face smooth; apical 1/3 of outer margin with five moderate socketed denticles, their length approximately as long as basal width. Meso- and metatibiae flattened; outer margin evenly rounded with seven moderate socketed denticles.

##### Etymology.

In gratitude for the assistance of Dr. Jianghua Sun (Chinese Academy of Sciences) who facilitated AIC’s access to wild China. Noun in genitive.

##### Distribution.

China (Yunnan).

##### Host plants.

This species is only known from *Quercus* (Fagaceae).

#### Xyleborinus octiesdentatus

Taxon classificationAnimaliaColeopteraCurculionidae

(Murayama, 1931)

Fig. 84E, F, K

Xyleborus octiesdentatus Murayama, 1931: 46.
Xyleborinus octiesdentatus (Murayama): Beaver et al. 2008: 234.

##### Type material.

***Syntypes*** (NMNH, 4).

##### New records.

Vietnam: Cao Bang, 22°33.9981'N, 105°52.591'E, 1051 m, 12–17.iv.2014, VN12, Cognato, Smith, Pham (MSUC, 1).

##### Diagnosis.

2.50–2.65 mm long (mean = 2.55 mm; n = 5); 2.79–3.13× as long as wide. This species is distinguished by the posterior margin of elytra broadly rounded; declivital face with interstriae 1 and 2 unarmed by tubercles; declivital face moderately sulcate; large body size; lateral declivital margins elevated, bearing 4–6 pairs of long, narrow, sharply pointed spines (often asymmetric), increasing in length from base to apex; sulcate area of declivity smooth, shiny; and elongate body form.

##### Similar species.

*Xyleborinus artestriatus*, *X. echinopterus*, *X. ephialtodes*, *X. schaufussi*, *X. speciosus*, *X. spinipennis*.

##### Distribution.

China (Sichuan), Japan, South Korea, Vietnam*. Imported and established in USA (Rabaglia et al. 2010; Gomez et al. 2018a).

##### Host plants.

Recorded from *Carpinus* (Betulaceae), *Illicium* (Illiciaceae), *Cleyera* and *Eurya* (Theaceae) (Wood and Bright 1992).

#### Xyleborinus perpusillus

Taxon classificationAnimaliaColeopteraCurculionidae

(Eggers, 1927)

Fig. 84G, H, L

Xyleborus perpusillus Eggers, 1927a: 404.
Xyleborinus perpusillus (Eggers): Hulcr 2010: 115.
Xyleborus perminutissimus Schedl, 1934b: 90. Synonymy: Hulcr 2010: 116.
Xyleborus angustatulus Schedl, 1942c: 42. Synonymy: Kalshoven 1959a: 152.

##### Type material.

***Paratype****Xyleborus perpusillus* (NMNH).

##### Diagnosis.

1.6–1.9 mm long (mean = 1.76 mm; n = 5); 2.67–3.17× as long as wide. This species is distinguished by the entire length of interstriae 2 unarmed by tubercles; posterolateral margin of elytra rounded, unarmed; declivital interstriae flat, interstriae 2 not impressed; and denticles on interstriae 1 and 3 small.

This species is very similar to *X. huifenyinae* and is distinguished by the shiny declivity, posterolateral declivity margin rounded and unarmed and smaller denticles on interstriae 1 and 3.

##### Similar species.

*Xyleborinus exiguus*, *X. huifenyinae*, *X. tritus*.

##### Distribution.

Indonesia (Java, Sumatra), East & West Malaysia, New Guinea, Thailand.

##### Host plants.

Recorded from small trees in several families, including palms (Arecaceae) (Beaver et al. 2014).

#### Xyleborinus saxesenii

Taxon classificationAnimaliaColeopteraCurculionidae

(Ratzeburg, 1837)

Fig. 85A, B, I

Bostrichus saxesenii Ratzeburg, 1837: 167.
Xyleborinus saxesenii (Ratzeburg): Reitter 1913: 79.
Xyleborus dohrni Wollaston, 1854: 290. Synonymy: Eichhoff 1878b: 362.
Xyleborus decolor Boieldieu, 1859: 473. Synonymy: Ferrari 1867: 22.
Xyleborus aesculi Ferrari, 1867: 22. Synonymy: Eichhoff 1878b: 362.
Xyleborus sobrinus Eichhoff, 1876a: 202. Synonymy: Schedl 1964d: 313.
Xyleborus subdepressus Rey, 1883: 142. Synonymy: Bedel 1888: 419.
Xyleborus frigidus Blackburn, 1885: 193. Synonymy: Samuelson 1981: 59.
Xyleborus floridensis Hopkins, 1915a: 60, 63. Synonymy: Wood 1962: 79.
Xyleborus pecanis Hopkins, 1915a: 60, 63. Synonymy: Wood 1962: 79.
Xyleborus quercus Hopkins, 1915a: 60, 63. Synonymy: Wood 1962: 79.
Xyleborus arbuti Hopkins, 1915a: 61, 64. Synonymy: Wood 1957: 403.
Xyleborinus tsugae Swaine, 1934: 204. Synonymy: Wood 1957: 403.
Xyleborinus librocedri Swaine, 1934: 205. Synonymy: Wood 1957: 403.
Xyleborus pseudogracilis Schedl, 1937c: 169. Synonymy: Wood 1989: 176.
Xyleborus retrusus Schedl 1940b, 208. Synonymy: Wood 1989: 176.
Xyleborus peregrinus Eggers, 1944: 142. Synonymy: Schedl 1980: 122.
Xyleborus pseudoangustatus Schedl, 1948: 28. Synonymy: Schedl 1964d: 313.
Xyleborus paraguayensis Schedl, 1949: 276. Synonymy: Wood 1989: 176.
Xyleborus opimulus Schedl, 1976: 77. Synonymy: Wood 2007: 473.

##### Type material.

***Holotype****Xyleborus floridensis* (NMNH). ***Holotype****Xyleborus pecanis* (NMNH). ***Holotype****Xyleborus quercus* (NMNH).

##### New records.

China: Chongqing, Jinfo Mtn, 9.v.2016, Tian-Shang, Lv-Jia, ex *Ficus* sp. (RABC, 2). Hong Kong, Kadoorie Farm, vi.2017, J. Skelton (UFFE, 1). Shanghai, Dongchuan, vii–viii.2017, Gao, ex trap w/ querciverol (MSUC, 4). Vietnam: Cao Bang, 22°36.402'N, 105°52.397'E, 1601 m, 13.iv.2014, VN17, Cognato, Smith, Pham, ex standing stump (MSUC, 1).

##### Diagnosis.

2.3–2.5 mm long (mean = 2.34 mm; n = 5); 3.13–3.29× as long as wide. This species is distinguished by the declivital face with interstriae 2 armed by granules at declivital summit, unarmed on declivital face; declivital interstriae 1 and 3 denticles subacutely pointed; denticles on ventrolateral areas of the elytra small, less acute; discal interstriae 1 and 2 unarmed; declivital interstriae 2 flattened; and moderate size.

##### Similar species.

*Xyleborinus attenuatus*, *X. subgranulatus*, *X. subspinosus*, *X. thaiphami*.

##### Distribution.

Occurs throughout the Palaearctic region. Recorded in the study region from China (Anhui, Chongqing*, Fujian, Guangxi, Guizhou, Hebei, Heilongjiang, Hong Kong*, Hunan, Jiangsu, Jiangxi, Jilin, Ningxia, Shaanxi, Shanghai*, Shanxi, Sichuan, Xizang, Yunnan, Zhejiang), India (Assam, Kashmir, Uttarakhand, West Bengal), Taiwan, Vietnam. Outside the Palaearctic, introduced and established in American Samoa, Australia, Hawaii, New Zealand, South Africa, North America (Canada, United States, Mexico) and several countries in South America (Wood and Bright 1992; Kirkendall 2018).

##### Host plants.

Strongly polyphagous attacking both gymnosperms and angiosperms (Wood and Bright 1992).

##### Remarks.

The biology of the species has been studied by Fischer (1954), Egger (1973), Hosking (1973), Peer and Taborsky (2007), Biedermann (2010), Biedermann and Taborsky (2011) and others. The larvae enlarge the gallery system as they develop, and frequently feed on fungus-infested wood rather than the ambrosia fungus alone (Wood 1982; Biedermann et al. 2009). Peer and Taborsky (2007) show that cooperative brood care occurs within the gallery system as a result of delayed dispersal by the new generation of females and that this can raise the number of offspring produced per gallery. The species is strongly attracted to ethanol (e.g., Markalas and Kalapanida 1997; Saruhan and Akyol 2012). It is a pest of hazelnut in the Mediterranean area (Saruhan and Akyol 2012), and of stressed trees in fruit orchards and forest plantations. Damage to timber is also caused by the galleries and associated staining of the wood (Chararas 1962).

**Figure 85.**
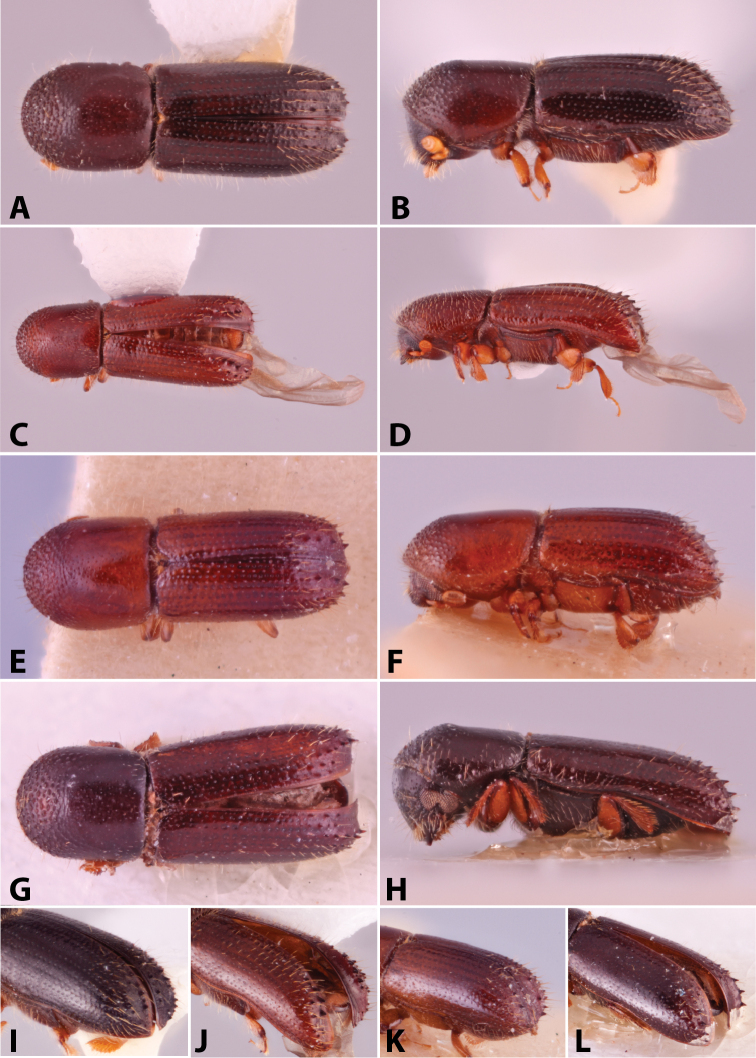
Dorsal, lateral and declivital view of *Xyleborinus saxesenii*, 2.3–2.5 mm (**A, B, I**), *X. schaufussi*, 2.5–3.1 mm (**C, D, J**), *X. sculptilis* paratype, 2.1–2.25 mm (**E, F, K**), and *X. speciosus* holotype, 2.6–2.7 mm (**G, H, L**).

#### Xyleborinus schaufussi

Taxon classificationAnimaliaColeopteraCurculionidae

(Blandford, 1894)

Fig. 85C, D, J

Xyleborus schaufussi Blandford, 1894b: 117.
Xyleborinus schaufussi (Blandford): Wood and Bright 1992: 816.
Xyleborus kraunhiae Niisima, 1910: 14. Synonymy: Smith et al. 2018b: 397.

##### Type material.

***Syntypes****Xyleborus schaufussi* (NHMUK). ***Syntypes*** of *Xyleborus kraunhiae* should be housed in NIAES but have not been located (Smith et al. 2018b).

##### New records.

China: Guizhou, Guiyang, Huaxi, 31.iv.2015, Guangyu Liu, ex ethanol trap (UFFE, 1). Sichuan, Tongjiang, 1.viii.1980, Yongguang Shen, ex *Betula* sp. (NMNH, 1).

##### Diagnosis.

2.5–3.1 mm long (mean = 2.73 mm; n = 5); 3.21–3.57× as long as wide. This species is distinguished by the posterior margin of elytra broadly rounded; declivital face with interstriae 1 and 2 unarmed by tubercles; declivital face strongly sulcate; large body size; lateral declivital margins strongly elevated, costate, bearing 4–6 pairs of large sharply pointed backwardly hooked denticles (often asymmetric); sulcate area of declivity impunctate, shagreened, dull; and elongate body form.

##### Similar species.

*Xyleborinus artestriatus*, *X. echinopterus*, *X. ephialtodes*, *X. octiesdentatus*, *X. speciosus*, *X. spinipennis*.

##### Distribution.

China* (Guizhou, Sichuan), Japan, Taiwan.

##### Host plants.

Recorded from *Millettia* (Fabaceae) (Niisima 1910), *Fagus* (Fagaceae) and *Symplocos* (Symplocaceae) (Beaver and Liu 2010), and *Betula* (Betulaceae).

#### Xyleborinus sculptilis

Taxon classificationAnimaliaColeopteraCurculionidae

(Schedl, 1964)

Fig. 85E, F, K

Xyleborus sculptilis Schedl, 1964b: 247.
Xyleborinus sculptilis (Schedl): Wood and Bright 1992: 816.

##### Type material.

***Paratype*** (NHMW).

##### New records.

Taiwan: Taipei City, TFRI Botanical Garden, 12.iii.2014, L.J. Wang, ex log (RABC, 1).

##### Diagnosis.

2.1–2.25 mm long (mean = 2.16 mm; n = 4); 2.81–3.14× as long as wide. This species is distinguished by the posterior margin of elytra broadly rounded; declivital face with interstriae 1 and 2 unarmed by tubercles; declivital face weakly sulcate; small body size; lateral declivital margins feebly elevated, bearing three small pairs of sharply pointed denticles, denticles increasing in size from base to apex; sulcate area punctate, surface smooth, opalescent; and elongate body form.

##### Similar species.

*Xyleborinus cuneatus*, *X. disgregus*, *X. jianghuasuni*.

##### Distribution.

Brunei, Laos, East Malaysia, Taiwan*, Thailand.

##### Host plants.

Recorded from *Artocarpus* (Moraceae) and *Mangifera* (Anacardiaceae) (Schedl 1964b; Sittichaya 2012).

#### Xyleborinus speciosus

Taxon classificationAnimaliaColeopteraCurculionidae

(Schedl, 1975)

Fig. 85G, H, L

Xyleborus speciosus Schedl, 1975b: 457.
Xyleborinus speciosus (Schedl): Wood and Bright 1992: 816.

##### Type material.

***Holotype*** (NHMW).

##### New records.

China: S. Yunnan, Xishuangbanna, 37 km NW Jinghong, vic. Guo Men Shan, 22°14.48'N, 100°36.22'E, 1080 m, 28.vi.2008, L. Meng (RABC, 1). Thailand: Chiang Mai, Doi Pui, 1400 m, EtOH trap, various dates from 6.ix–12.xi.2004, 10–31.x.2005, 8–12.v.2006, W. Puranasakul (RABC, 7).

##### Diagnosis.

2.6–2.7 mm long (mean = 2.65 mm; n = 3); 2.89–3.0× as long as wide. This species is distinguished by the posterior margin of elytra broadly rounded; declivital face with interstriae 1 and 2 unarmed by tubercles; declivital face weakly sulcate; large body size; lateral declivital margins elevated along interstriae 3, bearing three pairs of short, narrow, sharply pointed spines, spines equal in size; sulcate area of declivity smooth, shiny; interstriae impunctate; and elongate body form.

##### Similar species.

*Xyleborinus artestriatus*, *X. ephialtodes*, *X. echinopterus*, *X. octiesdentatus*, *X. schaufussi*, *X. spinipennis*.

##### Distribution.

China* (Yunnan), India (West Bengal), Thailand*.

##### Host plants.

Recorded from *Juglans* (Juglandaceae), *Litsea* (Lauraceae), *Prunus* (Rosaceae) and *Symplocos* (Symplocaceae) (Saha and Maiti 1996, as *Xyleborinus subspinosus* (Eggers)).

##### Remarks.

This species appears to have been misidentified by Saha and Maiti (1996) and Maiti and Saha (2004) as *Xyleborinus subspinosus*, a synonym of *X. saxesenii* (see above). It was misidentified by Beaver et al. (2014) as *Xyleborinus spinipennis* (Eggers).

#### Xyleborinus spinipennis

Taxon classificationAnimaliaColeopteraCurculionidae

(Eggers, 1930)

Fig. 86A, B, I

Xyleborus spinipennis Eggers, 1930: 202.
Xyleborinus spinipennis (Eggers): Wood and Bright 1992: 817.

##### Type material.

***Holotype*** (FRI).

##### New records.

China: Sichuan, Mt. Emei, 1000 m, 4–20.v.1989, V. Kubáň (RABC, 1); as previous except: 600–1050 m, 5–19.v.1989, L. Bocák (NHMB, 1). Nepal: W., Dhawalagiri, Parbat Distr., Karkineta–Nagdanda, 1600 m, 3.vii.1986, C. Holzschuh (RABC, 1). Vietnam: Cao Bang, 22°36.402'N, 105°52.397'E, 1601 m, 13.iv.2014, VN17, Cognato, Smith, Pham, ex standing stump (MSUC).

##### Diagnosis.

2.3–2.75 mm long (mean = 2.56 mm; n = 5); 2.83–2.89× as long as wide. This species is distinguished by the elytra parallel-sided in basal 1/2, tapering posteriorly; declivital face with interstriae 1 and 2 unarmed by tubercles; declivital face moderately sulcate; small body size; lateral declivital margins moderately elevated, costate, bearing 4–6 pairs of large sharply pointed backwardly hooked denticles (often asymmetric); strial punctures large; elytra with small denticles on interstriae 1–4 not extending further than the declivital summit; declivital surface shagreened, dull; and elongate body form.

This species is very similar to *X. cuneatus* and is distinguished by the larger size, less elongate form, elytra with small denticles on interstriae 1–4 not extending further than the declivital summit, pronotum less elongate, 1.14× as long as wide.

##### Similar species.

*Xyleborinus artestriatus*, *X. cuneatus*, *X. echinopterus*, *X. ephialtodes*, *X. octiesdentatus*, *X. schaufussi*, *X. speciosus*.

##### Distribution.

China* (Sichuan), India (Assam), Nepal*, Vietnam*.

##### Host plants.

Unknown.

##### Remarks.

Eggers (1930) stated that the species was 2.0 mm long in his description. The holotype was measured by S.L. Wood and was found to be 2.4 mm long.

**Figure 86.**
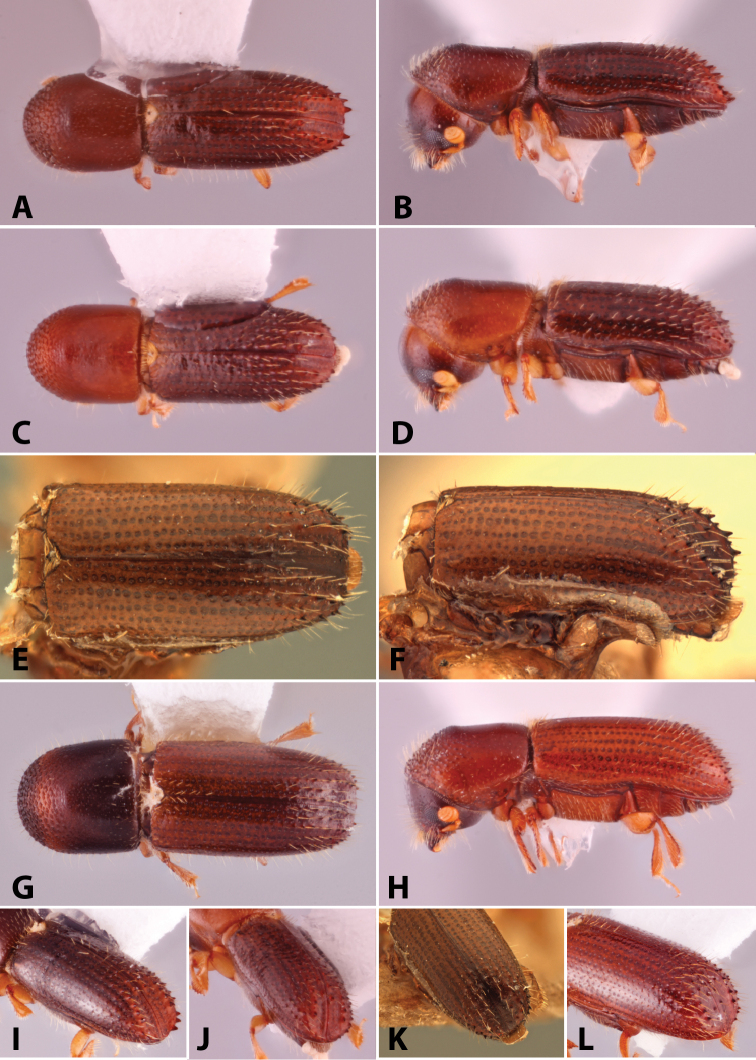
Dorsal, lateral and declivital view of *Xyleborinus spinipennis*, 2.3–2.75 mm (**A, B, I**), *X. subgranulatus*, 1.6–1.8 mm (**C, D, J**), *X. subspinosus* holotype, 2.3 mm (**E, F, K**), and *X. thaiphami* holotype, 2.8–2.9 mm (**G, H, L**).

#### Xyleborinus subgranulatus

Taxon classificationAnimaliaColeopteraCurculionidae

(Eggers, 1930)

Fig. 86C, D, J

Xyleborus subgranulatus Eggers, 1930: 202.
Xyleborinus subgranulatus (Eggers): Wood and Bright 1992: 817.

##### Type material.

***Holotype*** (FRI), ***paratype*** (NMNH).

##### New records.

China: Yunnan, Gaoligong Mts., 25.22N, 98.49E, 1500–2500 m, 17–24.v.1995, V. Kubáň (NKME, 1); S Yunnan, Xishuangbanna, 29 km NW Jinghong, vic. Da Nuo You NNNR, 22°12.41'N, 100°38.29'E, 790 m, fallow GF, 23.v.2008, A. Weigel (RABC, 1); as previous except: 37 km NW Jinghong, vic. Guo Men Shan, 22°14.48'N, 100°36.22'E, 1080 m, forest, EKL, 16.iii.2009, L. Meng (NKME, 1). Laos: NE, Houa Phan, Phou Pane mt., 20°13'09–19"N, 103°59'54"–104°00'03"E, 1480–1510 m, 22.iv–14.v.2008, V. Kubáň (MNHP, 1). Taiwan: Yilan co., Fushan, v.2009, ex sticky trap (RABC, 1). Vietnam: Cao Bang, 22°34.118'N, 105°52.537'E, 1048 m, 12.iv.2014, VN13, Cognato, Smith, Pham, ex large felled *Pinus* sp. (MSUC, 1). Vinh Phuc, Tam Dao, 6–9.v.1990, P. Pachlolátko (RABC, 1). Tuyen Quang, 3 km SE Pac Ban village, Na Hang Nature Reserve, 22.20; 105.25, 380 m, at light, 22–26.ii.1997, G. Csorba (HNHM, 1).

##### Diagnosis.

1.6–1.8 mm long (mean = 1.7 mm; n = 5); 3.0–3.4× as long as wide. This species is distinguished by the declivital face with interstriae 2 armed by granules at declivital summit, unarmed on declivital face; declivital interstriae 1 and 3 denticles subacutely pointed; denticles on ventrolateral areas of the elytra small, less acute; declivital interstriae 2 slightly impressed; discal interstriae 1 and 2 granulate, granules extending from declivital summit to mid-point of disc; and minute size.

##### Similar species.

*Xyleborinus attenuatus*, *X. saxesenii*, *X. subspinosus*, *X. thaiphami*.

##### Distribution.

China* (Yunnan), India (Assam, West Bengal), Laos*, Taiwan*, Thailand, Vietnam*,

##### Host plants.

Recorded from four genera in four different families (Maiti and Saha 2004) as well as *Pinus* (Pinaceae) and is presumably polyphagous.

##### Remarks.

This species is strikingly similar to, and has been recovered as, sister to *Xyleborinus saxesenii* using COI data (Cognato et al. 2020b) but the elytral morphology is inconsistent within single specimens. Given its close relationship with *X. saxesenii* the species is expected to have denticles along declivital interstriae 1 and 3. However this is not always the case and the species can also have declivital interstriae 1 and 2 unarmed with denticles along interstriae 3. Both tubercle patterns have been found on each elytron of single individuals, including the paratype examined and individuals from a series collected in [West] Bengal.

#### Xyleborinus subspinosus

Taxon classificationAnimaliaColeopteraCurculionidae

(Eggers, 1930) stat. res.

Fig. 86E, F, K

Xyleborus subspinosus Eggers, 1930: 203.
Xyleborinus subspinosus (Eggers): Saha and Maiti 1987: 73.

##### Type material.

***Holotype*** (FRI).

##### Diagnosis.

2.3 mm long (Eggers 1930). This species is distinguished from other Southeast Asian species by the unique sculpturing of the declivity with interstriae 1 unarmed and interstriae 2 and 3 denticulate along their lengths.

##### Similar species.

*Xyleborinus attenuatus*, *X. saxesenii*, *X. subgranulatus*, *X. thaiphami*.

##### Distribution.

India (Assam).

##### Host plants.

Unknown.

##### Remarks.

Wood (1989) placed *X. subspinosus* in synonymy with *X. saxesenii* without comment. The declivity of the two species are obviously different with that of *X. subspinosus* having declivital interstriae 1 unarmed except at base, and interstriae 2 denticulate throughout its length. In *X. saxesenii*, declivital interstriae 1 is denticulate throughout its length and interstriae 2 unarmed except at base. *Xyleborinus subspinosus* is here removed from synonymy with *X. saxesenii* due to clearly evident declivital differences.

#### Xyleborinus thaiphami sp. nov.

Taxon classificationAnimaliaColeopteraCurculionidae

http://zoobank.org/59795B16-5A61-45F4-AF6B-7678B50ADF30

Fig. 86G, H, L

##### Type material.

**Holotype**, female, Vietnam: Cao Bang, 22°36.402'N, 105°52.397'E, 1601 m, 13.iv.2014, VN17, Cognato, Smith, Pham, ex standing stump (MSUC). ***Paratypes***, female, China: Chongqing Mun., Wu Xi, viii.2015, Wang, J-L, Lv-Jia, Tian-Shang, ex *Pinus armandii* Franch. (RABC, 1); Guizhou, Guiyang, Huaxi, 25.x.2015, You Li, ex trap baited with ipsenol & EtOH (MSUC, 1); Sichuan, Emei Shan, 17.viii.2016, Tian-Shang (RABC, 1); Vietnam: Cao Bang, 22°36.804'N, 105°51.982'E, 1831 m, 17.iv.2014, VN44, Cognato, Smith, Pham, ex fallen tree, 10 cm branch (MSUC, 1; VNMN, 1); as previous except, VN45, ex 5 cm branch (NMNH, 1).

##### Diagnosis.

2.8–2.9 mm long (mean = 2.86 mm; n = 5); 2.9–3.11× as long as wide. This species is distinguished by the declivital face with interstriae 2 armed by granules at declivital summit (1–3 large denticles present in Vietnamese specimens), unarmed on declivital face; declivital interstriae 3 denticles larger than those of interstriae 1, pointed, spine-like; denticles on interstriae 5 large, sharply pointed, spine-like; discal interstriae 1 and 2 unarmed; declivital interstriae 2 flattened; and large size.

This species is nearly identical to *X. attenuatus* and is distinguished by the declivital interstriae 3 denticles which are acutely pointed rather than incurved, and interstriae 5 denticles never down-curved.

##### Similar species.

*Xyleborinus attenuatus*, *X. saxesenii*, *X. subgranulatus*, *X. subspinosus*.

##### Description

**(female).** 2.8–2.9 mm long (mean = 2.86 mm; n = 5); 2.9–3.11× as long as wide. Body light to dark brown. Legs and antennae light brown. ***Head***: epistoma entire, transverse, with a row of hair-like setae. Frons weakly convex to upper level of eyes, alutaceous, subshiny, finely, sparsely punctate, setose; punctures bearing a long, erect hair-like seta. Eyes shallowly emarginate just above antennal insertion, upper part smaller than lower part. Submentum large, distinctly triangular, deeply impressed. Antennal scape regularly thick, shorter than club. Pedicel as wide as scape, shorter than funicle. Funicle 4-segmented, segment 1 shorter than pedicel. Club longer than wide, obliquely truncate, type 1; segment 1 corneous, encircling anterior face; segment 2 narrow, concave, corneous on anterior face only; sutures absent on posterior face. ***Pronotum***: 1.04× as long as wide. In dorsal view long and rounded frontally, type 7, sides parallel in basal 3/4, rounded anteriorly; anterior margin without serrations. In lateral view elongate, disc much longer than anterior slope, type 7, summit prominent, on anterior 1/3. Anterior slope with densely spaced narrow asperities, becoming lower and more strongly transverse towards summit, bearing long, fine, semi-recumbent, hair-like setae. Disc subshiny, alutaceous, finely punctate, finely setose, setae short, erect, hair-like, some longer hair-like setae at margins. Lateral margins obliquely costate. Base transverse, posterior angles acutely rounded. ***Elytra***: 1.8× as long as wide, 1.73× as long as pronotum. Scutellum minute, conical, disconnected from elytra, surrounded by dense mycangial tuft of setae. Elytral base transverse, medially emarginate near scutellum and mycangial tuft, edge oblique, humeral angles rounded, parallel-sided in basal 3/4, then weakly rounded to apex. Disc occupying basal 2/3, smooth, shiny, unarmed; striae not impressed, glabrous, with moderate punctures separated by two diameters of a puncture; interstriae flat, sparsely finely uniseriate punctate, punctures 1/3 those of striae, each bearing erect hair-like setae, setae approximately as long as width of interstriae 2. Declivital face strongly shagreened, steeply rounded, three striae present, striae parallel, strial punctures as large as on disc, glabrous; interstriae impunctate, setose, setae uniseriate and as long as the width between suture and interstriae 3; interstriae 1 weakly convex, widened from base to declivital midpoint, then narrowed to apex, basal 1/2 armed with three small denticles; interstriae 2 flat, parallel, armed by granules only at declivital summit (1–3 denticles present in some specimens), unarmed on declivital face; interstriae 3 with a row of four large denticles along its length, their apices acutely pointed, spine-like. Posterolateral margin rounded, denticulate from interstriae 3–5. ***Legs***: procoxae contiguous; prosternal coxal piece slightly inflated, conical. Protibiae obliquely triangular, broadest at apical 1/3; posterior face smooth; apical 1/3 of outer margin with six moderate socketed denticles, their length approximately as long as basal width. Meso- and metatibiae flattened; outer margin evenly rounded with ten and nine moderate socketed denticles, respectively.

##### Etymology.

Named after our collaborator Dr. Thai Hong Pham who first saw the standing dead tree in which the holotype was living and who then sawed the tree down with great enthusiasm. Noun in genitive.

##### Distribution.

China (Chongqing, Guizhou, Sichuan), Vietnam.

##### Host plants.

This species is only known from *Pinus armandii* (Pinaceae).

#### Xyleborinus tritus sp. nov.

Taxon classificationAnimaliaColeopteraCurculionidae

http://zoobank.org/0A7C2D75-7120-4A5E-AFFF-BF2F7AD4DFD7

Fig. 87

##### Type material.

***Holotype***: female, Vietnam: Cao Bang, 22°36.804'N, 105°51.982'E, 1831 m, 17.iv.2014, VN46, Cognato, Smith, Pham, ex punky bark (MSUC). ***Paratypes***, female, as holotype except: 22°34.5'N, 105°52.4'E, 1080 m, 14.iv.2014, VN46, ex 8 mm twig (VNMN, 1); Lao Cai, Hoang Lien N.P., 22.35, 103.77, 1500–2000 m, 19.v.2019, VN184, S.M. Smith, A.I. Cognato, ex 6 cm trunk (MSUC, 3; NHMUK, 1); as previous except: VN191, S.M. Smith, A.I. Cognato, ex branches 1 and 7 cm (MSUC, 2; NMNH, 1).

##### Diagnosis.

2.2–2.5 mm long (mean = 2.33 mm; n = 4); 3.0–3.14× as long as wide. This species is distinguished by the declivital face with interstriae 2 unarmed by tubercles, granules present on apical 1/3 of disc; odd numbered declivital interstriae strongly convex, bases of denticles tumescent; and striae 1 nearly convergent with striae 2 on declivital face, interstriae 2 not apparent.

##### Similar species.

*Xyleborinus exiguus*, *X. huifenyinae*, *X. perpusillus*.

##### Description

**(female).** 2.2–2.5 mm long (mean = 2.33 mm; n = 4); 3.0–3.14× as long as wide. Body light to dark brown. Legs and antennae light brown. ***Head***: epistoma entire, transverse, with a row of hair-like setae. Frons weakly convex to upper level of eyes, alutaceous, subshiny, finely, sparsely punctate, setose; punctures bearing a long, erect hair-like seta. Eyes deeply emarginate just above antennal insertion, upper part smaller than lower part. Submentum large, distinctly triangular, deeply impressed. Antennal scape regularly thick, as long as club. Pedicel as wide as scape, shorter than funicle. Funicle 4-segmented, segment 1 shorter than pedicel. Club longer than wide, obliquely truncate, type 1; segment 1 corneous, encircling anterior face; segment 2 narrow, concave, corneous on anterior face only; sutures absent on posterior face. ***Pronotum***: 1.1× as long as wide. In dorsal view very elongate, rounded frontally, type 9, sides parallel on basal 3/4; anterior margin without serrations. In lateral view elongate with disc much longer than anterior slope, type 8, disc flat, summit prominent, at apical 1/3. Anterior slope with densely spaced narrow asperities, becoming lower and more strongly transverse towards summit, bearing long, fine, semi-recumbent, hair-like setae. Disc subshiny, alutaceous, finely punctate, finely setose, setae short, erect, hair-like, some longer hair-like setae at margins. Lateral margins obliquely costate. Base transverse, posterior angles acutely rounded. ***Elytra***: 2.1× as long as wide, 1.9× as long as pronotum. Scutellum minute, conical, disconnected from elytra, surrounded by dense mycangial tuft of setae. Elytral base transverse, medially emarginate near scutellum and mycangial tuft, edge oblique, humeral angles angulate, parallel-sided in basal 3/4, then broadly rounded to apex. Disc occupying basal 2/3, smooth, shiny, glabrous; striae not impressed, with small punctures separated by two diameters of a puncture; interstriae flat, sparsely finely uniseriate punctate, punctures 1/3 those of striae; interstriae 2 variably granulate on apical 1/3 of disc (1–3 granules present). Declivital face strongly shagreened, steeply rounded, three striae present, striae 1 and 3 convergent, interstriae 2 not apparent, strial punctures larger and shallower than on disc, glabrous; interstriae impunctate, setose, setae uniseriate and as long or longer than the width between suture and interstriae 3; odd numbered interstriae strongly convex, and denticulate, bases of tubercles tumescent; even numbered interstriae impressed; interstriae 2 impressed, unarmed along its length (granules on apical 1/3 of disc in one of two specimens examined). Posterolateral margin costate, granulate to interstriae 5. ***Legs***: procoxae contiguous; prosternal coxal piece tall and pointed. Protibiae obliquely triangular, broadest at apical 1/3; posterior face smooth; apical 1/3 of outer margin with six moderate socketed denticles, their length approximately as long as basal width. Meso- and metatibiae flattened; outer margin evenly rounded with eight and nine moderate socketed denticles, respectively.

##### Etymology.

L. *tritus* = commonplace. Named in reference to the uninteresting pattern of granules on the declivity. An adjective.

##### Distribution.

Vietnam.

##### Host plants.

Unknown.

**Figure 87.**
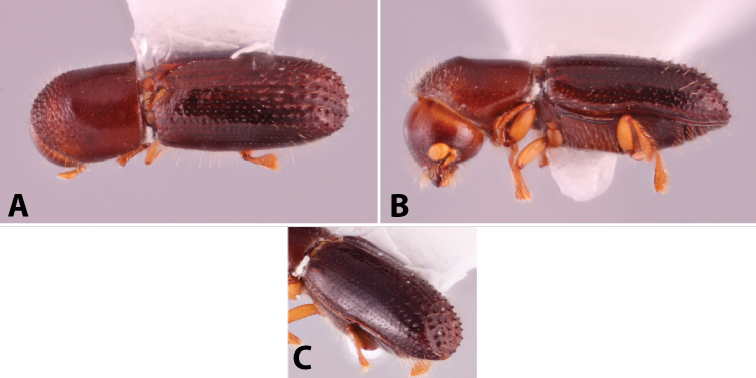
Dorsal, lateral and declivital view of *Xyleborinus tritus* holotype, 2.2 mm (**A–C**).

### *Xyleborus* Eichhoff, 1864

#### Xyleborus

Taxon classificationAnimaliaColeopteraCurculionidae

Eichhoff, 1864

Xyleborus Eichhoff, 1864: 37.
Anaeretus Dugès, 1888: 141. Synonymy: Hagedorn 1910b: 98.
Progenius Blandford, 1896a: 20. Synonymy: Hagedorn 1910b: 98.
Mesoscolytus Broun, 1904: 125. Synonymy: Beaver 1998: 181.
Boroxylon Hopkins, 1915a: 58. Synonymy: Schedl 1952c: 162.

##### Type species.

*Bostrichus monographus* Fabricius, 1793; subsequent designation: Lacordaire, 1865: 381.

##### Diagnosis.

1.9–3.9 mm, 2.5–3.51× as long as wide. *Xyleborus* is distinguished by a combination of homoplastic characters which include the scutellum flush with elytra and flat; mycangial tufts are absent; lateral margin of pronotum obliquely costate; procoxae contiguous; pronotum from dorsal view rounded frontally (types 0, 2, 6, 7), rarely quadrate (type 4 in *X. bidentatus*); elytral disc longer than declivity; elytral disc strial and interstrial punctures seriate; pronotal disc alutaceous; posterior face of the protibiae flat, unarmed; antennal club typically obliquely truncate with segment 1 nearly covering the entire posterior face (type 2), or flattened (type 3); antennal funicle 4-segmented; and anterior margin of pronotum typically unarmed (serrations on a carina in *X. bidentatus*).

##### Similar genera.

*Cryptoxyleborus*, *Fortiborus*, *Heteroborips*, *Planiculus*, *Stictodex*.

##### Distribution.

Widespread throughout temperate and tropical regions of the world.

##### Gallery system.

The gallery system usually consists of irregularly branched tunnels, usually in one horizontal plane, but sometimes spreading into three dimensions, and without brood chambers. However, given the heterogeneity of the genus, it is not surprising that there are variations on this pattern. In some species, small brood chambers may be present.

##### Remarks.

*Xyleborus* is in need of further taxonomic/phylogenetic investigation given its likely polyphyly (Cognato et al. 2020b).

#### Key to *Xyleborus* species (females only)

**Table:** 

1	Antennal club flattened, type 3 (Fig. 3)	**2**
–	Antennal club obliquely truncate, type 2 (Fig. 2)	**3**
2	Elytral apex acuminate; pronotum quadrate (type 4) when viewed dorsally, anterior margin conspicuously extended anteriad with prominent serrations; declivital interstriae 2 with a large spine; larger, 3.4–3.5 mm	*** bidentatus ***
–	Elytral apex broadly rounded; pronotum basic (type 0) when viewed dorsally, anterior margin without a row of serrations; declivital interstriae 2 and 3 equally tuberculate; smaller, 1.9–2.4 mm	*** singhi ***
3	Protibiae semi-circular with evenly rounded outer edge; elytral disc with confused interstrial punctures; antennal club wider than long; eyes deeply emarginated	**4**
–	Protibiae obliquely triangular or triangular; elytral disc with seriate punctures; antennal club circular or longer than wide; eyes moderately emarginated	**5**
4	Declivity lightly shagreened, strial punctures large, deep and distinct; discal interstrial setae uniseriate; larger, 3.0–3.1 mm, less elongate, 2.72–2.82× as long as wide	*** muticus ***
–	Declivity strongly shagreened, strial punctures large, very shallow, difficult to distinguish; discal interstrial setae biseriate; smaller, 2.7 mm, more elongate, 3.0× as long as wide	***opacus* sp. nov.**
5	Declivital interstriae 1 laterally broadened from base to declivital midpoint and then narrowing towards apex (Fig. 89C, J)	**6**
–	Declivital interstriae 1 parallel to suture along its length (Fig. 88A, I)	**12**
6	Posterolateral margin of declivity acutely carinate (Fig. 89D, J)	**7**
–	Posterolateral margin of declivity costate and often granulate (Fig. 89B, I)	**9**
7	Larger, 2.7–2.8 mm; posterolateral margin of declivity carinate to interstriae 6; all declivital striae distinctly impressed.	*** insidiosus ***
–	Smaller, 2.2–2.5 mm; posterolateral margin of declivity carinate to interstriae 7; declivital striae not impressed or striae feebly impressed	**8**
8	Declivital striae and interstriae clearly distinguishable; discal strial punctures 4–5× the diameter of those of interstriae; declivital interstriae 1 without denticles on low tumescences giving the declivity a finely sculptured appearance	*** glabratus ***
–	Declivital striae and interstriae difficult to distinguish; discal strial punctures 3× larger than interstrial punctures; declivital interstriae 1 bearing denticles on low tumescences giving the declivity a rugged sculptured appearance	*** mysticulus ***
9	Posterolateral margin of declivity costate and unarmed	**10**
–	Posterolateral margin of declivity costate and armed with a row of small spines or spinose granules	**11**
10	Declivital interstriae 1 armed by 3–7 large denticles, interstriae 2 armed with denticles or unarmed (highly variable), declivital interstriae 3 armed by 4–9 large denticles, denticles on interstriae 1 and 3 uniform in height; declivital strial punctures moderately sized, fine, uniseriate, never confused; larger, 3.6–3.9 mm	*** festivus ***
–	Declivital interstriae 1 armed by two or three large denticles, interstriae 2 unarmed, declivital interstriae 3 armed by two or three large denticles, denticles on interstriae 3 taller than those on interstriae 1; declivital strial punctures large, shallow, coarse and confused near large tubercles; smaller, 2.9–3.2 mm	*** pfeilii ***
11	Antennal club longer than wide; posterolateral margin of declivity costate and with a row of small spines to interstriae 6; all declivital interstriae with denticles only, lacking granules	***yunnanensis* sp. nov.**
–	Antennal club circular; posterolateral margin of declivity costate and bearing a row of spinose granules to interstriae 7; all declivital interstriae with small spines or granules	***sunisae* sp. nov.**
12	Declivital interstriae 2 unarmed along its entire length; declivity with a pair of prominent tubercles on interstriae 3; interstriae 1 armed only by a denticle at declivital summit	*** ferrugineus ***
–	Declivital interstriae 2 granulate at declivital summit or along entire length; declivity never with a pair of prominent tubercles on interstriae 3, uniformly granulate, with two or three pairs of moderate to large tubercles on interstriae 1 and 3; interstriae 1 armed by sparse tubercles along its entire length	**13**
13	Declivital interstriae 2 sparsely granulate along its entire length	*** volvulus ***
–	Declivital interstriae 2 sparsely granulate at declivital summit only	**14**
14	Declivital interstriae 1 and 3 armed with sparse uniformly sized small granules; declivity shagreened, dull (specimen must be dry)	*** affinis ***
–	Declivital interstriae 1 and 3 armed with two or three pairs of large tubercles; declivity smooth, shiny (specimen must be dry)	**15**
15	Larger, 2.8–3.1 mm and more elongate, 2.8–3.1× as long as wide; declivital interstriae 1 and 3 armed with two or three pairs of large tubercles; elytra typically bicolored	*** cognatus ***
–	Smaller, 2.3–2.6 mm and less elongate, 2.67–2.89× as long as wide; declivital interstriae 1 and 3 armed with two or three pairs of moderate tubercles; elytra typically unicolored	*** perforans ***

#### Xyleborus affinis

Taxon classificationAnimaliaColeopteraCurculionidae

Eichhoff, 1868

Fig. 88A, B, I

Xyleborus affinis Eichhoff, 1868b: 401.
Xyleborus affinis fuscobrunneus Eichhoff, 1878b: 372. Synonymy: Schedl 1959: 504.
Xyleborus affinis mascarensis Eichhoff, 1878b: 372. Synonymy: Wood 1960: 71.
Xyleborus affinis parvus Eichhoff, 1878b: 372. Synonymy: Wood 1960: 71.
Xyleborus sacchari Hopkins, 1915a: 64. Synonymy: Wood 1982: 830.
Xyleborus subaffinis Eggers, 1933a: 36. Synonymy: Schedl 1959: 504.
Xyleborus societatis Beeson, 1935a: 120. Synonymy: Beaver 1991: 94.
Xyleborus proximus Eggers, 1943: 66. Synonymy: Schedl 1963a: 331.

##### Type material.

***Holotype****Xyleborus sacchari* (NMNH). ***Holotype****Xyleborus societatis* (BPBM).

##### New records.

Cambodia: Kampong Speu, Aoral Wildlife Sanctuary, 11°42'10.75"N, 103°52'54.9"E, 200 m, dry dipterocarp forest, 16.xi.2013, O. Košulíc (MNHP, 1). China: Hainan, Changjiang, Bawangling Natl For. Park, 19.117N, 109.080E, 119 m, 5.xii.2016, Tian-Shang, Lv-Jia (RABC, 1). S Yunnan, Xishuangbanna, 20 km NW Jinghong, vic. Man Dian (NNNR), 22°07.80'N, 100°40.0'E, 740 m, rubber plantation, 23.v.2008, A. Weigel (NKME, 1); as previous except: 23 km NW Jinghong, vic. Na Ban (NNNR), 22°09.49'N, 100°39.92'E, transit zone, 730 m, 15.vi.2008, A. Weigel (NKME, 1); as previous except: forest, EKL, 26.iii.2009, L. Meng (RABC, 1); as previous except: 28 km NW Jinghong, vic. An Ma Xi Zhan (NNNR), 22°12'N, 100°38'E, 700 m, forest, EKL, 30.x.2008, A. Weigel (RABC, 1). India: Meghalaya, Nokrek N. P., 3 km S Darbokgiri, 25°27'N, 90°19'E, 1400 m, 26.iv.1999, Dembický, Pacholátko (RABC, 1). Tamil Nadu, Pondicherry, 10 km N. Auroville, 2.ii–2.iii.2011, F. Burger (NKME, 1). Laos: Vientiane, Ban Van Eue, 15.ii.1966, native collector (BPBM, 2); as previous except: 15.v.1966 (BPBM, 1). Myanmar: Yangon Division, Highland Lodge, 16°51.29'N, 96°08.29'E, 11.v.1998, J. Slovinsky, ex uv light trap in semi-tropical urban rainforest (CASC, 1). Vietnam: Dong Nai, Cat Tien N.P., 11.43771, 107.42253, 142 m, 21.ii.2017, VN84, A.I. Cognato, T.A. Hoang, ex 6–15 cm diameter branches (MSUC, 3). Lao Cai, Hoang Lien N.P., 22.35, 103.77, 1500 m, 21.v.2019, VN152, S.M. Smith, A.I. Cognato, ex FIT (MSUC, 4). Ninh Binh, Doi Vac, Cuc Phuong, 10–16.ix.2013, J.B. Heppner (FSCA, 2).

##### Diagnosis.

2.2–2.5 mm long (mean = 2.32 mm; n = 5); 2.56–3.14× as long as wide. This species is distinguished by the protibiae obliquely triangular, broadest at distal 1/3; declivity shagreened, dull (specimen must be dry); small size; declivital interstriae 1 and 3 armed with sparse uniformly sized small granules, interstriae 2 sparsely granulate at declivital summit; and declivity not appearing sulcate.

##### Similar species.

*Xyleborus cognatus*, *X. ferrugineus*, *X. festivus*, *X. perforans*, *X. pfeilii*, *X. volvulus*.

##### Distribution.

Probably native to tropical America (Wood 1977; Gohli et al. 2016), but now in temperate and tropical regions around the world. Less common in the Oriental region than in Africa and the Americas, but sometimes locally abundant. Recorded in the study region from Cambodia*, China* (Hainan, Yunnan), India (Meghalaya*, Tamil Nadu*, no state recorded), Laos*, Myanmar*, Nepal, Taiwan, Thailand, Vietnam*.

##### Host plants.

Strongly polyphagous (Schedl 1963a, as *Xyleborus mascarensis* Eichhoff; Wood 1982).

##### Remarks.

The biology of the species is reviewed by Schedl (1963a). Schneider (1987) notes that more than one generation may inhabit the same gallery system, and describes the oral mycangia. Seasonal changes in numbers caught in traps have been related to temperature and rainfall in Africa (Beaver and Löyttyniemi 1991; Madoffe and Bakke 1995), and in Central America (Rangel et al. 2012). Flight height preference in Amazonia is described by Abreu et al. (2001). Laboratory rearing techniques, and the occurrence of delayed dispersal and alloparental care are discussed by Biedermann et al. (2009, 2011). Although its attacks are secondary, the species can be of economic importance due to its abundance and wide host range.

**Figure 88.**
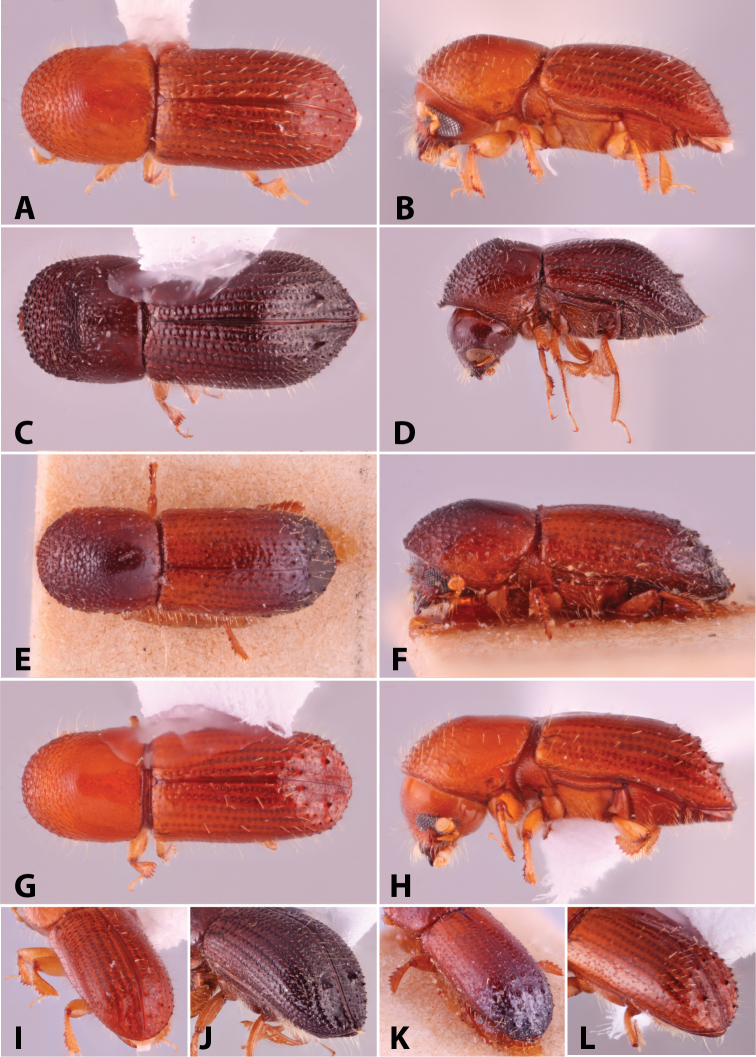
Dorsal, lateral and declivital view of *Xyleborus affinis*, 2.2–2.5 mm (**A, B, I**), *X. bidentatus*, 3.4–3.5 mm (**C, D, J**), *X. cognatus*, 2.8–3.1 mm (**E, F, K**), and *X. ferrugineus*, 2.5–3.1 mm (**G, H, L**).

#### Xyleborus bidentatus

Taxon classificationAnimaliaColeopteraCurculionidae

(Motschulsky, 1863)

Fig. 88C, D, J

Phloeotrogus bidentatus Motschulsky, 1863: 514.
Xyleborus bidentatus (Motschulsky): Eichhoff 1878b: 505.
Xyleborus subcostatus Eichhoff, 1869a: 281. Synonymy: Hulcr and Cognato 2013: 150.
Xyleborus riehlii Eichhoff, 1878b: 346. Synonymy: Schedl 1963a: 282.
Progenius fleutiauxi Blandford, 1896a: 21. Synonymy: Hulcr and Cognato 2013: 150.
Xyleborus laeviusculus Blandford, 1896a: 21. Synonymy: Schedl 1960b: 108.
Boroxylon stephegynis Hopkins, 1915a: 58. Synonymy: Wood 1960: 54.
Boroxylon webbi Hopkins, 1915a: 59. Synonymy: Hulcr and Cognato 2013: 150.
Xyleborus subcostatus dearmatus Eggers, 1923: 205. Synonymy: Hulcr and Cognato 2013: 150.
Xyleborus brevidentatus Eggers, 1930: 190. Synonymy: Schedl 1960b: 107.
Xyleborus quadridens Eggers, 1930: 191. Synonymy: Wood 1989: 176.

##### Type material.

***Holotype****Boroxylon stephegynis* (NMNH). ***Holotype****Boroxylon webbi* (NMNH). ***Holotype****Xyleborus brevidentatus* (FRI), ***paratype*** (NMNH). ***Holotype****Xyleborus quadridens* (FRI).

##### Diagnosis.

3.4–3.5 mm long (mean = 3.48 mm; n = 5); 2.5–2.69× as long as wide. This species is distinguished by the acuminate elytral apex; elytra broadest at apical 1/3; declivity gently sloped, almost concave near apex; protibiae slender, abruptly broadened and triangular on distal 1/3, apical mucro very large, prominent; pronotum quadrate (type 4) when viewed dorsally, anterior margin conspicuously extended anteriad with prominent serrations; pronotum strongly asperate on apical 1/2, disc weakly serrate; declivital interstriae 2 with a large spine; and large size.

##### Similar species.

*Ambrosiodmus* spp.

##### Distribution.

Australia, ‘Borneo’, India (Andaman Is, Nicobar Is, West Bengal), Indonesia (Java, Sulawesi, Maluku, Sumatra, Sumbawa), East & West Malaysia, Myanmar, New Guinea, Palau, Philippines, Singapore, Taiwan, Thailand, Vietnam. Also recorded from East Africa and Madagascar.

##### Host plants.

Polyphagous (Schedl 1963a).

##### Remarks.

Murphy and Meepol (1990) suggest an association with mangroves in southern Thailand, as do Maiti and Saha (2004) in the Sundarbans and Andaman Islands, but in general the species is polyphagous.

#### Xyleborus cognatus

Taxon classificationAnimaliaColeopteraCurculionidae

Blandford, 1896

Fig. 88E, F, K

Xyleborus cognatus Blandford, 1896a: 19.

##### Type material.

***Syntypes*** (NHMUK).

##### Diagnosis.

2.8–3.1 mm long (mean = 2.96 mm; n = 5); 2.8–3.1× as long as wide. This species is distinguished by the protibiae obliquely triangular, broadest at distal 1/3; declivity smooth, shiny (specimen must be dry); large size; declivital interstriae 1 and 3 armed with two or three pairs of large tubercles; interstriae 2 sparsely granulate at declivital summit; and elytra darker on declivity than disc.

This species is very similar to *X. perforans* with which it has often been treated as a synonym. It is distinguished by the larger size, generally more slender form (vs. 2.67–2.89× as long as wide), larger interstrial tubercles and typically bicolored elytra.

##### Similar species.

*Xyleborus affinis*, *X. ferrugineus*, *X. festivus*, *X. perforans*, *X. pfeilii*, *X. volvulus*.

##### Distribution.

Australia, India (Andaman Is, Bihar, Uttarakhand, West Bengal), Indonesia (Java, Kalimantan, Sulawesi, Maluku, Sumatra, Sumbawa), East & West Malaysia, Myanmar, New Caledonia, New Guinea, Philippines, Singapore, Solomon Islands, Sri Lanka, Thailand, Vietnam.

##### Host plants.

Polyphagous (Beeson 1930; Browne 1961b; Ohno 1990).

##### Remarks.

The species is frequently associated with mangrove forests, but also attacks a very wide variety of other trees (Browne 1961b; Maiti and Saha 2004).

#### Xyleborus ferrugineus

Taxon classificationAnimaliaColeopteraCurculionidae

(Fabricius, 1801)

Fig. 88G, H, L

Bostrichus ferrugineus Fabricius, 1801: 388.
Xyleborus ferrugineus (Fabricius): Ferrari 1867: 23.
Tomicus trypanaeoides Wollaston, 1867: 114. Synonymy: Browne 1955: 355; Schedl, 1960a: 9.
Xyleborus fuscatus Eichhoff, 1868a: 400. Synonymy: Schedl 1960a: 8.
Xyleborus confusus Eichhoff, 1868a: 401. Synonymy: Schedl 1957: 16.
Xyleborus retusicollis Zimmermann, 1868: 146. Synonymy: Bright 1968: 1312.
Xyleborus amplicollis Eichhoff, 1869: 280. Synonymy: Schedl 1960a: 8.
Xyleborus insularis Sharp, 1885: 193. Synonymy: Schedl 1941: 116.
Xyleborus tanganus Hagedorn, 1910a: 8. Synonymy: Schedl 1960a: 8.
Xyleborus nyssae Hopkins, 1915a: 66. Synonymy: Schedl 1960a: 9.
Xyleborus soltaui Hopkins, 1915a: 66. Synonymy: Bright 1968: 1312.
Xyleborus hopkinsi Beeson, 1929: 246. Synonymy: Schedl 1960a: 8.
Xyleborus argentinensis Schedl, 1931: 345. Synonymy: Schedl 1960a: 8.
Xyleborus rufopiceus Eggers, 1932: 303. Synonymy: Wood 1989: 176.
Xyleborus schedli Eggers, 1934a: 83. Synonymy: Schedl 1960a: 9.
Xyleborus nesianus Beeson, 1940: 200. Synonymy: Beaver 1991: 95.
Xyleborus notatus Eggers, 1941a: 107. Synonymy: Schedl 1960a: 8.
Xyleborus subitus Schedl, 1949: 280. Synonymy: Schedl 1960a: 9.

##### Type material.

***Holotype****Xyleborus hopkinsi* (NHMUK), ***paratype*** (FRI). ***Holotype****Xyleborus nesianus* (BPBM). ***Holotype****Xyleborus notatus* (NMNH). ***Holotype****Xyleborus nyssae* (NMNH). ***Holotype****Xyleborus retusicollis* (MCZ). ***Holotype****Xyleborus schedli* (NMNH). ***Holotype****Xyleborus soltaui* (NMNH).

##### Diagnosis.

2.5–3.1 mm long (mean = 2.84 mm; n = 5); 2.78–3.11× as long as wide. This species is distinguished by the protibiae obliquely triangular, broadest at distal 1/3; declivity smooth, shiny (specimen must be dry); declivity with a pair of prominent tubercles on interstriae 3; declivity distinctly sulcate between suture and interstriae 3; interstriae 1 armed only by a denticle at declivital summit; and interstriae 2 unarmed.

##### Similar species.

*Xyleborus affinis*, *X. cognatus*, *X. festivus*, *X. perforans*, *X. pfeilii*, *X. volvulus*.

##### Distribution.

Probably native to tropical America (Wood 1977; Gohli et al. 2016), but now in temperate and tropical regions around the world. Not common in the Oriental region, but more widely present than indicated by Wood and Bright (1992). Recorded in the study region only from India (West Bengal), and Taiwan.

##### Host plants.

Strongly polyphagous, with several hundred hosts recorded (Schedl 1963a; Ohno 1990; Ohno et al. 1988, 1989).

##### Remarks.

The biology of the species is described by Schedl (1963a) and Entwhistle (1972). Norris (1976) summarizes studies by his group on the role of the associated ambrosia fungi in the nutrition and development of the beetle, the requirement of a fungal-produced steroid for pupation, and of associated bacteria for oocyte maturation. The species has some economic importance as a pest of cocoa (*Theobroma cacao*) (Malvaceae) as a vector of cocoa wilt (Entwhistle 1972). Wood (2007) considers it one of the most destructive species of harvested timber in South America. Measurements were taken from Atkinson et al. (2013). We were unable to measure Asian specimens. Measurements were of New World specimens from Guyana, Panama, Peru and the United States (Florida and Michigan) in MSUC.

#### Xyleborus festivus

Taxon classificationAnimaliaColeopteraCurculionidae

Eichhoff, 1876

Fig. 89A, B, I

Xyleborus festivus Eichhoff, 1876a: 202.
Xyleborus pinicola Eggers, 1930: 206. Synonymy: Smith et al. 2018b: 397.
Xyleborus detectus Schedl, 1975a: 458. Synonymy: Smith et al. 2018b: 397.
Xyleborus pinivorus Browne, 1980a: 374. Synonymy: Smith et al. 2018b: 397.

##### Type material.

***Holotype****Xyleborus festivus* (UHZM). ***Holotype****Xyleborus detectus* (NHMW). ***Holotype****Xyleborus pinicola* (FRI), ***paratypes*** (NHMW, 1; NMNH, 2). ***Holotype****Xyleborus pinivorus* (NHMUK).

##### New records.

China: Fujian, Nanjing, Zhangzhou, 600 m, 3.iii.1962, Fusheng Huang, ex *Pinus massoniana* (NMNH, 5). Guizhou, Guiyang, Huaxi, 6.xi.2015, Y. Li, ex *Pinus massoniana* (UFFE, 1). Yunnan, sawmill near Ning’er, 19.vi.2010, Zhou, X-D, *Pinus kesiya* sawmill log (RABC, 1). Taiwan: Huisin Forest, 23.ix.2015, A. Black, J. Skelton, ex *Pinus taiwanensis* (UFFE, 1).

##### Diagnosis.

3.6–3.9 mm long (mean = 3.75 mm; n = 5); 2.85–3.17× as long as wide. This species is distinguished by the declivity steep, appearing convex from lateral view; declivital interstriae 1–3 laterally broadened from base to declivital midpoint then narrowing towards apex; large body size; declivital posterolateral margin costate extending to interstriae 7; declivital interstriae 1 and 3 convex, interstriae 2 impressed; declivital striae feebly impressed; declivital interstriae 1 armed by 3–7 large denticles, interstriae 2 armed with denticles or unarmed (highly variable), declivital interstriae 3 armed by 4–9 large denticles, denticles on interstriae 1 and 3 uniform in height; declivital strial punctures moderately sized, fine, uniseriate, never confused.

##### Similar species.

*Xyleborus affinis*, *X. cognatus*, *X. ferrugineus*, *X. perforans*, *X. pfeilii*, *X. volvulus*.

##### Distribution.

China (Fujian, Guangdong, Guangxi, Guizhou, Yunnan), Japan, Myanmar, Taiwan, Thailand, Vietnam.

##### Host plants.

This species is unusual amongst *Xyleborus* in attacking only species of *Pinus* (Pinaceae) (Wood and Bright 1992) including *P. kesiya*, *P. massoniana*, *P. yunnanensis* and *P. taiwanensis* (Li et al. 2020).

##### Remarks.

The Chinese host range and fungal associates of this species were recently reported (Li et al. 2020)

**Figure 89.**
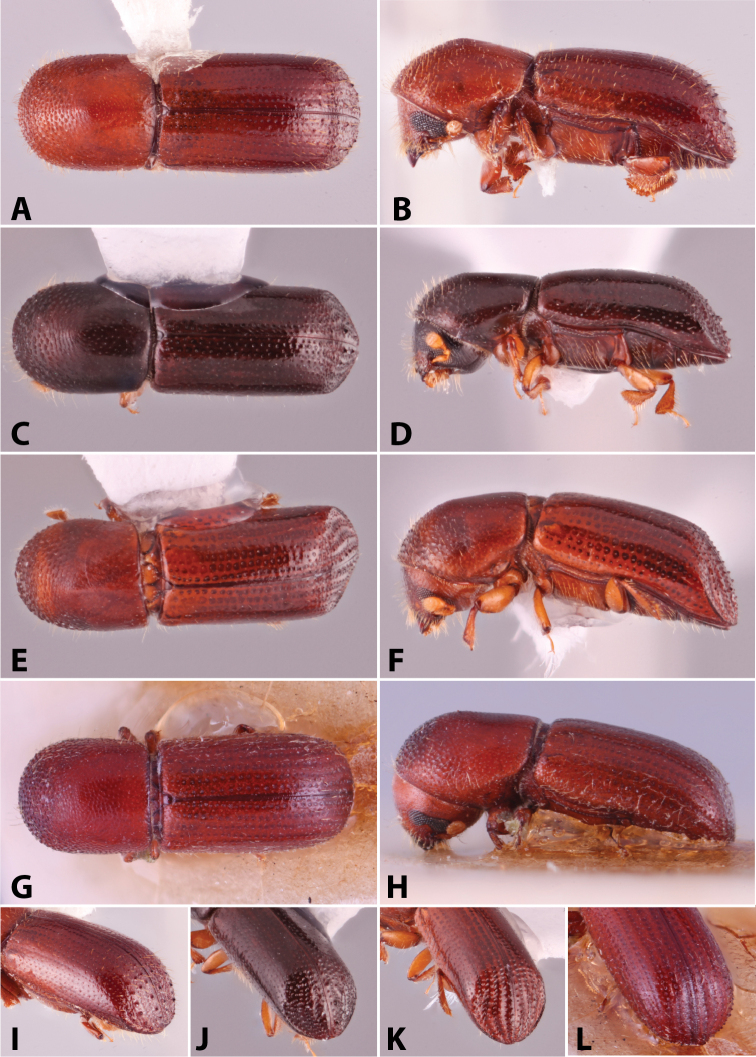
Dorsal, lateral and declivital view of *Xyleborus festivus*, 3.6–3.9 mm (**A, B, I**), *X. glabratus*, 2.2–2.5 mm (**C, D, J**), *X. insidiosus* holotype (**E, F, K**), and *X. muticus* holotype, 3.0–3.1 mm (**G, H, L**).

#### Xyleborus glabratus

Taxon classificationAnimaliaColeopteraCurculionidae

Eichhoff, 1877

Fig. 89C, D, J

Xyleborus glabratus Eichhoff, 1877: 127.
Xyleborus kumamotoensis Murayama, 1934: 288. Cognato et al. 2019: 1276.

##### Type material.

***Lectotype****Xyleborus glabratus* (MIIZ). ***Lectotype****Xyleborus kumamotoensis* (NMNH).

##### Diagnosis.

2.2–2.5 mm long (mean = 2.36 mm; n = 5); 3.14–3.57× as long as wide. This species is distinguished by declivital interstriae 1 laterally broadened from base to declivital midpoint and then narrowing towards apex; anterior 1/2 of the pronotum strongly shiny; discal interstriae 2× the width of striae; discal strial punctures 4–5× the diameter of those of interstriae; declivital striae and interstriae clearly distinguishable; declivital striae flat to feebly impressed; declivital interstriae 1 with at least one large denticle (typically three), numerous closely spaced granules and 1–3 small denticles (typically one); and posterolateral margin of declivity carinate to interstriae 7.

##### Similar species.

*Xyleborus insidiosus*, *X. mysticulus*.

##### Distribution.

Bangladesh, China (Fujian, Guangdong, Guangxi, Hong Kong, Hunan, Jiangxi, Sichuan), India (Assam, West Bengal), Japan, Myanmar, South Korea, Taiwan, Thailand, Vietnam. Imported to and established in USA (Rabaglia et al. 2006; Gomez et al. 2018a).

##### Host plants.

The species has an evident preference for the family Lauraceae, and its attacks are restricted to that family in the US (Rabaglia et al. 2006; Fraedrich et al. 2008). In the Oriental region, it has also been recorded on a few occasions from other families (Dipterocarpaceae, Fabaceae, Fagaceae, Pinaceae, Theaceae) (Beaver and Liu 2010; Hulcr and Lou 2013), but it is not clear whether it was breeding in these trees.

##### Remarks.

Although not of economic importance in its native range, the species is an invasive pest in the US, where it transmits a pathogenic fungus (*Raffaelea lauricola*) to a variety of Lauraceae trees (including avocado) (Harrington et al. 2011). Consequently, its host preferences, attractant volatiles, flight activity and other aspects of its biology, and possible management and control methods, have recently been studied intensively (e.g., Hanula et al. 2008; Hulcr et al. 2011; Brar et al. 2012, 2013; Kendra et al. 2012, 2015, 2016; Formby et al. 2013; Maner et al. 2013; Mayfield et al. 2013; Peña et al. 2015). Recent field collections in its native range revealed that the species exhibits the same biology there as it does in the US (Hulcr et al. 2017; Cognato et al. 2019).

#### Xyleborus insidiosus

Taxon classificationAnimaliaColeopteraCurculionidae

Cognato & Smith, 2019

Fig. 89E, F, K

Xyleborus insidiosus Cognato & Smith, 2019, (in Cognato et al. 2019): 1280.

##### Type material.

***Holotype*** (MSUC), ***paratypes*** (IZAS, 1; MSUC, 4; NHMUK, 1; NMNH, 3).

##### Diagnosis.

2.7–2.8 mm long (mean = 2.74 mm; n = 5); 3.0–3.5× as long as wide. This species is distinguished by declivital interstriae 1 laterally broadened from base to declivital midpoint then narrowing towards apex; large body size; broad discal interstriae, 4× the width of discal striae; discal strial punctures 3× the diameter of those of interstriae; declivital striae and interstriae clearly distinguishable, striae clearly impressed; interstriae uniformly granulate, never denticulate; anterior 1/2 of pronotum strongly shagreened; and declivital posterolateral margin carinate to interstriae 6.

##### Similar species.

*Xyleborus glabratus*, *X. mysticulus*.

##### Distribution.

China (Sichuan), Vietnam.

##### Host plants.

This species has been collected from Fagaceae as well as unidentified punky wood (Cognato et al. 2019).

#### Xyleborus muticus

Taxon classificationAnimaliaColeopteraCurculionidae

Blandford, 1894

Fig. 89G, H, L

Xyleborus muticus Blandford, 1894b: 112.
Xyleborus lignographus Schedl, 1953c: 28. syn. nov.
Xyleborus conditus Schedl, 1971b: 379. syn. nov.

##### Type material.

***Holotype****Xyleborus muticus* (NHMUK), ***paratype*** (NHMUK). ***Holotype****Xyleborus conditus* (NHMW). ***Lectotype****Xyleborus lignographus* (NHMW).

##### Diagnosis.

3.0–3.1 mm long (mean = 3.08 mm; n = 4); 2.72–2.82× as long as wide. This species is distinguished by the antennal club distinctly wider than long; protibiae with evenly rounded outer edge; elytral posterolateral costa absent, replaced by a short row of tubercles; declivity lightly shagreened, strial punctures large, deep and distinct; and discal interstrial setae uniseriate.

##### Similar species.

*Xyleborus sunisae*.

##### Distribution.

China (Fujian, Sichuan), India (Uttar Pradesh), Japan (Honshu, Kyushu), Nepal, South Korea, Vietnam.

##### Host plants.

This species has been recorded from *Quercus* (Fagaceae) and *Prunus* (Rosaceae) (Murayama 1954).

##### Remarks.

The holotypes of *X. muticus* and *X. conditus* and the lectotype of *X. lignographus* were directly compared. All three specimens were found to be conspecific with minor variations observed in the numbers of setae remaining on the specimens and numbers of granules on the declivital interstriae. *Xyleborus conditus* and *X. lignographus* are here placed in synonymy with *X. muticus*.

Wood and Bright (1992) erroneously reported this species from ‘*Pinus maximowiczii*’. Murayama (1954) reported the species from *Prunus maximowiczii*, Korean cherry, thus the record from *Pinus* is incorrect.

#### Xyleborus mysticulus

Taxon classificationAnimaliaColeopteraCurculionidae

Cognato & Smith, 2019

Fig. 90A, B, I

Xyleborus mysticulus Cognato & Smith, 2019 (in Cognato et al. 2019): 1281.

##### Type material.

***Holotype*** (MSUC), ***paratypes*** (MSUC, 9; NHMUK, 2; NMNH, 2).

##### New records.

Vietnam: Lao Cai, Nam Tha, 22.01218, 104.37685, 28.v.2015, Pham Thu, ex funnel trap (RJRC, 1); as previous except: Hoang Lien N.P., 22.35N, 103.77E, 1500 m, 21.v.2019, VN152, S.M. Smith, A.I. Cognato, ex FIT (MSUC, 1).

##### Diagnosis.

2.2–2.5 mm long (mean = 2.38 mm; n = 5); 3.14–3.57× as long as wide. This species is distinguished by declivital interstriae 1 laterally broadened from base to declivital midpoint then narrowing towards apex; discal interstriae 2× the width of discal striae; discal strial punctures 3× larger than interstrial punctures; declivital interstriae bear both denticles and granules, denticles on low tumescences giving the declivity a rugged sculptured appearance; declivital striae not impressed; declivital striae and interstriae difficult to distinguish; and declivital posterolateral margin carinate to interstriae 7.

##### Similar species.

*Xyleborus glabratus*, *X. insidiosus*.

##### Distribution.

Taiwan, Vietnam.

##### Host plants.

*Machilus* (Lauraceae) and unidentified Lauraceae (Cognato et al. 2019).

**Figure 90.**
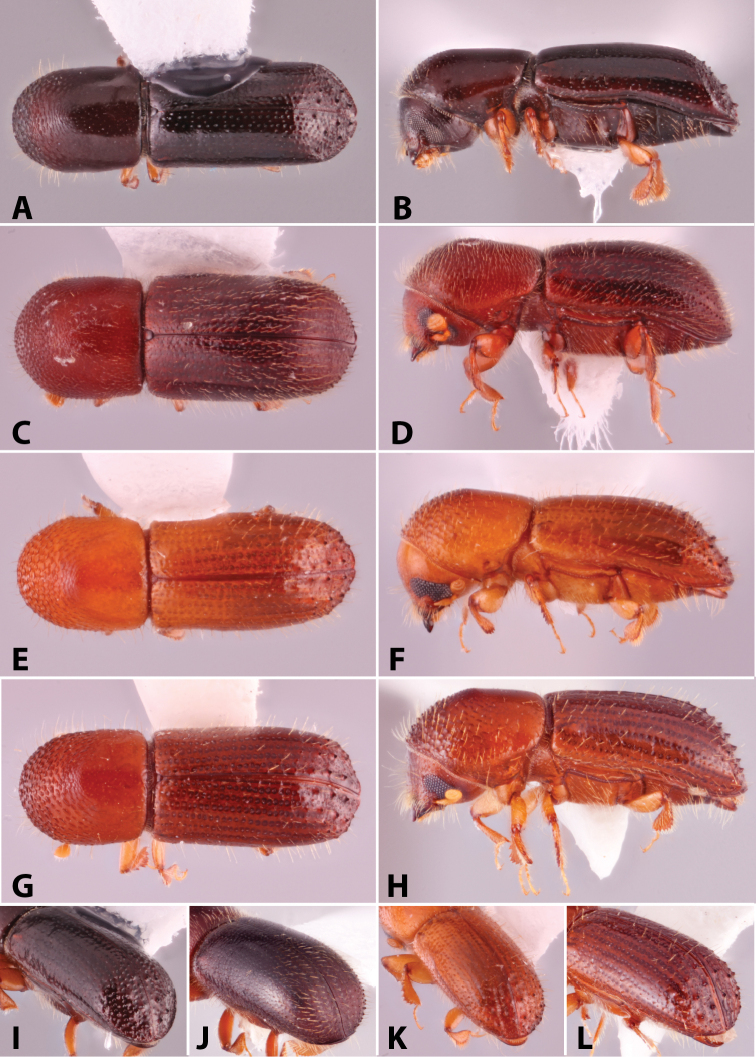
Dorsal, lateral and declivital view of *Xyleborus mysticulus* holotype, 2.2–2.5 mm (**A, B, I**), *X. opacus* paratype, 2.7 mm, 2.2–2.5 mm (**C, D, J**), *X. perforans*, 2.3–2.6 mm (**E, F, K**), and *X. pfeilii*, 2.9–3.2 mm (**G, H, L**).

#### Xyleborus opacus sp. nov.

Taxon classificationAnimaliaColeopteraCurculionidae

http://zoobank.org/300653C8-BA48-4402-8DF7-BF88B7427B30

Fig. 90C, D, J

##### Type material.

***Holotype***, female, Vietnam: Cao Bang, 22°36.454'N, 105°52.083'E, 1661 m, 17.iv.2014, VN40, Cognato, Smith, Pham, ex 3 pieces “firewood” (MSUC). ***Paratypes***, female, as holotype (MSUC, 1); as previous except: 22°36.3'N, 105°52.6'E, 1435–1601 m, 13–17.iv.2014, VN16, Cognato, Smith, Pham, ex FIT (MSUC, 1).

##### Diagnosis.

2.7 mm long (mean = 2.9 mm; n = 3); 3.0× as long as wide. This species is distinguished by the antennal club distinctly wider than long; protibiae with evenly rounded outer edge; elytral posterolateral costa absent, replaced by a short row of tubercles; declivity strongly shagreened, strial punctures large, very shallow, difficult to distinguish; and discal interstrial setae biseriate.

##### Similar species.

*Xyleborus muticus*.

##### Description

**(female).** 2.7 mm long (mean = 2.9 mm; n = 3); 3.0× as long as wide. Body red-brown to dark brown. Legs and antennae light brown. ***Head***: epistoma entire, transverse, with a row of hair-like setae. Frons weakly convex to upper level of eyes; median carina present; surface shagreened, alutaceous, punctate; punctures sparse, shallow, setose, each bearing a long, erect hair-like seta. Eyes moderately emarginate just above antennal insertion, upper part smaller than lower part. Submentum narrow, triangular, deeply impressed. Antennal scape regularly thick, slightly longer than club. Pedicel as wide as scape, as long as funicle. Funicle 4-segmented, segment 1 shorter than pedicel. Club wider than long, obliquely truncate, type 2; segment 1 corneous, transverse on anterior face, occupying basal 2/5 of club, nearly covering posterior face; segment 2 narrow, corneous; segments 1 and 2 present on posterior face. ***Pronotum***: 1.03× as long as wide. In dorsal view long and rounded frontally, type 7, sides parallel in basal 3/4, rounded anteriorly; anterior margin without serrations. In lateral view elongate, disc longer than anterior slope, type 7, summit on anterior 1/3. Anterior slope with densely spaced small asperities, becoming lower and more strongly transverse towards summit. Disc shagreened, alutaceous, with dense, fine punctures bearing long, fine, erect hair-like setae, some longer hair-like setae at margins. Lateral margins obliquely costate. Base transverse, posterior angles broadly rounded. ***Elytra***: 1.5× as long as wide, 1.57× as long as pronotum. Scutellum moderately sized, linguiform, flush with elytra, flat, shiny. Elytral base transverse, edge oblique, humeral angles rounded, parallel-sided in basal 3/4, then broadly rounded to apex. Disc subshiny, striae not impressed, with moderately coarse, shallow punctures separated by 2–3 diameters of a puncture, each puncture bearing a short, recumbent seta slightly longer than puncture diameter; interstriae flat, finely punctate, punctures smaller than those of striae and strongly confused, punctures more widely separated than those of striae bearing two rows of semi-erect long, fine, erect hair-like setae, setae approximately as long as width of interstriae 2. Declivity steeply rounded, strongly shagreened; three striae present, striae parallel, strial punctures much larger than on disc, glabrous; interstriae impunctate, setose, setae uniseriate and similar in size to those of discal interstriae. Posterolateral margin rounded, denticulate from interstriae 4–8. ***Legs***: procoxae contiguous; prosternal coxal piece tall, pointed. Protibiae obliquely triangular, broadest at apical 1/3; posterior face smooth; apical 1/2 of outer margin with eight large socketed denticles, their length longer than basal width. Meso- and metatibiae flattened; outer margins evenly rounded with ten and 12 small socketed denticles, respectively.

##### Etymology.

L. *opacus* = dark. In reference to the species’ habitus. An adjective.

##### Distribution.

Vietnam.

##### Host plants.

Unknown.

#### Xyleborus perforans

Taxon classificationAnimaliaColeopteraCurculionidae

(Wollaston, 1857)

Fig. 90E, F, K

Tomicus perforans Wollaston, 1857: 96.
Xyleborus perforans (Wollaston): Eichhoff 1878b: 403.
Bostrichus testaceus Walker, 1859: 260. Synonymy: Browne 1955: 355.
Xyleborus duponti Montrouzier, 1861: 265. Synonymy: Hagedorn 1910b: 108.
Anodius tuberculatus Motschulsky, 1863: 511. Synonymy: Wood 1969: 117.
Anodius denticulus Motschulsky, 1863: 512. Synonymy: Wood 1969: 117.
Xyleborus kraatzii Eichhoff, 1868b: 152. Synonymy: Schedl 1959: 503.
Xyleborus kraatzii philippinensis Eichhoff, 1878b: 374. Synonymy: Schedl 1959: 503.
Xyleborus immaturus Blackburn, 1885: 193. Synonymy: Beeson 1929: 240.
Xylopertha hirsuta Lea, 1894: 321. Synonymy: Schedl 1936b: 529, 1959: 503.
Xyleborus whitteni Beeson, 1935b: 113. Synonymy: Beaver 1991: 95.
Xyleborus apertus Schedl, 1939a: 355. Synonymy: Bright and Skidmore 1997: 4, 154.
Xyleborus criticus Schedl, 1950b: 899. Synonymy: Wood 1989: 177.
Xyleborus shionomisakiensis Murayama, 1951: 3. Synonymy: Smith et al. 2018b: 398.
Xyleborus cylindrus Schedl, 1951a: 94. Synonymy: Bright and Skidmore 1997: 4, 155.
Xyleborus minimus Schedl, 1955a: 305. Synonymy: Bright and Skidmore 1997: 4, 161.

##### Type material.

***Syntypes****Xyleborus whitteni* (BPBM).

##### New records.

China: Hong Kong, Tai Po Kau, vi.2017, J. Skelton (UFFE, 1). Laos: 10 km N Luang-Prabang, Mekhong river, 240 km N Vientiane, hills c. 250 m, poor settlem[ent], prim[ary] veget[ation] lux, iv.1993, Insomsay Somsy (MFNB, 2); as previous except: iii.1993 (MFNB, 2). Vientiane, Ban Van Eue, 15.xii.1965, native collector (BPBM, 3). Vietnam: NE region, Bac Giang, Tay Yen Tu Nature Res., 10.vi.2016, at light, 21°11.6'N, 106°45.232'E, G.S. Powell (MSUC, 1). [Da Lak], 10 km E of Ban ME Thout [*sic*] [= Buon Ma Thout], 855 m, 20.v.1960, R.E. Leech (BPBM, 1). Dong Nai, Cat Tien N.P., 11.40817, 107.38098, 134 m, 22–24.ii.2017, VN81, A.I. Cognato, T.A. Hoang, ex FIT (MSUC, 40); as previous except: Bien Hoa, 25.ii.1969, C.R. Joyce, ex at light (BPBM, 6); as previous except: 10.ix.1969 (BPBM, 2). Ninh Binh, Doi Vac, Cuc Phuong, 10–16.ix.2013, J.B. Heppner (FSCA, 11). Ha Tay, Ba Vi N.P. (lake lodge), 3–4.vii.2008, 196 m, J. B. Heppner (FSCA, 1). Thua Thien-Hue, Bach Ma N.P., 16.22897, 107.85349, 415 m, 15.ii.2017, VN57, A.I. Cognato, T.A. Hoang, ex 5 cm diameter branch; twig (MSUC, 23). Vinh Phuc, Me Linh Biodiversity Station, Dai Lai Lake, 100 m, 27–29.ix.2013, J.B. Heppner (FSCA, 1).

##### Diagnosis.

2.3–2.6 mm long (mean = 2.46 mm; n = 5); 2.67–2.89× as long as wide. This species is distinguished by the protibiae obliquely triangular, broadest at distal 1/3; declivity smooth, shiny (specimen must be dry); declivital interstriae 1 and 3 armed with two or three pairs of moderate tubercles; interstriae 2 sparsely granulate at declivital summit; and elytra unicolored.

This species is very similar to *X. cognatus* which has often been treated as a synonym of *X. perforans*, and *X. volvulus*. It is distinguished from *X. cognatus* by the smaller size (vs. 2.8–3.1 mm), generally stouter form (vs. 2.8–3.1× as long as wide), smaller interstrial tubercles and unicolored elytra. This species is also almost identical to *X. volvulus* and is distinguished the stouter form (vs. 3.13× as long as wide) and interstriae 2 granules only present at declivity summit (vs. entire length).

##### Similar species.

*Xyleborus affinis*, *X. cognatus*, *X. ferrugineus*, *X. festivus*, *X. pfeilii*, *X. volvulus*.

##### Distribution.

Throughout tropical parts of the Afrotropical, Australian and Oriental regions. Recorded in the study region from Bangladesh, Cambodia, China (Guangxi, Hong Kong*, Shanxi, Yunnan), India (Andaman Is, Assam, Chhattisgarh, Haryana, Jharkhand, Karnataka, Kerala, Madhya Pradesh, Maharashtra, Meghalaya, Nicobar Is, Tamil Nadu, Uttarakhand, Uttar Pradesh, West Bengal), Laos, Myanmar, Nepal, Taiwan, Thailand, Vietnam.

##### Host plants.

Strongly polyphagous (e.g., Browne 1961a; Schedl 1963a; Gray and Wylie 1974; Ohno 1990).

##### Remarks.

The biology has been described by Beeson (1961), Browne (1961a), Schedl (1963a) and Kalshoven (1964). The species sometimes attacks weakened or injured trees, and can be a minor pest (Browne 1968a), but its attacks are usually secondary. Due to its abundance, the species can be important in the downgrade of recently felled timber.

#### Xyleborus pfeilii

Taxon classificationAnimaliaColeopteraCurculionidae

(Ratzeburg, 1837)

Fig. 90G, H, L

Bostrichus pfeilii Ratzeburg, 1837: 168.
Xyleborus pfeilii (Ratzeburg): Eichhoff, 1864: 38.
Bostrichus alni Mulsant & Rey, 1856: 111. Synonymy: Eichhoff 1876b: 378.
Xyleborus vicarius Eichhoff, 1876a: 203. Synonymy: Schedl 1963a: 482.
Xyleborus adumbratus Blandford, 1894b: 115. Synonymy: Schedl 1963a: 482.
Xyleborus septentrionalis Niisima, 1909: 162. Synonymy: Smith et al. 2018b: 398.

##### Type material.

***Lectotype****Xyleborus septentrionalis* (NIAES), ***paralectotype*** (NIAES, 1).

##### New records.

China: Jiangxi, Jinggang Shan Mts., Xiangzhu vill. env., 26°35.5'N, 114°16.0'E, 374 m, rice fields, forested stream valley, M. Fikáček, J. Hájek (MNHP, 2; RABC, 1). India: Assam-Arunachal Pradesh border: Bhalukpong, 27°00'48"N, 92°39'08"E, 150 m, 1–8.v.2012, L. Dembický, FIT (ZFMK, 2); as previous except FIT (flight intercept trap) (ZFMK, 1). Laos: NE, Hua Phan, Ban Saluei, Phou Pan Mt., ~ 20°12'N, 104°01'E, 1300–1900 m, 17–26.v.2009, C. Holzschuh (RABC, 1). Vientiane, Ban Van Eue, 15.xii.1965, native collector (BPBM, 1). Sri Lanka: Monaragala Dist., Buttala, 50 m, 6.vi.1975, S.L. Wood, ex *Anogeissus latifolia* (NMNH, 1); as previous except: collected from log (NMNH, 2).

##### Diagnosis.

2.9–3.2 mm long (mean = 3.02 mm; n = 5); 2.73–3.2× as long as wide. This species is distinguished by the declivity steep, appearing flat when viewed laterally; the declivital interstriae 1–3 laterally broadened from base to declivital midpoint then narrowing towards apex; declivital posterolateral margin costate to interstriae 7; declivital interstriae 1 and 3 flat, interstriae 2 weakly impressed; declivital striae weakly impressed; declivital interstriae 1 armed by two or three large denticles, interstriae 2 unarmed, declivital interstriae 3 armed by two or three large denticles, denticles on interstriae 3 taller than those on interstriae 1; and declivital strial punctures large, shallow, coarse and confused near large tubercles.

##### Similar species.

*Xyleborus affinis*, *X. cognatus*, *X. ferrugineus*, *X. festivus*, *X. perforans*, *X. volvulus*.

##### Distribution.

Recorded in the study region from China (Fujian, Hunan, Jiangxi*, Sichuan, Yunnan), India (Andaman Is, Assam*), Laos*. Also recorded from Japan, South Korea, throughout Europe, and in North Africa and Turkey. Imported to and established in USA and Canada (Vandenberg et al. 2000; Gomez et al. 2018a).

##### Host plants.

Polyphagous (Wood and Bright 1992; Mizuno and Kajimura 2008).

##### Remarks.

Mizuno and Kajimura (2008) provide information on the biology, gallery system and development.

#### Xyleborus singhi

Taxon classificationAnimaliaColeopteraCurculionidae

Park & Smith, 2020

Fig. 91A, B, I

Xyleborus singhi Park & Smith, 2020 (in Park et al. 2020): 222.

##### Type material.

***Paratypes*** (ZFMK, 2).

##### Diagnosis.

1.9–2.4 mm long (mean = 2. 15 mm; n = 2); 2.53–3.0× as long as wide. This species is distinguished by declivital interstriae 1 unarmed; declivital interstriae 2 and 3 equally tuberculate; and protibiae obliquely triangular.

##### Similar species.

None.

##### Distribution.

India (Arunachal Pradesh), South Korea.

##### Host plants.

Unknown.

**Figure 91.**
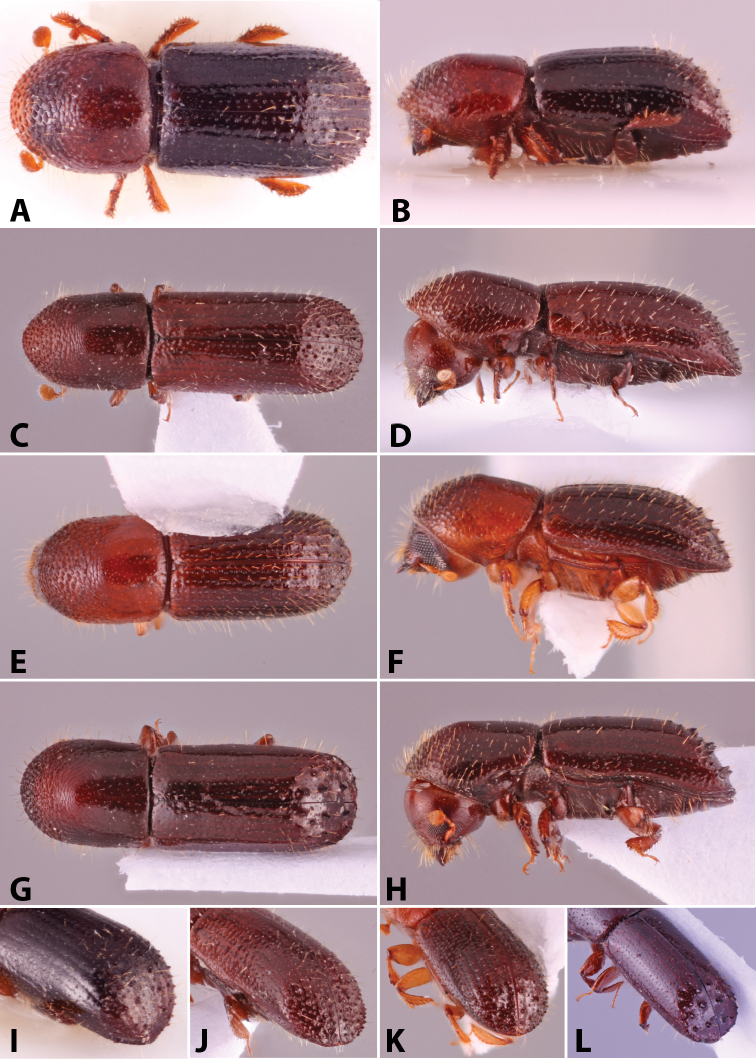
Dorsal, lateral and declivital view of *Xyleborus singhi* paratype, 1.9–2.4 mm (**A, B, I**), *X. sunisae* holotype, 2.7–2.75 mm (**C, D, J**), *X. volvulus*, 2.5 mm (**E, F, K**), and *X. yunnanensis* paratype, 2.7–2.75 mm (**G, H, L**).

#### Xyleborus sunisae sp. nov.

Taxon classificationAnimaliaColeopteraCurculionidae

http://zoobank.org/DF1A6A00-DE41-4D5C-96C9-7CDDD7ECFE2C

Fig. 91C, D, J

##### Type material.

***Holotype***, female, Thailand, Chiang Mai, Doi Pui, 18.841N, 98.899E, 1348 m, 2.ii.2010, S. Sanguansub, ex Fagaceae sp., fallen tree (NHMUK). ***Paratypes***, female, as holotype (MSUC, 1; SSC, 1; RABC, 2).

##### Diagnosis.

2.7–2.75 mm long (mean = 2.73 mm; n = 4); 3.24–3.38× as long as wide. This species is distinguished by its elongate form, the presence of denticles or granules on all declivital interstriae, including interstriae 2, the widening of declivital interstriae 1 from the base of the declivity to the apex; declivital striae not impressed; declivital posterolateral margin costate and bearing a row of spinose granules to interstriae 7; and discal interstriae with punctures much finer than strial punctures, very widely spaced.

##### Similar species.

*Xyleborus dryographus* (Ratzeburg, 1837) (from western Palearctic), *X. muticus*.

##### Description

**(female).** 2.7–2.75 mm long (mean = 2.73 mm; n = 4); 3.24–3.38 × as long as wide. Body dark brown. Legs and antennae light brown. ***Head***: epistoma entire, transverse, with a row of hair-like setae. Frons weakly convex to upper level of eyes; median carina absent; surface shagreened, reticulate, punctate; punctures sparse, large, shallow, setose, each bearing a long, erect hair-like seta. Eyes shallowly emarginate just above antennal insertion, upper part smaller than lower part. Submentum triangular, deeply impressed. Antennal scape regularly thick, slightly longer than club. Pedicel as wide as scape, as long as funicle. Funicle 4-segmented, segment 1 shorter than pedicel. Club approximately circular, obliquely truncate, type 2; segment 1 corneous, transverse on anterior face, occupying basal 1/2, nearly covering posterior face; segment 2 narrow, corneous; segment 1 present on posterior face. ***Pronotum***: 1.36× as long as wide. In dorsal view elongate, conical frontally, type 6, sides parallel on basal 2/3, conical anteriorly; anterior margin without serrations. In lateral view type 7, elongate, disc longer than anterior slope, summit on anterior 2/5. Anterior slope with densely spaced small asperities, becoming lower and more strongly transverse towards summit. Disc subshiny with sparse, large, coarse punctures bearing short, erect hair-like setae, some longer hair-like setae at margins. Lateral margins obliquely costate. Base transverse, posterior angles broadly rounded. ***Elytra***: 2.04× as long as wide, 1.5× as long as pronotum. Scutellum moderately sized, linguiform, flush with elytra, flat, shiny. Elytral base transverse, edge oblique, humeral angles rounded, parallel-sided in basal 4/5, then broadly rounded to apex. Disc shiny, striae not impressed, with small, shallow punctures separated by three diameters of a puncture, glabrous; interstriae flat, very sparsely finely punctate, punctures 1/2 size of strial punctures, each with a short, thick, erect seta. Declivity steep, strongly convex, shiny; striae not impressed, strial punctures larger, coarser than on disc; interstriae 1 widened from base to apex; all interstriae similarly armed, bearing small spines or granules. Posterolateral margin costate, granulate to interstriae 7, granules spinose. ***Legs***: procoxae contiguous; prosternal coxal piece bulging. Protibiae distinctly triangular; posterior face smooth; apical 1/2 of outer margin with six small socketed denticles, their length as long as basal width. Meso- and metatibiae flattened; outer margins obliquely triangular with eight small socketed denticles.

##### Etymology.

The species is named for Dr. Sunisa Sanguansub, the collector, for her contributions to our knowledge of bark and ambrosia beetles. Noun in genitive.

##### Distribution.

Thailand.

##### Host plants.

Recorded only from an unidentified species of Fagaceae.

#### Xyleborus volvulus

Taxon classificationAnimaliaColeopteraCurculionidae

(Fabricius, 1775)

Fig. 91E, F, K

Bostrichus volvulus Fabricius, 1775: 454.
Hylesinus volvulus (Fabricius): Fabricius 1801: 394.
Xyleborus volvulus (Fabricius): Eggers 1929: 47.
Xyleborus torquatus Eichhoff, 1868b: 146. Synonymy: Wood 1960: 69.
Xyleborus alternans Eichhoff, 1869: 280. Synonymy: Eggers 1929: 43.
Xyleborus badius Eichhoff, 1869: 280. Synonymy: Wood 1960: 69.
Xyleborus interstitialis Eichhoff, 1878b: 375. Synonymy: Wood 1982: 833.
Xyleborus guanajuatensis Dugès, 1887: 141. Synonymy: Wood 1983: 650.
Xyleborus grenadensis Hopkins, 1915a: 62, 65. Synonymy: Wood 1972: 200.
Xyleborus hubbardi Hopkins, 1915a: 62, 65. Synonymy: Schedl 1952d: 164.
Xyleborus rileyi Hopkins, 1915a: 62, 65. Synonymy: Bright 1968: 1318.
Xyleborus schwarzi Hopkins, 1915a: 62, 65. Synonymy: Bright 1968: 1318.
Xyleborus continentalis Eggers, 1920: 42. Synonymy: Beaver 2011: 285.
Xyleborus silvestris Beeson, 1929: 241. Synonymy: Wood 1989: 177.
Xyleborus vagabundus Schedl, 1949: 277. Synonymy: Wood 1972: 200.
Xyleborus granularis Schedl, 1950b: 898. Synonymy: Wood 1989: 177.

##### Type material.

***Holotype****Xyleborus continentalis* (MFNB). ***Holotype****Xyleborus silvestris* (NHMUK).

##### Diagnosis.

2.5 mm long (mean = 2.5 mm; n = 5); 3.13× as long as wide. This species is distinguished by the protibiae obliquely triangular, broadest at distal 1/3; declivity smooth, shiny (specimen must be dry); declivital interstriae 1 and 3 armed with two or three pairs of moderate tubercles; interstriae 2 sparsely granulate along its entire length; and elytra unicolored.

This species is almost identical to *X. perforans*, which is distinguished by its stouter form (2.67–2.89× as long as wide), and interstriae 2 granules only present at declivity summit.

##### Similar species.

*Xyleborus affinis*, *X. cognatus*, *X. ferrugineus*, *X. festivus*, *X. perforans*, *X. pfeilii*.

##### Distribution.

Probably of American origin (Wood 2007; Gohli et al. 2016) but now in temperate and tropical regions around the world. In the study region recorded from India (Nicobar Is), Bangladesh, Myanmar, Taiwan, Thailand.

##### Host plants.

Strongly polyphagous (Browne 1961b; Schedl 1963a, as *X. torquatus*).

##### Remarks.

Specimens from Southeast Asia were not available for examination. The measurements and diagnosis are based on specimens from Panama (Panama) and the United States (Florida).

Wood and Bright (1992) considered reports of the species ranging from Southeast Asia to the Southwest Pacific as referring to *X. perforans*. Some of the records from the countries of the study region given above may refer to *X. perforans* (Beaver et al. 2014). However, molecular studies have confirmed that the species does occur in Bangladesh and Thailand (Gohli et al. 2016).

Wood and Bright (1992) postulated that *X. pfeilii* is a synonym of *X. volvulus* and this was further suggested by Gomez et al. (2018a). Though appearing quite similar, the protibiae of these species are different. That of *X. pfeilii* is distinctly triangular while that of *X. volvulus* is obliquely triangular. Analysis of COI and CAD sequences has also shown that these species are separate lineages (Cognato et al. 2020b) and the validity of *X. pfeilii* is supported.

#### Xyleborus yunnanensis sp. nov.

Taxon classificationAnimaliaColeopteraCurculionidae

http://zoobank.org/049DCADD-70FB-423D-9135-D088AEBD344D

Fig. 91G, H, L

##### Type material.

***Holotype***, female, China: S-Yunnan, Xishuangbanna, 28 km NW Jinghong, vic. An Ma Xi Chan (NNNR), 22°12'N, 100°38'E, 700 m, forest, EKL, 05.iv.2009, L. Meng (NKME). ***Paratypes***, female, as holotype (RABC, 1); as holotype except: 28.vi.2008, A. Weigel (MSUC, 1).

##### Diagnosis.

2.7–2.75 mm long (mean = 2.73 mm; n = 3); 3.06–3.17× as long as wide. This species is distinguished by the declivital interstriae 1 widened from base to midpoint of declivity, then narrowed to apex; declivital striae not impressed; three strong spines on declivital interstriae 1, and three slightly weaker spines on declivital interstriae 3, declivital interstriae 2 with at most a small spine near top of declivity; all interstriae with spines only, lacking granules; discal interstriae much wider than striae, strial punctures approximately 2× diameter of interstrial punctures, the latter very sparse on disc; declivital posterolateral margin costate and with a row of small spines to interstriae 6; integument smooth and strongly shiny on both dorsal and ventral surfaces, only the head reticulate and less shiny.

##### Similar species.

*Xyleborus mysticulus*, *X. pfeilii*.

##### Description

**(female).** 2.7–2.75 mm long (mean = 2.73 mm; n = 3); 3.06–3.17 × as long as wide. Body red-brown. Legs and antennae light brown. ***Head***: epistoma entire, transverse, with a row of hair-like setae. Frons weakly convex to upper level of eyes; median carina absent; surface shagreened, alutaceous, punctate; punctures sparse, shallow, setose, each bearing a long, erect hair-like seta. Eyes shallowly emarginate just above antennal insertion, upper part smaller than lower part. Submentum triangular, deeply impressed. Antennal scape regularly thick, slightly longer than club. Pedicel as wide as scape, as long as funicle. Funicle 4-segmented, segment 1 shorter than pedicel. Club longer than broad, obliquely truncate, type 2; segment 1 corneous, transverse on anterior face, occupying basal 2/5, nearly covering posterior face; segment 2 narrow, corneous; segment 1 present on posterior face. ***Pronotum***: 1.33× as long as wide. In dorsal view long and rounded frontally, type 7, sides parallel in basal 3/4, rounded anteriorly; anterior margin without serrations. In lateral view elongate, disc longer than anterior slope, type 7, summit on anterior 1/3. Anterior slope with densely spaced, moderately large asperities, becoming lower and more strongly transverse towards summit. Disc shiny with sparse, fine punctures bearing long, fine, erect hair-like setae, some longer hair-like setae at margins. Lateral margins obliquely costate. Base transverse, posterior angles broadly rounded. ***Elytra***: 1.79× as long as wide, 1.34× as long as pronotum. Scutellum moderately sized, linguiform, flush with elytra, flat, shiny. Elytral base transverse, edge oblique, humeral angles rounded, parallel-sided in basal 3/4, then broadly rounded to apex. Disc shiny, striae not impressed, with small, shallow punctures separated by three diameters of a puncture, glabrous; interstriae flat, very sparsely finely punctate, punctures 1/2 size of strial punctures, each with a short, thick, erect seta. Declivity strongly convex, steep, shiny; striae not impressed, strial punctures larger, coarser than on disc; interstriae 1 widened from base to declivital midpoint, then narrowed to apex; interstriae 1 with three strong spines, interstriae 2 with at most a small spine near declivital summit, interstriae 3 with three slightly weaker spines than those of interstriae 1. Posterolateral margin costate, granulate to interstriae 6. ***Legs***: Procoxae contiguous; prosternal coxal piece bulging. Protibiae distinctly triangular, posterior face smooth; apical 1/2 of outer margin with five large socketed denticles, their length much longer than basal width. Meso- and metatibiae flattened; outer margins obliquely triangular with six large socketed denticles.

##### Etymology.

The specific name refers to the Chinese province where it was collected. Latinized adjective.

##### Distribution.

China (Yunnan).

##### Host plants.

Unknown.

### *Xylosandrus* Reitter, 1913

#### Xylosandrus

Taxon classificationAnimaliaColeopteraCurculionidae

Reitter, 1913

Xylosandrus Reitter, 1913: 83.
Apoxyleborus Wood, 1980: 90. Synonymy: Wood 1984: 229.

##### Type species.

*Xyleborus morigerus* Blandford, 1894a; monotypy.

##### Diagnosis.

*Xylosandrus* species are small to moderately sized, 1.3–3.9 mm, and stout 1.79–2.6× as long as wide. *Xylosandrus* is distinguished by the procoxae widely separated (narrowly separated in *X. formosae*); pronotum with a median mycangial tuft (absent in *X. formosae*); antennal club type 1, obliquely truncate with segment 1 covering the posterior face (flat and type 4 in *X. spinifer*); eyes moderately to deeply emarginate; scutellum visible, flat, flush with elytra; lateral margin of the pronotum obliquely costate; protibiae distinctly triangular or slender with fewer than six large socketed denticles; and declivity with zero, five or six striae.

##### Similar genera.

*Amasa*, *Anisandrus*, *Cnestus*, *Diuncus*, *Hadrodemius*. *Xylosandrus* is closely related to *Anisandrus*, *Cnestus*, and *Hadrodemius*, all of which possess a mesonotal mycangium and the associated dense tuft of hair-like setae at the scutellar area and pronotal base (Gohli et al. 2017; Johnson et al. 2018).

##### Distribution.

Globally distributed throughout temperate and tropical forests.

##### Gallery system.

The species typically breed in small diameter stems. The gallery system consists of a radial gallery leading to an irregular chamber in the center of the stem with longitudinal branches extending up and down the stem.

##### Remarks.

*Xylosandrus* was recently revised by Dole and Cognato (2010) but two additional species have since been described (Gomez et al. 2020; Park et al. 2020) and one species, *X. ramulorum* (Schedl, 1957), was transferred from *Amasa* (Sittichaya et al. 2019). Preliminary phylogenies suggest that *Anisandrus maiche* is monophyletic with *Xylosandrus* (Cognato et al. 2020b).

#### Key to species (females only)

**Table:** 

1	Procoxae narrowly separated; pronotal mycangial tuft absent (Fig. 95D)	*** formosae ***
–	Procoxae widely separated; pronotal mycangial tuft present, sparsely (Fig. 95B) to densely setose (Fig. 95H)	**2**
2	Declivital summit armed by a pair of very large spines; antennal club flat, type 4, with three sutures on posterior face (Fig. 3)	***spinifer* sp. nov.**
–	Declivital summit unarmed, granulate or denticulate; antennal club obliquely truncate, type 1, with no sutures on posterior face (Fig. 2)	**3**
3	Elytra truncate, posterolateral margin acutely carinate, forming a continuous circumdeclivital carina (Fig. 92D, J)	**4**
–	Elytra rounded (Fig. 92B, I) or obliquely truncate (Fig. 92F, K), posterolateral margin carinate to interstriae 7, never forming a continuous circumdeclivital carina	**6**
4	Circumdeclivital carina never granulate; declivital interstriae 1 uniformly weakly costate; smaller, 2.8 mm	*** amputatus ***
–	Circumdeclivital carina granulate on apical 1/3; sutural margin costate, costa increasing in height and size from base to apex; larger, 3.2–3.9 mm	**5**
5	Declivital face with declivital striae and interstrial punctures replaced by confused granules; larger, 3.9 mm	***bellinsulanus* sp. nov.**
–	Declivital face with four punctate striae; strial punctures large; interstriae granulate, granules more abundant near apex; smaller, 3.2–3.6 mm	*** mancus ***
6	Declivital striae punctate (Fig. 96G, L)	**7**
–	Declivital striae granulate (Fig. 93A, I)	**15**
7	Declivity with five punctate striae; declivity obliquely truncate	*** derupteterminatus ***
–	Declivity with six punctate striae; declivity rounded	**8**
8	Elytral disc strongly convex and appearing strongly humped (Fig. 96H), much shorter than declivity; pronotum rounded in lateral view (type 1)	*** morigerus ***
–	Elytral disc flat or weakly convex, not appearing humped (Fig. 94B), gradually curving toward declivity, at least as long as declivity; pronotum basic in lateral view (type 0)	**9**
9	Pronotum wider than long, 0.82–0.9× as long as wide; minute 1.3–1.7 mm	*** mesuae ***
–	Pronotum as long as wide or longer than wide, 1.0–1.1× as long as wide; generally larger, 1.5–2.4 mm	**10**
10	Pronotum as long as wide	**11**
–	Pronotum 1.1× as long as wide	**14**
11	Declivital interstriae denticulate-granulate, apices of granules acute (Fig. 94I)	**12**
–	Declivital interstriae granulate, apices of granules round (Fig. 95K)	**13**
12	Declivital striae setose, setae semi-recumbent and equal to the width of an interstria; interstrial setae erect, hair-like, longer than the width of two interstriae; smaller, 2.0 mm and stout, 2.0× as long as wide	*** adherescens ***
–	Declivital striae glabrous; interstrial setae erect, minute, less than the width of an interstria; larger, 2.15–2.36 mm and elongate, 2.4–2.56× as long as wide	*** dentipennis ***
13	Declivital striae setose, setae semi-recumbent hair-like setae and equal in length to the width of an interstria; smaller, 1.5–1.9 mm	*** compactus ***
–	Declivital striae glabrous; larger, 2.0–2.3 mm	*** eupatorii ***
14	Declivital striae feebly impressed, strial punctures small, shallow (Fig. 95K); larger and more elongate, 2.3–2.4 mm long, 2.3–2.56× as long as wide	*** germanus ***
–	Declivital striae clearly impressed, strial punctures large, deep (Fig. 96K); smaller, 1.8–2.3 mm, and stouter, 2.09–2.25× as long as wide	*** metagermanus ***
15	Declivity rounded, disc gradually curving into declivity; pronotum from dorsal view rounded (type 1), lateral view basic (type 0), summit at midpoint; pronotal disc shiny, finely minutely punctate; mycangial tuft on the pronotal base sparse	*** crassiusculus ***
–	Declivity obliquely truncate, disc abruptly separated from steep declivity; pronotum from dorsal view conical frontally (type 6), lateral view tall (type 2), summit at basal 1/4; pronotal disc dull, coarsely densely punctate; mycangial tuft on the pronotal base dense	**16**
16	Declivital strial granules relatively small, as large as those of interstriae	*** brevis ***
–	Declivital strial granules relatively large, at least 1.5× as large as those of interstriae (rarely a few interstrial granules as large as strial in *X. borealis*)	**17**
17	Declivital striae without setae	*** diversepilosus ***
–	Declivital striae setose	**18**
18	Declivital striae and interstriae only bearing recumbent setae on face (some erect setae may be present on margins); declivital face densely setose, its surface obscured; declivital face flattened, depressed below margins (Fig. 97I)	**19**
–	Declivital striae and interstriae bearing semi-recumbent or semi-erect setae and interstriae bearing a row of long erect setae; declivital face moderately setose, its surface readily visible; declivital face convex, flush with margins (Fig. 97H)	**20**
19	Declivital strial and interstrial setae recumbent, thick and scale-like, less than 1/2 width of an interstria; smaller, 2.5–2.9 mm	*** subsimilis ***
–	Declivital striae and interstrial setae recumbent, fine and hair-like, equal to the width of an interstria; larger, 3.0 mm	*** jaintianus ***
20	Declivital interstrial granules large, prominent; declivital surface dull or opalescent	*** borealis ***
–	Declivital interstrial granules small, inconspicuous; declivital surface shiny	**21**
21	Declivital interstriae with a row of erect, slightly thickened, bristle-like setae, their apices blunt	*** discolor ***
–	Declivital interstriae with a row of erect, fine, hair-like setae, their apices pointed	**22**
22	Declivital stria1 and interstria1 setae recumbent, very fine, hair-like; interstriae with a row of erect setae equal in length to the width of an interstria; usually larger, 2.45–3.0 mm	*** subsimiliformis ***
–	Declivital strial and interstrial setae semi-recumbent, hair-like; interstriae with a row of erect setae longer than the width of 1.5 interstriae; usually smaller, 2.2–2.7 mm	*** beesoni ***

#### Xylosandrus adherescens

Taxon classificationAnimaliaColeopteraCurculionidae

Schedl, 1971

Fig. 92A, B, I

Xylosandrus adherescens Schedl, 1971b: 375.

##### Type material.

***Holotype*** (NHMW).

##### New records.

Vietnam: Dong Nai, Cat Tien N.P., 11.42854, 107.42544, 148 m, 23.ii.2017, VN98, A.I. Cognato, T.A. Hoang, ex 5 cm diameter (MSUC, 2).

##### Diagnosis.

2.0 mm long (n = 3); 2.0× as long as wide. This species is distinguished by its small size; elytral disc flat, gradually curving toward declivity, elytra rounded; posterolateral margins of elytra carinate to interstriae 7; declivital face with six punctate striae, striae setose, setae semi-recumbent and equal to the width of an interstria; interstriae denticulate-granulate, uniseriate with erect hair-like setae longer than the width of two interstriae; pronotum as long as wide, from dorsal view rounded (type 1) and lateral view basic (type 0), summit at midpoint, basal 1/2 smooth, shiny, sparsely minutely punctate; and sparse mycangial tuft on the pronotal base.

##### Similar species.

*Xylosandrus compactus*, *X. derupteterminatus*, *X. mesuae*, *X. morigerus*.

##### Distribution.

Vietnam.

##### Host plants.

Unknown.

##### Remarks.

The gallery of this species was flat and a cave type. It was excavated against the grain of the wood (AIC, pers. obs.).

**Figure 92.**
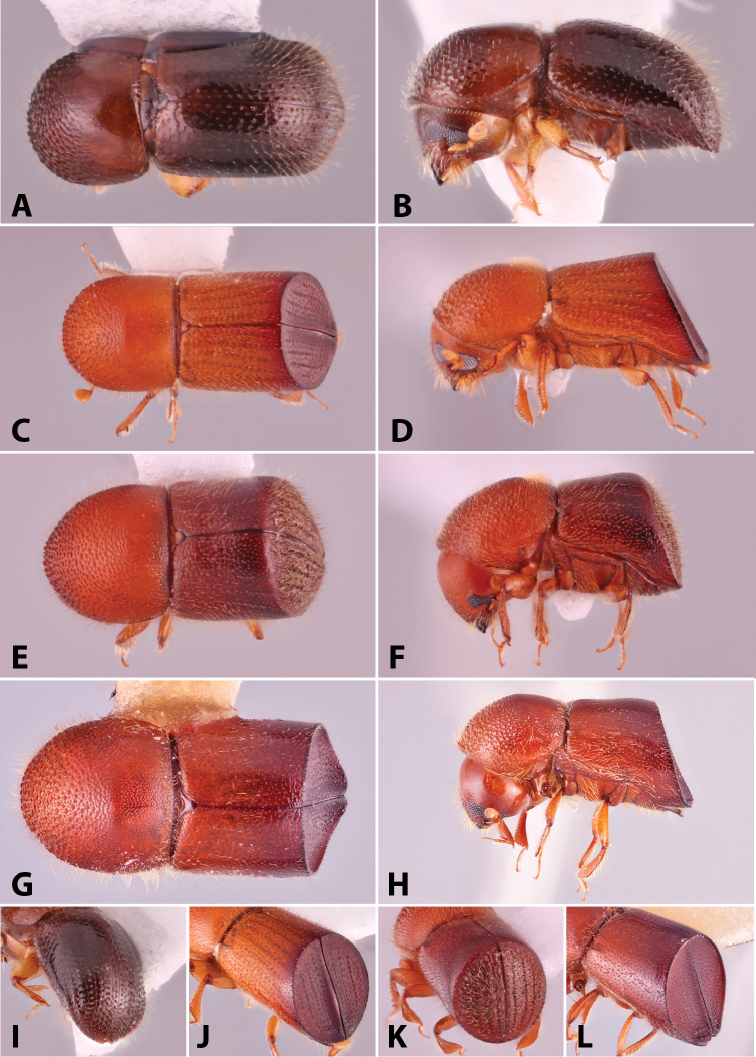
Dorsal, lateral and declivital view of *Xylosandrus adherescens*, 2.0 mm (**A, B, I**), *X. amputatus*, 2.8 mm (**C, D, J**), *X. beesoni*, 2.2–2.7 mm (**E, F, K**), and *X. bellinsulanus* holotype, 3.9 mm (**G, H, L**).

#### Xylosandrus amputatus

Taxon classificationAnimaliaColeopteraCurculionidae

(Blandford, 1894)

Fig. 92C, D, J

Xyleborus amputatus Blandford, 1894c: 575.
Amasa amputatus [*sic*] (Blandford): Wood and Bright 1992: 682.
Xylosandrus amputatus (Blandford): Dole and Cognato 2010: 473.
Xyleborus melli Schedl, 1938: 463. Synonymy: Beaver 2010: 55.

##### Type material.

***Holotype****Xyleborus amputatus* (NHMUK). ***Lectotype****Xyleborus melli* (NHMW).

##### New records.

Cambodia: Sihanouk, Sihanoukville (Rosan Hill), 9–10.ix.2016, 50 m, J.B. Heppner (FSCA, 1). China: Guangdong, W of Qixing, Heishiding Nature Reserve, 23°27.9'N, 114°16.0'E, 190 m, forested stream, valley, at light, 1–3.v.2011, M. Ficáček, J. Hájek (MNHP, 1). Shanghai, Dongchuan, vii–viii.2017, Gao, ex trap w/ querciverol (MSUC, 1). Vietnam: Cao Bang, 22°34.118'N, 105°52.537'E, 1048 m, 12–17.iv.2014, VN9, Cognato, Smith, Pham, ex FIT (MSUC, 4). Dong Nai, Cat Tien N.P., 11.46050, 107.37375, 379 m, 22–24.ii.2017, VN75, A.I. Cognato, T.A. Hoang, ex FIT (MSUC, 1). NE region, Lang Son, Mau Son Nat. Park, 13–14.vi.2016, at lights, 21°51.001'N, 106°55.074'E, G.S. Powell (MSUC, 3).

##### Diagnosis.

2.8 mm long (mean = 2.8 mm; n = 5); 2.15–2.33× as long as wide. This species is distinguished by its moderate size; upper part of eye smaller than lower part; elytral disc weakly ascending apically, longer than declivity; declivital face steep, abruptly separated from disc; elytra truncate; posterolateral margins of elytra carinate to suture forming a circumdeclivital carina; declivital face flat, coarsely shagreened, dull; declivity with four punctate, glabrous, straight striae visible; strial punctures large; interstriae glabrous, punctate; posterolateral margin smooth, not granulate; pronotum as long as wide, from dorsal view rounded (type 1) and lateral view basic (type 0), summit at midpoint, basal 1/2 shiny, densely punctate; and broad, dense mycangial tuft on the pronotal base.

##### Similar species.

*Amasa* spp., *Xylosandrus bellinsulanus*, *X. mancus*.

##### Distribution.

Cambodia*, China (Fujian, Guangdong*, Hunan, Shanghai*, Sichuan), Japan, Korea, Taiwan, Vietnam*. Imported and established in USA (Cognato et al. 2011; Gomez et al. 2018a).

##### Host plants.

Recorded from *Acer* (Aceraceae), *Cinnamomum*, *Machilus* (Lauraceae), *Pelargonium* (Geraniaceae), and *Ziziphus* (Rhamnaceae) (Dole and Cognato 2010).

#### Xylosandrus beesoni

Taxon classificationAnimaliaColeopteraCurculionidae

Saha, Maiti & Chakraborti, 1992

Fig. 92E, F, K

Xylosandrus beesoni Saha, Maiti & Chakraborti, 1992: 11.

##### Type material.

***Holotype*** (ZSI (Maiti and Saha 2004)). Not examined.

##### New records.

China: Yunnan, Kunming, 27.v.2013, J. Hulcr (UFFE, 1); S. Yunnan, Xishuangbanna, 23 km NW Jinghong. vic. Na Ban Village (NNNR), 22.10'N, 100.39'E, 700–1000 m, v–vii.2009, leg. L. Meng (RABC, 1). India: Arunachal Pradesh, Etalin vicinity, 28°36'56"N, 95°53'21"E, 700 m, 12–25.v.2012, L. Dembický (MSUC, 2; ZFMK, 2). Thailand: Chiang Mai, Doi Pui, 16.i.2005, R.A. Beaver (RABC, 1); as previous except: 1400 m, 29.viii-2.ix.2005, W. Puranasakul, ex EtOH trap (RABC, 1); Doi Suthep, ~ 1400 m, 18.x.2004, R.A. Beaver (RABC, 2). Vietnam: Lao Cai, Hoang Lien N.P., 22.35, 103.77, 1500–2000 m, 19.v.2019, VN172, S.M. Smith, A.I. Cognato (MSUC, 13). Ninh Binh, Cuc Phuong N.P., 20.28055, 105.67765, 5–7.iii.2018, 198 m, A.I. Cognato, S.M. Smith, ex FIT (MSUC, 1).

##### Diagnosis.

2.2–2.7 mm long (mean = 2.43 mm; n = 4); 2.0–2.25× as long as wide. This species is distinguished by its moderate size; elytral disc flat, longer than declivity; declivital face steep, abruptly separated from disc; elytra obliquely truncate; posterolateral margins of elytra carinate to interstriae 7; declivital face with four apparent granulate striae (striae 5 short, converging with striae 4 forming a loop); declivital face convex, striae setose, setae semi-recumbent hair-like and less than the width of an interstria; interstriae granulate, granules multiseriate, confused with a uniseriate row of erect hair-like setae longer than the width of 1.5 interstriae and confused semi-erect hair-like setae equal to the width of an interstria; strial granules 2× larger than those of interstriae; pronotum longer than wide, from dorsal view conical frontally (type 6) and lateral view taller (type 2), summit at basal 1/4, basal 1/4 shagreened, dull, densely punctate; and broad, dense mycangial tuft on the pronotal base.

##### Similar species.

*Xylosandrus borealis*, *X. discolor*, *X. diversepilosus*, *X. subsimiliformis*.

##### Distribution.

China (Yunnan)*, India (Arunachal Pradesh*, West Bengal), Thailand*, Vietnam*.

##### Host plants.

Recorded only from *Symplocos* (Symplocaceae) (Maiti and Saha 2004).

##### Remarks.

This species was collected in great abundance by SMS and AIC in Lao Cai province, Vietnam. In nearly all collecting events the species was found in small branches (1–5 cm in diameter) that were dry and often exposed to full sun, an unusual feeding habit, as most other xyleborines are unable to thrive under these conditions. Thai specimens were recorded as *Xylosandrus subsimiliformis* by Dole and Cognato (2010) and Beaver et al. (2014).

#### Xylosandrus bellinsulanus sp. nov.

Taxon classificationAnimaliaColeopteraCurculionidae

http://zoobank.org/341635BC-BE24-4E64-B90E-DC0D4940D1B7

Fig. 92G, H, L

##### Type material.

***Holotype***, female, 海南岛 尖峰, 600 m 1984-III-26 采集者:宋士美 [China: Hainan, Jianfengling Mt., 600 m; 26.iii.1984, Shimei Song] (NMNH).

##### Diagnosis.

3.9 mm long (n = 1); 2.16× as long as wide. This species is distinguished by its large size; lower part of eye larger than upper part; elytral disc ascending apically, longer than declivity; declivital face steep, abruptly separated from disc; elytra truncate; posterolateral margins of elytra carinate to suture forming a circumdeclivital ring; declivital face flat, strongly shagreened, dull, glabrous, no striae visible; sutural margin costate, costa increasing in height and size from base to apex, declivital striae and interstrial punctures replaced by confused granules, granules more abundant near apex (especially between interstriae 1 and 2); declivital posterolateral margin granulate; pronotum wider than long, from dorsal view rounded (type 1) and lateral view basic (type 0), summit at midpoint, basal 1/2 shiny, densely punctate; and broad, dense mycangial tuft on the pronotal base.

##### Similar species.

*Amasa* spp., *Xylosandrus amputatus*, *X. mancus*.

##### Description

**(female).** 3.9 mm long (n = 1); 2.16× as long as wide. Head, antennae, pronotum, elytral disc and legs dark red-brown, declivital face maroon. ***Head***: epistoma entire, transverse, with a row of hair-like setae. Frons weakly convex to upper level of eyes; median carina present; surface shagreened, impunctate, alutaceous, asperate; asperities longitudinal, larger, denser above epistoma, decreasing in density and height dorsally, becoming more weakly raised and sparse by upper level of eyes. Eyes shallowly emarginate just above antennal insertion, upper part smaller than lower part. Submentum narrow, triangular, slightly impressed. Antennal scape regularly thick, approximately as long as club. Pedicel as wide as scape, much shorter than funicle. Funicle 4-segmented, segment 1 as long as pedicel. Club longer than wide, obliquely truncate, type 1; segment 1 corneous, encircling anterior face; segment 2 narrow, concave, corneous on anterior face only; sutures absent on posterior face. ***Pronotum***: 0.81× as long as wide. In dorsal view rounded, type 1, sides parallel in basal 1/2, rounded anteriorly; anterior margin with a row of serrations. In lateral view basic, type 0, disc flat, summit at midpoint. Anterior slope with densely spaced, moderate asperities, becoming lower and more strongly transverse towards summit, bearing long, fine, erect hair-like setae. Some longer hair-like setae at anterior and lateral margins. Disc shiny, alutaceous with very dense, fine punctures, glabrous. Lateral margins obliquely costate. Base transverse, posterior angles broadly rounded. Mycangial tuft present along base, tuft narrow, dense, laterally extending to striae 3. ***Elytra***: 1.38× as long as wide, 1.7 × as long as pronotum. Scutellum moderately sized, linguiform, flush with elytra, flat, shiny. Elytral base transverse, edge oblique, humeral angles rounded, parallel-sided in basal 3/4, then sharply angulate to apex. Disc shiny, striae not impressed, punctures fine, shallow, strongly confused punctures separated by less than one diameter of a puncture, moderately setose, setae dense, erect, hair-like. Declivity truncate, strongly shagreened, glabrous; striae and interstriae strongly confused, indistinguishable, punctures replaced by granules; granules increasing in size and density apically and medially, especially between interstriae 1 and 2; sutural margin costae, costa increasing in height and size from base to apex. Posterolateral margin forming a circumdeclivital carina, carina granulate on apical 1/2. ***Legs***: procoxae widely separated; prosternal coxal piece flat. Protibiae distinctly triangular, broadest at apical 1/4; posterior face smooth; apical 1/3 of outer margin with six large socketed denticles, their length much longer than basal width; apical mucro prominent, strongly incurved. Meso- and metatibiae flattened; outer margins evenly rounded with 13 and 14, small and variably sized socketed denticles, their length no longer than basal width, respectively.

##### Etymology.

L. *bellus* = beautiful; *insulanus* = islander. In reference to the species’ beautiful declivity and its island type locality. Noun in apposition.

##### Distribution.

China (Hainan).

##### Host plants.

Unknown.

##### Remarks.

Locality labels on the holotype are in Chinese and were translated by You Li. An English locality label has been placed on the specimen below the original locality labels.

#### Xylosandrus borealis

Taxon classificationAnimaliaColeopteraCurculionidae

Nobuchi, 1981

Fig. 93A, B, I

Xylosandrus borealis Nobuchi, 1981b: 34.

##### Type material.

***Holotype*** (NIAES).

##### New records.

China: Guangdong, Shimentai, 28.iii.2003, P. Grootaert (RABC, 1). Hong Kong, Tai Po Kau, vi.2017, J. Skelton (MSUC, 1). Taiwan: Nantou, Sun Moon Lake, 28.vii.2014, C.-S. Lin (MSUC, 5).

##### Diagnosis.

2.0–2.2 mm long (mean = 2.12 mm; n = 5); 2.0× as long as wide. This species is distinguished by its moderate size; elytral disc flat, longer than declivity; declivital face steep, abruptly separated from disc; elytra obliquely truncate; posterolateral margins of elytra carinate to interstriae 7; declivital face with four apparent granulate striae (striae 5 short, converging with striae 4 forming a loop); declivital face convex; striae setose, setae semi-recumbent hair-like and equal to the width of an interstria; interstriae granulate, granules multiseriate, confused with a uniseriate row of very long erect hair-like setae longer than the width of two interstriae and confused semi-erect setae approximately the width of an interstria; strial granules large, 1–1.5× larger than those of interstriae; pronotum longer than wide, from dorsal view conical frontally (type 6) and lateral view taller (type 2), summit at basal 1/4, basal 1/4 shagreened, dull, densely punctate; and broad, dense mycangial tuft on the pronotal base.

##### Similar species.

*Xylosandrus beesoni*, *X. discolor*, *X. diversepilosus*.

##### Distribution.

China* (Guangdong*, Hong Kong*), Japan, Korea, Taiwan*.

##### Host plants.

Only reported from *Styrax* (Styracaceae) and *Camellia* (Theaceae) (Dole and Cognato 2010).

**Figure 93.**
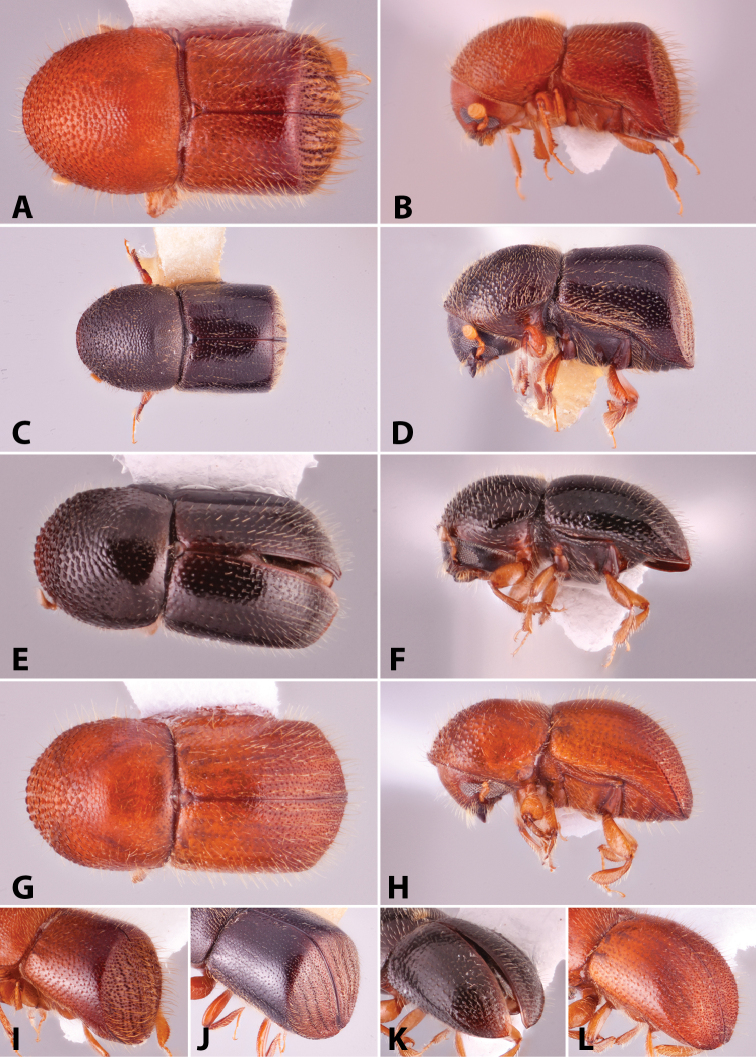
Dorsal, lateral and declivital view of *Xylosandrus borealis*, 2.0–2.2 mm (**A, B, I**), *X. brevis*, 2.75–2.9 mm (**C, D, J**), *X. compactus*, 1.5–1.9 mm (**E, F, K**), and *X. crassiusculus*, 2.3–2.9 mm (**G, H, L**).

#### Xylosandrus brevis

Taxon classificationAnimaliaColeopteraCurculionidae

(Eichhoff, 1877)

Fig. 93C, D, J

Xyleborus brevis Eichhoff, 1877: 121.
Xylosandrus brevis (Eichhoff): Browne 1965: 204.
Xyleborus cucullatus Blandford, 1894b: 121. Synonymy: Murayama 1954: 176.
Xyleborus montanus Niisima, 1910: 13. Synonymy: Smith et al. 2018b: 399.

##### Type material.

***Syntypes*** of *Xyleborus montanus* should be housed in NIAES but have not been located (Smith et al. 2018b).

##### New records.

Taiwan: Nantou, Ren’ai Township, C.-S. Lin, 15.iv.2014 (MSUC, 1).

##### Diagnosis.

2.75–2.90 mm long (mean = 2.87 mm; n = 5); 2.04–2.07× as long as wide. This species is distinguished by its moderate size; elytral disc flat, longer than declivity; declivital face steep, abruptly separated from disc; elytra obliquely truncate; posterolateral margins of elytra carinate to interstriae 7; declivital face with four apparent granulate striae (striae 5 short, converging with striae 4 forming a loop); declivital face convex; declivital striae and interstriae setose, setae recumbent, hair-like and equal to the width of an interstria; declivital interstriae granulate, granules multiseriate, confused with erect hair-like setae longer than the width of two interstriae; strial granules small, approximately equal to those of interstriae; pronotum longer than wide, from dorsal view conical frontally (type 6) and lateral view taller (type 2), summit at basal 1/4, basal 1/4 shagreened, dull, densely punctate; and broad, dense mycangial tuft on the pronotal base.

##### Similar species.

*Xylosandrus jaintianus*, *X. subsimiliformis*, *X. subsimilis*.

##### Distribution.

China (Xizang, Yunnan), Japan, Korea, Nepal, Taiwan, Thailand.

##### Host plants.

Polyphagous (Dole and Cognato 2010).

#### Xylosandrus compactus

Taxon classificationAnimaliaColeopteraCurculionidae

(Eichhoff, 1876)

Fig. 93E, F, K

Xyleborus compactus Eichhoff, 1876a: 201.
Xylosandrus compactus (Eichhoff): Nunberg 1959: 434.
Xyleborus morstatti Hagedorn, 1912a: 37. Synonymy: Murayama and Kalshoven 1962: 247.

##### Type material.

The holotype of *Xyleborus compactus* was destroyed in the bombing of UHZM in World War II (Wood and Bright 1992).

##### New records.

China: Hong Kong, Tai Po Kau, 17.vi.1965, Lee Kit Ming, Hui Wai Ming, ex hand net (BPBM, 2); as previous except: vi.2017, J. Skelton (MSUC, 1). Jiangsu, Nanjing, Laoshan National Park, Bacai Road, 32.09156N, 118.583701E, 15.viii.2017, Cognato, Li, Gao (MSUC, 2). Vietnam: Cao Bang, 22°37.702'N, 105°54.5467'E, 847 m, 10.iv.2014, VN3, Cognato, Smith, Pham, ex small 2–10 mm angiosperm branches (MSUC, 2). Dong Nai, Cat Tien National Park, near park headquarters, 11°25'23"N, 107°25'41"E, 120 m, 27–31.v.1999, B. Hubley, D. Currie, VIET1H95-99 039, ex flight intercept trap (SEMC, 1).

##### Diagnosis.

1.5–1.9 mm long (mean = 1.68 mm; n = 5); 2.0–2.5× as long as wide. This species is distinguished by its small size; elytral disc gradually curving toward declivity, elytra rounded; elytral disc flat; posterolateral margins of elytra carinate to interstriae 7; declivital face with six punctate striae; striae setose, setae semi-recumbent hair-like and equal in length to the width of an interstria; interstriae granulate, uniseriate with erect hair-like setae longer than the width of two interstriae; pronotum as long as wide, from dorsal view rounded (type 1) and lateral view basic (type 0), summit at midpoint, basal 1/2 smooth, shiny, densely minutely punctate; and sparse mycangial tuft on the pronotal base.

##### Similar species.

*Xylosandrus adherescens*, *X. derupteterminatus*, *X. mesuae*, *X. morigerus*.

##### Distribution.

In temperate and tropical regions around the world. Within the study region recorded from China (Fujian, Guangdong, Guangxi, Guizhou, Hainan, Hong Kong*, Hubei, Hunan, Jiangsu*, Jiangxi, Sichuan, Yunnan, Zhejiang), India (Karnataka, Kerala, Tamil Nadu), ‘Indochina’, South Korea, Taiwan, Thailand, Vietnam. Established in the Neotropics, USA and Europe (Wood 2007; Garonna et al. 2012; Gomez et al. 2018a).

##### Host plants.

Strongly polyphagous (Dole and Cognato 2010).

##### Remarks.

The biology has been reviewed by Browne (1961a), Brader (1964), Le Pelley (1968), Entwhistle (1972) and Beaver (1988) amongst others. This is a species of considerable economic importance because it can attack and breed in healthy shoots and twigs. This can result in the introduction of pathogenic fungi. The main economic host is coffee (*Coffea* spp.) (Rubiaceae), but it is also a pest of tea (*Camellia thea*) (Theaceae) in Japan, of cocoa (*Theobroma cacao*) (Malvaceae) and avocado (*Persea americana*) (Lauraceae) in southeast Asia and elsewhere, and may kill seedlings and saplings of shade and forest trees (e.g., Browne 1968a; Le Pelley 1968; Entwhistle 1972).

#### Xylosandrus crassiusculus

Taxon classificationAnimaliaColeopteraCurculionidae

(Motschulsky, 1866)

Fig. 93G, H, L

Phloeotrogus crassiusculus Motschulsky, 1866: 403.
Xylosandrus crassiusculus (Motschulsky): Wood 1977: 68.
Xyleborus semiopacus Eichhoff, 1878b: 334. Synonymy: Wood 1969: 119.
Xyleborus semigranosus Blandford, 1896b: 211. Synonymy: Schedl 1959: 496.
Dryocoetes bengalensis Stebbing, 1908: 12. Synonymy: Beeson 1915: 297.
Xyleborus mascarenus Hagedorn, 1908: 379. Synonymy: Eggers 1923: 130.
Xyleborus ebriosus Niisima, 1909: 154. Synonymy: Choo 1983: 98.
Xyleborus okoumeensis Schedl, 1935b: 271. Synonymy: Schedl 1959: 496.
Xyleborus declivigranulatus Schedl, 1936d: 30. Synonymy: Schedl 1959: 496.

##### Type material.

***Holotype****Xyleborus semigranosus* (NHMUK).

##### New records.

China: Chongqing, Peng Shui, 10.v.2015, Tian-Shang, ex *Castanea molissima* (RABC, 1); as previous except: Nan Chang, Jiangxi Agric. Univ. orchard, v.2015, Su, T-L., ex *Choerospondias axillaris* (RABC, 1); as previous except: Pengshui, 11.viii.2016, Tian-Shang (RABC, 3). Jiangsu, Nanjing, Laoshan National Park, Bacai Road, 32.09156; 118.583701, 15.viii.2017, Cognato, Li, Gao, ex *Populus* (MSUC, 2). Shanghai, Dongchuan, vii–viii.2017, Gao, ex trap w/ querciverol (MSUC, 4). India: Arunachal Pradesh, Etalin vicinity, 28°36'56"N, 95°53'21"E, 700 m, 12–25.v.2012, L. Dembický (ZFMK, 3). Vietnam: Cao Bang, 22°34.532'N, 105°52.480'E, 1087 m, 11.iv.2014, VN6, Cognato, Smith, Pham, ex 2 cm diameter branches, pithy, soft wood (MSUC, 1). Dong Nai, Cat Tien N.P., 11.43771, 107.42253, 142 m, 21.ii.2017, VN86, A.I. Cognato, T.A. Hoang, ex 5 cm diameter branch (MSUC, 3). Ha Tay, Ba Vi N.P. (lake lodge), 196 m, 3–4.vii.2008, J.B. Heppner (FSCA, 1). Kon Tum, Ngoc Linh, 2 km S., 15°5'18"N, 107°55'42"E, 1070 m, 7–12.ix.1998, B. Hubley, D.C. Currie, VIET1H95-99 046, ex malaise trap (SEMC, 1). NE region, Lang Son, Mau Son Nat. Park, 13–14.vi.2016, at lights, 21°51.001'N, 106°55.074'E, G.S. Powell (MSUC, 1). Thua Thien-Hue, Bach Ma N.P., 16.22897, 107.85349, 415 m, 15.ii.2017, VN57, A.I. Cognato, T.A. Hoang, ex 5 cm diameter branch; twig (MSUC, 9). Tuyen Quang, Doi Can Tuyen Quang, 21.72740, 105.22742, 15.iv.2015, R.J. Rabaglia, ex funnel trap (RJRC, 2). Yen Bai, Mau A’, 21.88226, 104.68040, 15.iv.2015, R.J. Rabaglia, ex funnel trap (RJRC, 1); as previous except: Tan Huong, 21.82410, 104.89651 (RJRC, 1).

##### Diagnosis.

2.3–2.9 mm long (mean = 2.58 mm; n = 5); 2.17–2.42× as long as wide. This species is distinguished by the moderate to large size; elytral disc gradually curving toward declivity, elytra rounded; posterolateral margins of elytra carinate to interstriae 7; declivital face with six striae; interstriae and striae granulate, confused, appearing dull, with erect hair-like setae longer than the width of two interstriae; pronotum as long as wide, pronotum from dorsal view rounded (type 1) and lateral view basic (type 0), summit at midpoint, basal 1/2 smooth, shiny, sparsely minutely punctate; and dense mycangial tuft on the pronotal base.

##### Similar species.

None.

##### Distribution.

In temperate and tropical regions around the world. Within the study region recorded from Bhutan, Cambodia, China (Anhui, Fujian, Guangdong, Guangxi, Guizhou, Hainan, Hebei, Hong Kong, Hubei, Hunan, Jiangsu*, Jiangxi, Shaanxi, Shandong, Shanghai*, Sichuan, Xizang, Yunnan, Zhejiang), India (Andaman Is, Arunachal Pradesh*, Assam, Himachal Pradesh, Karnataka, Meghalaya, Sikkim, Tamil Nadu, Uttarakhand, West Bengal), Laos, Myanmar, Nepal, Taiwan, Thailand, Vietnam. Also present in South & North Korea. Imported to and established in Europe, North, Central and South America (Kirkendall and Ødegaard 2007; Pennacchio et al. 2003; Flechtmann and Atkinson 2016; Landi et al. 2016; Gallego et al. 2017; Gomez et al. 2018a).

##### Host plants.

Strongly polyphagous (Dole and Cognato 2010).

##### Remarks.

The basic biology has been described by Browne (1961a), Schedl (1963a) (both as *Xyleborus semiopacus*), and Ranger et al. (2016) amongst others. Flight activity, and the attraction of flying adults to ethanol has been studied in the southern USA by Reding et al. (2011, 2013), attack densities and adult emergence on various hosts by Mayfield et al. (2013), and attraction to volatiles from the symbiotic ambrosia fungus by Hulcr et al. (2011). This is a species of economic importance because, like *X. compactus*, it can attack and breed in healthy shoots and twigs. This can result in the introduction of pathogenic fungi (Sreedharan et al. 1991; Davis and Dute 1997). It seems to be an infrequent pest in the Oriental and Afrotropical regions, although attacks on transplants have been recorded (e.g., Browne 1968a). It is of greater importance in USA, where its ecology and management in plant nurseries is discussed by Ranger et al. (2016).

#### Xylosandrus dentipennis

Taxon classificationAnimaliaColeopteraCurculionidae

Park & Smith, 2020

Fig. 94A, B, I

Xylosandrus dentipennis Park & Smith, 2020 (in Park et al. 2020): 224.

##### Type material.

***Holotype*** (RIFID), ***paratypes*** (MFNB, 1; MSUC, 2; UFFE, 3).

##### Diagnosis.

2.15–2.36 mm long (mean = 2.3 mm; n = 2); 2.4–2.56× as long as wide. This species is distinguished by its moderate size; elytral disc gradually curving toward declivity, elytra rounded; posterolateral margins of elytra carinate to interstriae 7; declivital face with six punctate striae; declivital interstriae denticulate-granulate, uniseriate with minute erect setae less than the width of an interstria; pronotum as long as wide; pronotum from dorsal view rounded (type 1) and lateral view basic (type 0), summit at midpoint, basal 1/2 smooth, shiny, sparsely minutely punctate; and sparse mycangial tuft on the pronotal base.

##### Similar species.

*Xylosandrus eupatorii*, *X. germanus*, *X. metagermanus*.

##### Distribution.

China (Fujian, Guizhou, Jiangxi, Shanghai, Yunnan), Japan, South Korea.

##### Host plants.

This species has only been recorded from *Magnolia* (Magnoliaceae) and *Camptotheca* (Nyssaceae).

**Figure 94.**
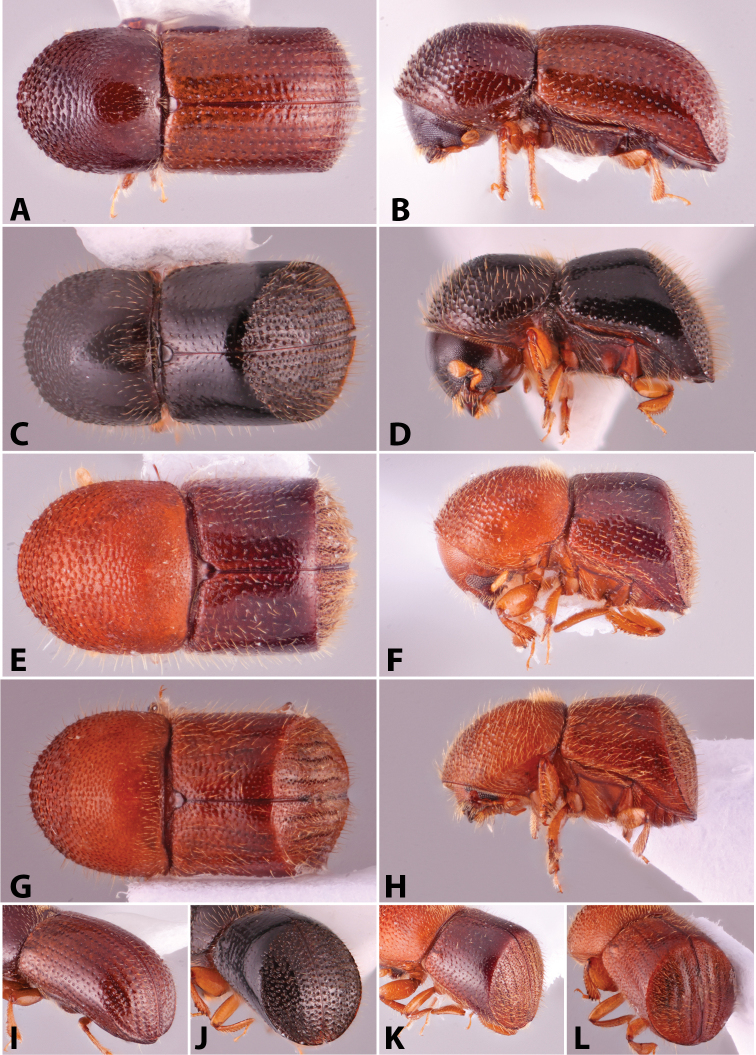
Dorsal, lateral and declivital view of *Xylosandrus dentipennis* paratype, 2.15–2.4 mm (**A, B, I**), *X. derupteterminatus*, 2.0–2.3 mm (**C, D, J**), *X. discolor*, 2.0–2.4 mm (**E, F, K**), and *X. diversepilosus*, 2.1–2.4 mm (**G, H, L**).

#### Xylosandrus derupteterminatus

Taxon classificationAnimaliaColeopteraCurculionidae

(Schedl, 1951)

Fig. 94C, D, J

Xyleborus derupteterminatus Schedl, 1951a: 64.
Xylosandrus derupteterminatus (Schedl): Schedl 1964c: 213.

##### Type material.

***Holotype*** (NHMW).

##### New records.

China: S-Yunnan, Xishuangbanna, 20 km NW Jinghong, Man Dian (NNNR), 740 m, 22°07.80'N, 100°40.05'E, forest, BF, 23.v.2008, A. Weigel (RABC, 1); as previous except: 15.vi.2008, GS/BS, rubber plantation (RABC, 1). Thailand: Chaiyaphum, Pa Hin Ngam N.P., Thepana w’fall, 15°38.948'N, 101°25.625'E, savanna, 23–24.x.2006, K. Sa-nog, B. Adnafai, pan traps (RABC, 1). Chiang Mai, Doi Pui, Chang Khian Highl. Res. Stn, 3.vii.2013, S. Buranapanichpan, ex *Mangifera indica* (RABC, 1); Doi Chiang Dao N.P. HQ, 19°24.278'N, 98°55.311'E, 491 m, 2–3.viii.2007, S. Jugsu, A. Watwanich, pan trap (MSUC, 1; QSBG, 1); as previous except: 5–6.viii.2007 (RABC, 1); as previous except: Doi Chiang Dao WS, nat. trail, 30.ix–1.x.2007, Songkran & Apichart (QSBG, 1); as previous except: 30.ix–7.x.2007, Malaise trap (RABC, 1); Doi Phahompok N.P. HQ, 19°57.961'N, 99°9.355'E, 569 m, 11–18.vii.2007, Wongchai P., Malaise trap (QSBG, 1). Chumphon, 1.v.2010, W. Sittichaya, ex EtOH trap in durian plantn (RABC, 2). Nakhon Nayok, Khao Yai N.P., entrance of Hnong Pak Chee Trail, 14°27.115'N, 101°21.951'E, 733 m, 8–9.v.2007, W. Sukho, pan trap (QSBG, 1). Nakhon Si Thammarat, Kiriwong village, 20.vi.2015, #20, S. Steininger and W. Sittichaya (MSUC, 1).

##### Diagnosis.

2.0–2.3 mm long (mean = 2.18; n = 5); 1.82–2.0× as long as wide. This species is distinguished by the small size; declivity obliquely truncate, abruptly separated from disc; posterolateral margins of elytra carinate to interstriae 7; declivital face with five punctate striae; declivital interstriae densely uniseriate granulate; pronotum from dorsal view rounded (type 1) and lateral view basic (type 0), pronotal summit at midpoint, basal 1/2 smooth, shiny, sparsely minutely punctate; and sparse mycangial tuft on the pronotal base.

##### Similar species.

*Xylosandrus adherescens*, *X. compactus*, *X. mesuae*, *X. morigerus*.

##### Distribution.

China* (Yunnan), Indonesia (Java, Moluccas, Sulawesi), Thailand*.

##### Host plants.

Recorded only from *Mangifera indica* (Anacardiaceae) and *Agathis* (Araucariaceae).

#### Xylosandrus discolor

Taxon classificationAnimaliaColeopteraCurculionidae

(Blandford, 1898)

Fig. 94E, F, K

Xyleborus discolor Blandford, 1898: 429.
Xylosandrus discolor (Blandford): Browne 1963: 55.
Xyleborus posticestriatus Eggers, 1939b: 119. Synonymy: Dole and Cognato 2010: 488.

##### Type material.

***Holotype****Xyleborus discolor* (NHMUK). ***Lectotype****Xyleborus posticestriatus* (NMNH).

##### New records.

China: Chongqing, Peng Shui, 10.v.2015, Tian-Shang, ex *Castanea molissima* (RABC, 1). Guangxi, Beihai, Yintan, viii.2015, Su T-L. (RABC, 1). Hong Kong, Pokfulan, 150 m, 31.v.1964, J.L. Gressitt (BPBM, 1). Jiangxi, Gan Zhou, 5.vii.2015, Lv-Jia (RABC, 1); as previous except: Jin Xian, 4.v.2016, Lv-Jia, ex *Cinnamomum camphora* (RABC, 1). Laos: Bolikhamxai, Ban Nape (8 km NE), 18°21'N, 105°08'E, 600 m, 1–18.v.2001, V. Kubáň (NHMB, 1). Louangnamtha, Namtha to Muang Sing, 21°09'N, 101°19'E, 900–1200 m, 5–31.v.1997, V. Kubáň (RABC, 1). Vietnam: Ninh Binh, Cuc Phuong N.P., 10–16.ix.2013, J.B. Heppner (FSCA, 5); as previous except: Mac Lake. 20°15'29.0"N, 105°42'27.5"E, 155 m, 4–7.v.2009, ex blacklight trap (FSCA, 1).

##### Diagnosis.

2.0–2.4 mm long (mean = 2.02 mm; n = 5); 2.0–2.29× as long as wide. This species is distinguished by its moderate size; elytral disc flat, longer than declivity; declivital face steep, abruptly separated from disc; elytra obliquely truncate; posterolateral margins of elytra carinate to interstriae 7; declivital face with four apparent granulate striae (striae 5 short, converging with striae 4 forming a loop); declivital face convex; declivital striae setose, setae semi-recumbent hair-like and equal to the width of an interstria; interstriae granulate, granules multiseriate, confused, with a uniseriate row of erect bristles equal in length to the width of an interstria; strial granules at least 2× larger than those of interstriae; pronotum longer than wide, from dorsal view conical frontally (type 6) and lateral view taller (type 2), summit at basal 1/4, basal 1/4 shagreened, dull, densely punctate; and broad, dense mycangial tuft on the pronotal base.

##### Similar species.

*Xylosandrus beesoni*, *X. borealis*, *X. diversepilosus*.

##### Distribution.

Within the study region recorded from China (Chongqing*, Fujian, Guangdong, Hainan, Hong Kong*, Jiangxi*, Sichuan, Yunnan), India (Andaman Is, Assam, Karnataka, Meghalaya, Sikkim, Tamil Nadu, Uttarakhand, West Bengal), Laos*, Myanmar, Taiwan, Thailand, Vietnam*. Also recorded from Australia, Indonesia, Japan (Ryukyu Is), Malaysia, New Guinea, Philippines, Sri Lanka.

##### Host plants.

Polyphagous (Dole and Cognato 2010).

##### Remarks.

The biology is described by Kalshoven (1959b) and Browne (1961a). Le Pelley (1968) notes that the species attacks green, living branches of coffee in Sri Lanka, but is not considered an important pest.

#### Xylosandrus diversepilosus

Taxon classificationAnimaliaColeopteraCurculionidae

(Eggers, 1941)

Fig. 94G, H, L

Xyleborus diversepilosus (Eggers), 1941b: 224.
Xylosandrus diversepilosus (Eggers): Browne 1963: 55.

##### Type material.

***Holotype*** (ZMFK). Not examined.

##### New records.

China: Guizhou, [no locality], 29.x.2016, Wu, Y-K., ex *Magnolia grandiflora* (RABC, 1).

##### Diagnosis.

2.4 mm long; 1.92× as long as wide. This species is distinguished by its large size; elytral disc flat, longer than declivity; declivital face steep, abruptly separated from disc; elytra obliquely truncate; posterolateral margins of elytra carinate to interstriae 7; declivital face with four apparent granulate striae (striae 5 short, converging with striae 4 forming a loop); declivital face convex; declivital striae glabrous; interstriae granulate, granules multiseriate, confused with a row of erect hair-like setae longer than the width of 1–2 interstriae, granules with an erect hair-like seta; strial granules 2–3× larger than those of interstriae; pronotum longer than wide, from dorsal view conical frontally (type 6) and lateral view taller (type 2), summit at basal 1/4, basal 1/4 shagreened, dull, densely punctate; and broad, dense mycangial tuft on the pronotal base.

##### Similar species.

*Xylosandrus beesoni*, *X. borealis*, *X. discolor*.

##### Distribution.

China (Fujian, Guizhou*), Taiwan.

##### Host plants.

Recorded only from *Magnolia grandiflora* (Magnoliaceae).

#### Xylosandrus eupatorii

Taxon classificationAnimaliaColeopteraCurculionidae

(Eggers, 1940)

Fig. 95A, B, I

Xyleborus eupatorii Eggers, 1940: 140.
Xylosandrus eupatorii (Eggers): Schedl 1964c: 213.

##### Type material.

***Paratypes*** (NMNH, 2).

##### New records.

China: Hainan, Wu-zhi-shan Town, 18.902N, 109.663E, 703 m, 2.xii.2016, Tian-Shang, Lv-Jia (RABC, 2). Hong Kong, Tai Po Kau, vi.2017, J. Skelton (UFFE, 1). Vietnam: Cao Bang, 22°36.804'N, 105°51.982'E, 1831 m, 17.iv.2014, VN42, Cognato, Smith, Pham, ex 0.3–3 cm twigs/branches (MSUC, 1); 22°33.9981'N, 105°52.591'E, 1051 m, 12–17.iv.2014, VN11, Cognato, Smith, Pham, ex FIT (MSUC, 26). Lao Cai, Hoang Lien N.P., 22.35, 103.77, 1500–2000 m, 20.v.2019, VN185, S.M. Smith, A.I. Cognato, ex 1–2 cm branch (MSUC, 2). Thua Thien-Hue, Bach Ma N.P., 16.19718, 107.86002, 1409 m, 14.ii.2017, VN51, A.I. Cognato, T.A. Hoang, ex top half of tree, 10 cm diameter branches (MSUC, 12).

##### Diagnosis.

2.0–2.3 mm long (mean = 2.12 mm; n = 5); 2.22–2.3× as long as wide. This species is distinguished by its moderate size; elytral disc gradually curving toward declivity, elytra rounded; posterolateral margins of elytra carinate to interstriae 7; declivital face with six punctate striae; declivital interstriae uniseriate granulate, with erect hair-like setae longer than the width of two interstriae; pronotum as long as wide, pronotum from dorsal view rounded (type 1) and lateral view basic (type 0), summit at midpoint, basal 1/2 smooth, shiny, sparsely minutely punctate; and sparse mycangial tuft on the pronotal base.

This species is nearly identical to *X. germanus* and is most readily distinguished by the pronotum that is as long as wide; and pronotum base with sparser more dispersed setae.

##### Similar species.

*Xylosandrus dentipennis*, *X. germanus*, *X. metagermanus*.

##### Distribution.

China (Hainan, Hong Kong*, Yunnan), Indonesia (Java), Thailand, Vietnam*.

##### Host plants.

Recorded only from *Eupatorium* (Asteraceae) (Eggers 1940).

**Figure 95.**
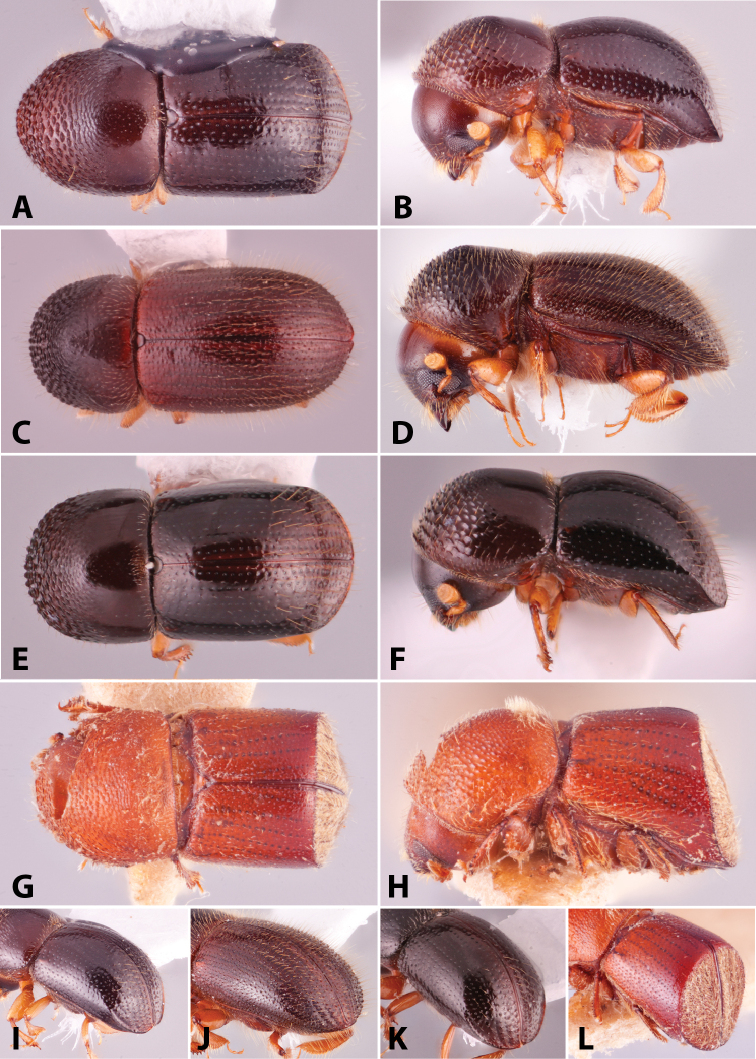
Dorsal, lateral and declivital view of *Xylosandrus eupatorii*, 2.0–2.3 mm (**A, B, I**), *X. formosae*, 2.5–3.0 mm (**C, D, J**), *X. germanus*, 2.3–2.4 mm (**E, F, K**), and *X. jaintianus* holotype, 3.0 mm (**G, H, L**).

#### Xylosandrus formosae

Taxon classificationAnimaliaColeopteraCurculionidae

(Wood, 1992) comb. nov.

Fig. 95C, D, J

Xyleborus formosanus Browne, 1981a: 131.
Xyleborus formosae Wood, 1992: 80 (new name for X. formosanus Browne nec Eggers 1930).
Cyclorhipidion formosanum (Browne): Beaver and Liu 2010: 24.

##### Type material.

***Holotype*** (NHMUK).

##### New records.

China: Fujian, Chong’an, Guidun, 1000 m, 25.vi.1979, Fusheng Huang, ex *Machilus leptophylla* (NMNH, 1). Jiangxi, Wuxi Mt., 16.v.2017, Shengchang Lai, Tian S. et al. (RABC, 1); as previous except: 17.vii.2017 (RABC, 1). Tibet [Xizang], Motuo, 1200 m, 1.ix.1974, Fusheng Huang; ex *Mallotus* sp. (NMNH, 1); as previous except: 800 m, 4.ix.1974, ex *Saurauia tristyla* (NMNH, 1). India: [West] Bengal, Darjeeling, Debrepani, 6000 ft, 11.xi.1929, J.C.M. Gardner, ex *Michelia excelsa* (NMNH, 1); as previous except: 8.xii.1929 (NMNH, 1); as previous except: Kalimpong, Samsingh, 11.v.1934, N.C. Chatterjee, ex *Amoora rohituka* (NMNH, 2). Taiwan: Taipei Co., Fu-Shan, 10.ix.2001, J. & L. Stange, ex malaise trap (FSCA, 1). Thailand: Chiang Mai, Doi Inthanon 5.viii.2002, R.A. Beaver, K. Koivisto (RABC, 1); as previous except: 13.xi.2011, W. Sittichaya (RABC, 2); as previous except: checkpoint 2, 18°31.559'N, 98°29.941'E, 1700 m, 13–21.ix.2006, Y. Areeluck, Malaise trap (QSBG, 1). Vietnam: Cao Bang, 22°36.454'N, 105°52.083'E, 1661 m, 15.iv.2014, VN33, Cognato, Smith, Pham, ex branches from large tree fall (MSUC, 12). Lao Cai, Hoang Lien N.P., 22.35, 103.77, 1500–2000 m, 19.v.2019, VN184, S.M. Smith, A.I. Cognato, ex 6 cm trunk (MSUC, 1). Thua Thien-Hue, Bach Ma N.P., 16.19718, 107.86002, 1409 m, 14.ii.2017, VN51, A.I. Cognato, T.A. Hoang, ex top half of tree, 10 cm diameter branches (MSUC, 6).

##### Diagnosis.

2.5–3.0 mm long (mean = 2.76 mm; n = 5); 2.27–2.55× as long as wide. This species is distinguished by the narrowly separated procoxae; mesonotal mycangial tuft absent; abundant hair-like elytral vestiture; declivital striae and interstriae uniseriate punctate; and declivity rounded, convex, unarmed, surface shagreened, appearing dull.

##### Similar species.

*Anisandrus lineatus*, *Coptodryas inornata*, *Cyclorhipidion* spp., *Euwallacea fornicatus*, *E. kuroshio*, *E. perbrevis*.

##### Distribution.

China* (Fujian, Jiangxi, Xizang), India* (West Bengal), Taiwan, Thailand*, Vietnam*.

##### Host plants.

This species is polyphagous and has been recorded from *Saurauia* (Actinidiaceae), *Machilus* (Euphorbiaceae), *Michelia* (Magnoliaceae), and *Amoora* (Meliaceae).

##### Remarks.

The unusual morphology of this species is superficially similar to that of several other genera (see *similar species* above). This presents a challenge in the generic identification of specimens especially if they are not pinned. Molecular phylogenetics revealed this species belongs in *Xylosandrus* and represents the only known Oriental species of an otherwise Australasian species group comprised of *X. monteithi* Dole and Beaver, *X. rotundicollis* (Browne), and *X. woodi* Dole and Beaver, in SE Asia (Cognato et al. 2020b). It is likely most closely related to *X. monteithi* and *X. woodi* which also lack both a mesonotal mycangial tuft, and a posterolateral declivital carina.

#### Xylosandrus germanus

Taxon classificationAnimaliaColeopteraCurculionidae

(Blandford, 1894)

Fig. 95E, F, K

Xyleborus germanus Blandford, 1894b: 106.
Xylosandrus germanus (Blandford): Hoffman 1941: 38.
Xyleborus orbatus Blandford, 1894b: 123. Synonymy: Nobuchi 1981b: 31.

##### Type material.

***Syntypes****Xyleborus germanus* (NHMUK). ***Holotype****Xyleborus orbatus* (NHMUK).

##### New records.

China: Chongqing, Chengkou, 16.vii.2016, Tian-Shang (RABC, 1); as previous except: Jinfo Mtn., 9.v.2016, Tian-Shang, Lv-Jia, ex Taxodiaceae sp. (RABC, 1); as previous except: Pengshui, 11.viii.2016, Tian-Shang (RABC, 1); as previous except: Simian Mtn., 7.v.2016, Tian-Shang, Lv-Jia (RABC, 1). Jiangsu, Nanjing, Laoshan National Park, Bacai Road, 32.09156N, 118.583701E, 15.viii.2017, Cognato, Li, Gao (MSUC, 2). Jiangxi, Nanchang, Jiangxi Agric. Univ. orchard, v.2015, Su, T-L., ex *Choerospondias axillaris* (RABC, 2); as previous except: Jin Xian, 4.v.2016, Lv-Jia, ex *Cinnamomum camphora* (RABC, 1); as previous except: Xun Wu, 18.vii.2016, Lv-Jia, Lai, S-C., ex *Citrus reticulata* (RABC, 1).

##### Diagnosis.

2.3–2.4 mm long (mean = 2.32 mm; n = 5); 2.3–2.56× as long as wide. This species is distinguished by its moderate size; elytral disc gradually curving toward declivity, elytra rounded; posterolateral margins of elytra carinate to interstriae 7; declivital face with six punctate striae; declivital interstriae granulate, uniseriate with erect hair-like setae longer than the width of 1.5 interstriae; pronotum 1.1× long as wide, pronotum from dorsal view rounded (type 1) and lateral view basic (type 0), summit at midpoint, basal 1/2 smooth, shiny, sparsely minutely punctate; and sparse mycangial tuft on the pronotal base.

This species is nearly identical to *X. eupatorii* and is most easily distinguished by the pronotum 1.1× as long as wide and the pronotal base with more dense setae and from *X. metagermanus* by the smaller, shallower strial punctures and feebly impressed striae.

##### Similar species.

*Anisandrus dispar*, *A. maiche*, *A. paragogus*, *Xylosandrus dentipennis*, *X. eupatorii*, *X. metagermanus*.

##### Distribution.

China (Anhui, Chongqing*, Fujian, Guangdong, Guangxi, Guizhou, Hainan, Henan, Hubei, Hunan, Jiangsu*, Jiangxi*, Shaanxi, Shanxi, Sichuan, Xizang, Yunnan, Zhejiang), Taiwan, Vietnam. Also present in Japan, Korea, Russia (Far East, Sakhalin, Kurile Is). Introduced to and established in Europe and Turkey, USA (including Hawaii) and Canada (Gomez et al. 2018a). The record in Dole and Cognato (2010) from Thailand is incorrect. The cited specimens belong to the closely similar species, *Xylosandrus eupatorii* (see above).

##### Host plants.

Polyphagous (Weber and McPherson 1983b; Dole and Cognato 2010).

##### Remarks.

The basic biology is described by Nobuchi (1981b), Weber and McPherson (1983a), and Ranger et al. (2016). Peer and Taborsky (2004, 2005) have studied male dispersal, variations in sex ratio, and outbreeding depression in the species. Ito et al. (2008) discuss the genetic structure of Japanese populations. Although usually attacking stressed trees, the species sometimes attacks apparently healthy and newly transplanted trees and shrubs (e.g., Nobuchi 1981b; Ranger et al. 2010, 2015). Ranger et al. (2016) discuss the ecology and management of the species in ornamental plant nurseries in USA.

#### Xylosandrus jaintianus

Taxon classificationAnimaliaColeopteraCurculionidae

(Schedl, 1967)

Fig. 95G, H, L

Xyleborus jaintianus Schedl, 1967: 161.
Xylosandrus jaintianus (Schedl): Wood and Bright 1992: 796.

##### Type material.

***Holotype*** (NHMW).

##### Diagnosis.

3.0 mm long (mean = 2.89 mm; n = 1); 2.0× as long as wide. This species is distinguished by its large size; elytral disc flat, longer than declivity; declivital face steep, abruptly separated from disc; elytra obliquely truncate; posterolateral margins of elytra carinate to interstriae 7; declivital face with four apparent granulate striae (striae 5 short, converging with striae 4 forming a loop); declivital face flattened, depressed below declivital margins; declivital striae and interstriae setose, setae recumbent, hair-like, equal to the width of an interstria; interstriae granulate, granules multiseriate, confused; strial granules at least 2× larger than those of interstriae; pronotum longer than wide, from dorsal view conical frontally (type 6) and lateral view taller (type 2), summit at basal 1/4, basal 1/4 shagreened, dull, densely punctate; and broad, dense mycangial tuft on the pronotal base.

##### Similar species.

*Xylosandrus brevis*, *X. subsimiliformis*, *X. subsimilis*.

##### Distribution.

India (Meghalaya), Myanmar, Nepal.

##### Host plants.

Unknown.

#### Xylosandrus mancus

Taxon classificationAnimaliaColeopteraCurculionidae

(Blandford, 1898)

Fig. 96A, B, I

Xyleborus mancus Blandford, 1898: 428.
Apoxyleborus mancus (Blandford): Wood 1980: 90.
Xylosandrus mancus (Blandford): Wood 1984: 229.
Xyleborus abruptus Sampson, 1914: 388. Synonymy: Schedl 1951a: 51.
Xyleborus mancus formosanus Eggers, 1930: 186. Synonymy: Schedl 1952b: 61.

##### Type material.

***Holotype****Xyleborus mancus* (NHMUK).

##### New records.

China: Chongqing, Chengkou, 16.vii.2016, Tian-Shang (RABC, 1); as previous except: Jinfo Mtn., 9.v.2016, Tian-Shang, Lv-Jia (RABC, 1); as previous except: Simian Mtn., 7.v.2016, Tian-Shang, Lv-Jia (RABC, 1); as previous except: Youyang, 14.v.2016, Tian-Shang (RABC, 1). Hong Kong, Lantau Island, San Shek Wan, v.1988, C. O’Connell (BPBM, 1); as previous except: Tai Po Kau, vi.2017, J. Skelton (MSUC, 1). Laos: CE, Bolimkhamxai, Ban Nape (8 km NE), 18°21'N, 105°08'E, 600 m, 1–18.v.2001, V. Kubáň (RABC, 1). Vietnam: Cao Bang, Phia Oac Hotel, 22°37.702'N, 105°54.5467'E, 847 m, 10–17.iv.2014, VN2, Cognato, Smith, Pham, ex FIT (MSUC, 3). Dong Nai, Cat Tien N.P., 11.40817, 107.38098, 134 m, 22–24.ii.2017, VN82, A.I. Cognato, T.A. Hoang, ex 3 cm diameter branch (MSUC, 1). Tonkin, Hoa-Binh, 1932, A De Cooman (MNHN, 1). Vinh Phuc, Me Linh Biological station, Dai Lai 20–21.iv.2015, 100 m, J.B. Heppner (FSCA, 1). Yen Bai, Mau A’, 21.88226, 104.68040, 29.vi.2015, Pham Thu, ex funnel trap (RJRC, 1); as previous except: Tan Huong, 21.82410, 104.89651, 15.iv.2015, R.J. Rabaglia (RJRC, 1).

##### Diagnosis.

3.2–3.6 mm long (mean = 3.46 mm; n = 5); 2.13–2.4× as long as wide. This species is distinguished by its large size; upper part of eye smaller than lower part; elytral disc strongly ascending apically, longer than declivity; declivital face steep, abruptly separated from disc; elytra truncate; posterolateral margins of elytra carinate to suture forming a circumdeclivital ring; declivital face flat, shagreened, four punctate, glabrous and somewhat wavy striae visible; strial punctures large; interstriae glabrous, granulate, granules more abundant near apex (especially between interstriae 1 and 2); declivital posterolateral margin granulate; pronotum as long as wide, from dorsal view rounded (type 1) and lateral view basic (type 0), summit at midpoint, basal 1/2 shiny, densely punctate; and broad, dense mycangial tuft on the pronotal base.

##### Similar species.

*Amasa* spp., *Xylosandrus amputatus*, *X. bellinsulanus*.

##### Distribution.

Within the study region recorded from China (Chongqing*, Gansu, Hainan, Hong Kong*, Xizang, Yunnan), India (Karnataka, Kerala), Laos*, Taiwan, Thailand, Vietnam. Outside the region recorded from East Africa (Tanzania), Indonesia (Java, Sumatra), Madagascar, East & West Malaysia, Mauritius, Philippines, Seychelles, Sri Lanka.

##### Host plants.

Polyphagous (Dole and Cognato 2010).

**Figure 96.**
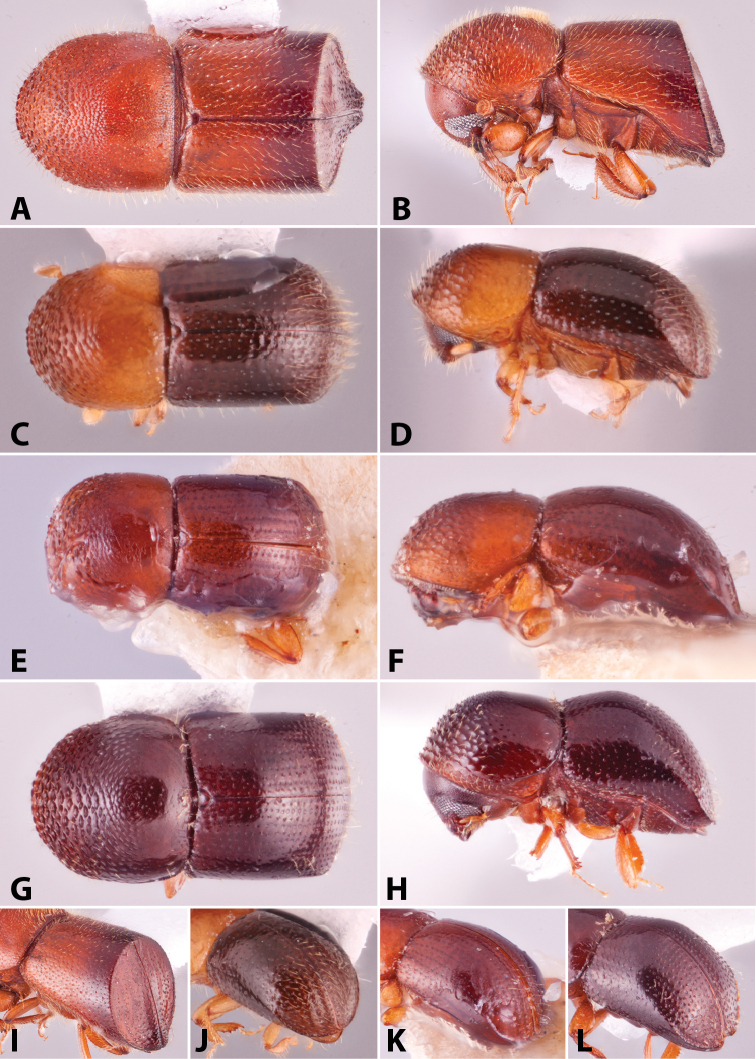
Dorsal, lateral and declivital view of *Xylosandrus mancus*, 3.2–3.6 mm (**A, B, I**), *X. mesuae*, 1.3–1.7 mm (**C, D, J**), *X. metagermanus* holotype, 1.8–2.3 mm (**E, F, K**), and *X. morigerus*, 1.4–2.0 mm (**G H, L**).

#### Xylosandrus mesuae

Taxon classificationAnimaliaColeopteraCurculionidae

(Eggers, 1930)

Fig. 96C, D, J

Xyleborus mesuae Eggers, 1930: 182.
Xylosandrus mesuae (Eggers): Browne 1963: 55.

##### Type material.

***Holotype*** (FRI), ***paratype*** (NMNH, 1).

##### New records.

China: Hong Kong, Tai Po Kau, vi.2017, J. Skelton, ex *Machilus* (MSUC, 3). India: Arunachal Pradesh, Etalin vicinity, 28°36'56"N, 95°53'21"E, 700 m, L. Dembický, 12–25.v.2012 (ZFMK, 1). Taiwan: Yilan, Toucheng Township, 8.vii.2017, C.-S. Lin (MSUC, 1). Thailand: Chumporn *sic* [= Chumphon Province], Durian Orchard, 1.ii.2010, Wisut [Sittichaya], EtOH trap (MSUC, 1). Mae Hong Son, Pang Mapha, Sop Pong, 605 m, 20–24.vii.2009, J.B. Heppner (FSCA, 1).

##### Diagnosis.

1.3–1.7 mm long (mean = 1.5 mm; n = 5); 2.29–2.6× as long as wide. This species is distinguished by its minute size; elytral disc gradually curving toward declivity, elytra rounded; elytral disc convex, disc as long as declivity; posterolateral margins of elytra carinate to interstriae 7; declivital face with six punctate striae; declivital striae setose, setae semi-recumbent hair-like, less than the width of one interstria; interstriae granulate, uniseriate with erect hair-like setae longer than the width of one interstria; pronotum wider than long, 0.82–0.90× as long as wide, from dorsal view rounded (type 1) and lateral view rounded (type 0), summit at midpoint, basal 1/2 smooth, shiny, sparsely minutely punctate; and sparse mycangial tuft on the pronotal base.

##### Similar species.

*Xylosandrus adherescens*, *X. compactus*, *X. derupteterminatus*, *X. morigerus*.

##### Distribution.

China* (Hong Kong*), India (Arunachal Pradesh, Uttarakhand, West Bengal), Sri Lanka, Taiwan, Thailand.

##### Host plants.

Recorded from *Mesua* (Calophyllaceae), *Dipterocarpus*, *Shorea* (Dipterocarpaceae), *Macaranga* (Euphorbiaceae), *Osbeckia* (Melastomataceae) (Dole and Cognato 2010), and *Machilus* (Lauraceae).

#### Xylosandrus metagermanus

Taxon classificationAnimaliaColeopteraCurculionidae

(Schedl, 1951)

Fig. 96E, F, K

Xyleborus metagermanus Schedl, 1951a: 58.
Xylosandrus metagermanus (Schedl): Browne 1963: 55.

##### Type material.

***Holotype*** (NHMW).

##### New records.

India: Assam-Arunachal Pradesh border: Bhalukpong, 27°00'48"N, 92°39'08"E, 150 m, 1–8.v.2012, L. Dembický, ex FIT (ZFMK, 1).

##### Diagnosis.

1.8–2.3 mm long (mean = 2.05 mm; n = 2); 2.09–2.25× as long as wide. This species is distinguished by its small size; elytral disc gradually curving toward declivity, elytra rounded; posterolateral margins of elytra carinate to interstriae 7; declivital face with six punctate and clearly impressed striae; declivital interstriae granulate, uniseriate with erect hair-like setae longer 1–1.5× the width of an interstria; pronotum 1.1× long as wide, pronotum from dorsal view rounded (type 1) and lateral view basic (type 0), summit at midpoint, basal 1/2 smooth, shiny, sparsely minutely punctate; and sparse mycangial tuft on the pronotal base.

This species is very similar to *X. germanus* and is most easily distinguished by the larger, deeper strial punctures and clearly impressed striae.

##### Similar species.

*Xylosandrus dentipennis*, *X. eupatorii*, *X. germanus*.

##### Distribution.

India (Assam).

##### Host plants.

Recorded only from *Gmelina* (Lamiaceae) (Schedl 1951a).

#### Xylosandrus morigerus

Taxon classificationAnimaliaColeopteraCurculionidae

(Blandford, 1894)

Fig. 96G, H, L

Xyleborus morigerus Blandford, 1894a: 264.
Xylosandrus morigerus (Blandford): Reitter 1913: 84.
Xyleborus coffeae Wurth, 1908: 64. Synonymy: Strohmeyer 1910: 86; Schedl 1951b: 136.
Xyleborus difficilis Eggers, 1923: 174. Synonymy: Bright and Skidmore 1997: 4, 169.
Xyleborus luzonicus Eggers, 1923: 174. Synonymy: Wood 1974: 287.
Xyleborus abruptoides Schedl, 1955a: 298. Synonymy: Beaver 1995b: 17.

##### Type material.

***Holotype****Xyleborus abruptoides* (BPBM). ***Lectotype****Xyleborus difficilis* (NMNH). ***Syntypes****Xylosandrus morigerus* (NHMUK).

##### New records.

China: S Yunnan, Xishuangbanna, 20 km NW Jinghong, vic. Man Dian (NNNR), 22°07.80'N, 100°40.0'E, 730 m, forest, 6.vi.2008, A. Weigel (RABC, 1).

##### Diagnosis.

1.4–2.0 mm long (mean = 1.82 mm; n = 5); 2.0–2.33× as long as wide. This species is distinguished by its small size; disc strongly convex, much shorter than declivity; posterolateral margins of elytra carinate to interstriae 7; declivital face with six punctate striae; declivital striae setose, setae minute, semi-recumbent, hair-like; interstriae granulate, uniseriate with erect hair-like setae longer than the width of one interstria; pronotum wider than long, from dorsal view rounded (type 1) and lateral view rounded (type 1), summit at midpoint, basal half smooth, shiny, sparsely minutely punctate; and sparse mycangial tuft on the pronotal base.

##### Similar species.

*Xylosandrus adherescens*, *X. compactus*, *X. derupteterminatus*, *X. mesuae*.

##### Distribution.

Circumtropical. Within the study region recorded from China* (Yunnan), India (Tamil Nadu, West Bengal), Laos, Myanmar, Taiwan, Thailand, Vietnam. Introduced to Europe (Kirkendall and Faccoli 2010) and South and Central America (Wood 1982, 2007).

##### Host plants.

Strongly polyphagous (Dole and Cognato 2010).

##### Remarks.

The biology has been studied by Browne (1961a) and Kalshoven (1961). These and other studies are reviewed by Schedl (1963a) and Le Pelley (1968). The species has some economic importance as a pest of coffee (Kalshoven 1961; Le Pelley 1968) and of other crop trees.

#### Xylosandrus spinifer sp. nov.

Taxon classificationAnimaliaColeopteraCurculionidae

http://zoobank.org/78CB095F-9A93-4BD6-B2C7-94B20F507A31

Fig. 97A, B, G

##### Type material.

***Holotype***, female, Thailand: SE Chanthaburi, 45 m, 25–30.iv.1958 (BPBM). ***Paratypes***, female, China: Hong Kong, Tai Po Kau, vi.2017, J. Skelton, P. Carlson, Y. Li, J Hulcr, uffeID: 31231 (UFFE, 1), uffeID: 31217 (UFFE, 2); Vietnam: N, (Na Hang), 160 km NNW Hanoi, NE env. of Na Hang, 150–200 m NN, 03–13.vi.1996, A. Napolov, I. Roma (RABC, 1).

##### Diagnosis.

3.3 mm long (n = 1); 2.3× as long as wide. This species is unique among all *Xylosandrus* because of the unmistakable pair of very large spines on the declivital summit and a flat antennal club, type 4, with three sutures visible on the posterior face.

*Xylosandrus spinifer* superficially resembles *Diuncus* spp. but can be differentiated by the following characteristics: base of the pronotum has an elongate patch of dense punctures bearing a tuft of setae; anterior margin of pronotum evenly rounded, asperities just above the margin are of equal size, rather than with a median, larger pair; procoxae separated; and posterolateral margin carinate and granulate.

##### Similar species.

*Diuncus* spp.

##### Description

**(female).** 3.3 mm long (n = 1); 2.3× as long as wide. Head, pronotum and elytral disc light red-brown, declivity dark red-brown, antennae and legs light brown. ***Head***: epistoma entire, transverse, with a row of hair-like setae. Frons weakly convex to upper level of eyes; median carina present; surface shagreened, impunctate, alutaceous, asperate; asperities longitudinal, larger, denser above epistoma, decreasing in density and height dorsally, becoming more weakly raised and sparse by upper level of eyes. Eyes very shallowly emarginate just above antennal insertion, upper part smaller than lower part. Submentum narrow, triangular, slightly impressed. Antennal scape regularly thick, approximately as long as club. Pedicel as wide as scape, shorter than funicle. Funicle 4-segmented, segment 1 as long as pedicel. Club longer than wide, flattened, type 4; segment 1 corneous, small, convex; segment 2 larger than segment 1, narrow, transverse, corneous; segments 1–3 present on posterior face. ***Pronotum***: 0.97 × as long as wide. In dorsal view rounded, type 1, sides parallel in basal 1/2, rounded anteriorly; anterior margin with a row of serrations. In lateral view basic, type 0, disc flat, summit at midpoint. Anterior slope with densely spaced, moderate asperities, becoming lower and more strongly transverse towards summit, bearing long, fine, erect hair-like setae, some longer hair-like setae at anterior and lateral margins. Disc shiny, alutaceous with very dense, fine punctures, glabrous. Lateral margins obliquely costate. Base transverse, posterior angles broadly rounded. Mycangial tuft present along basal margin and basal median 1/2 of disc along median line, tuft broad, dense, approximately the width of scutellum. ***Elytra***: 1.31× as long as wide, 1.39× as long as pronotum. Scutellum moderately sized, linguiform, flush with elytra, flat, shiny. Elytral base transverse, edge oblique, humeral angles rounded, parallel-sided in basal 3/4, then broadly rounded to apex. Disc shiny, striae not impressed, seriate; interstriae impunctate, moderately setose, setae semi-erect, hair-like. Declivity sharply distinct from disc, declivital summit armed by large denticles on interstriae 1, 2, 4, and 5 and a very large spine on interstriae 3, its apex incurved; interstrial punctures replaced by uniseriate denticles, each denticle bearing a long, erect hair-like seta equal in length to width of distance between interstriae 1 and 3; six striae present, striae 1 impressed at declivital summit, strial punctures larger, deeper than those of disc. Posterolateral margin carinate, granulate to interstriae 7. ***Legs***: procoxae moderately separated; prosternal coxal piece tall, pointed. Protibiae distinctly triangular, broadest at apical 1/4; posterior face smooth; outer margin of apical 1/3 with five large socketed denticles, their length much longer than basal width; apical mucro prominent, strongly incurved. Meso- and metatibiae flattened, outer margins evenly rounded with nine and 12 small, similarly sized, socketed denticles, their length no longer than basal width, respectively.

##### Distribution.

China (Hong Kong), Thailand, Vietnam.

##### Host plants.

Unknown.

##### Etymology.

L. *spinifer* = thorn-bearing. In reference to the spines on the declivity which are atypical for the genus. A noun in apposition.

**Figure 97.**
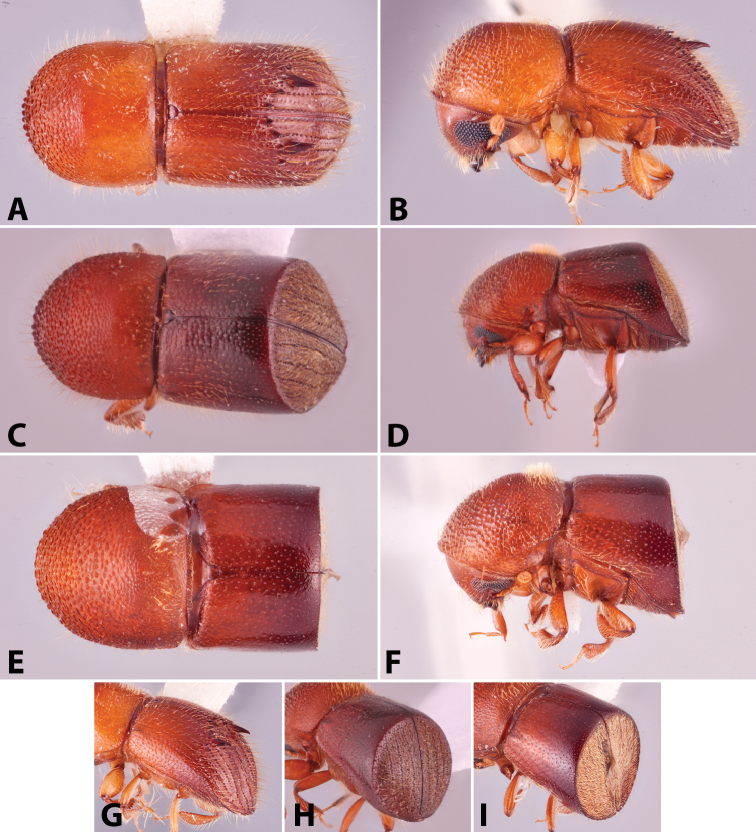
Dorsal, lateral and declivital view of *Xylosandrus spinifer* holotype, 3.3 mm (**A, B, G**), *X. subsimiliformis* holotype, 2.45–3.0 mm (**C, D, H**), and *X. subsimilis*, 2.5–2.9 mm (**E, F, I**).

#### Xylosandrus subsimiliformis

Taxon classificationAnimaliaColeopteraCurculionidae

(Eggers, 1939)

Fig. 97C, D, H

Xyleborus subsimiliformis Eggers, 1939a: 11.
Xylosandrus subsimiliformis (Eggers): Wood and Bright 1992: 800.

##### Type material.

***Holotype*** (NHRS).

##### New records.

Vietnam: Lao Cai, Hoang Lien N.P., 22.35, 103.77, 1500–2000 m, 20.v.2019, VN185, S.M. Smith, A.I. Cognato, ex 1–2 cm branch (MSUC, 37).

##### Diagnosis.

2.45–3.0 mm long (mean = 2.6 mm; n = 5); 1.96–2.14× as long as wide. This species is distinguished by its large size; elytral disc flat, longer than declivity; declivital face steep, abruptly separated from disc; elytra obliquely truncate; posterolateral margins of elytra carinate to interstriae 7; declivital face with four apparent granulate striae (striae 5 short, converging with striae 4 forming a loop); declivital face convex; declivital striae and interstriae setose, setae recumbent, very fine, hair-like, equal to the width of an interstria; interstriae granulate, granules multiseriate, confused; with a uniseriate row of erect hair-like setae equal in length to the width of an interstria; strial granules 2× larger than those of interstriae; pronotum longer than wide, from dorsal view conical frontally (type 6) and lateral view taller (type 2), summit at basal 1/4, basal 1/4 shagreened, dull, densely punctate; and broad, dense mycangial tuft on the pronotal base.

##### Similar species.

*Xylosandrus beesoni*, *X. brevis*, *X. jaintianus*, *X. subsimilis*.

##### Distribution.

China (Yunnan), Myanmar, Vietnam*.

##### Host plants.

Unknown.

##### Remarks.

This species was collected in great abundance by SMS and AIC in Lao Cai province, Vietnam. In nearly all collecting events the species was found in small branches (1–4 cm in diameter) that were dry and often exposed to full sun, an unusual feeding habit, as most other xyleborines are unable to thrive under these conditions. Records from Thailand (Dole and Cognato 2010; Beaver et al. 2014) should be referred to *X. beesoni*.

#### Xylosandrus subsimilis

Taxon classificationAnimaliaColeopteraCurculionidae

(Eggers, 1930)

Fig. 97E, F, I

Xyleborus subsimilis Eggers, 1930: 186.
Xylosandrus subsimilis (Eggers): Wood and Bright 1992: 800.

##### Type material.

***Holotype*** (FRI), ***paratype*** (NHMW, 1).

##### New records.

India: Arunachal Pradesh, Hunli vicinity, 28°19'32"N, 95°57'31"E, 1300 ± 100 m, 26.v–1.vi.2012, L. Dembický (ZFMK, 1).

##### Diagnosis.

2.5–2.9 mm long (mean = 2.64 mm; n = 5); 1.79–2.0× as long as wide. This species is distinguished by its moderate size; elytral disc flat, longer than declivity; declivital face steep, abruptly separated from disc; elytra truncate; posterolateral margins of elytra carinate to interstriae 7; declivital face with four apparent granulate striae (striae 5 short, converging with striae 4 forming a loop); declivital face flattened, depressed below declivital margins; declivital striae and interstriae setose, setae recumbent, thick, less than 1/2 width of an interstria; interstriae granulate, granules multiseriate, confused strial granules at least 2× larger than those of interstriae; pronotum longer than wide, from dorsal view conical frontally (type 6) and lateral view taller (type 2), summit at basal 1/4, basal 1/4 shagreened, dull, densely punctate; and broad, dense mycangial tuft on the pronotal base.

##### Similar species.

*Xylosandrus brevis*, *X. jaintianus*, *X. subsimiliformis*.

##### Distribution.

China (Hainan, Yunnan), India (Arunachal Pradesh*, Assam, Meghalaya, West Bengal), Laos, Myanmar, Thailand.

##### Host plants.

Recorded from five different families (Maiti and Saha 2004; Dole and Cognato 2010), and presumably polyphagous.

## Footnotes

Modified from Cognato et al. 2020a.

Modified from Beaver 2010.

Modified from Cognato et al. 2020a

## References

[B1] Abreu RLS Fonseca CRV Guerrero JCH Paula EVCM (2001) Preferência de vôo de nove espécies da família Scolytidae (Insecta: Coleoptera) na Amazônia Central. Acta Amazonica 31: 61–68. 10.1590/1809-43922001311068

[B2] Alonso-Zarazaga MA Lyal CHC (2009) A catalogue of family and genus group names in Scolytinae and Platypodinae with nomenclatural remarks (Coleoptera: Curculionidae). Zootaxa 2258: 1–134. 10.11646/zootaxa.2258.1.1

[B3] Anderson WH Anderson DM (1971) Type specimens in the Hans Eggers collection of scolytid beetles (Coleoptera). Smithsonian Contributions to Zoology 94: 1–38. 10.5479/si.00810282.94

[B4] Atkinson TH (2018) Bark and ambrosia beetles. http://www.barkbeetles.info

[B5] Atkinson TH Rabaglia RJ Bright DE (1990) Newly detected exotic species of *Xyleborus* (Coleoptera: Scolytidae) with a revised key to species in eastern North America. The Canadian Entomologist 122: 92–104. 10.4039/Ent12293-1

[B6] Atkinson TH Carrillo D Duncan RE Peña JE (2013) Occurrence of *Xyleborus bispinatus* (Coleoptera: Curculionidae: Scolytinae) Eichhoff in southern Florida. Zootaxa 3669: 96–100. 10.11646/zootaxa.3669.1.10 26312324

[B7] Bateman C Kendra PE Rabaglia R Hulcr J (2015) Fungal symbionts in three exotic ambrosia beetles, *Xylosandrus amputatus*, *Xyleborinus andrewesi*, and *Dryoxylon onoharaense* (Coleoptera: Curculionidae: Scolytinae: Xyleborini) in Florida. Symbiosis 66: 141–148. 10.1007/s13199-015-0353-z

[B8] Beaver RA (1987) The bark and ambrosia beetles (Coleoptera: Scolytidae and Platypodidae) of Tonga. New Zealand Entomologist 9: 64–70. 10.1080/00779962.1987.9722496

[B9] Beaver RA (1988) Biological studies on ambrosia beetles of the Seychelles (Col, Scolytidae and Platypodidae). Journal of Applied Entomology 105: 62–73. 10.1111/j.1439-0418.1988.tb00162.x

[B10] Beaver RA (1989) Insect-fungus relationships in the bark and ambrosia beetles. In: Wilding N Collins NM Hammond PM Webber JF (Eds) Insect–Fungus Interactions.Academic Press, London, 121–143. 10.1016/B978-0-12-751800-8.50011-2

[B11] Beaver RA (1990) New records and new species of bark and ambrosia beetles from Thailand (Coleoptera: Scolytidae and Platypodidae). Deutsche Entomologische Zeitschrift 37: 279–284. 10.1002/mmnd.19900370408

[B12] Beaver RA (1991) New synonymy and taxonomic changes in Pacific Scolytidae (Coleoptera). Annalen des Naturhistorisches Museums in Wien 92B: 87–97.

[B13] Beaver RA (1995a) New synonymy and taxonomic changes in Oriental and Australasian Scolytidae and Platypodidae (Insecta: Coleoptera). Annalen des Naturhistorischen Museums in Wien 97B: 197–204.

[B14] Beaver RA (1995b) Additions and corrections to the bark and ambrosia beetle fauna of Fiji (Coleoptera: Scolytidae). South Pacific Journal of Natural Science 14: 11–26.

[B15] Beaver RA (1998) New synonymy, new combinations and taxonomic notes on Scolytidae and Platypodidae (Insecta: Coleoptera). Annalen des Naturhistorischen Museums in Wien, Series B, Botanik und Zoologie 100B: 179–192.

[B16] Beaver RA (2005) A remarkable new species of *Cyclorhipidion* Hagedorn, and new records of bark and ambrosia beetles from Gabon (Coleoptera: Curculionidae, Scolytinae and Platypodinae). The Entomologist’s Monthly Magazine 141: 113–119.

[B17] Beaver RA (2010) A review of the genus *Hadrodemius* Wood, with new synonymy and a key to species (Coleoptera: Curculionidae: Scolytinae). Zootaxa 2444: 51–57. 10.11646/zootaxa.2444.1.3 32230686

[B18] Beaver RA (2011) New synonymy and taxonomic changes in bark and ambrosia beetles (Coleoptera: Curculionidae: Scolytinae, Platypodinae). Koleopterologische Rundschau 81: 277–289.

[B19] Beaver RA Browne FG (1975) The Scolytidae and Platypodidae (Coleoptera) of Thailand, a checklist with biological and zoogeographical notes. Oriental Insects 9: 283–311. 10.1080/00305316.1975.10434499

[B20] Beaver RA Browne FG (1979) The Scolytidae and Platypodidae (Coleoptera) of Penang, Malaysia. Oriental Insects 12(1978): 575–624. 10.1080/00305316.1978.10432538

[B21] Beaver RA Hulcr J (2008) A review of the ambrosia beetle genus *Cryptoxyleborus* Schedl (Coleoptera, Curculionidae: Scolytinae). The Coleopterists Bulletin 62: 133–153. 10.1649/1026.1

[B22] Beaver RA Liu L-Y (2010) An annotated synopsis of Taiwanese bark and ambrosia beetles, with new synonymy, new combinations and new records (Coleoptera: Curculionidae: Scolytinae). Zootaxa 2602: 1–47. 10.11646/zootaxa.2602.1.1

[B23] Beaver RA Liu L-Y (2018) A synopsis of the bark and ambrosia beetles of Nepal with a key to the genera (Insecta: Coleoptera: Curculionidae: Platypodinae and Scolytinae). In: Hartmann M Barclay MVL Weipert J (Eds) Biodiversität und Naturausstattung im Himalaya VI.Verein der Freunde und Förderer des Naturkundemuseum Erfurt, Erfurt, 521–553.

[B24] Beaver RA Löyttyniemi K (1991) Annual flight patterns and diversity of bark and ambrosia beetles (Col., Scolytidae and Platypodidae) attracted to bait logs in Zambia. Journal of Applied Entomology 112: 505–511. 10.1111/j.1439-0418.1991.tb01084.x

[B25] Beaver RA Kajimura H Goto H (2008) Taxonomic changes and new records of Japanese bark and ambrosia beetles (Coleoptera, Curculionidae, Scolytinae). Elytra, Tokyo 36: 231–239.

[B26] Beaver RA Sittichaya W Liu L-Y (2014) A synopsis of the scolytine ambrosia beetles of Thailand (Coleoptera: Curculionidae: Scolytinae). Zootaxa 3875: 1–82. 10.11646/zootaxa.3875.1.1 25544343

[B27] Beaver RA Sittichaya W Liu L-Y (2019) A review of the genus *Immanus* Hulcr & Cognato with a key to species (Coleoptera: Curculionidae: Scolytinae: Xyleborini). Zootaxa 4585: 378–386. 10.11646/zootaxa.4585.2.11 31716176

[B28] Bedel LEM (1888) Faune des Coléoptères du Bassin de la Seine (Vol. VI). Rhynchophora. Annales de la Société Entomologique de France (6) 7, Publication Hors Série, 385–444.

[B29] Beeson CFC (1915) Notes on some Indian forest beetles. Indian Forester 16: 294–299.

[B30] Beeson CFC (1929) Platypodidae and Scolytidae Insects of Samoa, Part 4, Coleoptera, Fascicle 4: 217–248.

[B31] Beeson CFC (1930) The biology of the genus *Xyleborus*, with more new species. Indian Forest Records 14: 209–272.

[B32] Beeson CFC (1935a) Platypodidae and Scolytidae of the Society Islands. Bulletin of the Bernice P. Bishop Museum 142: 115–121.

[B33] Beeson CFC (1935b) Scolytidae of the Marquesas. Bulletin of the Bernice P. Bishop Museum 142: 101–114.

[B34] Beeson CFC (1940) Scolytidae and Platypodidae of the Mangarevan Expedition. Occasional Papers of the Bernice P. Bishop Museum 15: 191–203.

[B35] Beeson CFC (1961) The Ecology and Control of the Forest Insects of India and the Neighbouring Countries. (2^nd^ edn.). Government of India, New Delhi, 767 pp.

[B36] Biedermann PHW (2010) Observations on sex ratio and behavior of males in *Xyleborinus saxesenii* Ratzeburg (Scolytinae, Coleoptera). In: Cognato AI Knížek M (Eds) Sixty years of discovering scolytine and platypodine diversity: A tribute to Stephen L. Wood.ZooKeys 56: 253–267. 10.3897/zookeys.56.530 PMC3088316 21594184

[B37] Biedermann PHW Taborsky M (2011) Larval helpers and age polyethism in ambrosia beetles. Proceedings of the National Academy of Science USA 108: 17064–17069. 10.1073/pnas.1107758108 PMC3193236 21969580

[B38] Biedermann PHW Klepzig KR Taborsky M (2009) Fungus cultivation by ambrosia beetles: behavior and laboratory breeding success in three Xyleborine species. Environmental Entomology 38: 1096–1105. 10.1603/022.038.0417 19689888

[B39] Biedermann PHW Klepzig KD Taborsky M (2011) Costs of delayed dispersal and alloparental care in the fungus-cultivating ambrosia beetle *Xyleborus affinis* Eichhoff (Scolytinae: Curculionidae). Behavioral Ecology and Sociobiology 65: 1753–1761. 10.1007/s00265-011-1183-5

[B40] Blackburn T (1885) [new taxa] In: Blackburn T Sharp D (Eds) Memoirs on the Coleoptera of the Hawaiian Islands.The Scientific Transactions of the Royal Dublin Society, Series 2, 3, 119–289. [300, pls. IV, V.]

[B41] Blandford WFH (1894a) Notes on Scolytidae and their food-plants. Insect Life 6: 260–265.

[B42] Blandford WFH (1894b) The Rhynchophorous Coleoptera of Japan. Part III. Scolytidae. Transactions of the Entomological Society of London 1894: 53–141.

[B43] Blandford WFH (1894c) Supplementary notes on the Scolytidae of Japan, with a list of species. Transactions of the Entomological Society of London 1894: 575–580. 10.1111/j.1365-2311.1894.tb02101.x

[B44] Blandford WFH (1895) A list of the Scolytidae collected in Ceylon by Mr. George Lewis, with descriptions of new species. Annals and Magazine of Natural History, series 6, 15: 315–328. 10.1080/00222939508677888

[B45] Blandford WFH (1896a) Contributions à la faune indo-chinoise 16^e^ memoire. Annales de la Société Entomologique de France 65: 19–22.

[B46] Blandford WFH (1896b) Descriptions of new Scolytidae from the Indo-Malayan and Austro-Malayan regions. Transactions of the Entomological Society of London 1896: 191–228. 10.1111/j.1365-2311.1896.tb00962.x

[B47] Blandford WFH (1898) On some Oriental Scolytidae of economic importance, with descriptions of five new species. Transactions of the Entomological Society of London 1898: 423–430. 10.1111/j.1365-2311.1898.tb03298.x

[B48] Bohemann CH (1859) Coleoptera. Species nova descripsit. [pp. 1–112, pl. I.] In: Kongliga Svenska Fregatten Eugenies Resa omkring jorden under befall af C. A. Virgin, åren 1851–1853. Vetenskapliga iakttagelser på H. M. Konung Oscar Den Förstes befallning. K. Svenska Vetenskaps Akademien. Andra Delen. Zoologi. 1. Insecta. P. A. Norstedt & Söner, Almquist et Wiksell, Uppsala et Stockholm. 1858–1868, 614 pp. [9 pls.]

[B49] Boieldieu M (1859) Descriptions d’espèces nouvelles de coléoptères. Annales de la Société Entomologique de France, series 3, 7: 461–482. [pl. 8.]

[B50] Boland JM (2016) The impact of an invasive ambrosia beetle on the riparian habitats of the Tijuana River Valley, California. Peer Journal 4: e2141. 10.7717/peerj.2141 PMC4924130 27366644

[B51] Bousquet Y (2018) The dating of the fourth volume of Guillaume-Antoine Olivier’s “Entomologie, ou histoire naturelle des insectes”. ZooKeys 734: 137–148. 10.3897/zookeys.734.22901 PMC5904342 29674858

[B52] Brader L (1964) Étude de la relation entre le scolyte des rameaux du caféier, *Xyleborus compactus* Eichh (*X morstatti* Hag) et sa plante-hôte. Mededelingen van de Landbouwhogeschool Wageningen 64: 1–109.

[B53] Brar GS Capinera JL McLean S Kendra PE Ploetz RC Peña JE (2012) Effect of trap size, trap height, and age of lure on sampling *Xyleborus glabratus* (Coleoptera: Curculionidae: Scolytinae) and its flight periodicity and seasonality. Florida Entomologist 95: 1003–1011. 10.1653/024.095.0428

[B54] Brar GS Capinera JL Kendra PE McLean S Peña JE (2013) Life cycle, development and culture of *Xyleborus glabratus* (Coleoptera: Curculionidae: Scolytinae). Florida Entomologist 96: 1158–1167. 10.1653/024.096.0357

[B55] Bright DE (1968) Review of the tribe Xyleborini in America north of Mexico (Coleoptera: Scolytidae). The Canadian Entomologist 100: 1288–1323. 10.4039/Ent1001288-12

[B56] Bright DE (1972) The Scolytidae and Platypodidae of Jamaica (Coleoptera). Bulletin of Institute of Jamaica, Science Series 21: 1–108.

[B57] Bright DE (1985) Studies on West Indian Scolytidae (Coleoptera), 3: Checklist of Scolytidae of the West Indies with descriptions of new species and taxonomic notes. Entomologischen Arbeit aus dem Museum G. Frey 33–34: 169–188.

[B58] Bright DE (2014) A catalog of Scolytidae and Platypodidae (Coleoptera), Supplement 3 (2000–2010), with notes on subfamily and tribal reclassifications. Insecta Mundi 0356: 1–336.

[B59] Bright DE (2019) A taxonomic monograph of the bark and ambrosia beetles of the West Indies (Coleoptera: Curculionoidea: Scolytidae). Studies on West Indian Scolytidae (Coleoptera) 7. Occasional Papers of the Florida State Collection of Arthropods 12: 1–491.

[B60] Bright DE Rabaglia RJ (1999) *Dryoxylon*, a new genus for *Xyleborus onoharaensis* Murayama, recently established in the Southeastern United States (Coleoptera: Scolytidae). The Coleopterists Bulletin 53: 333–337.

[B61] Bright DE Skidmore RE (1997) A Catalog of Scolytidae and Platypodidae (Coleoptera), Supplement 1 (1990–1994). NRC Research Press, Ottawa, 368 pp.

[B62] Bright DE Skidmore RE (2002) A Catalog of Scolytidae and Platypodidae (Coleoptera), Supplement 2 (1995–1999). NRC Research Press, Ottawa, 523 pp.

[B63] Broun T (1904) Descriptions of new genera and species of New Zealand Coleoptera (Concluded). The Annals and Magazine of Natural History, series 7, 14: 105–127. 10.1080/03745480409442977

[B64] Browne FG (1949) Notes on Malayan Scolytoidea (Coleoptera) with descriptions of new species. The Annals and Magazine of Natural History, series 12, 1: 892–912. 10.1080/00222934808653953

[B65] Browne FG (1950) New Scolytidae and Platypodidae (Coleoptera) from Malaya. The Annals and Magazine of Natural History, series 12, 3: 641–650. 10.1080/00222935008654090

[B66] Browne FG (1955) Synonymy and descriptions of some Oriental Scolytidae and Platypodidae (Coleoptera). Sarawak Museum Journal 6: 343–373.

[B67] Browne FG (1958) New species of *Sclytomimus* [sic] and *Webbia* (Scolytidae) from Borneo and Malaya. Sarawak Museum Journal 8: 487–497.

[B68] Browne FG (1959) Appendix. In: Kalshoven LGE (Ed.) New cases of synonymy in Indomalayan scolytids.Entomologische Berichten 19: 1–97.

[B69] Browne FG (1961a) Borer beetles from Bako National Park (Sarawak). Sarawak Museum Journal 10: 300–318.

[B70] Browne FG (1961b) The biology of Malayan Scolytidae and Platypodidae. Malayan Forest Records 22: 1–255.

[B71] Browne FG (1961c) The generic characters, habits and taxonomic status of *Premnobius* Eichh. (Coleopt., Scolytidae). Report of the West African Timber Borer Research Unit 4: 45–51.

[B72] Browne FG (1962) Some Scolytidae and Platypodidae (Coleoptera) from the Oriental Region. Philippine Journal of Science 89: 201–220.

[B73] Browne FG (1963) Taxonomic notes on Scolytidae (Coleoptera). Entomologische Berichten 23: 53–59.

[B74] Browne FG (1965) On some Scolytidae and Platypodidae (Coleoptera), mainly from Africa and the Oriental region. Zoologische Mededelingen 40: 187–209.

[B75] Browne FG (1966) Some Platypodidae and Scolytidae (Coleoptera) from the Philippine, Bismarck and Solomon islands. Entomologiske Meddelelser 34: 233–257.

[B76] Browne FG (1968a) Pests and Diseases of Forest Plantation Trees: An Annotated List of the Principal Species Occurring in the British Commonwealth. Clarendon Press, Oxford, 1330 pp.

[B77] Browne FG (1968b) A collection of Scolytidae and Platypodidae (Coleoptera) from Vietnam. The Entomologist’s Monthly Magazine 104: 133–134.

[B78] Browne FG (1972) Some oriental Scolytidae and Platypodidae (Coleoptera). Oriental Insects 6: 19–32. 10.1080/00305316.1972.10434049

[B79] Browne FG (1974) A summary of the scolytid fauna (Coleoptera) of Fiji, with some new species. Commonwealth Forestry Review 53: 63–71.

[B80] Browne FG (1980a) Bark beetles and ambrosia beetles (Coleoptera, Scolytidae and Platypodidae) intercepted at Japanese ports, with descriptions of new species, I. Kontyû 48: 370–379.

[B81] Browne FG (1980b) Bark beetles and ambrosia beetles (Coleoptera, Scolytidae and Platypodidae) intercepted at Japanese ports, with descriptions of new species, II. Kontyû 48: 380–389.

[B82] Browne FG (1981a) Bark beetles and ambrosia beetles (Coleoptera, Scolytidae and Platypodidae) intercepted at Japanese ports, with descriptions of new species, V. Kontyû 49: 125–136.

[B83] Browne FG (1981b) Bark beetles and ambrosia beetles (Coleoptera, Scolytidae and Platypodidae) intercepted at Japanese ports, with descriptions of new species, VI. Kontyû 49: 597–606.

[B84] Browne FG (1983a) Bark beetles and ambrosia beetles (Coleoptera, Scolytidae and Platypodidae) intercepted at Japanese ports, with descriptions of new species, VII. Kontyû 51: 554–572.

[B85] Browne FG (1983b) Three new species of Scolytidae (Coleoptera) from southern China. The Entomologist’s Monthly Magazine 119: 33–34.

[B86] Browne FG (1984) More new species of Scolytidae (Coleoptera) from Papua New Guinea. South Pacific Journal of Natural Science 6: 86–102.

[B87] Browne FG (1986) Bark beetles and ambrosia beetles (Coleoptera, Scolytidae and Platypodidae) intercepted at Japanese ports, with descriptions of new species, XIII. Kontyû 54: 89–99.

[B88] Bucini D Balestra GM Pucci C Paparatti B (2005) Bio-ethology of *Anisandrus dispar* F. and its possible involvement in dieback (Moria) diseases of hazelnut (*Corylus avellana* L.) plants in central Italy. Acta Horticulturae 686: 435–444. 10.17660/ActaHortic.2005.686.60

[B89] Bussler H Immler T (2007) Neue Borkenkäferarten in Bayern. Forstschutz Aktuell 38: 5–8.

[B90] Carlton C Bayless V (2011) A case of *Cnestus mutilatus* (Blandford) (Curculionidae: Scolytinae: Xyleborini) females damaging plastic fuel storage containers in Louisiana, U.S.A. The Coleopterists Bulletin 65: 290–291. 10.1649/072.065.0308

[B91] Carrillo D Cruz LF Kendra PE Narvaez TI Montgomery WS Monterroso A De Grave C Cooperband MF (2016) Distribution, pest status and fungal associates of Euwallacea nr. fornicatus in Florida avocado groves. Insects 7: 1–55. 10.3390/insects7040055 PMC5198203 27754408

[B92] Chararas C (1962) Étude Biologique des Scolytides des Conifères. Encyclopédie Entomologique, Sér. A, 38. Lechevalier, Paris, 556 pp.

[B93] Chen Y Dallara PL Nelson LJ Coleman TW Hishinuma SM Carrillo D Seybold SJ (2016) Comparative morphometric and chemical analyses of phenotypes of two invasive ambrosia beetles (*Euwallacea* spp.) in the United States of America. Insect Science 24: 647–662. 10.1111/1744-7917.12329 26931091

[B94] Choo HY (1983) Taxonomic studies on the Platypodidae and Scolytidae (Coleoptera) from Korea. Doctoral Thesis. Seoul National University, Seoul, 128 pp.

[B95] Choo HY Woo KS (1985) A list of Korean bark and ambrosia beetles, and their host plants. Korean Journal of Plant Protection 24: 163–167.

[B96] Cognato AI Olson RO Rabaglia RJ (2011) An Asian ambrosia beetle, *Xylosandrus amputatus* (Blandford) (Curculionidae: Scolytinae: Xyleborini), discovered in Florida, U.S.A. The Coleopterists Bulletin 65: 43–45. 10.1649/0010-065X-65.1.43

[B97] Cognato AI Rabaglia RJ Vandenberg NJ (2013) Another Asian ambrosia beetle, *Xyleborinus artestriatus* (Eichhoff 1878) (Coleoptera: Curculionidae: Scolytinae: Xyleborini), newly detected in North America. The Pan-Pacific Entomologist 89: 27–31. 10.3956/2012-53.1

[B98] Cognato AI Hoebeke ER Kajimura H Smith SM (2015) History of the exotic ambrosia beetles *Euwallacea interjectus* and *Euwallacea validus* (Coleoptera: Curculionidae: Xyleborini) in the United States. Journal of Economic Entomology 108: 1129–1135. 10.1093/jee/tov073 26470238

[B99] Cognato AI Jordal BH Rubinoff D (2018) Ancient ‘Wanderlust’ leads to diversification of endemic Hawaian *Xyleborus* species (Coleoptera: Curculionidae: Scolytinae). Insect Systematics and Diversity 2(3): 1–9. 10.1093/isd/ixy005

[B100] Cognato AI Smith SM Li Y Pham T-H Hulcr J (2019) Genetic variability among native *Xyleborus glabratus* Eichhoff populations and the description of two related species. Journal of Economic Entomology 112: 1274–1284. 10.1093/jee/toz026 30785204

[B101] Cognato AI Smith SM Beaver RA (2020a) Two new genera of Oriental xyleborine ambrosia beetles (Coleoptera, Curculionidae: Scolytinae). Zootaxa 4722: 540–554. 10.11646/zootaxa.4722.6.2 32230598

[B102] Cognato AI Sari G Smith SM Beaver RA Li Y Hulcr J Jordal BH Kajimura H Lin C-S Pham TH Singh S Sittichaya W (2020b) The essential role of taxonomic expertise in the creation of DNA databases for the identification and delimitation of Southeast Asian ambrosia beetle species (Coleoptera: Curculionidae: Scolytinae: Xyleborini). Frontiers in Ecology and Evolution 8. 10.3389/fevo.2020.00027

[B103] Coleman TW Poloni AL Chen Y Thu PQ Li Q Sun J Rabaglia RJ Man G Seybold SJ (2019) Hardwood injury and mortality associated with two shot hole borers, *Euwallacea* spp., in the invaded region of southern California, USA, and the native region of Southeast Asia. Annals of Forest Science 76: 1–61. 10.1007/s13595-019-0847-6

[B104] Cooperband MF Stouthamer R Carillo D Eskalen A Thibault T Cossé AA Castrillo LA Vandenberg JD Rugman-Jones PF (2016) Biology of two members of the *Euwallacea fornicatus* species complex (Coleoptera: Curculionidae: Scolytinae), recently invasive in the U.S.A., reared on an ambrosia beetle artificial diet. Agricultural and Forest Entomology 18: 223–237. 10.1111/afe.12155

[B105] Coyle DR Booth DC Wallace MS (2005) Ambrosia beetle (Coleoptera: Scolytidae) species, flight, and attack on living eastern cottonwood trees. Journal of Economic Entomology 98: 2049–2057. 10.1603/0022-0493-98.6.2049 16539132

[B106] Davis MA Dute RR (1997) Fungal associates of the Asian ambrosia beetle, *Xylosandrus crassiusculus*. Southern Nursery Association Research Conference 42: 106–112.

[B107] Dodelin B (2018) Espèce invasive nouveau pour la faune de France Scolyte *Cyclorhipidion fukiense* installé en Europe. https://entomodata.wordpress.com/2018/04/24/cyclorhipidion-fukiense-installe-en-europe

[B108] Dole SA Beaver RA (2008) A review of the Australian species of *Xylosandrus* Reitter (Coleoptera: Curculionidae: Scolytinae). The Coleopterists Bulletin 62: 481–492. 10.1649/1108.1

[B109] Dole SA Cognato AI (2010) Revision of *Xylosandrus* Reitter (Curculionidae: Scolytinae). Proceedings of the California of Science 61: 451–545.

[B110] Drake CJ (1921) A new ambrosia beetle from the Adirondacks: notes on the work of *Xyloterinus politus* Say. Ohio Journal of Science 21: 201–205.

[B111] Dugès E (1888) Métamorphoses de quelques Coléoptères du Mexique. Annales de la Société entomologique de Belgique 31: 137–147. [2 pls.]

[B112] Egger A (1973) Beiträge zur Biologie und Bekämpfung von Xyleborus (Anisandrus) dispar F. und *X. saxeseni* Ratz (Col, Scolytidae). Anzeiger für Schädlingskunde Pflanzen- und Umweltschutz 46: 183–186. 10.1007/BF01991812

[B113] Eggers H (1920) 60 neue Borkenkäfer (Ipidae) aus Afrika, nebst zehn neuen Gattungen, zwei Abarten. (Schluss). Entomologische Blätter 16: 33–45.

[B114] Eggers H (1922) Seltene und neue paläarktische Borkenkäfer, III. Entomologische Blätter 18: 12–18.

[B115] Eggers H (1923) Neue indomalayische Borkenkäfer (Ipidae). Zoologische Mededeelingen 7: 129–220.

[B116] Eggers H (1925) Ipidae aus Birma. Sborník Entomologického Oddělení Národního Musea v Praze 3: 151–160.

[B117] Eggers H (1926) Japanische Borkenkäfer, I. Entomologische Blätter 22: 145–148.

[B118] Eggers H (1927a) Neue indomalayische Borkenkäfer (Ipidae). I. Nachtrag. Treubia 9: 390–408.

[B119] Eggers H (1927b) New Indo-Malayische Borkenkäfer (Ipidae), II. Nachtrag. Philippine Journal of Science 33: 67–108.

[B120] Eggers H (1929) Zur Synonymie der Borkenkäfer (Ipidae, Col) I. Wiener Entomologische Zeitung 46: 41–55.

[B121] Eggers H (1930) Neue *Xyleborus*-Arten (Col. Scolytidae) aus Indien. Indian Forest Records, Entomology Series 14: 177–208.

[B122] Eggers H (1932) Neue Borkenkafer (Ipidae, Col.) aus Africa (Nachtrag V.). Revue de Zoologie et de Botanique Africaines 22: 191–304.

[B123] Eggers H (1933a) Borkenkäfer (Ipidae, Col) aus Südamerika, VI. Material des Muséum Paris aus Franz. Guayana und Venezuela. Travaux du Laboratoire d’Entomologie, Muséum National d’Histoire Naturelle, Mémoires Originaux 1: 1–37.

[B124] Eggers H (1933b) Zur paläarktischen Borkenkäferfauna, I. Entomologische Blätter 29: 49–56.

[B125] Eggers H (1934a) Borkenkäfer (Ipidae, Col) aus Sudamerika, VII. Entomologische Blätter 30: 78–84.

[B126] Eggers H (1934b) Zur Synonymie der Borkenkäfer (Ipidae, Col) IV. Entomologisches Nachrichtenblatt 8: 25–29.

[B127] Eggers H (1935) Borkenkäfer aus Südamerika (Ipidae, Col) (Fortsetzung). VII. Vergessene und neue Gattungen (1. Teil). Revista de Entomologia, Rio de Janeiro 5: 153–159.

[B128] Eggers H (1936a) Neue Borkenkäfer (Scolytidae, Col) aus Indien. The Annals and Magazine of Natural History, series 10, 17: 626–636. 10.1080/00222933608655163

[B129] Eggers H (1936b) Neue indomalayische Borkenkäfer (Ipidae) III Nachtrag. Tijdschrift voor Entomologie 79: 77–91.

[B130] Eggers H (1937) Zur paläarktischen Borkenkäferfauna, IV. Entomologische Blätter 33: 334–335.

[B131] Eggers H (1939a) Entomological results from the Swedish expedition 1934 to Burma and British India. Coleoptera: Ipidae, gesammelt von René Malaise. Arkiv för Zoologi 31A: 1–14.

[B132] Eggers H (1939b) Japanische Borkenkäfer, II. Arbeiten über Morphologische und Taxonomische Entomologie aus Berlin-Dahlem 6: 114–123.

[B133] Eggers H (1940) Neue indomalayische Borkenkäfer (Ipidae) III Nachtrag (Forstsetzung). Tijdschrift voor Entomologie 83: 132–154.

[B134] Eggers H (1941a) Borkenkäfer aus Südamerika. (Coleoptera: Ipidae). IX. Insel Guadeloupe. Arbeiten über Morphologische und Taxonomische Entomologie aus Berlin-Dahlem 8: 99–109.

[B135] Eggers H (1941b) Neue Borkenkäfer (Ipidae, Col) aus China. Entomologische Blätter 37: 222–226.

[B136] Eggers H (1942) Zur palaearktischen Borkenkäferfauna (Coleoptera: Ipidae). VIII. Borkenkäfer aus dem asiatischen Russland. Arbeiten über Morphologische und Taxonomische Entomologie aus Berlin-Dahlem 9: 27–36.

[B137] Eggers H (1943) Ipidae (Scolytidae) (ColeopteraPhytophaga). Exploration du Parc National Albert, I. Mission C. F. De Witte 1933–1935, Fascicle 43: 63–68.

[B138] Eggers H (1944) Zur paläarktischen Borkenkäferfauna (Coleoptera, Ipidae) X. Entomologische Blätter 40: 140–143.

[B139] Eichhoff WJ (1864) Ueber die Mundtheile und die Fühlerbildung der europäischen Xylophagi sens strict. Berliner Entomologische Zeitschrift 8: 17–46. 10.1002/mmnd.18640080103

[B140] Eichhoff WJ (1866) Ueber einige Bostrichiden. Berliner Entomologische Zeitschrift 10: 275–278. ttps://10.1002/mmnd.18660100118

[B141] Eichhoff WJ (1868a) Neue amerikanische Borkenkäfer-Gattungen und Arten. Berliner Entomologische Zeitschrift 11: 399–402. 10.1002/mmnd.18670110320

[B142] Eichhoff WJ (1868b) Neue amerikanische Borkenkäfer-Gattung und Arten. Berliner Entomologische Zeitschrift 12: 145–152. 10.1002/mmnd.18680120213

[B143] Eichhoff WJ (1869) Neue exotische *Xyleborus*-Arten. Berliner Entomologische Zeitschrift 12: 273–280. 10.1002/mmnd.18680120214

[B144] Eichhoff WJ (1876a) Tomicides. In: Chapuis F Eichhoff WJ (Eds) Scolytides recueillis au Japon par M. G. Lewis.Annales de la Société Entomologique de Belgique 18: 195–204.

[B145] Eichhoff WJ (1876b) Synonymisches über Tomiciden. Stettiner Entomogische Zeitung 37: 378–379.

[B146] Eichhoff WJ (1877) Japanische Scolytidae. Deutsche Entomologische Zeitschrift 21: 117–128. 10.1002/mmnd.4800210121

[B147] Eichhoff WJ (1878a) Neue oder noch unbeschriebene Tomicinen. Stettiner Entomologische Zeitung 39: 383–392.

[B148] Eichhoff WJ (1878b) Ratio, descriptio, emendatio eorum Tomicinorum qui sunt in Dr medic. Chapuisii et autoris ipsius collectionibus et quos praeterea recognovit scriptor. Mémoires de la Société Royale des Sciences de Liège, Série 2e, 8: 1–531. [pls. I–V.]

[B149] Eichhoff WJ (1880) Description of a new species of the family Scolytidae from Sumatra. Notes from the Leyden Museum 2: 189–190.

[B150] Eichhoff WJ (1886) Zwei neue ost-indische Scolytiden-Gattungen. Notes from the Leyden Museum 8: 24–26.

[B151] Entwhistle PF (1972) Pests of Cocoa. Longman, London, 779 pp.

[B152] Erichson WF (1836) Systematische Auseinandersetzung der Familie der Borkenkäfer (Bostrichidae). Archiv für Naturgeschichte 2: 45–65.

[B153] Erichson WF (1842) Beitrag zur Insecten-Fauna von Vandiemensland, mit besonderer Berücksichtigung der geographischen Verbreitung der Insecten. Archiv für Naturgeschichte 8: 83–287. [2 pls.] 10.5962/bhl.part.21656

[B154] Eskalen A Stouthamer R Lynch SC Rugman-Jones PF Twizeyimana M Gonzalez A Thibault T (2013) Host range of Fusarium dieback and its ambrosia beetle (Coleoptera: Scolytinae) vector in southern California. Plant Disease 97: 938–951. 10.1094/PDIS-11-12-1026-RE 30722538

[B155] Fabricius JC (1775) Systema Entomologiae, sistens Insectorum Classes, Ordines, Genera, Species Adiectis Synonymis, Locis, Descriptionibus, Observationibus. Flensburgi et Lipsiae: Officina Libraria Kortii, 832 pp. 10.5962/bhl.title.36510

[B156] Fabricius JC (1792) Entomologia Systematica Emendata et Aucta. Secundum Classes, Ordines, Genera, Species Adjectis Synonimis, Locis, Observationibus, Descriptionibus. Tomus I. Pars II. Hafniae: C.G. Proft, 538 pp 10.5962/bhl.title.122153

[B157] Fabricius JC (1801) Systema Eleutheratorum Secundum Ordines, Genera, Species; Adiectis Synonimis, Locis, Observationibus, Descriptionibus, Tomus II. Kiliae: Bibliopoli Academici Novi, 687 pp. 10.5962/bhl.title.137098

[B158] Faccoli M (2008) First record of *Xyleborus atratus* Eichhoff from Europe with an illustrated key to the European Xyleborini (Coleoptera: Curculionidae: Scolytinae). Zootaxa 1772: 55–62. 10.11646/zootaxa.1772.1.2

[B159] Faccoli M Frigimelica G Mori N Toffolo EP Vettorazzo M Simonato M (2009) First record of *Ambrosiodmus* (Hopkins, 1915) (Coleoptera, Curculionidae, Scolytinae) in Europe. Zootaxa 2303: 57–60. 10.11646/zootaxa.2303.1.4

[B160] Ferrari JA (1867) Die Forst- und Baumzuchtschädlichen Borkenkäfer (Tomicides Lac.) aus der Familie der Holzverderber (Scolytides Lac.), mit Besonderer Berücksichtigung Vorzüglich der Europäischen Formen, und der Sammlung des k. k. Zoologischen Kabinets in Wien. Carl Gerold’s Sohn, Wien, 96 pp.

[B161] Fischer M (1954) Untersuchungen über den Kleinen Holzbohrer (*Xyleborinus saxeseni* Ratz). Plflanzenschutzberichte 12: 137–180.

[B162] Flechtmann CAH Cognato AI (2011) First report of *Amasa truncata* (Coleoptera: Curculionidae: Scolytinae) in Brazil. The Coleopterists Bulletin 65: 417–421. 10.1649/072.065.0419

[B163] Flechtmann CAH Atkinson TH (2016) First records of *Xylosandrus crassiusculus* (Motschulsky) (Coleoptera: Curculionidae: Scolytinae) from South America, with notes on its distribution and spread in the New World. The Coleopterists Bulletin 70: 79–83. 10.1649/072.070.0109

[B164] Formby JP Krishnan N Riggins JJ (2013) Supercooling in the redbay ambrosia beetle (Coleoptera: Curculionidae). The Florida Entomologist 96: 1530–1540. 10.1653/024.096.0435

[B165] Fraedrich SW Harrington TC Rabaglia RJ Ulyshen MD Mayfield III AE Hanula JL Eickwort JM Miller DR (2008) A fungal symbiont of the redbay ambrosia beetle causes a lethal wilt in redbay and other Lauraceae in the Southeastern United States. Plant Disease 92: 215–224. 10.1094/PDIS-92-2-0215 30769391

[B166] Freeman S Sharon M Maymon M Mendel Z Protasov A Aoki T Eskalen A O’Donnell K (2013) *Fusarium euwallaceae* sp. nov. –a symbiotic fungus of *Euwallacea* sp., an invasive ambrosia beetle in Israel and California. Mycologia 105: 1595–1606. 10.3852/13-066 23928415

[B167] French JR Roeper RA (1975) Studies on the biology of the ambrosia beetle *Xyleborus dispar* (F) (Coleoptera: Scolytidae). Zeitschrift für Angewandte Entomologie 78: 241–247. 10.1111/j.1439-0418.1975.tb04178.x

[B168] Galko J Nikolov C Kimoto T Kunca A Gubka A Vakula J Zúbrik M Ostriboň M (2014) Attraction of ambrosia beetles to ethanol baited traps in a Slovakian oak forest. Biologia 69: 1376–1383. 10.2478/s11756-014-0443-z

[B169] Gallego D Lencina JL Mas H Ceveró J Faccoli M (2017) First record of the granulate ambrosia beetle, *Xylosandrus crassiusculus* (Coleoptera: Curculionidae: Scolytinae), in the Iberian Peninsula. Zootaxa 2743: 431–434. 10.11646/zootaxa.4273.3.7 28610243

[B170] Garonna AP Dole SA Saracino A Mazzoleni S Cristinzio G (2012) First record of the black twig borer *Xylosandrus compactus* (Eichhoff) (Coleoptera: Curculionidae, Scolytinae) from Europe. Zootaxa 3251: 64–68. 10.11646/zootaxa.3251.1.5

[B171] Gohli J Selvarajah T Kirkendall LR Jordal BH (2016) Globally distributed *Xyleborus* species reveal recurrent intercontinental dispersal in a landscape of ancient worldwide distributions. BMC Evolutionary Biology 16: 1–37. 10.1186/s12862-016-0610-7 26877088 PMC4753646

[B172] Gohli J Kirkendall LR Smith SM Cognato AI Hulcr J Jordal BH (2017) Biological factors contributing to bark and ambrosia beetle species diversification. Evolution 71: 1258–1272. 10.1111/evo.13219 28257556

[B173] Gray B Wylie FR (1974) Forest tree and timber insect pests in Papua New Guinea II. Pacific Insects 16: 67–115.

[B174] Gómez D Suárez M Martínez G (2017) *Amasa truncata* (Erichson) (Coleoptera: Curculionidae: Scolytinae): a new exotic ambrosia beetle in Uruguay. The Coleopterists Bulletin 71: 825–826. 10.1111/evo.13219

[B175] Gomez DF Rabaglia RJ Fairbanks KEO Hulcr J (2018a) North American Xyleborini north of Mexico: a review and key to genera and species (Coleoptera, Curculionidae, Scolytinae). ZooKeys 768: 19–68. 10.3897/zookeys.768.24697 PMC6019436 29955211

[B176] Gomez DF Skelton J Steininger MS Stouthamer R Rugman-Jones P Sittichaya W Rabaglia RJ Hulcr J (2018b) Species within the *Euwallacea fornicatus* (Coleoptera: Curculionidae) complex revealed by morphometric and phylogenetic analyses. Insect Systematics and Diversity 2(6): 1–11. 10.1093/isd/ixy018

[B177] Gomez DF Johnson AJ Carton de Grammont P Alfonso-Simonetti J Montaigne J Elizondo AI Muiño BL Ojeda D Vidal J Hulcr J (2020) New records of bark and ambrosia beetles (Coleoptera: Scolytinae) from Cuba with description of a new species. Florida Entomologist 102: 717–724. 10.1653/024.102.0408

[B178] Haack RA Rabaglia RJ (2013) Exotic bark and ambrosia beetles in the USA: potential and current invaders. In: Peña J (Ed.) Potential Invasive Pests of Agricultural Crops.CAB International, Wallingford, 48–74. 10.1079/9781845938291.0048

[B179] Hagedorn M (1904) Enumeratio Scolytidarum e Sikkim et Japan natarum Musei historico-naturalis Parisiorum, quas dominus J. Harmand annis 1890 et 1901 collegit descriptionibus specierum novarum adjectis. Bulletin du Muséum d’Histoire Naturelle 10: 122–126.

[B180] Hagedorn M (1908) Diagnosen bisher unbeschriebener Borkenkäfer. Erste Serie. Deutsche Entomologische Zeitschrift 1908: 369–382. 10.1002/mmnd.48019080310

[B181] Hagedorn M (1909) Diagnosen bisher unbeschriebener Borkenkäfer (Col). Deutsche Entomologische Zeitschrift 1909: 733–746. 10.1002/mmnd.48019090606

[B182] Hagedon M (1910a) Diagnosen bisher unbeschriebener Borkenkäfer (Col). Zweite Serie, zweite Hälfte. Deutsche Entomologische Zeitschrift 1910: 1–13. 10.1002/mmnd.4801910101

[B183] Hagedorn M (1910b) Ipidae. In: Schenkling S (Ed.) Coleopterorum Catalogus Auspiciis et Auxilio. Pars 4. W. Junk, Den Haag, 134 pp 10.1007/978-94-011-9697-0_1

[B184] Hagedorn M (1912a) Ipiden als Kaffeeschädlinge. Entomologische Blätter 8: 33–43.

[B185] Hagedorn M (1912b) Neue Borkenkäfergattungen und Arten aus Afrika (Col.). Deutsche Entomologische Zeitschrift 1912: 351–356. [pls. 6–7.] 10.1002/mmnd.48019120313

[B186] Halbert SE (2011) Entomology Section. Tri-ology 50: 6–7.

[B187] Hanula JL Mayfield III AE Fraedrich SW Rabaglia RJ (2008) Biology and host associations of redbay ambrosia beetle (Coleoptera: Curculionidae: Scolytinae), exotic vector of laurel wilt killing redbay trees in Southeastern United States. Journal of Economic Entomology 101: 1276–1286. 10.1093/jee/101.4.1276 18767737

[B188] Harrington TC Yun HY Lu SS Goto H Aghayeva DN Fraedrich SW (2011) Isolations from the redbay ambrosia beetle, *Xyleborus glabratus*, confirm that the laurel wilt pathogen *Raffaelea lauricola*, originated in Asia. Mycologia 103: 1028–1036. 10.3852/10-417 21471288

[B189] Hey J (2006) On the failure of modern species concepts. Trends in Ecology & Evolution 21: 447–50. 10.1016/j.tree.2006.05.011 16762447

[B190] Hoebeke ER (1991) An Asian ambrosia beetle, *Ambrosiodmus lewisi*, new to North America (Coleoptera: Scolytidae). Proceedings of the Entomological Society of Washington 93: 420–424.

[B191] Hoebeke ER Rabaglia RJ (2007) First reported occurrence of *Xyleborinus alni* (Coleoptera: Curculionidae: Scolytinae) in the eastern United States, with notes on its recognition and tree hosts. Proceedings of the Entomological Society of Washington 109: 240–248.

[B192] Hoebeke ER Rabaglia RJ (2008) *Xyleborus seriatus* Blandford (Coleoptera: Curculionidae: Scolytinae), an Asian ambrosia beetle new to North America. Proceedings of the Entomological Society of Washington 110: 470–476. 10.4289/07-048.1

[B193] Hoebeke ER Rabaglia RJ Knížek M Weaver JS (2018) First records of *Cyclorhipidion fukiense* (Eggers) (Coleoptera: Curculionidae: Scolytinae: Xyleborini), an ambrosia beetle native to Asia, in North America. Zootaxa 4394: 243–250. 10.11646/zootaxa.4394.2.7 29690374

[B194] Hoffman CE (1941) Biological observations of *Xylosandrus germanus* (Bldfd.). Journal of Economic Entomology 34: 38–42. 10.1093/jee/34.1.38

[B195] Hopkins AD (1915a) Classification of the Cryphalinae with Descriptions of new Genera and Species. United States Department of Agriculture, Report No. 99. Government Printing Office, Washington, 75 pp [4 pls.] 10.5962/bhl.title.65905

[B196] Hopkins AD (1915b) Contributions Toward a Monograph of the Scolytid Beetles, Part II. Preliminary Classification of the Superfamily Scolytoidea. United States Department of Agriculture, Technical Series, No. 17. Government Printing Office, Washington, 165–232. [pls. IX–XV.]

[B197] Hosking GP (1973) *Xyleborus saxeseni*, its life history and flight behavior in New Zealand. New Zealand Journal of Forestry Science 3: 37–53.

[B198] Hubbard HG (1897) The ambrosia beetles of the United States. Bulletin of the United States Department of Agriculture, Division of Entomology, new series 7: 9–30.

[B199] Hughes MA Riggins JJ Koch FH Cognato AI Anderson C Formby JR Dreaden TJ Ploetz RC Smith JA (2017) No rest for the laurels: symbiotic invaders cause unprecedented damage to southern USA forests. Biological Invasions 19: 2143–2157. 10.1007/s10530-017-1427-z

[B200] Hulcr J (2010) Taxonomic changes in palaeotropical Xyleborini (Coleoptera: Curculionidae: Scolytinae). ZooKeys 56: 105–119. 10.3897/zookeys.56.520 PMC3088323 21594174

[B201] Hulcr J Cognato AI (2009) Three new genera of Oriental Xyleborini (Coleoptera: Curculionidae: Scolytinae). Zootaxa 2204: 19–36. 10.11646/zootaxa.2204.1.2 32230598

[B202] Hulcr J Cognato AI (2010a) New genera of Palaeotropical Xyleborini (Coleoptera: Curculionidae: Scolytinae) based on congruence between morphological and molecular characters. Zootaxa 2717: 1–33. 10.11646/zootaxa.2717.1.1

[B203] Hulcr J Cognato AI (2010b) Repeated evolution of theft in fungus farming ambrosia beetles. Evolution 64: 3205–3212. 10.1111/j.1558-5646.2010.01055.x 20633043

[B204] Hulcr J Smith S (2010) Xyleborini ambrosia beetles: an identification tool to the world genera. http://itp.lucidcentral.org/id/wbb/xyleborini/index.htm.

[B205] Hulcr J Cognato AI (2013) Xyleborini of New Guinea: A Taxonomic Monograph. Thomas Say Publications in Entomology, Entomological Society of America, Lanham, 176 pp.

[B206] Hulcr J Lou Q-Z (2013) The redbay ambrosia beetle (Coleoptera: Curculionidae) prefers Lauraceae in its native range: records form the Chinese National Insect Collection. Florida Entomologist 96: 1595–1597. 10.1653/024.096.0444

[B207] Hulcr J Stelinski LL (2017) The ambrosia symbiosis: from evolutionary ecology to practical management. Annual Reviews of Entomology 62: 285–303. 10.1146/annurev-ento-031616-035105 27860522

[B208] Hulcr J Dole SA Beaver RA Cognato AI (2007) Cladistic review of generic taxonomic characters in Xyleborini (Coleoptera: Curculionidae: Scolytinae). Systematic Entomology 32: 568–584. 10.1111/j.1365-3113.2007.00386.x

[B209] Hulcr J Mann R Stelinski LL (2011) The scent of a partner: ambrosia beetles are attracted to volatiles from their fungal symbiont. Journal of Chemical Ecology 37: 1374–1377. 10.1007/s10886-011-0046-x 22161224

[B210] Hulcr J Atkinson TH Cognato AI Jordal BH McKenna DD (2015) Morphology, taxonomy, and phylogenetics of bark beetles. In: Vega FE Hofstetter RW (Eds) Bark Beetles.Biology and Ecology of Native and Invasive Species. Academic Press, London, 41–84. 10.1016/B978-0-12-417156-5.00002-2

[B211] Hulcr J Black A Prior K Chen C-Y Li H-F (2017) Studies of ambrosia beetles in their native range help predict invasion impact. Florida Entomologist 100: 257–261. 10.1653/024.100.0219

[B212] Ito M Kajimura H Hamaguchi K Araya K Lakatos F (2008) Genetic structure of Japanese populations of an ambrosia beetle, *Xylosandrus germanus* (Curculionidae: Scolytinae). Entomological Science 11: 375–383. 10.1111/j.1479-8298.2008.00280.x

[B213] Jordal BH (2002) Elongation Factor 1 a resolves the monophyly of the haplodiploid ambrosia beetles Xyleborini (Coleoptera: Curculionidae). Insect Molecular Biology 11: 453–465. 10.1046/j.1365-2583.2002.00354.x 12230544

[B214] Jordal BH Cognato AI (2012) Molecular phylogeny of bark and ambrosia beetles reveals multiple origins of fungus farming during periods of global warming. BMC Evolutionary Biology 12: 133. 10.1186/1471-2148-12-133 PMC3514184 22852794

[B215] Jordal BH Normark BB Farrell BD (2000) Evolutionary radiation of an inbreeding haplodiploid beetle lineage (Curculionidae, Scolytidae). Biological Journal of the Linnean Society 71: 483–499. 10.1111/j.1095-8312.2000.tb01270.x

[B216] Jordal BH Beaver RA Kirkendall LR (2001) Breaking taboos in the tropics: incest promotes colonization by wood-boring beetles. Global Ecology and Biogeography 10: 345–357. 10.1046/j.1466-822X.2001.00242.x

[B217] Kajimura H Hijii N (1992) Dynamics of the fungal symbionts in the gallery system and the mycangia of the ambrosia beetle, *Xylosandrus mutilatus* (Blandford) (Coleoptera: Scolytidae) in relation to its life history. Ecological Research 7: 107–117. 10.1007/BF02348489

[B218] Kajimura H Hijii N (1994) Reproduction and resource utilization of the ambrosia beetle *Xylosandrus mutilatus*, in field and experimental populations. Entomologia Experimentalis et Applicata 71: 121–132. 10.1111/j.1570-7458.1994.tb01778.x

[B219] Kalshoven LGE (1959a) New cases of synonymy in Indomalayan scolytids. Entomologische Berichten, Amsterdam 19: 93–97.

[B220] Kalshoven LGE (1959b) Studies on the biology of Indonesian Scolytoidea 4. Data on the habits of Scolytidae. Second part. Tijdschrift voor Entomologie 102: 135–173. [pls. 15–22.]

[B221] Kalshoven LGE (1960) Two new cases of synonymy in Indomalayan Platypodidae and Scolytidae. Entomologische Berichten, Amsterdam 20: 63–64.

[B222] Kalshoven LGE (1961) A study of the twig borer *Xyleborus morigerus* Blandford, mainly based on observations in Java. Tijdschrift voor Entomologie 104: 93–110.

[B223] Kalshoven LGE (1962) Note on the habits of *Xyleborus destruens* Bldf, the near-primary borer of teak trees on Java. Entomologische Berichten, Amsterdam 22: 7–18.

[B224] Kalshoven LGE (1964) The occurrence of *Xyleborus perforans* (Woll) and *X. similis* in Java (Coleoptera, Scolytidae). Beaufortia 11: 131–142.

[B225] Kasson MT Wickert KL Stauder CM Macias AM Berger MC Simmons DR Short DPG DeVallance DB Hulcr J (2016) Mutualism with aggressive wood-degrading *Flavodon ambrosius* (Polyporales) facilitates niche expansion and communal social structure in *Ambrosiophilus* ambrosia beetles. Fungal Ecology 23: 86–96. 10.1016/j.funeco.2016.07.002

[B226] Kendra PE Montgomery WS Niogret J Deyrup MA Guillen L Epsky ND (2012) *Xyleborus glabratus*, *X. affinis*, and *X. ferrugineus* (Coleoptera: Curculionidae: Scolytinae): Electroantennogram responses to host-based attractants and temporal patterns in host-seeking flight. Environmental Entomology 41: 1597–1605. 10.1603/EN12164 23321108

[B227] Kendra PE Niogret J Montgomery WS Deyrup MA Epsky ND (2015) Cubeb oil lures: terpenoid emissions, trapping efficacy, and longevity for attraction of redbay ambrosia beetle (Coleoptera: Curculionidae: Scolytinae). Journal of Economic Entomology 108: 350–361. 10.1093/jee/tou023 26470139

[B228] Kendra PE Montgomery WS Deyrup MA Wakarchuk D (2016) Improved lure for redbay ambrosia beetle developed by enrichment of α-copaene content. Journal of Pest Science 89: 427–438. 10.1007/s10340-015-0708-5

[B229] Kirkendall LR (1993) Ecology and evolution of biased sex ratios in bark and ambrosia beetles. In: Wrensch DL Ebbert MA (Eds) Evolution and Diversity of Sex Ratio in Insects and Mites.Chapman and Hall, New York, 235–345. 10.1007/978-1-4684-1402-8_8

[B230] Kirkendall LR (2018) Invasive bark beetles (Coleoptera, Curculionidae, Scolytinae) in Chile and Argentina, including two species new for South America, and the correct identity of the *Orthotomicus* species in Chile and Argentina. Diversity 10: 1–40. 10.3390/d10020040

[B231] Kirkendall LR Ødegaard F (2007) Ongoing invasions of old-growth tropical forests: establishment of three incestuous beetle species in Central America (Curculionidae, Scolytinae). Zootaxa 1588: 53–62. 10.11646/zootaxa.1588.1.3

[B232] Kirkendall LR Faccoli M (2010) Bark beetles and pinhole borers (Curculionidae, Scolytinae, Platypodinae) alien to Europe. ZooKeys 56: 227–251. 10.3897/zookeys.56.529 PMC3088324 21594183

[B233] Kirkendall LR Biedermann PHW Jordal BH (2015) Evolution and diversity of bark and ambrosia beetles. In: Vega FE Hofstetter RW (Eds) Bark Beetles.Biology and Ecology of Native and Invasive Species. Academic Press, London, 85–156. 10.1016/B978-0-12-417156-5.00003-4

[B234] Knížek M (1988) *Xyleborus alni* Niijima, 1909. Acta Entomologica Bohemoslovaca 85: 396–396.

[B235] Knížek M (2011) Scolytinae. In: Löbl I Smetana A (Eds) Catalogue of Palaearctic Coleoptera (Vol.7), Curculionoidea I. Apollo Books, Stenstrup, 204–251.

[B236] Kolenati FA (1846) Meletemata Entomologica. Fascicule III. Brachyelytra Caucasi cum distributione geographica adnexis Pselaphinis, Scydmaenis, Notoxibus, et Xylophagis. Petropoli: Typis Imperialis Academiae Scientarum [6] 44 pp. [pls 12–14.]

[B237] Kôno M (1938) Neue und wenig bekannte Ipiden als Schädlinge an Sachalintannen und Ezofichten in Hokkaido. Insecta Matsumurana 12: 64–73.

[B238] Kurentzov AI (1941) Koroedy Dalnego Vostoka SSSR [Bark-beetles of the Far East, USSR]. Izdatelstvo Akademii Nauk SSSR, Moskva, 234 pp.

[B239] Kurentzov AI (1948) Novye dannye po faune koroedov (Coleoptera, Ipidae) Primorskogo Kraya [New data on the bark-beetle fauna of the Maritime Region (Soviet Far East). Entomologischeskoe Obozrenie 30: 50–52.

[B240] Lacordaire T (1865) Histoire Naturelle des Insectes. Genera des Coléoptères ou exposé méthodique et critique de tous les genres proposés jusqu’ici dans cet ordre d’insectes (Vol. 7). [1866] Roret, Paris, 620 pp.

[B241] Landi L Gómez D Braccini CL Pereyra VA Smith SM Marvaldi AE (2017) Morphological and molecular identification of the invasive *Xylosandrus crassiusculus* (Motschulsky) (Coleoptera: Curculionidae: Scolytinae) and its South American range extending into Argentina and Uruguay. Journal of Economic Entomology 110: 344–349. 10.1093/aesa/sax032

[B242] Landi L Braccini CL Knížek M Pereyra VA Marvaldi AE (2019) A newly detected exotic ambrosia beetle in Argentina: *Euwallacea interjectus* (Coleoptera: Curculionidae: Scolytinae). Florida Entomologist 102: 240–242. 10.1653/024.102.0141

[B243] Le Pelley RH (1968) Pests of Coffee. Longmans, Green and Co. Ltd., London and Harlow, 590 pp.

[B244] Lea AM (1894) Descriptions of new species of Bostrychidae. Proceedings of the Linnean Society of New South Wales, series 2, 8: 317–323.

[B245] Lea AM (1910) On Australian and Tasmanian Coleoptera, with descriptions of new species Part I. Proceedings of the Royal Society of Victoria, New Series 22: 113–152. [pl. 30.]

[B246] Li Y Bateman CC Skelton J Jusino MA Nolen ZJ Simmons DR Hulcr J (2017) Wood decay fungus *Flavodon ambrosius* (Basidiomycota: Polyporales) is widely farmed by two genera of ambrosia beetles. Fungal Biology 121: 984–989. 10.1016/j.funbio.2017.08.004 29029704

[B247] Li Y Lin W Tang Y Hulcr J Gao L (2020) . *Xyleborus festivus* in southern China: distribution, host range and symbiotic fungi. Plant Protection 46: 147–151. (Chinese with English abstract). 10.16688/j.zwbh.2019001

[B248] Lightle DM Gandhi KJK Cognato AI Mosley BJ Nielsen DG Herms DA (2007) New reports of exotic and native ambrosia and bark beetle species (Coleoptera: Curculionidae: Scolytinae) from Ohio. The Great Lakes Entomologist 40: 194–200.

[B249] Lin W Li Y Johnson AJ Gao L (2019) New area records and new hosts of *Ambrosiodmus minor* (Stebbing) (Coleoptera: Curculionidae: Scolytinae) in Mainland China. The Coleopterists Bulletin 73: 684–686. 10.1649/0010-065X-73.3.684

[B250] Lynn KMT Wingfield MJ Durán A Marincowitz S Oliveira LSS de Beer W Barnes I (2020) *Euwallacea perbrevis* (Coleoptera: Curculionidae: Scolytinae), a confirmed pest on *Acacia crassicarpa* in Riau, Indonesia, and a new fungal symbiont; *Fusarium rekanum* sp. nov.. Antonie van Leeuwenhoek 113: 803–823. 10.1007/s10482-020-01392-8 32086683

[B251] Madoffe S Bakke A (1995) Seasonal fluctuations and diversity of bark and wood-boring beetles in lowland forest: implications for management practices. South African Forestry Journal 173: 9–15. 10.1080/00382167.1995.9629684

[B252] Maiti PK Saha N (1986) Contributions to the knowledge of the bark and timber beetles (Scolytidae: Coleoptera) of the Andaman and Nicobar Islands. Records of the Zoological Survey of India, Miscellaneous Publications, Occasional Papers 86: 1–182.

[B253] Maiti PK Saha N (2004) Fauna of India and the Adjacent Countries. Scolytidae: Coleoptera (Bark- and Ambrosia-beetles) (Vol. 1). Part-1. Introduction and Tribe Xyleborini. Zoological Survey of India, Kolkata, 268 pp.

[B254] Mandelshtam MY (2006) New synonymies and new combinations in Scolytidae from the Kuril Archipelago and continental territories of the Russian Far East (Coleoptera). Zoosystematica Rossica 15: 323–325.

[B255] Mandelshtam MY Nikitsky NB (2010) [Review of Scolytidae (Coleoptera) type specimens from V. Motschulsky collection preserved in the Zoological Museum of Moscow State University]. Byulleten’ Moskovskogo Obshchestva Ispytatelei Prirody Otdel Biologicheskii 115(5): 13–21.

[B256] Mandelshtam MYu Yakushkin EA Petrov AV (2018) Oriental ambrosia beetles (Coleoptera: Curculionidae: Scolytinae): new inhabitants of Primorsky krai in Russia. Russian Journal of Biological Invasions 9(4): 355–365. 10.1134/S2075111718040082

[B257] Mandelshtam MYu Petrov AV Smith SM Cognato AI (2019) Resurrection of *Heteroborips* Reitter, 1913 (Coleoptera: Curculionidae: Scolytinae) from synonymy with *Xyleborus* Eichhoff, 1864. The Coleopterists Bulletin 73: 387–394. 10.1649/0010-065X-73.2.387

[B258] Maner ML Hanula JL Braman SK (2013) Gallery productivity, emergence, and flight activity of the redbay ambrosia beetle (Coleoptera: Curculionidae: Scolytinae). Environmental Entomology 42: 642–647. 10.1603/EN13014 23905726

[B259] Markalas S Kalapanida M (1997) Flight pattern of some Scolytidae attracted to flight barrier traps baited with ethanol in oak forest in Greece. Anzeiger für Schädlingskunde, Pflanzenschutz, Umweltschutz 70: 55–67. 10.1007/BF01996922

[B260] Mayfield III AE MacKenzie M Cannon P Oak S Horn S Hwang J Kendra PE (2013) Suitability of California bay laurel and other species as hosts for the non-native redbay ambrosia beetle and granulate ambrosia beetle. Agricultural and Forest Entomology 15: 227–235. 10.1111/afe.12009

[B261] McCullough DM Work TT Cavey JF Liebhold AM Marshall D (2006) Interceptions of nonindigenous plant pests at US ports of entry and border crossings over a 17-year period. Biological Invasions 8: 611–630. 10.1007/s10530-005-1798-4

[B262] McPherson BA Erbilgin N Wood DL Svihra P Storer AJ Standiford RB (2008) Attraction of ambrosia and bark beetles to coast live oaks infected by *Phytophthora ramorum*. Agricultural and Forest Entomology 10: 315–321. 10.1111/j.1461-9563.2008.00386.x

[B263] Mendel Z Protasov A Sharon M Zveibil A Yehuda SB O’Donnell K Rabaglia R Wysocki M Freeman S (2012) An Asian ambrosia beetle *Euwallacea fornicatus* and its novel symbiotic fungus *Fusarium* sp. pose a serious threat to Israeli avocado industry. Phytoparasitica 40: 235–238. 10.1007/s12600-012-0223-7

[B264] Mizuno T Kajimura H (2002) Reproduction of the ambrosia beetle, *Xyleborus pfeili* (Ratzeburg) (Col., Scolytidae), on semi-artificial diet. Journal of Applied Entomology 126: 455–462. 10.1046/j.1439-0418.2002.00691.x

[B265] Montrouzier X (1861) Essai sur la faune entomologique de la Nouvelle Calédonie et des îles des Pins, Art, Lifu, etc. (suite). Annales de la Société Entomologique de France, série 4, 1: 265–306.

[B266] Motschulsky V (1863) Essai d’un catalogue des insectes de l’île Ceylan. (Suite). Bulletin de la Société Impériale des Naturalistes de Moscou 36: 421–532.

[B267] Motschulsky V (1866) Essai d’un catalogue des insectes de l’île de Ceylan. (Supplément). Bulletin de la Société Impériale des Naturalistes de Moscou 39: 393–446.

[B268] Mulsant ME Rey C (1856) Description d’une nouvelle espèce de Coléoptère du genre *Bostrichus*. Opuscules Entomologiques 7(1856): 111–113. 10.5962/bhl.title.2682

[B269] Murayama J (1930) Révisions des familes des Ipides et des Platypides de Corée. Journal of the Chosen Natural History Society 11: 1–34. [2 pls.]

[B270] Murayama J (1931) Révision des familles des Ipides et Platypides (Coléoptères) de l’ile de Quelpart. Annotationes Zoologicae Japonenses 13: 39–62. [2 pls.]

[B271] Murayama J (1934) Notes on the Ipidae (Coleoptera) from Kiushu. Annotationes Zoologicae Japonenses 14: 287–300.

[B272] Murayama J (1936) Notes sur les Scolytides (Coléoptères) de Honshû et Kiushû, Japon. Tenthredo 1: 121–149.

[B273] Murayama J (1943) Nouvelles espèces des Scolytides (Coléoptères) du Manchoukuo. Annotationes Zoologicae Japonenses 22: 96–100.

[B274] Murayama J (1950) A new genus and some new species of Scolytidae from Japan (Coleoptera). Transactions of the Shikoku Entomological Society 1: 49–53.

[B275] Murayama J (1951) New genus and species of Scolytidae (Coleoptera) from Ohshima and Shionomisaki, Wakayama prefecture. Bulletin of the Faculty of Agriculture, Yamaguti University 2: 1–7.

[B276] Murayama J (1952) Notes on the scolytid-beetles (Coleoptera) from southern and western parts of Izu Peninsula, Shizuoka Prefecture. Bulletin of the Faculty of Agriculture, Yamaguti University 3: 15–23.

[B277] Murayama J (1953) The insect fauna of Mt Ishizuchi and Omogo valley, Iyo, Japan. The Scolytidae and Platypodidae (Coleoptera). Transactions of the Shikoku Entomological Society 3: 144–165.

[B278] Murayama J (1954) Scolytid-fauna on the northern half of Honshu with a distribution table of all the scolytid-species described from Japan. Bulletin of the Faculty of Agriculture, Yamaguti University 5: 149–212.

[B279] Murayama J (1955) Supplementary notes on the scolytid-fauna of Japan. Bulletin of the Faculty of Agriculture, Yamaguti University 6: 81–106. [pls. 3, 4.]

[B280] Murayama J (1958) Studies in the scolytid-fauna of the northern half of the Far East, IV: new genera and new species. Bulletin of the Faculty of Agriculture, Yamaguti University 9: 927–936.

[B281] Murayama J Kalshoven LGE (1962) *Xyleborus morstatti* Hag, a synonym of *X. compactus* Eichh. (Col, Scolytidae). Entomologische Berichten, Amsterdam 22: 247–250.

[B282] Murphy DH Meepol W (1990) Timber beetles of the Ranong mangrove forests. Mangrove Ecosystems Occasional Papers 7: 5–8.

[B283] Nakashima T Otomo T Owada Y Iizuka T (1992) SEM observations on growing conditions of the fungi in the galleries of several ambrosia beetles: (Coleoptera: Scolytidae and Platypodidae). Journal of the Faculty of Agriculture, Hokkaido University 65: 239–273.

[B284] Niisima Y (1909) Die Scolytiden Hokkaidos unter Berücksichtigung ihrer Bedeutung für Forstschäden. Journal of the College of Agriculture, Tohoku Imperial University, Sapporo 3: 109–179.

[B285] Niisima Y (1910) Die Borkenkaefer Nord- und Mittel-Japans. Transactions of the Sapporo Natural History Society 3: 1–18.

[B286] Nikulina T Mandelshtam M Petrov A Nazarenko V Yunakov N (2015) A survey of the weevils of Ukraine. Bark and ambrosia beetles (Coleoptera: Curculionidae: Platypodinae and Scolytinae) Zootaxa 3912: 1–61. 10.11646/zootaxa.3912.1.1 25661778

[B287] Nobuchi A (1966) Bark-beetles injurious to pine in Japan. Bulletin of the Government Forest Experiment Station, Tokyo 185: 1–49. [pls. 1–6.] [in Japanese; summary and key in English]

[B288] Nobuchi A (1979) Ambrosia beetles of mahogany in the Philippines (Coleoptera: Scolytidae and Platypodidae). Kontyû 47: 406–407.

[B289] Nobuchi A (1981a) Studies on Scolytidae (Coleoptera) XXII. Six new species and two new females of the genus *Xyleborus* from Japan. Kontyû 49: 143–154.

[B290] Nobuchi A (1981b) Studies on Scolytidae XXIII. The ambrosia beetles of the genus *Xylosandrus* Reitter from Japan (Coleoptera). Bulletin of the Forestry and Forestry Products Research Institute 314: 27–37.

[B291] Nobuchi A (1985) Family Scolytidae. Check-list of Coleoptera of Japan 30: 1–32.

[B292] Norris DM (1976) Chemical interdependencies among *Xyleborus* spp. ambrosia beetles and their symbiotic microbes. Material und Organismen, Beiheft 3: 479–488.

[B293] Nunberg M (1956) Zmiany nazw i synonimika niektórych korników (Coleoptera, Scolytidae) [Namensänderungen und Synonymie einiger Borkenkäfer]. Annales Zoologici 16: 207–214.

[B294] Nunberg M (1959) Die Gattung *Xyleborus* Eichhoff (Coleoptera: Scolytidae) Ergänzungen, Berichtigungen und Erweiterung der Diagnosen. Beiträge zur Entomologie 9: 413–466.

[B295] Nunberg M (1961) Zur Kenntnis der malayischen und aethiopischen Borken- und Kernkäferfauna (Col. Scolytidae und Platypodidae). The Annals and Magazine of Natural History, series 13, 3: 609–632. 10.1080/00222936008651066

[B296] Nunberg M (1963) Die Gattung *Xyleborus* Eichhoff (Coleoptera, Scolytidae). Ergänzungen, Berichtigungen und Erweiterung der Diagnosen (II. Teil). Annales du Musée Royale du Congo Belge, Série in 8°, Sciences Zoologiques 115: 1–127.

[B297] O’Donnell K Sink S Libeskind-Hadas R Hulcr J Kasson MT Ploetz RC Konkol JL Ploetz JN Carillo D Campbell A Duncan RE Liyanage PN Eskalen A Na F Geiser DM Bateman C Freeman S Mendel Z Sharon M Aoki T Cossé AA Rooney AP (2015) Discordant phylogenies suggest repeated host shifts in the *Fusarium*-*Euwallacea* ambrosia beetle mutualism. Fungal Genetics and Biology 82: 277–290. 10.1016/j.fgb.2014.10.014 25445310

[B298] Ohno S (1990) The Scolytidae and Platypodidae (Coleoptera) from Borneo found in logs at Nagoya port 1. Research Bulletin of the Plant Protection Service, Japan 26: 83–94.

[B299] Ohno S Yoshioka K Yoneyama K Nakazawa H (1988) The Scolytidae and Platypodidae (Coleoptera) from Solomon Islands, found in logs at Nagoya port, I. Research Bulletin of the Plant Protection Service, Japan 24: 91–95.

[B300] Ohno S Yoshioka K Uchida N Yoneyama K Tsukamoto K (1989) The Scolytidae and Platypodidae (Coleoptera) from Bismark Archipelago found in logs at Nagoya port Japan. Research Bulletin of the Plant Protection Service, Japan 25: 59–69.

[B301] Okins KE Thomas MC (2010) New North American record for *Xyleborinus andrewesi* (Coleoptera: Curculionidae: Scolytinae). Florida Entomologist 93: 133–134. 10.1653/024.093.0122

[B302] Olivier AG (1800) Entomologie, ou Histoire Naturelle des Insectes, avec Leurs Caractères Génériques et Spécifiques, leur Description, leur Synonymie, et leur Figure Enluminée. Coléoptères. Tome quatrième. de Lanneau, Paris, 519 pp [72 pls.] 10.5962/bhl.title.49479

[B303] Palm T (1959) Die Holz- und Rinden-Käfer der süd- und mittelschwedischen Laubbäume. Opuscula Entomologica, Supplementum 16: 1–374.

[B304] Panzer GWF (1793) Faunae Insectorum Germanicae Initia oder Deutschlands Insekten. Heft 8. Nürenberg: Felsecker, 24 pp. [24 pls.] 10.5962/bhl.title.15007

[B305] Papp T de Beer ZW Migliorini D Nel WJ Wingfield MJ (2018) The polyphagous shot hole borer (PSHB) and its fungal symbiont *Fusarium euwallaceae*: a new invasion in South Africa. Australasian Plant Pathology 47: 231–237. 10.1007/s13313-018-0545-0

[B306] Park S Smith SM Cognato AI Beaver RA (2020) Catalogue of Korean xyleborine ambrosia beetles (Coleoptera: Curculionidae) with seven new species. Journal of Asia-Pacific Biodiversity 13: 210–228. 10.1016/j.japb.2020.01.002

[B307] Peck WD (1817) On the insects which destroy the young branches of the pear-tree, and the leading shoot of the weymouth-pine. Massachusetts Agricultural Repository and Journal 4: 205–2011. [pls 1–2.]

[B308] Peer K Taborsky M (2004) Female ambrosia beetles adjust their offspring sex ratio according to outbreeding opportunities for their sons. Journal of Evolutionary Biology 17: 257–264. 10.1111/j.1420-9101.2003.00687.x 15009259

[B309] Peer K Taborsky M (2005) Outbreeding depression, but no inbreeding depression in haplodiploid ambrosia beetles with regular sibling mating. Evolution 59: 317–323. 10.1111/j.0014-3820.2005.tb00992.x 15807418

[B310] Peer K Taborsky M (2007) Delayed dispersal as a potential route to cooperative breeding in ambrosia beetles. Behavioral Ecology and Sociobiology 61: 729–739. 10.1007/s00265-006-0303-0

[B311] Peña JE Weihman SW McLean S Cave RD Carillo D Duncan RE Evans G Krauth S Thomas MC Lu SS Kendra PE Roda AL (2015) Predators and parasitoids associated with Scolytinae in *Persea* species (Laurales: Lauraceae) and other Lauraceae in Florida and Taiwan. Florida Entomologist 98: 903–910. 10.1653/024.098.0314

[B312] Pennacchio F Roversi PF Francardi V Gatti E (2003) *Xylosandrus crassiusculus* (Motschulsky) a bark beetle new to Europe (ColeopteraScolytidae). Redia 86: 77–80.

[B313] Petrov AV Mandelshtam MYu (2018) Description of a new species of *Cnestus* Sampson, 1911, and notes on other species from South America (Coleoptera: Curculionidae: Scolytinae). Koleopterologische Rundschau 88: 269–274.

[B314] Pfeffer A (1944) Bemerkungen zur Arbeit von Hans Eggers: Zur Palearktischen Borkenkäferfauna. VIII. Borkenkäfer aus dem asiatischen Russland (Col: Ipidae). Arbeiten über Morphologische und Taxonomische Entomologie aus Berlin-Dahlem 11: 130–131.

[B315] Pfeffer A (1994) Zentral- und Westpaläarktische Borken- und Kernkäfer. Pro Entomologia, Basel, 310 pp.

[B316] Rabaglia RJ Okins KE (2011) Entomology section. Tri-ology 50(3): 6–9.

[B317] Rabaglia RJ Dole SA Cognato AI (2006) Review of America Xyleborina (Coleoptera: Curculionidae: Scolytinae) occurring north of Mexico, with an illustrated key. Annals of the Entomological Society of America 99: 1034–1056. 10.1603/0013-8746(2006)99[1034:ROAXCC]2.0.CO;2

[B318] Rabaglia RJ Vandenburg N Acciavatti RE (2009) First records of *Anisandrus maiche* Stark (Coleoptera: Curculionidae: Scolytinae) from North America. Zootaxa 2137: 23–28. 10.11646/zootaxa.2137.1.2

[B319] Rabaglia RJ Knížek M Johnson W (2010) First records of *Xyleborinus octiesdentatus* (Murayama) (Coleoptera, Curculionidae, Scolytinae) from North America. ZooKeys 56: 219–226. 10.3897/zookeys.56.528 PMC3088321 21594182

[B320] Rabaglia RJ Cognato AI Hoebeke ER Johnson CW Labonte JR Carter ME Vlach JJ (2019) Early detection and rapid response. A 10-year summary of the USDA Forest Service program of surveillance for non-native bark and ambrosia beetles. American Entomologist 65: 29–42. 10.1093/ae/tmz015

[B321] Rabaglia RJ Smith SL Rugman-Jones P DiGirolomo M Ewing C Eskalen A (2020a) Establishment of a non-native xyleborine ambrosia beetle, *Xyleborus monographus* (Fabricius) (Coleoptera: Curculionidae: Scolytinae), new to North America in California. Zootaxa 4786: 269–276. 10.11646/zootaxa.4786.2.8 33056488

[B322] Rabaglia RJ Beaver RA Johnson AJ Schmaedick MA Smith SM (2020b) The bark and ambrosia beetles (Coleoptera: Curculionidae: Scolytinae and Platypodinae) of American Samoa. Zootaxa 4808: 171–195. 10.11646/zootaxa.4808.1.11 33055997

[B323] Rangel R Pérez M Sánchez S Capello S (2012) Population fluctuation of *Xyleborus ferrugineus* and *X. affinis* (Coleoptera: Curculionidae) in ecosystems of Tabasco, Mexico. Revista de Biología Tropical 60: 1577–1588. 10.15517/rbt.v60i4.2075 23342512

[B324] Ranger CM Reding ME Persad AB Herms DA (2010) Ability of stress-related volatiles to attract and induce attacks by *Xylosandrus germanus* and other ambrosia beetles (Coleoptera: Curculionidae, Scolytinae). Agricultural and Forest Entomology 12: 177–185. 10.1111/j.1461-9563.2009.00469.x

[B325] Ranger CM Schultz PB Frank SD Chong JH Reding ME (2015) Non-native ambrosia beetles as opportunistic exploiters of living but weakened trees. PLoS ONE 10: e0131496. 10.1371/journal.pone.0131496 PMC4489854 26134522

[B326] Ranger CM Reding ME Schultz PB Oliver JB Frank SD Addesso KM Chong JH Sampson B Werle C Gill S Krause C (2016) Biology, ecology, and management of nonnative ambrosia beetles (Coleoptera: Curculionidae: Scolytinae) in ornamental plant nurseries. Journal of Integrated Pest Management 7: 1–9. 10.1603/EN11299

[B327] Ratzeburg JTC (1837) Die Forst-insekten: oder Abbildung und Beschreibung der in den Wäldern Preussens und der Nachbarstaaten als schädlich oder nützlich bekannt gewordenen Insekten. Erster Theil. Die Käfer. Nicolai, Berlin, 202 pp [21 pls.] 10.5962/bhl.title.34392

[B328] Reding ME Schultz PB Ranger CM Oliver JB (2011) Optimizing ethanol-baited traps for monitoring damaging ambrosia beetles (Coleoptera: Curculionidae: Scolytinae) in ornamental nurseries. Journal of Economic Entomology 104: 2017–2024. 10.1603/EC11119 22299365

[B329] Reding ME Ranger CM Oliver JB Schultz PB (2013) Monitoring attack and flight activity of *Xylosandrus* spp. (Coleoptera: Curculionidae: Scolytinae): The influence of temperature on activity. Journal of Economic Entomology 106: 1780–1787. 10.1603/EC13134 24020293

[B330] Reitter E (1913) Bestimmungs-Tabelle der Borkenkäfer (Scolytidae) aus Europa und den Angrenzenden Ländern. Wiener Entomologische Zeitung, 32, Beiheft, 116 pp 10.3931/e-rara-68983

[B331] Rey C (1883) [new taxa]. In: Eichhoff WJ (Ed.) Les xylophages d’Europe. Avec des notes et additions concernant la faune gallo-rhénane. (Suite et fin).Revue d’Entomologie 2: 121–145.

[B332] Roberts H (1977) Observations on the biology of some tropical rain forest Scolytidae (Coleoptera) from Fiji II. Subfamily Ipinae- tribe Xyleborini. Journal of Natural History 11: 251–272. 10.1080/00222937700770181

[B333] Saha N Maiti PK (1984) On a collection of scolytid beetles (Scolytidae: Coleoptera) from Sikkim, India. Records of the Zoological Survey of India 81(3–4): 1–8.

[B334] Saha N Maiti PK (1987) Description of hitherto unknown males of three species of scolytid beetles (Scolytidae: Coleoptera) from India. Bulletin of the Zoological Survey of India 8: 71–76.

[B335] Saha N Maiti PK (1996) Insecta: Coleoptera: Scolytidae. Fauna of West Bengal 6B: 775–866.

[B336] Saha N Maiti PK Chakraborti S (1992) On some species of *Xylosandrus* Reitter (Coleoptera: Scolytidae) from the sub-Himalayan West Bengal with description of a new species. Records of the Zoological Survey of India 91: 9–27.

[B337] Sahlberg CR (1836) Dissertatio entomologica insecta fennica enumerans, cujus particulam decimam partis secundae, cons. ampl. facult. philos. ad Imper. Univers. Alexandr. in Fennia, publico offert examini Carolus Reginaldus Sahlberg, respondente Alfredo Wacklin, Ostrobottniensi. In audit. philos. die 7 Maji 1836. J. C. Frenckel, Aboae, 145–160.

[B338] Sampson FW (1911) On two new wood-boring beetles (Ipidae). The Annals and Magazine of Natural History, series 8, 8: 381–384. 10.1080/00222931108693046

[B339] Sampson FW (1912) Some new species of Ipidae and Platypodidae in the British Museum. The Annals and Magazine of Natural History, series 8, 10: 245–250. 10.1080/00222931208693228

[B340] Sampson FW (1913) Some hitherto undescribed Ipidae and Platypodidae from India and Burma. The Annals and Magazine of Natural History, series 8, 12: 443–452. 10.1080/00222931308693422

[B341] Sampson FW (1914) No. XVIII. – Coleoptera; Platypodidae and Ipidae from the Seychelles Islands. Transactions of the Linnean Society of London, Zoology, Second Series 16: 379–391. 10.1111/j.1096-3642.1913.tb00155.x

[B342] Sampson FW (1919) Notes on Platypodidae and Scolytidae collected by Mr. G. E. Bryant and others. The Annals and Magazine of Natural History, series 9, 4: 105–114. 10.1080/00222932108632486

[B343] Sampson FW (1921) Further notes on Platypodidae and Scolytidae collected by Mr. G. E. Bryant and others. The Annals and Magazine of Natural History, series 9, 7: 25–37. 10.1080/00222932108632486

[B344] Sampson FW (1922) Previously undescribed Scolytidae and Platypodidae from the Indian area. The Annals and Magazine of Natural History, series 9, 10: 145–152. 10.1080/00222932208632753

[B345] Sampson FW (1923) Previously undescribed Scolytidae and Platypodidae from the Indian area, Part II. The Annals and Magazine of Natural History, series 9, 11: 285–289. 10.1080/00222932308632858

[B346] Samuelson GA (1981) A synopsis of Hawaiian Xyleborini (Coleoptera: Scolytidae). Pacific Insects 23: 50–92.

[B347] Saruhan H Akyol I (2012) Monitoring population density and fluctuations of *Anisandrus dispar* and *Xyleborinus saxesenii* (Coleoptera: Scolytinae, Curculionidae) in hazelnut orchards. African Journal of Biotechnology 11: 4202–4207. 10.5897/AJB11.4185

[B348] Schaufuss CFC (1897) Beitrag zur Käferfauna Madagascars III. Missions scientifiques de M Ch Alluaud aux îles Séchelles (1892) et à Diego-Suarez, Madagascar (1893) (Scolytidae et Platypodidae). Tijdschrift voor Entomologie 40: 209–225.

[B349] Schedl KE (1931) Notes on the genus *Xyleborus* Eichh. The Annals and Magazine of Natural History, series 10, 8: 339–347. 10.1080/00222933108673402

[B350] Schedl KE (1933) New Scolytidae from the Philippines. Philippine Journal of Science 51: 101–107.

[B351] Schedl KE (1934a) Neue Borkenkäfer. Entomologische Blätter 30: 37–39.

[B352] Schedl KE (1934b) Neue indomalayische Scolytidae. II. Beitrag. Entomologische Berichten, Amsterdam 9: 84–92.

[B353] Schedl KE (1934c) Scolytidae and Platypodidae In: Winkler A (Ed.) Catalogus Coleopterorum Regionis Palaearctica.(4 volumes). By author, Wien, 1632–1647.

[B354] Schedl KE (1935a) Fauna Philippinensis (Platypodidae et Scolytidae) III. Philippine Journal of Science 56: 395–403.

[B355] Schedl KE (1935b) New bark beetles and ambrosia-beetles (Col). Stylops 4: 270–276. 10.1111/j.1365-3113.1935.tb00659.x

[B356] Schedl KE (1936a) Notes on Malayan Scolytidae and Platypodidae and descriptions of some new species. Journal of the Federated Malay States Museums 18: 1–18.

[B357] Schedl KE (1936b) Scolytidae and Platypodidae. Contribution 35. The collection of the South Australian Museum. Records of the South Australian Museum 5: 513–535.

[B358] Schedl KE (1936c) Scolytidae and Platypodidae: Fauna Philippinensis, IV. Philippine Journal of Science 60: 59–67.

[B359] Schedl KE (1936d) Some new Scolytidae and Platypodidae from the Malay Peninsula. Journal of the Federated Malay States Museums 18: 19–35.

[B360] Schedl KE (1937a) Scolytidae and Platypodidae. 34. Contribution. Fauna Borneensis. Part I. Sarawak Museum Journal 4: 543–552.

[B361] Schedl KE (1937b) Scolytidae und Platypodidae. 45. Beitrag. Vereinschrift der Gesellschaft Luxemburger Naturfreunde 31: 15–17.

[B362] Schedl KE (1937c) Scolytidae und Platypodidae-Zentral und südamerikanische Arten. Arquivos do Instituto de Biologia Vegetal, Rio de Janeiro 3: 155–170.

[B363] Schedl KE (1938) Scolytidae und Platypodidae. 41. Beitrag zur Morphologie und Systematik der Scolytoidea. Mitteilungen aus dem Zoologischen Museum in Berlin 23: 459–464.

[B364] Schedl KE (1939a) Malaysian Scolytidae and Platypodidae (IV). 57^th^ contribution. Journal of the Federated Malay States Museums 18: 327–364.

[B365] Schedl KE (1939b) Scolytidae and Platypodidae. 47. Beitrag zur Morphologie und Systematik der Scolytoidea. Tijdschrift voor Entomologie 82: 30–53.

[B366] Schedl KE (1940a) Scolytidae and Platypodidae 61. Contribution to the morphology and taxonomy of the Scolytoidea The Annals and Magazine of Natural History, series 11, 5: 433–442. 10.1080/00222934008527057

[B367] Schedl KE (1940b) Scolytidae und Platypodidae (Coleoptera). Arbeiten über Morphologische und Taxonomische Entomologie aus Berlin-Dahlem 7: 203–208.

[B368] Schedl KE (1941) 77^th^ Contribution to the morphology and taxonomy of the Scolytoidea. Proceedings of the Hawaiian Entomological Society 11: 109–116.

[B369] Schedl KE (1942a) Forschungsberichte zur Scolytoiden-Fauna der Malayischen Halbinsel, V. 80. Beitrag zur Morphologie und Systematik der Scolytoidea. Kolonialforstliche Mitteilungen 5: 169–218.

[B370] Schedl KE (1942b) Interessante und neue Scolytiden und Platypodiden aus der australischen Region. 79. Beitrag zur Morphologie und Systematik der Scolytoidea. Mitteilungen der Münchener Entomologischen Gesellschaft 32: 162–201.

[B371] Schedl KE (1942c) Neue Scolytidae aus Java. 76. Beitrag zur Morphologie und Systematik der Scolytoidea. Tijdschrift voor Entomologie 85: 1–49.

[B372] Schedl KE (1948) New species and records of Australian Scolytidae. Proceedings of the Royal Society of Queensland 60: 25–29.

[B373] Schedl KE (1949) Neotropical Scolytoidea. I. 97^th^ Contribution to the morphology and taxonomy of the Scolytoidea (Col). Revista Brasileira de Biologia 9: 261–284.

[B374] Schedl KE (1950a) Fauna Fijiana (Scolytoidea). 94. Contribution to the morphology and taxonomy of the Scolytoidea. Occasional Papers of the Bernice P. Bishop Museum 20: 35–54.

[B375] Schedl KE (1950b) Fauna Indo-Malayensis, II. 104. Contribution to the morphology and taxonomy of the Scolytoidea The Annals and Magazine of Natural History, series 12, 3: 892–900. 10.1080/00222935008654721

[B376] Schedl KE (1951a) Fauna Indomalayaensis, I. Tijdschrift voor Entomologie 93: 41–98. 10.1080/00222935008654721

[B377] Schedl KE (1951b) Fauna Samoanus (Scolytoides), I. 109. Contribution. Bernice P. Bishop Museum Occasional Papers 20: 131–156.

[B378] Schedl KE (1952a) Fauna Philippinensis, VIII. 123. Contribution to the morphology and taxonomy of the Scolytoidea. Philippine Journal of Science 80: 363–371.

[B379] Schedl KE (1952b) Formosan Scolytoidea, I. lll. Contribution. Philippine Journal of Science 81: 61–65.

[B380] Schedl KE (1952c) Scolytoidea Congolais IV. Contribution 132 à la morphologie et à la systématique des Scolytoidea. Bulletin – Institut Royal des Sciences Naturelles de Belgique 28(32): 1–12.

[B381] Schedl KE (1952d) Zur synonymie der Borkenkäfer I. Entomologische Blätter 47/48: 158–164.

[B382] Schedl KE (1953a) Bark and ambrosia-beetles from Indochina. 127. Contribution to the morphology and taxonomy of the Scolytoidea. Revue Française d’Entomologie 20: 123–130.

[B383] Schedl KE (1953b) Fauna Indomalayensis.- III. Contribution 133 to the morphology and taxonomy of the Scolytoidea The Annals and Magazine of Natural History, series 12, 6: 288–304. 10.1080/00222935308654424

[B384] Schedl KE (1953c) Fauna Sinensis, I. Entomologische Blätter 49: 22–30.

[B385] Schedl KE (1953d) New Scolytoidea. Queensland Museum Memoirs 13: 80–83.

[B386] Schedl KE (1954a) Fauna Indomalayensis, IV: 141. Beitrag zur Morphologie und Systematik der Scolytoidea. The Philippine Journal of Science 83: 137–159.

[B387] Schedl KE (1954b) Scolytoidea from the Gold Coast. I. (135. Contribution to the morphology and taxonomy of the Scolytoidea). Revue de Zoologie et de Botanique Africaines 50: 45–88.

[B388] Schedl KE (1955a) Borken- und Ambrosiakäfer aus dem pazifischen Raum. 150. Beitrag zur Morphologie und Systematik der Scolytoidea. Entomologische Arbeiten aus dem Museum G. Frey 6: 277–310.

[B389] Schedl KE (1955b) Fauna Sinensis, II. 152. Beitrag zur Morphologie und Systematik der Scolytoidea. Entomologische Blätter 51: 45–46.

[B390] Schedl KE (1957) Scolytoidea nouveaux du Congo Belge. II. Mission R. Mayné – K. E. Schedl 1952. Annales du Musée Royale du Congo Belge, Série 8°, Sciences Zoologiques 56: 1–162.

[B391] Schedl KE (1958a) A few new African Scolytidae in the British Museum. 168. Contribution to the morphology and taxonomy of the Scolytoidea The Annals and Magazine of Natural History, series 13, 1: 557–560. 10.1080/00222935808650982

[B392] Schedl KE (1958b) Bark and timber beetles from Malaya. Malayan Forester 21: 99–105.

[B393] Schedl KE (1958c) Zur Synonymie der Borkenkäfer II. Tijdschrift voor Entomologie 101: 141–155.

[B394] Schedl KE (1959) A checklist of the Scolytidae and Platypodidae (Coleoptera) of Ceylon with descriptions of new species and biological notes. Transactions of the Royal Entomological Society of London 111: 469–534. 10.1111/j.1365-2311.1959.tb02874.x

[B395] Schedl KE (1960a) Synonymies of bark beetles (Scolytidae), IV. 174. Contribution to the morphology and taxonomy of the Scolytoidea. The Coleopterists Bulletin 14: 5–12.

[B396] Schedl KE (1960b) Zur Synonymie der Borkenkäfer, V. 181. Beitrag zur Morphologie und Systematik der Scolytoidea. Entomologische Blätter 56: 103–112.

[B397] Schedl KE (1962a) Zur Synonymie der Borkenkäfer VI. 203. Beitrag zur Morphologie und Systematik der Scolytoidea. Entomologische Blätter 58: 201–211.

[B398] Schedl KE (1962b) Synonymies of bark beetles VII. 204. Contribution to the morphology and taxonomy of the Scolytoidea Annals and Magazine of Natural History, series 13, 4: 697–699. 10.1080/00222936108651195

[B399] Schedl KE (1963a) Scolytidae und Platypodidae Afrikas. Band II. Familie Scolytidae (Fortsetzung), Unterfamilie Ipinae (Fortsetzung). Revista de Entomologia de Moçambique 5: 1–594.

[B400] Schedl KE (1963b) Zur Synonymie der Borkenkäfer IX. 209. Beitrag zur Morphologie und Systematik der Scolytoidea. Entomologische Abhandlungen und Berichte aus dem Staatlichen Museum für Tierkunde in Dresden 28: 257–268.

[B401] Schedl KE (1964a) Neue und interessante Scolytoidea von den Sunda-Inseln, Neu Guinea und Australien. 202. Beitrag zur Morphologie und Systematik der Scolytoidea. Tijdschrift voor Entomologie 107: 297–306.

[B402] Schedl KE (1964b) Scolytoidea from Borneo III. 185. Contribution to the morphology and taxonomy of the Scolytoidea. Reichenbachia 4: 241–254.

[B403] Schedl KE (1964c) Zur Synonymie der Borkenkäfer XIV. 223. Beitrag zur Morphologie und Systematik der Scolytoidea. Reichenbachia 2: 303–317.

[B404] Schedl KE (1964d) Zur Synonymie der Borkenkäfer XV. 228. Beitrag zur Morphologie und Systematik der Scolytoidea. Reichenbachia 3: 303–317.

[B405] Schedl KE (1965) Scolytidae und Platypodidae aus dem Naturhistorika Riksmuseum in Stockholm. 235. Beitrag zur Morphologie und Systematik der Scolytoidea. Arkiv för Zoologi 18: 17–31.

[B406] Schedl KE (1966a) Check List of the Scolytidae and Platypodidae from the Philippine Islands. 196. Contribution to the morphology and taxonomy of the Scolytoidea. Entomologische Abhandlungen 35: 1–122.

[B407] Schedl KE (1966b) Pin-hole borers and bark-beetles (Scolytidae and Platypodidae) intercepted from imported logs in Japanese ports. 241. Contribution to the morphology and taxonomy of the Scolytoidea. Kontyû 34: 29–43.

[B408] Schedl KE (1967) Zur Synonymie der Borkenkäfer, XVI. 242. Beitrag zur Morphologie und Systematik der Scolytoidea. Entomologisk Tidskrift 88: 146–163.

[B409] Schedl KE (1969a) Bark-beetles and pin-hole borers (Scolytidae and Platypodidae) intercepted from imported logs in Japanese ports, III. 258. Contribution to the morphology and taxonomy of the Scolytoidea. Kontyû 37: 202–219.

[B410] Schedl KE (1969b) Indian bark and timber beetles, V. 217. Contribution to the morphology and taxonomy of the Scolytoidea. Oriental Insects 3: 47–70. 10.1080/00305316.1969.10433895

[B411] Schedl KE (1970a) Another collection of Scolytidae and Platypodidae of economic importance from the territory of Papua and New Guinea. 254. Contribution to the morphology and taxonomy of the Scolytoidea. Proceedings of the Linnean Society of New South Wales 94: 128–132.

[B412] Schedl KE (1970b) Zur Synonymie der Borkenkäfer, XX. Annalen des Naturhistorisches Museum Wien 74: 221–231.

[B413] Schedl KE (1971a) Coleoptera: Scolytidae and Platypodidae from Ceylon. Entomologica Scandinavica Supplementum 1: 274–285.

[B414] Schedl KE (1971b) Indomalayan bark and timber beetles. 273. Contribution to the morphology and taxonomy of the Scolytoidea. Oriental Insects 5: 361–399. 10.1080/00305316.1971.10434023

[B415] Schedl KE (1972a) New Scolytidae and Platypodidae from the Papuan subregion and Australia. 279. Contribution to the morphology and taxonomy of the Scolytoidea. Papua New Guinea Agricultural Journal 23: 61–72.

[B416] Schedl KE (1972b) Zur Synonymie der Borkenkäfer XXI. 281. Beitrag zur Morphologie und Systematik der Scolytoidea. Entomologische Arbeiten aus dem Museum G. Frey 23: 150–161.

[B417] Schedl KE (1973) New Scolytidae and Platypodidae from the Papuan subregion. 299. Contribution to the morphology and taxonomy of the Scolytoidea. Papua New Guinea Agricultural Journal 24: 87–97.

[B418] Schedl KE (1974) Borken- und ambrosiakäfer aus Vietnam (IV). 298. Beitrag zur Morphologie und Systematik der Scolytoidea. Travaux du Museum d’Histoire Naturelle Gregoire Antipa, Bukarest 14: 261–266.

[B419] Schedl KE (1975a) Bark and timber beetles of the Oriental region. 319. Contribution to the morphology and taxonomy of the Scolytoidea. Oriental Insects 9: 451–460. 10.1080/00305316.1975.10434514

[B420] Schedl KE (1975b) Indian bark and timber beetles VI. 312. Contribution to the morphology and taxonomy of the Scolytoidea. Revue Suisse de Zoologie 82: 445–458. 10.5962/bhl.part.78268

[B421] Schedl KE (1975c) New Scolytidae and Platypodidae from Papua and New Guinea, IV. 317. Contribution to the morphology and taxonomy of the Scolytoidea. Annalen des Naturhistorisches Museum, Wien 79: 337–399.

[B422] Schedl KE (1975d) New Scolytidae and Platypodidae from Papua/New Guinea (Coleoptera). 315. Contribution to the morphology and taxonomy of the Scolytoidea. Reichenbachia 15: 215–232.

[B423] Schedl KE (1975e) Zur Synonymie der Borkenkäfer, XXVI. 318. Beitrag zur Morphologie und Systematik der Scolytoidea. Zeitschrift der Arbeitsgemeinschaft Österreichischer Entomologen 27: 33–38.

[B424] Schedl KE (1976) Neotropische Scolytoidea, XIII. Faunistische Abhandlungen, Staatlichen Museum für Tierkunde in Dresden 41: 49–92.

[B425] Schedl KE (1977) Some new bark beetles from the Indomalayan region. 332. Contribution to the morphology and taxonomy of the Scolytoidea. Oriental Insects 11: 490–504. 10.1080/00305316.1977.11090920

[B426] Schedl KE (1979a) Bark and timber beetles from Australia. 326. Contribution. Entomologischen Arbeiten aus dem Museum G. Frey 28: 157–164.

[B427] Schedl KE (1979b) New Scolytidae and Platypodidae from Papua New Guinea (V). 311^th^ Contribution to the morphology and taxonomy of the Scolytoidea. Faunistische Abhandlungen, Staatlichen Museum für Tierkunde in Dresden 7: 95–120.

[B428] Schedl KE (1980) Zur Synonymie der Borkenkäfer XXVIII. 339. Beitrag zur Morphologie und Systematik der Scolytoidea. Zeitschrift der Arbeitsgemeinschaft Österreichischer Entomologen 31: 117–124.

[B429] Schiefer TL Bright DE (2004) *Xylosandrus mutilatus* (Blandford), an exotic ambrosia beetle (Coleoptera: Curculionidae: Scolytinae: Xyleborini) new to North America. The Coleopterists Bulletin 58: 431–438. 10.1649/760

[B430] Schiefer TL (2018) First record of the introduced ambrosia beetle *Ambrosiophilus nodulosus* (Eggers) in Mississippi, with notes on the distribution of *Ambrosiodmus minor* (Stebbing) (Coleoptera: Curculionidae: Scolytinae). The Coleopterists Bulletin 72: 384–385. 10.1649/0010-065X-72.2.384

[B431] Schneider I (1987) Verbreitung, Pilzübertragung und Brutsystem des Ambrosiakäfers *Xyleborus affinis* im vergleich mit *X. mascarensis* (Coleoptera: Scolytidae) Entomologia Generalis 12: 267–275. 10.1127/entom.gen/12/1987/267

[B432] Sharp D (1885) Memoirs on the Coleoptera of the Hawaiian Islands. In: Blackburn T Sharp D (Eds) Memoirs on the Coleoptera of the Hawaiian Islands.The Scientific Transactions of the Royal Dublin Society 3: 119–300. [pls. IV–V.]

[B433] Sittichaya W (2012) Bark and ambrosia beetles (Coleoptera: Curculionidae: Scolytinae and Platypodinae) infesting mango trees (*Mangifera indica* L.) in Southern Thailand, with new species recorded for Thailand. Songklanakarin Journal of Science and Technology 34: 153–155.

[B434] Sittichaya W Beaver RA (2018) *Cnestus quadrispinosus*, a new species of xyleborine ambrosia beetle from Thailand and Borneo (Coleoptera, Curculionidae, Scolytinae, Xyleborini). ZooKeys 795: 31–37. 10.3897/zookeys.795.28384 PMC6232242 30429655

[B435] Sittichaya W Smith SM Beaver RA (2019) Ten newly recorded species of xyleborine ambrosia beetles (Coleoptera, Curculionidae, Scolytinae, Xyleborini) from Thailand. ZooKeys 862: 109–127. 10.3897/zookeys.862.34766 31341388 PMC6635385

[B436] Six DL Stone WD de Beer ZW Woolfolk SW (2009) *Ambrosiella beaveri* sp. nov., associated with an exotic ambrosia beetle, *Xylosandrus mutilatus* (Coleoptera: Curculionidae, Scolytinae) in Mississippi, USA. Antonie van Leeuwenhoek 96: 17–29. 10.1007/s10482-009-9331-x 19319658

[B437] Smith SM (2017) *Dinoxyleborus* Smith, a new genus of Neotropical xyleborine ambrosia beetle (Coleoptera, Curculionidae: Scolytinae). Zootaxa 4303: 131–139. 10.11646/zootaxa.4303.1.8

[B438] Smith SM Cognato AI (2015) *Ambrosiophilus peregrinus* n. sp., an exotic ambrosia beetle discovered in Georgia, U.S.A. (Coleoptera: Curculionidae: Scolytinae). The Coleopterists Bulletin 69: 213–220. 10.1649/0010-065X-69.2.213

[B439] Smith SM Petrov AV Cognato AI (2017a) Beetles (Coleoptera) of Peru: A survey of the Families. Curculionidae: Scolytinae. The Coleopterists Bulletin 71: 77–94. 10.1649/0010-065X-71.1.77

[B440] Smith SM Beaver RA Cognato AI (2017b) The ambrosia beetle *Ambrosiophilus peregrinus*, introduced to the United States, is *Ambrosiophilus nodulosus* (n. comb.) (Curculionidae: Scolytinae). The Coleopterists Bulletin 71: 552–553. 10.1649/0010-065X-71.3.552

[B441] Smith SM Beaver RA Singh S Cognato AI (2018a) Taxonomic clarification and neotype designation for three Indian xyleborine species (Coleoptera: Curculionidae: Scolytinae). Zootaxa 4394: 138–140. 10.11646/zootaxa.4394.1.9 29690388

[B442] Smith SM Beaver RA Cognato AI (2018b) New synonymy, new combinations and taxonomic changes in Japanese xyleborine ambrosia beetles (Coleoptera: Curculionidae: Scolytinae). Zootaxa 4521: 391–403. 10.11646/zootaxa.4521.3.5 30486154

[B443] Smith SM Rabaglia RJ Beaver RA Thu PQ Cognato AI (2018c) Attraction of ambrosia beetles (Curculionidae: Scolytinae) to semiochemicals in Vietnam with new records and a new species. The Coleopterists Bulletin 72: 838–844. 10.1649/0010-065X-72.4.838

[B444] Smith SM Beaver RA Cognato AI Hulcr J Redford AJ (2019a) Southeast Asian Ambrosia Beetle ID. USDA APHIS Identification Technology Program (ITP) and Michigan State University. Fort Collins, CO. http://idtools.org/id/wbb/sea-ambrosia/

[B445] Smith SM Gomez DF Beaver RA Hulcr J Cognato AI (2019b) Reassessment of the species in the *Euwallacea fornicatus* (Coleoptera: Curculionidae: Scolytinae) complex after the rediscovery of the “lost” type specimen. Insects 10: 1–261. 10.3390/insects10090261 PMC6780773 31443370

[B446] Smith SM Beaver RA Cognato AI (2020) Taxonomic changes for Indo-Malayan ambrosia beetles (Coleoptera: Curculionidae: Scolytinae: Xyleborini). The Coleopterists Bulletin 74: 37–41. 10.1649/0010-065X-74.1.37

[B447] Speranza S Bucini D Paparatti B (2009) New observation on biology of European shot-hole borer [*Xyleborus dispar* (F.)] on hazel in northern Latium (Central Italy). Acta Horticulturae 845: 539–542. 10.17660/ActaHortic.2009.845.84

[B448] Sreedharan K Balakrishnan M Sanuel S Bhat P (1991) A note on the association of wood boring beetles and a fungus with the death of silver oak trees on coffee plantations. Journal of Coffee Research 21: 145–148.

[B449] Stebbing EP (1907) On some Assam sal (*Shorea robusta*) insect pests, with notes upon some insects predaceous and parasitic upon them. Indian Forest Bulletin 11: 1–66.

[B450] Stebbing EP (1908) On some undescribed Scolytidae of economic importance from the Indian Region I. Indian Forest Memoirs 1: 1–12.

[B451] Stebbing EP (1909) On some undescribed Scolytidae of economic importance from the Indian Region, II. Indian Forest Memoirs 1: 13–32.

[B452] Stebbing EP (1914) Indian Forest Insects of Economic Importance. Eyre & Spottiswoode, London, 648 pp 10.5962/bhl.title.9203

[B453] Stone WD Nebeker TE (2007) Distribution and seasonal abundance of *Xylosandrus mutilatus* (Coleoptera: Curculionidae). Journal of Entomological Science 42: 409–412. 10.18474/0749-8004-42.3.409

[B454] Stone WD Nebeker TE Gerard PD (2007) Host plants of *Xylosandrus mutilatus* in Mississippi. Florida Entomologist 90: 191–195. 10.1653/0015-4040(2007)90[191:HPOXMI]2.0.CO;2

[B455] Storer CG Breinholt JW Hulcr J (2015) *Wallacellus* is *Euwallacea*: molecular phylogenetics settles generic relationships (Coleoptera: Curculionidae: Scolytinae: Xyleborini). Zootaxa 3974: 391–400. 10.11646/zootaxa.3974.3.6 26249912

[B456] Stouthamer R Rugman-Jones P Thu PQ Eskalen A Thibault T Hulcr J Wang L-J Jordal BH Chen C-Y Cooperband M Lin C-S Kamata N Lu S-S Masuya H Mendel Z Rabaglia R Sanguansub S Shih H-H Sittichaya W Zong S (2017) Tracing the origin of a cryptic invader: phylogeography of the *Euwallacea fornicatus* (Coleoptera: Curculionidae: Scolytinae) species complex. Agricultural and Forest Entomology 19: 366–375. 10.1111/afe.12215

[B457] Strohmeyer H (1910) Ueber Kaffeeschädlinge auf der Insel Java. Entomologische Blätter 6: 186–187.

[B458] Strohmeyer H (1912) H. Sauter’s Formosa-Ausbeute. Ipidae und Platypodidae. Entomologische Mitteilungen 1: 38–42. 10.5962/bhl.part.25902

[B459] Swaine JM (1918) Canadian bark beetles. Part II. A preliminary classification, with an account of the habits and means of control. Dominion of Canada Department of Agriculture, Entomological Branch, Technical Bulletin 14: 1–143. 10.5962/bhl.title.27867

[B460] Swaine JM (1934) Three new species of Scolytidae (Coleoptera). The Canadian Entomologist 66: 204–206. 10.4039/Ent66204-9

[B461] Tang W-Q (2000) Biological characteristics of *Xyleborus mutilatus* and its control. Journal of Zhejiang Forestry College 17: 417–420.

[B462] Terekhova VV Skrylnik YuYe (2012) Biological peculiarities of the alien for Europe *Anisandrus maiche* Stark (Coleoptera: Curculionidae: Scolytinae) bark beetle in Ukraine. Russian Journal of Biological Invasions 3: 139–144. 10.1134/S2075111712020105

[B463] Vandenberg NJ Rabaglia RJ Bright DE (2000) New records of two *Xyleborus* (Coleoptera: Scolytidae) in North America. Proceedings of the Entomological Society of Washington 102: 62–68.

[B464] Veen H (1897) Description of a new species of the genus *Tomicus* (Coleoptera: Scolytidae). Notes from the Leyden Museum 19: 135–136.

[B465] Walker F (1859) Characters of some apparently undescribed Ceylon insects. The Annals and Magazine of Natural History, series 3, 3: 258–265. 10.1080/00222935908697111

[B466] Wang J Smith SM Cognato AI (2020) *Immanus virago*, a new species from Borneo (Coleoptera, Curculionidae: Scolytinae: Xyleborini). The Coleopterists Bulletin 74: 438–440. 10.1649/0010-065X-74.2.438

[B467] Weber BC McPherson JE (1983a) Life history of the ambrosia beetle *Xylosandrus germanus* (Coleoptera: Scolytidae). Annals of the Entomological Society of America 76: 455–462. 10.1093/aesa/76.3.455

[B468] Weber BC McPherson JE (1983b) World list of host plants of *Xylosandrus germanus* (Coleoptera: Scolytidae). The Coleopterists Bulletin 37: 114–134.

[B469] Wichmann HE (1914) XXX Coleoptera, VII: Ipiden und Platypodiden In: Zoological results of the Abor Expedition, 1911-1912. Records of the Indian Museum 8: 411–414. 10.5962/bhl.part.1194

[B470] Wollaston TV (1854) Insecta Maderensia; Being an Account of the Insects of the Islands of the Madeiran Group. John van Voorst, London, 634 pp [13 pls.] 10.5962/bhl.title.140700

[B471] Wollaston TV (1857) Catalogue of the Coleopterous Insects of Madeira in the Collection of the British Museum, London, 234 pp. [1 pl.] 10.5962/bhl.title.9900

[B472] Wollaston TV (1867) Coleoptera Hesperidum; being an enumeration of the Coleopterous insects of the Cape Verde archipelago. J. van Voorst, London, 285 pp [1 map.] 10.5962/bhl.title.48651

[B473] Wood SL (1957) Distributional notes on and synonymies of some North American Scolytidae (Coleoptera). The Canadian Entomologist 89: 396–403. 10.4039/Ent89396-9

[B474] Wood SL (1960) Coleoptera: Platypodidae and Scolytidae. Insects of Micronesia 18: 1–73.

[B475] Wood SL (1962) Miscellaneous taxonomic notes on Scolytidae (Coleoptera). The Great Basin Naturalist 21: 87–107. 10.5962/bhl.part.11210

[B476] Wood SL (1969) New synonymy and records of Platypodidae and Scolytidae (Coleoptera). The Great Basin Naturalist 29: 113–128. 10.5962/bhl.part.17054

[B477] Wood SL (1972) New synonymy in American bark beetles (Scolytidae: Coleoptera), Part II. Great Basin Naturalist 32: 190–201. 10.5962/bhl.part.25774

[B478] Wood SL (1974) New synonymy and records of American bark beetles (Coleoptera: Scolytidae). The Great Basin Naturalist 34: 277–290. 10.5962/bhl.part.15523

[B479] Wood SL (1975a) New synonymy and new species of American bark beetles (Coleoptera: Scolytidae). The Great Basin Naturalist 35: 21–32.

[B480] Wood SL (1975b) New synonymy and new species of American bark beetles (Coleoptera: Scolytidae) Part II. The Great Basin Naturalist 35: 391–401.

[B481] Wood SL (1977) Introduced and exported American Scolytidae (Coleoptera). The Great Basin Naturalist 37: 67–74.

[B482] Wood SL (1980) New genera and new generic synonymy in Scolytidae (Coleoptera). The Great Basin Naturalist 40: 89–97. 10.5962/bhl.part.20006

[B483] Wood SL (1982) The bark and ambrosia beetles of North and Central America (Coleoptera: Scolytidae), a taxonomic monograph. Great Basin Naturalist Memoirs 8: 1–1359.

[B484] Wood SL (1983) New synonymy and new species of American bark beetles (Coleoptera: Scolytidae), Part IX. The Great Basin Naturalist 43: 647–659. 10.5962/bhl.part.4489

[B485] Wood SL (1984) New generic synonymy and new genera of Scolytidae (Coleoptera). The Great Basin Naturalist 44: 223–230.

[B486] Wood SL (1986) A reclassification of the genera of Scolytidae (Coleoptera). Great Basin Naturalist Memoirs 10: 1–126.

[B487] Wood SL (1989) Nomenclatural changes and new species of Scolytidae (Coleoptera), part IV. The Great Basin Naturalist 49: 167–185. 10.5962/bhl.part.22642

[B488] Wood SL (1992) Nomenclatural changes and new species of Platypodidae and Scolytidae (Coleoptera), part II. The Great Basin Naturalist 52: 78–88.

[B489] Wood SL (2007) Bark and Ambrosia Beetles of South America (Coleoptera: Scolytidae). Brigham Young University, M.L. Bean Life Science Museum, Provo, 900 pp. [230 plates.]

[B490] Wood SL Bright DE (1992) A catalog of Scolytidae and Platypodidae (Coleoptera), Part 2: Taxonomic index. The Great Basin Naturalist Memoirs 13: 1–1553.

[B491] Wurth T (1908) De boeboek (*Xyleborus coffeae* n. sp.) op *Coffea robusta*. Mededeelingen van het Algemeen-Proefstation op Java te Salatiga (2) 3: 1–20. [pls. i–iii.]

[B492] Yeates DK Seago A Nelson L Cameron SL Joseph L Trueman JWH (2011) Integrative taxonomy, or iterative taxonomy? Systematic Entomology 36: 209–217. 10.1111/j.1365-3113.2010.00558.x

[B493] Yin H-F Huang F-S Li Z-L (1984) Economic insect fauna of China. Fasc. 29. Coleoptera: Scolytidae. Science Press, Beijing, 205 pp. [XVII Tab.]

[B494] Zheng S Li Y Wang S (2017) Identification and distribution of *Xyleborinus artestriatus* Eichhoff 1878 (Coleoptera: Curculionidae: Scolytinae) in China. Journal of Environmental Entomology 40: 1445–1450. (Chinese with English abstract).

[B495] Zimmermann C (1868) Synopsis of the Scolytidae of America north of Mexico. Transactions of the American Entomological Society 2: 141–149. 10.2307/25076203

